# Modern Treatment of Pulmonary Embolism (USCDT vs MT): Results From a Real-World, Big Data Analysis (REAL-PE)

**DOI:** 10.1016/j.jscai.2023.101192

**Published:** 2023-10-24

**Authors:** Peter Monteleone, Ryan Ahern, Subhash Banerjee, Kush R. Desai, Daniella Kadian-Dodov, Emily Webber, Sally Omidvar, Patrick Troy, Sahil A. Parikh

**Affiliations:** a The University of Texas at Austin Dell School of Medicine, Ascension Texas Cardiovascular, Austin, Texas; b Truveta, Inc, Bellevue, Washington; c University of Washington, Seattle, Washington; d Baylor University Medical Center, Dallas, Texas; e Northwestern University Feinberg School of Medicine, Chicago, Illinois; f Mt Sinai School of Medicine, New York, New York; g Hartford Hospital, University of Connecticut School of Medicine, Hartford, Connecticut; h Center for Interventional Cardiovascular Care and Division of Cardiology, Department of Medicine, Columbia University Irving Medical Center, New York, New York

**Keywords:** bleeding, pulmonary embolism, thrombectomy, thrombolysis

## Abstract

**Background:**

Advanced therapies are increasingly utilized to treat pulmonary embolism (PE). A unique data platform allows access to electronic health record data for comparison of the safety of PE therapies.

**Methods:**

All data from Truveta (Truveta, Inc) were analyzed (16 systems, 83,612,413 patients, 535,567 with PE). All patients treated with ultrasound-assisted catheter-directed thrombolysis (USCDT) (Boston Scientific) or mechanical thrombectomy (MT) (Inari Medical) for PE were identified. The primary analysis was based on index procedures performed from January 2009 to May 2023, and contemporary analysis on those performed from January 2018 to May 2023. Bleeding was assessed via direct laboratory analysis and transfusion administration documentation. International Society for Thrombosis and Hemostasis (ISTH) and Bleeding Academic Research Consortium (BARC) 3b definitions were recreated. Multiple logistic regression analysis of major bleeding was performed. In-hospital death and median length of stay were measured.

**Results:**

For the primary analysis, 2259 patients (N = 1577 USCDT, N = 682 MT) and for the contemporary analysis 1798 patients (N = 1137 USCDT, N = 661 MT) met the criteria. Incidence of hemoglobin reduction (>2 and >5 g/dL) and transfusions received were significantly higher among MT-treated patients in both analyses, as was ISTH and BARC 3b major bleeding (primary: ISTH MT 17.3% vs USCDT 12.4% *P* = .002; BARC 3b MT 15.4% vs USCDT 11.8% *P* = .019) (contemporary: ISTH MT 17.2% vs USCDT 11.0% *P* = .0002; BARC 3b MT 15.4% vs USCDT 10.6% *P* = .002). Regression analysis demonstrated that MT is associated with major bleeding. Median length of stay, all-cause 30-day readmission and in-hospital mortality were similar between groups. Intracranial hemorrhage was more common with MT.

**Conclusions:**

Major bleeding derived from direct laboratory and transfusion data occurred more frequently with MT vs USCDT. Intracranial hemorrhage was more common among MT-treated patients. In the absence of randomized data, these results provide guidance regarding the bleeding risk and safety of strategies for advanced PE therapy.

## Highlights

• Novel big data tools can transform real-time analysis of technology implementation.
• Ultrasound-assisted catheter-directed thrombolysis for pulmonary embolism results in a lower risk of bleeding than mechanical thrombectomy in real-world experience.
• Bleeding was assessed through direct laboratory measurement and transfusion records.
• No differences are demonstrated between length of stay and readmission between mechanical thrombectomy and ultrasound-assisted catheter-directed thrombolysis.

## Central Illustration

**Figure:**
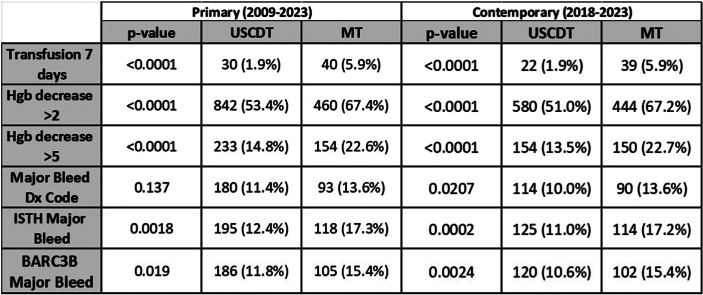

## Introduction

Pulmonary embolism (PE) is estimated to result in approximately 100,000 annual deaths in the United States with 30-day and 1-year mortality of approximately 4% and 13%, respectively.^1^^,^^2^ Historic initial treatment of PE was limited to anticoagulation alone and systemic thrombolytic therapy for emergent life-threatening PE. Risk stratification of PE progressed to allow the targeting of therapies. Measurement of the ratio of right ventricle (RV) compared with left ventricle (LV) enlargement in PE has been associated with a 3- to 4-fold increase in in-hospital mortality with step-wise mortality increase based on severity of enlargement.^3^^,^^4^ Improved categorization of the severity of illness with risk stratification to low, intermediate-low, intermediate-high, and high risk have been presented in the ESC 2019 guidelines.^5^ Novel therapies including ultrasound-assisted catheter-directed thrombolysis (USCDT) and mechanical thrombectomy (MT) have been developed to address the increased morbidity and mortality of elevated-risk PE.

The EkoSonic Endovascular System (EKOS, Boston Scientific) is a device that facilitates USCDT.^6^^,^^7^ EKOS was developed and first approved by the United States Food and Drug Administration (FDA) in 2004 and remains to date the only FDA-approved device for USCDT.^8^ ULTIMA, a multicenter randomized trial in 2014, demonstrated a reduction in RV/LV ratio at 24 hours with no increase in adverse safety events when compared to standard parenteral anticoagulation.^9^ The therapy delivered by EKOS was refined in the OPTALYSE PE multicenter, prospective trial in 2018 which demonstrated the effectiveness of lower doses and shorter infusion durations or thrombolytic therapy.^10^ In 2021, the KNOCOUT-PE registry demonstrated routine adoption of these low protocols.^11^ In 1000 retrospective and 500 prospective EKOS-treated patients, the mean tPA dose was 17.9 mg and the mean duration was 10.4 hours with 32.4% of patients receiving ≤12 mg. There were no intracerebral hemorrhagic events.

The FlowTriever system (Inari Medical) is a large-bore syringe-based suction and MT catheter that can aspirate thrombus with negative pressure. The FlowTriever Pulmonary Embolectomy Clinical Study (FLARE) trial was a prospective, single-arm, multicenter clinical trial that examined patients with intermediate-risk PE and reported RV/LV ratio improvement at 48 hours with no device-related deaths and 1 major bleed in 106 treated patients.^12^ In 2020, the FLASH registry evaluated FlowTriever treatment of 500 acute PE patients and reported 0% mortality at 48 hours with only 3 non-intracranial hemorrhage (ICH) major bleeds and 1 access site complication.^13^ Other MT devices have been developed and studied in PE and venous thromboembolism with the Indigo Aspiration System (Penumbra, Inc) and the AngioVac devices (AngioDynamics) having received FDA approval.^14^^,^^15^

Despite promising clinical data, no randomized prospective data exists beyond ULTIMA and no class 1 indication for catheter-based approaches has been updated within professional societal guidelines.^5^^,^^16^^,^^17^ Variations in reported outcomes including bleeding definitions have been used between trials and between therapies.^18^

Novel large data analytics platforms may help rapidly answer questions that remain unanswered in health care. One novel platform was utilized to determine the current outcomes of 2 common advanced therapies for the management of patients with serious PE.

## Methods

All data from Truveta (Truveta, Inc), an electronic health record (EHR)-based platform including 16 United States health systems were analyzed. This included deidentified, up-to-date EHR data including 83,612,413 patients as of May 2023. The primary analysis was based on all available data (index procedures from January 2009 through May 2023). A contemporary analysis was also performed (index procedures from January 2018 through May 2023). The goal of this distinct contemporary analysis was to address advances in technology, technique, and physician expertise. The contemporary analysis therefore compares MT to USCDT during the current era of experienced practice. Patients who underwent a procedure using the EKOS or FlowTriever device for the treatment of PE were identified using a Unique Device Identifier. Device data were mapped by trained medical annotators from free text or procedure and device ontologies, such as Current Procedural Terminology (CPT) and Unique Device Identifier respectively, to Truveta codes, which were used to identify patients who had procedures with either device.

**Figure 1:**
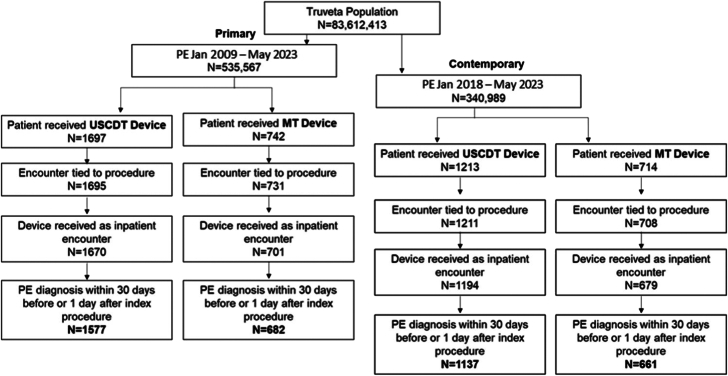
**Patient flowchart for inclusion in the primary and contemporary cohorts**.

Only patients with a PE diagnosis in the EHR within 30 days before the date of the USCDT or MT procedure or up to 1 day after the procedure were included in the analysis. The inclusion of 1 day following the procedure was performed to account for a potential short delay in diagnoses being populated within the EHR. Specific codes from standard ontologies (ICD-10, RxNorm, SNOMED-CT, LOINC) used in data definitions are provided in the Supplemental Appendix. Only patients who underwent the USCDT or MT procedure during an inpatient encounter or who were admitted for inpatient care within 24 hours after the procedure (ie, in situations where the emergency room or procedural department was coded as an outpatient department) were included. (Figure 1) Truveta data has been validated through processes measuring the completeness and accuracy of the data.

Adverse events were recorded within 7 days of the index procedure to mimic the event reporting of contemporary PE clinical trials. A decrease in hemoglobin levels was assessed directly via the capture of laboratory results. The change in hemoglobin is calculated by subtracting the nadir hemoglobin level (0-7 days postprocedure) from the preprocedure maximal level. Transfusion performance was identified through CPT documentation of transfusion.

The International Society for Thrombosis and Hemostasis (ISTH)-modeled major hemorrhage definition includes patients who had an ICD-10 or SNOMED code related to major bleed, or if they had both a blood transfusion (CPT: “36430”) and a corresponding decrease in lab value hemoglobin level (LOIN-C: “30350-3) of ≥2 g/dL.^19^ A separate major bleeding analysis was reported to approximate the Bleeding Academic Research Consortium (BARC) 3b bleeding utilized in prior trials.^20^ This BARC 3b-modeled major bleeding definition included patients who had a ≥5 g/dL decrease in hemoglobin (calculated by the identical method described above) regardless of whether patients had a transfusion. Patient records were reviewed to evaluate for a discharge disposition of death in the EHR defined as an inpatient mortality event. Other adverse events are classified using standard ontologies for diseases and can be found in the condition tables in the Supplemental Appendix.

Descriptive and inferential statistical analyses were conducted to elucidate differences among the USCDT and MT device groups. χ^2^ tests for independence were conducted using the R chisq.test. Several multivariable logistic regression analyses were conducted using the R glm statistical function to predict the risk of adverse events while controlling for the influence of patient demographics, clinical characteristics, and treatment received. A 2-sided *P* value of 0.05 was used to determine statistical significance. Regression analyses were conducted to better understand the relative contribution of multiple clinical variables in predicting key outcomes. Only adverse events that included at least 5% of patients (primary analysis: 113 events; contemporary analysis: 90 events) from the outcome of interest groups were included in the regression. Explanatory dependent variables were selected from the comorbidities and demographic features examined for this analysis.

Postprocedure length of stay (LOS) is calculated as the procedural start time to the end of the inpatient encounter. Patients without an end time value were excluded (excluded subjects for LOS analysis: Primary: USCDT = 410, MT = 277; Contemporary: USCDT = 347, MT = 265). Median LOS distributions are calculated using a Wilcoxon rank sum test with continuity correction. Patients were classified as readmitted if the EHR documented an inpatient encounter within a participating facility within 30 days of the end of the original inpatient encounter. The risk of ischemic stroke and intracerebral hemorrhage were derived from coding data. Death during inpatient admission was abstracted from the EHR.

## Results

For the primary analysis, 2259 patients (N = 1577 USCDT, N = 682 MT) and for the contemporary analysis 1798 patients (N = 1137 USCDT, N = 661 MT) met the criteria. Demographics of all patients diagnosed with PE from both cohorts are provided (Table 1). The MT group in the primary analyses had a greater proportion of females (MT 47.8% vs USCDT 43.4%, *P* = .04), a finding not noted in the contemporary analysis (MT 47.7% vs USCDT 44.2% *P* = .121). The MT groups had a greater proportion of patients aged ≥60 years (primary: MT 63.5% vs 55.6% *P* < .001; contemporary: MT 63.7% vs USCDT 57.2 *P* = .004) and a greater proportion of patients with a history of cancer (primary: MT 21.4% vs USCDT 15.2% *P* < .001; contemporary: MT 20.7% vs USCDT 15.0% *P* = .002).

**Table 1:** Demographics, medical history, and preceding anticoagulant use of analyzed patient cohorts.

	Primary (2009-2023)			Contemporary (2018-2023)		
	*P* value	USCDT	MT	*P* value	USCDT	MT
Female sex	.040	684 (43.4%)	326 (47.8%)	.121	502 (44.2%)	315 (47.7%)
Age ≥60 y	<.001	877 (55.6%)	433 (63.5%)	.004	650 (57.2%)	421 (63.7%)
Race						
White	.139	1295 (82.1%)	542 (79.5%)	.836	906 (79.7%)	524 (79.3%)
Black	.121	183 (11.6%)	64 (9.4%)	.009	154 (13.5%)	62 (9.4%)
Other	.866	27 (1.7%)	11 (1.6%)	.882	20 (1.8%)	11 (1.7%)
Hispanic or Latino ethnicity	<.001	55 (3.5%)	55 (8.1%)	<.001	49 (4.3%)	56 (8.5%)
Medical history						
Cancer	<.001	240 (15.2%)	146 (21.4%)	.002	171 (15.0%)	137 (20.7%)
Chronic kidney disease	.938	138 (8.8%)	59 (8.7%)	.48	104 (9.1%)	54 (8.2%)
Chronic obstructive pulmonary disease	.434	99 (6.3%)	37 (5.4%)	.629	56 (4.9%)	36 (5.4%)
Coronary artery disease	.854	167 (10.6%)	74 (10.9%)	.521	113 (9.9%)	72 (10.9%)
Hypertension	.870	704 (44.6%)	307 (45.0%)	.676	519 (45.6%)	295 (44.6%)
Deep vein thrombosis	.180	306 (19.4%)	116 (17.0%)	.946	182 (16.0%)	105 (15.9%)
Ischemic stroke	.910	54 (3.4%)	24 (3.5%)	.901	40 (3.5%)	24 (3.6%)
Prior myocardial infarction	.788	88 (5.6%)	40 (5.9%)	.691	62 (5.5%)	39 (5.9)
Diabetes mellitus	.713	283 (17.9%)	118 (17.3%)	.600	202 (17.8%)	111 (16.8%)
Anticoagulant use:						
Direct oral anticoagulants	.054	261 (16.6%)	91 (13.3%)	.032	193 (17.0%)	87 (13.2%)
Vitamin K antagonists	.069	52 (3.3%)	13 (1.9%)	.797	19 (1.7%)	10 (1.5%)
Unfractionated heparin	.928	1401 (88.8%)	605 (88.7%)	.562	1018 (89.5%)	586 (88.7%)
Low-molecular-weight heparin	.124	404 (25.6%)	154 (22.6%)	.131	296 (26.0%)	151 (22.8%)

MT, mechanical thrombectomy; USCDT, ultrasound-assisted catheter-directed thrombolysis.

There were no significant differences in the incidence of prior stroke, prior anticoagulant use within 30 days, chronic kidney disease, prior myocardial infarction, or other factors as recorded in Table 1.

Anticoagulant usage prior to and within 30 days of the procedure was reported (Table 1). There were no significant differences between groups in the use of vitamin K antagonists, unfractionated heparin, or low molecular weight heparin. There was a statistically significant difference with increased use of direct oral anticoagulant in the 30 days prior to the procedure in patients treated with USCDT in the contemporary analysis.

In both analyses, treatment with MT vs USCDT was associated with an increased risk of both a decrease in hemoglobin >2 g/dL and >5 g/dL (Central Illustration). In both the primary and contemporary analyses, MT treatment was associated with an increased need for a blood transfusion (Central Illustration). Coding for bleeding events (see Supplemental Appendix for full list) was also associated with MT treatment in the contemporary analysis (Central Illustration). ISTH major bleeding and BARC 3b major bleeding were associated with treatment with MT in both the primary and the contemporary analyses (Central Illustration).

**Central Illustration:**
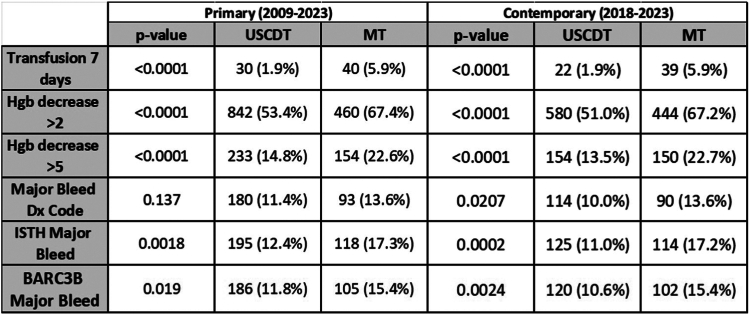
Assessed components of comparative bleeding incidence. χ^2^ test *P* values provided. dx code, diagnostic code; Hgb, hemoglobin; transfusion 7 days, blood transfusion received within 7 days of the index procedure.

Postprocedure LOS are similar between USCDT and MT patients in both analyzed cohorts (primary USCDT 3.6 [2.6, 5.6] vs MT 3.6 [2.5, 5.9] *P* = .6); (contemporary USCDT 3.6 [2.6, 5.5] vs MT 3.6 [2.5, 5.9] *P* = .08). There were no statistically significant differences in 30-day readmission rates between MT and USCDT-treated patients. There were no differences in mortality during inpatient stays between the groups in either analysis (Table 2).

**Table 2:** Adverse events derived from electronic health record data.

	Primary 2009-2023			Contemporary 2018-2023		
	*P* value	USCDT	MT	*P* value	USCDT	MT
In-hospital death	.167	41 (2.6%)	25 (3.7%)	.497	33 (2.9%)	23 (3.5%)
Ischemic stroke	.368	26 (1.6%)	15 (2.2%)	.450	20 (1.8%)	15 (2.3%)
Intracerebral hemorrhage	.005	5 (0.3%)	9 (1.3%)	.015	4 (0.4%)	9 (1.4%)
30-day readmission	.777	81 (5.1%)	37 (5.4%)	.730	56 (4.9%)	35 (5.3%)

χ^2^ test *P* values provided.

MT, mechanical thrombectomy; USCDT, ultrasound-assisted catheter-directed thrombolysis.

Unadjusted multiple logistic regression was performed on the primary and contemporary analyses to determine if device type, patient characteristics, or patient medical history was associated with an increased risk of ISTH or BARC 3b major bleeding (Table 3). The regression analysis demonstrates that MT-treated patients were significantly more likely to experience major bleeding. The only other assessed variables predictive of ISTH or BARC 3b bleeding were a history of major bleeding and hemorrhagic stroke within both analyses and an association in white patients solely in the contemporary analysis with ISTH bleeding risk (Table 3).

**Table 3:** ISTH-modeled and BARC 3b-modeled bleeding regression analysis for the primary and contemporary analyses.

	Primary (2009-2023)		Contemporary (2018-2023)	
	ISTH	BARC 3b	ISTH	BARC 3b
	Odds ratio (CI)	Odds ratio (CI)	Odds ratio (CI)	Odds ratio (CI)
MT	1.367 (1.075-1.737)	1.232 (0.961-1.579)	1.608 (1.234-2.095)	1.763 (1.397-2.225)
Hx of COPD	0.830 (0.528-1.305)	0.752 (0.466-1.213)	0.705 (0.401-1.240)	0.778 (0.468-1.294)
Hx of CAD	1.379 (0.944-2.016)	1.220 (0.820-1.815)	1.083 (0.688-1.703)	1.151 (0.764-1.735)
Hx of hypertension	0.990 (0.759-1.290)	0.999 (0.762-1.310)	1.140 (0.840-1.549)	0.940 (0.716-1.234)
Hx of CKD	0.820 (0.546-1.234)	0.805 (0.527-1.229)	0.711 (0.438-1.153)	0.769 (0.496-1.192)
Hx of CDT	1.004 (0.149-6.776)	1.031 (0.150-7.097)	2.419 (0.281-20.824)	2.443 (0.298-20.010)
Hx of MI	0.850 (0.507-1.428)	0.781 (0.450-1.356)	0.763 (0.409-1.425)	0.803 (0.462-1.396)
Hx of ischemic stroke	0.692 (0.384-1.249)	0.817 (0.454-1.472)	0.584 (0.287-1.191)	0.826 (0.445-1.532)
Hx of DVT	1.193 (0.907-1.570)	1.134 (0.855-1.505)	1.330 (0.957-1.849)	1.141 (0.842-1.547)
Hx cancer	0.994 (0.739-1.337)	1.009 (0.744-1.368)	1.026 (0.732-1.438)	1.040 (0.765-1.413)
Prior major bleed	4.842 (3.769-6.220)	5.027 (3.889-6.498)	4.099 (3.051-5.506)	2.972 (2.256-3.914)
Prior hemorrhagic stroke	2.551 (1.143-5.692)	2.225 (0.996-4.971)	4.129 (1.609-10.596)	3.314 (1.314-8.356)
Hx DM	0.740 (0.535-1.024)	0.709 (0.506-0.993)	0.803 (0.550-1.173)	0.829 (0.592-1.163)
Sex = male	0.873 (0.696-1.097)	0.908 (0.719-1.148)	0.824 (0.633-1.073)	0.918 (0.728-1.158)
Race = other	1.178 (0.435-3.194)	1.190 (0.438-3.236)	2.265 (0.698-7.349)	1.280 (0.465-3.520)
Race = White	1.438 (0.966-2.142)	1.373 (0.916-2.059)	2.170 (1.308-3.598)	1.189 (0.816-1.734)
Ethnicity = not Hispanic or Latino	1.164 (0.605-2.239)	1.041 (0.540-2.006)	1.477 (0.706-3.087)	1.041 (0.588-1.844)
Age <60 y	0.951 (0.742-1.218)	0.964 (0.748-1.243)	0.974 (0.729-1.300)	0.971 (0.754-1.250)

BARC, Bleeding Academic Research Consortium; CAD, coronary artery disease; CDT, catheter-directed thrombolysis; CKD, chronic kidney disease; COPD, chronic obstructive pulmonary disease; DM, diabetes mellitus; DVT, deep vein thrombosis; Hx, history; ISTH, International Society for Thrombosis and Hemostasis; MI, myocardial infarction; MT, mechanical thrombectomy; USCDT, ultrasound-assisted catheter-directed thrombolysis.

## Discussion

This large, modern real-world analysis of interventional PE therapy comparing USCDT and MT utilizing a novel data platform revealed increased rates of major bleeding with MT. This work provides further evidence of the increased bleeding risk of MT vs USCDT previously demonstrated only in single-center analyses.^21^

In 2015, a large meta-analysis of 15 trials utilizing full-dose parenteral thrombolytic therapy reported a significant increase in major hemorrhage (OR; 2.91, 95% CI: 1.95-4.36) and fatal or intracranial bleeding (OR: 3.18, 95% CI: 1.25-8.11) associated with the full dose, systemic parenteral tPA vs traditional anticoagulation alone.^22^ Concerns have been raised in contemporary practice ever since this historic work that MT should be utilized in clinical scenarios for patients deemed to be at high risk for bleeding as a means of avoiding the administration of even low doses of thrombolytic therapy. Trial data with low-dose USCDT via EKOS (ULTIMA, KNOCOUT-PE) presented extremely low bleeding risks. Nonetheless, a perceived reduction in bleeding risk with MT over USCDT often persists in practice despite requirements for large-bore venous access and immediate postprocedural anticoagulation with MT.

The actual experience of PE patients among 83 million patients demonstrates findings in contrast to what contemporary practice may have assumed. In this cohort, an increased risk of major bleeding by multiple definitions is associated with the use of MT rather than USCDT. Beyond coding data capturing diagnoses associated with bleeding, this increased bleeding risk with MT is identified here through direct assessment of changes in hemoglobin levels as well as by direct evaluation of the administration of blood transfusion. Coupled with coding information, ISTH and BARC 3b major bleed criteria are modeled and are consistent with these comparisons. Despite the differences in bleeding risks demonstrated between therapies, similar differences in LOS and inpatient mortality were not identified despite prior associations between these safety risks.^23^^,^^24^ It is possible that this work was underpowered to demonstrate associated LOS and inpatient mortality differences associated with the degree of bleeding variation identified. It is possible that the association between these risks is less profound in the setting of PE treated with advanced therapies than with other conditions. It is also possible that the novel mechanism by which bleeding was revealed in this analysis does not carry the same direct associations with LOS and inpatient mortality as that when identified by other methods.

This is not a prospective randomized trial. This work utilizes a large real-world data platform that includes tools to ensure completeness of data, validity, timeliness, and normalization of data. The coding-based finding regarding the increased risk of ICH with MT is thought-provoking for these reasons but not confirmatory. It demonstrates that ICH risk is extremely low with USCDT in the contemporary era of the use of very low doses and short duration of thrombolytic administration with USCDT. However, this finding also raises concerns documented elsewhere in the literature of the possibility of stroke via paradoxical embolization through a patent foramen ovale when an organized thrombus is entrapped on the end of an MT catheter without complete aspiration. This scenario can and has led to cerebral embolization and may explain the finding of intracranial injury and resultant ICH reported here.^25^

Also of interest in this work and not complicated by the limitations of diagnostic coding are the LOS findings. Despite claims of shortening LOS by avoiding infusion-based therapies, there was no demonstrated difference in postprocedure median LOS between MT and USCDT in this analysis. Of note, the median LOS assessed here has similarly been utilized in comparative work in MT previously.^12^^,^^26^ Also of note is the lack of an inpatient stay among an excluded cohort of patients. Procedural death as the etiology for this cohort is unlikely as this would have been captured in the record. Further exploration of this cohort when future data are available will be important and may reveal an emerging practice present in the community that is outside of the standard of care.

There are limitations to all data that is not acquired through prospective randomization including an opportunity for underappreciated confounding factors. However, utilization of tools that allow big data analytics like those incorporated here allows an improved understanding of the actual real-world experience and outcomes of patients. MT and USCDT-treated patients have inherent differences in age and cancer history as reported here. However, the performance of regression analysis including the history of cancer and age did not negate the increased risk of major bleed with MT over USCDT. Other possible confounding factors include a potentially sicker population being treated with MT or differences in periprocedural exposure to systemic dose thrombolytic. We have no evidence of these differences, but they are clinically plausible.

Of note, we did not stratify the severity of PE treated by the European Society of Cardiology criteria.^5^ This will be a point of future study when the system allows for reliable characterization of RV size and function. The reported correlation of lower bleeding with USCDT vs MT is striking regardless of any potential confounding factor because it may be in direct contrast to what many would predict in contemporary clinical practice. Multiple logistic regression of available variables helped confirm these findings within this available data.

The field of advanced therapies for PE has moved as fast as any in modern device history. It is our obligation to understand the safety and efficacy of these therapies as quickly as they are being developed. Though the value of prospective randomized data are unmatched, advanced real-world data analytics like those utilized here provide detailed, laboratory-based analyses of data updated daily and will extend our understanding of these therapies in near real-time. These tools hold the potential to improve our processes of reviewing the safety and efficacy of our therapies to improve our practice and protect our patients.
