# Supplementary material for: Modern Treatment of Pulmonary Embolism (USCDT vs MT): Results From a Real-World, Big Data Analysis (REAL-PE)
Source: J Soc Cardiovasc Angiogr Interv. 2023 Oct 24;3(1):101192. doi: 10.1016/j.jscai.2023.101192 (PMC11308131; doi:10.1016/j.jscai.2023.101192)
Supplement: Supplemental Appendix [file mmc1.pdf]

SUPPLEMENTARY APPENDIX

Summary

**Concepts**	**1. Data Definition**	**1. Adverse Event**	**3. Medical History (table #)**
Deep Vein Thrombosis	yes		T3
Ambulatory Encounter Class	yes		
EKOS	yes		
EKOS w/ UDI Removed	yes		
FlowTriever	yes		
FlowTriever UDIs Removed	yes		
Inpatient Encounter Class	yes		
Pulmonary Embolism	yes		
Ischemic Stroke		yes	T3
MI Type I Only		yes	T3
Hemorrhagic Stroke		yes	
ECMO			S2
Body Mass Index			T1
Cancer			T3
Catheter Directed Thrombectomy			T3
Chronic Obstructive Pulmonary Disease			T3
Coronary Artery Disease			T3
D Dimer			T3
Hypertension			T3
Natriuretic Peptide			T3
Troponin			T3
Direct Oral Anticoagulants			T4
Drug Not Administered			T4
Low Molecular Weight Heparin			T4
Tpa			T4
Unfractionated heparin			T4
Vitamin K Antagonist			T4

Data Definitions

ConceptId	CodeSystem	ConceptCode	ConceptName	Concepts
2649593	Truveta	2649593	EKOS Endovascular System	EKOS w/ UDI Removed
3181980	Truveta	3181980	BOSTON SCIENTIFIC CORPORATION - EkoSonic - Catheter, Continuous Flush	EKOS w/ UDI Removed
3182691	Truveta	3182691	BOSTON SCIENTIFIC CORPORATION - EKOS + - Catheter, continuous flush	EKOS w/ UDI Removed
3186228	Truveta	3186228	BOSTON SCIENTIFIC CORPORATION - EKOS - Catheter, Continuous Flush	EKOS w/ UDI Removed
3164038	Truveta	3164038	EKOS CORPORATION - EkoSonic - Catheter, Continuous Flush	EKOS w/ UDI Removed
2650925	UDI	8.58593E+11	EkoSonic¬Æ - 500-55130 - 6779-005	EKOS w/ UDI Removed
2650921	UDI	8.58593E+11	EkoSonic¬Æ - 500-55106 - 6779-001	EKOS w/ UDI Removed
2650922	UDI	8.58593E+11	EkoSonic¬Æ - 500-55112 - 6779-002	EKOS w/ UDI Removed
2650923	UDI	8.58593E+11	EkoSonic¬Æ - 500-55118 - 6779-003	EKOS w/ UDI Removed
2650924	UDI	8.58593E+11	EkoSonic¬Æ - 500-55124 - 6779-004	EKOS w/ UDI Removed
2983657	UDI	1.91506E+11	EKOS,Ñ¢ + - H74939605106080	EKOS w/ UDI Removed
2983658	UDI	1.91506E+11	EKOS,Ñ¢ + - H74939605106120	EKOS w/ UDI Removed
2983659	UDI	1.91506E+11	EKOS,Ñ¢ + - H74939605106160	EKOS w/ UDI Removed
2983660	UDI	1.91506E+11	EKOS,Ñ¢ + - H74939605106200	EKOS w/ UDI Removed
2983661	UDI	1.91506E+11	EKOS,Ñ¢ + - H74939605135080	EKOS w/ UDI Removed
2983662	UDI	1.91506E+11	EKOS,Ñ¢ + - H74939605135120	EKOS w/ UDI Removed
2983663	UDI	1.91506E+11	EKOS,Ñ¢ + - H74939605135160	EKOS w/ UDI Removed
2983664	UDI	1.91506E+11	EKOS,Ñ¢ + - H74939605135200	EKOS w/ UDI Removed
2650928	UDI	8.58593E+11	EkoSonic¬Æ - 500-56112 - 6795-002	EKOS w/ UDI Removed
2650929	UDI	8.58593E+11	EkoSonic¬Æ - 500-56130 - 6795-005	EKOS w/ UDI Removed
2649593	Truveta	2649593	EKOS Endovascular System	EKOS
3181980	Truveta	3181980	BOSTON SCIENTIFIC CORPORATION - EkoSonic - Catheter, Continuous Flush	EKOS
3182691	Truveta	3182691	BOSTON SCIENTIFIC CORPORATION - EKOS + - Catheter, continuous flush	EKOS
3186228	Truveta	3186228	BOSTON SCIENTIFIC CORPORATION - EKOS - Catheter, Continuous Flush	EKOS
3164038	Truveta	3164038	EKOS CORPORATION - EkoSonic - Catheter, Continuous Flush	EKOS
2650926	UDI	8.58593E+11	EkoSonic¬Æ - 500-55140 - 6779-006	EKOS

2650927	UDI	8.58593E+11	EkoSonic¬Æ - 500-55150 - 6779-007 - Device Size: 50 cm TZ, Length: 106 cm - Catheter, Continuous Flush	EKOS
2650925	UDI	8.58593E+11	EkoSonic¬Æ - 500-55130 - 6779-005	EKOS
2650921	UDI	8.58593E+11	EkoSonic¬Æ - 500-55106 - 6779-001	EKOS
2650922	UDI	8.58593E+11	EkoSonic¬Æ - 500-55112 - 6779-002	EKOS
2650923	UDI	8.58593E+11	EkoSonic¬Æ - 500-55118 - 6779-003	EKOS
2650924	UDI	8.58593E+11	EkoSonic¬Æ - 500-55124 - 6779-004	EKOS
2983657	UDI	1.91506E+11	EKOS,Ñ¢ + - H74939605106080	EKOS
2983658	UDI	1.91506E+11	EKOS,Ñ¢ + - H74939605106120	EKOS
2983659	UDI	1.91506E+11	EKOS,Ñ¢ + - H74939605106160	EKOS
2983660	UDI	1.91506E+11	EKOS,Ñ¢ + - H74939605106200	EKOS
2983661	UDI	1.91506E+11	EKOS,Ñ¢ + - H74939605135080	EKOS
2983662	UDI	1.91506E+11	EKOS,Ñ¢ + - H74939605135120	EKOS
2983663	UDI	1.91506E+11	EKOS,Ñ¢ + - H74939605135160	EKOS
2983664	UDI	1.91506E+11	EKOS,Ñ¢ + - H74939605135200	EKOS
2650928	UDI	8.58593E+11	EkoSonic¬Æ - 500-56112 - 6795-002	EKOS
2650929	UDI	8.58593E+11	EkoSonic¬Æ - 500-56130 - 6795-005	EKOS
2703948	UDI	8.58593E+11	EKOS,Ñ¢ - 700-10410 - EKOS Connector Interface Cable	EKOS
2703949	UDI	8.58593E+11	EKOS,Ñ¢ - 600-40500 - EKOS Control Unit 4.0	EKOS
2703950	UDI	8.58593E+11	EKOS,Ñ¢ CU 4-0 Cart - 700-60401	EKOS
2703951	UDI	8.58593E+11	EkoSonic¬Æ - 600-30000	EKOS
2703952	UDI	8.58593E+11	EkoSonic¬Æ - 700-10302	EKOS
2650931	UDI	8.58593E+11	EkoSonic¬Æ - 500-56150 - 6795-007	EKOS
2650930	UDI	8.58593E+11	EkoSonic¬Æ - 500-56140 - 6795-006 - Device Size: 40 cm TZ, Length: 135 cm - Catheter, Continuous Flush	EKOS
3196964	Truveta	3196964	INARI MEDICAL INC - FlowTriever Catheter, XL	FlowTriever
3198958	Truveta	3198958	INARI MEDICAL INC - FlowTriever Catheter, M	FlowTriever
3181169	Truveta	3181169	INARI MEDICAL INC - FlowTriever - Catheter, Embolectomy	FlowTriever
3194887	Truveta	3194887	INARI MEDICAL INC - FlowTriever	FlowTriever
3197910	Truveta	3197910	INARI MEDICAL INC - FlowTriever Catheter, Large	FlowTriever
3197913	Truveta	3197913	INARI MEDICAL INC - FlowTriever Catheter, S	FlowTriever
2703651	Truveta	2703651	FlowTriever Catheter	FlowTriever

2703624	UDI	8.50291E+11	FlowTriever Catheter, S - 10-101	FlowTriever
2703625	UDI	8.50291E+11	FlowTriever Catheter, XL - 10-104	FlowTriever
2703626	UDI	8.50291E+11	FlowTriever Catheter, Large - 10-103	FlowTriever
2703627	UDI	8.50291E+11	FlowTriever Catheter, M - 10-102	FlowTriever
2703628	UDI	8.50291E+11	FlowTriever - 31-101	FlowTriever
2703629	UDI	8.50291E+11	FlowTriever - 10-105	FlowTriever
2703630	UDI	8.50291E+11	FlowTriever - 30-101	FlowTriever
2703631	UDI	8.50291E+11	FlowTriever - 20-101	FlowTriever
2703632	UDI	8.50291E+11	FlowTriever 2 Catheter, M - 11-102 - Length: 120 cm, Outer Diameter: 4 mm	FlowTriever
3177314	Truveta	3177314	INARI MEDICAL INC - FlowTriever Catheter, M - Peripheral Mechanical Thrombectomy With Aspiration	FlowTriever
3165858	Truveta	3165858	INARI MEDICAL INC - FlowTriever Catheter, XL - Peripheral Mechanical Thrombectomy With Aspiration	FlowTriever
3151294	Truveta	3151294	INARI MEDICAL INC - FlowTriever Catheter, XL - Catheter, Embolectomy	FlowTriever
3173271	Truveta	3173271	INARI MEDICAL INC - FlowTriever Catheter, S - Peripheral Mechanical Thrombectomy With Aspiration	FlowTriever
3184207	Truveta	3184207	INARI MEDICAL INC - FlowTriever Catheter, Large - Catheter, Embolectomy	FlowTriever
3185627	Truveta	3185627	INARI MEDICAL INC - FlowTriever Catheter, Large - Peripheral Mechanical Thrombectomy With Aspiration	FlowTriever
3162854	Truveta	3162854	INARI MEDICAL INC - FlowTriever Catheter, M - Catheter, Embolectomy	FlowTriever
2649593	Truveta	2649593	EKOS Endovascular System	FlowTriever UDIs Removed
3181980	Truveta	3181980	BOSTON SCIENTIFIC CORPORATION - EkoSonic - Catheter, Continuous Flush	FlowTriever UDIs Removed
3182691	Truveta	3182691	BOSTON SCIENTIFIC CORPORATION - EKOS + - Catheter, continuous flush	FlowTriever UDIs Removed
3186228	Truveta	3186228	BOSTON SCIENTIFIC CORPORATION - EKOS - Catheter, Continuous Flush	FlowTriever UDIs Removed
3164038	Truveta	3164038	EKOS CORPORATION - EkoSonic - Catheter, Continuous Flush	FlowTriever UDIs Removed
2650925	UDI	8.58593E+11	EkoSonic¬Æ - 500-55130 - 6779-005	FlowTriever UDIs Removed
2650921	UDI	8.58593E+11	EkoSonic¬Æ - 500-55106 - 6779-001	FlowTriever UDIs Removed

2650922	UDI	8.58593E+11	EkoSonic¬Æ - 500-55112 - 6779-002	FlowTriever UDIs Removed
2650923	UDI	8.58593E+11	EkoSonic¬Æ - 500-55118 - 6779-003	FlowTriever UDIs Removed
2650924	UDI	8.58593E+11	EkoSonic¬Æ - 500-55124 - 6779-004	FlowTriever UDIs Removed
2983657	UDI	1.91506E+11	EKOS,Ñ¢ + - H74939605106080	FlowTriever UDIs Removed
2983658	UDI	1.91506E+11	EKOS,Ñ¢ + - H74939605106120	FlowTriever UDIs Removed
2983659	UDI	1.91506E+11	EKOS,Ñ¢ + - H74939605106160	FlowTriever UDIs Removed
2983660	UDI	1.91506E+11	EKOS,Ñ¢ + - H74939605106200	FlowTriever UDIs Removed
2983661	UDI	1.91506E+11	EKOS,Ñ¢ + - H74939605135080	FlowTriever UDIs Removed
2983662	UDI	1.91506E+11	EKOS,Ñ¢ + - H74939605135120	FlowTriever UDIs Removed
2983663	UDI	1.91506E+11	EKOS,Ñ¢ + - H74939605135160	FlowTriever UDIs Removed
2983664	UDI	1.91506E+11	EKOS,Ñ¢ + - H74939605135200	FlowTriever UDIs Removed
2650928	UDI	8.58593E+11	EkoSonic¬Æ - 500-56112 - 6795-002	FlowTriever UDIs Removed
2650929	UDI	8.58593E+11	EkoSonic¬Æ - 500-56130 - 6795-005	FlowTriever UDIs Removed
529579	ICD10CM	I82.220	Acute embolism and thrombosis of inferior vena cava	Deep Vein Thrombosis
529587	ICD10CM	I82.401	Acute embolism and thrombosis of unspecified deep veins of right lower extremity	Deep Vein Thrombosis
529588	ICD10CM	I82.402	Acute embolism and thrombosis of unspecified deep veins of left lower extremity	Deep Vein Thrombosis
529589	ICD10CM	I82.403	Acute embolism and thrombosis of unspecified deep veins of lower extremity, bilateral	Deep Vein Thrombosis
529590	ICD10CM	I82.409	Acute embolism and thrombosis of unspecified deep veins of unspecified lower extremity	Deep Vein Thrombosis
529592	ICD10CM	I82.411	Acute embolism and thrombosis of right femoral vein	Deep Vein Thrombosis
529593	ICD10CM	I82.412	Acute embolism and thrombosis of left femoral vein	Deep Vein Thrombosis
529594	ICD10CM	I82.413	Acute embolism and thrombosis of femoral vein, bilateral	Deep Vein Thrombosis
529595	ICD10CM	I82.419	Acute embolism and thrombosis of unspecified femoral vein	Deep Vein Thrombosis
529597	ICD10CM	I82.421	Acute embolism and thrombosis of right iliac vein	Deep Vein Thrombosis
529598	ICD10CM	I82.422	Acute embolism and thrombosis of left iliac vein	Deep Vein Thrombosis
529599	ICD10CM	I82.423	Acute embolism and thrombosis of iliac vein, bilateral	Deep Vein Thrombosis

529600	ICD10CM	I82.429	Acute embolism and thrombosis of unspecified iliac vein	Deep Vein Thrombosis
529602	ICD10CM	I82.431	Acute embolism and thrombosis of right popliteal vein	Deep Vein Thrombosis
529603	ICD10CM	I82.432	Acute embolism and thrombosis of left popliteal vein	Deep Vein Thrombosis
529604	ICD10CM	I82.433	Acute embolism and thrombosis of popliteal vein, bilateral	Deep Vein Thrombosis
529605	ICD10CM	I82.439	Acute embolism and thrombosis of unspecified popliteal vein	Deep Vein Thrombosis
529607	ICD10CM	I82.441	Acute embolism and thrombosis of right tibial vein	Deep Vein Thrombosis
529608	ICD10CM	I82.442	Acute embolism and thrombosis of left tibial vein	Deep Vein Thrombosis
529609	ICD10CM	I82.443	Acute embolism and thrombosis of tibial vein, bilateral	Deep Vein Thrombosis
529610	ICD10CM	I82.449	Acute embolism and thrombosis of unspecified tibial vein	Deep Vein Thrombosis
529612	ICD10CM	I82.451	Acute embolism and thrombosis of right peroneal vein	Deep Vein Thrombosis
529613	ICD10CM	I82.452	Acute embolism and thrombosis of left peroneal vein	Deep Vein Thrombosis
529614	ICD10CM	I82.453	Acute embolism and thrombosis of peroneal vein, bilateral	Deep Vein Thrombosis
529615	ICD10CM	I82.459	Acute embolism and thrombosis of unspecified peroneal vein	Deep Vein Thrombosis
529617	ICD10CM	I82.461	Acute embolism and thrombosis of right calf muscular vein	Deep Vein Thrombosis
529618	ICD10CM	I82.462	Acute embolism and thrombosis of left calf muscular vein	Deep Vein Thrombosis
529619	ICD10CM	I82.463	Acute embolism and thrombosis of calf muscular vein, bilateral	Deep Vein Thrombosis
529620	ICD10CM	I82.469	Acute embolism and thrombosis of unspecified calf muscular vein	Deep Vein Thrombosis
529622	ICD10CM	I82.491	Acute embolism and thrombosis of other specified deep vein of right lower extremity	Deep Vein Thrombosis
529623	ICD10CM	I82.492	Acute embolism and thrombosis of other specified deep vein of left lower extremity	Deep Vein Thrombosis
529624	ICD10CM	I82.493	Acute embolism and thrombosis of other specified deep vein of lower extremity, bilateral	Deep Vein Thrombosis
529625	ICD10CM	I82.499	Acute embolism and thrombosis of other specified deep vein of unspecified lower extremity	Deep Vein Thrombosis

529627	ICD10CM	I82.4Y1	Acute embolism and thrombosis of unspecified deep veins of right proximal lower extremity	Deep Vein Thrombosis
529628	ICD10CM	I82.4Y2	Acute embolism and thrombosis of unspecified deep veins of left proximal lower extremity	Deep Vein Thrombosis
529629	ICD10CM	I82.4Y3	Acute embolism and thrombosis of unspecified deep veins of proximal lower extremity, bilateral	Deep Vein Thrombosis
529630	ICD10CM	I82.4Y9	Acute embolism and thrombosis of unspecified deep veins of unspecified proximal lower extremity	Deep Vein Thrombosis
529632	ICD10CM	I82.4Z1	Acute embolism and thrombosis of unspecified deep veins of right distal lower extremity	Deep Vein Thrombosis
529633	ICD10CM	I82.4Z2	Acute embolism and thrombosis of unspecified deep veins of left distal lower extremity	Deep Vein Thrombosis
529634	ICD10CM	I82.4Z3	Acute embolism and thrombosis of unspecified deep veins of distal lower extremity, bilateral	Deep Vein Thrombosis
529635	ICD10CM	I82.4Z9	Acute embolism and thrombosis of unspecified deep veins of unspecified distal lower extremity	Deep Vein Thrombosis
529699	ICD10CM	I82.621	Acute embolism and thrombosis of deep veins of right upper extremity	Deep Vein Thrombosis
529700	ICD10CM	I82.622	Acute embolism and thrombosis of deep veins of left upper extremity	Deep Vein Thrombosis
529701	ICD10CM	I82.623	Acute embolism and thrombosis of deep veins of upper extremity, bilateral	Deep Vein Thrombosis
529702	ICD10CM	I82.629	Acute embolism and thrombosis of deep veins of unspecified upper extremity	Deep Vein Thrombosis
1065216	Truveta	1065216	ambulatory	Ambulatory Encounter Class
1065220	Truveta	1065220	inpatient encounter	Inpatient Encounter Class
1065215	Truveta	1065215	inpatient acute	Inpatient Encounter Class
1065223	Truveta	1065223	inpatient non-acute	Inpatient Encounter Class
528423	ICD10CM	I26.01	Septic pulmonary embolism with acute cor pulmonale	Pulmonary Embolism
528424	ICD10CM	I26.02	Saddle embolus of pulmonary artery with acute cor pulmonale	Pulmonary Embolism
528425	ICD10CM	I26.09	Other pulmonary embolism with acute cor pulmonale	Pulmonary Embolism
528428	ICD10CM	I26.92	Saddle embolus of pulmonary artery without acute cor pulmonale	Pulmonary Embolism
528429	ICD10CM	I26.93	Single subsegmental pulmonary embolism without acute cor pulmonale	Pulmonary Embolism

528430	ICD10CM	I26.94	Multiple subsegmental pulmonary emboli without acute cor pulmonale	Pulmonary Embolism
528431	ICD10CM	I26.99	Other pulmonary embolism without acute cor pulmonale	Pulmonary Embolism

Adverse Events

ConceptId	CodeSystem	ConceptCode	ConceptName	Concepts
457866	SNOMED CT	6.7435E+14	Apraxia due to and following hemorrhagic cerebrovascular accident	Hemorrhagic Stroke
501319	SNOMED CT	230706003	Hemorrhagic stroke	Hemorrhagic Stroke
349704	SNOMED CT	417506008	Hemorrhagic stroke monitoring	Hemorrhagic Stroke
177814	SNOMED CT	195163003	Intracerebral hemorrhage (& [cerebrovascular accident due to])	Hemorrhagic Stroke
212907	SNOMED CT	230707007	Anterior cerebral circulation hemorrhagic infarction	Hemorrhagic Stroke
212908	SNOMED CT	230708002	Posterior cerebral circulation hemorrhagic infarction	Hemorrhagic Stroke
2646758	SNOMED CT	1163482004	Hemorrhagic cerebral infarction caused by Aspergillus	Hemorrhagic Stroke
319787	SNOMED CT	384993003	Periventricular hemorrhagic venous infarct	Hemorrhagic Stroke
528678	ICD10CM	I63.10	Cerebral infarction due to embolism of unspecified precerebral artery	Ischemic Stroke
528680	ICD10CM	I63.111	Cerebral infarction due to embolism of right vertebral artery	Ischemic Stroke
528681	ICD10CM	I63.112	Cerebral infarction due to embolism of left vertebral artery	Ischemic Stroke
528682	ICD10CM	I63.113	Cerebral infarction due to embolism of bilateral vertebral arteries	Ischemic Stroke
528683	ICD10CM	I63.119	Cerebral infarction due to embolism of unspecified vertebral artery	Ischemic Stroke
528684	ICD10CM	I63.12	Cerebral infarction due to embolism of basilar artery	Ischemic Stroke
528686	ICD10CM	I63.131	Cerebral infarction due to embolism of right carotid artery	Ischemic Stroke
528687	ICD10CM	I63.132	Cerebral infarction due to embolism of left carotid artery	Ischemic Stroke

528688	ICD10CM	I63.133	Cerebral infarction due to embolism of bilateral carotid arteries	Ischemic Stroke
528689	ICD10CM	I63.139	Cerebral infarction due to embolism of unspecified carotid artery	Ischemic Stroke
528690	ICD10CM	I63.19	Cerebral infarction due to embolism of other precerebral artery	Ischemic Stroke
528729	ICD10CM	I63.40	Cerebral infarction due to embolism of unspecified cerebral artery	Ischemic Stroke
528731	ICD10CM	I63.411	Cerebral infarction due to embolism of right middle cerebral artery	Ischemic Stroke
528732	ICD10CM	I63.412	Cerebral infarction due to embolism of left middle cerebral artery	Ischemic Stroke
528733	ICD10CM	I63.413	Cerebral infarction due to embolism of bilateral middle cerebral arteries	Ischemic Stroke
528734	ICD10CM	I63.419	Cerebral infarction due to embolism of unspecified middle cerebral artery	Ischemic Stroke
528736	ICD10CM	I63.421	Cerebral infarction due to embolism of right anterior cerebral artery	Ischemic Stroke
528737	ICD10CM	I63.422	Cerebral infarction due to embolism of left anterior cerebral artery	Ischemic Stroke
528738	ICD10CM	I63.423	Cerebral infarction due to embolism of bilateral anterior cerebral arteries	Ischemic Stroke
528739	ICD10CM	I63.429	Cerebral infarction due to embolism of unspecified anterior cerebral artery	Ischemic Stroke
528741	ICD10CM	I63.431	Cerebral infarction due to embolism of right posterior cerebral artery	Ischemic Stroke
528742	ICD10CM	I63.432	Cerebral infarction due to embolism of left posterior cerebral artery	Ischemic Stroke
528743	ICD10CM	I63.433	Cerebral infarction due to embolism of bilateral posterior cerebral arteries	Ischemic Stroke
528744	ICD10CM	I63.439	Cerebral infarction due to embolism of unspecified posterior cerebral artery	Ischemic Stroke
528746	ICD10CM	I63.441	Cerebral infarction due to embolism of right cerebellar artery	Ischemic Stroke

528747	ICD10CM	I63.442	Cerebral infarction due to embolism of left cerebellar artery	Ischemic Stroke
528748	ICD10CM	I63.443	Cerebral infarction due to embolism of bilateral cerebellar arteries	Ischemic Stroke
528749	ICD10CM	I63.449	Cerebral infarction due to embolism of unspecified cerebellar artery	Ischemic Stroke
528750	ICD10CM	I63.49	Cerebral infarction due to embolism of other cerebral artery	Ischemic Stroke
1175289	ICD9CM	433.01	Occlusion and stenosis of basilar artery with cerebral infarction	Ischemic Stroke
1175291	ICD9CM	433.1	Occlusion and stenosis of carotid artery without mention of cerebral infarction	Ischemic Stroke
1175292	ICD9CM	433.11	Occlusion and stenosis of carotid artery with cerebral infarction	Ischemic Stroke
1175295	ICD9CM	433.21	Occlusion and stenosis of vertebral artery with cerebral infarction	Ischemic Stroke
1175298	ICD9CM	433.31	Occlusion and stenosis of multiple and bilateral precerebral arteries with cerebral infarction	Ischemic Stroke
1175301	ICD9CM	433.81	Occlusion and stenosis of other specified precerebral artery with cerebral infarction	Ischemic Stroke
1175304	ICD9CM	433.91	Occlusion and stenosis of unspecified precerebral artery with cerebral infarction	Ischemic Stroke
1175307	ICD9CM	434	Cerebral thrombosis without mention of cerebral infarction	Ischemic Stroke
1175308	ICD9CM	434.01	Cerebral thrombosis with cerebral infarction	Ischemic Stroke
1175311	ICD9CM	434.11	Cerebral embolism with cerebral infarction	Ischemic Stroke
1175314	ICD9CM	434.91	Cerebral artery occlusion, unspecified with cerebral infarction	Ischemic Stroke
1175322	ICD9CM	436	Acute, but ill-defined, cerebrovascular disease	Ischemic Stroke
177837	SNOMED CT	195186005	Cerebral infarction due to embolism of precerebral arteries	Ischemic Stroke
177841	SNOMED CT	195190007	Cerebral infarction due to embolism of cerebral arteries	Ischemic Stroke

Medical History

ConceptId	CodeSystem	ConceptCode	ConceptName	Concepts
809891	LOINC	39156-5	Body mass index (BMI) [Ratio]	Body Mass Index
528318	ICD10CM	I20.0	Unstable angina	Coronary Artery Disease
528319	ICD10CM	I20.1	Angina pectoris with documented spasm	Coronary Artery Disease
528320	ICD10CM	I20.8	Other forms of angina pectoris	Coronary Artery Disease
528321	ICD10CM	I20.9	Angina pectoris, unspecified	Coronary Artery Disease
528356	ICD10CM	I24.0	Acute coronary thrombosis not resulting in myocardial infarction	Coronary Artery Disease
528358	ICD10CM	I24.8	Other forms of acute ischemic heart disease	Coronary Artery Disease
528359	ICD10CM	I24.9	Acute ischemic heart disease, unspecified	Coronary Artery Disease
528362	ICD10CM	I25.10	Atherosclerotic heart disease of native coronary artery without angina pectoris	Coronary Artery Disease
528364	ICD10CM	I25.110	Atherosclerotic heart disease of native coronary artery with unstable angina pectoris	Coronary Artery Disease
528365	ICD10CM	I25.111	Atherosclerotic heart disease of native coronary artery with angina pectoris with documented spasm	Coronary Artery Disease
528366	ICD10CM	I25.118	Atherosclerotic heart disease of native coronary artery with other forms of angina pectoris	Coronary Artery Disease
528367	ICD10CM	I25.119	Atherosclerotic heart disease of native coronary artery with unspecified angina pectoris	Coronary Artery Disease
528373	ICD10CM	I25.5	Ischemic cardiomyopathy	Coronary Artery Disease
528374	ICD10CM	I25.6	Silent myocardial ischemia	Coronary Artery Disease
528377	ICD10CM	I25.700	Atherosclerosis of coronary artery bypass graft(s), unspecified, with unstable angina pectoris	Coronary Artery Disease
528378	ICD10CM	I25.701	Atherosclerosis of coronary artery bypass graft(s), unspecified, with angina pectoris with documented spasm	Coronary Artery Disease
528379	ICD10CM	I25.708	Atherosclerosis of coronary artery bypass graft(s), unspecified, with other forms of angina pectoris	Coronary Artery Disease
528380	ICD10CM	I25.709	Atherosclerosis of coronary artery bypass graft(s), unspecified, with unspecified angina pectoris	Coronary Artery Disease
528382	ICD10CM	I25.710	Atherosclerosis of autologous vein coronary artery bypass	Coronary Artery Disease

			graft(s) with unstable angina pectoris	
528383	ICD10CM	I25.711	Atherosclerosis of autologous vein coronary artery bypass graft(s) with angina pectoris with documented spasm	Coronary Artery Disease
528384	ICD10CM	I25.718	Atherosclerosis of autologous vein coronary artery bypass graft(s) with other forms of angina pectoris	Coronary Artery Disease
528385	ICD10CM	I25.719	Atherosclerosis of autologous vein coronary artery bypass graft(s) with unspecified angina pectoris	Coronary Artery Disease
528387	ICD10CM	I25.720	Atherosclerosis of autologous artery coronary artery bypass graft(s) with unstable angina pectoris	Coronary Artery Disease
528388	ICD10CM	I25.721	Atherosclerosis of autologous artery coronary artery bypass graft(s) with angina pectoris with documented spasm	Coronary Artery Disease
528389	ICD10CM	I25.728	Atherosclerosis of autologous artery coronary artery bypass graft(s) with other forms of angina pectoris	Coronary Artery Disease
528390	ICD10CM	I25.729	Atherosclerosis of autologous artery coronary artery bypass graft(s) with unspecified angina pectoris	Coronary Artery Disease
528392	ICD10CM	I25.730	Atherosclerosis of nonautologous biological coronary artery bypass graft(s) with unstable angina pectoris	Coronary Artery Disease
528393	ICD10CM	I25.731	Atherosclerosis of nonautologous biological coronary artery bypass graft(s) with angina pectoris with documented spasm	Coronary Artery Disease
528394	ICD10CM	I25.738	Atherosclerosis of nonautologous biological coronary artery bypass graft(s) with other forms of angina pectoris	Coronary Artery Disease
528395	ICD10CM	I25.739	Atherosclerosis of nonautologous biological coronary artery bypass graft(s) with unspecified angina pectoris	Coronary Artery Disease
528397	ICD10CM	I25.750	Atherosclerosis of native coronary artery of transplanted heart with unstable angina	Coronary Artery Disease

528398	ICD10CM	I25.751	Atherosclerosis of native coronary artery of transplanted heart with angina pectoris with documented spasm	Coronary Artery Disease
528399	ICD10CM	I25.758	Atherosclerosis of native coronary artery of transplanted heart with other forms of angina pectoris	Coronary Artery Disease
528400	ICD10CM	I25.759	Atherosclerosis of native coronary artery of transplanted heart with unspecified angina pectoris	Coronary Artery Disease
528402	ICD10CM	I25.760	Atherosclerosis of bypass graft of coronary artery of transplanted heart with unstable angina	Coronary Artery Disease
528403	ICD10CM	I25.761	Atherosclerosis of bypass graft of coronary artery of transplanted heart with angina pectoris with documented spasm	Coronary Artery Disease
528404	ICD10CM	I25.768	Atherosclerosis of bypass graft of coronary artery of transplanted heart with other forms of angina pectoris	Coronary Artery Disease
528405	ICD10CM	I25.769	Atherosclerosis of bypass graft of coronary artery of transplanted heart with unspecified angina pectoris	Coronary Artery Disease
528407	ICD10CM	I25.790	Atherosclerosis of other coronary artery bypass graft(s) with unstable angina pectoris	Coronary Artery Disease
528408	ICD10CM	I25.791	Atherosclerosis of other coronary artery bypass graft(s) with angina pectoris with documented spasm	Coronary Artery Disease
528409	ICD10CM	I25.798	Atherosclerosis of other coronary artery bypass graft(s) with other forms of angina pectoris	Coronary Artery Disease
528410	ICD10CM	I25.799	Atherosclerosis of other coronary artery bypass graft(s) with unspecified angina pectoris	Coronary Artery Disease
528413	ICD10CM	I25.810	Atherosclerosis of coronary artery bypass graft(s) without angina pectoris	Coronary Artery Disease
528414	ICD10CM	I25.811	Atherosclerosis of native coronary artery of transplanted heart without angina pectoris	Coronary Artery Disease
528415	ICD10CM	I25.812	Atherosclerosis of bypass graft of coronary artery of	Coronary Artery Disease

			transplanted heart without angina pectoris	
528416	ICD10CM	I25.82	Chronic total occlusion of coronary artery	Coronary Artery Disease
528417	ICD10CM	I25.83	Coronary atherosclerosis due to lipid rich plaque	Coronary Artery Disease
528419	ICD10CM	I25.89	Other forms of chronic ischemic heart disease	Coronary Artery Disease
528420	ICD10CM	I25.9	Chronic ischemic heart disease, unspecified	Coronary Artery Disease
613307	ICD10CM	Z95.1	Presence of aortocoronary bypass graft	Coronary Artery Disease
613311	ICD10CM	Z95.5	Presence of coronary angioplasty implant and graft	Coronary Artery Disease
613402	ICD10CM	Z98.61	Coronary angioplasty status	Coronary Artery Disease
4213	SNOMED CT	4557003	Preinfarction syndrome	Coronary Artery Disease
9688	SNOMED CT	10365005	Right main coronary artery thrombosis	Coronary Artery Disease
69913	SNOMED CT	74218008	Coronary artery arising from main pulmonary artery	Coronary Artery Disease
71020	SNOMED CT	75398000	Anomalous origin of coronary artery	Coronary Artery Disease
80308	SNOMED CT	85284003	Angina, class III	Coronary Artery Disease
82250	SNOMED CT	87343002	Prinzmetal angina	Coronary Artery Disease
84128	SNOMED CT	89323001	Angina, class IV	Coronary Artery Disease
87180	SNOMED CT	92517006	Calcific coronary arteriosclerosis	Coronary Artery Disease
110805	SNOMED CT	123641001	Left coronary artery occlusion	Coronary Artery Disease
110806	SNOMED CT	123642008	Right coronary artery occlusion	Coronary Artery Disease
292954	SNOMED CT	314116003	Post infarct angina	Coronary Artery Disease
293810	SNOMED CT	315025001	Refractory angina	Coronary Artery Disease
294094	SNOMED CT	315348000	Asymptomatic coronary heart disease	Coronary Artery Disease
314605	SNOMED CT	371803003	Multi vessel coronary artery disease	Coronary Artery Disease
314606	SNOMED CT	371804009	Left main coronary artery disease	Coronary Artery Disease
314607	SNOMED CT	371805005	Significant coronary bypass graft disease	Coronary Artery Disease
314608	SNOMED CT	371806006	Progressive angina	Coronary Artery Disease
314609	SNOMED CT	371807002	Atypical angina	Coronary Artery Disease

314610	SNOMED CT	371808007	Recurrent angina after percutaneous transluminal coronary angioplasty	Coronary Artery Disease
314611	SNOMED CT	371809004	Recurrent angina following placement of coronary artery stent	Coronary Artery Disease
314612	SNOMED CT	371810009	Recurrent angina after coronary artery bypass graft	Coronary Artery Disease
314613	SNOMED CT	371811008	Recurrent angina post rotational atherectomy	Coronary Artery Disease
314614	SNOMED CT	371812001	Recurrent angina after directional coronary atherectomy	Coronary Artery Disease
331852	SNOMED CT	398274000	Coronary artery thrombosis	Coronary Artery Disease
341461	SNOMED CT	408546009	Coronary artery bypass graft occlusion	Coronary Artery Disease
352046	SNOMED CT	420006002	Obliterative coronary artery disease	Coronary Artery Disease
353263	SNOMED CT	421327009	Coronary artery stent thrombosis	Coronary Artery Disease
359601	SNOMED CT	427919004	Coronary arteriosclerosis due to radiation	Coronary Artery Disease
360850	SNOMED CT	429245005	Recurrent coronary arteriosclerosis after percutaneous transluminal coronary angioplasty	Coronary Artery Disease
361145	SNOMED CT	429559004	Typical angina	Coronary Artery Disease
371565	SNOMED CT	443502000	Coronary atherosclerosis	Coronary Artery Disease
215985	SNOMED CT	233817007	Triple vessel disease of the heart	Coronary Artery Disease
215987	SNOMED CT	233819005	Stable angina	Coronary Artery Disease
215989	SNOMED CT	233821000	New onset angina	Coronary Artery Disease
216137	SNOMED CT	233970002	Coronary artery stenosis	Coronary Artery Disease
280819	SNOMED CT	300995000	Exercise-induced angina	Coronary Artery Disease
177476	SNOMED CT	194823009	Acute coronary insufficiency	Coronary Artery Disease
177481	SNOMED CT	194828000	Angina pectoris	Coronary Artery Disease
177495	SNOMED CT	194842008	Single coronary vessel disease	Coronary Artery Disease
177496	SNOMED CT	194843003	Double coronary vessel disease	Coronary Artery Disease
17901	SNOMED CT	19057007	Status anginosus	Coronary Artery Disease

20182	SNOMED CT	21470009	Syncope anginosa	Coronary Artery Disease
26588	SNOMED CT	28248000	Left anterior descending coronary artery thrombosis	Coronary Artery Disease
28124	SNOMED CT	29899005	Coronary artery embolism	Coronary Artery Disease
33836	SNOMED CT	35928006	Nocturnal angina	Coronary Artery Disease
38950	SNOMED CT	41334000	Angina, class II	Coronary Artery Disease
47640	SNOMED CT	50570003	Aneurysm of coronary vessels	Coronary Artery Disease
50629	SNOMED CT	53741008	Coronary arteriosclerosis	Coronary Artery Disease
55578	SNOMED CT	59021001	Angina decubitus	Coronary Artery Disease
57904	SNOMED CT	61490001	Angina, class I	Coronary Artery Disease
58584	SNOMED CT	62207008	Syphilitic ostial coronary disease	Coronary Artery Disease
60014	SNOMED CT	63739005	Coronary occlusion	Coronary Artery Disease
63744	SNOMED CT	67682002	Coronary artery atheroma	Coronary Artery Disease
361253	SNOMED CT	429673002	Arteriosclerosis of coronary artery bypass graft	Coronary Artery Disease
370322	SNOMED CT	442224005	Arteriosclerosis of autologous vein coronary artery bypass graft	Coronary Artery Disease
370338	SNOMED CT	442240008	Arteriosclerosis of nonautologous coronary artery bypass graft	Coronary Artery Disease
370511	SNOMED CT	442421004	Arteriosclerosis of arterial coronary artery bypass graft	Coronary Artery Disease
372853	SNOMED CT	444855007	Arteriosclerosis of coronary artery bypass graft of transplanted heart	Coronary Artery Disease
372854	SNOMED CT	444856008	Arteriosclerosis of internal mammary artery coronary artery bypass graft	Coronary Artery Disease
377787	SNOMED CT	450300002	Anomalous origin of right coronary artery from pulmonary artery	Coronary Artery Disease
377788	SNOMED CT	450301003	Anomalous origin of left coronary artery from pulmonary artery	Coronary Artery Disease
377789	SNOMED CT	450302005	Anomalous origin of left anterior descending coronary artery from pulmonary artery	Coronary Artery Disease
378654	SNOMED CT	458039003	Anomalous origin of left circumflex coronary artery from pulmonary artery	Coronary Artery Disease

378797	SNOMED CT	459066006	Anomalous origin of accessory coronary artery from pulmonary artery	Coronary Artery Disease
378929	SNOMED CT	460437005	Anomalous origin of dual left anterior descending coronary arteries from right coronary artery and left coronary artery	Coronary Artery Disease
378930	SNOMED CT	460438000	Anomalous origin of large conus artery from right coronary artery	Coronary Artery Disease
378934	SNOMED CT	460471001	Anomalous origin of conus artery from separate aortic sinus orifice	Coronary Artery Disease
378941	SNOMED CT	460510005	Anomalous origin of coronary arteries from anterior aortic sinus	Coronary Artery Disease
378942	SNOMED CT	460517008	Anomalous origin of coronary arteries from both aortic sinuses of bicuspid valve	Coronary Artery Disease
378943	SNOMED CT	460524009	Anomalous origin of right coronary artery from left anterior descending coronary artery	Coronary Artery Disease
378944	SNOMED CT	460531008	Anomalous origin of right coronary artery from left circumflex coronary artery	Coronary Artery Disease
378945	SNOMED CT	460538002	Anomalous origin of left coronary artery from right coronary artery	Coronary Artery Disease
378947	SNOMED CT	460581004	Anomalous origin of accessory coronary artery from aortic sinus	Coronary Artery Disease
378948	SNOMED CT	460582006	Anomalous origin of left coronary artery and right coronary artery from pulmonary artery	Coronary Artery Disease
378949	SNOMED CT	460583001	Anomalous origin of sinus node coronary artery from separate aortic sinus orifice	Coronary Artery Disease
378952	SNOMED CT	460586009	Anomalous origin of right coronary artery from left coronary artery	Coronary Artery Disease
378953	SNOMED CT	460587000	Anomalous origin of single coronary artery from left coronary artery aortic sinus	Coronary Artery Disease
378954	SNOMED CT	460588005	Anomalous origin of left circumflex coronary artery from right coronary aortic sinus	Coronary Artery Disease
378993	SNOMED CT	460923005	Anomalous origin of right coronary artery from left coronary artery aortic sinus	Coronary Artery Disease

378994	SNOMED CT	460930004	Anomalous origin of left coronary artery from right coronary aortic sinus	Coronary Artery Disease
378995	SNOMED CT	460937001	Anomalous origin of single coronary artery from right coronary artery aortic sinus	Coronary Artery Disease
378996	SNOMED CT	460944005	Anomalous origin of right coronary artery from left coronary artery aortic sinus and anomalous origin of left coronary artery from right coronary artery aortic sinus	Coronary Artery Disease
379033	SNOMED CT	461105005	Anomalous origin of coronary artery from aorta	Coronary Artery Disease
379110	SNOMED CT	461435009	Anomalous origin of left anterior descending coronary artery from right coronary artery aortic sinus	Coronary Artery Disease
379111	SNOMED CT	461436005	Anomalous origin of dual left anterior descending coronary arteries	Coronary Artery Disease
385990	SNOMED CT	471285003	Anomalous origin of coronary artery from aortic sinus to left of nonfacing aortic sinus	Coronary Artery Disease
385991	SNOMED CT	471286002	Anomalous origin of coronary artery from aortic sinus to right of nonfacing aortic sinus	Coronary Artery Disease
385992	SNOMED CT	471287006	Anomalous origin of left coronary artery and right coronary artery with dual orifices from aortic sinus to left of nonfacing aortic sinus	Coronary Artery Disease
385993	SNOMED CT	471288001	Anomalous origin of left coronary artery and right coronary artery with dual orifices from aortic sinus to right of nonfacing aortic sinus	Coronary Artery Disease
385994	SNOMED CT	471289009	Anomalous origin of circumflex artery from aortic sinus to right of nonfacing aortic sinus and anomalous origin of left anterior descending coronary artery and right coronary artery from aortic sinus to left of nonfacing aortic sinus	Coronary Artery Disease
385995	SNOMED CT	471290000	Anomalous origin of left anterior descending artery from aortic sinus to right of nonfacing aortic sinus and anomalous origin of circumflex artery and right coronary artery from aortic sinus to left of nonfacing aortic sinus	Coronary Artery Disease

385996	SNOMED CT	471291001	Anomalous origin of right coronary artery from aortic sinus to right of nonfacing aortic sinus and anomalous origin of left coronary artery from aortic sinus to left of nonfacing aortic sinus	Coronary Artery Disease
385997	SNOMED CT	471292008	Anomalous origin of right coronary artery and circumflex artery from aortic sinus to right of nonfacing aortic sinus and anomalous origin of left anterior descending artery from aortic sinus to left on nonfacing aortic sinus	Coronary Artery Disease
385998	SNOMED CT	471293003	Anomalous origin of left anterior descending artery and right coronary artery from aortic sinus to right of nonfacing aortic sinus and anomalous origin of circumflex artery from aortic sinus to left of nonfacing aortic sinus	Coronary Artery Disease
386002	SNOMED CT	471297002	Anomalous origin of single coronary artery from nonfacing aortic sinus	Coronary Artery Disease
386003	SNOMED CT	471298007	Anomalous origin of single coronary artery from aortic sinus to left of nonfacing aortic sinus	Coronary Artery Disease
386004	SNOMED CT	471299004	Anomalous origin of single coronary artery from aortic sinus to right of nonfacing aortic sinus	Coronary Artery Disease
386706	SNOMED CT	473362006	Congenital stenosis of distal coronary artery	Coronary Artery Disease
386786	SNOMED CT	473443007	Anomalous origin of right coronary artery	Coronary Artery Disease
386787	SNOMED CT	473444001	Anomalous origin of left coronary artery	Coronary Artery Disease
216012	SNOMED CT	233844002	Accelerated coronary artery disease in transplanted heart	Coronary Artery Disease
232696	SNOMED CT	251024009	Coronary graft stenosis	Coronary Artery Disease
235269	SNOMED CT	253700001	Variant coronary origin from aortic sinus	Coronary Artery Disease
235272	SNOMED CT	253703004	Anomalous origin of coronary artery from non-facing sinus	Coronary Artery Disease
235273	SNOMED CT	253704005	Anomalous origin of left anterior descending from right coronary artery	Coronary Artery Disease
235275	SNOMED CT	253706007	Anomalous origin of coronary artery from pulmonary arterial tree	Coronary Artery Disease

235276	SNOMED CT	253707003	Anomalous origin of coronary artery from right pulmonary artery	Coronary Artery Disease
235277	SNOMED CT	253708008	Anomalous origin of coronary artery from left pulmonary artery	Coronary Artery Disease
392798	SNOMED CT	703356002	Coronary artery occlusion due to neoplastic disease	Coronary Artery Disease
406510	SNOMED CT	719678003	Non-obstructive atherosclerosis of coronary artery	Coronary Artery Disease
409982	SNOMED CT	723862008	Atherosclerosis of non-autologous coronary artery bypass graft	Coronary Artery Disease
410358	SNOMED CT	724431008	Atherosclerosis of autologous coronary artery bypass graft	Coronary Artery Disease
420111	SNOMED CT	735565009	Perforation of coronary artery co-occurrent and due to aneurysm of coronary artery	Coronary Artery Disease
420112	SNOMED CT	735566005	Rupture of coronary artery co-occurrent and due to aneurysm of coronary artery	Coronary Artery Disease
437434	SNOMED CT	780842009	Aortopulmonary coronary arterial course	Coronary Artery Disease
438745	SNOMED CT	782699008	Congenital stenosis of coronary ostium	Coronary Artery Disease
445050	SNOMED CT	840310005	Occlusion of proximal left anterior descending coronary artery	Coronary Artery Disease
445052	SNOMED CT	840313007	Occlusion of mid left anterior descending coronary artery	Coronary Artery Disease
445053	SNOMED CT	840315000	Occlusion of distal left anterior descending coronary artery	Coronary Artery Disease
445279	SNOMED CT	840608004	Occlusion of anterior descending branch of left coronary artery	Coronary Artery Disease
445343	SNOMED CT	840679006	Occlusion of septal branch of anterior descending branch of left coronary artery	Coronary Artery Disease
445463	SNOMED CT	846667001	Occlusion of diagonal branch of anterior descending branch of left coronary artery	Coronary Artery Disease
445475	SNOMED CT	846684007	Occlusion of intermediate artery	Coronary Artery Disease
446319	SNOMED CT	868215007	Occlusion of proximal portion of right coronary artery	Coronary Artery Disease
446320	SNOMED CT	868216008	Occlusion of distal portion of right coronary artery	Coronary Artery Disease
446322	SNOMED CT	868219001	Occlusion of mid portion of right coronary artery	Coronary Artery Disease
446324	SNOMED CT	868221006	Occlusion of marginal branch of right coronary artery	Coronary Artery Disease

446325	SNOMED CT	868222004	Occlusion of posterior descending branch of right coronary artery	Coronary Artery Disease
446326	SNOMED CT	868223009	Occlusion of posterior lateral branch of right coronary artery	Coronary Artery Disease
450851	SNOMED CT	7.874E+13	Acute coronary artery occlusion not resulting in myocardial infarction	Coronary Artery Disease
451481	SNOMED CT	1.171E+14	Chronic total occlusion of coronary artery	Coronary Artery Disease
451989	SNOMED CT	1.39E+14	Coronary arteriosclerosis following coronary artery bypass graft	Coronary Artery Disease
452344	SNOMED CT	2.851E+14	Arteriosclerosis of autologous arterial coronary artery bypass graft	Coronary Artery Disease
452345	SNOMED CT	2.852E+14	Arteriosclerosis of autologous coronary artery bypass graft	Coronary Artery Disease
456789	SNOMED CT	4.51E+14	Atherosclerosis of coronary artery without angina pectoris	Coronary Artery Disease
456821	SNOMED CT	4.514E+14	Lipid-rich atherosclerosis of coronary artery	Coronary Artery Disease
457254	SNOMED CT	4.559E+14	Occlusion of circumflex coronary artery	Coronary Artery Disease
461180	SNOMED CT	1.102E+16	Coronary arteriosclerosis after percutaneous coronary angioplasty	Coronary Artery Disease
464006	SNOMED CT	1.596E+16	Unstable angina co-occurrent and due to coronary arteriosclerosis	Coronary Artery Disease
464007	SNOMED CT	1.596E+16	Angina co-occurrent and due to coronary arteriosclerosis	Coronary Artery Disease
464008	SNOMED CT	1.596E+16	Angina co-occurrent and due to arteriosclerosis of coronary artery bypass graft	Coronary Artery Disease
464009	SNOMED CT	1.596E+16	Unstable angina due to arteriosclerosis of autologous vein coronary artery bypass graft	Coronary Artery Disease
464010	SNOMED CT	1.596E+16	Arteriosclerosis of autologous vein coronary artery bypass graft with angina	Coronary Artery Disease
464011	SNOMED CT	1.596E+16	Unstable angina co-occurrent and due to arteriosclerosis of coronary artery bypass graft	Coronary Artery Disease
464012	SNOMED CT	1.596E+16	Arteriosclerosis of autologous arterial coronary artery bypass graft with angina	Coronary Artery Disease
464490	SNOMED CT	1.603E+16	Aneurysm of coronary artery due to and following acute febrile mucocutaneous lymph node syndrome	Coronary Artery Disease

465438	SNOMED CT	1.675E+16	Stable angina due to coronary arteriosclerosis	Coronary Artery Disease
465499	SNOMED CT	1.689E+16	Coronary artery disease due to type 2 diabetes mellitus	Coronary Artery Disease
465542	SNOMED CT	1.983E+16	Thrombosis of left circumflex artery	Coronary Artery Disease
516340	SNOMED CT	7.91E+11	Angina due to type 2 diabetes mellitus	Coronary Artery Disease
177474	SNOMED CT	194821006	Coronary thrombosis not resulting in myocardial infarction	Coronary Artery Disease
186915	SNOMED CT	204378009	Congenital coronary aneurysm	Coronary Artery Disease
186916	SNOMED CT	204379001	Congenital stenosis of coronary artery	Coronary Artery Disease
1201864	SNOMED CT	1.596E+16	Unstable angina due to arteriosclerosis of coronary artery bypass graft of transplanted heart	Coronary Artery Disease
1201865	SNOMED CT	1.596E+16	Unstable angina due to arteriosclerosis of autologous arterial coronary artery bypass graft	Coronary Artery Disease
1202040	SNOMED CT	1.689E+16	Coronary artery disease due to type 1 diabetes mellitus	Coronary Artery Disease
1202970	SNOMED CT	876856005	Stenosis of right coronary artery	Coronary Artery Disease
1202971	SNOMED CT	876857001	Stenosis of left coronary artery main stem	Coronary Artery Disease
1202972	SNOMED CT	876858006	Stenosis of circumflex branch of left coronary artery	Coronary Artery Disease
1202973	SNOMED CT	876859003	Stenosis of anterior descending branch of left coronary artery	Coronary Artery Disease
1204022	SNOMED CT	896690007	Occlusion of circumflex branch of left coronary artery	Coronary Artery Disease
25311	SNOMED CT	26900001	Coronary ostium stenosis	Coronary Artery Disease
40398	SNOMED CT	42866003	Congenital coronary artery sclerosis	Coronary Artery Disease
52323	SNOMED CT	55546004	Anomalous origin of left circumflex artery from right coronary artery	Coronary Artery Disease
52996	SNOMED CT	56276002	Left main coronary artery thrombosis	Coronary Artery Disease
55618	SNOMED CT	59062007	Coronary stricture	Coronary Artery Disease
518419	ICD10CM	C00.0	Malignant neoplasm of external upper lip	Cancer
518420	ICD10CM	C00.1	Malignant neoplasm of external lower lip	Cancer
518421	ICD10CM	C00.2	Malignant neoplasm of external lip, unspecified	Cancer

518422	ICD10CM	C00.3	Malignant neoplasm of upper lip, inner aspect	Cancer
518423	ICD10CM	C00.4	Malignant neoplasm of lower lip, inner aspect	Cancer
518424	ICD10CM	C00.5	Malignant neoplasm of lip, unspecified, inner aspect	Cancer
518425	ICD10CM	C00.6	Malignant neoplasm of commissure of lip, unspecified	Cancer
518426	ICD10CM	C00.8	Malignant neoplasm of overlapping sites of lip	Cancer
518427	ICD10CM	C00.9	Malignant neoplasm of lip, unspecified	Cancer
518428	ICD10CM	C01	Malignant neoplasm of base of tongue	Cancer
518430	ICD10CM	C02.0	Malignant neoplasm of dorsal surface of tongue	Cancer
518431	ICD10CM	C02.1	Malignant neoplasm of border of tongue	Cancer
518432	ICD10CM	C02.2	Malignant neoplasm of ventral surface of tongue	Cancer
518433	ICD10CM	C02.3	Malignant neoplasm of anterior two-thirds of tongue, part unspecified	Cancer
518434	ICD10CM	C02.4	Malignant neoplasm of lingual tonsil	Cancer
518435	ICD10CM	C02.8	Malignant neoplasm of overlapping sites of tongue	Cancer
518436	ICD10CM	C02.9	Malignant neoplasm of tongue, unspecified	Cancer
518438	ICD10CM	C03.0	Malignant neoplasm of upper gum	Cancer
518439	ICD10CM	C03.1	Malignant neoplasm of lower gum	Cancer
518440	ICD10CM	C03.9	Malignant neoplasm of gum, unspecified	Cancer
518442	ICD10CM	C04.0	Malignant neoplasm of anterior floor of mouth	Cancer
518443	ICD10CM	C04.1	Malignant neoplasm of lateral floor of mouth	Cancer
518444	ICD10CM	C04.8	Malignant neoplasm of overlapping sites of floor of mouth	Cancer
518445	ICD10CM	C04.9	Malignant neoplasm of floor of mouth, unspecified	Cancer
518447	ICD10CM	C05.0	Malignant neoplasm of hard palate	Cancer
518448	ICD10CM	C05.1	Malignant neoplasm of soft palate	Cancer
518449	ICD10CM	C05.2	Malignant neoplasm of uvula	Cancer
518450	ICD10CM	C05.8	Malignant neoplasm of overlapping sites of palate	Cancer

518451	ICD10CM	C05.9	Malignant neoplasm of palate, unspecified	Cancer
518453	ICD10CM	C06.0	Malignant neoplasm of cheek mucosa	Cancer
518454	ICD10CM	C06.1	Malignant neoplasm of vestibule of mouth	Cancer
518455	ICD10CM	C06.2	Malignant neoplasm of retromolar area	Cancer
518457	ICD10CM	C06.80	Malignant neoplasm of overlapping sites of unspecified parts of mouth	Cancer
518458	ICD10CM	C06.89	Malignant neoplasm of overlapping sites of other parts of mouth	Cancer
518459	ICD10CM	C06.9	Malignant neoplasm of mouth, unspecified	Cancer
518460	ICD10CM	C07	Malignant neoplasm of parotid gland	Cancer
518462	ICD10CM	C08.0	Malignant neoplasm of submandibular gland	Cancer
518463	ICD10CM	C08.1	Malignant neoplasm of sublingual gland	Cancer
518464	ICD10CM	C08.9	Malignant neoplasm of major salivary gland, unspecified	Cancer
518466	ICD10CM	C09.0	Malignant neoplasm of tonsillar fossa	Cancer
518467	ICD10CM	C09.1	Malignant neoplasm of tonsillar pillar (anterior) (posterior)	Cancer
518468	ICD10CM	C09.8	Malignant neoplasm of overlapping sites of tonsil	Cancer
518469	ICD10CM	C09.9	Malignant neoplasm of tonsil, unspecified	Cancer
518471	ICD10CM	C10.0	Malignant neoplasm of vallecula	Cancer
518472	ICD10CM	C10.1	Malignant neoplasm of anterior surface of epiglottis	Cancer
518473	ICD10CM	C10.2	Malignant neoplasm of lateral wall of oropharynx	Cancer
518474	ICD10CM	C10.3	Malignant neoplasm of posterior wall of oropharynx	Cancer
518475	ICD10CM	C10.4	Malignant neoplasm of branchial cleft	Cancer
518476	ICD10CM	C10.8	Malignant neoplasm of overlapping sites of oropharynx	Cancer
518477	ICD10CM	C10.9	Malignant neoplasm of oropharynx, unspecified	Cancer
518479	ICD10CM	C11.0	Malignant neoplasm of superior wall of nasopharynx	Cancer
518480	ICD10CM	C11.1	Malignant neoplasm of posterior wall of nasopharynx	Cancer

518481	ICD10CM	C11.2	Malignant neoplasm of lateral wall of nasopharynx	Cancer
518482	ICD10CM	C11.3	Malignant neoplasm of anterior wall of nasopharynx	Cancer
518483	ICD10CM	C11.8	Malignant neoplasm of overlapping sites of nasopharynx	Cancer
518484	ICD10CM	C11.9	Malignant neoplasm of nasopharynx, unspecified	Cancer
518485	ICD10CM	C12	Malignant neoplasm of pyriform sinus	Cancer
518487	ICD10CM	C13.0	Malignant neoplasm of postcricoid region	Cancer
518488	ICD10CM	C13.1	Malignant neoplasm of aryepiglottic fold, hypopharyngeal aspect	Cancer
518489	ICD10CM	C13.2	Malignant neoplasm of posterior wall of hypopharynx	Cancer
518490	ICD10CM	C13.8	Malignant neoplasm of overlapping sites of hypopharynx	Cancer
518491	ICD10CM	C13.9	Malignant neoplasm of hypopharynx, unspecified	Cancer
518493	ICD10CM	C14.0	Malignant neoplasm of pharynx, unspecified	Cancer
518494	ICD10CM	C14.2	Malignant neoplasm of Waldeyer's ring	Cancer
518495	ICD10CM	C14.8	Malignant neoplasm of overlapping sites of lip, oral cavity and pharynx	Cancer
518497	ICD10CM	C15.3	Malignant neoplasm of upper third of esophagus	Cancer
518498	ICD10CM	C15.4	Malignant neoplasm of middle third of esophagus	Cancer
518499	ICD10CM	C15.5	Malignant neoplasm of lower third of esophagus	Cancer
518500	ICD10CM	C15.8	Malignant neoplasm of overlapping sites of esophagus	Cancer
518501	ICD10CM	C15.9	Malignant neoplasm of esophagus, unspecified	Cancer
518503	ICD10CM	C16.0	Malignant neoplasm of cardia	Cancer
518504	ICD10CM	C16.1	Malignant neoplasm of fundus of stomach	Cancer
518505	ICD10CM	C16.2	Malignant neoplasm of body of stomach	Cancer
518506	ICD10CM	C16.3	Malignant neoplasm of pyloric antrum	Cancer
518507	ICD10CM	C16.4	Malignant neoplasm of pylorus	Cancer
518508	ICD10CM	C16.5	Malignant neoplasm of lesser curvature of stomach, unspecified	Cancer

518509	ICD10CM	C16.6	Malignant neoplasm of greater curvature of stomach, unspecified	Cancer
518510	ICD10CM	C16.8	Malignant neoplasm of overlapping sites of stomach	Cancer
518511	ICD10CM	C16.9	Malignant neoplasm of stomach, unspecified	Cancer
518513	ICD10CM	C17.0	Malignant neoplasm of duodenum	Cancer
518514	ICD10CM	C17.1	Malignant neoplasm of jejunum	Cancer
518515	ICD10CM	C17.2	Malignant neoplasm of ileum	Cancer
518516	ICD10CM	C17.3	Meckel's diverticulum, malignant	Cancer
518517	ICD10CM	C17.8	Malignant neoplasm of overlapping sites of small intestine	Cancer
518518	ICD10CM	C17.9	Malignant neoplasm of small intestine, unspecified	Cancer
518520	ICD10CM	C18.0	Malignant neoplasm of cecum	Cancer
518521	ICD10CM	C18.1	Malignant neoplasm of appendix	Cancer
518522	ICD10CM	C18.2	Malignant neoplasm of ascending colon	Cancer
518523	ICD10CM	C18.3	Malignant neoplasm of hepatic flexure	Cancer
518524	ICD10CM	C18.4	Malignant neoplasm of transverse colon	Cancer
518525	ICD10CM	C18.5	Malignant neoplasm of splenic flexure	Cancer
518526	ICD10CM	C18.6	Malignant neoplasm of descending colon	Cancer
518527	ICD10CM	C18.7	Malignant neoplasm of sigmoid colon	Cancer
518528	ICD10CM	C18.8	Malignant neoplasm of overlapping sites of colon	Cancer
518529	ICD10CM	C18.9	Malignant neoplasm of colon, unspecified	Cancer
518530	ICD10CM	C19	Malignant neoplasm of rectosigmoid junction	Cancer
518531	ICD10CM	C20	Malignant neoplasm of rectum	Cancer
518535	ICD10CM	C21.2	Malignant neoplasm of cloacogenic zone	Cancer
518536	ICD10CM	C21.8	Malignant neoplasm of overlapping sites of rectum, anus and anal canal	Cancer
518562	ICD10CM	C26.0	Malignant neoplasm of intestinal tract, part unspecified	Cancer
518563	ICD10CM	C26.1	Malignant neoplasm of spleen	Cancer
518564	ICD10CM	C26.9	Malignant neoplasm of ill-defined sites within the digestive system	Cancer
518566	ICD10CM	C30.0	Malignant neoplasm of nasal cavity	Cancer

518567	ICD10CM	C30.1	Malignant neoplasm of middle ear	Cancer
518569	ICD10CM	C31.0	Malignant neoplasm of maxillary sinus	Cancer
518570	ICD10CM	C31.1	Malignant neoplasm of ethmoidal sinus	Cancer
518571	ICD10CM	C31.2	Malignant neoplasm of frontal sinus	Cancer
518572	ICD10CM	C31.3	Malignant neoplasm of sphenoid sinus	Cancer
518573	ICD10CM	C31.8	Malignant neoplasm of overlapping sites of accessory sinuses	Cancer
518574	ICD10CM	C31.9	Malignant neoplasm of accessory sinus, unspecified	Cancer
518576	ICD10CM	C32.0	Malignant neoplasm of glottis	Cancer
518577	ICD10CM	C32.1	Malignant neoplasm of supraglottis	Cancer
518578	ICD10CM	C32.2	Malignant neoplasm of subglottis	Cancer
518579	ICD10CM	C32.3	Malignant neoplasm of laryngeal cartilage	Cancer
518580	ICD10CM	C32.8	Malignant neoplasm of overlapping sites of larynx	Cancer
518581	ICD10CM	C32.9	Malignant neoplasm of larynx, unspecified	Cancer
518582	ICD10CM	C33	Malignant neoplasm of trachea	Cancer
518585	ICD10CM	C34.00	Malignant neoplasm of unspecified main bronchus	Cancer
518586	ICD10CM	C34.01	Malignant neoplasm of right main bronchus	Cancer
518587	ICD10CM	C34.02	Malignant neoplasm of left main bronchus	Cancer
518589	ICD10CM	C34.10	Malignant neoplasm of upper lobe, unspecified bronchus or lung	Cancer
518590	ICD10CM	C34.11	Malignant neoplasm of upper lobe, right bronchus or lung	Cancer
518591	ICD10CM	C34.12	Malignant neoplasm of upper lobe, left bronchus or lung	Cancer
518592	ICD10CM	C34.2	Malignant neoplasm of middle lobe, bronchus or lung	Cancer
518594	ICD10CM	C34.30	Malignant neoplasm of lower lobe, unspecified bronchus or lung	Cancer
518595	ICD10CM	C34.31	Malignant neoplasm of lower lobe, right bronchus or lung	Cancer
518596	ICD10CM	C34.32	Malignant neoplasm of lower lobe, left bronchus or lung	Cancer

518598	ICD10CM	C34.80	Malignant neoplasm of overlapping sites of unspecified bronchus and lung	Cancer
518599	ICD10CM	C34.81	Malignant neoplasm of overlapping sites of right bronchus and lung	Cancer
518600	ICD10CM	C34.82	Malignant neoplasm of overlapping sites of left bronchus and lung	Cancer
518602	ICD10CM	C34.90	Malignant neoplasm of unspecified part of unspecified bronchus or lung	Cancer
518603	ICD10CM	C34.91	Malignant neoplasm of unspecified part of right bronchus or lung	Cancer
518604	ICD10CM	C34.92	Malignant neoplasm of unspecified part of left bronchus or lung	Cancer
518605	ICD10CM	C37	Malignant neoplasm of thymus	Cancer
518607	ICD10CM	C38.0	Malignant neoplasm of heart	Cancer
518608	ICD10CM	C38.1	Malignant neoplasm of anterior mediastinum	Cancer
518609	ICD10CM	C38.2	Malignant neoplasm of posterior mediastinum	Cancer
518610	ICD10CM	C38.3	Malignant neoplasm of mediastinum, part unspecified	Cancer
518611	ICD10CM	C38.4	Malignant neoplasm of pleura	Cancer
518612	ICD10CM	C38.8	Malignant neoplasm of overlapping sites of heart, mediastinum and pleura	Cancer
518614	ICD10CM	C39.0	Malignant neoplasm of upper respiratory tract, part unspecified	Cancer
518615	ICD10CM	C39.9	Malignant neoplasm of lower respiratory tract, part unspecified	Cancer
518618	ICD10CM	C40.00	Malignant neoplasm of scapula and long bones of unspecified upper limb	Cancer
518619	ICD10CM	C40.01	Malignant neoplasm of scapula and long bones of right upper limb	Cancer
518620	ICD10CM	C40.02	Malignant neoplasm of scapula and long bones of left upper limb	Cancer
518622	ICD10CM	C40.10	Malignant neoplasm of short bones of unspecified upper limb	Cancer
518623	ICD10CM	C40.11	Malignant neoplasm of short bones of right upper limb	Cancer
518624	ICD10CM	C40.12	Malignant neoplasm of short bones of left upper limb	Cancer

518626	ICD10CM	C40.20	Malignant neoplasm of long bones of unspecified lower limb	Cancer
518627	ICD10CM	C40.21	Malignant neoplasm of long bones of right lower limb	Cancer
518628	ICD10CM	C40.22	Malignant neoplasm of long bones of left lower limb	Cancer
518630	ICD10CM	C40.30	Malignant neoplasm of short bones of unspecified lower limb	Cancer
518631	ICD10CM	C40.31	Malignant neoplasm of short bones of right lower limb	Cancer
518632	ICD10CM	C40.32	Malignant neoplasm of short bones of left lower limb	Cancer
518634	ICD10CM	C40.80	Malignant neoplasm of overlapping sites of bone and articular cartilage of unspecified limb	Cancer
518635	ICD10CM	C40.81	Malignant neoplasm of overlapping sites of bone and articular cartilage of right limb	Cancer
518636	ICD10CM	C40.82	Malignant neoplasm of overlapping sites of bone and articular cartilage of left limb	Cancer
518638	ICD10CM	C40.90	Malignant neoplasm of unspecified bones and articular cartilage of unspecified limb	Cancer
518639	ICD10CM	C40.91	Malignant neoplasm of unspecified bones and articular cartilage of right limb	Cancer
518640	ICD10CM	C40.92	Malignant neoplasm of unspecified bones and articular cartilage of left limb	Cancer
518642	ICD10CM	C41.0	Malignant neoplasm of bones of skull and face	Cancer
518643	ICD10CM	C41.1	Malignant neoplasm of mandible	Cancer
518644	ICD10CM	C41.2	Malignant neoplasm of vertebral column	Cancer
518645	ICD10CM	C41.3	Malignant neoplasm of ribs, sternum and clavicle	Cancer
518646	ICD10CM	C41.4	Malignant neoplasm of pelvic bones, sacrum and coccyx	Cancer
518647	ICD10CM	C41.9	Malignant neoplasm of bone and articular cartilage, unspecified	Cancer
518649	ICD10CM	C43.0	Malignant melanoma of lip	Cancer
518651	ICD10CM	C43.10	Malignant melanoma of unspecified eyelid, including canthus	Cancer
518652	ICD10CM	C43.11	Malignant melanoma of right eyelid, including canthus	Cancer
518655	ICD10CM	C43.12	Malignant melanoma of left eyelid, including canthus	Cancer

518659	ICD10CM	C43.20	Malignant melanoma of unspecified ear and external auricular canal	Cancer
518660	ICD10CM	C43.21	Malignant melanoma of right ear and external auricular canal	Cancer
518661	ICD10CM	C43.22	Malignant melanoma of left ear and external auricular canal	Cancer
518663	ICD10CM	C43.30	Malignant melanoma of unspecified part of face	Cancer
518664	ICD10CM	C43.31	Malignant melanoma of nose	Cancer
518665	ICD10CM	C43.39	Malignant melanoma of other parts of face	Cancer
518666	ICD10CM	C43.4	Malignant melanoma of scalp and neck	Cancer
518668	ICD10CM	C43.51	Malignant melanoma of anal skin	Cancer
518669	ICD10CM	C43.52	Malignant melanoma of skin of breast	Cancer
518670	ICD10CM	C43.59	Malignant melanoma of other part of trunk	Cancer
518672	ICD10CM	C43.60	Malignant melanoma of unspecified upper limb, including shoulder	Cancer
518673	ICD10CM	C43.61	Malignant melanoma of right upper limb, including shoulder	Cancer
518674	ICD10CM	C43.62	Malignant melanoma of left upper limb, including shoulder	Cancer
518676	ICD10CM	C43.70	Malignant melanoma of unspecified lower limb, including hip	Cancer
518677	ICD10CM	C43.71	Malignant melanoma of right lower limb, including hip	Cancer
518678	ICD10CM	C43.72	Malignant melanoma of left lower limb, including hip	Cancer
518679	ICD10CM	C43.8	Malignant melanoma of overlapping sites of skin	Cancer
518680	ICD10CM	C43.9	Malignant melanoma of skin, unspecified	Cancer
518683	ICD10CM	C44.00	Unspecified malignant neoplasm of skin of lip	Cancer
518684	ICD10CM	C44.01	Basal cell carcinoma of skin of lip	Cancer
518685	ICD10CM	C44.02	Squamous cell carcinoma of skin of lip	Cancer
518686	ICD10CM	C44.09	Other specified malignant neoplasm of skin of lip	Cancer
518689	ICD10CM	C44.101	Unspecified malignant neoplasm of skin of unspecified eyelid, including canthus	Cancer
518690	ICD10CM	C44.102	Unspecified malignant neoplasm of skin of right eyelid, including canthus	Cancer

518693	ICD10CM	C44.109	Unspecified malignant neoplasm of skin of left eyelid, including canthus	Cancer
518697	ICD10CM	C44.111	Basal cell carcinoma of skin of unspecified eyelid, including canthus	Cancer
518698	ICD10CM	C44.112	Basal cell carcinoma of skin of right eyelid, including canthus	Cancer
518701	ICD10CM	C44.119	Basal cell carcinoma of skin of left eyelid, including canthus	Cancer
518705	ICD10CM	C44.121	Squamous cell carcinoma of skin of unspecified eyelid, including canthus	Cancer
518706	ICD10CM	C44.122	Squamous cell carcinoma of skin of right eyelid, including canthus	Cancer
518709	ICD10CM	C44.129	Squamous cell carcinoma of skin of left eyelid, including canthus	Cancer
518721	ICD10CM	C44.191	Other specified malignant neoplasm of skin of unspecified eyelid, including canthus	Cancer
518722	ICD10CM	C44.192	Other specified malignant neoplasm of skin of right eyelid, including canthus	Cancer
518725	ICD10CM	C44.199	Other specified malignant neoplasm of skin of left eyelid, including canthus	Cancer
518730	ICD10CM	C44.201	Unspecified malignant neoplasm of skin of unspecified ear and external auricular canal	Cancer
518731	ICD10CM	C44.202	Unspecified malignant neoplasm of skin of right ear and external auricular canal	Cancer
518732	ICD10CM	C44.209	Unspecified malignant neoplasm of skin of left ear and external auricular canal	Cancer
518734	ICD10CM	C44.211	Basal cell carcinoma of skin of unspecified ear and external auricular canal	Cancer
518735	ICD10CM	C44.212	Basal cell carcinoma of skin of right ear and external auricular canal	Cancer
518736	ICD10CM	C44.219	Basal cell carcinoma of skin of left ear and external auricular canal	Cancer
518738	ICD10CM	C44.221	Squamous cell carcinoma of skin of unspecified ear and external auricular canal	Cancer
518739	ICD10CM	C44.222	Squamous cell carcinoma of skin of right ear and external auricular canal	Cancer

518740	ICD10CM	C44.229	Squamous cell carcinoma of skin of left ear and external auricular canal	Cancer
518742	ICD10CM	C44.291	Other specified malignant neoplasm of skin of unspecified ear and external auricular canal	Cancer
518743	ICD10CM	C44.292	Other specified malignant neoplasm of skin of right ear and external auricular canal	Cancer
518744	ICD10CM	C44.299	Other specified malignant neoplasm of skin of left ear and external auricular canal	Cancer
518747	ICD10CM	C44.300	Unspecified malignant neoplasm of skin of unspecified part of face	Cancer
518748	ICD10CM	C44.301	Unspecified malignant neoplasm of skin of nose	Cancer
518749	ICD10CM	C44.309	Unspecified malignant neoplasm of skin of other parts of face	Cancer
518751	ICD10CM	C44.310	Basal cell carcinoma of skin of unspecified parts of face	Cancer
518752	ICD10CM	C44.311	Basal cell carcinoma of skin of nose	Cancer
518753	ICD10CM	C44.319	Basal cell carcinoma of skin of other parts of face	Cancer
518755	ICD10CM	C44.320	Squamous cell carcinoma of skin of unspecified parts of face	Cancer
518756	ICD10CM	C44.321	Squamous cell carcinoma of skin of nose	Cancer
518757	ICD10CM	C44.329	Squamous cell carcinoma of skin of other parts of face	Cancer
518759	ICD10CM	C44.390	Other specified malignant neoplasm of skin of unspecified parts of face	Cancer
518760	ICD10CM	C44.391	Other specified malignant neoplasm of skin of nose	Cancer
518761	ICD10CM	C44.399	Other specified malignant neoplasm of skin of other parts of face	Cancer
518763	ICD10CM	C44.40	Unspecified malignant neoplasm of skin of scalp and neck	Cancer
518764	ICD10CM	C44.41	Basal cell carcinoma of skin of scalp and neck	Cancer
518765	ICD10CM	C44.42	Squamous cell carcinoma of skin of scalp and neck	Cancer
518766	ICD10CM	C44.49	Other specified malignant neoplasm of skin of scalp and neck	Cancer
518769	ICD10CM	C44.500	Unspecified malignant neoplasm of anal skin	Cancer

518770	ICD10CM	C44.501	Unspecified malignant neoplasm of skin of breast	Cancer
518771	ICD10CM	C44.509	Unspecified malignant neoplasm of skin of other part of trunk	Cancer
518773	ICD10CM	C44.510	Basal cell carcinoma of anal skin	Cancer
518774	ICD10CM	C44.511	Basal cell carcinoma of skin of breast	Cancer
518775	ICD10CM	C44.519	Basal cell carcinoma of skin of other part of trunk	Cancer
518777	ICD10CM	C44.520	Squamous cell carcinoma of anal skin	Cancer
518778	ICD10CM	C44.521	Squamous cell carcinoma of skin of breast	Cancer
518779	ICD10CM	C44.529	Squamous cell carcinoma of skin of other part of trunk	Cancer
518781	ICD10CM	C44.590	Other specified malignant neoplasm of anal skin	Cancer
518782	ICD10CM	C44.591	Other specified malignant neoplasm of skin of breast	Cancer
518783	ICD10CM	C44.599	Other specified malignant neoplasm of skin of other part of trunk	Cancer
518786	ICD10CM	C44.601	Unspecified malignant neoplasm of skin of unspecified upper limb, including shoulder	Cancer
518787	ICD10CM	C44.602	Unspecified malignant neoplasm of skin of right upper limb, including shoulder	Cancer
518788	ICD10CM	C44.609	Unspecified malignant neoplasm of skin of left upper limb, including shoulder	Cancer
518790	ICD10CM	C44.611	Basal cell carcinoma of skin of unspecified upper limb, including shoulder	Cancer
518791	ICD10CM	C44.612	Basal cell carcinoma of skin of right upper limb, including shoulder	Cancer
518792	ICD10CM	C44.619	Basal cell carcinoma of skin of left upper limb, including shoulder	Cancer
518794	ICD10CM	C44.621	Squamous cell carcinoma of skin of unspecified upper limb, including shoulder	Cancer
518795	ICD10CM	C44.622	Squamous cell carcinoma of skin of right upper limb, including shoulder	Cancer
518796	ICD10CM	C44.629	Squamous cell carcinoma of skin of left upper limb, including shoulder	Cancer

518798	ICD10CM	C44.691	Other specified malignant neoplasm of skin of unspecified upper limb, including shoulder	Cancer
518799	ICD10CM	C44.692	Other specified malignant neoplasm of skin of right upper limb, including shoulder	Cancer
518800	ICD10CM	C44.699	Other specified malignant neoplasm of skin of left upper limb, including shoulder	Cancer
518803	ICD10CM	C44.701	Unspecified malignant neoplasm of skin of unspecified lower limb, including hip	Cancer
518804	ICD10CM	C44.702	Unspecified malignant neoplasm of skin of right lower limb, including hip	Cancer
518805	ICD10CM	C44.709	Unspecified malignant neoplasm of skin of left lower limb, including hip	Cancer
518807	ICD10CM	C44.711	Basal cell carcinoma of skin of unspecified lower limb, including hip	Cancer
518808	ICD10CM	C44.712	Basal cell carcinoma of skin of right lower limb, including hip	Cancer
518809	ICD10CM	C44.719	Basal cell carcinoma of skin of left lower limb, including hip	Cancer
518811	ICD10CM	C44.721	Squamous cell carcinoma of skin of unspecified lower limb, including hip	Cancer
518812	ICD10CM	C44.722	Squamous cell carcinoma of skin of right lower limb, including hip	Cancer
518813	ICD10CM	C44.729	Squamous cell carcinoma of skin of left lower limb, including hip	Cancer
518815	ICD10CM	C44.791	Other specified malignant neoplasm of skin of unspecified lower limb, including hip	Cancer
518816	ICD10CM	C44.792	Other specified malignant neoplasm of skin of right lower limb, including hip	Cancer
518817	ICD10CM	C44.799	Other specified malignant neoplasm of skin of left lower limb, including hip	Cancer
518819	ICD10CM	C44.80	Unspecified malignant neoplasm of overlapping sites of skin	Cancer
518820	ICD10CM	C44.81	Basal cell carcinoma of overlapping sites of skin	Cancer
518821	ICD10CM	C44.82	Squamous cell carcinoma of overlapping sites of skin	Cancer
518822	ICD10CM	C44.89	Other specified malignant neoplasm of overlapping sites of skin	Cancer

518824	ICD10CM	C44.90	Unspecified malignant neoplasm of skin, unspecified	Cancer
518825	ICD10CM	C44.91	Basal cell carcinoma of skin, unspecified	Cancer
518826	ICD10CM	C44.92	Squamous cell carcinoma of skin, unspecified	Cancer
518827	ICD10CM	C44.99	Other specified malignant neoplasm of skin, unspecified	Cancer
518829	ICD10CM	C45.0	Mesothelioma of pleura	Cancer
518830	ICD10CM	C45.1	Mesothelioma of peritoneum	Cancer
518831	ICD10CM	C45.2	Mesothelioma of pericardium	Cancer
518832	ICD10CM	C45.7	Mesothelioma of other sites	Cancer
518833	ICD10CM	C45.9	Mesothelioma, unspecified	Cancer
518847	ICD10CM	C47.0	Malignant neoplasm of peripheral nerves of head, face and neck	Cancer
518849	ICD10CM	C47.10	Malignant neoplasm of peripheral nerves of unspecified upper limb, including shoulder	Cancer
518850	ICD10CM	C47.11	Malignant neoplasm of peripheral nerves of right upper limb, including shoulder	Cancer
518851	ICD10CM	C47.12	Malignant neoplasm of peripheral nerves of left upper limb, including shoulder	Cancer
518853	ICD10CM	C47.20	Malignant neoplasm of peripheral nerves of unspecified lower limb, including hip	Cancer
518854	ICD10CM	C47.21	Malignant neoplasm of peripheral nerves of right lower limb, including hip	Cancer
518855	ICD10CM	C47.22	Malignant neoplasm of peripheral nerves of left lower limb, including hip	Cancer
518856	ICD10CM	C47.3	Malignant neoplasm of peripheral nerves of thorax	Cancer
518857	ICD10CM	C47.4	Malignant neoplasm of peripheral nerves of abdomen	Cancer
518858	ICD10CM	C47.5	Malignant neoplasm of peripheral nerves of pelvis	Cancer
518859	ICD10CM	C47.6	Malignant neoplasm of peripheral nerves of trunk, unspecified	Cancer
518860	ICD10CM	C47.8	Malignant neoplasm of overlapping sites of peripheral nerves and autonomic nervous system	Cancer
518861	ICD10CM	C47.9	Malignant neoplasm of peripheral nerves and autonomic nervous system, unspecified	Cancer

518863	ICD10CM	C48.0	Malignant neoplasm of retroperitoneum	Cancer
518864	ICD10CM	C48.1	Malignant neoplasm of specified parts of peritoneum	Cancer
518865	ICD10CM	C48.2	Malignant neoplasm of peritoneum, unspecified	Cancer
518866	ICD10CM	C48.8	Malignant neoplasm of overlapping sites of retroperitoneum and peritoneum	Cancer
518868	ICD10CM	C49.0	Malignant neoplasm of connective and soft tissue of head, face and neck	Cancer
518870	ICD10CM	C49.10	Malignant neoplasm of connective and soft tissue of unspecified upper limb, including shoulder	Cancer
518871	ICD10CM	C49.11	Malignant neoplasm of connective and soft tissue of right upper limb, including shoulder	Cancer
518872	ICD10CM	C49.12	Malignant neoplasm of connective and soft tissue of left upper limb, including shoulder	Cancer
518874	ICD10CM	C49.20	Malignant neoplasm of connective and soft tissue of unspecified lower limb, including hip	Cancer
518875	ICD10CM	C49.21	Malignant neoplasm of connective and soft tissue of right lower limb, including hip	Cancer
518876	ICD10CM	C49.22	Malignant neoplasm of connective and soft tissue of left lower limb, including hip	Cancer
518877	ICD10CM	C49.3	Malignant neoplasm of connective and soft tissue of thorax	Cancer
518878	ICD10CM	C49.4	Malignant neoplasm of connective and soft tissue of abdomen	Cancer
518879	ICD10CM	C49.5	Malignant neoplasm of connective and soft tissue of pelvis	Cancer
518880	ICD10CM	C49.6	Malignant neoplasm of connective and soft tissue of trunk, unspecified	Cancer
518881	ICD10CM	C49.8	Malignant neoplasm of overlapping sites of connective and soft tissue	Cancer
518882	ICD10CM	C49.9	Malignant neoplasm of connective and soft tissue, unspecified	Cancer

518884	ICD10CM	C49.A0	Gastrointestinal stromal tumor, unspecified site	Cancer
518885	ICD10CM	C49.A1	Gastrointestinal stromal tumor of esophagus	Cancer
518886	ICD10CM	C49.A2	Gastrointestinal stromal tumor of stomach	Cancer
518887	ICD10CM	C49.A3	Gastrointestinal stromal tumor of small intestine	Cancer
518888	ICD10CM	C49.A4	Gastrointestinal stromal tumor of large intestine	Cancer
518889	ICD10CM	C49.A5	Gastrointestinal stromal tumor of rectum	Cancer
518890	ICD10CM	C49.A9	Gastrointestinal stromal tumor of other sites	Cancer
518892	ICD10CM	C4A.0	Merkel cell carcinoma of lip	Cancer
518894	ICD10CM	C4A.10	Merkel cell carcinoma of unspecified eyelid, including canthus	Cancer
518895	ICD10CM	C4A.11	Merkel cell carcinoma of right eyelid, including canthus	Cancer
518898	ICD10CM	C4A.12	Merkel cell carcinoma of left eyelid, including canthus	Cancer
518902	ICD10CM	C4A.20	Merkel cell carcinoma of unspecified ear and external auricular canal	Cancer
518903	ICD10CM	C4A.21	Merkel cell carcinoma of right ear and external auricular canal	Cancer
518904	ICD10CM	C4A.22	Merkel cell carcinoma of left ear and external auricular canal	Cancer
518906	ICD10CM	C4A.30	Merkel cell carcinoma of unspecified part of face	Cancer
518907	ICD10CM	C4A.31	Merkel cell carcinoma of nose	Cancer
518908	ICD10CM	C4A.39	Merkel cell carcinoma of other parts of face	Cancer
518909	ICD10CM	C4A.4	Merkel cell carcinoma of scalp and neck	Cancer
518911	ICD10CM	C4A.51	Merkel cell carcinoma of anal skin	Cancer
518912	ICD10CM	C4A.52	Merkel cell carcinoma of skin of breast	Cancer
518913	ICD10CM	C4A.59	Merkel cell carcinoma of other part of trunk	Cancer
518915	ICD10CM	C4A.60	Merkel cell carcinoma of unspecified upper limb, including shoulder	Cancer
518916	ICD10CM	C4A.61	Merkel cell carcinoma of right upper limb, including shoulder	Cancer
518917	ICD10CM	C4A.62	Merkel cell carcinoma of left upper limb, including shoulder	Cancer

518919	ICD10CM	C4A.70	Merkel cell carcinoma of unspecified lower limb, including hip	Cancer
518920	ICD10CM	C4A.71	Merkel cell carcinoma of right lower limb, including hip	Cancer
518921	ICD10CM	C4A.72	Merkel cell carcinoma of left lower limb, including hip	Cancer
518922	ICD10CM	C4A.8	Merkel cell carcinoma of overlapping sites	Cancer
518923	ICD10CM	C4A.9	Merkel cell carcinoma, unspecified	Cancer
518927	ICD10CM	C50.011	Malignant neoplasm of nipple and areola, right female breast	Cancer
518928	ICD10CM	C50.012	Malignant neoplasm of nipple and areola, left female breast	Cancer
518929	ICD10CM	C50.019	Malignant neoplasm of nipple and areola, unspecified female breast	Cancer
518931	ICD10CM	C50.021	Malignant neoplasm of nipple and areola, right male breast	Cancer
518932	ICD10CM	C50.022	Malignant neoplasm of nipple and areola, left male breast	Cancer
518933	ICD10CM	C50.029	Malignant neoplasm of nipple and areola, unspecified male breast	Cancer
518936	ICD10CM	C50.111	Malignant neoplasm of central portion of right female breast	Cancer
518937	ICD10CM	C50.112	Malignant neoplasm of central portion of left female breast	Cancer
518938	ICD10CM	C50.119	Malignant neoplasm of central portion of unspecified female breast	Cancer
518940	ICD10CM	C50.121	Malignant neoplasm of central portion of right male breast	Cancer
518941	ICD10CM	C50.122	Malignant neoplasm of central portion of left male breast	Cancer
518942	ICD10CM	C50.129	Malignant neoplasm of central portion of unspecified male breast	Cancer
518945	ICD10CM	C50.211	Malignant neoplasm of upper-inner quadrant of right female breast	Cancer
518946	ICD10CM	C50.212	Malignant neoplasm of upper-inner quadrant of left female breast	Cancer
518947	ICD10CM	C50.219	Malignant neoplasm of upper-inner quadrant of unspecified female breast	Cancer
518949	ICD10CM	C50.221	Malignant neoplasm of upper-inner quadrant of right male breast	Cancer

518950	ICD10CM	C50.222	Malignant neoplasm of upper-inner quadrant of left male breast	Cancer
518951	ICD10CM	C50.229	Malignant neoplasm of upper-inner quadrant of unspecified male breast	Cancer
518954	ICD10CM	C50.311	Malignant neoplasm of lower-inner quadrant of right female breast	Cancer
518955	ICD10CM	C50.312	Malignant neoplasm of lower-inner quadrant of left female breast	Cancer
518956	ICD10CM	C50.319	Malignant neoplasm of lower-inner quadrant of unspecified female breast	Cancer
518958	ICD10CM	C50.321	Malignant neoplasm of lower-inner quadrant of right male breast	Cancer
518959	ICD10CM	C50.322	Malignant neoplasm of lower-inner quadrant of left male breast	Cancer
518960	ICD10CM	C50.329	Malignant neoplasm of lower-inner quadrant of unspecified male breast	Cancer
518963	ICD10CM	C50.411	Malignant neoplasm of upper-outer quadrant of right female breast	Cancer
518964	ICD10CM	C50.412	Malignant neoplasm of upper-outer quadrant of left female breast	Cancer
518965	ICD10CM	C50.419	Malignant neoplasm of upper-outer quadrant of unspecified female breast	Cancer
518967	ICD10CM	C50.421	Malignant neoplasm of upper-outer quadrant of right male breast	Cancer
518968	ICD10CM	C50.422	Malignant neoplasm of upper-outer quadrant of left male breast	Cancer
518969	ICD10CM	C50.429	Malignant neoplasm of upper-outer quadrant of unspecified male breast	Cancer
518972	ICD10CM	C50.511	Malignant neoplasm of lower-outer quadrant of right female breast	Cancer
518973	ICD10CM	C50.512	Malignant neoplasm of lower-outer quadrant of left female breast	Cancer
518974	ICD10CM	C50.519	Malignant neoplasm of lower-outer quadrant of unspecified female breast	Cancer

518976	ICD10CM	C50.521	Malignant neoplasm of lower-outer quadrant of right male breast	Cancer
518977	ICD10CM	C50.522	Malignant neoplasm of lower-outer quadrant of left male breast	Cancer
518978	ICD10CM	C50.529	Malignant neoplasm of lower-outer quadrant of unspecified male breast	Cancer
518981	ICD10CM	C50.611	Malignant neoplasm of axillary tail of right female breast	Cancer
518982	ICD10CM	C50.612	Malignant neoplasm of axillary tail of left female breast	Cancer
518983	ICD10CM	C50.619	Malignant neoplasm of axillary tail of unspecified female breast	Cancer
518985	ICD10CM	C50.621	Malignant neoplasm of axillary tail of right male breast	Cancer
518986	ICD10CM	C50.622	Malignant neoplasm of axillary tail of left male breast	Cancer
518987	ICD10CM	C50.629	Malignant neoplasm of axillary tail of unspecified male breast	Cancer
518990	ICD10CM	C50.811	Malignant neoplasm of overlapping sites of right female breast	Cancer
518991	ICD10CM	C50.812	Malignant neoplasm of overlapping sites of left female breast	Cancer
518992	ICD10CM	C50.819	Malignant neoplasm of overlapping sites of unspecified female breast	Cancer
518994	ICD10CM	C50.821	Malignant neoplasm of overlapping sites of right male breast	Cancer
518995	ICD10CM	C50.822	Malignant neoplasm of overlapping sites of left male breast	Cancer
518996	ICD10CM	C50.829	Malignant neoplasm of overlapping sites of unspecified male breast	Cancer
518999	ICD10CM	C50.911	Malignant neoplasm of unspecified site of right female breast	Cancer
519000	ICD10CM	C50.912	Malignant neoplasm of unspecified site of left female breast	Cancer
519001	ICD10CM	C50.919	Malignant neoplasm of unspecified site of unspecified female breast	Cancer
519003	ICD10CM	C50.921	Malignant neoplasm of unspecified site of right male breast	Cancer

519004	ICD10CM	C50.922	Malignant neoplasm of unspecified site of left male breast	Cancer
519005	ICD10CM	C50.929	Malignant neoplasm of unspecified site of unspecified male breast	Cancer
519007	ICD10CM	C51.0	Malignant neoplasm of labium majus	Cancer
519008	ICD10CM	C51.1	Malignant neoplasm of labium minus	Cancer
519009	ICD10CM	C51.2	Malignant neoplasm of clitoris	Cancer
519010	ICD10CM	C51.8	Malignant neoplasm of overlapping sites of vulva	Cancer
519011	ICD10CM	C51.9	Malignant neoplasm of vulva, unspecified	Cancer
519012	ICD10CM	C52	Malignant neoplasm of vagina	Cancer
519014	ICD10CM	C53.0	Malignant neoplasm of endocervix	Cancer
519015	ICD10CM	C53.1	Malignant neoplasm of exocervix	Cancer
519016	ICD10CM	C53.8	Malignant neoplasm of overlapping sites of cervix uteri	Cancer
519017	ICD10CM	C53.9	Malignant neoplasm of cervix uteri, unspecified	Cancer
519019	ICD10CM	C54.0	Malignant neoplasm of isthmus uteri	Cancer
519020	ICD10CM	C54.1	Malignant neoplasm of endometrium	Cancer
519021	ICD10CM	C54.2	Malignant neoplasm of myometrium	Cancer
519022	ICD10CM	C54.3	Malignant neoplasm of fundus uteri	Cancer
519023	ICD10CM	C54.8	Malignant neoplasm of overlapping sites of corpus uteri	Cancer
519024	ICD10CM	C54.9	Malignant neoplasm of corpus uteri, unspecified	Cancer
519025	ICD10CM	C55	Malignant neoplasm of uterus, part unspecified	Cancer
519028	ICD10CM	C56.1	Malignant neoplasm of right ovary	Cancer
519029	ICD10CM	C56.2	Malignant neoplasm of left ovary	Cancer
519030	ICD10CM	C56.9	Malignant neoplasm of unspecified ovary	Cancer
519033	ICD10CM	C57.00	Malignant neoplasm of unspecified fallopian tube	Cancer
519034	ICD10CM	C57.01	Malignant neoplasm of right fallopian tube	Cancer
519035	ICD10CM	C57.02	Malignant neoplasm of left fallopian tube	Cancer
519037	ICD10CM	C57.10	Malignant neoplasm of unspecified broad ligament	Cancer

519038	ICD10CM	C57.11	Malignant neoplasm of right broad ligament	Cancer
519039	ICD10CM	C57.12	Malignant neoplasm of left broad ligament	Cancer
519041	ICD10CM	C57.20	Malignant neoplasm of unspecified round ligament	Cancer
519042	ICD10CM	C57.21	Malignant neoplasm of right round ligament	Cancer
519043	ICD10CM	C57.22	Malignant neoplasm of left round ligament	Cancer
519044	ICD10CM	C57.3	Malignant neoplasm of parametrium	Cancer
519045	ICD10CM	C57.4	Malignant neoplasm of uterine adnexa, unspecified	Cancer
519046	ICD10CM	C57.7	Malignant neoplasm of other specified female genital organs	Cancer
519047	ICD10CM	C57.8	Malignant neoplasm of overlapping sites of female genital organs	Cancer
519048	ICD10CM	C57.9	Malignant neoplasm of female genital organ, unspecified	Cancer
519049	ICD10CM	C58	Malignant neoplasm of placenta	Cancer
519051	ICD10CM	C60.0	Malignant neoplasm of prepuce	Cancer
519052	ICD10CM	C60.1	Malignant neoplasm of glans penis	Cancer
519053	ICD10CM	C60.2	Malignant neoplasm of body of penis	Cancer
519054	ICD10CM	C60.8	Malignant neoplasm of overlapping sites of penis	Cancer
519055	ICD10CM	C60.9	Malignant neoplasm of penis, unspecified	Cancer
519056	ICD10CM	C61	Malignant neoplasm of prostate	Cancer
519059	ICD10CM	C62.00	Malignant neoplasm of unspecified undescended testis	Cancer
519060	ICD10CM	C62.01	Malignant neoplasm of undescended right testis	Cancer
519061	ICD10CM	C62.02	Malignant neoplasm of undescended left testis	Cancer
519063	ICD10CM	C62.10	Malignant neoplasm of unspecified descended testis	Cancer
519064	ICD10CM	C62.11	Malignant neoplasm of descended right testis	Cancer
519065	ICD10CM	C62.12	Malignant neoplasm of descended left testis	Cancer
519067	ICD10CM	C62.90	Malignant neoplasm of unspecified testis, unspecified whether descended or undescended	Cancer

519068	ICD10CM	C62.91	Malignant neoplasm of right testis, unspecified whether descended or undescended	Cancer
519069	ICD10CM	C62.92	Malignant neoplasm of left testis, unspecified whether descended or undescended	Cancer
519072	ICD10CM	C63.00	Malignant neoplasm of unspecified epididymis	Cancer
519073	ICD10CM	C63.01	Malignant neoplasm of right epididymis	Cancer
519074	ICD10CM	C63.02	Malignant neoplasm of left epididymis	Cancer
519076	ICD10CM	C63.10	Malignant neoplasm of unspecified spermatic cord	Cancer
519077	ICD10CM	C63.11	Malignant neoplasm of right spermatic cord	Cancer
519078	ICD10CM	C63.12	Malignant neoplasm of left spermatic cord	Cancer
519079	ICD10CM	C63.2	Malignant neoplasm of scrotum	Cancer
519080	ICD10CM	C63.7	Malignant neoplasm of other specified male genital organs	Cancer
519081	ICD10CM	C63.8	Malignant neoplasm of overlapping sites of male genital organs	Cancer
519082	ICD10CM	C63.9	Malignant neoplasm of male genital organ, unspecified	Cancer
519085	ICD10CM	C64.1	Malignant neoplasm of right kidney, except renal pelvis	Cancer
519086	ICD10CM	C64.2	Malignant neoplasm of left kidney, except renal pelvis	Cancer
519087	ICD10CM	C64.9	Malignant neoplasm of unspecified kidney, except renal pelvis	Cancer
519090	ICD10CM	C65.1	Malignant neoplasm of right renal pelvis	Cancer
519091	ICD10CM	C65.2	Malignant neoplasm of left renal pelvis	Cancer
519092	ICD10CM	C65.9	Malignant neoplasm of unspecified renal pelvis	Cancer
519095	ICD10CM	C66.1	Malignant neoplasm of right ureter	Cancer
519096	ICD10CM	C66.2	Malignant neoplasm of left ureter	Cancer
519097	ICD10CM	C66.9	Malignant neoplasm of unspecified ureter	Cancer
519099	ICD10CM	C67.0	Malignant neoplasm of trigone of bladder	Cancer
519100	ICD10CM	C67.1	Malignant neoplasm of dome of bladder	Cancer
519101	ICD10CM	C67.2	Malignant neoplasm of lateral wall of bladder	Cancer

519102	ICD10CM	C67.3	Malignant neoplasm of anterior wall of bladder	Cancer
519103	ICD10CM	C67.4	Malignant neoplasm of posterior wall of bladder	Cancer
519104	ICD10CM	C67.5	Malignant neoplasm of bladder neck	Cancer
519105	ICD10CM	C67.6	Malignant neoplasm of ureteric orifice	Cancer
519106	ICD10CM	C67.7	Malignant neoplasm of urachus	Cancer
519107	ICD10CM	C67.8	Malignant neoplasm of overlapping sites of bladder	Cancer
519108	ICD10CM	C67.9	Malignant neoplasm of bladder, unspecified	Cancer
519110	ICD10CM	C68.0	Malignant neoplasm of urethra	Cancer
519111	ICD10CM	C68.1	Malignant neoplasm of paraurethral glands	Cancer
519112	ICD10CM	C68.8	Malignant neoplasm of overlapping sites of urinary organs	Cancer
519113	ICD10CM	C68.9	Malignant neoplasm of urinary organ, unspecified	Cancer
519116	ICD10CM	C69.00	Malignant neoplasm of unspecified conjunctiva	Cancer
519117	ICD10CM	C69.01	Malignant neoplasm of right conjunctiva	Cancer
519118	ICD10CM	C69.02	Malignant neoplasm of left conjunctiva	Cancer
519120	ICD10CM	C69.10	Malignant neoplasm of unspecified cornea	Cancer
519121	ICD10CM	C69.11	Malignant neoplasm of right cornea	Cancer
519122	ICD10CM	C69.12	Malignant neoplasm of left cornea	Cancer
519124	ICD10CM	C69.20	Malignant neoplasm of unspecified retina	Cancer
519125	ICD10CM	C69.21	Malignant neoplasm of right retina	Cancer
519126	ICD10CM	C69.22	Malignant neoplasm of left retina	Cancer
519128	ICD10CM	C69.30	Malignant neoplasm of unspecified choroid	Cancer
519129	ICD10CM	C69.31	Malignant neoplasm of right choroid	Cancer
519130	ICD10CM	C69.32	Malignant neoplasm of left choroid	Cancer
519132	ICD10CM	C69.40	Malignant neoplasm of unspecified ciliary body	Cancer
519133	ICD10CM	C69.41	Malignant neoplasm of right ciliary body	Cancer
519134	ICD10CM	C69.42	Malignant neoplasm of left ciliary body	Cancer

519136	ICD10CM	C69.50	Malignant neoplasm of unspecified lacrimal gland and duct	Cancer
519137	ICD10CM	C69.51	Malignant neoplasm of right lacrimal gland and duct	Cancer
519138	ICD10CM	C69.52	Malignant neoplasm of left lacrimal gland and duct	Cancer
519140	ICD10CM	C69.60	Malignant neoplasm of unspecified orbit	Cancer
519141	ICD10CM	C69.61	Malignant neoplasm of right orbit	Cancer
519142	ICD10CM	C69.62	Malignant neoplasm of left orbit	Cancer
519144	ICD10CM	C69.80	Malignant neoplasm of overlapping sites of unspecified eye and adnexa	Cancer
519145	ICD10CM	C69.81	Malignant neoplasm of overlapping sites of right eye and adnexa	Cancer
519146	ICD10CM	C69.82	Malignant neoplasm of overlapping sites of left eye and adnexa	Cancer
519148	ICD10CM	C69.90	Malignant neoplasm of unspecified site of unspecified eye	Cancer
519149	ICD10CM	C69.91	Malignant neoplasm of unspecified site of right eye	Cancer
519150	ICD10CM	C69.92	Malignant neoplasm of unspecified site of left eye	Cancer
519152	ICD10CM	C70.0	Malignant neoplasm of cerebral meninges	Cancer
519153	ICD10CM	C70.1	Malignant neoplasm of spinal meninges	Cancer
519154	ICD10CM	C70.9	Malignant neoplasm of meninges, unspecified	Cancer
519156	ICD10CM	C71.0	Malignant neoplasm of cerebrum, except lobes and ventricles	Cancer
519157	ICD10CM	C71.1	Malignant neoplasm of frontal lobe	Cancer
519158	ICD10CM	C71.2	Malignant neoplasm of temporal lobe	Cancer
519159	ICD10CM	C71.3	Malignant neoplasm of parietal lobe	Cancer
519160	ICD10CM	C71.4	Malignant neoplasm of occipital lobe	Cancer
519161	ICD10CM	C71.5	Malignant neoplasm of cerebral ventricle	Cancer
519162	ICD10CM	C71.6	Malignant neoplasm of cerebellum	Cancer
519163	ICD10CM	C71.7	Malignant neoplasm of brain stem	Cancer

519164	ICD10CM	C71.8	Malignant neoplasm of overlapping sites of brain	Cancer
519165	ICD10CM	C71.9	Malignant neoplasm of brain, unspecified	Cancer
519167	ICD10CM	C72.0	Malignant neoplasm of spinal cord	Cancer
519168	ICD10CM	C72.1	Malignant neoplasm of cauda equina	Cancer
519170	ICD10CM	C72.20	Malignant neoplasm of unspecified olfactory nerve	Cancer
519171	ICD10CM	C72.21	Malignant neoplasm of right olfactory nerve	Cancer
519172	ICD10CM	C72.22	Malignant neoplasm of left olfactory nerve	Cancer
519174	ICD10CM	C72.30	Malignant neoplasm of unspecified optic nerve	Cancer
519175	ICD10CM	C72.31	Malignant neoplasm of right optic nerve	Cancer
519176	ICD10CM	C72.32	Malignant neoplasm of left optic nerve	Cancer
519178	ICD10CM	C72.40	Malignant neoplasm of unspecified acoustic nerve	Cancer
519179	ICD10CM	C72.41	Malignant neoplasm of right acoustic nerve	Cancer
519180	ICD10CM	C72.42	Malignant neoplasm of left acoustic nerve	Cancer
519182	ICD10CM	C72.50	Malignant neoplasm of unspecified cranial nerve	Cancer
519183	ICD10CM	C72.59	Malignant neoplasm of other cranial nerves	Cancer
519184	ICD10CM	C72.9	Malignant neoplasm of central nervous system, unspecified	Cancer
519185	ICD10CM	C73	Malignant neoplasm of thyroid gland	Cancer
519188	ICD10CM	C74.00	Malignant neoplasm of cortex of unspecified adrenal gland	Cancer
519189	ICD10CM	C74.01	Malignant neoplasm of cortex of right adrenal gland	Cancer
519190	ICD10CM	C74.02	Malignant neoplasm of cortex of left adrenal gland	Cancer
519192	ICD10CM	C74.10	Malignant neoplasm of medulla of unspecified adrenal gland	Cancer
519193	ICD10CM	C74.11	Malignant neoplasm of medulla of right adrenal gland	Cancer
519194	ICD10CM	C74.12	Malignant neoplasm of medulla of left adrenal gland	Cancer
519196	ICD10CM	C74.90	Malignant neoplasm of unspecified part of unspecified adrenal gland	Cancer

519197	ICD10CM	C74.91	Malignant neoplasm of unspecified part of right adrenal gland	Cancer
519198	ICD10CM	C74.92	Malignant neoplasm of unspecified part of left adrenal gland	Cancer
519200	ICD10CM	C75.0	Malignant neoplasm of parathyroid gland	Cancer
519201	ICD10CM	C75.1	Malignant neoplasm of pituitary gland	Cancer
519202	ICD10CM	C75.2	Malignant neoplasm of craniopharyngeal duct	Cancer
519203	ICD10CM	C75.3	Malignant neoplasm of pineal gland	Cancer
519204	ICD10CM	C75.4	Malignant neoplasm of carotid body	Cancer
519205	ICD10CM	C75.5	Malignant neoplasm of aortic body and other paraganglia	Cancer
519206	ICD10CM	C75.8	Malignant neoplasm with pluriglandular involvement, unspecified	Cancer
519207	ICD10CM	C75.9	Malignant neoplasm of endocrine gland, unspecified	Cancer
519209	ICD10CM	C76.0	Malignant neoplasm of head, face and neck	Cancer
519210	ICD10CM	C76.1	Malignant neoplasm of thorax	Cancer
519211	ICD10CM	C76.2	Malignant neoplasm of abdomen	Cancer
519212	ICD10CM	C76.3	Malignant neoplasm of pelvis	Cancer
519214	ICD10CM	C76.40	Malignant neoplasm of unspecified upper limb	Cancer
519215	ICD10CM	C76.41	Malignant neoplasm of right upper limb	Cancer
519216	ICD10CM	C76.42	Malignant neoplasm of left upper limb	Cancer
519218	ICD10CM	C76.50	Malignant neoplasm of unspecified lower limb	Cancer
519219	ICD10CM	C76.51	Malignant neoplasm of right lower limb	Cancer
519220	ICD10CM	C76.52	Malignant neoplasm of left lower limb	Cancer
519222	ICD10CM	C76.8	Malignant neoplasm of other specified ill-defined sites	Cancer
519224	ICD10CM	C77.0	Secondary and unspecified malignant neoplasm of lymph nodes of head, face and neck	Cancer
519225	ICD10CM	C77.1	Secondary and unspecified malignant neoplasm of intrathoracic lymph nodes	Cancer

519226	ICD10CM	C77.2	Secondary and unspecified malignant neoplasm of intra-abdominal lymph nodes	Cancer
519227	ICD10CM	C77.3	Secondary and unspecified malignant neoplasm of axilla and upper limb lymph nodes	Cancer
519228	ICD10CM	C77.4	Secondary and unspecified malignant neoplasm of inguinal and lower limb lymph nodes	Cancer
519229	ICD10CM	C77.5	Secondary and unspecified malignant neoplasm of intrapelvic lymph nodes	Cancer
519230	ICD10CM	C77.8	Secondary and unspecified malignant neoplasm of lymph nodes of multiple regions	Cancer
519231	ICD10CM	C77.9	Secondary and unspecified malignant neoplasm of lymph node, unspecified	Cancer
519234	ICD10CM	C78.00	Secondary malignant neoplasm of unspecified lung	Cancer
519235	ICD10CM	C78.01	Secondary malignant neoplasm of right lung	Cancer
519236	ICD10CM	C78.02	Secondary malignant neoplasm of left lung	Cancer
519237	ICD10CM	C78.1	Secondary malignant neoplasm of mediastinum	Cancer
519238	ICD10CM	C78.2	Secondary malignant neoplasm of pleura	Cancer
519240	ICD10CM	C78.30	Secondary malignant neoplasm of unspecified respiratory organ	Cancer
519241	ICD10CM	C78.39	Secondary malignant neoplasm of other respiratory organs	Cancer
519242	ICD10CM	C78.4	Secondary malignant neoplasm of small intestine	Cancer
519243	ICD10CM	C78.5	Secondary malignant neoplasm of large intestine and rectum	Cancer
519244	ICD10CM	C78.6	Secondary malignant neoplasm of retroperitoneum and peritoneum	Cancer
519245	ICD10CM	C78.7	Secondary malignant neoplasm of liver and intrahepatic bile duct	Cancer
519247	ICD10CM	C78.80	Secondary malignant neoplasm of unspecified digestive organ	Cancer
519248	ICD10CM	C78.89	Secondary malignant neoplasm of other digestive organs	Cancer
519251	ICD10CM	C79.00	Secondary malignant neoplasm of unspecified kidney and renal pelvis	Cancer
519252	ICD10CM	C79.01	Secondary malignant neoplasm of right kidney and renal pelvis	Cancer

519253	ICD10CM	C79.02	Secondary malignant neoplasm of left kidney and renal pelvis	Cancer
519255	ICD10CM	C79.10	Secondary malignant neoplasm of unspecified urinary organs	Cancer
519256	ICD10CM	C79.11	Secondary malignant neoplasm of bladder	Cancer
519257	ICD10CM	C79.19	Secondary malignant neoplasm of other urinary organs	Cancer
519258	ICD10CM	C79.2	Secondary malignant neoplasm of skin	Cancer
519260	ICD10CM	C79.31	Secondary malignant neoplasm of brain	Cancer
519261	ICD10CM	C79.32	Secondary malignant neoplasm of cerebral meninges	Cancer
519263	ICD10CM	C79.40	Secondary malignant neoplasm of unspecified part of nervous system	Cancer
519264	ICD10CM	C79.49	Secondary malignant neoplasm of other parts of nervous system	Cancer
519266	ICD10CM	C79.51	Secondary malignant neoplasm of bone	Cancer
519267	ICD10CM	C79.52	Secondary malignant neoplasm of bone marrow	Cancer
519269	ICD10CM	C79.60	Secondary malignant neoplasm of unspecified ovary	Cancer
519270	ICD10CM	C79.61	Secondary malignant neoplasm of right ovary	Cancer
519271	ICD10CM	C79.62	Secondary malignant neoplasm of left ovary	Cancer
519273	ICD10CM	C79.70	Secondary malignant neoplasm of unspecified adrenal gland	Cancer
519274	ICD10CM	C79.71	Secondary malignant neoplasm of right adrenal gland	Cancer
519275	ICD10CM	C79.72	Secondary malignant neoplasm of left adrenal gland	Cancer
519277	ICD10CM	C79.81	Secondary malignant neoplasm of breast	Cancer
519278	ICD10CM	C79.82	Secondary malignant neoplasm of genital organs	Cancer
519279	ICD10CM	C79.89	Secondary malignant neoplasm of other specified sites	Cancer
519280	ICD10CM	C79.9	Secondary malignant neoplasm of unspecified site	Cancer
519283	ICD10CM	C7A.00	Malignant carcinoid tumor of unspecified site	Cancer
519285	ICD10CM	C7A.010	Malignant carcinoid tumor of the duodenum	Cancer
519286	ICD10CM	C7A.011	Malignant carcinoid tumor of the jejunum	Cancer

519287	ICD10CM	C7A.012	Malignant carcinoid tumor of the ileum	Cancer
519288	ICD10CM	C7A.019	Malignant carcinoid tumor of the small intestine, unspecified portion	Cancer
519290	ICD10CM	C7A.020	Malignant carcinoid tumor of the appendix	Cancer
519297	ICD10CM	C7A.029	Malignant carcinoid tumor of the large intestine, unspecified portion	Cancer
519299	ICD10CM	C7A.090	Malignant carcinoid tumor of the bronchus and lung	Cancer
519300	ICD10CM	C7A.091	Malignant carcinoid tumor of the thymus	Cancer
519301	ICD10CM	C7A.092	Malignant carcinoid tumor of the stomach	Cancer
519302	ICD10CM	C7A.093	Malignant carcinoid tumor of the kidney	Cancer
519303	ICD10CM	C7A.094	Malignant carcinoid tumor of the foregut, unspecified	Cancer
519304	ICD10CM	C7A.095	Malignant carcinoid tumor of the midgut, unspecified	Cancer
519305	ICD10CM	C7A.096	Malignant carcinoid tumor of the hindgut, unspecified	Cancer
519306	ICD10CM	C7A.098	Malignant carcinoid tumors of other sites	Cancer
519307	ICD10CM	C7A.1	Malignant poorly differentiated neuroendocrine tumors	Cancer
519308	ICD10CM	C7A.8	Other malignant neuroendocrine tumors	Cancer
519311	ICD10CM	C7B.00	Secondary carcinoid tumors, unspecified site	Cancer
519314	ICD10CM	C7B.03	Secondary carcinoid tumors of bone	Cancer
519315	ICD10CM	C7B.04	Secondary carcinoid tumors of peritoneum	Cancer
519316	ICD10CM	C7B.09	Secondary carcinoid tumors of other sites	Cancer
519317	ICD10CM	C7B.1	Secondary Merkel cell carcinoma	Cancer
519318	ICD10CM	C7B.8	Other secondary neuroendocrine tumors	Cancer
519320	ICD10CM	C80.0	Disseminated malignant neoplasm, unspecified	Cancer
519321	ICD10CM	C80.1	Malignant (primary) neoplasm, unspecified	Cancer
519322	ICD10CM	C80.2	Malignant neoplasm associated with transplanted organ	Cancer
519325	ICD10CM	C81.00	Nodular lymphocyte predominant Hodgkin lymphoma, unspecified site	Cancer

519326	ICD10CM	C81.01	Nodular lymphocyte predominant Hodgkin lymphoma, lymph nodes of head, face, and neck	Cancer
519327	ICD10CM	C81.02	Nodular lymphocyte predominant Hodgkin lymphoma, intrathoracic lymph nodes	Cancer
519328	ICD10CM	C81.03	Nodular lymphocyte predominant Hodgkin lymphoma, intra-abdominal lymph nodes	Cancer
519329	ICD10CM	C81.04	Nodular lymphocyte predominant Hodgkin lymphoma, lymph nodes of axilla and upper limb	Cancer
519330	ICD10CM	C81.05	Nodular lymphocyte predominant Hodgkin lymphoma, lymph nodes of inguinal region and lower limb	Cancer
519331	ICD10CM	C81.06	Nodular lymphocyte predominant Hodgkin lymphoma, intrapelvic lymph nodes	Cancer
519332	ICD10CM	C81.07	Nodular lymphocyte predominant Hodgkin lymphoma, spleen	Cancer
519333	ICD10CM	C81.08	Nodular lymphocyte predominant Hodgkin lymphoma, lymph nodes of multiple sites	Cancer
519334	ICD10CM	C81.09	Nodular lymphocyte predominant Hodgkin lymphoma, extranodal and solid organ sites	Cancer
519336	ICD10CM	C81.10	Nodular sclerosis Hodgkin lymphoma, unspecified site	Cancer
519337	ICD10CM	C81.11	Nodular sclerosis Hodgkin lymphoma, lymph nodes of head, face, and neck	Cancer
519338	ICD10CM	C81.12	Nodular sclerosis Hodgkin lymphoma, intrathoracic lymph nodes	Cancer
519339	ICD10CM	C81.13	Nodular sclerosis Hodgkin lymphoma, intra-abdominal lymph nodes	Cancer
519340	ICD10CM	C81.14	Nodular sclerosis Hodgkin lymphoma, lymph nodes of axilla and upper limb	Cancer
519341	ICD10CM	C81.15	Nodular sclerosis Hodgkin lymphoma, lymph nodes of inguinal region and lower limb	Cancer

519342	ICD10CM	C81.16	Nodular sclerosis Hodgkin lymphoma, intrapelvic lymph nodes	Cancer
519343	ICD10CM	C81.17	Nodular sclerosis Hodgkin lymphoma, spleen	Cancer
519344	ICD10CM	C81.18	Nodular sclerosis Hodgkin lymphoma, lymph nodes of multiple sites	Cancer
519345	ICD10CM	C81.19	Nodular sclerosis Hodgkin lymphoma, extranodal and solid organ sites	Cancer
519347	ICD10CM	C81.20	Mixed cellularity Hodgkin lymphoma, unspecified site	Cancer
519348	ICD10CM	C81.21	Mixed cellularity Hodgkin lymphoma, lymph nodes of head, face, and neck	Cancer
519349	ICD10CM	C81.22	Mixed cellularity Hodgkin lymphoma, intrathoracic lymph nodes	Cancer
519350	ICD10CM	C81.23	Mixed cellularity Hodgkin lymphoma, intra-abdominal lymph nodes	Cancer
519351	ICD10CM	C81.24	Mixed cellularity Hodgkin lymphoma, lymph nodes of axilla and upper limb	Cancer
519352	ICD10CM	C81.25	Mixed cellularity Hodgkin lymphoma, lymph nodes of inguinal region and lower limb	Cancer
519353	ICD10CM	C81.26	Mixed cellularity Hodgkin lymphoma, intrapelvic lymph nodes	Cancer
519354	ICD10CM	C81.27	Mixed cellularity Hodgkin lymphoma, spleen	Cancer
519355	ICD10CM	C81.28	Mixed cellularity Hodgkin lymphoma, lymph nodes of multiple sites	Cancer
519356	ICD10CM	C81.29	Mixed cellularity Hodgkin lymphoma, extranodal and solid organ sites	Cancer
519358	ICD10CM	C81.30	Lymphocyte depleted Hodgkin lymphoma, unspecified site	Cancer
519359	ICD10CM	C81.31	Lymphocyte depleted Hodgkin lymphoma, lymph nodes of head, face, and neck	Cancer
519360	ICD10CM	C81.32	Lymphocyte depleted Hodgkin lymphoma, intrathoracic lymph nodes	Cancer
519361	ICD10CM	C81.33	Lymphocyte depleted Hodgkin lymphoma, intra-abdominal lymph nodes	Cancer

519362	ICD10CM	C81.34	Lymphocyte depleted Hodgkin lymphoma, lymph nodes of axilla and upper limb	Cancer
519363	ICD10CM	C81.35	Lymphocyte depleted Hodgkin lymphoma, lymph nodes of inguinal region and lower limb	Cancer
519364	ICD10CM	C81.36	Lymphocyte depleted Hodgkin lymphoma, intrapelvic lymph nodes	Cancer
519365	ICD10CM	C81.37	Lymphocyte depleted Hodgkin lymphoma, spleen	Cancer
519366	ICD10CM	C81.38	Lymphocyte depleted Hodgkin lymphoma, lymph nodes of multiple sites	Cancer
519367	ICD10CM	C81.39	Lymphocyte depleted Hodgkin lymphoma, extranodal and solid organ sites	Cancer
519369	ICD10CM	C81.40	Lymphocyte-rich Hodgkin lymphoma, unspecified site	Cancer
519370	ICD10CM	C81.41	Lymphocyte-rich Hodgkin lymphoma, lymph nodes of head, face, and neck	Cancer
519371	ICD10CM	C81.42	Lymphocyte-rich Hodgkin lymphoma, intrathoracic lymph nodes	Cancer
519372	ICD10CM	C81.43	Lymphocyte-rich Hodgkin lymphoma, intra-abdominal lymph nodes	Cancer
519373	ICD10CM	C81.44	Lymphocyte-rich Hodgkin lymphoma, lymph nodes of axilla and upper limb	Cancer
519374	ICD10CM	C81.45	Lymphocyte-rich Hodgkin lymphoma, lymph nodes of inguinal region and lower limb	Cancer
519375	ICD10CM	C81.46	Lymphocyte-rich Hodgkin lymphoma, intrapelvic lymph nodes	Cancer
519376	ICD10CM	C81.47	Lymphocyte-rich Hodgkin lymphoma, spleen	Cancer
519377	ICD10CM	C81.48	Lymphocyte-rich Hodgkin lymphoma, lymph nodes of multiple sites	Cancer
519378	ICD10CM	C81.49	Lymphocyte-rich Hodgkin lymphoma, extranodal and solid organ sites	Cancer
519380	ICD10CM	C81.70	Other Hodgkin lymphoma, unspecified site	Cancer
519381	ICD10CM	C81.71	Other Hodgkin lymphoma, lymph nodes of head, face, and neck	Cancer

519382	ICD10CM	C81.72	Other Hodgkin lymphoma, intrathoracic lymph nodes	Cancer
519383	ICD10CM	C81.73	Other Hodgkin lymphoma, intra-abdominal lymph nodes	Cancer
519384	ICD10CM	C81.74	Other Hodgkin lymphoma, lymph nodes of axilla and upper limb	Cancer
519385	ICD10CM	C81.75	Other Hodgkin lymphoma, lymph nodes of inguinal region and lower limb	Cancer
519386	ICD10CM	C81.76	Other Hodgkin lymphoma, intrapelvic lymph nodes	Cancer
519387	ICD10CM	C81.77	Other Hodgkin lymphoma, spleen	Cancer
519388	ICD10CM	C81.78	Other Hodgkin lymphoma, lymph nodes of multiple sites	Cancer
519389	ICD10CM	C81.79	Other Hodgkin lymphoma, extranodal and solid organ sites	Cancer
519391	ICD10CM	C81.90	Hodgkin lymphoma, unspecified, unspecified site	Cancer
519392	ICD10CM	C81.91	Hodgkin lymphoma, unspecified, lymph nodes of head, face, and neck	Cancer
519393	ICD10CM	C81.92	Hodgkin lymphoma, unspecified, intrathoracic lymph nodes	Cancer
519394	ICD10CM	C81.93	Hodgkin lymphoma, unspecified, intra-abdominal lymph nodes	Cancer
519395	ICD10CM	C81.94	Hodgkin lymphoma, unspecified, lymph nodes of axilla and upper limb	Cancer
519396	ICD10CM	C81.95	Hodgkin lymphoma, unspecified, lymph nodes of inguinal region and lower limb	Cancer
519397	ICD10CM	C81.96	Hodgkin lymphoma, unspecified, intrapelvic lymph nodes	Cancer
519398	ICD10CM	C81.97	Hodgkin lymphoma, unspecified, spleen	Cancer
519399	ICD10CM	C81.98	Hodgkin lymphoma, unspecified, lymph nodes of multiple sites	Cancer
519400	ICD10CM	C81.99	Hodgkin lymphoma, unspecified, extranodal and solid organ sites	Cancer
519403	ICD10CM	C82.00	Follicular lymphoma grade I, unspecified site	Cancer
519404	ICD10CM	C82.01	Follicular lymphoma grade I, lymph nodes of head, face, and neck	Cancer
519405	ICD10CM	C82.02	Follicular lymphoma grade I, intrathoracic lymph nodes	Cancer
519406	ICD10CM	C82.03	Follicular lymphoma grade I, intra-abdominal lymph nodes	Cancer

519407	ICD10CM	C82.04	Follicular lymphoma grade I, lymph nodes of axilla and upper limb	Cancer
519408	ICD10CM	C82.05	Follicular lymphoma grade I, lymph nodes of inguinal region and lower limb	Cancer
519409	ICD10CM	C82.06	Follicular lymphoma grade I, intrapelvic lymph nodes	Cancer
519410	ICD10CM	C82.07	Follicular lymphoma grade I, spleen	Cancer
519411	ICD10CM	C82.08	Follicular lymphoma grade I, lymph nodes of multiple sites	Cancer
519412	ICD10CM	C82.09	Follicular lymphoma grade I, extranodal and solid organ sites	Cancer
519414	ICD10CM	C82.10	Follicular lymphoma grade II, unspecified site	Cancer
519415	ICD10CM	C82.11	Follicular lymphoma grade II, lymph nodes of head, face, and neck	Cancer
519416	ICD10CM	C82.12	Follicular lymphoma grade II, intrathoracic lymph nodes	Cancer
519417	ICD10CM	C82.13	Follicular lymphoma grade II, intra-abdominal lymph nodes	Cancer
519418	ICD10CM	C82.14	Follicular lymphoma grade II, lymph nodes of axilla and upper limb	Cancer
519419	ICD10CM	C82.15	Follicular lymphoma grade II, lymph nodes of inguinal region and lower limb	Cancer
519420	ICD10CM	C82.16	Follicular lymphoma grade II, intrapelvic lymph nodes	Cancer
519421	ICD10CM	C82.17	Follicular lymphoma grade II, spleen	Cancer
519422	ICD10CM	C82.18	Follicular lymphoma grade II, lymph nodes of multiple sites	Cancer
519423	ICD10CM	C82.19	Follicular lymphoma grade II, extranodal and solid organ sites	Cancer
519425	ICD10CM	C82.20	Follicular lymphoma grade III, unspecified, unspecified site	Cancer
519426	ICD10CM	C82.21	Follicular lymphoma grade III, unspecified, lymph nodes of head, face, and neck	Cancer
519427	ICD10CM	C82.22	Follicular lymphoma grade III, unspecified, intrathoracic lymph nodes	Cancer
519428	ICD10CM	C82.23	Follicular lymphoma grade III, unspecified, intra-abdominal lymph nodes	Cancer
519429	ICD10CM	C82.24	Follicular lymphoma grade III, unspecified, lymph nodes of axilla and upper limb	Cancer

519430	ICD10CM	C82.25	Follicular lymphoma grade III, unspecified, lymph nodes of inguinal region and lower limb	Cancer
519431	ICD10CM	C82.26	Follicular lymphoma grade III, unspecified, intrapelvic lymph nodes	Cancer
519432	ICD10CM	C82.27	Follicular lymphoma grade III, unspecified, spleen	Cancer
519433	ICD10CM	C82.28	Follicular lymphoma grade III, unspecified, lymph nodes of multiple sites	Cancer
519434	ICD10CM	C82.29	Follicular lymphoma grade III, unspecified, extranodal and solid organ sites	Cancer
519436	ICD10CM	C82.30	Follicular lymphoma grade IIIa, unspecified site	Cancer
519437	ICD10CM	C82.31	Follicular lymphoma grade IIIa, lymph nodes of head, face, and neck	Cancer
519438	ICD10CM	C82.32	Follicular lymphoma grade IIIa, intrathoracic lymph nodes	Cancer
519439	ICD10CM	C82.33	Follicular lymphoma grade IIIa, intra-abdominal lymph nodes	Cancer
519440	ICD10CM	C82.34	Follicular lymphoma grade IIIa, lymph nodes of axilla and upper limb	Cancer
519441	ICD10CM	C82.35	Follicular lymphoma grade IIIa, lymph nodes of inguinal region and lower limb	Cancer
519442	ICD10CM	C82.36	Follicular lymphoma grade IIIa, intrapelvic lymph nodes	Cancer
519443	ICD10CM	C82.37	Follicular lymphoma grade IIIa, spleen	Cancer
519444	ICD10CM	C82.38	Follicular lymphoma grade IIIa, lymph nodes of multiple sites	Cancer
519445	ICD10CM	C82.39	Follicular lymphoma grade IIIa, extranodal and solid organ sites	Cancer
519447	ICD10CM	C82.40	Follicular lymphoma grade IIIb, unspecified site	Cancer
519448	ICD10CM	C82.41	Follicular lymphoma grade IIIb, lymph nodes of head, face, and neck	Cancer
519449	ICD10CM	C82.42	Follicular lymphoma grade IIIb, intrathoracic lymph nodes	Cancer
519450	ICD10CM	C82.43	Follicular lymphoma grade IIIb, intra-abdominal lymph nodes	Cancer
519451	ICD10CM	C82.44	Follicular lymphoma grade IIIb, lymph nodes of axilla and upper limb	Cancer

519452	ICD10CM	C82.45	Follicular lymphoma grade IIIb, lymph nodes of inguinal region and lower limb	Cancer
519453	ICD10CM	C82.46	Follicular lymphoma grade IIIb, intrapelvic lymph nodes	Cancer
519454	ICD10CM	C82.47	Follicular lymphoma grade IIIb, spleen	Cancer
519455	ICD10CM	C82.48	Follicular lymphoma grade IIIb, lymph nodes of multiple sites	Cancer
519456	ICD10CM	C82.49	Follicular lymphoma grade IIIb, extranodal and solid organ sites	Cancer
519458	ICD10CM	C82.50	Diffuse follicle center lymphoma, unspecified site	Cancer
519459	ICD10CM	C82.51	Diffuse follicle center lymphoma, lymph nodes of head, face, and neck	Cancer
519460	ICD10CM	C82.52	Diffuse follicle center lymphoma, intrathoracic lymph nodes	Cancer
519461	ICD10CM	C82.53	Diffuse follicle center lymphoma, intra-abdominal lymph nodes	Cancer
519462	ICD10CM	C82.54	Diffuse follicle center lymphoma, lymph nodes of axilla and upper limb	Cancer
519463	ICD10CM	C82.55	Diffuse follicle center lymphoma, lymph nodes of inguinal region and lower limb	Cancer
519464	ICD10CM	C82.56	Diffuse follicle center lymphoma, intrapelvic lymph nodes	Cancer
519465	ICD10CM	C82.57	Diffuse follicle center lymphoma, spleen	Cancer
519466	ICD10CM	C82.58	Diffuse follicle center lymphoma, lymph nodes of multiple sites	Cancer
519467	ICD10CM	C82.59	Diffuse follicle center lymphoma, extranodal and solid organ sites	Cancer
519469	ICD10CM	C82.60	Cutaneous follicle center lymphoma, unspecified site	Cancer
519470	ICD10CM	C82.61	Cutaneous follicle center lymphoma, lymph nodes of head, face, and neck	Cancer
519471	ICD10CM	C82.62	Cutaneous follicle center lymphoma, intrathoracic lymph nodes	Cancer
519472	ICD10CM	C82.63	Cutaneous follicle center lymphoma, intra-abdominal lymph nodes	Cancer
519473	ICD10CM	C82.64	Cutaneous follicle center lymphoma, lymph nodes of axilla and upper limb	Cancer
519474	ICD10CM	C82.65	Cutaneous follicle center lymphoma, lymph nodes of inguinal region and lower limb	Cancer

519475	ICD10CM	C82.66	Cutaneous follicle center lymphoma, intrapelvic lymph nodes	Cancer
519476	ICD10CM	C82.67	Cutaneous follicle center lymphoma, spleen	Cancer
519477	ICD10CM	C82.68	Cutaneous follicle center lymphoma, lymph nodes of multiple sites	Cancer
519478	ICD10CM	C82.69	Cutaneous follicle center lymphoma, extranodal and solid organ sites	Cancer
519490	ICD10CM	C82.80	Other types of follicular lymphoma, unspecified site	Cancer
519491	ICD10CM	C82.81	Other types of follicular lymphoma, lymph nodes of head, face, and neck	Cancer
519492	ICD10CM	C82.82	Other types of follicular lymphoma, intrathoracic lymph nodes	Cancer
519493	ICD10CM	C82.83	Other types of follicular lymphoma, intra-abdominal lymph nodes	Cancer
519494	ICD10CM	C82.84	Other types of follicular lymphoma, lymph nodes of axilla and upper limb	Cancer
519495	ICD10CM	C82.85	Other types of follicular lymphoma, lymph nodes of inguinal region and lower limb	Cancer
519496	ICD10CM	C82.86	Other types of follicular lymphoma, intrapelvic lymph nodes	Cancer
519497	ICD10CM	C82.87	Other types of follicular lymphoma, spleen	Cancer
519498	ICD10CM	C82.88	Other types of follicular lymphoma, lymph nodes of multiple sites	Cancer
519499	ICD10CM	C82.89	Other types of follicular lymphoma, extranodal and solid organ sites	Cancer
519501	ICD10CM	C82.90	Follicular lymphoma, unspecified, unspecified site	Cancer
519502	ICD10CM	C82.91	Follicular lymphoma, unspecified, lymph nodes of head, face, and neck	Cancer
519503	ICD10CM	C82.92	Follicular lymphoma, unspecified, intrathoracic lymph nodes	Cancer
519504	ICD10CM	C82.93	Follicular lymphoma, unspecified, intra-abdominal lymph nodes	Cancer

519505	ICD10CM	C82.94	Follicular lymphoma, unspecified, lymph nodes of axilla and upper limb	Cancer
519506	ICD10CM	C82.95	Follicular lymphoma, unspecified, lymph nodes of inguinal region and lower limb	Cancer
519507	ICD10CM	C82.96	Follicular lymphoma, unspecified, intrapelvic lymph nodes	Cancer
519508	ICD10CM	C82.97	Follicular lymphoma, unspecified, spleen	Cancer
519509	ICD10CM	C82.98	Follicular lymphoma, unspecified, lymph nodes of multiple sites	Cancer
519510	ICD10CM	C82.99	Follicular lymphoma, unspecified, extranodal and solid organ sites	Cancer
519513	ICD10CM	C83.00	Small cell B-cell lymphoma, unspecified site	Cancer
519514	ICD10CM	C83.01	Small cell B-cell lymphoma, lymph nodes of head, face, and neck	Cancer
519515	ICD10CM	C83.02	Small cell B-cell lymphoma, intrathoracic lymph nodes	Cancer
519516	ICD10CM	C83.03	Small cell B-cell lymphoma, intra-abdominal lymph nodes	Cancer
519517	ICD10CM	C83.04	Small cell B-cell lymphoma, lymph nodes of axilla and upper limb	Cancer
519518	ICD10CM	C83.05	Small cell B-cell lymphoma, lymph nodes of inguinal region and lower limb	Cancer
519519	ICD10CM	C83.06	Small cell B-cell lymphoma, intrapelvic lymph nodes	Cancer
519520	ICD10CM	C83.07	Small cell B-cell lymphoma, spleen	Cancer
519521	ICD10CM	C83.08	Small cell B-cell lymphoma, lymph nodes of multiple sites	Cancer
519522	ICD10CM	C83.09	Small cell B-cell lymphoma, extranodal and solid organ sites	Cancer
519524	ICD10CM	C83.10	Mantle cell lymphoma, unspecified site	Cancer
519525	ICD10CM	C83.11	Mantle cell lymphoma, lymph nodes of head, face, and neck	Cancer
519526	ICD10CM	C83.12	Mantle cell lymphoma, intrathoracic lymph nodes	Cancer
519527	ICD10CM	C83.13	Mantle cell lymphoma, intra-abdominal lymph nodes	Cancer
519528	ICD10CM	C83.14	Mantle cell lymphoma, lymph nodes of axilla and upper limb	Cancer

519529	ICD10CM	C83.15	Mantle cell lymphoma, lymph nodes of inguinal region and lower limb	Cancer
519530	ICD10CM	C83.16	Mantle cell lymphoma, intrapelvic lymph nodes	Cancer
519531	ICD10CM	C83.17	Mantle cell lymphoma, spleen	Cancer
519532	ICD10CM	C83.18	Mantle cell lymphoma, lymph nodes of multiple sites	Cancer
519533	ICD10CM	C83.19	Mantle cell lymphoma, extranodal and solid organ sites	Cancer
519545	ICD10CM	C83.30	Diffuse large B-cell lymphoma, unspecified site	Cancer
519546	ICD10CM	C83.31	Diffuse large B-cell lymphoma, lymph nodes of head, face, and neck	Cancer
519547	ICD10CM	C83.32	Diffuse large B-cell lymphoma, intrathoracic lymph nodes	Cancer
519548	ICD10CM	C83.33	Diffuse large B-cell lymphoma, intra-abdominal lymph nodes	Cancer
519549	ICD10CM	C83.34	Diffuse large B-cell lymphoma, lymph nodes of axilla and upper limb	Cancer
519550	ICD10CM	C83.35	Diffuse large B-cell lymphoma, lymph nodes of inguinal region and lower limb	Cancer
519551	ICD10CM	C83.36	Diffuse large B-cell lymphoma, intrapelvic lymph nodes	Cancer
519552	ICD10CM	C83.37	Diffuse large B-cell lymphoma, spleen	Cancer
519553	ICD10CM	C83.38	Diffuse large B-cell lymphoma, lymph nodes of multiple sites	Cancer
519554	ICD10CM	C83.39	Diffuse large B-cell lymphoma, extranodal and solid organ sites	Cancer
519566	ICD10CM	C83.50	Lymphoblastic (diffuse) lymphoma, unspecified site	Cancer
519567	ICD10CM	C83.51	Lymphoblastic (diffuse) lymphoma, lymph nodes of head, face, and neck	Cancer
519568	ICD10CM	C83.52	Lymphoblastic (diffuse) lymphoma, intrathoracic lymph nodes	Cancer
519569	ICD10CM	C83.53	Lymphoblastic (diffuse) lymphoma, intra-abdominal lymph nodes	Cancer
519570	ICD10CM	C83.54	Lymphoblastic (diffuse) lymphoma, lymph nodes of axilla and upper limb	Cancer
519571	ICD10CM	C83.55	Lymphoblastic (diffuse) lymphoma, lymph nodes of inguinal region and lower limb	Cancer

519572	ICD10CM	C83.56	Lymphoblastic (diffuse) lymphoma, intrapelvic lymph nodes	Cancer
519573	ICD10CM	C83.57	Lymphoblastic (diffuse) lymphoma, spleen	Cancer
519574	ICD10CM	C83.58	Lymphoblastic (diffuse) lymphoma, lymph nodes of multiple sites	Cancer
519575	ICD10CM	C83.59	Lymphoblastic (diffuse) lymphoma, extranodal and solid organ sites	Cancer
519587	ICD10CM	C83.70	Burkitt lymphoma, unspecified site	Cancer
519588	ICD10CM	C83.71	Burkitt lymphoma, lymph nodes of head, face, and neck	Cancer
519589	ICD10CM	C83.72	Burkitt lymphoma, intrathoracic lymph nodes	Cancer
519590	ICD10CM	C83.73	Burkitt lymphoma, intra-abdominal lymph nodes	Cancer
519591	ICD10CM	C83.74	Burkitt lymphoma, lymph nodes of axilla and upper limb	Cancer
519592	ICD10CM	C83.75	Burkitt lymphoma, lymph nodes of inguinal region and lower limb	Cancer
519593	ICD10CM	C83.76	Burkitt lymphoma, intrapelvic lymph nodes	Cancer
519594	ICD10CM	C83.77	Burkitt lymphoma, spleen	Cancer
519595	ICD10CM	C83.78	Burkitt lymphoma, lymph nodes of multiple sites	Cancer
519596	ICD10CM	C83.79	Burkitt lymphoma, extranodal and solid organ sites	Cancer
519598	ICD10CM	C83.80	Other non-follicular lymphoma, unspecified site	Cancer
519599	ICD10CM	C83.81	Other non-follicular lymphoma, lymph nodes of head, face, and neck	Cancer
519600	ICD10CM	C83.82	Other non-follicular lymphoma, intrathoracic lymph nodes	Cancer
519601	ICD10CM	C83.83	Other non-follicular lymphoma, intra-abdominal lymph nodes	Cancer
519602	ICD10CM	C83.84	Other non-follicular lymphoma, lymph nodes of axilla and upper limb	Cancer
519603	ICD10CM	C83.85	Other non-follicular lymphoma, lymph nodes of inguinal region and lower limb	Cancer
519604	ICD10CM	C83.86	Other non-follicular lymphoma, intrapelvic lymph nodes	Cancer
519605	ICD10CM	C83.87	Other non-follicular lymphoma, spleen	Cancer
519606	ICD10CM	C83.88	Other non-follicular lymphoma, lymph nodes of multiple sites	Cancer

519607	ICD10CM	C83.89	Other non-follicular lymphoma, extranodal and solid organ sites	Cancer
519609	ICD10CM	C83.90	Non-follicular (diffuse) lymphoma, unspecified, unspecified site	Cancer
519610	ICD10CM	C83.91	Non-follicular (diffuse) lymphoma, unspecified, lymph nodes of head, face, and neck	Cancer
519611	ICD10CM	C83.92	Non-follicular (diffuse) lymphoma, unspecified, intrathoracic lymph nodes	Cancer
519612	ICD10CM	C83.93	Non-follicular (diffuse) lymphoma, unspecified, intra-abdominal lymph nodes	Cancer
519613	ICD10CM	C83.94	Non-follicular (diffuse) lymphoma, unspecified, lymph nodes of axilla and upper limb	Cancer
519614	ICD10CM	C83.95	Non-follicular (diffuse) lymphoma, unspecified, lymph nodes of inguinal region and lower limb	Cancer
519615	ICD10CM	C83.96	Non-follicular (diffuse) lymphoma, unspecified, intrapelvic lymph nodes	Cancer
519616	ICD10CM	C83.97	Non-follicular (diffuse) lymphoma, unspecified, spleen	Cancer
519617	ICD10CM	C83.98	Non-follicular (diffuse) lymphoma, unspecified, lymph nodes of multiple sites	Cancer
519618	ICD10CM	C83.99	Non-follicular (diffuse) lymphoma, unspecified, extranodal and solid organ sites	Cancer
519621	ICD10CM	C84.00	Mycosis fungoides, unspecified site	Cancer
519622	ICD10CM	C84.01	Mycosis fungoides, lymph nodes of head, face, and neck	Cancer
519623	ICD10CM	C84.02	Mycosis fungoides, intrathoracic lymph nodes	Cancer
519624	ICD10CM	C84.03	Mycosis fungoides, intra-abdominal lymph nodes	Cancer
519625	ICD10CM	C84.04	Mycosis fungoides, lymph nodes of axilla and upper limb	Cancer
519626	ICD10CM	C84.05	Mycosis fungoides, lymph nodes of inguinal region and lower limb	Cancer
519627	ICD10CM	C84.06	Mycosis fungoides, intrapelvic lymph nodes	Cancer
519628	ICD10CM	C84.07	Mycosis fungoides, spleen	Cancer
519629	ICD10CM	C84.08	Mycosis fungoides, lymph nodes of multiple sites	Cancer

519630	ICD10CM	C84.09	Mycosis fungoides, extranodal and solid organ sites	Cancer
519632	ICD10CM	C84.10	Sezary disease, unspecified site	Cancer
519633	ICD10CM	C84.11	Sezary disease, lymph nodes of head, face, and neck	Cancer
519634	ICD10CM	C84.12	Sezary disease, intrathoracic lymph nodes	Cancer
519635	ICD10CM	C84.13	Sezary disease, intra-abdominal lymph nodes	Cancer
519636	ICD10CM	C84.14	Sezary disease, lymph nodes of axilla and upper limb	Cancer
519637	ICD10CM	C84.15	Sezary disease, lymph nodes of inguinal region and lower limb	Cancer
519638	ICD10CM	C84.16	Sezary disease, intrapelvic lymph nodes	Cancer
519639	ICD10CM	C84.17	Sezary disease, spleen	Cancer
519640	ICD10CM	C84.18	Sezary disease, lymph nodes of multiple sites	Cancer
519641	ICD10CM	C84.19	Sezary disease, extranodal and solid organ sites	Cancer
519663	ICD10CM	C84.40	Peripheral T-cell lymphoma, not classified, unspecified site	Cancer
519664	ICD10CM	C84.41	Peripheral T-cell lymphoma, not classified, lymph nodes of head, face, and neck	Cancer
519665	ICD10CM	C84.42	Peripheral T-cell lymphoma, not classified, intrathoracic lymph nodes	Cancer
519666	ICD10CM	C84.43	Peripheral T-cell lymphoma, not classified, intra-abdominal lymph nodes	Cancer
519667	ICD10CM	C84.44	Peripheral T-cell lymphoma, not classified, lymph nodes of axilla and upper limb	Cancer
519668	ICD10CM	C84.45	Peripheral T-cell lymphoma, not classified, lymph nodes of inguinal region and lower limb	Cancer
519669	ICD10CM	C84.46	Peripheral T-cell lymphoma, not classified, intrapelvic lymph nodes	Cancer
519670	ICD10CM	C84.47	Peripheral T-cell lymphoma, not classified, spleen	Cancer
519671	ICD10CM	C84.48	Peripheral T-cell lymphoma, not classified, lymph nodes of multiple sites	Cancer
519672	ICD10CM	C84.49	Peripheral T-cell lymphoma, not classified, extranodal and solid organ sites	Cancer
519684	ICD10CM	C84.60	Anaplastic large cell lymphoma, ALK-positive, unspecified site	Cancer

519685	ICD10CM	C84.61	Anaplastic large cell lymphoma, ALK-positive, lymph nodes of head, face, and neck	Cancer
519686	ICD10CM	C84.62	Anaplastic large cell lymphoma, ALK-positive, intrathoracic lymph nodes	Cancer
519687	ICD10CM	C84.63	Anaplastic large cell lymphoma, ALK-positive, intra-abdominal lymph nodes	Cancer
519688	ICD10CM	C84.64	Anaplastic large cell lymphoma, ALK-positive, lymph nodes of axilla and upper limb	Cancer
519689	ICD10CM	C84.65	Anaplastic large cell lymphoma, ALK-positive, lymph nodes of inguinal region and lower limb	Cancer
519690	ICD10CM	C84.66	Anaplastic large cell lymphoma, ALK-positive, intrapelvic lymph nodes	Cancer
519691	ICD10CM	C84.67	Anaplastic large cell lymphoma, ALK-positive, spleen	Cancer
519692	ICD10CM	C84.68	Anaplastic large cell lymphoma, ALK-positive, lymph nodes of multiple sites	Cancer
519693	ICD10CM	C84.69	Anaplastic large cell lymphoma, ALK-positive, extranodal and solid organ sites	Cancer
519695	ICD10CM	C84.70	Anaplastic large cell lymphoma, ALK-negative, unspecified site	Cancer
519696	ICD10CM	C84.71	Anaplastic large cell lymphoma, ALK-negative, lymph nodes of head, face, and neck	Cancer
519697	ICD10CM	C84.72	Anaplastic large cell lymphoma, ALK-negative, intrathoracic lymph nodes	Cancer
519698	ICD10CM	C84.73	Anaplastic large cell lymphoma, ALK-negative, intra-abdominal lymph nodes	Cancer
519699	ICD10CM	C84.74	Anaplastic large cell lymphoma, ALK-negative, lymph nodes of axilla and upper limb	Cancer
519700	ICD10CM	C84.75	Anaplastic large cell lymphoma, ALK-negative, lymph nodes of inguinal region and lower limb	Cancer
519701	ICD10CM	C84.76	Anaplastic large cell lymphoma, ALK-negative, intrapelvic lymph nodes	Cancer
519702	ICD10CM	C84.77	Anaplastic large cell lymphoma, ALK-negative, spleen	Cancer
519703	ICD10CM	C84.78	Anaplastic large cell lymphoma, ALK-negative, lymph nodes of multiple sites	Cancer

519704	ICD10CM	C84.79	Anaplastic large cell lymphoma, ALK-negative, extranodal and solid organ sites	Cancer
519706	ICD10CM	C84.90	Mature T/NK-cell lymphomas, unspecified, unspecified site	Cancer
519707	ICD10CM	C84.91	Mature T/NK-cell lymphomas, unspecified, lymph nodes of head, face, and neck	Cancer
519708	ICD10CM	C84.92	Mature T/NK-cell lymphomas, unspecified, intrathoracic lymph nodes	Cancer
519709	ICD10CM	C84.93	Mature T/NK-cell lymphomas, unspecified, intra-abdominal lymph nodes	Cancer
519710	ICD10CM	C84.94	Mature T/NK-cell lymphomas, unspecified, lymph nodes of axilla and upper limb	Cancer
519711	ICD10CM	C84.95	Mature T/NK-cell lymphomas, unspecified, lymph nodes of inguinal region and lower limb	Cancer
519712	ICD10CM	C84.96	Mature T/NK-cell lymphomas, unspecified, intrapelvic lymph nodes	Cancer
519713	ICD10CM	C84.97	Mature T/NK-cell lymphomas, unspecified, spleen	Cancer
519714	ICD10CM	C84.98	Mature T/NK-cell lymphomas, unspecified, lymph nodes of multiple sites	Cancer
519715	ICD10CM	C84.99	Mature T/NK-cell lymphomas, unspecified, extranodal and solid organ sites	Cancer
519717	ICD10CM	C84.A0	Cutaneous T-cell lymphoma, unspecified, unspecified site	Cancer
519718	ICD10CM	C84.A1	Cutaneous T-cell lymphoma, unspecified lymph nodes of head, face, and neck	Cancer
519719	ICD10CM	C84.A2	Cutaneous T-cell lymphoma, unspecified, intrathoracic lymph nodes	Cancer
519720	ICD10CM	C84.A3	Cutaneous T-cell lymphoma, unspecified, intra-abdominal lymph nodes	Cancer
519721	ICD10CM	C84.A4	Cutaneous T-cell lymphoma, unspecified, lymph nodes of axilla and upper limb	Cancer
519722	ICD10CM	C84.A5	Cutaneous T-cell lymphoma, unspecified, lymph nodes of inguinal region and lower limb	Cancer
519723	ICD10CM	C84.A6	Cutaneous T-cell lymphoma, unspecified, intrapelvic lymph nodes	Cancer

519724	ICD10CM	C84.A7	Cutaneous T-cell lymphoma, unspecified, spleen	Cancer
519725	ICD10CM	C84.A8	Cutaneous T-cell lymphoma, unspecified, lymph nodes of multiple sites	Cancer
519726	ICD10CM	C84.A9	Cutaneous T-cell lymphoma, unspecified, extranodal and solid organ sites	Cancer
519728	ICD10CM	C84.Z0	Other mature T/NK-cell lymphomas, unspecified site	Cancer
519729	ICD10CM	C84.Z1	Other mature T/NK-cell lymphomas, lymph nodes of head, face, and neck	Cancer
519730	ICD10CM	C84.Z2	Other mature T/NK-cell lymphomas, intrathoracic lymph nodes	Cancer
519731	ICD10CM	C84.Z3	Other mature T/NK-cell lymphomas, intra-abdominal lymph nodes	Cancer
519732	ICD10CM	C84.Z4	Other mature T/NK-cell lymphomas, lymph nodes of axilla and upper limb	Cancer
519733	ICD10CM	C84.Z5	Other mature T/NK-cell lymphomas, lymph nodes of inguinal region and lower limb	Cancer
519734	ICD10CM	C84.Z6	Other mature T/NK-cell lymphomas, intrapelvic lymph nodes	Cancer
519735	ICD10CM	C84.Z7	Other mature T/NK-cell lymphomas, spleen	Cancer
519736	ICD10CM	C84.Z8	Other mature T/NK-cell lymphomas, lymph nodes of multiple sites	Cancer
519737	ICD10CM	C84.Z9	Other mature T/NK-cell lymphomas, extranodal and solid organ sites	Cancer
519750	ICD10CM	C85.10	Unspecified B-cell lymphoma, unspecified site	Cancer
519751	ICD10CM	C85.11	Unspecified B-cell lymphoma, lymph nodes of head, face, and neck	Cancer
519752	ICD10CM	C85.12	Unspecified B-cell lymphoma, intrathoracic lymph nodes	Cancer
519753	ICD10CM	C85.13	Unspecified B-cell lymphoma, intra-abdominal lymph nodes	Cancer
519754	ICD10CM	C85.14	Unspecified B-cell lymphoma, lymph nodes of axilla and upper limb	Cancer
519755	ICD10CM	C85.15	Unspecified B-cell lymphoma, lymph nodes of inguinal region and lower limb	Cancer

519756	ICD10CM	C85.16	Unspecified B-cell lymphoma, intrapelvic lymph nodes	Cancer
519757	ICD10CM	C85.17	Unspecified B-cell lymphoma, spleen	Cancer
519758	ICD10CM	C85.18	Unspecified B-cell lymphoma, lymph nodes of multiple sites	Cancer
519759	ICD10CM	C85.19	Unspecified B-cell lymphoma, extranodal and solid organ sites	Cancer
519761	ICD10CM	C85.20	Mediastinal (thymic) large B-cell lymphoma, unspecified site	Cancer
519762	ICD10CM	C85.21	Mediastinal (thymic) large B-cell lymphoma, lymph nodes of head, face, and neck	Cancer
519763	ICD10CM	C85.22	Mediastinal (thymic) large B-cell lymphoma, intrathoracic lymph nodes	Cancer
519764	ICD10CM	C85.23	Mediastinal (thymic) large B-cell lymphoma, intra-abdominal lymph nodes	Cancer
519765	ICD10CM	C85.24	Mediastinal (thymic) large B-cell lymphoma, lymph nodes of axilla and upper limb	Cancer
519766	ICD10CM	C85.25	Mediastinal (thymic) large B-cell lymphoma, lymph nodes of inguinal region and lower limb	Cancer
519767	ICD10CM	C85.26	Mediastinal (thymic) large B-cell lymphoma, intrapelvic lymph nodes	Cancer
519768	ICD10CM	C85.27	Mediastinal (thymic) large B-cell lymphoma, spleen	Cancer
519769	ICD10CM	C85.28	Mediastinal (thymic) large B-cell lymphoma, lymph nodes of multiple sites	Cancer
519770	ICD10CM	C85.29	Mediastinal (thymic) large B-cell lymphoma, extranodal and solid organ sites	Cancer
519782	ICD10CM	C85.80	Other specified types of non-Hodgkin lymphoma, unspecified site	Cancer
519783	ICD10CM	C85.81	Other specified types of non-Hodgkin lymphoma, lymph nodes of head, face, and neck	Cancer
519784	ICD10CM	C85.82	Other specified types of non-Hodgkin lymphoma, intrathoracic lymph nodes	Cancer
519785	ICD10CM	C85.83	Other specified types of non-Hodgkin lymphoma, intra-abdominal lymph nodes	Cancer

519786	ICD10CM	C85.84	Other specified types of non-Hodgkin lymphoma, lymph nodes of axilla and upper limb	Cancer
519787	ICD10CM	C85.85	Other specified types of non-Hodgkin lymphoma, lymph nodes of inguinal region and lower limb	Cancer
519788	ICD10CM	C85.86	Other specified types of non-Hodgkin lymphoma, intrapelvic lymph nodes	Cancer
519789	ICD10CM	C85.87	Other specified types of non-Hodgkin lymphoma, spleen	Cancer
519790	ICD10CM	C85.88	Other specified types of non-Hodgkin lymphoma, lymph nodes of multiple sites	Cancer
519791	ICD10CM	C85.89	Other specified types of non-Hodgkin lymphoma, extranodal and solid organ sites	Cancer
519793	ICD10CM	C85.90	Non-Hodgkin lymphoma, unspecified, unspecified site	Cancer
519794	ICD10CM	C85.91	Non-Hodgkin lymphoma, unspecified, lymph nodes of head, face, and neck	Cancer
519795	ICD10CM	C85.92	Non-Hodgkin lymphoma, unspecified, intrathoracic lymph nodes	Cancer
519796	ICD10CM	C85.93	Non-Hodgkin lymphoma, unspecified, intra-abdominal lymph nodes	Cancer
519797	ICD10CM	C85.94	Non-Hodgkin lymphoma, unspecified, lymph nodes of axilla and upper limb	Cancer
519798	ICD10CM	C85.95	Non-Hodgkin lymphoma, unspecified, lymph nodes of inguinal region and lower limb	Cancer
519799	ICD10CM	C85.96	Non-Hodgkin lymphoma, unspecified, intrapelvic lymph nodes	Cancer
519800	ICD10CM	C85.97	Non-Hodgkin lymphoma, unspecified, spleen	Cancer
519801	ICD10CM	C85.98	Non-Hodgkin lymphoma, unspecified, lymph nodes of multiple sites	Cancer
519802	ICD10CM	C85.99	Non-Hodgkin lymphoma, unspecified, extranodal and solid organ sites	Cancer
519804	ICD10CM	C86.0	Extranodal NK/T-cell lymphoma, nasal type	Cancer
519805	ICD10CM	C86.1	Hepatosplenic T-cell lymphoma	Cancer
519806	ICD10CM	C86.2	Enteropathy-type (intestinal) T-cell lymphoma	Cancer

519807	ICD10CM	C86.3	Subcutaneous panniculitis-like T-cell lymphoma	Cancer
519808	ICD10CM	C86.4	Blastic NK-cell lymphoma	Cancer
519809	ICD10CM	C86.5	Angioimmunoblastic T-cell lymphoma	Cancer
519810	ICD10CM	C86.6	Primary cutaneous CD30-positive T-cell proliferations	Cancer
519814	ICD10CM	C88.2	Heavy chain disease	Cancer
519815	ICD10CM	C88.3	Immunoproliferative small intestinal disease	Cancer
519816	ICD10CM	C88.4	Extranodal marginal zone B-cell lymphoma of mucosa-associated lymphoid tissue [MALT-lymphoma]	Cancer
519818	ICD10CM	C88.8	Other malignant immunoproliferative diseases	Cancer
519819	ICD10CM	C88.9	Malignant immunoproliferative disease, unspecified	Cancer
519822	ICD10CM	C90.00	Multiple myeloma not having achieved remission	Cancer
519823	ICD10CM	C90.01	Multiple myeloma in remission	Cancer
519824	ICD10CM	C90.02	Multiple myeloma in relapse	Cancer
519826	ICD10CM	C90.10	Plasma cell leukemia not having achieved remission	Cancer
519827	ICD10CM	C90.11	Plasma cell leukemia in remission	Cancer
519828	ICD10CM	C90.12	Plasma cell leukemia in relapse	Cancer
519830	ICD10CM	C90.20	Extramedullary plasmacytoma not having achieved remission	Cancer
519831	ICD10CM	C90.21	Extramedullary plasmacytoma in remission	Cancer
519832	ICD10CM	C90.22	Extramedullary plasmacytoma in relapse	Cancer
519834	ICD10CM	C90.30	Solitary plasmacytoma not having achieved remission	Cancer
519835	ICD10CM	C90.31	Solitary plasmacytoma in remission	Cancer
519836	ICD10CM	C90.32	Solitary plasmacytoma in relapse	Cancer
519839	ICD10CM	C91.00	Acute lymphoblastic leukemia not having achieved remission	Cancer
519840	ICD10CM	C91.01	Acute lymphoblastic leukemia, in remission	Cancer
519841	ICD10CM	C91.02	Acute lymphoblastic leukemia, in relapse	Cancer
519843	ICD10CM	C91.10	Chronic lymphocytic leukemia of B-cell type not having achieved remission	Cancer
519844	ICD10CM	C91.11	Chronic lymphocytic leukemia of B-cell type in remission	Cancer

519845	ICD10CM	C91.12	Chronic lymphocytic leukemia of B-cell type in relapse	Cancer
519849	ICD10CM	C91.30	Prolymphocytic leukemia of B-cell type not having achieved remission	Cancer
519850	ICD10CM	C91.31	Prolymphocytic leukemia of B-cell type, in remission	Cancer
519851	ICD10CM	C91.32	Prolymphocytic leukemia of B-cell type, in relapse	Cancer
519853	ICD10CM	C91.40	Hairy cell leukemia not having achieved remission	Cancer
519854	ICD10CM	C91.41	Hairy cell leukemia, in remission	Cancer
519855	ICD10CM	C91.42	Hairy cell leukemia, in relapse	Cancer
519857	ICD10CM	C91.50	Adult T-cell lymphoma/leukemia (HTLV-1-associated) not having achieved remission	Cancer
519858	ICD10CM	C91.51	Adult T-cell lymphoma/leukemia (HTLV-1-associated), in remission	Cancer
519859	ICD10CM	C91.52	Adult T-cell lymphoma/leukemia (HTLV-1-associated), in relapse	Cancer
519861	ICD10CM	C91.60	Prolymphocytic leukemia of T-cell type not having achieved remission	Cancer
519862	ICD10CM	C91.61	Prolymphocytic leukemia of T-cell type, in remission	Cancer
519863	ICD10CM	C91.62	Prolymphocytic leukemia of T-cell type, in relapse	Cancer
519867	ICD10CM	C91.90	Lymphoid leukemia, unspecified not having achieved remission	Cancer
519868	ICD10CM	C91.91	Lymphoid leukemia, unspecified, in remission	Cancer
519869	ICD10CM	C91.92	Lymphoid leukemia, unspecified, in relapse	Cancer
519871	ICD10CM	C91.A0	Mature B-cell leukemia Burkitt-type not having achieved remission	Cancer
519872	ICD10CM	C91.A1	Mature B-cell leukemia Burkitt-type, in remission	Cancer
519873	ICD10CM	C91.A2	Mature B-cell leukemia Burkitt-type, in relapse	Cancer
519875	ICD10CM	C91.Z0	Other lymphoid leukemia not having achieved remission	Cancer
519876	ICD10CM	C91.Z1	Other lymphoid leukemia, in remission	Cancer
519877	ICD10CM	C91.Z2	Other lymphoid leukemia, in relapse	Cancer
519880	ICD10CM	C92.00	Acute myeloblastic leukemia, not having achieved remission	Cancer

519881	ICD10CM	C92.01	Acute myeloblastic leukemia, in remission	Cancer
519882	ICD10CM	C92.02	Acute myeloblastic leukemia, in relapse	Cancer
519884	ICD10CM	C92.10	Chronic myeloid leukemia, BCR/ABL-positive, not having achieved remission	Cancer
519885	ICD10CM	C92.11	Chronic myeloid leukemia, BCR/ABL-positive, in remission	Cancer
519886	ICD10CM	C92.12	Chronic myeloid leukemia, BCR/ABL-positive, in relapse	Cancer
519888	ICD10CM	C92.20	Atypical chronic myeloid leukemia, BCR/ABL-negative, not having achieved remission	Cancer
519889	ICD10CM	C92.21	Atypical chronic myeloid leukemia, BCR/ABL-negative, in remission	Cancer
519890	ICD10CM	C92.22	Atypical chronic myeloid leukemia, BCR/ABL-negative, in relapse	Cancer
519892	ICD10CM	C92.30	Myeloid sarcoma, not having achieved remission	Cancer
519893	ICD10CM	C92.31	Myeloid sarcoma, in remission	Cancer
519894	ICD10CM	C92.32	Myeloid sarcoma, in relapse	Cancer
519896	ICD10CM	C92.40	Acute promyelocytic leukemia, not having achieved remission	Cancer
519897	ICD10CM	C92.41	Acute promyelocytic leukemia, in remission	Cancer
519898	ICD10CM	C92.42	Acute promyelocytic leukemia, in relapse	Cancer
519900	ICD10CM	C92.50	Acute myelomonocytic leukemia, not having achieved remission	Cancer
519901	ICD10CM	C92.51	Acute myelomonocytic leukemia, in remission	Cancer
519902	ICD10CM	C92.52	Acute myelomonocytic leukemia, in relapse	Cancer
519904	ICD10CM	C92.60	Acute myeloid leukemia with 11q23-abnormality not having achieved remission	Cancer
519905	ICD10CM	C92.61	Acute myeloid leukemia with 11q23-abnormality in remission	Cancer
519906	ICD10CM	C92.62	Acute myeloid leukemia with 11q23-abnormality in relapse	Cancer
519910	ICD10CM	C92.90	Myeloid leukemia, unspecified, not having achieved remission	Cancer
519911	ICD10CM	C92.91	Myeloid leukemia, unspecified in remission	Cancer
519912	ICD10CM	C92.92	Myeloid leukemia, unspecified in relapse	Cancer

519914	ICD10CM	C92.A0	Acute myeloid leukemia with multilineage dysplasia, not having achieved remission	Cancer
519915	ICD10CM	C92.A1	Acute myeloid leukemia with multilineage dysplasia, in remission	Cancer
519916	ICD10CM	C92.A2	Acute myeloid leukemia with multilineage dysplasia, in relapse	Cancer
519918	ICD10CM	C92.Z0	Other myeloid leukemia not having achieved remission	Cancer
519919	ICD10CM	C92.Z1	Other myeloid leukemia, in remission	Cancer
519920	ICD10CM	C92.Z2	Other myeloid leukemia, in relapse	Cancer
519923	ICD10CM	C93.00	Acute monoblastic/monocytic leukemia, not having achieved remission	Cancer
519924	ICD10CM	C93.01	Acute monoblastic/monocytic leukemia, in remission	Cancer
519925	ICD10CM	C93.02	Acute monoblastic/monocytic leukemia, in relapse	Cancer
519927	ICD10CM	C93.10	Chronic myelomonocytic leukemia not having achieved remission	Cancer
519928	ICD10CM	C93.11	Chronic myelomonocytic leukemia, in remission	Cancer
519929	ICD10CM	C93.12	Chronic myelomonocytic leukemia, in relapse	Cancer
519933	ICD10CM	C93.30	Juvenile myelomonocytic leukemia, not having achieved remission	Cancer
519934	ICD10CM	C93.31	Juvenile myelomonocytic leukemia, in remission	Cancer
519935	ICD10CM	C93.32	Juvenile myelomonocytic leukemia, in relapse	Cancer
519939	ICD10CM	C93.90	Monocytic leukemia, unspecified, not having achieved remission	Cancer
519940	ICD10CM	C93.91	Monocytic leukemia, unspecified in remission	Cancer
519941	ICD10CM	C93.92	Monocytic leukemia, unspecified in relapse	Cancer
519943	ICD10CM	C93.Z0	Other monocytic leukemia, not having achieved remission	Cancer
519944	ICD10CM	C93.Z1	Other monocytic leukemia, in remission	Cancer
519945	ICD10CM	C93.Z2	Other monocytic leukemia, in relapse	Cancer
519948	ICD10CM	C94.00	Acute erythroid leukemia, not having achieved remission	Cancer

519949	ICD10CM	C94.01	Acute erythroid leukemia, in remission	Cancer
519950	ICD10CM	C94.02	Acute erythroid leukemia, in relapse	Cancer
519954	ICD10CM	C94.20	Acute megakaryoblastic leukemia not having achieved remission	Cancer
519955	ICD10CM	C94.21	Acute megakaryoblastic leukemia, in remission	Cancer
519956	ICD10CM	C94.22	Acute megakaryoblastic leukemia, in relapse	Cancer
519958	ICD10CM	C94.30	Mast cell leukemia not having achieved remission	Cancer
519959	ICD10CM	C94.31	Mast cell leukemia, in remission	Cancer
519960	ICD10CM	C94.32	Mast cell leukemia, in relapse	Cancer
519962	ICD10CM	C94.40	Acute panmyelosis with myelofibrosis not having achieved remission	Cancer
519963	ICD10CM	C94.41	Acute panmyelosis with myelofibrosis, in remission	Cancer
519964	ICD10CM	C94.42	Acute panmyelosis with myelofibrosis, in relapse	Cancer
519967	ICD10CM	C94.6	Myelodysplastic disease, not classified	Cancer
519971	ICD10CM	C94.80	Other specified leukemias not having achieved remission	Cancer
519972	ICD10CM	C94.81	Other specified leukemias, in remission	Cancer
519973	ICD10CM	C94.82	Other specified leukemias, in relapse	Cancer
519976	ICD10CM	C95.00	Acute leukemia of unspecified cell type not having achieved remission	Cancer
519977	ICD10CM	C95.01	Acute leukemia of unspecified cell type, in remission	Cancer
519978	ICD10CM	C95.02	Acute leukemia of unspecified cell type, in relapse	Cancer
519980	ICD10CM	C95.10	Chronic leukemia of unspecified cell type not having achieved remission	Cancer
519981	ICD10CM	C95.11	Chronic leukemia of unspecified cell type, in remission	Cancer
519982	ICD10CM	C95.12	Chronic leukemia of unspecified cell type, in relapse	Cancer
519988	ICD10CM	C95.90	Leukemia, unspecified not having achieved remission	Cancer
519989	ICD10CM	C95.91	Leukemia, unspecified, in remission	Cancer
519990	ICD10CM	C95.92	Leukemia, unspecified, in relapse	Cancer

519992	ICD10CM	C96.0	Multifocal and multisystemic (disseminated) Langerhans-cell histiocytosis	Cancer
519994	ICD10CM	C96.2	Malignant mast cell neoplasm	Cancer
519995	ICD10CM	C96.20	Malignant mast cell neoplasm, unspecified	Cancer
519996	ICD10CM	C96.21	Aggressive systemic mastocytosis	Cancer
519997	ICD10CM	C96.22	Mast cell sarcoma	Cancer
519998	ICD10CM	C96.29	Other malignant mast cell neoplasm	Cancer
520000	ICD10CM	C96.4	Sarcoma of dendritic cells (accessory cells)	Cancer
520004	ICD10CM	C96.9	Malignant neoplasm of lymphoid, hematopoietic and related tissue, unspecified	Cancer
520005	ICD10CM	C96.A	Histiocytic sarcoma	Cancer
520006	ICD10CM	C96.Z	Other specified malignant neoplasms of lymphoid, hematopoietic and related tissue	Cancer
520041	ICD10CM	D03.0	Melanoma in situ of lip	Cancer
520043	ICD10CM	D03.10	Melanoma in situ of unspecified eyelid, including canthus	Cancer
520044	ICD10CM	D03.11	Melanoma in situ of right eyelid, including canthus	Cancer
520047	ICD10CM	D03.12	Melanoma in situ of left eyelid, including canthus	Cancer
520051	ICD10CM	D03.20	Melanoma in situ of unspecified ear and external auricular canal	Cancer
520052	ICD10CM	D03.21	Melanoma in situ of right ear and external auricular canal	Cancer
520053	ICD10CM	D03.22	Melanoma in situ of left ear and external auricular canal	Cancer
520055	ICD10CM	D03.30	Melanoma in situ of unspecified part of face	Cancer
520056	ICD10CM	D03.39	Melanoma in situ of other parts of face	Cancer
520057	ICD10CM	D03.4	Melanoma in situ of scalp and neck	Cancer
520059	ICD10CM	D03.51	Melanoma in situ of anal skin	Cancer
520060	ICD10CM	D03.52	Melanoma in situ of breast (skin) (soft tissue)	Cancer
520061	ICD10CM	D03.59	Melanoma in situ of other part of trunk	Cancer
520063	ICD10CM	D03.60	Melanoma in situ of unspecified upper limb, including shoulder	Cancer
520064	ICD10CM	D03.61	Melanoma in situ of right upper limb, including shoulder	Cancer
520065	ICD10CM	D03.62	Melanoma in situ of left upper limb, including shoulder	Cancer

520067	ICD10CM	D03.70	Melanoma in situ of unspecified lower limb, including hip	Cancer
520068	ICD10CM	D03.71	Melanoma in situ of right lower limb, including hip	Cancer
520069	ICD10CM	D03.72	Melanoma in situ of left lower limb, including hip	Cancer
520070	ICD10CM	D03.8	Melanoma in situ of other sites	Cancer
520071	ICD10CM	D03.9	Melanoma in situ, unspecified	Cancer
520627	ICD10CM	D47.Z2	Castleman disease	Cancer
520651	ICD10CM	D49.511	Neoplasm of unspecified behavior of right kidney	Cancer
520652	ICD10CM	D49.512	Neoplasm of unspecified behavior of left kidney	Cancer
520653	ICD10CM	D49.519	Neoplasm of unspecified behavior of unspecified kidney	Cancer
520654	ICD10CM	D49.59	Neoplasm of unspecified behavior of other genitourinary organ	Cancer
518653	ICD10CM	C43.111	Malignant melanoma of right upper eyelid, including canthus	Cancer
518654	ICD10CM	C43.112	Malignant melanoma of right lower eyelid, including canthus	Cancer
518656	ICD10CM	C43.121	Malignant melanoma of left upper eyelid, including canthus	Cancer
518657	ICD10CM	C43.122	Malignant melanoma of left lower eyelid, including canthus	Cancer
518691	ICD10CM	C44.1021	Unspecified malignant neoplasm of skin of right upper eyelid, including canthus	Cancer
518692	ICD10CM	C44.1022	Unspecified malignant neoplasm of skin of right lower eyelid, including canthus	Cancer
518694	ICD10CM	C44.1091	Unspecified malignant neoplasm of skin of left upper eyelid, including canthus	Cancer
518695	ICD10CM	C44.1092	Unspecified malignant neoplasm of skin of left lower eyelid, including canthus	Cancer
518699	ICD10CM	C44.1121	Basal cell carcinoma of skin of right upper eyelid, including canthus	Cancer
518700	ICD10CM	C44.1122	Basal cell carcinoma of skin of right lower eyelid, including canthus	Cancer
518702	ICD10CM	C44.1191	Basal cell carcinoma of skin of left upper eyelid, including canthus	Cancer
518703	ICD10CM	C44.1192	Basal cell carcinoma of skin of left lower eyelid, including canthus	Cancer

518707	ICD10CM	C44.1221	Squamous cell carcinoma of skin of right upper eyelid, including canthus	Cancer
518708	ICD10CM	C44.1222	Squamous cell carcinoma of skin of right lower eyelid, including canthus	Cancer
518710	ICD10CM	C44.1291	Squamous cell carcinoma of skin of left upper eyelid, including canthus	Cancer
518711	ICD10CM	C44.1292	Squamous cell carcinoma of skin of left lower eyelid, including canthus	Cancer
518723	ICD10CM	C44.1921	Other specified malignant neoplasm of skin of right upper eyelid, including canthus	Cancer
518724	ICD10CM	C44.1922	Other specified malignant neoplasm of skin of right lower eyelid, including canthus	Cancer
518726	ICD10CM	C44.1991	Other specified malignant neoplasm of skin of left upper eyelid, including canthus	Cancer
518727	ICD10CM	C44.1992	Other specified malignant neoplasm of skin of left lower eyelid, including canthus	Cancer
518896	ICD10CM	C4A.111	Merkel cell carcinoma of right upper eyelid, including canthus	Cancer
518897	ICD10CM	C4A.112	Merkel cell carcinoma of right lower eyelid, including canthus	Cancer
518899	ICD10CM	C4A.121	Merkel cell carcinoma of left upper eyelid, including canthus	Cancer
518900	ICD10CM	C4A.122	Merkel cell carcinoma of left lower eyelid, including canthus	Cancer
520045	ICD10CM	D03.111	Melanoma in situ of right upper eyelid, including canthus	Cancer
520046	ICD10CM	D03.112	Melanoma in situ of right lower eyelid, including canthus	Cancer
520048	ICD10CM	D03.121	Melanoma in situ of left upper eyelid, including canthus	Cancer
520049	ICD10CM	D03.122	Melanoma in situ of left lower eyelid, including canthus	Cancer
1169907	ICD9CM	140	Malignant neoplasm of upper lip, vermilion border	Cancer
1169908	ICD9CM	140.1	Malignant neoplasm of lower lip, vermilion border	Cancer
1169909	ICD9CM	140.3	Malignant neoplasm of upper lip, inner aspect	Cancer
1169910	ICD9CM	140.4	Malignant neoplasm of lower lip, inner aspect	Cancer
1169911	ICD9CM	140.5	Malignant neoplasm of lip, unspecified, inner aspect	Cancer

1169912	ICD9CM	140.6	Malignant neoplasm of commissure of lip	Cancer
1169913	ICD9CM	140.8	Malignant neoplasm of other sites of lip	Cancer
1169914	ICD9CM	140.9	Malignant neoplasm of lip, unspecified, vermilion border	Cancer
1169916	ICD9CM	141	Malignant neoplasm of base of tongue	Cancer
1169917	ICD9CM	141.1	Malignant neoplasm of dorsal surface of tongue	Cancer
1169918	ICD9CM	141.2	Malignant neoplasm of tip and lateral border of tongue	Cancer
1169919	ICD9CM	141.3	Malignant neoplasm of ventral surface of tongue	Cancer
1169920	ICD9CM	141.4	Malignant neoplasm of anterior two-thirds of tongue, part unspecified	Cancer
1169921	ICD9CM	141.5	Malignant neoplasm of junctional zone of tongue	Cancer
1169922	ICD9CM	141.6	Malignant neoplasm of lingual tonsil	Cancer
1169923	ICD9CM	141.8	Malignant neoplasm of other sites of tongue	Cancer
1169924	ICD9CM	141.9	Malignant neoplasm of tongue, unspecified	Cancer
1169926	ICD9CM	142	Malignant neoplasm of parotid gland	Cancer
1169927	ICD9CM	142.1	Malignant neoplasm of submandibular gland	Cancer
1169928	ICD9CM	142.2	Malignant neoplasm of sublingual gland	Cancer
1169929	ICD9CM	142.8	Malignant neoplasm of other major salivary glands	Cancer
1169930	ICD9CM	142.9	Malignant neoplasm of salivary gland, unspecified	Cancer
1169932	ICD9CM	143	Malignant neoplasm of upper gum	Cancer
1169933	ICD9CM	143.1	Malignant neoplasm of lower gum	Cancer
1169934	ICD9CM	143.8	Malignant neoplasm of other sites of gum	Cancer
1169935	ICD9CM	143.9	Malignant neoplasm of gum, unspecified	Cancer
1169937	ICD9CM	144	Malignant neoplasm of anterior portion of floor of mouth	Cancer
1169938	ICD9CM	144.1	Malignant neoplasm of lateral portion of floor of mouth	Cancer
1169939	ICD9CM	144.8	Malignant neoplasm of other sites of floor of mouth	Cancer
1169940	ICD9CM	144.9	Malignant neoplasm of floor of mouth, part unspecified	Cancer

1169942	ICD9CM	145	Malignant neoplasm of cheek mucosa	Cancer
1169943	ICD9CM	145.1	Malignant neoplasm of vestibule of mouth	Cancer
1169944	ICD9CM	145.2	Malignant neoplasm of hard palate	Cancer
1169945	ICD9CM	145.3	Malignant neoplasm of soft palate	Cancer
1169946	ICD9CM	145.4	Malignant neoplasm of uvula	Cancer
1169947	ICD9CM	145.5	Malignant neoplasm of palate, unspecified	Cancer
1169948	ICD9CM	145.6	Malignant neoplasm of retromolar area	Cancer
1169949	ICD9CM	145.8	Malignant neoplasm of other specified parts of mouth	Cancer
1169950	ICD9CM	145.9	Malignant neoplasm of mouth, unspecified	Cancer
1169952	ICD9CM	146	Malignant neoplasm of tonsil	Cancer
1169953	ICD9CM	146.1	Malignant neoplasm of tonsillar fossa	Cancer
1169954	ICD9CM	146.2	Malignant neoplasm of tonsillar pillars (anterior) (posterior)	Cancer
1169955	ICD9CM	146.3	Malignant neoplasm of vallecula epiglottica	Cancer
1169956	ICD9CM	146.4	Malignant neoplasm of anterior aspect of epiglottis	Cancer
1169957	ICD9CM	146.5	Malignant neoplasm of junctional region of oropharynx	Cancer
1169958	ICD9CM	146.6	Malignant neoplasm of lateral wall of oropharynx	Cancer
1169959	ICD9CM	146.7	Malignant neoplasm of posterior wall of oropharynx	Cancer
1169960	ICD9CM	146.8	Malignant neoplasm of other specified sites of oropharynx	Cancer
1169961	ICD9CM	146.9	Malignant neoplasm of oropharynx, unspecified site	Cancer
1169963	ICD9CM	147	Malignant neoplasm of superior wall of nasopharynx	Cancer
1169964	ICD9CM	147.1	Malignant neoplasm of posterior wall of nasopharynx	Cancer
1169965	ICD9CM	147.2	Malignant neoplasm of lateral wall of nasopharynx	Cancer
1169966	ICD9CM	147.3	Malignant neoplasm of anterior wall of nasopharynx	Cancer
1169967	ICD9CM	147.8	Malignant neoplasm of other specified sites of nasopharynx	Cancer
1169968	ICD9CM	147.9	Malignant neoplasm of nasopharynx, unspecified site	Cancer

1169970	ICD9CM	148	Malignant neoplasm of postcricoid region of hypopharynx	Cancer
1169971	ICD9CM	148.1	Malignant neoplasm of pyriform sinus	Cancer
1169972	ICD9CM	148.2	Malignant neoplasm of aryepiglottic fold, hypopharyngeal aspect	Cancer
1169973	ICD9CM	148.3	Malignant neoplasm of posterior hypopharyngeal wall	Cancer
1169974	ICD9CM	148.8	Malignant neoplasm of other specified sites of hypopharynx	Cancer
1169975	ICD9CM	148.9	Malignant neoplasm of hypopharynx, unspecified site	Cancer
1169977	ICD9CM	149	Malignant neoplasm of pharynx, unspecified	Cancer
1169978	ICD9CM	149.1	Malignant neoplasm of waldeyer's ring	Cancer
1169979	ICD9CM	149.8	Malignant neoplasm of other sites within the lip and oral cavity	Cancer
1169980	ICD9CM	149.9	Malignant neoplasm of ill-defined sites within the lip and oral cavity	Cancer
1170001	ICD9CM	150	Malignant neoplasm of cervical esophagus	Cancer
1170002	ICD9CM	150.1	Malignant neoplasm of thoracic esophagus	Cancer
1170003	ICD9CM	150.2	Malignant neoplasm of abdominal esophagus	Cancer
1170004	ICD9CM	150.3	Malignant neoplasm of upper third of esophagus	Cancer
1170005	ICD9CM	150.4	Malignant neoplasm of middle third of esophagus	Cancer
1170006	ICD9CM	150.5	Malignant neoplasm of lower third of esophagus	Cancer
1170007	ICD9CM	150.8	Malignant neoplasm of other specified part of esophagus	Cancer
1170008	ICD9CM	150.9	Malignant neoplasm of esophagus, unspecified site	Cancer
1170010	ICD9CM	151	Malignant neoplasm of cardia	Cancer
1170011	ICD9CM	151.1	Malignant neoplasm of pylorus	Cancer
1170012	ICD9CM	151.2	Malignant neoplasm of pyloric antrum	Cancer
1170013	ICD9CM	151.3	Malignant neoplasm of fundus of stomach	Cancer
1170014	ICD9CM	151.4	Malignant neoplasm of body of stomach	Cancer
1170015	ICD9CM	151.5	Malignant neoplasm of lesser curvature of stomach, unspecified	Cancer

1170016	ICD9CM	151.6	Malignant neoplasm of greater curvature of stomach, unspecified	Cancer
1170017	ICD9CM	151.8	Malignant neoplasm of other specified sites of stomach	Cancer
1170018	ICD9CM	151.9	Malignant neoplasm of stomach, unspecified site	Cancer
1170020	ICD9CM	152	Malignant neoplasm of duodenum	Cancer
1170021	ICD9CM	152.1	Malignant neoplasm of jejunum	Cancer
1170022	ICD9CM	152.2	Malignant neoplasm of ileum	Cancer
1170023	ICD9CM	152.3	Malignant neoplasm of Meckel's diverticulum	Cancer
1170024	ICD9CM	152.8	Malignant neoplasm of other specified sites of small intestine	Cancer
1170025	ICD9CM	152.9	Malignant neoplasm of small intestine, unspecified site	Cancer
1170027	ICD9CM	153	Malignant neoplasm of hepatic flexure	Cancer
1170028	ICD9CM	153.1	Malignant neoplasm of transverse colon	Cancer
1170029	ICD9CM	153.2	Malignant neoplasm of descending colon	Cancer
1170030	ICD9CM	153.3	Malignant neoplasm of sigmoid colon	Cancer
1170031	ICD9CM	153.4	Malignant neoplasm of cecum	Cancer
1170032	ICD9CM	153.5	Malignant neoplasm of appendix vermiformis	Cancer
1170033	ICD9CM	153.6	Malignant neoplasm of ascending colon	Cancer
1170034	ICD9CM	153.7	Malignant neoplasm of splenic flexure	Cancer
1170035	ICD9CM	153.8	Malignant neoplasm of other specified sites of large intestine	Cancer
1170036	ICD9CM	153.9	Malignant neoplasm of colon, unspecified site	Cancer
1170038	ICD9CM	154	Malignant neoplasm of rectosigmoid junction	Cancer
1170039	ICD9CM	154.1	Malignant neoplasm of rectum	Cancer
1170042	ICD9CM	154.8	Malignant neoplasm of other sites of rectum, rectosigmoid junction, and anus	Cancer
1170044	ICD9CM	155	Malignant neoplasm of liver, primary	Cancer
1170045	ICD9CM	155.1	Malignant neoplasm of intrahepatic bile ducts	Cancer
1170046	ICD9CM	155.2	Malignant neoplasm of liver, not specified as primary or secondary	Cancer
1170048	ICD9CM	156	Malignant neoplasm of gallbladder	Cancer

1170049	ICD9CM	156.1	Malignant neoplasm of extrahepatic bile ducts	Cancer
1170050	ICD9CM	156.2	Malignant neoplasm of ampulla of vater	Cancer
1170051	ICD9CM	156.8	Malignant neoplasm of other specified sites of gallbladder and extrahepatic bile ducts	Cancer
1170052	ICD9CM	156.9	Malignant neoplasm of biliary tract, part unspecified site	Cancer
1170054	ICD9CM	157	Malignant neoplasm of head of pancreas	Cancer
1170055	ICD9CM	157.1	Malignant neoplasm of body of pancreas	Cancer
1170056	ICD9CM	157.2	Malignant neoplasm of tail of pancreas	Cancer
1170057	ICD9CM	157.3	Malignant neoplasm of pancreatic duct	Cancer
1170058	ICD9CM	157.4	Malignant neoplasm of islets of langerhans	Cancer
1170059	ICD9CM	157.8	Malignant neoplasm of other specified sites of pancreas	Cancer
1170060	ICD9CM	157.9	Malignant neoplasm of pancreas, part unspecified	Cancer
1170062	ICD9CM	158	Malignant neoplasm of retroperitoneum	Cancer
1170063	ICD9CM	158.8	Malignant neoplasm of specified parts of peritoneum	Cancer
1170064	ICD9CM	158.9	Malignant neoplasm of peritoneum, unspecified	Cancer
1170066	ICD9CM	159	Malignant neoplasm of intestinal tract, part unspecified	Cancer
1170067	ICD9CM	159.1	Malignant neoplasm of spleen, not elsewhere classified	Cancer
1170068	ICD9CM	159.8	Malignant neoplasm of other sites of digestive system and intra-abdominal organs	Cancer
1170069	ICD9CM	159.9	Malignant neoplasm of ill-defined sites within the digestive organs and peritoneum	Cancer
1170114	ICD9CM	160	Malignant neoplasm of nasal cavities	Cancer
1170115	ICD9CM	160.1	Malignant neoplasm of auditory tube, middle ear, and mastoid air cells	Cancer
1170116	ICD9CM	160.2	Malignant neoplasm of maxillary sinus	Cancer
1170117	ICD9CM	160.3	Malignant neoplasm of ethmoidal sinus	Cancer
1170118	ICD9CM	160.4	Malignant neoplasm of frontal sinus	Cancer

1170119	ICD9CM	160.5	Malignant neoplasm of sphenoidal sinus	Cancer
1170120	ICD9CM	160.8	Malignant neoplasm of other accessory sinuses	Cancer
1170121	ICD9CM	160.9	Malignant neoplasm of accessory sinus, unspecified	Cancer
1170123	ICD9CM	161	Malignant neoplasm of glottis	Cancer
1170124	ICD9CM	161.1	Malignant neoplasm of supraglottis	Cancer
1170125	ICD9CM	161.2	Malignant neoplasm of subglottis	Cancer
1170126	ICD9CM	161.3	Malignant neoplasm of laryngeal cartilages	Cancer
1170127	ICD9CM	161.8	Malignant neoplasm of other specified sites of larynx	Cancer
1170128	ICD9CM	161.9	Malignant neoplasm of larynx, unspecified	Cancer
1170130	ICD9CM	162	Malignant neoplasm of trachea	Cancer
1170131	ICD9CM	162.2	Malignant neoplasm of main bronchus	Cancer
1170132	ICD9CM	162.3	Malignant neoplasm of upper lobe, bronchus or lung	Cancer
1170133	ICD9CM	162.4	Malignant neoplasm of middle lobe, bronchus or lung	Cancer
1170134	ICD9CM	162.5	Malignant neoplasm of lower lobe, bronchus or lung	Cancer
1170135	ICD9CM	162.8	Malignant neoplasm of other parts of bronchus or lung	Cancer
1170136	ICD9CM	162.9	Malignant neoplasm of bronchus and lung, unspecified	Cancer
1170138	ICD9CM	163	Malignant neoplasm of parietal pleura	Cancer
1170139	ICD9CM	163.1	Malignant neoplasm of visceral pleura	Cancer
1170140	ICD9CM	163.8	Malignant neoplasm of other specified sites of pleura	Cancer
1170141	ICD9CM	163.9	Malignant neoplasm of pleura, unspecified	Cancer
1170143	ICD9CM	164	Malignant neoplasm of thymus	Cancer
1170144	ICD9CM	164.1	Malignant neoplasm of heart	Cancer
1170145	ICD9CM	164.2	Malignant neoplasm of anterior mediastinum	Cancer
1170146	ICD9CM	164.3	Malignant neoplasm of posterior mediastinum	Cancer
1170147	ICD9CM	164.8	Malignant neoplasm of other parts of mediastinum	Cancer
1170148	ICD9CM	164.9	Malignant neoplasm of mediastinum, part unspecified	Cancer

1170150	ICD9CM	165	Malignant neoplasm of upper respiratory tract, part unspecified	Cancer
1170151	ICD9CM	165.8	Malignant neoplasm of other sites within the respiratory system and intrathoracic organs	Cancer
1170152	ICD9CM	165.9	Malignant neoplasm of ill-defined sites within the respiratory system	Cancer
1170196	ICD9CM	170	Malignant neoplasm of bones of skull and face, except mandible	Cancer
1170197	ICD9CM	170.1	Malignant neoplasm of mandible	Cancer
1170198	ICD9CM	170.2	Malignant neoplasm of vertebral column, excluding sacrum and coccyx	Cancer
1170199	ICD9CM	170.3	Malignant neoplasm of ribs, sternum, and clavicle	Cancer
1170200	ICD9CM	170.4	Malignant neoplasm of scapula and long bones of upper limb	Cancer
1170201	ICD9CM	170.5	Malignant neoplasm of short bones of upper limb	Cancer
1170202	ICD9CM	170.6	Malignant neoplasm of pelvic bones, sacrum, and coccyx	Cancer
1170203	ICD9CM	170.7	Malignant neoplasm of long bones of lower limb	Cancer
1170204	ICD9CM	170.8	Malignant neoplasm of short bones of lower limb	Cancer
1170205	ICD9CM	170.9	Malignant neoplasm of bone and articular cartilage, site unspecified	Cancer
1170207	ICD9CM	171	Malignant neoplasm of connective and other soft tissue of head, face, and neck	Cancer
1170208	ICD9CM	171.2	Malignant neoplasm of connective and other soft tissue of upper limb, including shoulder	Cancer
1170209	ICD9CM	171.3	Malignant neoplasm of connective and other soft tissue of lower limb, including hip	Cancer
1170210	ICD9CM	171.4	Malignant neoplasm of connective and other soft tissue of thorax	Cancer
1170211	ICD9CM	171.5	Malignant neoplasm of connective and other soft tissue of abdomen	Cancer
1170212	ICD9CM	171.6	Malignant neoplasm of connective and other soft tissue of pelvis	Cancer
1170213	ICD9CM	171.7	Malignant neoplasm of connective and other soft tissue of trunk, unspecified	Cancer

1170214	ICD9CM	171.8	Malignant neoplasm of other specified sites of connective and other soft tissue	Cancer
1170215	ICD9CM	171.9	Malignant neoplasm of connective and other soft tissue, site unspecified	Cancer
1170217	ICD9CM	172	Malignant melanoma of skin of lip	Cancer
1170218	ICD9CM	172.1	Malignant melanoma of skin of eyelid, including canthus	Cancer
1170219	ICD9CM	172.2	Malignant melanoma of skin of ear and external auditory canal	Cancer
1170220	ICD9CM	172.3	Malignant melanoma of skin of other and unspecified parts of face	Cancer
1170221	ICD9CM	172.4	Malignant melanoma of skin of scalp and neck	Cancer
1170222	ICD9CM	172.5	Malignant melanoma of skin of trunk, except scrotum	Cancer
1170223	ICD9CM	172.6	Malignant melanoma of skin of upper limb, including shoulder	Cancer
1170224	ICD9CM	172.7	Malignant melanoma of skin of lower limb, including hip	Cancer
1170225	ICD9CM	172.8	Malignant melanoma of other specified sites of skin	Cancer
1170226	ICD9CM	172.9	Melanoma of skin, site unspecified	Cancer
1170229	ICD9CM	173	Unspecified malignant neoplasm of skin of lip	Cancer
1170230	ICD9CM	173.01	Basal cell carcinoma of skin of lip	Cancer
1170231	ICD9CM	173.02	Squamous cell carcinoma of skin of lip	Cancer
1170232	ICD9CM	173.09	Other specified malignant neoplasm of skin of lip	Cancer
1170234	ICD9CM	173.1	Unspecified malignant neoplasm of eyelid, including canthus	Cancer
1170235	ICD9CM	173.11	Basal cell carcinoma of eyelid, including canthus	Cancer
1170236	ICD9CM	173.12	Squamous cell carcinoma of eyelid, including canthus	Cancer
1170237	ICD9CM	173.19	Other specified malignant neoplasm of eyelid, including canthus	Cancer
1170239	ICD9CM	173.2	Unspecified malignant neoplasm of skin of ear and external auditory canal	Cancer
1170240	ICD9CM	173.21	Basal cell carcinoma of skin of ear and external auditory canal	Cancer
1170241	ICD9CM	173.22	Squamous cell carcinoma of skin of ear and external auditory canal	Cancer

1170242	ICD9CM	173.29	Other specified malignant neoplasm of skin of ear and external auditory canal	Cancer
1170244	ICD9CM	173.3	Unspecified malignant neoplasm of skin of other and unspecified parts of face	Cancer
1170245	ICD9CM	173.31	Basal cell carcinoma of skin of other and unspecified parts of face	Cancer
1170246	ICD9CM	173.32	Squamous cell carcinoma of skin of other and unspecified parts of face	Cancer
1170247	ICD9CM	173.39	Other specified malignant neoplasm of skin of other and unspecified parts of face	Cancer
1170249	ICD9CM	173.4	Unspecified malignant neoplasm of scalp and skin of neck	Cancer
1170250	ICD9CM	173.41	Basal cell carcinoma of scalp and skin of neck	Cancer
1170251	ICD9CM	173.42	Squamous cell carcinoma of scalp and skin of neck	Cancer
1170252	ICD9CM	173.49	Other specified malignant neoplasm of scalp and skin of neck	Cancer
1170254	ICD9CM	173.5	Unspecified malignant neoplasm of skin of trunk, except scrotum	Cancer
1170255	ICD9CM	173.51	Basal cell carcinoma of skin of trunk, except scrotum	Cancer
1170256	ICD9CM	173.52	Squamous cell carcinoma of skin of trunk, except scrotum	Cancer
1170257	ICD9CM	173.59	Other specified malignant neoplasm of skin of trunk, except scrotum	Cancer
1170259	ICD9CM	173.6	Unspecified malignant neoplasm of skin of upper limb, including shoulder	Cancer
1170260	ICD9CM	173.61	Basal cell carcinoma of skin of upper limb, including shoulder	Cancer
1170261	ICD9CM	173.62	Squamous cell carcinoma of skin of upper limb, including shoulder	Cancer
1170262	ICD9CM	173.69	Other specified malignant neoplasm of skin of upper limb, including shoulder	Cancer
1170264	ICD9CM	173.7	Unspecified malignant neoplasm of skin of lower limb, including hip	Cancer
1170265	ICD9CM	173.71	Basal cell carcinoma of skin of lower limb, including hip	Cancer
1170266	ICD9CM	173.72	Squamous cell carcinoma of skin of lower limb, including hip	Cancer

1170267	ICD9CM	173.79	Other specified malignant neoplasm of skin of lower limb, including hip	Cancer
1170269	ICD9CM	173.8	Unspecified malignant neoplasm of other specified sites of skin	Cancer
1170270	ICD9CM	173.81	Basal cell carcinoma of other specified sites of skin	Cancer
1170271	ICD9CM	173.82	Squamous cell carcinoma of other specified sites of skin	Cancer
1170272	ICD9CM	173.89	Other specified malignant neoplasm of other specified sites of skin	Cancer
1170274	ICD9CM	173.9	Unspecified malignant neoplasm of skin, site unspecified	Cancer
1170275	ICD9CM	173.91	Basal cell carcinoma of skin, site unspecified	Cancer
1170276	ICD9CM	173.92	Squamous cell carcinoma of skin, site unspecified	Cancer
1170277	ICD9CM	173.99	Other specified malignant neoplasm of skin, site unspecified	Cancer
1170279	ICD9CM	174	Malignant neoplasm of nipple and areola of female breast	Cancer
1170280	ICD9CM	174.1	Malignant neoplasm of central portion of female breast	Cancer
1170281	ICD9CM	174.2	Malignant neoplasm of upper-inner quadrant of female breast	Cancer
1170282	ICD9CM	174.3	Malignant neoplasm of lower-inner quadrant of female breast	Cancer
1170283	ICD9CM	174.4	Malignant neoplasm of upper-outer quadrant of female breast	Cancer
1170284	ICD9CM	174.5	Malignant neoplasm of lower-outer quadrant of female breast	Cancer
1170285	ICD9CM	174.6	Malignant neoplasm of axillary tail of female breast	Cancer
1170286	ICD9CM	174.8	Malignant neoplasm of other specified sites of female breast	Cancer
1170287	ICD9CM	174.9	Malignant neoplasm of breast (female), unspecified	Cancer
1170289	ICD9CM	175	Malignant neoplasm of nipple and areola of male breast	Cancer
1170290	ICD9CM	175.9	Malignant neoplasm of other and unspecified sites of male breast	Cancer
1170292	ICD9CM	176	Kaposi's sarcoma, skin	Cancer
1170293	ICD9CM	176.1	Kaposi's sarcoma, soft tissue	Cancer
1170294	ICD9CM	176.2	Kaposi's sarcoma, palate	Cancer
1170295	ICD9CM	176.3	Kaposi's sarcoma, gastrointestinal sites	Cancer
1170296	ICD9CM	176.4	Kaposi's sarcoma, lung	Cancer

1170297	ICD9CM	176.5	Kaposi's sarcoma, lymph nodes	Cancer
1170298	ICD9CM	176.8	Kaposi's sarcoma, other specified sites	Cancer
1170299	ICD9CM	176.9	Kaposi's sarcoma, unspecified site	Cancer
1170300	ICD9CM	179	Malignant neoplasm of uterus, part unspecified	Cancer
1170325	ICD9CM	180	Malignant neoplasm of endocervix	Cancer
1170326	ICD9CM	180.1	Malignant neoplasm of exocervix	Cancer
1170327	ICD9CM	180.8	Malignant neoplasm of other specified sites of cervix	Cancer
1170328	ICD9CM	180.9	Malignant neoplasm of cervix uteri, unspecified site	Cancer
1170329	ICD9CM	181	Malignant neoplasm of placenta	Cancer
1170331	ICD9CM	182	Malignant neoplasm of corpus uteri, except isthmus	Cancer
1170332	ICD9CM	182.1	Malignant neoplasm of isthmus	Cancer
1170333	ICD9CM	182.8	Malignant neoplasm of other specified sites of body of uterus	Cancer
1170335	ICD9CM	183	Malignant neoplasm of ovary	Cancer
1170336	ICD9CM	183.2	Malignant neoplasm of fallopian tube	Cancer
1170337	ICD9CM	183.3	Malignant neoplasm of broad ligament of uterus	Cancer
1170338	ICD9CM	183.4	Malignant neoplasm of parametrium	Cancer
1170339	ICD9CM	183.5	Malignant neoplasm of round ligament of uterus	Cancer
1170340	ICD9CM	183.8	Malignant neoplasm of other specified sites of uterine adnexa	Cancer
1170341	ICD9CM	183.9	Malignant neoplasm of uterine adnexa, unspecified site	Cancer
1170343	ICD9CM	184	Malignant neoplasm of vagina	Cancer
1170344	ICD9CM	184.1	Malignant neoplasm of labia majora	Cancer
1170345	ICD9CM	184.2	Malignant neoplasm of labia minora	Cancer
1170346	ICD9CM	184.3	Malignant neoplasm of clitoris	Cancer
1170347	ICD9CM	184.4	Malignant neoplasm of vulva, unspecified site	Cancer
1170348	ICD9CM	184.8	Malignant neoplasm of other specified sites of female genital organs	Cancer
1170349	ICD9CM	184.9	Malignant neoplasm of female genital organ, site unspecified	Cancer
1170350	ICD9CM	185	Malignant neoplasm of prostate	Cancer
1170352	ICD9CM	186	Malignant neoplasm of undescended testis	Cancer

1170353	ICD9CM	186.9	Malignant neoplasm of other and unspecified testis	Cancer
1170355	ICD9CM	187.1	Malignant neoplasm of prepuce	Cancer
1170356	ICD9CM	187.2	Malignant neoplasm of glans penis	Cancer
1170357	ICD9CM	187.3	Malignant neoplasm of body of penis	Cancer
1170358	ICD9CM	187.4	Malignant neoplasm of penis, part unspecified	Cancer
1170359	ICD9CM	187.5	Malignant neoplasm of epididymis	Cancer
1170360	ICD9CM	187.6	Malignant neoplasm of spermatic cord	Cancer
1170361	ICD9CM	187.7	Malignant neoplasm of scrotum	Cancer
1170362	ICD9CM	187.8	Malignant neoplasm of other specified sites of male genital organs	Cancer
1170363	ICD9CM	187.9	Malignant neoplasm of male genital organ, site unspecified	Cancer
1170365	ICD9CM	188	Malignant neoplasm of trigone of urinary bladder	Cancer
1170366	ICD9CM	188.1	Malignant neoplasm of dome of urinary bladder	Cancer
1170367	ICD9CM	188.2	Malignant neoplasm of lateral wall of urinary bladder	Cancer
1170368	ICD9CM	188.3	Malignant neoplasm of anterior wall of urinary bladder	Cancer
1170369	ICD9CM	188.4	Malignant neoplasm of posterior wall of urinary bladder	Cancer
1170370	ICD9CM	188.5	Malignant neoplasm of bladder neck	Cancer
1170371	ICD9CM	188.6	Malignant neoplasm of ureteric orifice	Cancer
1170372	ICD9CM	188.7	Malignant neoplasm of urachus	Cancer
1170373	ICD9CM	188.8	Malignant neoplasm of other specified sites of bladder	Cancer
1170374	ICD9CM	188.9	Malignant neoplasm of bladder, part unspecified	Cancer
1170376	ICD9CM	189	Malignant neoplasm of kidney, except pelvis	Cancer
1170377	ICD9CM	189.1	Malignant neoplasm of renal pelvis	Cancer
1170378	ICD9CM	189.2	Malignant neoplasm of ureter	Cancer
1170379	ICD9CM	189.3	Malignant neoplasm of urethra	Cancer
1170380	ICD9CM	189.4	Malignant neoplasm of paraurethral glands	Cancer
1170381	ICD9CM	189.8	Malignant neoplasm of other specified sites of urinary organs	Cancer
1170382	ICD9CM	189.9	Malignant neoplasm of urinary organ, site unspecified	Cancer

1170401	ICD9CM	190	Malignant neoplasm of eyeball, except conjunctiva, cornea, retina, and choroid	Cancer
1170402	ICD9CM	190.1	Malignant neoplasm of orbit	Cancer
1170403	ICD9CM	190.2	Malignant neoplasm of lacrimal gland	Cancer
1170404	ICD9CM	190.3	Malignant neoplasm of conjunctiva	Cancer
1170405	ICD9CM	190.4	Malignant neoplasm of cornea	Cancer
1170406	ICD9CM	190.5	Malignant neoplasm of retina	Cancer
1170407	ICD9CM	190.6	Malignant neoplasm of choroid	Cancer
1170408	ICD9CM	190.7	Malignant neoplasm of lacrimal duct	Cancer
1170409	ICD9CM	190.8	Malignant neoplasm of other specified sites of eye	Cancer
1170410	ICD9CM	190.9	Malignant neoplasm of eye, part unspecified	Cancer
1170412	ICD9CM	191	Malignant neoplasm of cerebrum, except lobes and ventricles	Cancer
1170413	ICD9CM	191.1	Malignant neoplasm of frontal lobe	Cancer
1170414	ICD9CM	191.2	Malignant neoplasm of temporal lobe	Cancer
1170415	ICD9CM	191.3	Malignant neoplasm of parietal lobe	Cancer
1170416	ICD9CM	191.4	Malignant neoplasm of occipital lobe	Cancer
1170417	ICD9CM	191.5	Malignant neoplasm of ventricles	Cancer
1170418	ICD9CM	191.6	Malignant neoplasm of cerebellum nos	Cancer
1170419	ICD9CM	191.7	Malignant neoplasm of brain stem	Cancer
1170420	ICD9CM	191.8	Malignant neoplasm of other parts of brain	Cancer
1170421	ICD9CM	191.9	Malignant neoplasm of brain, unspecified	Cancer
1170423	ICD9CM	192	Malignant neoplasm of cranial nerves	Cancer
1170424	ICD9CM	192.1	Malignant neoplasm of cerebral meninges	Cancer
1170425	ICD9CM	192.2	Malignant neoplasm of spinal cord	Cancer
1170426	ICD9CM	192.3	Malignant neoplasm of spinal meninges	Cancer
1170427	ICD9CM	192.8	Malignant neoplasm of other specified sites of nervous system	Cancer
1170428	ICD9CM	192.9	Malignant neoplasm of nervous system, part unspecified	Cancer

1170431	ICD9CM	194	Malignant neoplasm of adrenal gland	Cancer
1170432	ICD9CM	194.1	Malignant neoplasm of parathyroid gland	Cancer
1170433	ICD9CM	194.3	Malignant neoplasm of pituitary gland and craniopharyngeal duct	Cancer
1170434	ICD9CM	194.4	Malignant neoplasm of pineal gland	Cancer
1170435	ICD9CM	194.5	Malignant neoplasm of carotid body	Cancer
1170436	ICD9CM	194.6	Malignant neoplasm of aortic body and other paraganglia	Cancer
1170437	ICD9CM	194.8	Malignant neoplasm of other endocrine glands and related structures	Cancer
1170438	ICD9CM	194.9	Malignant neoplasm of endocrine gland, site unspecified	Cancer
1170440	ICD9CM	195	Malignant neoplasm of head, face, and neck	Cancer
1170441	ICD9CM	195.1	Malignant neoplasm of thorax	Cancer
1170442	ICD9CM	195.2	Malignant neoplasm of abdomen	Cancer
1170443	ICD9CM	195.3	Malignant neoplasm of pelvis	Cancer
1170444	ICD9CM	195.4	Malignant neoplasm of upper limb	Cancer
1170445	ICD9CM	195.5	Malignant neoplasm of lower limb	Cancer
1170446	ICD9CM	195.8	Malignant neoplasm of other specified sites	Cancer
1170448	ICD9CM	196	Secondary and unspecified malignant neoplasm of lymph nodes of head, face, and neck	Cancer
1170449	ICD9CM	196.1	Secondary and unspecified malignant neoplasm of intrathoracic lymph nodes	Cancer
1170450	ICD9CM	196.2	Secondary and unspecified malignant neoplasm of intra-abdominal lymph nodes	Cancer
1170451	ICD9CM	196.3	Secondary and unspecified malignant neoplasm of lymph nodes of axilla and upper limb	Cancer
1170452	ICD9CM	196.5	Secondary and unspecified malignant neoplasm of lymph nodes of inguinal region and lower limb	Cancer
1170453	ICD9CM	196.6	Secondary and unspecified malignant neoplasm of intrapelvic lymph nodes	Cancer
1170454	ICD9CM	196.8	Secondary and unspecified malignant neoplasm of lymph nodes of multiple sites	Cancer

1170455	ICD9CM	196.9	Secondary and unspecified malignant neoplasm of lymph nodes, site unspecified	Cancer
1170462	ICD9CM	197.5	Secondary malignant neoplasm of large intestine and rectum	Cancer
1170523	ICD9CM	200	Reticulosarcoma, unspecified site, extranodal and solid organ sites	Cancer
1170524	ICD9CM	200.01	Reticulosarcoma, lymph nodes of head, face, and neck	Cancer
1170525	ICD9CM	200.02	Reticulosarcoma, intrathoracic lymph nodes	Cancer
1170526	ICD9CM	200.03	Reticulosarcoma, intra-abdominal lymph nodes	Cancer
1170527	ICD9CM	200.04	Reticulosarcoma, lymph nodes of axilla and upper limb	Cancer
1170528	ICD9CM	200.05	Reticulosarcoma, lymph nodes of inguinal region and lower limb	Cancer
1170529	ICD9CM	200.06	Reticulosarcoma, intrapelvic lymph nodes	Cancer
1170530	ICD9CM	200.07	Reticulosarcoma, spleen	Cancer
1170531	ICD9CM	200.08	Reticulosarcoma, lymph nodes of multiple sites	Cancer
1170533	ICD9CM	200.1	Lymphosarcoma, unspecified site, extranodal and solid organ sites	Cancer
1170534	ICD9CM	200.11	Lymphosarcoma, lymph nodes of head, face, and neck	Cancer
1170535	ICD9CM	200.12	Lymphosarcoma, intrathoracic lymph nodes	Cancer
1170536	ICD9CM	200.13	Lymphosarcoma, intra-abdominal lymph nodes	Cancer
1170537	ICD9CM	200.14	Lymphosarcoma, lymph nodes of axilla and upper limb	Cancer
1170538	ICD9CM	200.15	Lymphosarcoma, lymph nodes of inguinal region and lower limb	Cancer
1170539	ICD9CM	200.16	Lymphosarcoma, intrapelvic lymph nodes	Cancer
1170540	ICD9CM	200.17	Lymphosarcoma, spleen	Cancer
1170541	ICD9CM	200.18	Lymphosarcoma, lymph nodes of multiple sites	Cancer
1170543	ICD9CM	200.2	Burkitt's tumor or lymphoma, unspecified site, extranodal and solid organ sites	Cancer
1170544	ICD9CM	200.21	Burkitt's tumor or lymphoma, lymph nodes of head, face, and neck	Cancer
1170545	ICD9CM	200.22	Burkitt's tumor or lymphoma, intrathoracic lymph nodes	Cancer

1170546	ICD9CM	200.23	Burkitt's tumor or lymphoma, intra-abdominal lymph nodes	Cancer
1170547	ICD9CM	200.24	Burkitt's tumor or lymphoma, lymph nodes of axilla and upper limb	Cancer
1170548	ICD9CM	200.25	Burkitt's tumor or lymphoma, lymph nodes of inguinal region and lower limb	Cancer
1170549	ICD9CM	200.26	Burkitt's tumor or lymphoma, intrapelvic lymph nodes	Cancer
1170550	ICD9CM	200.27	Burkitt's tumor or lymphoma, spleen	Cancer
1170551	ICD9CM	200.28	Burkitt's tumor or lymphoma, lymph nodes of multiple sites	Cancer
1170553	ICD9CM	200.3	Marginal zone lymphoma, unspecified site, extranodal and solid organ sites	Cancer
1170554	ICD9CM	200.31	Marginal zone lymphoma, lymph nodes of head, face, and neck	Cancer
1170555	ICD9CM	200.32	Marginal zone lymphoma, intrathoracic lymph nodes	Cancer
1170556	ICD9CM	200.33	Marginal zone lymphoma, intraabdominal lymph nodes	Cancer
1170557	ICD9CM	200.34	Marginal zone lymphoma, lymph nodes of axilla and upper limb	Cancer
1170558	ICD9CM	200.35	Marginal zone lymphoma, lymph nodes of inguinal region and lower limb	Cancer
1170559	ICD9CM	200.36	Marginal zone lymphoma, intrapelvic lymph nodes	Cancer
1170560	ICD9CM	200.37	Marginal zone lymphoma, spleen	Cancer
1170561	ICD9CM	200.38	Marginal zone lymphoma, lymph nodes of multiple sites	Cancer
1170563	ICD9CM	200.4	Mantle cell lymphoma, unspecified site, extranodal and solid organ sites	Cancer
1170564	ICD9CM	200.41	Mantle cell lymphoma, lymph nodes of head, face, and neck	Cancer
1170565	ICD9CM	200.42	Mantle cell lymphoma, intrathoracic lymph nodes	Cancer
1170566	ICD9CM	200.43	Mantle cell lymphoma, intra-abdominal lymph nodes	Cancer
1170567	ICD9CM	200.44	Mantle cell lymphoma, lymph nodes of axilla and upper limb	Cancer
1170568	ICD9CM	200.45	Mantle cell lymphoma, lymph nodes of inguinal region and lower limb	Cancer
1170569	ICD9CM	200.46	Mantle cell lymphoma, intrapelvic lymph nodes	Cancer
1170570	ICD9CM	200.47	Mantle cell lymphoma, spleen	Cancer

1170571	ICD9CM	200.48	Mantle cell lymphoma, lymph nodes of multiple sites	Cancer
1170573	ICD9CM	200.5	Primary central nervous system lymphoma, unspecified site, extranodal and solid organ sites	Cancer
1170574	ICD9CM	200.51	Primary central nervous system lymphoma, lymph nodes of head, face, and neck	Cancer
1170575	ICD9CM	200.52	Primary central nervous system lymphoma, intrathoracic lymph nodes	Cancer
1170576	ICD9CM	200.53	Primary central nervous system lymphoma, intra-abdominal lymph nodes	Cancer
1170577	ICD9CM	200.54	Primary central nervous system lymphoma, lymph nodes of axilla and upper limb	Cancer
1170578	ICD9CM	200.55	Primary central nervous system lymphoma, lymph nodes of inguinal region and lower limb	Cancer
1170579	ICD9CM	200.56	Primary central nervous system lymphoma, intrapelvic lymph nodes	Cancer
1170580	ICD9CM	200.57	Primary central nervous system lymphoma, spleen	Cancer
1170581	ICD9CM	200.58	Primary central nervous system lymphoma, lymph nodes of multiple sites	Cancer
1170583	ICD9CM	200.6	Anaplastic large cell lymphoma, unspecified site, extranodal and solid organ sites	Cancer
1170584	ICD9CM	200.61	Anaplastic large cell lymphoma, lymph nodes of head, face, and neck	Cancer
1170585	ICD9CM	200.62	Anaplastic large cell lymphoma, intrathoracic lymph nodes	Cancer
1170586	ICD9CM	200.63	Anaplastic large cell lymphoma, intra-abdominal lymph nodes	Cancer
1170587	ICD9CM	200.64	Anaplastic large cell lymphoma, lymph nodes of axilla and upper limb	Cancer
1170588	ICD9CM	200.65	Anaplastic large cell lymphoma, lymph nodes of inguinal region and lower limb	Cancer
1170589	ICD9CM	200.66	Anaplastic large cell lymphoma, intrapelvic lymph nodes	Cancer
1170590	ICD9CM	200.67	Anaplastic large cell lymphoma, spleen	Cancer
1170591	ICD9CM	200.68	Anaplastic large cell lymphoma, lymph nodes of multiple sites	Cancer

1170593	ICD9CM	200.7	Large cell lymphoma, unspecified site, extranodal and solid organ sites	Cancer
1170594	ICD9CM	200.71	Large cell lymphoma, lymph nodes of head, face, and neck	Cancer
1170595	ICD9CM	200.72	Large cell lymphoma, intrathoracic lymph nodes	Cancer
1170596	ICD9CM	200.73	Large cell lymphoma, intra-abdominal lymph nodes	Cancer
1170597	ICD9CM	200.74	Large cell lymphoma, lymph nodes of axilla and upper limb	Cancer
1170598	ICD9CM	200.75	Large cell lymphoma, lymph nodes of inguinal region and lower limb	Cancer
1170599	ICD9CM	200.76	Large cell lymphoma, intrapelvic lymph nodes	Cancer
1170600	ICD9CM	200.77	Large cell lymphoma, spleen	Cancer
1170601	ICD9CM	200.78	Large cell lymphoma, lymph nodes of multiple sites	Cancer
1170603	ICD9CM	200.8	Other named variants of lymphosarcoma and reticulosarcoma, unspecified site, extranodal and solid organ sites	Cancer
1170604	ICD9CM	200.81	Other named variants of lymphosarcoma and reticulosarcoma, lymph nodes of head, face, and neck	Cancer
1170605	ICD9CM	200.82	Other named variants of lymphosarcoma and reticulosarcoma,intrathoracic lymph nodes	Cancer
1170606	ICD9CM	200.83	Other named variants of lymphosarcoma and reticulosarcoma, intra-abdominal lymph nodes	Cancer
1170607	ICD9CM	200.84	Other named variants of lymphosarcoma and reticulosarcoma, lymph nodes of axilla and upper limb	Cancer
1170608	ICD9CM	200.85	Other named variants of lymphosarcoma and reticulosarcoma, lymph nodes of inguinal region and lower limb	Cancer
1170609	ICD9CM	200.86	Other named variants of lymphosarcoma and reticulosarcoma, intrapelvic lymph nodes	Cancer
1170610	ICD9CM	200.87	Other named variants of lymphosarcoma and reticulosarcoma, spleen	Cancer

1170611	ICD9CM	200.88	Other named variants of lymphosarcoma and reticulosarcoma, lymph nodes of multiple sites	Cancer
1170614	ICD9CM	201	Hodgkin's paragranuloma, unspecified site, extranodal and solid organ sites	Cancer
1170615	ICD9CM	201.01	Hodgkin's paragranuloma, lymph nodes of head, face, and neck	Cancer
1170616	ICD9CM	201.02	Hodgkin's paragranuloma, intrathoracic lymph nodes	Cancer
1170617	ICD9CM	201.03	Hodgkin's paragranuloma, intra-abdominal lymph nodes	Cancer
1170618	ICD9CM	201.04	Hodgkin's paragranuloma, lymph nodes of axilla and upper limb	Cancer
1170619	ICD9CM	201.05	Hodgkin's paragranuloma, lymph nodes of inguinal region and lower limb	Cancer
1170620	ICD9CM	201.06	Hodgkin's paragranuloma, intrapelvic lymph nodes	Cancer
1170621	ICD9CM	201.07	Hodgkin's paragranuloma, spleen	Cancer
1170622	ICD9CM	201.08	Hodgkin's paragranuloma, lymph nodes of multiple sites	Cancer
1170624	ICD9CM	201.1	Hodgkin's granuloma, unspecified site, extranodal and solid organ sites	Cancer
1170625	ICD9CM	201.11	Hodgkin's granuloma, lymph nodes of head, face, and neck	Cancer
1170626	ICD9CM	201.12	Hodgkin's granuloma, intrathoracic lymph nodes	Cancer
1170627	ICD9CM	201.13	Hodgkin's granuloma, intra-abdominal lymph nodes	Cancer
1170628	ICD9CM	201.14	Hodgkin's granuloma, lymph nodes of axilla and upper limb	Cancer
1170629	ICD9CM	201.15	Hodgkin's granuloma, lymph nodes of inguinal region and lower limb	Cancer
1170630	ICD9CM	201.16	Hodgkin's granuloma, intrapelvic lymph nodes	Cancer
1170631	ICD9CM	201.17	Hodgkin's granuloma, spleen	Cancer
1170632	ICD9CM	201.18	Hodgkin's granuloma, lymph nodes of multiple sites	Cancer
1170634	ICD9CM	201.2	Hodgkin's sarcoma, unspecified site, extranodal and solid organ sites	Cancer
1170635	ICD9CM	201.21	Hodgkin's sarcoma, lymph nodes of head, face, and neck	Cancer
1170636	ICD9CM	201.22	Hodgkin's sarcoma, intrathoracic lymph nodes	Cancer

1170637	ICD9CM	201.23	Hodgkin's sarcoma, intra-abdominal lymph nodes	Cancer
1170638	ICD9CM	201.24	Hodgkin's sarcoma, lymph nodes of axilla and upper limb	Cancer
1170639	ICD9CM	201.25	Hodgkin's sarcoma, lymph nodes of inguinal region and lower limb	Cancer
1170640	ICD9CM	201.26	Hodgkin's sarcoma, intrapelvic lymph nodes	Cancer
1170641	ICD9CM	201.27	Hodgkin's sarcoma, spleen	Cancer
1170642	ICD9CM	201.28	Hodgkin's sarcoma, lymph nodes of multiple sites	Cancer
1170644	ICD9CM	201.4	Hodgkin's disease, lymphocytic-histiocytic predominance, unspecified site, extranodal and solid organ sites	Cancer
1170645	ICD9CM	201.41	Hodgkin's disease, lymphocytic-histiocytic predominance, lymph nodes of head, face, and neck	Cancer
1170646	ICD9CM	201.42	Hodgkin's disease, lymphocytic-histiocytic predominance, intrathoracic lymph nodes	Cancer
1170647	ICD9CM	201.43	Hodgkin's disease, lymphocytic-histiocytic predominance, intra-abdominal lymph nodes	Cancer
1170648	ICD9CM	201.44	Hodgkin's disease, lymphocytic-histiocytic predominance, lymph nodes of axilla and upper limb	Cancer
1170649	ICD9CM	201.45	Hodgkin's disease, lymphocytic-histiocytic predominance, lymph nodes of inguinal region and lower limb	Cancer
1170650	ICD9CM	201.46	Hodgkin's disease, lymphocytic-histiocytic predominance, intrapelvic lymph nodes	Cancer
1170651	ICD9CM	201.47	Hodgkin's disease, lymphocytic-histiocytic predominance, spleen	Cancer
1170652	ICD9CM	201.48	Hodgkin's disease, lymphocytic-histiocytic predominance, lymph nodes of multiple sites	Cancer
1170654	ICD9CM	201.5	Hodgkin's disease, nodular sclerosis, unspecified site, extranodal and solid organ sites	Cancer
1170655	ICD9CM	201.51	Hodgkin's disease, nodular sclerosis, lymph nodes of head, face, and neck	Cancer
1170656	ICD9CM	201.52	Hodgkin's disease, nodular sclerosis, intrathoracic lymph nodes	Cancer

1170657	ICD9CM	201.53	Hodgkin's disease, nodular sclerosis, intra-abdominal lymph nodes	Cancer
1170658	ICD9CM	201.54	Hodgkin's disease, nodular sclerosis, lymph nodes of axilla and upper limb	Cancer
1170659	ICD9CM	201.55	Hodgkin's disease, nodular sclerosis, lymph nodes of inguinal region and lower limb	Cancer
1170660	ICD9CM	201.56	Hodgkin's disease, nodular sclerosis, intrapelvic lymph nodes	Cancer
1170661	ICD9CM	201.57	Hodgkin's disease, nodular sclerosis, spleen	Cancer
1170662	ICD9CM	201.58	Hodgkin's disease, nodular sclerosis, lymph nodes of multiple sites	Cancer
1170664	ICD9CM	201.6	Hodgkin's disease, mixed cellularity, unspecified site, extranodal and solid organ sites	Cancer
1170665	ICD9CM	201.61	Hodgkin's disease, mixed cellularity, lymph nodes of head, face, and neck	Cancer
1170666	ICD9CM	201.62	Hodgkin's disease, mixed cellularity, intrathoracic lymph nodes	Cancer
1170667	ICD9CM	201.63	Hodgkin's disease, mixed cellularity, intra-abdominal lymph nodes	Cancer
1170668	ICD9CM	201.64	Hodgkin's disease, mixed cellularity, lymph nodes of axilla and upper limb	Cancer
1170669	ICD9CM	201.65	Hodgkin's disease, mixed cellularity, lymph nodes of inguinal region and lower limb	Cancer
1170670	ICD9CM	201.66	Hodgkin's disease, mixed cellularity, intrapelvic lymph nodes	Cancer
1170671	ICD9CM	201.67	Hodgkin's disease, mixed cellularity, spleen	Cancer
1170672	ICD9CM	201.68	Hodgkin's disease, mixed cellularity, lymph nodes of multiple sites	Cancer
1170674	ICD9CM	201.7	Hodgkin's disease, lymphocytic depletion, unspecified site, extranodal and solid organ sites	Cancer
1170675	ICD9CM	201.71	Hodgkin's disease, lymphocytic depletion, lymph nodes of head, face, and neck	Cancer

1170676	ICD9CM	201.72	Hodgkin's disease, lymphocytic depletion, intrathoracic lymph nodes	Cancer
1170677	ICD9CM	201.73	Hodgkin's disease, lymphocytic depletion, intra-abdominal lymph nodes	Cancer
1170678	ICD9CM	201.74	Hodgkin's disease, lymphocytic depletion, lymph nodes of axilla and upper limb	Cancer
1170679	ICD9CM	201.75	Hodgkin's disease, lymphocytic depletion, lymph nodes of inguinal region and lower limb	Cancer
1170680	ICD9CM	201.76	Hodgkin's disease, lymphocytic depletion, intrapelvic lymph nodes	Cancer
1170681	ICD9CM	201.77	Hodgkin's disease, lymphocytic depletion, spleen	Cancer
1170682	ICD9CM	201.78	Hodgkin's disease, lymphocytic depletion, lymph nodes of multiple sites	Cancer
1170684	ICD9CM	201.9	Hodgkin's disease, unspecified type, unspecified site, extranodal and solid organ sites	Cancer
1170685	ICD9CM	201.91	Hodgkin's disease, unspecified type, lymph nodes of head, face, and neck	Cancer
1170686	ICD9CM	201.92	Hodgkin's disease, unspecified type, intrathoracic lymph nodes	Cancer
1170687	ICD9CM	201.93	Hodgkin's disease, unspecified type, intra-abdominal lymph nodes	Cancer
1170688	ICD9CM	201.94	Hodgkin's disease, unspecified type, lymph nodes of axilla and upper limb	Cancer
1170689	ICD9CM	201.95	Hodgkin's disease, unspecified type, lymph nodes of inguinal region and lower limb	Cancer
1170690	ICD9CM	201.96	Hodgkin's disease, unspecified type, intrapelvic lymph nodes	Cancer
1170691	ICD9CM	201.97	Hodgkin's disease, unspecified type, spleen	Cancer
1170692	ICD9CM	201.98	Hodgkin's disease, unspecified type, lymph nodes of multiple sites	Cancer
1170695	ICD9CM	202	Nodular lymphoma, unspecified site, extranodal and solid organ sites	Cancer
1170696	ICD9CM	202.01	Nodular lymphoma, lymph nodes of head, face, and neck	Cancer
1170697	ICD9CM	202.02	Nodular lymphoma, intrathoracic lymph nodes	Cancer

1170698	ICD9CM	202.03	Nodular lymphoma, intra-abdominal lymph nodes	Cancer
1170699	ICD9CM	202.04	Nodular lymphoma, lymph nodes of axilla and upper limb	Cancer
1170700	ICD9CM	202.05	Nodular lymphoma, lymph nodes of inguinal region and lower limb	Cancer
1170701	ICD9CM	202.06	Nodular lymphoma, intrapelvic lymph nodes	Cancer
1170702	ICD9CM	202.07	Nodular lymphoma, spleen	Cancer
1170703	ICD9CM	202.08	Nodular lymphoma, lymph nodes of multiple sites	Cancer
1170705	ICD9CM	202.1	Mycosis fungoides, unspecified site, extranodal and solid organ sites	Cancer
1170706	ICD9CM	202.11	Mycosis fungoides, lymph nodes of head, face, and neck	Cancer
1170707	ICD9CM	202.12	Mycosis fungoides, intrathoracic lymph nodes	Cancer
1170708	ICD9CM	202.13	Mycosis fungoides, intra-abdominal lymph nodes	Cancer
1170709	ICD9CM	202.14	Mycosis fungoides, lymph nodes of axilla and upper limb	Cancer
1170710	ICD9CM	202.15	Mycosis fungoides, lymph nodes of inguinal region and lower limb	Cancer
1170711	ICD9CM	202.16	Mycosis fungoides, intrapelvic lymph nodes	Cancer
1170712	ICD9CM	202.17	Mycosis fungoides, spleen	Cancer
1170713	ICD9CM	202.18	Mycosis fungoides, lymph nodes of multiple sites	Cancer
1170715	ICD9CM	202.2	Sezary's disease, unspecified site, extranodal and solid organ sites	Cancer
1170716	ICD9CM	202.21	Sezary's disease, lymph nodes of head, face, and neck	Cancer
1170717	ICD9CM	202.22	Sezary's disease, intrathoracic lymph nodes	Cancer
1170718	ICD9CM	202.23	Sezary's disease, intra-abdominal lymph nodes	Cancer
1170719	ICD9CM	202.24	Sezary's disease, lymph nodes of axilla and upper limb	Cancer
1170720	ICD9CM	202.25	Sezary's disease, lymph nodes of inguinal region and lower limb	Cancer
1170721	ICD9CM	202.26	Sezary's disease, intrapelvic lymph nodes	Cancer
1170722	ICD9CM	202.27	Sezary's disease, spleen	Cancer
1170723	ICD9CM	202.28	Sezary's disease, lymph nodes of multiple sites	Cancer

1170725	ICD9CM	202.3	Malignant histiocytosis, unspecified site, extranodal and solid organ sites	Cancer
1170726	ICD9CM	202.31	Malignant histiocytosis, lymph nodes of head, face, and neck	Cancer
1170727	ICD9CM	202.32	Malignant histiocytosis, intrathoracic lymph nodes	Cancer
1170728	ICD9CM	202.33	Malignant histiocytosis, intra-abdominal lymph nodes	Cancer
1170729	ICD9CM	202.34	Malignant histiocytosis, lymph nodes of axilla and upper limb	Cancer
1170730	ICD9CM	202.35	Malignant histiocytosis, lymph nodes of inguinal region and lower limb	Cancer
1170731	ICD9CM	202.36	Malignant histiocytosis, intrapelvic lymph nodes	Cancer
1170732	ICD9CM	202.37	Malignant histiocytosis, spleen	Cancer
1170733	ICD9CM	202.38	Malignant histiocytosis, lymph nodes of multiple sites	Cancer
1170735	ICD9CM	202.4	Leukemic reticuloendotheliosis, unspecified site, extranodal and solid organ sites	Cancer
1170736	ICD9CM	202.41	Leukemic reticuloendotheliosis, lymph nodes of head, face, and neck	Cancer
1170737	ICD9CM	202.42	Leukemic reticuloendotheliosis, intrathoracic lymph nodes	Cancer
1170738	ICD9CM	202.43	Leukemic reticuloendotheliosis, intra-abdominal lymph nodes	Cancer
1170739	ICD9CM	202.44	Leukemic reticuloendotheliosis, lymph nodes of axilla and upper arm	Cancer
1170740	ICD9CM	202.45	Leukemic reticuloendotheliosis, lymph nodes of inguinal region and lower limb	Cancer
1170741	ICD9CM	202.46	Leukemic reticuloendotheliosis, intrapelvic lymph nodes	Cancer
1170742	ICD9CM	202.47	Leukemic reticuloendotheliosis, spleen	Cancer
1170743	ICD9CM	202.48	Leukemic reticuloendotheliosis, lymph nodes of multiple sites	Cancer
1170745	ICD9CM	202.5	Letterer-siwe disease, unspecified site, extranodal and solid organ sites	Cancer
1170746	ICD9CM	202.51	Letterer-siwe disease, lymph nodes of head, face, and neck	Cancer
1170747	ICD9CM	202.52	Letterer-siwe disease, intrathoracic lymph nodes	Cancer
1170748	ICD9CM	202.53	Letterer-siwe disease, intra-abdominal lymph nodes	Cancer

1170749	ICD9CM	202.54	Letterer-siwe disease, lymph nodes of axilla and upper limb	Cancer
1170750	ICD9CM	202.55	Letterer-siwe disease, lymph nodes of inguinal region and lower limb	Cancer
1170751	ICD9CM	202.56	Letterer-siwe disease, intrapelvic lymph nodes	Cancer
1170752	ICD9CM	202.57	Letterer-siwe disease, spleen	Cancer
1170753	ICD9CM	202.58	Letterer-siwe disease, lymph nodes of multiple sites	Cancer
1170755	ICD9CM	202.6	Malignant mast cell tumors, unspecified site, extranodal and solid organ sites	Cancer
1170756	ICD9CM	202.61	Malignant mast cell tumors, lymph nodes of head, face, and neck	Cancer
1170757	ICD9CM	202.62	Malignant mast cell tumors, intrathoracic lymph nodes	Cancer
1170758	ICD9CM	202.63	Malignant mast cell tumors, intra-abdominal lymph nodes	Cancer
1170759	ICD9CM	202.64	Malignant mast cell tumors, lymph nodes of axilla and upper limb	Cancer
1170760	ICD9CM	202.65	Malignant mast cell tumors, lymph nodes of inguinal region and lower limb	Cancer
1170761	ICD9CM	202.66	Malignant mast cell tumors, intrapelvic lymph nodes	Cancer
1170762	ICD9CM	202.67	Malignant mast cell tumors, spleen	Cancer
1170763	ICD9CM	202.68	Malignant mast cell tumors, lymph nodes of multiple sites	Cancer
1170765	ICD9CM	202.7	Peripheral T cell lymphoma, unspecified site, extranodal and solid organ sites	Cancer
1170766	ICD9CM	202.71	Peripheral T cell lymphoma, lymph nodes of head, face, and neck	Cancer
1170767	ICD9CM	202.72	Peripheral T cell lymphoma, intrathoracic lymph nodes	Cancer
1170768	ICD9CM	202.73	Peripheral T cell lymphoma, intra-abdominal lymph nodes	Cancer
1170769	ICD9CM	202.74	Peripheral T cell lymphoma, lymph nodes of axilla and upper limb	Cancer
1170770	ICD9CM	202.75	Peripheral T cell lymphoma, lymph nodes of inguinal region and lower limb	Cancer
1170771	ICD9CM	202.76	Peripheral T cell lymphoma, intrapelvic lymph nodes	Cancer

1170772	ICD9CM	202.77	Peripheral T cell lymphoma, spleen	Cancer
1170773	ICD9CM	202.78	Peripheral T cell lymphoma, lymph nodes of multiple sites	Cancer
1170775	ICD9CM	202.8	Other malignant lymphomas, unspecified site, extranodal and solid organ sites	Cancer
1170776	ICD9CM	202.81	Other malignant lymphomas, lymph nodes of head, face, and neck	Cancer
1170777	ICD9CM	202.82	Other malignant lymphomas, intrathoracic lymph nodes	Cancer
1170778	ICD9CM	202.83	Other malignant lymphomas, intra-abdominal lymph nodes	Cancer
1170779	ICD9CM	202.84	Other malignant lymphomas, lymph nodes of axilla and upper limb	Cancer
1170780	ICD9CM	202.85	Other malignant lymphomas, lymph nodes of inguinal region and lower limb	Cancer
1170781	ICD9CM	202.86	Other malignant lymphomas, intrapelvic lymph nodes	Cancer
1170782	ICD9CM	202.87	Other malignant lymphomas, spleen	Cancer
1170783	ICD9CM	202.88	Other malignant lymphomas, lymph nodes of multiple sites	Cancer
1170785	ICD9CM	202.9	Other and unspecified malignant neoplasms of lymphoid and histiocytic tissue, unspecified site, extranodal and solid organ sites	Cancer
1170786	ICD9CM	202.91	Other and unspecified malignant neoplasms of lymphoid and histiocytic tissue, lymph nodes of head, face, and neck	Cancer
1170787	ICD9CM	202.92	Other and unspecified malignant neoplasms of lymphoid and histiocytic tissue, intrathoracic lymph nodes	Cancer
1170788	ICD9CM	202.93	Other and unspecified malignant neoplasms of lymphoid and histiocytic tissue, intra-abdominal lymph nodes	Cancer
1170789	ICD9CM	202.94	Other and unspecified malignant neoplasms of lymphoid and histiocytic tissue, lymph nodes of axilla and upper limb	Cancer
1170790	ICD9CM	202.95	Other and unspecified malignant neoplasms of lymphoid and histiocytic tissue, lymph nodes of inguinal region and lower limb	Cancer

1170791	ICD9CM	202.96	Other and unspecified malignant neoplasms of lymphoid and histiocytic tissue, intrapelvic lymph nodes	Cancer
1170792	ICD9CM	202.97	Other and unspecified malignant neoplasms of lymphoid and histiocytic tissue, spleen	Cancer
1170793	ICD9CM	202.98	Other and unspecified malignant neoplasms of lymphoid and histiocytic tissue, lymph nodes of multiple sites	Cancer
1170796	ICD9CM	203	Multiple myeloma, without mention of having achieved remission	Cancer
1170797	ICD9CM	203.01	Multiple myeloma, in remission	Cancer
1170798	ICD9CM	203.02	Multiple myeloma, in relapse	Cancer
1170800	ICD9CM	203.1	Plasma cell leukemia, without mention of having achieved remission	Cancer
1170801	ICD9CM	203.11	Plasma cell leukemia, in remission	Cancer
1170802	ICD9CM	203.12	Plasma cell leukemia, in relapse	Cancer
1170804	ICD9CM	203.8	Other immunoproliferative neoplasms, without mention of having achieved remission	Cancer
1170805	ICD9CM	203.81	Other immunoproliferative neoplasms, in remission	Cancer
1170806	ICD9CM	203.82	Other immunoproliferative neoplasms, in relapse	Cancer
1170809	ICD9CM	204	Acute lymphoid leukemia, without mention of having achieved remission	Cancer
1170810	ICD9CM	204.01	Acute lymphoid leukemia, in remission	Cancer
1170813	ICD9CM	204.1	Chronic lymphoid leukemia, without mention of having achieved remission	Cancer
1170814	ICD9CM	204.11	Chronic lymphoid leukemia, in remission	Cancer
1170817	ICD9CM	204.2	Subacute lymphoid leukemia, without mention of having achieved remission	Cancer
1170818	ICD9CM	204.21	Subacute lymphoid leukemia, in remission	Cancer
1170821	ICD9CM	204.8	Other lymphoid leukemia, without mention of having achieved remission	Cancer
1170822	ICD9CM	204.81	Other lymphoid leukemia, in remission	Cancer
1170825	ICD9CM	204.9	Unspecified lymphoid leukemia, without mention of having achieved remission	Cancer

1170826	ICD9CM	204.91	Unspecified lymphoid leukemia, in remission	Cancer
1170830	ICD9CM	205	Acute myeloid leukemia, without mention of having achieved remission	Cancer
1170831	ICD9CM	205.01	Acute myeloid leukemia, in remission	Cancer
1170834	ICD9CM	205.1	Chronic myeloid leukemia, without mention of having achieved remission	Cancer
1170835	ICD9CM	205.11	Chronic myeloid leukemia, in remission	Cancer
1170838	ICD9CM	205.2	Subacute myeloid leukemia, without mention of having achieved remission	Cancer
1170839	ICD9CM	205.21	Subacute myeloid leukemia,in remission	Cancer
1170842	ICD9CM	205.3	Myeloid sarcoma, without mention of having achieved remission	Cancer
1170843	ICD9CM	205.31	Myeloid sarcoma, in remission	Cancer
1170846	ICD9CM	205.8	Other myeloid leukemia, without mention of having achieved remission	Cancer
1170847	ICD9CM	205.81	Other myeloid leukemia, in remission	Cancer
1170850	ICD9CM	205.9	Unspecified myeloid leukemia, without mention of having achieved remission	Cancer
1170851	ICD9CM	205.91	Unspecified myeloid leukemia, in remission	Cancer
1170855	ICD9CM	206	Acute monocytic leukemia, without mention of having achieved remission	Cancer
1170856	ICD9CM	206.01	Acute monocytic leukemia,in remission	Cancer
1170859	ICD9CM	206.1	Chronic monocytic leukemia, without mention of having achieved remission	Cancer
1170860	ICD9CM	206.11	Chronic monocytic leukemia, in remission	Cancer
1170863	ICD9CM	206.2	Subacute monocytic leukemia, without mention of having achieved remission	Cancer
1170864	ICD9CM	206.21	Subacute monocytic leukemia, in remission	Cancer
1170867	ICD9CM	206.8	Other monocytic leukemia, without mention of having achieved remission	Cancer
1170868	ICD9CM	206.81	Other monocytic leukemia, in remission	Cancer

1170871	ICD9CM	206.9	Unspecified monocytic leukemia, without mention of having achieved remission	Cancer
1170872	ICD9CM	206.91	Unspecified monocytic leukemia, in remission	Cancer
1170876	ICD9CM	207	Acute erythremia and erythroleukemia, without mention of having achieved remission	Cancer
1170877	ICD9CM	207.01	Acute erythremia and erythroleukemia, in remission	Cancer
1170880	ICD9CM	207.1	Chronic erythremia, without mention of having achieved remission	Cancer
1170881	ICD9CM	207.11	Chronic erythremia, in remission	Cancer
1170884	ICD9CM	207.2	Megakaryocytic leukemia, without mention of having achieved remission	Cancer
1170885	ICD9CM	207.21	Megakaryocytic leukemia, in remission	Cancer
1170888	ICD9CM	207.8	Other specified leukemia, without mention of having achieved remission	Cancer
1170889	ICD9CM	207.81	Other specified leukemia, in remission	Cancer
1170893	ICD9CM	208	Acute leukemia of unspecified cell type, without mention of having achieved remission	Cancer
1170894	ICD9CM	208.01	Acute leukemia of unspecified cell type, in remission	Cancer
1170897	ICD9CM	208.1	Chronic leukemia of unspecified cell type, without mention of having achieved remission	Cancer
1170898	ICD9CM	208.11	Chronic leukemia of unspecified cell type, in remission	Cancer
1170901	ICD9CM	208.2	Subacute leukemia of unspecified cell type, without mention of having achieved remission	Cancer
1170902	ICD9CM	208.21	Subacute leukemia of unspecified cell type, in remission	Cancer
1170905	ICD9CM	208.8	Other leukemia of unspecified cell type, without mention of having achieved remission	Cancer
1170906	ICD9CM	208.81	Other leukemia of unspecified cell type, in remission	Cancer
1170909	ICD9CM	208.9	Unspecified leukemia, without mention of having achieved remission	Cancer

121	SNOMED CT	223003	Tumor of body of uterus affecting pregnancy	Cancer
204	SNOMED CT	308006	Pearly penile papules	Cancer
3816	SNOMED CT	4135001	11p partial monosomy syndrome	Cancer
5870	SNOMED CT	6331000	Angiokeratoma of Fordyce	Cancer
6522	SNOMED CT	7021009	Gastrointestinal eosinophilic granuloma	Cancer
7530	SNOMED CT	8090002	Eosinophilic granuloma of oral mucosa	Cancer
12224	SNOMED CT	13048006	Orbital lymphoma	Cancer
119878	SNOMED CT	134312002	Odontogenic ghost cell carcinoma	Cancer
289752	SNOMED CT	310599006	Malignant neoplasm of canthus	Cancer
291120	SNOMED CT	312111009	Carcinoma of ascending colon	Cancer
291121	SNOMED CT	312112002	Carcinoma of transverse colon	Cancer
291122	SNOMED CT	312113007	Carcinoma of descending colon	Cancer
291123	SNOMED CT	312114001	Carcinoma of hepatic flexure	Cancer
291124	SNOMED CT	312115000	Carcinoma of splenic flexure	Cancer
291939	SNOMED CT	312949007	Retinal pigment epithelial adenocarcinoma	Cancer
292226	SNOMED CT	313248004	Malignant melanoma of chest wall	Cancer
292227	SNOMED CT	313249007	Malignant neoplasm of upper eyelid	Cancer
292328	SNOMED CT	313353007	Squamous cell carcinoma of bronchus in left lower lobe	Cancer
292329	SNOMED CT	313354001	Squamous cell carcinoma of bronchus in left upper lobe	Cancer
292330	SNOMED CT	313355000	Squamous cell carcinoma of bronchus in right lower lobe	Cancer
292331	SNOMED CT	313356004	Squamous cell carcinoma of bronchus in right middle lobe	Cancer
292332	SNOMED CT	313357008	Squamous cell carcinoma of bronchus in right upper lobe	Cancer
292386	SNOMED CT	313428008	Seminoma of undescended testis	Cancer
292387	SNOMED CT	313429000	Seminoma of descended testis	Cancer
293029	SNOMED CT	314191009	Cystadenocarcinoma of ovary	Cancer
293241	SNOMED CT	314408000	Leukemic infiltrate of choroid	Cancer

293251	SNOMED CT	314418005	Leukemic infiltrate of retina	Cancer
293252	SNOMED CT	314419002	Bilateral diffuse uveal melanocytic proliferation	Cancer
293734	SNOMED CT	314947008	Carcinoid tumor of ampulla of Vater	Cancer
293738	SNOMED CT	314951005	Local recurrence of malignant tumor of tongue	Cancer
293739	SNOMED CT	314952003	Local recurrence of malignant tumor of buccal cavity	Cancer
293740	SNOMED CT	314953008	Local recurrence of malignant tumor of thyroid gland	Cancer
293741	SNOMED CT	314954002	Local recurrence of malignant tumor of lung	Cancer
293747	SNOMED CT	314960002	Local recurrence of malignant tumor of esophagus	Cancer
293748	SNOMED CT	314961003	Local recurrence of malignant tumor of stomach	Cancer
293752	SNOMED CT	314965007	Local recurrence of malignant tumor of colon	Cancer
293754	SNOMED CT	314967004	Local recurrence of malignant tumor of kidney	Cancer
293755	SNOMED CT	314968009	Local recurrence of malignant tumor of urinary bladder	Cancer
293756	SNOMED CT	314969001	Local recurrence of malignant tumor of prostate	Cancer
293757	SNOMED CT	314970000	Local recurrence of malignant tumor of cervix	Cancer
293760	SNOMED CT	314973003	Local recurrence of malignant tumor of bone	Cancer
293763	SNOMED CT	314976006	Local recurrence of malignant melanoma of skin	Cancer
303057	SNOMED CT	359782004	Metastatic malignant neoplasm to apex of urinary bladder	Cancer
303060	SNOMED CT	359785002	Metastatic malignant neoplasm to dome of urinary bladder	Cancer
303251	SNOMED CT	359987004	Krukenberg tumor	Cancer
306576	SNOMED CT	363348004	Malignant tumor of lip	Cancer
306577	SNOMED CT	363349007	Malignant tumor of stomach	Cancer
306580	SNOMED CT	363352004	Malignant tumor of anal canal	Cancer
306582	SNOMED CT	363354003	Malignant tumor of cervix	Cancer
306583	SNOMED CT	363355002	Malignant tumor of adrenal gland	Cancer
306585	SNOMED CT	363358000	Malignant tumor of lung	Cancer

306586	SNOMED CT	363359008	Malignant tumor of middle ear	Cancer
306587	SNOMED CT	363360003	Malignant tumor of anterior two-thirds of tongue	Cancer
306592	SNOMED CT	363365008	Malignant tumor of soft tissue of thorax	Cancer
306597	SNOMED CT	363370001	Malignant neoplasm of mesentery	Cancer
306599	SNOMED CT	363372009	Malignant tumor of vermilion border of upper lip	Cancer
306600	SNOMED CT	363373004	Malignant tumor of vermilion border of lower lip	Cancer
306601	SNOMED CT	363374005	Malignant tumor of commissure of lip	Cancer
306602	SNOMED CT	363375006	Malignant tumor of tongue	Cancer
306603	SNOMED CT	363376007	Malignant tumor of base of tongue	Cancer
306604	SNOMED CT	363377003	Malignant tumor of lingual tonsil	Cancer
306605	SNOMED CT	363378008	Malignant tumor of major salivary gland	Cancer
306606	SNOMED CT	363379000	Malignant tumor of parotid gland	Cancer
306607	SNOMED CT	363380002	Malignant tumor of submandibular gland	Cancer
306608	SNOMED CT	363381003	Malignant tumor of sublingual gland	Cancer
306609	SNOMED CT	363382005	Malignant tumor of gum	Cancer
306610	SNOMED CT	363383000	Malignant tumor of upper gingiva	Cancer
306611	SNOMED CT	363384006	Malignant tumor of lower gingiva	Cancer
306612	SNOMED CT	363385007	Malignant tumor of floor of mouth	Cancer
306613	SNOMED CT	363386008	Malignant tumor of buccal mucosa	Cancer
306614	SNOMED CT	363387004	Malignant tumor of hard palate	Cancer
306615	SNOMED CT	363388009	Malignant tumor of soft palate	Cancer
306616	SNOMED CT	363389001	Malignant tumor of uvula	Cancer
306617	SNOMED CT	363390005	Malignant tumor of palate	Cancer
306618	SNOMED CT	363391009	Malignant tumor of retromolar area	Cancer
306619	SNOMED CT	363392002	Malignant tumor of oropharynx	Cancer
306620	SNOMED CT	363393007	Malignant tumor of tonsil	Cancer

306621	SNOMED CT	363394001	Malignant tumor of tonsillar fossa	Cancer
306622	SNOMED CT	363395000	Malignant tumor of vallecula	Cancer
306625	SNOMED CT	363398003	Malignant tumor of lateral wall of nasopharynx	Cancer
306626	SNOMED CT	363399006	Malignant tumor of hypopharynx	Cancer
306627	SNOMED CT	363400004	Malignant tumor of postcricoid region	Cancer
306628	SNOMED CT	363401000	Malignant tumor of pyriform fossa	Cancer
306629	SNOMED CT	363402007	Malignant tumor of esophagus	Cancer
306630	SNOMED CT	363403002	Malignant tumor of duodenum	Cancer
306633	SNOMED CT	363406005	Malignant tumor of colon	Cancer
306634	SNOMED CT	363407001	Malignant tumor of hepatic flexure	Cancer
306635	SNOMED CT	363408006	Malignant tumor of transverse colon	Cancer
306636	SNOMED CT	363409003	Malignant tumor of descending colon	Cancer
306637	SNOMED CT	363410008	Malignant tumor of sigmoid colon	Cancer
306639	SNOMED CT	363412000	Malignant tumor of ascending colon	Cancer
306640	SNOMED CT	363413005	Malignant tumor of splenic flexure	Cancer
306641	SNOMED CT	363414004	Malignant tumor of rectosigmoid junction	Cancer
306644	SNOMED CT	363417006	Malignant tumor of ampulla of Vater	Cancer
306648	SNOMED CT	363421004	Malignant neoplasm of omentum	Cancer
306649	SNOMED CT	363422006	Malignant tumor of nasal cavity	Cancer
306650	SNOMED CT	363423001	Malignant tumor of nasal septum	Cancer
306651	SNOMED CT	363424007	Malignant tumor of mastoid air cells	Cancer
306652	SNOMED CT	363425008	Malignant tumor of maxillary sinus	Cancer
306653	SNOMED CT	363426009	Malignant tumor of ethmoid sinus	Cancer
306654	SNOMED CT	363427000	Malignant tumor of frontal sinus	Cancer
306655	SNOMED CT	363428005	Malignant tumor of sphenoid sinus	Cancer
306656	SNOMED CT	363429002	Malignant tumor of larynx	Cancer

306657	SNOMED CT	363430007	Malignant tumor of subglottis	Cancer
306658	SNOMED CT	363431006	Malignant tumor of laryngeal cartilage	Cancer
306659	SNOMED CT	363432004	Malignant tumor of trachea	Cancer
306660	SNOMED CT	363433009	Malignant tumor of pleura	Cancer
306661	SNOMED CT	363434003	Malignant tumor of thymus	Cancer
306662	SNOMED CT	363435002	Malignant tumor of heart	Cancer
306663	SNOMED CT	363436001	Malignant tumor of endocardium	Cancer
306664	SNOMED CT	363437005	Malignant tumor of myocardium	Cancer
306665	SNOMED CT	363438000	Malignant neoplasm of vertebral column	Cancer
306670	SNOMED CT	363443007	Malignant tumor of ovary	Cancer
306671	SNOMED CT	363444001	Malignant tumor of fallopian tube	Cancer
306672	SNOMED CT	363445000	Malignant tumor of vagina	Cancer
306673	SNOMED CT	363446004	Malignant neoplasm of labia majora	Cancer
306674	SNOMED CT	363447008	Malignant neoplasm of labia minora	Cancer
306676	SNOMED CT	363449006	Malignant tumor of testis	Cancer
306678	SNOMED CT	363451005	Malignant tumor of glans penis	Cancer
306679	SNOMED CT	363452003	Malignant tumor of epididymis	Cancer
306680	SNOMED CT	363453008	Malignant tumor of spermatic cord	Cancer
306681	SNOMED CT	363454002	Malignant tumor of scrotum	Cancer
306683	SNOMED CT	363456000	Malignant tumor of urachus	Cancer
306684	SNOMED CT	363457009	Malignant tumor of renal pelvis	Cancer
306685	SNOMED CT	363458004	Malignant tumor of ureter	Cancer
306686	SNOMED CT	363459007	Malignant tumor of urethra	Cancer
306687	SNOMED CT	363460002	Malignant tumor of paraurethral gland	Cancer
306688	SNOMED CT	363461003	Malignant neoplasm of eye	Cancer
306689	SNOMED CT	363462005	Malignant tumor of orbit	Cancer

306690	SNOMED CT	363463000	Malignant tumor of conjunctiva	Cancer
306691	SNOMED CT	363464006	Malignant tumor of cornea	Cancer
306692	SNOMED CT	363465007	Malignant tumor of retina	Cancer
306693	SNOMED CT	363466008	Malignant tumor of choroid	Cancer
306694	SNOMED CT	363467004	Malignant neoplasm of frontal lobe	Cancer
306695	SNOMED CT	363468009	Malignant neoplasm of temporal lobe	Cancer
306696	SNOMED CT	363469001	Malignant neoplasm of parietal lobe	Cancer
306697	SNOMED CT	363470000	Malignant neoplasm of occipital lobe	Cancer
306698	SNOMED CT	363471001	Malignant neoplasm of cerebral ventricles	Cancer
306700	SNOMED CT	363473003	Malignant neoplasm of brainstem	Cancer
306701	SNOMED CT	363474009	Malignant neoplasm of cerebral meninges	Cancer
306702	SNOMED CT	363475005	Malignant tumor of spinal cord	Cancer
306703	SNOMED CT	363476006	Malignant neoplasm of spinal meninges	Cancer
306705	SNOMED CT	363478007	Malignant tumor of thyroid gland	Cancer
306708	SNOMED CT	363481002	Malignant tumor of parathyroid gland	Cancer
306709	SNOMED CT	363482009	Malignant tumor of pituitary gland	Cancer
306710	SNOMED CT	363483004	Malignant tumor of pineal gland	Cancer
306712	SNOMED CT	363485006	Malignant tumor of minor salivary gland	Cancer
306713	SNOMED CT	363486007	Malignant tumor of vocal cord	Cancer
306714	SNOMED CT	363487003	Malignant tumor of aryepiglottic fold - laryngeal aspect	Cancer
306715	SNOMED CT	363488008	Malignant tumor of false cord	Cancer
306717	SNOMED CT	363490009	Malignant tumor of anus	Cancer
306718	SNOMED CT	363491008	Malignant tumor of cloacogenic zone	Cancer
306719	SNOMED CT	363492001	Malignant tumor of peritoneum	Cancer
306720	SNOMED CT	363493006	Malignant tumor of bronchus	Cancer
306721	SNOMED CT	363494000	Malignant tumor of mediastinum	Cancer

306724	SNOMED CT	363497007	Malignant tumor of meninges	Cancer
306725	SNOMED CT	363498002	Malignant tumor of optic nerve	Cancer
306729	SNOMED CT	363502009	Malignant tumor of axilla	Cancer
306733	SNOMED CT	363506007	Malignant tumor of nasal sinuses	Cancer
306734	SNOMED CT	363507003	Malignant tumor of pharynx	Cancer
306740	SNOMED CT	363514001	Malignant tumor of female genital organ	Cancer
306741	SNOMED CT	363515000	Malignant tumor of male genital organ	Cancer
306742	SNOMED CT	363516004	Malignant tumor of penis	Cancer
306743	SNOMED CT	363517008	Malignant tumor of urinary tract proper	Cancer
306744	SNOMED CT	363518003	Malignant tumor of kidney	Cancer
312599	SNOMED CT	369463005	Malignant tumor involving ureter by direct extension from bladder	Cancer
312600	SNOMED CT	369464004	Malignant tumor involving ureter by separate metastasis from bladder	Cancer
312601	SNOMED CT	369465003	Malignant tumor involving urethra by direct extension from bladder	Cancer
312602	SNOMED CT	369466002	Malignant tumor involving urethra by direct extension from prostate	Cancer
312603	SNOMED CT	369467006	Malignant tumor involving urethra by separate metastasis from bladder	Cancer
312604	SNOMED CT	369468001	Malignant tumor involving urethra by separate metastasis from prostate	Cancer
312605	SNOMED CT	369469009	Malignant tumor involving bladder by direct extension from endometrium	Cancer
312606	SNOMED CT	369470005	Malignant tumor involving bladder by direct extension from fallopian tube	Cancer
312607	SNOMED CT	369471009	Malignant tumor involving bladder by direct extension from ovary	Cancer
312608	SNOMED CT	369472002	Malignant tumor involving bladder by direct extension from prostate	Cancer
312611	SNOMED CT	369475000	Malignant tumor involving bladder by direct extension from vagina	Cancer

312612	SNOMED CT	369476004	Malignant tumor involving bladder by separate metastasis from endometrium	Cancer
312613	SNOMED CT	369477008	Malignant tumor involving bladder by separate metastasis from fallopian tube	Cancer
312614	SNOMED CT	369478003	Malignant tumor involving bladder by separate metastasis from ovary	Cancer
312615	SNOMED CT	369479006	Malignant tumor involving bladder by separate metastasis from prostate	Cancer
312616	SNOMED CT	369480009	Malignant tumor involving bladder by separate metastasis from uterine cervix	Cancer
312617	SNOMED CT	369481008	Malignant tumor involving bladder by separate metastasis from uterus	Cancer
312618	SNOMED CT	369482001	Malignant tumor involving bladder by separate metastasis from vagina	Cancer
312620	SNOMED CT	369484000	Malignant tumor involving vasa deferentia by separate metastasis from prostate	Cancer
312621	SNOMED CT	369485004	Malignant tumor involving prostate by direct extension from bladder	Cancer
312622	SNOMED CT	369486003	Malignant tumor involving prostate by separate metastasis from bladder	Cancer
312623	SNOMED CT	369487007	Primary malignant neoplasm of seminal vesicle	Cancer
312625	SNOMED CT	369489005	Malignant tumor involving seminal vesicle by direct extension from bladder	Cancer
312626	SNOMED CT	369490001	Malignant tumor involving seminal vesicle by direct extension from prostate	Cancer
312627	SNOMED CT	369491002	Malignant tumor involving seminal vesicle by separate metastasis from bladder	Cancer
312628	SNOMED CT	369492009	Malignant tumor involving seminal vesicle by separate metastasis from prostate	Cancer
312629	SNOMED CT	369493004	Malignant tumor involving uterine corpus by direct extension from bladder	Cancer
312630	SNOMED CT	369494005	Malignant tumor involving uterine corpus by direct extension from ovary	Cancer

312631	SNOMED CT	369495006	Malignant tumor involving uterine corpus by direct extension from uterine cervix	Cancer
312632	SNOMED CT	369496007	Malignant tumor involving uterine corpus by direct extension from vagina	Cancer
312633	SNOMED CT	369497003	Malignant tumor involving uterine cervix by direct extension from fallopian tube	Cancer
312634	SNOMED CT	369498008	Malignant tumor involving uterine cervix by direct extension from ovary	Cancer
312635	SNOMED CT	369499000	Malignant tumor involving uterine cervix by direct extension from vagina	Cancer
312636	SNOMED CT	369500009	Malignant tumor involving uterine cervix by separate metastasis from fallopian tube	Cancer
312637	SNOMED CT	369501008	Malignant tumor involving uterine cervix by separate metastasis from ovary	Cancer
312638	SNOMED CT	369502001	Malignant tumor involving uterine corpus by separate metastasis from bladder	Cancer
312639	SNOMED CT	369503006	Malignant tumor involving vagina by direct extension from uterus	Cancer
312640	SNOMED CT	369504000	Malignant tumor involving vagina by direct extension from bladder	Cancer
312641	SNOMED CT	369505004	Malignant tumor involving vagina by direct extension from endometrium	Cancer
312642	SNOMED CT	369506003	Malignant tumor involving vagina by direct extension from fallopian tube	Cancer
312648	SNOMED CT	369512008	Malignant tumor involving vagina by direct extension from ovary	Cancer
312649	SNOMED CT	369513003	Primary malignant neoplasm of left fallopian tube	Cancer
312651	SNOMED CT	369515005	Malignant tumor involving left fallopian tube by direct extension from endometrium	Cancer
312652	SNOMED CT	369516006	Malignant tumor involving left fallopian tube by direct extension from ovary	Cancer
312653	SNOMED CT	369517002	Malignant tumor involving left fallopian tube by direct extension from right fallopian tube	Cancer

312654	SNOMED CT	369518007	Malignant tumor involving left fallopian tube by direct extension from uterine cervix	Cancer
312655	SNOMED CT	369519004	Malignant tumor involving left fallopian tube by direct extension from uterus	Cancer
312656	SNOMED CT	369520005	Primary malignant neoplasm of right fallopian tube	Cancer
312658	SNOMED CT	369522002	Primary malignant neoplasm of left ovary	Cancer
312660	SNOMED CT	369524001	Malignant tumor involving left ovary by direct extension from endometrium	Cancer
312661	SNOMED CT	369525000	Malignant tumor involving left ovary by direct extension from fallopian tube	Cancer
312662	SNOMED CT	369526004	Malignant tumor involving left ovary by direct extension from right ovary	Cancer
312663	SNOMED CT	369527008	Malignant tumor involving left ovary by direct extension from uterine cervix	Cancer
312664	SNOMED CT	369528003	Malignant tumor involving left ovary by direct extension from uterus	Cancer
312665	SNOMED CT	369529006	Primary malignant neoplasm of right ovary	Cancer
312667	SNOMED CT	369531002	Malignant tumor involving right ovary by direct extension from endometrium	Cancer
312668	SNOMED CT	369532009	Malignant tumor involving right ovary by direct extension from fallopian tube	Cancer
312669	SNOMED CT	369533004	Malignant tumor involving right ovary by direct extension from left ovary	Cancer
312670	SNOMED CT	369534005	Malignant tumor involving right ovary by direct extension from uterine cervix	Cancer
312673	SNOMED CT	369537003	Malignant tumor involving left broad ligament by direct extension from ovary	Cancer
312674	SNOMED CT	369538008	Malignant tumor involving left broad ligament by metastasis from ovary	Cancer
312675	SNOMED CT	369539000	Malignant tumor involving right broad ligament by direct extension from ovary	Cancer
312676	SNOMED CT	369540003	Malignant tumor involving right broad ligament by metastasis from ovary	Cancer

312677	SNOMED CT	369541004	Malignant tumor involving left fallopian tube by direct extension from vagina	Cancer
312683	SNOMED CT	369547000	Malignant tumor involving right fallopian tube by direct extension from endometrium	Cancer
312684	SNOMED CT	369548005	Malignant tumor involving right fallopian tube by direct extension from left fallopian tube	Cancer
312685	SNOMED CT	369549002	Malignant tumor involving right fallopian tube by direct extension from ovary	Cancer
312686	SNOMED CT	369550002	Malignant tumor involving right fallopian tube by direct extension from uterine cervix	Cancer
312687	SNOMED CT	369551003	Malignant tumor involving right fallopian tube by direct extension from uterus	Cancer
312688	SNOMED CT	369552005	Malignant tumor involving right fallopian tube by direct extension from vagina	Cancer
312695	SNOMED CT	369559001	Malignant tumor involving left ovary by direct extension from vagina	Cancer
312702	SNOMED CT	369566000	Malignant tumor involving right ovary by direct extension from uterus	Cancer
312703	SNOMED CT	369567009	Malignant tumor involving right ovary by direct extension from vagina	Cancer
312710	SNOMED CT	369574004	Malignant tumor involving uterine cervix by separate metastasis from vagina	Cancer
312711	SNOMED CT	369575003	Malignant tumor involving uterine corpus by separate metastasis from fallopian tube	Cancer
312712	SNOMED CT	369576002	Malignant tumor involving uterine corpus by separate metastasis from ovary	Cancer
312713	SNOMED CT	369577006	Malignant tumor involving uterine corpus by separate metastasis from uterine cervix	Cancer
312714	SNOMED CT	369578001	Malignant tumor involving uterine corpus by separate metastasis from vagina	Cancer
312715	SNOMED CT	369579009	Malignant tumor involving uterine corpus by direct extension from fallopian tube	Cancer

312716	SNOMED CT	369580007	Malignant tumor involving vagina by direct extension from uterine cervix	Cancer
313868	SNOMED CT	370967009	Retinoblastoma	Cancer
313887	SNOMED CT	370987005	Anaplastic astrocytoma of spinal cord	Cancer
313911	SNOMED CT	371012000	Acute lymphoblastic leukemia, transitional pre-B-cell	Cancer
314030	SNOMED CT	371134001	Malignant lymphoma, large cell, polymorphous, immunoblastic	Cancer
314759	SNOMED CT	371962007	Primary malignant neoplasm of abdominal esophagus	Cancer
314760	SNOMED CT	371963002	Primary malignant neoplasm of adrenal cortex	Cancer
314761	SNOMED CT	371964008	Malignant neoplasm of adrenal cortex	Cancer
314762	SNOMED CT	371965009	Malignant neoplasm of adrenal medulla	Cancer
314763	SNOMED CT	371966005	Primary malignant neoplasm of adrenal medulla	Cancer
314764	SNOMED CT	371967001	Primary malignant neoplasm of ampulla of Vater	Cancer
314765	SNOMED CT	371968006	Primary malignant neoplasm of anterior two-thirds of tongue	Cancer
314766	SNOMED CT	371969003	Primary malignant neoplasm of apex of urinary bladder	Cancer
314768	SNOMED CT	371971003	Primary malignant neoplasm of body of uterus	Cancer
314769	SNOMED CT	371972005	Malignant neoplasm of body of uterus	Cancer
314770	SNOMED CT	371973000	Malignant neoplasm of uterus	Cancer
314771	SNOMED CT	371974006	Malignant neoplasm of border of tongue	Cancer
314772	SNOMED CT	371975007	Primary malignant neoplasm of border of tongue	Cancer
314773	SNOMED CT	371976008	Primary malignant neoplasm of buccal mucosa	Cancer
314775	SNOMED CT	371978009	Primary malignant neoplasm of cervical esophagus	Cancer
314776	SNOMED CT	371979001	Malignant neoplasm of clitoris	Cancer
314777	SNOMED CT	371980003	Primary malignant neoplasm of clitoris	Cancer
314778	SNOMED CT	371981004	Primary malignant neoplasm of commissure of lip	Cancer
314779	SNOMED CT	371982006	Malignant neoplasm of endocrine gland	Cancer
314780	SNOMED CT	371983001	Primary malignant neoplasm of endocrine gland	Cancer

314781	SNOMED CT	371984007	Primary malignant neoplasm of esophagus	Cancer
314783	SNOMED CT	371986009	Primary malignant neoplasm of eye	Cancer
314784	SNOMED CT	371987000	Primary malignant neoplasm of fallopian tube	Cancer
314785	SNOMED CT	371988005	Primary malignant neoplasm of false vocal cord	Cancer
314786	SNOMED CT	371989002	Primary malignant neoplasm of glans penis	Cancer
314787	SNOMED CT	371990006	Primary malignant neoplasm of gum	Cancer
314788	SNOMED CT	371991005	Primary malignant neoplasm of hard palate	Cancer
314790	SNOMED CT	371993008	Primary malignant neoplasm of lacrimal gland	Cancer
314791	SNOMED CT	371994002	Primary malignant neoplasm of laryngeal aspect of aryepiglottic fold	Cancer
314792	SNOMED CT	371995001	Primary malignant neoplasm of larynx	Cancer
314793	SNOMED CT	371996000	Primary malignant neoplasm of lip	Cancer
314794	SNOMED CT	371997009	Primary malignant neoplasm of lower gum	Cancer
314795	SNOMED CT	371998004	Primary malignant neoplasm of lower third of esophagus	Cancer
314796	SNOMED CT	371999007	Primary malignant neoplasm of middle third of esophagus	Cancer
314797	SNOMED CT	372000001	Primary malignant neoplasm of minor salivary gland	Cancer
314798	SNOMED CT	372001002	Primary malignant neoplasm of oral cavity	Cancer
314799	SNOMED CT	372002009	Primary malignant neoplasm of palate	Cancer
314801	SNOMED CT	372004005	Primary malignant neoplasm of parotid gland	Cancer
314802	SNOMED CT	372005006	Primary malignant neoplasm of penis	Cancer
314803	SNOMED CT	372006007	Primary malignant neoplasm of prepuce	Cancer
314805	SNOMED CT	372008008	Primary malignant neoplasm of scaphoid bone	Cancer
314806	SNOMED CT	372009000	Primary malignant neoplasm of scrotum	Cancer
314810	SNOMED CT	372013007	Primary malignant neoplasm of spermatic cord	Cancer
314811	SNOMED CT	372014001	Primary malignant neoplasm of stomach	Cancer
314812	SNOMED CT	372015000	Primary malignant neoplasm of the mesentery	Cancer

314813	SNOMED CT	372016004	Primary malignant neoplasm of the peritoneum	Cancer
314814	SNOMED CT	372017008	Primary malignant neoplasm of thoracic esophagus	Cancer
314815	SNOMED CT	372018003	Malignant neoplasm of thoracic vertebral column	Cancer
314816	SNOMED CT	372019006	Primary malignant neoplasm of thoracic vertebral column	Cancer
314817	SNOMED CT	372020000	Primary malignant neoplasm of tonsil	Cancer
314818	SNOMED CT	372021001	Primary malignant neoplasm of trapezium	Cancer
314819	SNOMED CT	372022008	Primary malignant neoplasm of upper gum	Cancer
314821	SNOMED CT	372024009	Primary malignant neoplasm of uterine cervix	Cancer
314822	SNOMED CT	372025005	Primary malignant neoplasm of vagina	Cancer
314823	SNOMED CT	372026006	Primary malignant neoplasm of vermilion border of lower lip	Cancer
314824	SNOMED CT	372027002	Primary malignant neoplasm of vermilion border of upper lip	Cancer
314825	SNOMED CT	372028007	Primary malignant neoplasm of vertebral column	Cancer
314827	SNOMED CT	372030009	Primary malignant neoplasm of vocal cord	Cancer
314859	SNOMED CT	372062007	Malignant neoplasm of central nervous system	Cancer
314861	SNOMED CT	372064008	Malignant neoplasm of female breast	Cancer
314862	SNOMED CT	372065009	Malignant neoplasm of main bronchus	Cancer
314894	SNOMED CT	372097009	Malignant neoplasm of endocervix	Cancer
314895	SNOMED CT	372098004	Carcinoma of endocervix	Cancer
314896	SNOMED CT	372099007	Malignant neoplasm of exocervix	Cancer
314897	SNOMED CT	372100004	Carcinoma of exocervix	Cancer
314900	SNOMED CT	372103002	Carcinoma of glottis	Cancer
314901	SNOMED CT	372104008	Carcinoma of subglottis	Cancer
314902	SNOMED CT	372105009	Carcinoma of supraglottis	Cancer
314903	SNOMED CT	372106005	Carcinoma of penis	Cancer
314904	SNOMED CT	372107001	Primary malignant neoplasm of ribs and/or sternum and/or clavicle	Cancer

314905	SNOMED CT	372108006	Malignant neoplasm of bone of lower limb	Cancer
314907	SNOMED CT	372110008	Primary malignant neoplasm of lower lobe, bronchus or lung	Cancer
314908	SNOMED CT	372111007	Carcinoma of lower lobe, bronchus or lung	Cancer
314912	SNOMED CT	372115003	Primary malignant neoplasm of pelvic bones, sacrum and coccyx	Cancer
314913	SNOMED CT	372116002	Carcinoma of pelvic bones, sacrum and coccyx	Cancer
314917	SNOMED CT	372120003	Carcinoma of main bronchus	Cancer
314918	SNOMED CT	372121004	Carcinoma of ribs and/or sternum and/or clavicle	Cancer
314928	SNOMED CT	372133009	Primary malignant neoplasm of upper limb bones and scapula	Cancer
314933	SNOMED CT	372138000	Carcinoma of esophagus	Cancer
314936	SNOMED CT	372141009	Carcinoma of vocal cord	Cancer
314938	SNOMED CT	372143007	Carcinoma of stomach	Cancer
315825	SNOMED CT	373168002	Reticulosarcoma	Cancer
320249	SNOMED CT	385478001	Adenoma malignum	Cancer
323119	SNOMED CT	388871003	Primary optic nerve sheath meningioma	Cancer
329858	SNOMED CT	396198006	Small cell carcinoma of prostate	Cancer
330636	SNOMED CT	397008008	Aggressive lymphadenopathic mastocytosis with eosinophilia	Cancer
330637	SNOMED CT	397009000	Mast cell malignancy	Cancer
330639	SNOMED CT	397011009	Mast cell malignancy of lymph nodes	Cancer
330643	SNOMED CT	397015000	Systemic mastocytosis with associated clonal hematological non-mast cell lineage disease	Cancer
332145	SNOMED CT	398623004	Myelodysplastic syndrome with excess blasts	Cancer
332200	SNOMED CT	398679002	Bowenoid papulosis of penis	Cancer
332287	SNOMED CT	398768004	Queyrat's erythroplasia	Cancer
332350	SNOMED CT	398831006	Bowens disease of penis	Cancer
332584	SNOMED CT	399068003	Malignant tumor of prostate	Cancer
332836	SNOMED CT	399326009	Malignant tumor of urinary bladder	Cancer

332998	SNOMED CT	399490008	Adenocarcinoma of prostate	Cancer
333095	SNOMED CT	399590005	Squamous cell carcinoma of prostate	Cancer
333163	SNOMED CT	399660006	Ring melanoma of ciliary body	Cancer
333436	SNOMED CT	399967006	Neoplasm of uncertain behavior of stomach	Cancer
333470	SNOMED CT	400001003	Primary cutaneous lymphoma	Cancer
333560	SNOMED CT	400092004	Penile intraepithelial neoplasia grade III	Cancer
333586	SNOMED CT	400118002	Bowenoid papulosis of penis (PIN III)	Cancer
333590	SNOMED CT	400122007	Primary cutaneous T-cell lymphoma	Cancer
334735	SNOMED CT	401312001	Anal intraepithelial neoplasia grade II	Cancer
334736	SNOMED CT	401313006	Anal intraepithelial neoplasia grade I	Cancer
335880	SNOMED CT	402494003	Basal cell carcinoma of lower eyelid	Cancer
335881	SNOMED CT	402495002	Basal cell carcinoma of medial canthus	Cancer
335882	SNOMED CT	402496001	Basal cell carcinoma of lateral canthus	Cancer
335889	SNOMED CT	402503002	Basal cell carcinoma of nasal columella	Cancer
335890	SNOMED CT	402504008	Basal cell carcinoma of nasolabial groove	Cancer
335891	SNOMED CT	402505009	Basal cell carcinoma of upper lip	Cancer
335892	SNOMED CT	402506005	Basal cell carcinoma of lower lip	Cancer
335893	SNOMED CT	402507001	Basal cell carcinoma of cheek	Cancer
335901	SNOMED CT	402515003	Basal cell carcinoma of anterior chest	Cancer
336204	SNOMED CT	402819001	Basal cell carcinoma of skin of lip	Cancer
336264	SNOMED CT	402880009	Primary cutaneous large T-cell lymphoma	Cancer
336265	SNOMED CT	402882001	Hodgkin's disease affecting skin	Cancer
336728	SNOMED CT	403467008	Lichen sclerosus-associated penile intraepithelial neoplasia	Cancer
336729	SNOMED CT	403468003	Squamous cell carcinoma of penis	Cancer
336965	SNOMED CT	403714009	PUVA therapy-associated malignant melanoma	Cancer
337120	SNOMED CT	403889000	Verrucous carcinoma of oral cavity	Cancer

337150	SNOMED CT	403919005	Basal cell carcinoma of upper eyelid	Cancer
337157	SNOMED CT	403926005	Malignant melanoma of oral cavity	Cancer
337179	SNOMED CT	403950007	Moll's gland adenocarcinoma	Cancer
337319	SNOMED CT	404090003	Malignant infiltration of oral cavity by underlying tumor	Cancer
337323	SNOMED CT	404094007	Metastasis involving oral cavity	Cancer
337332	SNOMED CT	404103007	Lymphomatoid papulosis type A (CD-30 positive type)	Cancer
337333	SNOMED CT	404104001	Lymphomatoid papulosis type B - mycosis fungoides-like	Cancer
337334	SNOMED CT	404105000	Lymphomatoid papulosis type C (anaplastic large-cell lymphoma-like)	Cancer
337335	SNOMED CT	404107008	Patch/plaque stage mycosis fungoides	Cancer
337336	SNOMED CT	404108003	Poikilodermatous mycosis fungoides	Cancer
337337	SNOMED CT	404109006	Follicular mucinosis type mycosis fungoides	Cancer
337338	SNOMED CT	404110001	Hypomelanotic mycosis fungoides	Cancer
337339	SNOMED CT	404111002	Lymphomatoid papulosis-associated mycosis fungoides	Cancer
337340	SNOMED CT	404112009	Granulomatous mycosis fungoides	Cancer
337341	SNOMED CT	404113004	Tumor stage mycosis fungoides	Cancer
337342	SNOMED CT	404114005	Erythrodermic mycosis fungoides	Cancer
337343	SNOMED CT	404115006	Bullous mycosis fungoides	Cancer
337344	SNOMED CT	404116007	Mycosis fungoides with systemic infiltration	Cancer
337345	SNOMED CT	404117003	Spongiotic mycosis fungoides	Cancer
337346	SNOMED CT	404118008	Syringotropic mycosis fungoides	Cancer
337347	SNOMED CT	404119000	Pagetoid reticulosis	Cancer
337348	SNOMED CT	404120006	Localized pagetoid reticulosis	Cancer
337349	SNOMED CT	404121005	Generalized pagetoid reticulosis	Cancer
337351	SNOMED CT	404126000	CD-30 positive pleomorphic large T-cell cutaneous lymphoma	Cancer
337352	SNOMED CT	404127009	CD-30 positive T-immunoblastic cutaneous lymphoma	Cancer

337353	SNOMED CT	404128004	CD-30 negative cutaneous T-cell lymphoma	Cancer
337354	SNOMED CT	404129007	CD-30 negative anaplastic large T-cell cutaneous lymphoma	Cancer
337355	SNOMED CT	404130002	CD-30 negative pleomorphic large T-cell cutaneous lymphoma	Cancer
337356	SNOMED CT	404131003	CD-30 negative T-immunoblastic cutaneous lymphoma	Cancer
337358	SNOMED CT	404133000	Subcutaneous panniculitis-like T-cell lymphoma	Cancer
337359	SNOMED CT	404134006	Anaplastic large T-cell systemic malignant lymphoma	Cancer
337360	SNOMED CT	404137004	Precursor B-cell lymphoblastic lymphoma involving skin	Cancer
337361	SNOMED CT	404138009	Small lymphocytic B-cell lymphoma involving skin	Cancer
337362	SNOMED CT	404139001	Leukemic infiltration of skin in hairy-cell leukemia	Cancer
337363	SNOMED CT	404140004	Primary cutaneous marginal zone B-cell lymphoma	Cancer
337364	SNOMED CT	404141000	Primary cutaneous immunocytoma	Cancer
337365	SNOMED CT	404142007	Primary cutaneous plasmacytoma	Cancer
337366	SNOMED CT	404144008	Primary cutaneous diffuse large cell B-cell lymphoma	Cancer
337369	SNOMED CT	404147001	Follicular center B-cell lymphoma (nodal/systemic with skin involvement)	Cancer
337370	SNOMED CT	404148006	Diffuse large B-cell lymphoma (nodal/systemic with skin involvement)	Cancer
337371	SNOMED CT	404149003	Lymphoplasmacytic B-cell lymphoma, nodal/systemic with skin involvement	Cancer
337372	SNOMED CT	404150003	Mantle cell B-cell lymphoma (nodal/systemic with skin involvement)	Cancer
337373	SNOMED CT	404151004	Leukemic infiltration of skin in myeloid leukemia	Cancer
337374	SNOMED CT	404152006	Leukemic infiltration of skin in acute myeloid leukemia	Cancer
337375	SNOMED CT	404153001	Leukemic infiltration of skin in chronic myeloid leukemia	Cancer
337376	SNOMED CT	404154007	Leukemic infiltration of skin in monocytic leukemia	Cancer
337377	SNOMED CT	404155008	Granulocytic sarcoma affecting skin	Cancer
337388	SNOMED CT	404169008	Malignant histiocytosis involving skin	Cancer

337391	SNOMED CT	404172001	Mast cell leukemia affecting skin	Cancer
337818	SNOMED CT	404653000	Chiasmal glioma	Cancer
337829	SNOMED CT	404664002	Malignant optic glioma	Cancer
338696	SNOMED CT	405546008	Malignant pericardial effusion	Cancer
338969	SNOMED CT	405822008	Squamous cell carcinoma of larynx	Cancer
339092	SNOMED CT	405945003	Malignant neoplasm of metatarsal bone of foot	Cancer
341546	SNOMED CT	408642003	Transitional cell carcinoma of kidney	Cancer
341548	SNOMED CT	408644002	Adenocarcinoma of duodenum	Cancer
341551	SNOMED CT	408647009	Adenocarcinoma of stomach	Cancer
341552	SNOMED CT	408648004	Squamous cell carcinoma of epiglottis	Cancer
341553	SNOMED CT	408649007	Squamous cell carcinoma of pharynx	Cancer
346033	SNOMED CT	413389003	Accelerated phase chronic myeloid leukemia	Cancer
346078	SNOMED CT	413441006	Acute monocytic leukemia	Cancer
346079	SNOMED CT	413442004	Acute monocytic/monoblastic leukemia	Cancer
346160	SNOMED CT	413537009	Angioimmunoblastic T-cell lymphoma	Cancer
346273	SNOMED CT	413656006	Blastic phase chronic myeloid leukemia	Cancer
346443	SNOMED CT	413842007	Chronic myeloid leukemia in lymphoid blast crisis	Cancer
346444	SNOMED CT	413843002	Chronic myeloid leukemia in myeloid blast crisis	Cancer
346448	SNOMED CT	413847001	Chronic phase chronic myeloid leukemia	Cancer
346815	SNOMED CT	414250001	Fibrous histiocytoma of orbit	Cancer
347152	SNOMED CT	414626005	Lymphangioma of orbit	Cancer
347192	SNOMED CT	414666009	Meningioma of orbit	Cancer
347202	SNOMED CT	414676007	Metastatic neuroblastoma of orbit proper	Cancer
347294	SNOMED CT	414785000	Multiple solitary plasmacytomas	Cancer
347453	SNOMED CT	414950005	Optic nerve glioma of orbit	Cancer
347608	SNOMED CT	415112005	Plasmacytoma	Cancer

347763	SNOMED CT	415283002	Myelodysplastic syndrome with excess blasts-1	Cancer
347764	SNOMED CT	415284008	Myelodysplastic syndrome with excess blasts-2	Cancer
347765	SNOMED CT	415285009	Myelodysplastic syndrome with multilineage dysplasia	Cancer
347766	SNOMED CT	415286005	Myelodysplastic syndrome with ring sideroblasts and multilineage dysplasia	Cancer
347767	SNOMED CT	415287001	Relapsing chronic myeloid leukemia	Cancer
347980	SNOMED CT	415653001	Submucosal tumor of esophagus	Cancer
348598	SNOMED CT	416351002	Intraocular optic nerve glioma	Cancer
348751	SNOMED CT	416510003	Neoplasm of lacrimal system	Cancer
348944	SNOMED CT	416712009	Clear cell (mesonephric) neoplasm of ovary	Cancer
348983	SNOMED CT	416753001	Neoplasm of nasolacrimal duct	Cancer
349065	SNOMED CT	416842003	Malignant sacral teratoma	Cancer
349123	SNOMED CT	416901002	Malignant medulloepithelioma of ciliary body	Cancer
349618	SNOMED CT	417417007	Malignant teratoma of undescended testis	Cancer
349750	SNOMED CT	417554000	Malignant teratoma of descended testis	Cancer
349811	SNOMED CT	417619001	Intracranial optic nerve glioma	Cancer
350238	SNOMED CT	418134006	Conjunctival intraepithelial neoplasia	Cancer
350469	SNOMED CT	418372008	Squamous cell carcinoma of mucous membrane of lower lip	Cancer
351122	SNOMED CT	419052002	Malignant tumor of urinary system	Cancer
351304	SNOMED CT	419240004	Squamous cell carcinoma of mucous membrane of upper lip	Cancer
351378	SNOMED CT	419317000	Neoplasm of uncertain behavior of lacrimal drainage structure	Cancer
351388	SNOMED CT	419327006	Endometrial intraepithelial neoplasia	Cancer
351889	SNOMED CT	419842002	Squamous cell carcinoma of oral mucous membrane	Cancer
351933	SNOMED CT	419889000	Neoplasm of lacrimal drainage system	Cancer
352536	SNOMED CT	420519005	Malignant lymphoma of the eye region	Cancer
352777	SNOMED CT	420788006	Primary intraocular non-Hodgkin malignant lymphoma	Cancer

352820	SNOMED CT	420835009	Corneal intraepithelial neoplasia	Cancer
353192	SNOMED CT	421246008	Precursor T-cell lymphoblastic lymphoma	Cancer
353195	SNOMED CT	421249001	Malignant tumor of vermilion border of lip	Cancer
354369	SNOMED CT	422541001	Undifferentiated carcinoma of nasopharynx	Cancer
354517	SNOMED CT	422691006	Squamous cell carcinoma of nasopharynx	Cancer
354584	SNOMED CT	422758009	Carcinoma of nasal meatus	Cancer
354607	SNOMED CT	422782004	Ovarian cancer, disseminated	Cancer
354656	SNOMED CT	422833009	Adenoid cystic carcinoma of salivary gland	Cancer
354675	SNOMED CT	422853008	Lymphoma of retroperitoneal space	Cancer
354708	SNOMED CT	422886007	Olfactory neuroblastoma	Cancer
354789	SNOMED CT	422968005	Non-small cell carcinoma of lung, TNM stage 3	Cancer
354824	SNOMED CT	423005002	Primary malignant neoplasm of lacrimal gland duct	Cancer
354851	SNOMED CT	423032007	Leukemic infiltration of orbit	Cancer
354857	SNOMED CT	423038006	Polymorphous low grade adenocarcinoma of salivary gland	Cancer
354868	SNOMED CT	423050000	Large cell carcinoma of lung, TNM stage 2	Cancer
354924	SNOMED CT	423106003	Adenocarcinoma of nasopharynx	Cancer
354938	SNOMED CT	423121009	Non-small cell carcinoma of lung, TNM stage 4	Cancer
354975	SNOMED CT	423158009	Hurthle cell carcinoma of thyroid	Cancer
355006	SNOMED CT	423189008	Adenoid cystic carcinoma of submandibular gland	Cancer
355012	SNOMED CT	423195009	Primary malignant neoplasm of lacrimal drainage system	Cancer
355092	SNOMED CT	423278008	Lentigo maligna of skin of eyelid	Cancer
355094	SNOMED CT	423280002	Malignant melanoma of skin of canthus of eye	Cancer
355108	SNOMED CT	423295000	Squamous cell carcinoma of lung, TNM stage 1	Cancer
355237	SNOMED CT	423424005	Mucoepidermoid carcinoma of submandibular gland	Cancer
355238	SNOMED CT	423425006	Malignant neoplasm of skin of eyelid	Cancer

355260	SNOMED CT	423447006	Malignant melanoma of skin of lower eyelid	Cancer
355276	SNOMED CT	423464009	Squamous cell carcinoma of oropharynx	Cancer
355280	SNOMED CT	423468007	Squamous cell carcinoma of lung, TNM stage 2	Cancer
355306	SNOMED CT	423494003	Malignant melanoma of skin of upper eyelid	Cancer
355411	SNOMED CT	423600008	Large cell carcinoma of lung, TNM stage 4	Cancer
355418	SNOMED CT	423607006	Adenocarcinoma of anus	Cancer
355426	SNOMED CT	423615009	Adenoid cystic carcinoma of parotid gland	Cancer
355430	SNOMED CT	423619003	Teratoma of orbit	Cancer
355438	SNOMED CT	423627007	Sarcoma of ovary	Cancer
355484	SNOMED CT	423673009	Malignant melanoma of retina	Cancer
355502	SNOMED CT	423691004	Malignant neoplasm of gum and contiguous sites	Cancer
355519	SNOMED CT	423708008	Mucoepidermoid carcinoma of salivary gland	Cancer
355779	SNOMED CT	423973006	Carcinoma of uterine cervix, invasive	Cancer
355793	SNOMED CT	423987006	Cancer of vulva, disseminated	Cancer
355935	SNOMED CT	424132000	Non-small cell carcinoma of lung, TNM stage 1	Cancer
355953	SNOMED CT	424151006	Anaplastic glioma of brain	Cancer
356058	SNOMED CT	424260006	Squamous cell carcinoma of nasolabial fold	Cancer
356073	SNOMED CT	424276002	Malignant glioma of brainstem	Cancer
356096	SNOMED CT	424302003	Malignant melanoma of skin of lower lip	Cancer
356128	SNOMED CT	424334007	Malignant tumor of spinal cord, intramedullary	Cancer
356200	SNOMED CT	424408000	Keratoacanthoma of eyelid	Cancer
356213	SNOMED CT	424422000	Osteoma of orbit	Cancer
356277	SNOMED CT	424487008	Malignant melanoma of skin of upper lip	Cancer
356338	SNOMED CT	424549003	Malignant tumor of spinal cord, extramedullary	Cancer
356628	SNOMED CT	424849005	Primary sarcoma of tongue	Cancer

356665	SNOMED CT	424887002	Thyroid cancer metastatic to bone	Cancer
356714	SNOMED CT	424938000	Large cell carcinoma of lung, TNM stage 1	Cancer
356746	SNOMED CT	424970000	Large cell carcinoma of lung, TNM stage 3	Cancer
356824	SNOMED CT	425048006	Non-small cell carcinoma of lung, TNM stage 2	Cancer
356842	SNOMED CT	425066001	Carcinoma of urinary bladder, invasive	Cancer
356902	SNOMED CT	425127006	Carcinoma ex pleomorphic adenoma of parotid gland	Cancer
356948	SNOMED CT	425178004	Adenocarcinoma of rectosigmoid junction	Cancer
356994	SNOMED CT	425225007	Malignant mixed tumor of salivary gland	Cancer
356999	SNOMED CT	425230006	Squamous cell carcinoma of lung, TNM stage 3	Cancer
357000	SNOMED CT	425231005	Carcinoma of urinary bladder, superficial	Cancer
357141	SNOMED CT	425376008	Squamous cell carcinoma of lung, TNM stage 4	Cancer
357446	SNOMED CT	425688002	Philadelphia chromosome-positive acute lymphoblastic leukemia	Cancer
357507	SNOMED CT	425749006	Subacute myeloid leukemia in remission	Cancer
357624	SNOMED CT	425869007	Acute promyelocytic leukemia, FAB M3, in remission	Cancer
357691	SNOMED CT	425941003	Precursor B-cell acute lymphoblastic leukemia in remission	Cancer
357814	SNOMED CT	426071002	Hodgkin's disease in remission	Cancer
357867	SNOMED CT	426124006	Acute myeloid leukemia with maturation, FAB M2, in remission	Cancer
357934	SNOMED CT	426191007	Myxoma of heart	Cancer
357960	SNOMED CT	426217000	Aleukaemic leukaemia in remission	Cancer
357991	SNOMED CT	426248008	Aleukemic lymphoid leukemia in remission	Cancer
358077	SNOMED CT	426336007	Solitary osseous myeloma	Cancer
358109	SNOMED CT	426370008	Subacute lymphoid leukemia in remission	Cancer
358371	SNOMED CT	426642002	Erythroleukemia, FAB M6 in remission	Cancer

358609	SNOMED CT	426885008	Hodgkin's disease, lymphocytic depletion of lymph nodes of head	Cancer
358687	SNOMED CT	426964009	Epidermal growth factor receptor positive non-small cell lung cancer	Cancer
358759	SNOMED CT	427038005	Epidermal growth factor receptor negative non-small cell lung cancer	Cancer
358777	SNOMED CT	427056005	Subacute leukemia in remission	Cancer
358862	SNOMED CT	427141003	Malignant lymphoma in remission	Cancer
359207	SNOMED CT	427492003	Hormone refractory prostate cancer	Cancer
359356	SNOMED CT	427642009	T-cell acute lymphoblastic leukemia in remission	Cancer
359372	SNOMED CT	427658007	Acute myelomonocytic leukemia, FAB M4, in remission	Cancer
359733	SNOMED CT	428061005	Malignant neoplasm of brain	Cancer
359769	SNOMED CT	428100006	Malignant neoplasm of thoracic cavity structure	Cancer
359939	SNOMED CT	428281000	Malignant neoplasm of bone	Cancer
360384	SNOMED CT	428753007	Low grade glioma of cerebellum	Cancer
360582	SNOMED CT	428960009	Low grade glioma of brainstem	Cancer
360585	SNOMED CT	428964000	Low grade glioma of cerebrum	Cancer
360586	SNOMED CT	428965004	Low grade glioma of thalamus	Cancer
360649	SNOMED CT	429033009	Malignant neoplasm of cerebrum	Cancer
361007	SNOMED CT	429408002	Low grade glioma of brain	Cancer
361151	SNOMED CT	429565004	Germ cell tumor of the brain	Cancer
361883	SNOMED CT	430338009	Smoldering chronic lymphocytic leukemia	Cancer
362143	SNOMED CT	430621000	Malignant neoplasm of lower respiratory tract	Cancer
364285	SNOMED CT	433067002	Adamantinoma of femur	Cancer
367104	SNOMED CT	438946002	Siewert type I adenocarcinoma of esophagogastric junction	Cancer
367633	SNOMED CT	439478008	Siewert type III adenocarcinoma of esophagogastric junction	Cancer
368320	SNOMED CT	440173001	Nonsquamous nonsmall cell neoplasm of lung	Cancer

368543	SNOMED CT	440397000	Primary osteosarcoma of pelvis	Cancer
368646	SNOMED CT	440501006	Siewert type II adenocarcinoma of esophagogastric junction	Cancer
368670	SNOMED CT	440525003	Primary small cell neoplasm of thymus	Cancer
368672	SNOMED CT	440527006	Primary squamous cell carcinoma of naris	Cancer
369688	SNOMED CT	441559006	Mantle cell lymphoma of spleen	Cancer
370070	SNOMED CT	441962003	Large cell lymphoma of intrapelvic lymph nodes	Cancer
370625	SNOMED CT	442537007	Non-Hodgkin lymphoma associated with Human immunodeficiency virus infection	Cancer
371550	SNOMED CT	443487006	Mantle cell lymphoma	Cancer
372556	SNOMED CT	444545003	Glioma of brainstem	Cancer
372607	SNOMED CT	444597005	Mucosa-associated lymphoid tissue (MALT) lymphoma of stomach	Cancer
372867	SNOMED CT	444869007	Cavernous hemangioma of brain	Cancer
372907	SNOMED CT	444910004	Primary mediastinal (thymic) large B-cell lymphoma	Cancer
372908	SNOMED CT	444911000	Acute myeloid leukemia with t(9:11)(p22;q23); MLLT3-MLL	Cancer
373216	SNOMED CT	445227008	Juvenile myelomonocytic leukemia	Cancer
373391	SNOMED CT	445406001	Hepatosplenic T-cell lymphoma	Cancer
373432	SNOMED CT	445448008	Acute myeloid leukemia with myelodysplasia-related changes	Cancer
373494	SNOMED CT	445513004	Intracranial cavernous hemangioma	Cancer
373693	SNOMED CT	445736006	Gastrointestinal stromal tumor of stomach	Cancer
373978	SNOMED CT	446022000	Carcinoma of uterus	Cancer
374032	SNOMED CT	446076000	Extragastrointestinal stromal tumor of peritoneum	Cancer
374079	SNOMED CT	446124001	Gastrointestinal stromal tumor of esophagus	Cancer
374537	SNOMED CT	446593006	Bizarre parosteal osteochondromatous proliferation	Cancer
374651	SNOMED CT	446710005	Low grade prostatic intraepithelial neoplasia	Cancer

374652	SNOMED CT	446711009	High grade prostatic intraepithelial neoplasia	Cancer
374824	SNOMED CT	446887007	Endometrial stromal tumor	Cancer
375031	SNOMED CT	447100004	Marginal zone lymphoma	Cancer
375188	SNOMED CT	447266004	Sarcoma of endometrium	Cancer
375301	SNOMED CT	447389009	Leiomyosarcoma of uterus	Cancer
375302	SNOMED CT	447390000	Adenosarcoma of uterus	Cancer
375565	SNOMED CT	447656001	Lymphoma of pylorus of stomach	Cancer
375567	SNOMED CT	447658000	Lymphoma of fundus of stomach	Cancer
375612	SNOMED CT	447705002	Malignant germ cell neoplasm of posterior mediastinum	Cancer
375614	SNOMED CT	447707005	Leiomyosarcoma of cardia of stomach	Cancer
375615	SNOMED CT	447708000	Malignant germ cell neoplasm of anterior mediastinum	Cancer
375618	SNOMED CT	447711004	Follicular neoplasm of thyroid	Cancer
375619	SNOMED CT	447712006	Malignant melanoma of skin of anus	Cancer
375645	SNOMED CT	447738006	Paget's disease of skin of scrotum	Cancer
375664	SNOMED CT	447757002	Angiosarcoma of cheek	Cancer
375673	SNOMED CT	447766003	Lymphoma of pyloric antrum of stomach	Cancer
375675	SNOMED CT	447768002	Follicular adenoma of ectopic thyroid tissue	Cancer
375688	SNOMED CT	447781009	Carcinoma of peritoneum	Cancer
375689	SNOMED CT	447782002	Carcinoma of female breast	Cancer
375690	SNOMED CT	447783007	Sarcoma of peritoneum	Cancer
375691	SNOMED CT	447784001	Sarcoma of axillary tail of female breast	Cancer
375692	SNOMED CT	447785000	Leiomyosarcoma of stomach	Cancer
375707	SNOMED CT	447800002	Adenocarcinoma of scrotum	Cancer
375712	SNOMED CT	447805007	Lymphoma of greater curvature of stomach	Cancer
375713	SNOMED CT	447806008	Lymphoma of cardia of stomach	Cancer
375850	SNOMED CT	447949005	Carcinoma of cheek	Cancer

375890	SNOMED CT	447989004	Non-Hodgkin's lymphoma of extranodal site	Cancer
376047	SNOMED CT	448148000	Functioning pituitary neoplasm	Cancer
376110	SNOMED CT	448212009	Anaplastic large cell lymphoma, ALK negative	Cancer
376111	SNOMED CT	448213004	Diffuse non-Hodgkin's lymphoma of prostate	Cancer
376112	SNOMED CT	448214005	Malignant epithelial neoplasm of oropharynx	Cancer
376113	SNOMED CT	448215006	Carcinoma of renal pelvis	Cancer
376114	SNOMED CT	448216007	Carcinoma of thyroid	Cancer
376115	SNOMED CT	448217003	Follicular non-Hodgkin's lymphoma of prostate	Cancer
376116	SNOMED CT	448218008	Malignant neoplasm of cerebellopontine angle	Cancer
376118	SNOMED CT	448220006	Non-Hodgkin's lymphoma of bone	Cancer
376119	SNOMED CT	448221005	Sarcoma of fibula	Cancer
376127	SNOMED CT	448229007	Leiomyosarcoma of lower esophagus	Cancer
376129	SNOMED CT	448231003	Follicular non-Hodgkin's lymphoma of nose	Cancer
376131	SNOMED CT	448233000	Malignant neoplasm of urinary organ	Cancer
376146	SNOMED CT	448248006	Malignant neoplasm of axial suprasellar region of brain	Cancer
376148	SNOMED CT	448250003	Malignant teratoma of pineal region	Cancer
376152	SNOMED CT	448254007	Non-Hodgkin's lymphoma of central nervous system	Cancer
376156	SNOMED CT	448258005	Sarcoma of mesentery	Cancer
376157	SNOMED CT	448259002	Sarcoma of posterior mediastinum	Cancer
376167	SNOMED CT	448269008	Lymphoma of lesser curvature of stomach	Cancer
376171	SNOMED CT	448273006	Malignant melanoma of skin of scrotum	Cancer
376196	SNOMED CT	448298007	Malignant melanoma of skin of penis	Cancer
376197	SNOMED CT	448299004	Malignant neoplasm of mastoid	Cancer
376198	SNOMED CT	448300007	Malignant melanoma of skin of vulva	Cancer
376213	SNOMED CT	448315008	Carcinoma of anus	Cancer

376215	SNOMED CT	448317000	Follicular non-Hodgkin's lymphoma of soft tissue	Cancer
376217	SNOMED CT	448319002	Diffuse non-Hodgkin's lymphoma of nasopharynx	Cancer
376252	SNOMED CT	448354009	Non-Hodgkin's lymphoma of intestine	Cancer
376269	SNOMED CT	448371005	Non-Hodgkin's lymphoma of nasopharynx	Cancer
376270	SNOMED CT	448372003	Non-Hodgkin's lymphoma of lung	Cancer
376274	SNOMED CT	448376000	Non-Hodgkin's lymphoma of ovary	Cancer
376275	SNOMED CT	448377009	Sarcoma of sacrum	Cancer
376276	SNOMED CT	448378004	Sarcoma of bone of foot	Cancer
376282	SNOMED CT	448384001	Non-Hodgkin's lymphoma of nose	Cancer
376284	SNOMED CT	448386004	Non-Hodgkin's lymphoma of oral cavity	Cancer
376285	SNOMED CT	448387008	Non-Hodgkin's lymphoma of testis	Cancer
376286	SNOMED CT	448388003	Sarcoma of lower outer quadrant of female breast	Cancer
376298	SNOMED CT	448401007	Choriocarcinoma of placenta	Cancer
376305	SNOMED CT	448408001	Sarcoma of upper inner quadrant of female breast	Cancer
376326	SNOMED CT	448435005	Sarcoma lower inner quadrant of female breast	Cancer
376327	SNOMED CT	448436006	Sarcoma of central portion of female breast	Cancer
376338	SNOMED CT	448447004	Non-Hodgkin's lymphoma of skin	Cancer
376340	SNOMED CT	448449001	Sarcoma of female breast	Cancer
376341	SNOMED CT	448450001	Sarcoma of omentum	Cancer
376342	SNOMED CT	448451002	Sarcoma upper outer quadrant of female breast	Cancer
376356	SNOMED CT	448465000	Diffuse non-Hodgkin's lymphoma of testis	Cancer
376359	SNOMED CT	448468003	Diffuse non-Hodgkin's lymphoma of oral cavity	Cancer
376398	SNOMED CT	448509007	Transglottic malignant neoplasm of larynx	Cancer
376442	SNOMED CT	448553002	Lymphoma of pelvis	Cancer
376444	SNOMED CT	448555009	Lymphoma of body of stomach	Cancer

376447	SNOMED CT	448558006	Malignant neoplasm of maxillofacial bone	Cancer
376449	SNOMED CT	448560008	Diffuse non-Hodgkin's lymphoma of extranodal site	Cancer
376450	SNOMED CT	448561007	Follicular non-Hodgkin's lymphoma of extranodal site	Cancer
376496	SNOMED CT	448607004	Diffuse non-Hodgkin's lymphoma of uterine cervix	Cancer
376498	SNOMED CT	448609001	Diffuse non-Hodgkin's lymphoma of ovary	Cancer
376552	SNOMED CT	448663003	Diffuse non-Hodgkin's lymphoma of stomach	Cancer
376554	SNOMED CT	448665005	Malignant epithelial neoplasm of hypopharynx	Cancer
376555	SNOMED CT	448666006	Follicular non-Hodgkin's lymphoma of bone	Cancer
376557	SNOMED CT	448668007	Malignant neoplasm of mandible	Cancer
376558	SNOMED CT	448669004	Malignant neoplasm of soft tissue of orbit	Cancer
376559	SNOMED CT	448670003	Malignant neoplasm of posterior mediastinum	Cancer
376561	SNOMED CT	448672006	Follicular non-Hodgkin's lymphoma of lung	Cancer
376563	SNOMED CT	448674007	Malignant neoplasm of parametrium	Cancer
376598	SNOMED CT	448709005	Non-Hodgkin's lymphoma of stomach	Cancer
376599	SNOMED CT	448710000	Sarcoma of bone	Cancer
376601	SNOMED CT	448712008	Sarcoma of femur	Cancer
376627	SNOMED CT	448738008	Non-Hodgkin's lymphoma of soft tissue	Cancer
376663	SNOMED CT	448774004	Non-Hodgkin's lymphoma of uterine cervix	Cancer
376664	SNOMED CT	448775003	Sarcoma of radius	Cancer
376665	SNOMED CT	448776002	Sarcoma of vertebra	Cancer
376748	SNOMED CT	448863000	Carcinoma of pineal gland	Cancer
376749	SNOMED CT	448864006	Carcinoma of ureter	Cancer
376750	SNOMED CT	448865007	Follicular non-Hodgkin's lymphoma of skin	Cancer
376752	SNOMED CT	448867004	Diffuse non-Hodgkin's lymphoma of lung	Cancer
376753	SNOMED CT	448868009	Malignant neoplasm of lateral wall of oropharynx	Cancer

376796	SNOMED CT	448911008	Torsion of uterine fibroid	Cancer
376837	SNOMED CT	448952004	Infiltrating duct carcinoma of female breast	Cancer
376839	SNOMED CT	448954003	Carcinoma of urethra	Cancer
376873	SNOMED CT	448989001	Carcinoma of brain	Cancer
376874	SNOMED CT	448990005	Carcinoma of nasal cavity	Cancer
376877	SNOMED CT	448993007	Carcinoma of lung	Cancer
376879	SNOMED CT	448995000	Follicular non-Hodgkin's lymphoma of central nervous system	Cancer
376916	SNOMED CT	449034009	Malignant neoplasm of anterior and lateral floor of mouth	Cancer
376933	SNOMED CT	449053004	Lymphoma of lower esophagus	Cancer
376934	SNOMED CT	449054005	Carcinoma of fundus of uterus	Cancer
376935	SNOMED CT	449055006	Leiomyosarcoma of cardioesophageal junction	Cancer
376938	SNOMED CT	449058008	Follicular non-Hodgkin's lymphoma of tonsil	Cancer
376939	SNOMED CT	449059000	Follicular non-Hodgkin's lymphoma of uterine cervix	Cancer
376943	SNOMED CT	449063007	Follicular non-Hodgkin's lymphoma of oral cavity	Cancer
376945	SNOMED CT	449065000	Diffuse non-Hodgkin's lymphoma of nose	Cancer
376946	SNOMED CT	449066004	Malignant neoplasm of upper respiratory tract	Cancer
376947	SNOMED CT	449067008	Malignant neoplasm of parietal pleura	Cancer
376952	SNOMED CT	449072004	Gastrointestinal lymphoma	Cancer
376953	SNOMED CT	449073009	Carcinoma of corpus uteri	Cancer
376954	SNOMED CT	449074003	Lymphoma of small bowel	Cancer
376955	SNOMED CT	449075002	Lymphoma of cardioesophageal junction	Cancer
376957	SNOMED CT	449077005	Carcinoma of maxilla	Cancer
376974	SNOMED CT	449097000	Toxic thyroid adenoma	Cancer
376977	SNOMED CT	449100005	Sarcoma of bone of pelvis	Cancer
376978	SNOMED CT	449101009	Sarcoma of sternum	Cancer

376985	SNOMED CT	449108003	Philadelphia chromosome positive chronic myelogenous leukemia	Cancer
377027	SNOMED CT	449153001	Adenocarcinoma of lower esophagus	Cancer
377030	SNOMED CT	449156009	Carcinoma of floor of mouth	Cancer
377047	SNOMED CT	449173006	Diffuse non-Hodgkin's lymphoma of tonsil	Cancer
377050	SNOMED CT	449176003	Diffuse non-Hodgkin's lymphoma of intestine	Cancer
377051	SNOMED CT	449177007	Diffuse non-Hodgkin's lymphoma of bone	Cancer
377080	SNOMED CT	449206007	Sarcoma of tibia	Cancer
377081	SNOMED CT	449207003	Sarcoma of humerus	Cancer
377082	SNOMED CT	449208008	Sarcoma of coccyx	Cancer
377090	SNOMED CT	449216004	Diffuse non-Hodgkin's lymphoma of soft tissue	Cancer
377091	SNOMED CT	449217008	Diffuse non-Hodgkin's lymphoma of skin	Cancer
377092	SNOMED CT	449218003	Lymphoma of sigmoid colon	Cancer
377093	SNOMED CT	449219006	Follicular non-Hodgkin's lymphoma of nasopharynx	Cancer
377094	SNOMED CT	449220000	Diffuse follicle center lymphoma	Cancer
377095	SNOMED CT	449221001	Diffuse non-Hodgkin's lymphoma of central nervous system	Cancer
377096	SNOMED CT	449222008	Follicular non-Hodgkin's lymphoma of stomach	Cancer
377097	SNOMED CT	449223003	Malignant neoplasm of alveolus of maxilla	Cancer
377098	SNOMED CT	449224009	Malignant neoplasm of anterior mediastinum	Cancer
377122	SNOMED CT	449248000	Nasopharyngeal carcinoma	Cancer
377127	SNOMED CT	449253005	Carcinoma of hypothalamus	Cancer
377128	SNOMED CT	449254004	Carcinoma of pharynx	Cancer
377133	SNOMED CT	449259009	Malignant neoplasm of broad ligament of uterus	Cancer
377134	SNOMED CT	449260004	Malignant neoplasm of alveolus dentalis	Cancer
377141	SNOMED CT	449267001	Sarcoma of mandible	Cancer

377142	SNOMED CT	449268006	Sarcoma of clavicle	Cancer
377143	SNOMED CT	449269003	Sarcoma of rib	Cancer
377166	SNOMED CT	449292003	Non-Hodgkin's lymphoma of tonsil	Cancer
377168	SNOMED CT	449294002	Sarcoma of scapula	Cancer
377169	SNOMED CT	449295001	Sarcoma of skull	Cancer
377181	SNOMED CT	449307001	Follicular non-Hodgkin's lymphoma of ovary	Cancer
377182	SNOMED CT	449308006	Malignant neoplasm of visceral pleura	Cancer
377183	SNOMED CT	449309003	Malignant neoplasm of skin of scrotum	Cancer
377192	SNOMED CT	449318001	Non-Hodgkin's lymphoma of prostate	Cancer
377245	SNOMED CT	449377002	Malignant neoplasm of pelvic peritoneum	Cancer
377254	SNOMED CT	449386007	Philadelphia chromosome negative chronic myelogenous leukemia	Cancer
377284	SNOMED CT	449416001	Carcinoma of skin of anus	Cancer
377285	SNOMED CT	449417005	Carcinoma of nasal septum	Cancer
377286	SNOMED CT	449418000	Follicular non-Hodgkin's lymphoma of testis	Cancer
377287	SNOMED CT	449419008	Follicular non-Hodgkin's lymphoma of intestine	Cancer
377288	SNOMED CT	449420002	Malignant neoplasm of cerebellum	Cancer
377340	SNOMED CT	449472007	Malignant epithelial neoplasm of alveolus dentalis	Cancer
377355	SNOMED CT	449487002	Carcinoma of mandible	Cancer
377365	SNOMED CT	449497006	Sarcoma of pelvic peritoneum	Cancer
377443	SNOMED CT	449578008	Malignant neoplasm of alveolus of mandible	Cancer
377489	SNOMED CT	449628003	Malignant neoplasm of long bone of lower leg	Cancer
377497	SNOMED CT	449636007	Malignant melanoma of skin of lower leg	Cancer
377498	SNOMED CT	449637003	Malignant melanoma of skin of upper arm	Cancer
388330	SNOMED CT	698646006	Acute monoblastic leukemia in remission	Cancer
212377	SNOMED CT	230156002	Malignant meningitis	Cancer

214021	SNOMED CT	231829006	Malignant tumor of eyelid	Cancer
214022	SNOMED CT	231831002	Squamous cell carcinoma of eyelid	Cancer
214023	SNOMED CT	231832009	Basal cell carcinoma of eyelid	Cancer
214024	SNOMED CT	231833004	Meibomian gland carcinoma	Cancer
214025	SNOMED CT	231834005	Malignant melanoma of eyelid	Cancer
214259	SNOMED CT	232075002	Lymphoma of retina	Cancer
216022	SNOMED CT	233854003	Atrial myxoma	Cancer
216023	SNOMED CT	233855002	Familial atrial myxoma	Cancer
217825	SNOMED CT	235686008	Fundic gland polyposis of stomach	Cancer
218637	SNOMED CT	236512004	Leukemic infiltrate of kidney	Cancer
218638	SNOMED CT	236513009	Lymphoma of kidney	Cancer
219817	SNOMED CT	237719001	Pituitary adenoma with extrasellar extension	Cancer
219886	SNOMED CT	237795006	Virilizing ovarian tumor	Cancer
222189	SNOMED CT	240163000	Malignant neoplasm of nasopharyngeal wall	Cancer
223841	SNOMED CT	241861008	Metastatic malignant neoplasm to nasopharynx	Cancer
234571	SNOMED CT	252991009	Cervical intraepithelial neoplasia grade III with severe dysplasia	Cancer
234583	SNOMED CT	253003009	Carcinoid bronchial adenoma	Cancer
235940	SNOMED CT	254389005	Carcinoma of vermilion border of upper lip	Cancer
235941	SNOMED CT	254390001	Carcinoma of vermilion border of lower lip	Cancer
235944	SNOMED CT	254393004	Carcinoma of frenum of lip	Cancer
235949	SNOMED CT	254398008	Carcinoma of frenum of upper lip	Cancer
235953	SNOMED CT	254402004	Carcinoma of frenum of lower lip	Cancer
235955	SNOMED CT	254404003	Carcinoma of commissure of lip	Cancer
235959	SNOMED CT	254408000	Malignant tumor of anterior two-thirds of tongue - lateral margin	Cancer
235963	SNOMED CT	254412006	Malignant tumor of tip of tongue	Cancer

235968	SNOMED CT	254417000	Carcinoma of frenum linguae	Cancer
235974	SNOMED CT	254423005	Carcinoma of lingual tonsil	Cancer
235975	SNOMED CT	254424004	Carcinoma of upper gum	Cancer
235976	SNOMED CT	254425003	Carcinoma of lower gum	Cancer
235978	SNOMED CT	254427006	Carcinoma of anterior part of floor of mouth	Cancer
235982	SNOMED CT	254431000	Carcinoma of lateral part of floor of mouth	Cancer
235985	SNOMED CT	254434008	Carcinoma of hard palate	Cancer
235986	SNOMED CT	254435009	Carcinoma of soft palate	Cancer
235988	SNOMED CT	254437001	Squamous cell carcinoma of buccal mucosa	Cancer
235992	SNOMED CT	254441002	Carcinoma of upper buccal sulcus	Cancer
235996	SNOMED CT	254445006	Carcinoma of lower buccal sulcus	Cancer
236001	SNOMED CT	254450000	Carcinoma of upper labial sulcus	Cancer
236005	SNOMED CT	254454009	Carcinoma of lower labial sulcus	Cancer
236008	SNOMED CT	254457002	Carcinoma of retromolar area	Cancer
236010	SNOMED CT	254459004	Malignant tumor of anterior pillar of fauces	Cancer
236013	SNOMED CT	254462001	Carcinoma of parotid gland	Cancer
236016	SNOMED CT	254465004	Carcinoma of submandibular gland	Cancer
236017	SNOMED CT	254466003	Carcinoma of sublingual gland	Cancer
236028	SNOMED CT	254478004	Malignant tumor of inferior turbinate	Cancer
236031	SNOMED CT	254481009	Malignant tumor of middle turbinate	Cancer
236034	SNOMED CT	254484001	Malignant tumor of posterior margin of nasal septum and choanae	Cancer
236051	SNOMED CT	254501009	Tumor of inferior surface of soft palate	Cancer
236053	SNOMED CT	254503007	Malignant tumor of inferior surface of soft palate	Cancer
236059	SNOMED CT	254509006	Malignant tumor of anterior commissure	Cancer
236063	SNOMED CT	254513004	Malignant tumor of posterior commissure	Cancer

236067	SNOMED CT	254517003	Malignant tumor of suprahyoid epiglottis	Cancer
236070	SNOMED CT	254520006	Malignant tumor of infrahyoid epiglottis	Cancer
236076	SNOMED CT	254526000	Malignant tumor of laryngeal ventricle	Cancer
236080	SNOMED CT	254530002	Malignant tumor of parapharyngeal space	Cancer
236085	SNOMED CT	254535007	Carcinoma of cervical part of esophagus	Cancer
236089	SNOMED CT	254539001	Carcinoma of thoracic part of esophagus	Cancer
236093	SNOMED CT	254543002	Carcinoma of abdominal part of esophagus	Cancer
236097	SNOMED CT	254547001	Carcinoma of upper third of esophagus	Cancer
236099	SNOMED CT	254549003	Carcinoma of middle third of esophagus	Cancer
236101	SNOMED CT	254551004	Carcinoma of lower third of esophagus	Cancer
236103	SNOMED CT	254553001	Carcinoma of cardia	Cancer
236105	SNOMED CT	254555008	Carcinoma of fundus of stomach	Cancer
236107	SNOMED CT	254557000	Carcinoma of body of stomach	Cancer
236109	SNOMED CT	254559002	Carcinoma of pyloric antrum	Cancer
236111	SNOMED CT	254561006	Carcinoma of pylorus	Cancer
236113	SNOMED CT	254563009	Carcinoma of lesser curve of stomach	Cancer
236117	SNOMED CT	254567005	Carcinoma of greater curve of stomach	Cancer
236120	SNOMED CT	254570009	Carcinoma of duodenum	Cancer
236124	SNOMED CT	254574000	Diffuse leiomyomatosis of esophagus	Cancer
236136	SNOMED CT	254586002	Malignant tumor of anorectal junction	Cancer
236159	SNOMED CT	254609000	Carcinoma of ampulla of Vater	Cancer
236161	SNOMED CT	254611009	Malignant tumor of endocrine pancreas	Cancer
236162	SNOMED CT	254612002	Carcinoma of endocrine pancreas	Cancer
236163	SNOMED CT	254613007	Carcinoid tumor of pancreas	Cancer
236165	SNOMED CT	254615000	Endocrine pancreatic adenoma	Cancer
236169	SNOMED CT	254619006	Adenoid cystic carcinoma of trachea	Cancer

236170	SNOMED CT	254620000	Squamous cell carcinoma of trachea	Cancer
236171	SNOMED CT	254621001	Laryngotracheal papillomatosis	Cancer
236172	SNOMED CT	254622008	Squamous cell carcinoma of bronchus	Cancer
236175	SNOMED CT	254625005	Malignant tumor of lung parenchyma	Cancer
236176	SNOMED CT	254626006	Adenocarcinoma of lung	Cancer
236177	SNOMED CT	254627002	Carcinoid tumor of lung	Cancer
236179	SNOMED CT	254629004	Large cell carcinoma of lung	Cancer
236181	SNOMED CT	254631008	Giant cell carcinoma of lung	Cancer
236182	SNOMED CT	254632001	Small cell carcinoma of lung	Cancer
236183	SNOMED CT	254633006	Oat cell carcinoma of lung	Cancer
236184	SNOMED CT	254634000	Squamous cell carcinoma of lung	Cancer
236185	SNOMED CT	254635004	Epithelioid hemangioendothelioma of lung	Cancer
236187	SNOMED CT	254637007	Non-small cell lung cancer	Cancer
236188	SNOMED CT	254638002	Pancoast tumor	Cancer
236191	SNOMED CT	254641006	Histiocytoma of lung	Cancer
236193	SNOMED CT	254643009	Intrapulmonary teratoma	Cancer
236194	SNOMED CT	254644003	Hamartoma of lung	Cancer
236195	SNOMED CT	254645002	Malignant mesothelioma of pleura	Cancer
236278	SNOMED CT	254730000	Superficial spreading malignant melanoma of skin	Cancer
236279	SNOMED CT	254731001	Nodular malignant melanoma of skin	Cancer
236280	SNOMED CT	254732008	Acral lentiginous malignant melanoma of skin	Cancer
236281	SNOMED CT	254733003	Malignant melanoma arising in intradermal nevus	Cancer
236282	SNOMED CT	254734009	Malignant melanoma arising in congenital nevus	Cancer
236338	SNOMED CT	254792006	Proliferating angioendotheliomatosis	Cancer
236395	SNOMED CT	254849005	Malignant epithelial tumor of ovary	Cancer
236396	SNOMED CT	254850005	Serous papillary cystadenocarcinoma ovary	Cancer

236397	SNOMED CT	254851009	Mucinous cystadenocarcinoma of ovary	Cancer
236398	SNOMED CT	254852002	Endometrioid carcinoma ovary	Cancer
236402	SNOMED CT	254856004	Undifferentiated carcinoma of ovary	Cancer
236406	SNOMED CT	254860001	Malignant sex cord tumor of ovary	Cancer
236409	SNOMED CT	254863004	Granulosa cell tumor of ovary	Cancer
236410	SNOMED CT	254864005	Theca cell tumor of ovary	Cancer
236412	SNOMED CT	254866007	Sertoli-Leydig cell tumor of ovary	Cancer
236413	SNOMED CT	254867003	Gynandroblastoma of ovary	Cancer
236414	SNOMED CT	254868008	Hilus cell tumor of ovary	Cancer
236415	SNOMED CT	254869000	Malignant germ cell tumor of ovary	Cancer
236416	SNOMED CT	254870004	Choriocarcinoma of ovary	Cancer
236418	SNOMED CT	254872007	Embryonal carcinoma of ovary	Cancer
236420	SNOMED CT	254874008	Dysgerminoma of ovary	Cancer
236422	SNOMED CT	254876005	Endodermal sinus tumor of ovary	Cancer
236423	SNOMED CT	254877001	Sarcoma of uterus	Cancer
236424	SNOMED CT	254878006	Endometrial carcinoma	Cancer
236429	SNOMED CT	254883003	Intravenous leiomyomatosis	Cancer
236432	SNOMED CT	254886006	Squamous cell carcinoma of cervix	Cancer
236433	SNOMED CT	254887002	Adenocarcinoma of cervix	Cancer
236434	SNOMED CT	254888007	Adenosquamous carcinoma of cervix	Cancer
236435	SNOMED CT	254889004	Carcinoma of cervix stage 0	Cancer
236439	SNOMED CT	254893005	Carcinoma of vagina	Cancer
236440	SNOMED CT	254894004	Vaginal intraepithelial neoplasia	Cancer
236445	SNOMED CT	254900004	Carcinoma of prostate	Cancer
236446	SNOMED CT	254901000	Prostatic intraepithelial neoplasia	Cancer
236449	SNOMED CT	254904008	Carcinoma of glans penis	Cancer

236453	SNOMED CT	254908006	Malignant tumor of skin of penis	Cancer
236454	SNOMED CT	254909003	Carcinoma of foreskin	Cancer
236457	SNOMED CT	254912000	Regressed malignant testicular tumor	Cancer
236460	SNOMED CT	254915003	Clear cell carcinoma of kidney	Cancer
236461	SNOMED CT	254916002	Cystadenocarcinoma of kidney	Cancer
236462	SNOMED CT	254917006	Papillary cystadenocarcinoma of kidney	Cancer
236463	SNOMED CT	254918001	Sarcoma of kidney	Cancer
236464	SNOMED CT	254919009	Cortical adenoma of kidney	Cancer
236465	SNOMED CT	254920003	Cystadenoma of kidney	Cancer
236466	SNOMED CT	254921004	Angiomyolipoma of kidney	Cancer
236467	SNOMED CT	254922006	Oncocytoma of kidney	Cancer
236468	SNOMED CT	254923001	Hemangiopericytoma of kidney	Cancer
236479	SNOMED CT	254934003	Malignant tumor of urethral stump	Cancer
236481	SNOMED CT	254936001	Glial tumor of brain	Cancer
236483	SNOMED CT	254938000	Astrocytoma of brain	Cancer
236485	SNOMED CT	254940005	Oligodendroglioma of brain	Cancer
236486	SNOMED CT	254941009	Mixed glial tumor of brain	Cancer
236490	SNOMED CT	254945000	Embryonal tumor of brain	Cancer
236491	SNOMED CT	254946004	Glial tumor of spinal cord	Cancer
236492	SNOMED CT	254947008	Glioma of spinal cord	Cancer
236493	SNOMED CT	254948003	Astrocytoma of spinal cord	Cancer
236495	SNOMED CT	254950006	Oligodendroglioma of spinal cord	Cancer
236496	SNOMED CT	254951005	Mixed glial tumor of spinal cord	Cancer
236499	SNOMED CT	254954002	Embryonal tumor of spinal cord	Cancer
236500	SNOMED CT	254955001	Pituitary carcinoma	Cancer
236501	SNOMED CT	254956000	Pituitary adenoma	Cancer

236502	SNOMED CT	254957009	Somatotroph adenoma	Cancer
236503	SNOMED CT	254958004	Corticotroph adenoma	Cancer
236504	SNOMED CT	254959007	Thyrotroph adenoma	Cancer
236505	SNOMED CT	254960002	Gonadotroph adenoma	Cancer
236506	SNOMED CT	254961003	Mixed-functioning pituitary adenoma	Cancer
236507	SNOMED CT	254962005	Functionless pituitary adenoma	Cancer
236508	SNOMED CT	254963000	Pituitary microadenoma	Cancer
236509	SNOMED CT	254964006	Pituitary mesoadenoma	Cancer
236510	SNOMED CT	254965007	Pituitary macroadenoma	Cancer
236511	SNOMED CT	254966008	Suprasellar extension of pituitary adenoma	Cancer
236513	SNOMED CT	254968009	Tumor of hypothalamus	Cancer
236514	SNOMED CT	254969001	Malignant tumor of olfactory tract	Cancer
236517	SNOMED CT	254972008	Malignant tumor of optic nerve and sheath	Cancer
236518	SNOMED CT	254973003	Malignant astrocytoma of optic nerve	Cancer
236519	SNOMED CT	254974009	Malignant tumor of optic nerve sheath	Cancer
236520	SNOMED CT	254975005	Malignant meningioma of optic nerve sheath	Cancer
236521	SNOMED CT	254976006	Optic nerve glioma	Cancer
236523	SNOMED CT	254978007	Meningioma of optic nerve sheath	Cancer
236524	SNOMED CT	254979004	Melanocytoma of optic nerve head	Cancer
236532	SNOMED CT	254987003	Adenoid cystic carcinoma of lacrimal gland	Cancer
236533	SNOMED CT	254988008	Adenocarcinoma of lacrimal gland	Cancer
236534	SNOMED CT	254989000	Carcinoma ex pleomorphic adenoma of lacrimal gland	Cancer
236535	SNOMED CT	254990009	Mucoepidermoid tumor of lacrimal gland	Cancer
236538	SNOMED CT	254993006	Liposarcoma of orbit	Cancer
236539	SNOMED CT	254994000	Rhabdomyosarcoma of orbit	Cancer
236540	SNOMED CT	254995004	Malignant hemangiopericytoma of orbit	Cancer

236541	SNOMED CT	254996003	Malignant fibrous histiocytoma of orbit	Cancer
236542	SNOMED CT	254997007	Capillary hemangioma of orbit	Cancer
236543	SNOMED CT	254998002	Cavernous hemangioma of orbit	Cancer
236544	SNOMED CT	254999005	Hemangiopericytoma of orbit	Cancer
236545	SNOMED CT	255000005	Neurofibroma of orbit	Cancer
236548	SNOMED CT	255003007	Squamous cell carcinoma of conjunctiva	Cancer
236549	SNOMED CT	255004001	Malignant melanoma of conjunctiva	Cancer
236552	SNOMED CT	255008003	Squamous cell carcinoma of cornea	Cancer
236556	SNOMED CT	255012009	Malignant melanoma of iris	Cancer
236559	SNOMED CT	255015006	Malignant melanoma of ciliary body	Cancer
236560	SNOMED CT	255016007	Adenocarcinoma of pigmented epithelium of ciliary body	Cancer
236561	SNOMED CT	255017003	Adenocarcinoma of non-pigmented epithelium of ciliary body	Cancer
236565	SNOMED CT	255021005	Malignant melanoma of choroid	Cancer
236572	SNOMED CT	255028004	Follicular thyroid carcinoma	Cancer
236573	SNOMED CT	255029007	Papillary thyroid carcinoma	Cancer
236574	SNOMED CT	255030002	Mixed follicular and papillary thyroid carcinoma	Cancer
236575	SNOMED CT	255031003	Anaplastic thyroid carcinoma	Cancer
236576	SNOMED CT	255032005	Medullary thyroid carcinoma	Cancer
236578	SNOMED CT	255034006	Thyroid follicular adenoma	Cancer
236579	SNOMED CT	255035007	Adrenal carcinoma	Cancer
236581	SNOMED CT	255037004	Parathyroid carcinoma	Cancer
236611	SNOMED CT	255067005	Sarcoma of bone and connective tissue	Cancer
236614	SNOMED CT	255070009	Overlapping malignant neoplasm of oral cavity and lip and salivary gland	Cancer
236615	SNOMED CT	255071008	Squamous cell carcinoma of lip	Cancer
236616	SNOMED CT	255072001	Malignant tumor of salivary gland	Cancer

236618	SNOMED CT	255074000	Malignant tumor of nasal cavity and nasopharynx	Cancer
236619	SNOMED CT	255075004	Malignant tumor of lateral nasal wall	Cancer
236622	SNOMED CT	255078002	Malignant tumor of esophagus, stomach and duodenum	Cancer
236628	SNOMED CT	255084004	Squamous cell carcinoma of anal margin	Cancer
236634	SNOMED CT	255090000	Malignant neoplasm of carpal bones	Cancer
236635	SNOMED CT	255091001	Malignant neoplasm of metacarpal bones	Cancer
236652	SNOMED CT	255108000	Carcinoma of bladder	Cancer
236653	SNOMED CT	255109008	Transitional cell carcinoma of bladder	Cancer
236654	SNOMED CT	255110003	Adenocarcinoma of bladder	Cancer
236655	SNOMED CT	255111004	Squamous cell carcinoma of bladder	Cancer
236656	SNOMED CT	255112006	Malignant tumor of pituitary and hypothalamus	Cancer
236660	SNOMED CT	255119002	Lymphangitis carcinomatosa	Cancer
236663	SNOMED CT	255123005	Metastasis to nervous system and eye	Cancer
236730	SNOMED CT	255191003	Localized malignant reticulohistiocytoma	Cancer
246092	SNOMED CT	264906008	Pituitary macroadenoma with extrasellar extension	Cancer
250584	SNOMED CT	269459004	Malignant tumor of lesser curve of stomach	Cancer
250585	SNOMED CT	269460009	Malignant tumor of greater curve of stomach	Cancer
250587	SNOMED CT	269463006	Malignant tumor of middle ear and mastoid	Cancer
250588	SNOMED CT	269464000	Malignant neoplasm of upper lobe, bronchus or lung	Cancer
250591	SNOMED CT	269467007	Malignant neoplasm of hand bones	Cancer
250599	SNOMED CT	269475001	Malignant tumor of lymphoid hemopoietic and related tissue	Cancer
250600	SNOMED CT	269476000	Nodular lymphoma	Cancer
250639	SNOMED CT	269515006	Carcinoma of lip	Cancer
250640	SNOMED CT	269516007	Tongue carcinoma	Cancer
250657	SNOMED CT	269533000	Carcinoma of colon	Cancer

250668	SNOMED CT	269544008	Carcinoma of the rectosigmoid junction	Cancer
250704	SNOMED CT	269581007	Malignant melanoma of lower limb	Cancer
251958	SNOMED CT	271568003	Malignant tumor of lower labial mucosa	Cancer
252330	SNOMED CT	271943005	Carcinoma of base of tongue	Cancer
254418	SNOMED CT	274084007	Palate carcinoma	Cancer
254419	SNOMED CT	274085008	Tonsil carcinoma	Cancer
254421	SNOMED CT	274087000	Malignant melanoma of eye	Cancer
255204	SNOMED CT	274905008	Malignant lymphoma - lymphocytic, intermediate differentiation	Cancer
255686	SNOMED CT	275394001	Carcinoma ventral surface of tongue	Cancer
255687	SNOMED CT	275395000	Carcinoma anterior 2/3 tongue ventrum	Cancer
255688	SNOMED CT	275396004	Carcinoma of anterior two-thirds of tongue - dorsal surface	Cancer
255689	SNOMED CT	275397008	Carcinoma of midline of tongue	Cancer
255691	SNOMED CT	275399006	Malignant tumor of lipstick area of lip	Cancer
255782	SNOMED CT	275490009	Carcinoma of tongue base - dorsal surface	Cancer
256599	SNOMED CT	276419004	Malignant tumor of corpus spongiosum	Cancer
256600	SNOMED CT	276420005	Malignant tumor of corpus cavernosum	Cancer
256926	SNOMED CT	276751004	Amelanotic malignant melanoma of skin	Cancer
256978	SNOMED CT	276803003	Adenocarcinoma of esophagus	Cancer
256979	SNOMED CT	276804009	Squamous cell carcinoma of esophagus	Cancer
256983	SNOMED CT	276808007	Carcinoid tumor of stomach	Cancer
256984	SNOMED CT	276809004	Early gastric cancer	Cancer
256985	SNOMED CT	276810009	Late gastric cancer	Cancer
256986	SNOMED CT	276811008	Gastric lymphoma	Cancer
256990	SNOMED CT	276815004	Lymphoma of intestine	Cancer
256996	SNOMED CT	276821000	Malignant melanoma of anus	Cancer

257001	SNOMED CT	276826005	Malignant glioma of brain	Cancer
257002	SNOMED CT	276827001	Malignant glioma of spinal cord	Cancer
257003	SNOMED CT	276828006	Glioblastoma multiforme of brain	Cancer
257004	SNOMED CT	276829003	Glioblastoma multiforme of spinal cord	Cancer
257011	SNOMED CT	276836002	Primary cerebral lymphoma	Cancer
257027	SNOMED CT	276852004	Papillary tumor of ampulla of Vater	Cancer
257035	SNOMED CT	276860003	Squamous cell carcinoma of scrotum	Cancer
257045	SNOMED CT	276870001	Carcinoma of fallopian tube	Cancer
257046	SNOMED CT	276871002	Vaginal intraepithelial neoplasia grade 1	Cancer
257047	SNOMED CT	276872009	Vaginal intraepithelial neoplasia grade 2	Cancer
257124	SNOMED CT	276952000	Squamous cell carcinoma of tongue	Cancer
257125	SNOMED CT	276953005	Squamous cell carcinoma of gum	Cancer
257126	SNOMED CT	276954004	Squamous cell carcinoma of floor of mouth	Cancer
257134	SNOMED CT	276962007	Squamous cell carcinoma of palate	Cancer
257140	SNOMED CT	276968006	Odontogenic tumor of jaw	Cancer
257147	SNOMED CT	276975007	Carcinoma of larynx	Cancer
257617	SNOMED CT	277456001	Suprasellar germ cell tumor	Cancer
257622	SNOMED CT	277461004	Anaplastic astrocytoma of brain	Cancer
257633	SNOMED CT	277473004	B-cell chronic lymphocytic leukemia	Cancer
257634	SNOMED CT	277474005	B-cell chronic lymphocytic leukemia variant	Cancer
257661	SNOMED CT	277505007	Medulloblastoma of cerebellum	Cancer
257663	SNOMED CT	277507004	Pilocytic astrocytoma of cerebellum	Cancer
257664	SNOMED CT	277508009	Pineal germ cell tumor	Cancer
257679	SNOMED CT	277523004	Primary melanocytic lesion of meninges	Cancer
257682	SNOMED CT	277526007	Diffuse melanosis of meninges	Cancer
257683	SNOMED CT	277527003	Melanocytoma of meninges	Cancer

257686	SNOMED CT	277530005	Malignant melanoma of meninges	Cancer
257700	SNOMED CT	277545003	T-cell chronic lymphocytic leukemia	Cancer
257704	SNOMED CT	277549009	Chronic lymphocytic prolymphocytic leukemia syndrome	Cancer
257705	SNOMED CT	277551008	Splenic lymphoma with villous lymphocytes	Cancer
257721	SNOMED CT	277567002	T-cell prolymphocytic leukemia	Cancer
257722	SNOMED CT	277568007	Hairy cell leukemia variant	Cancer
257723	SNOMED CT	277569004	Large granular lymphocytic leukemia	Cancer
257724	SNOMED CT	277570003	Lymphoma with spill	Cancer
257725	SNOMED CT	277571004	B-cell acute lymphoblastic leukemia	Cancer
257726	SNOMED CT	277572006	Precursor B-cell acute lymphoblastic leukemia	Cancer
257727	SNOMED CT	277573001	Common acute lymphoblastic leukemia	Cancer
257728	SNOMED CT	277574007	Null cell acute lymphoblastic leukemia	Cancer
257729	SNOMED CT	277575008	T-cell acute lymphoblastic leukemia	Cancer
257741	SNOMED CT	277587001	Juvenile chronic myeloid leukemia	Cancer
257743	SNOMED CT	277589003	Atypical chronic myeloid leukemia	Cancer
257751	SNOMED CT	277597005	Myelodysplastic syndrome with isolated del(5q)	Cancer
257755	SNOMED CT	277601005	Acute monoblastic leukemia	Cancer
257756	SNOMED CT	277602003	Acute megakaryoblastic leukemia	Cancer
257758	SNOMED CT	277604002	Acute eosinophilic leukemia	Cancer
257762	SNOMED CT	277613000	Cutaneous/peripheral T-cell lymphoma	Cancer
257763	SNOMED CT	277614006	Prethymic and thymic T-cell lymphoma/leukemia	Cancer
257764	SNOMED CT	277615007	Low grade B-cell lymphoma	Cancer
257765	SNOMED CT	277616008	Diffuse low grade B-cell lymphoma	Cancer
257766	SNOMED CT	277617004	High grade B-cell lymphoma	Cancer
257767	SNOMED CT	277618009	Follicular low grade B-cell lymphoma	Cancer

257768	SNOMED CT	277619001	B-cell prolymphocytic leukemia	Cancer
257771	SNOMED CT	277622004	Mucosa-associated lymphoma	Cancer
257772	SNOMED CT	277623009	Monocytoid B-cell lymphoma	Cancer
257773	SNOMED CT	277624003	Follicular malignant lymphoma - mixed cell type	Cancer
257774	SNOMED CT	277625002	Follicular malignant lymphoma - small cleaved cell	Cancer
257775	SNOMED CT	277626001	Diffuse high grade B-cell lymphoma	Cancer
257776	SNOMED CT	277627005	Nodular high grade B-cell lymphoma	Cancer
257777	SNOMED CT	277628000	Diffuse malignant lymphoma - large cleaved cell	Cancer
257778	SNOMED CT	277629008	Diffuse malignant lymphoma - large non-cleaved cell	Cancer
257781	SNOMED CT	277632006	Diffuse malignant lymphoma - centroblastic polymorphic	Cancer
257786	SNOMED CT	277637000	Large cell anaplastic lymphoma	Cancer
257791	SNOMED CT	277642008	Low grade T-cell lymphoma	Cancer
257792	SNOMED CT	277643003	High grade T-cell lymphoma	Cancer
257800	SNOMED CT	277651000	Peripheral T-cell lymphoma - pleomorphic small cell	Cancer
257802	SNOMED CT	277653002	Peripheral T-cell lymphoma - pleomorphic medium and large cell	Cancer
257812	SNOMED CT	277664004	Malignant lymphoma of testis	Cancer
257927	SNOMED CT	277782009	Malignant peritoneal local recurrence	Cancer
258169	SNOMED CT	278024000	Rhabdomyosarcoma of bladder	Cancer
258186	SNOMED CT	278042005	Malignant teratoma of mediastinum	Cancer
258187	SNOMED CT	278043000	Malignant seminoma of mediastinum	Cancer
258188	SNOMED CT	278044006	Malignant neuroma of mediastinum	Cancer
258190	SNOMED CT	278046008	Sarcoma of bladder	Cancer
258195	SNOMED CT	278051002	Malignant lymphoma of thyroid gland	Cancer
258196	SNOMED CT	278052009	Malignant lymphoma of breast	Cancer
258199	SNOMED CT	278055006	Malignant Leydig cell tumor of testis	Cancer

258201	SNOMED CT	278057003	Sertoli cell tumor of testis	Cancer
258204	SNOMED CT	278060005	Endometrioid carcinoma of prostate	Cancer
258328	SNOMED CT	278189009	Hypergranular promyelocytic leukemia	Cancer
258581	SNOMED CT	278453007	Acute biphenotypic leukemia	Cancer
258619	SNOMED CT	278491007	Mixed seminoma teratoma of testis	Cancer
260224	SNOMED CT	280116003	Tumor of tunica vaginalis	Cancer
261059	SNOMED CT	280959007	Malignant tumor of lacrimal drainage structure	Cancer
261652	SNOMED CT	281560004	Neuroblastoma of brain	Cancer
261653	SNOMED CT	281561000	Sacrococcygeal teratoma	Cancer
261654	SNOMED CT	281562007	Adrenal neuroblastoma	Cancer
261655	SNOMED CT	281563002	Thoracic neuroblastoma	Cancer
261658	SNOMED CT	281566005	Abdominothoracic neuroblastoma	Cancer
261792	SNOMED CT	281702006	Tibial adamantinoma	Cancer
265321	SNOMED CT	285307007	Squamous cell carcinoma of skin of upper lip	Cancer
265322	SNOMED CT	285308002	Squamous cell carcinoma of skin of lower lip	Cancer
265323	SNOMED CT	285309005	Squamous cell carcinoma of skin of cheek	Cancer
265324	SNOMED CT	285310000	Carcinoma of anal canal	Cancer
265325	SNOMED CT	285311001	Ameloblastoma of jaw	Cancer
265326	SNOMED CT	285312008	Carcinoma of sigmoid colon	Cancer
265439	SNOMED CT	285432005	Carcinoma of cervix	Cancer
265603	SNOMED CT	285598005	Metastasis to trachea of unknown primary	Cancer
265608	SNOMED CT	285603002	Metastasis to bronchus of unknown primary	Cancer
265609	SNOMED CT	285604008	Metastasis to lung of unknown primary	Cancer
265610	SNOMED CT	285605009	Metastasis to pleura of unknown primary	Cancer
265611	SNOMED CT	285606005	Metastasis to heart of unknown primary	Cancer
265612	SNOMED CT	285607001	Metastasis to mediastinum of unknown primary	Cancer

265613	SNOMED CT	285608006	Metastasis to thymus of unknown primary	Cancer
265616	SNOMED CT	285611007	Metastasis to colon of unknown primary	Cancer
265621	SNOMED CT	285616002	Metastasis to peritoneum of unknown primary	Cancer
265623	SNOMED CT	285618001	Metastasis to bone of unknown primary	Cancer
265624	SNOMED CT	285619009	Metastasis to vertebral column of unknown primary	Cancer
265639	SNOMED CT	285634003	Metastasis to breast of unknown primary	Cancer
265640	SNOMED CT	285635002	Metastasis to uterus of unknown primary	Cancer
265641	SNOMED CT	285636001	Cervical intraepithelial neoplasia	Cancer
265642	SNOMED CT	285637005	Metastasis to ovary of unknown primary	Cancer
265644	SNOMED CT	285639008	Metastasis to kidney of unknown primary	Cancer
265645	SNOMED CT	285640005	Metastasis to bladder of unknown primary	Cancer
265646	SNOMED CT	285641009	Metastasis to brain of unknown primary	Cancer
265647	SNOMED CT	285642002	Metastasis to eye of unknown primary	Cancer
265648	SNOMED CT	285643007	Metastasis to adrenal gland of unknown primary	Cancer
265649	SNOMED CT	285644001	Metastasis to lymph node of unknown primary	Cancer
265764	SNOMED CT	285769009	Acute promyelocytic leukemia - hypogranular variant	Cancer
265771	SNOMED CT	285776004	Intermediate grade B-cell lymphoma	Cancer
265830	SNOMED CT	285836003	Cervical intraepithelial neoplasia grade 1	Cancer
265832	SNOMED CT	285838002	Cervical intraepithelial neoplasia grade 2	Cancer
265833	SNOMED CT	285839005	Acute myelomonocytic leukemia - eosinophilic variant	Cancer
266869	SNOMED CT	286889008	Carcinoma of upper limb bones/scapula	Cancer
266870	SNOMED CT	286890004	Carcinoma of lower limb bones	Cancer
280812	SNOMED CT	300988009	Transitional cell carcinoma of ureter	Cancer
281577	SNOMED CT	301756000	Adenocarcinoma of sigmoid colon	Cancer
282607	SNOMED CT	302815008	Malignant tumor of frenum of lip	Cancer

282612	SNOMED CT	302820008	Intracranial meningioma	Cancer
282615	SNOMED CT	302823005	Glucagonoma	Cancer
282616	SNOMED CT	302824004	Gastrinoma	Cancer
282618	SNOMED CT	302826002	Adrenal cortical adenoma	Cancer
282628	SNOMED CT	302837001	Lentigo maligna melanoma	Cancer
282632	SNOMED CT	302841002	Malignant lymphoma - small lymphocytic	Cancer
282633	SNOMED CT	302842009	Diffuse malignant lymphoma - centroblastic	Cancer
282636	SNOMED CT	302845006	Nodular malignant lymphoma, lymphocytic - well differentiated	Cancer
282639	SNOMED CT	302848008	Nodular malignant lymphoma, lymphocytic - intermediate differentiation	Cancer
282640	SNOMED CT	302849000	Nephroblastoma	Cancer
282646	SNOMED CT	302855005	Subacute leukemia	Cancer
282647	SNOMED CT	302856006	Aleukemic leukemia	Cancer
282791	SNOMED CT	303012000	Malignant tumor of posterior wall of hypopharynx	Cancer
282795	SNOMED CT	303017006	Malignant lymphoma, convoluted cell type	Cancer
282808	SNOMED CT	303055001	Malignant lymphoma, follicular center cell	Cancer
282809	SNOMED CT	303056000	Malignant lymphoma, follicular center cell, cleaved	Cancer
282810	SNOMED CT	303057009	Malignant lymphoma, follicular center cell, non-cleaved	Cancer
282909	SNOMED CT	303194003	Metastasis to head and neck lymph node	Cancer
282910	SNOMED CT	303201005	Metastasis to multiple lymph nodes	Cancer
286736	SNOMED CT	307216009	Perforated carcinoma of esophagus	Cancer
286859	SNOMED CT	307340003	Monosomy 7 syndrome	Cancer
286860	SNOMED CT	307341004	Atypical hairy cell leukemia	Cancer
287021	SNOMED CT	307502000	Squamous cell carcinoma of mouth	Cancer
287094	SNOMED CT	307576001	Osteosarcoma	Cancer
287109	SNOMED CT	307592006	Basophilic leukemia	Cancer

287117	SNOMED CT	307601000	Pseudomyxoma peritonei	Cancer
287119	SNOMED CT	307603002	Malignant blue nevus of skin	Cancer
287120	SNOMED CT	307604008	Mesoblastic nephroma	Cancer
287121	SNOMED CT	307605009	Osteoblastoma of bone	Cancer
287122	SNOMED CT	307606005	Osteochondroma of bone	Cancer
287123	SNOMED CT	307607001	Chondromyxoid fibroma of bone	Cancer
287124	SNOMED CT	307609003	Adamantinoma of long bone	Cancer
287132	SNOMED CT	307617006	Neutrophilic leukemia	Cancer
287133	SNOMED CT	307618001	Juxtaglomerular tumor	Cancer
287137	SNOMED CT	307622006	Prolymphocytic lymphosarcoma	Cancer
287138	SNOMED CT	307623001	Malignant lymphoma - lymphoplasmacytic	Cancer
287139	SNOMED CT	307624007	Diffuse malignant lymphoma - centroblastic-centrocytic	Cancer
287140	SNOMED CT	307625008	Malignant lymphoma - centrocytic	Cancer
287148	SNOMED CT	307636001	Malignant lymphoma, mixed lymphocytic-histiocytic, nodular	Cancer
287149	SNOMED CT	307637005	Malignant lymphoma, centroblastic-centrocytic, follicular	Cancer
287158	SNOMED CT	307646004	Malignant lymphoma, lymphocytic, poorly differentiated, nodular	Cancer
287159	SNOMED CT	307647008	Malignant lymphoma, centroblastic type, follicular	Cancer
287161	SNOMED CT	307649006	Primary central nervous system lymphoma	Cancer
287162	SNOMED CT	307650006	Histiocytic medullary reticulosis	Cancer
288483	SNOMED CT	309245001	Adenocarcinoma of uterus	Cancer
389317	SNOMED CT	699657009	Hepatosplenic gamma-delta cell lymphoma	Cancer
389466	SNOMED CT	699818003	T-cell large granular lymphocytosis	Cancer
391911	SNOMED CT	702446006	Core binding factor acute myeloid leukemia	Cancer
391939	SNOMED CT	702476004	Therapy-related myelodysplastic syndrome	Cancer
392235	SNOMED CT	702785000	Large cell (Ki-1 positive) lymphoma	Cancer

392236	SNOMED CT	702786004	Follicular non-Hodgkin's lymphoma diffuse follicle center sub-type grade 1	Cancer
392422	SNOMED CT	702977001	Follicular non-Hodgkin's lymphoma diffuse follicle center cell sub-type grade 2	Cancer
392829	SNOMED CT	703387000	Cytogenetically normal acute myeloid leukemia	Cancer
393056	SNOMED CT	703626001	Anaplastic large cell lymphoma, T/Null cell, primary systemic type	Cancer
394372	SNOMED CT	705061009	Childhood myelodysplastic syndrome	Cancer
398202	SNOMED CT	709471005	Periodontitis co-occurrent with leukemia	Cancer
401079	SNOMED CT	713325002	Primary cerebral lymphoma co-occurrent with human immunodeficiency virus infection	Cancer
401229	SNOMED CT	713483007	Reticulosarcoma co-occurrent with human immunodeficiency virus infection	Cancer
401260	SNOMED CT	713516007	Primary effusion lymphoma	Cancer
401458	SNOMED CT	713718006	Diffuse non-Hodgkin immunoblastic lymphoma co-occurrent with human immunodeficiency virus infection	Cancer
401635	SNOMED CT	713897006	Burkitt lymphoma co-occurrent with human immunodeficiency virus infection	Cancer
401986	SNOMED CT	714251006	Philadelphia chromosome-negative precursor B-cell acute lymphoblastic leukemia	Cancer
402196	SNOMED CT	714463003	Primary effusion lymphoma co-occurrent with infection caused by Human herpesvirus 8	Cancer
403230	SNOMED CT	715664005	Interdigitating dendritic cell sarcoma	Cancer
403481	SNOMED CT	715950008	ALK-positive large B-cell lymphoma	Cancer
404204	SNOMED CT	716788007	Epstein-Barr virus positive diffuse large B-cell lymphoma of elderly	Cancer
405258	SNOMED CT	718200007	Primary pulmonary lymphoma	Cancer
407862	SNOMED CT	721302006	Myelodysplastic/myeloproliferative neoplasm with ring sideroblasts and thrombocytosis	Cancer
407863	SNOMED CT	721303001	Refractory neutropenia	Cancer

407864	SNOMED CT	721304007	Refractory thrombocytopenia	Cancer
407865	SNOMED CT	721305008	Acute myeloid leukemia due to recurrent genetic abnormality	Cancer
407866	SNOMED CT	721306009	Therapy related acute myeloid leukemia and myelodysplastic syndrome	Cancer
407868	SNOMED CT	721308005	Acute leukemia of ambiguous lineage	Cancer
407869	SNOMED CT	721310007	Aggressive natural killer-cell leukemia	Cancer
407871	SNOMED CT	721313009	Indeterminate dendritic cell neoplasm	Cancer
407872	SNOMED CT	721314003	Fibroblastic reticular cell neoplasm	Cancer
408112	SNOMED CT	721555001	Follicular lymphoma of small intestine	Cancer
408248	SNOMED CT	721695008	Primary adenocarcinoma of ascending colon and right flexure	Cancer
408249	SNOMED CT	721696009	Primary adenocarcinoma of transverse colon	Cancer
408252	SNOMED CT	721699002	Primary adenocarcinoma of descending colon and splenic flexure	Cancer
408315	SNOMED CT	721762007	Adult T-cell leukemia/lymphoma of skin	Cancer
409175	SNOMED CT	722795004	Meningeal leukemia	Cancer
409312	SNOMED CT	722953004	B-cell lymphoma unclassifiable with features intermediate between Burkitt lymphoma and diffuse large B-cell lymphoma	Cancer
409313	SNOMED CT	722954005	B-cell lymphoma unclassifiable with features intermediate between classical Hodgkin lymphoma and diffuse large B-cell lymphoma	Cancer
410008	SNOMED CT	723889003	B lymphoblastic leukemia lymphoma with t(9:22)(q34;q11.2); BCR-ABL 1	Cancer
410550	SNOMED CT	724644005	Myeloid leukemia co-occurrent with Down syndrome	Cancer
410551	SNOMED CT	724645006	T-cell histiocyte rich large B-cell lymphoma	Cancer
410552	SNOMED CT	724647003	Diffuse large B-cell lymphoma co-occurrent with chronic inflammation caused by Epstein-Barr virus	Cancer
410553	SNOMED CT	724648008	Plasmablastic lymphoma	Cancer
410554	SNOMED CT	724649000	Langerhans cell sarcoma	Cancer

410555	SNOMED CT	724650000	Primary follicular dendritic cell sarcoma	Cancer
411108	SNOMED CT	725390002	Acute myeloid leukemia with t(8;16)(p11;p13) translocation	Cancer
411146	SNOMED CT	725437002	Chronic lymphocytic leukemia genetic mutation variant	Cancer
412055	SNOMED CT	726721002	Nodal marginal zone B-cell lymphoma	Cancer
418689	SNOMED CT	733598001	Acute myeloid leukemia with t(6;9)(p23;q34) translocation	Cancer
419008	SNOMED CT	734066005	Diffuse large B-cell lymphoma of central nervous system	Cancer
419344	SNOMED CT	734522002	Acute myeloid leukemia with FMS-like tyrosine kinase-3 mutation	Cancer
419908	SNOMED CT	735332000	Primary cutaneous diffuse large cell B-cell lymphoma leg type	Cancer
420721	SNOMED CT	736322001	Pediatric follicular lymphoma	Cancer
421234	SNOMED CT	737058005	Microsatellite instability-high colorectal cancer	Cancer
421772	SNOMED CT	738527001	Myeloid and/or lymphoid neoplasm associated with platelet derived growth factor receptor alpha rearrangement	Cancer
422416	SNOMED CT	762315004	Therapy related acute myeloid leukemia due to and following administration of antineoplastic agent	Cancer
422739	SNOMED CT	762690000	Classical Hodgkin lymphoma	Cancer
423095	SNOMED CT	763309005	Acute myeloid leukemia with NPM1 somatic mutation	Cancer
423235	SNOMED CT	763477007	Primary lymphoma of conjunctiva	Cancer
423396	SNOMED CT	763666008	Splenic marginal zone B-cell lymphoma	Cancer
423431	SNOMED CT	763719001	Hydroa vacciniforme-like lymphoma	Cancer
423480	SNOMED CT	763796007	Megakaryoblastic acute myeloid leukemia with t(1;22)(p13;q13)	Cancer
423549	SNOMED CT	763884007	Splenic diffuse red pulp small B-cell lymphoma	Cancer
424189	SNOMED CT	764855007	Acute myeloid leukemia with CEBPA somatic mutations	Cancer
424262	SNOMED CT	764940002	Inherited acute myeloid leukemia	Cancer
424418	SNOMED CT	765136002	Primary cutaneous CD8 positive aggressive epidermotropic cytotoxic T-cell lymphoma	Cancer

424568	SNOMED CT	765328000	Classic mycosis fungoides	Cancer
424939	SNOMED CT	766045006	Acute myeloid leukemia and myelodysplastic syndrome related to alkylating agent	Cancer
424940	SNOMED CT	766046007	Acute myeloid leukemia and myelodysplastic syndrome related to topoisomerase type 2 inhibitor	Cancer
424942	SNOMED CT	766048008	Acute myeloid leukemia and myelodysplastic syndrome related to radiation	Cancer
425472	SNOMED CT	766935007	Primary bone lymphoma	Cancer
425513	SNOMED CT	766981007	Squamous cell carcinoma of colon	Cancer
428184	SNOMED CT	770402000	Aleukemic mast cell leukemia	Cancer
430514	SNOMED CT	773537001	Differentiation syndrome due to and following chemotherapy co-occurrent with acute promyelocytic leukemia	Cancer
430868	SNOMED CT	773995001	Primary cutaneous anaplastic large cell lymphoma	Cancer
437436	SNOMED CT	780844005	Acute myeloid leukemia with inv(3)(q21q26.2) or t(3;3)(q21;q26.2); RPN1-EVI1	Cancer
439345	SNOMED CT	783541009	Breast implant-associated anaplastic large-cell lymphoma	Cancer
439360	SNOMED CT	783565007	Indolent T-cell lymphoproliferative disorder of gastrointestinal tract	Cancer
439395	SNOMED CT	783615009	Erythropoietic uroporphyria associated with myeloid malignancy	Cancer
442911	SNOMED CT	788874003	B-cell prolymphocytic leukemia in remission	Cancer
443008	SNOMED CT	788972003	Juvenile myelomonocytic leukemia in remission	Cancer
444376	SNOMED CT	830057003	Relapsing classical Hodgkin lymphoma	Cancer
444764	SNOMED CT	836486002	Lymphomatous infiltrate of kidney	Cancer
444836	SNOMED CT	838340006	B lymphoblastic leukemia lymphoma with t(5;14)(q31;q32); IL3-IGH	Cancer
444837	SNOMED CT	838341005	B lymphoblastic leukemia lymphoma with t(v;11q23); MLL rearranged	Cancer

444838	SNOMED CT	838342003	B lymphoblastic leukemia lymphoma with t(12;21)(p13;q22); TEL/AML1 (ETV6-RUNX1)	Cancer
444839	SNOMED CT	838343008	B lymphoblastic leukemia lymphoma with t(1;19)(Q23;P13.3); E2A-PBX1 (TCF3/PBX1)	Cancer
444840	SNOMED CT	838344002	B lymphoblastic leukemia lymphoma with hypodiploidy	Cancer
444842	SNOMED CT	838346000	B lymphoblastic leukemia lymphoma with hyperdiploidy	Cancer
444851	SNOMED CT	838355002	Acute myeloid leukemia with inv(16)(p13.1q22) or t(16;16)(p13.1;q22) CBFB-MYH11	Cancer
445137	SNOMED CT	840423002	Diffuse large B-cell lymphoma of small intestine	Cancer
445138	SNOMED CT	840424008	Diffuse large B-cell lymphoma of stomach	Cancer
446146	SNOMED CT	866098005	Large B-cell lymphoma arising in HHV8-associated multicentric Castleman disease	Cancer
447895	SNOMED CT	1.701E+12	Primary adenocarcinoma of colon	Cancer
449530	SNOMED CT	1.228E+13	Relapsing acute myeloid leukemia	Cancer
449531	SNOMED CT	1.229E+13	Refractory acute myeloid leukemia	Cancer
449532	SNOMED CT	1.23E+13	Acute lymphoid leukemia relapse	Cancer
449534	SNOMED CT	1.231E+13	Refractory acute lymphoid leukemia	Cancer
450577	SNOMED CT	6.129E+13	Disorder of central nervous system co-occurrent and due to acute lymphoid leukemia in remission	Cancer
450578	SNOMED CT	6.13E+13	Disorder of central nervous system co-occurrent and due to acute lymphoid leukemia	Cancer
450579	SNOMED CT	6.131E+13	Acute myeloid leukemia, minimal differentiation, FAB M0 in remission	Cancer
450580	SNOMED CT	6.132E+13	Acute myeloid leukemia without maturation, FAB M1 in remission	Cancer
450710	SNOMED CT	6.826E+13	Diffuse non-Hodgkin's lymphoma Lugano stage I	Cancer
450711	SNOMED CT	6.827E+13	Diffuse non-Hodgkin's lymphoma Lugano stage II	Cancer
450712	SNOMED CT	6.829E+13	Diffuse non-Hodgkin's lymphoma Lugano stage IV	Cancer

450913	SNOMED CT	8.481E+13	Follicular dendritic sarcoma of intraabdominal lymph node	Cancer
450914	SNOMED CT	8.483E+13	Follicular dendritic sarcoma of lymph nodes of multiple sites	Cancer
451091	SNOMED CT	9.628E+13	Overlapping malignant neoplasm of colon and rectum	Cancer
451105	SNOMED CT	9.698E+13	Malignant neoplasm of rectosigmoid junction metastatic to brain	Cancer
451471	SNOMED CT	1.167E+14	Marginal zone lymphoma of spleen	Cancer
451472	SNOMED CT	1.167E+14	Marginal zone lymphoma of inguinal lymph node	Cancer
451473	SNOMED CT	1.167E+14	Marginal zone lymphoma of thoracic lymph node	Cancer
451474	SNOMED CT	1.168E+14	Non-Hodgkin lymphoma of central nervous system metastatic to lymph node of lower limb	Cancer
451475	SNOMED CT	1.168E+14	Non-Hodgkin lymphoma of central nervous system metastatic to lymph node of upper limb	Cancer
451476	SNOMED CT	1.168E+14	Marginal zone lymphoma of lymph nodes of multiple sites	Cancer
451477	SNOMED CT	1.169E+14	Mantle cell lymphoma of lymph nodes of multiple sites	Cancer
451482	SNOMED CT	1.171E+14	Peripheral T-cell lymphoma of spleen	Cancer
451483	SNOMED CT	1.171E+14	Peripheral T-cell lymphoma of axillary lymph node	Cancer
451484	SNOMED CT	1.171E+14	Peripheral T-cell lymphoma of thoracic lymph node	Cancer
451485	SNOMED CT	1.172E+14	Peripheral T-cell lymphoma of lymph nodes of multiple sites	Cancer
451605	SNOMED CT	1.229E+14	Chronic monocytic leukemia in relapse	Cancer
451606	SNOMED CT	1.229E+14	Myeloid leukemia in relapse	Cancer
451607	SNOMED CT	1.23E+14	Lymphoid leukemia in relapse	Cancer
451608	SNOMED CT	1.23E+14	Chronic lymphoid leukemia in relapse	Cancer
451609	SNOMED CT	1.23E+14	Plasma cell leukemia in relapse	Cancer
451637	SNOMED CT	1.238E+14	Chronic leukemia in relapse	Cancer
451728	SNOMED CT	1.28E+14	Aleukemic myeloid leukemia in relapse	Cancer

451781	SNOMED CT	1.304E+14	Primary malignant neuroendocrine neoplasm of ascending colon	Cancer
451863	SNOMED CT	1.338E+14	Lymphoma of colon	Cancer
452266	SNOMED CT	1.849E+14	Primary adenocarcinoma of rectosigmoid junction	Cancer
454382	SNOMED CT	3.51E+14	B-cell lymphoma of intra-abdominal lymph nodes	Cancer
454385	SNOMED CT	3.512E+14	B-cell lymphoma of lymph nodes of multiple sites	Cancer
454414	SNOMED CT	3.523E+14	Small lymphocytic B-cell lymphoma of lymph nodes of multiple sites	Cancer
454418	SNOMED CT	3.524E+14	Small lymphocytic B-cell lymphoma of intra-abdominal lymph nodes	Cancer
454419	SNOMED CT	3.528E+14	Non-Hodgkin's lymphoma of lymph nodes of multiple sites	Cancer
454465	SNOMED CT	3.549E+14	Follicular non-Hodgkin's lymphoma of lymph nodes of multiple sites	Cancer
456737	SNOMED CT	4.505E+14	Marginal zone lymphoma of axillary lymph node	Cancer
457333	SNOMED CT	4.569E+14	Diffuse non-Hodgkin's lymphoma Lugano stage III	Cancer
457722	SNOMED CT	4.613E+14	Refractory Hodgkin's lymphoma	Cancer
458064	SNOMED CT	6.816E+14	Primary adenocarcinoma of ascending colon	Cancer
494106	SNOMED CT	53132006	Excessive gastrin secretion	Cancer
496739	SNOMED CT	92516002	Burkitt's tumor of unspecified, extranodal, or solid organ site	Cancer
496758	SNOMED CT	93451002	Erythroleukaemia, FAB M6	Cancer
496759	SNOMED CT	93487009	Hodgkin disease, lymphocytic depletion of lymph nodes of axilla AND/OR upper limb	Cancer
496760	SNOMED CT	93488004	Hodgkin disease, lymphocytic depletion of lymph nodes of head, face AND/OR neck	Cancer
496762	SNOMED CT	93489007	Hodgkin disease, lymphocytic depletion of lymph nodes of inguinal region AND/OR lower limb	Cancer
496763	SNOMED CT	93492006	Hodgkin disease, lymphocytic depletion of extranodal AND/OR solid organ site	Cancer
496764	SNOMED CT	93493001	Hodgkin's paragranuloma of intra-abdominal lymph nodes	Cancer

496767	SNOMED CT	93494007	Hodgkin disease, lymphocytic-histiocytic predominance of intrapelvic lymph nodes	Cancer
496768	SNOMED CT	93495008	Hodgkin disease, lymphocytic-histiocytic predominance of intrathoracic lymph nodes	Cancer
496769	SNOMED CT	93496009	Hodgkin disease, lymphocytic-histiocytic predominance of lymph nodes of axilla AND/OR upper limb	Cancer
496770	SNOMED CT	93497000	Hodgkin disease, lymphocytic-histiocytic predominance of lymph nodes of head, face AND/OR neck	Cancer
496771	SNOMED CT	93498005	Hodgkin disease, lymphocytic-histiocytic predominance of lymph nodes of inguinal region AND/OR lower limb	Cancer
496772	SNOMED CT	93500006	Hodgkin paragranuloma of spleen	Cancer
496773	SNOMED CT	93501005	Hodgkin disease, lymphocytic-histiocytic predominance of extranodal AND/OR solid organ site	Cancer
496774	SNOMED CT	93505001	Hodgkin disease, mixed cellularity of lymph nodes of axilla AND/OR upper limb	Cancer
496775	SNOMED CT	93506000	Hodgkin disease, mixed cellularity of lymph nodes of head, face AND/OR neck	Cancer
496776	SNOMED CT	93507009	Hodgkin disease, mixed cellularity of lymph nodes of inguinal region AND/OR lower limb	Cancer
496777	SNOMED CT	93509007	Hodgkin disease, mixed cellularity of spleen	Cancer
496778	SNOMED CT	93510002	Hodgkin disease, mixed cellularity of extranodal AND/OR solid organ site	Cancer
496779	SNOMED CT	93514006	Hodgkin disease, nodular sclerosis of lymph nodes of axilla AND/OR upper limb	Cancer
496780	SNOMED CT	93515007	Hodgkin disease, nodular sclerosis of lymph nodes of head, face AND/OR neck	Cancer
496781	SNOMED CT	93516008	Hodgkin disease, nodular sclerosis of lymph nodes of inguinal region AND/OR lower limb	Cancer
496782	SNOMED CT	93518009	Hodgkin disease, nodular sclerosis of spleen	Cancer

496783	SNOMED CT	93519001	Hodgkin disease, nodular sclerosis of extranodal AND/OR solid organ site	Cancer
496784	SNOMED CT	93520007	Hodgkin disease of intra-abdominal lymph nodes	Cancer
496785	SNOMED CT	93521006	Hodgkin disease of intrapelvic lymph nodes	Cancer
496786	SNOMED CT	93522004	Hodgkin disease of intrathoracic lymph nodes	Cancer
496787	SNOMED CT	93523009	Hodgkin disease of lymph nodes of axilla AND/OR upper limb	Cancer
496788	SNOMED CT	93524003	Hodgkin disease of lymph nodes of head, face AND/OR neck	Cancer
496790	SNOMED CT	93525002	Hodgkin disease of lymph nodes of inguinal region AND/OR lower limb	Cancer
496791	SNOMED CT	93526001	Hodgkin disease of lymph nodes of multiple sites	Cancer
496792	SNOMED CT	93527005	Hodgkin disease of spleen	Cancer
496793	SNOMED CT	93528000	Hodgkin disease of extranodal AND/OR solid organ site	Cancer
496794	SNOMED CT	93530003	Hodgkin granuloma of intrapelvic lymph nodes	Cancer
496795	SNOMED CT	93531004	Hodgkin granuloma of intrathoracic lymph nodes	Cancer
496796	SNOMED CT	93532006	Hodgkin granuloma of lymph nodes of axilla AND/OR upper limb	Cancer
496797	SNOMED CT	93533001	Hodgkin granuloma of lymph nodes of head, face AND/OR neck	Cancer
496798	SNOMED CT	93534007	Hodgkin granuloma of lymph nodes of inguinal region AND/OR lower limb	Cancer
496799	SNOMED CT	93536009	Hodgkin granuloma of spleen	Cancer
496800	SNOMED CT	93537000	Hodgkin granuloma of extranodal AND/OR solid organ site	Cancer
496801	SNOMED CT	93541001	Hodgkin paragranuloma of lymph nodes of axilla AND/OR upper limb	Cancer
496802	SNOMED CT	93542008	Hodgkin paragranuloma of lymph nodes of head, face AND/OR neck	Cancer
496803	SNOMED CT	93543003	Hodgkin paragranuloma of lymph nodes of inguinal region AND/OR lower limb	Cancer

496807	SNOMED CT	93546006	Hodgkin paragranuloma of extranodal AND/OR solid organ site	Cancer
496808	SNOMED CT	93547002	Hodgkin sarcoma of intra-abdominal lymph nodes	Cancer
496809	SNOMED CT	93548007	Hodgkin sarcoma of intrapelvic lymph nodes	Cancer
496810	SNOMED CT	93549004	Hodgkin sarcoma of intrathoracic lymph nodes	Cancer
496811	SNOMED CT	93550004	Hodgkin sarcoma of lymph nodes of axilla AND/OR upper limb	Cancer
496812	SNOMED CT	93551000	Hodgkin sarcoma of lymph nodes of head, face AND/OR neck	Cancer
496813	SNOMED CT	93552007	Hodgkin sarcoma of lymph nodes of inguinal region AND/OR lower limb	Cancer
496814	SNOMED CT	93554008	Hodgkin sarcoma of spleen	Cancer
496815	SNOMED CT	93555009	Hodgkin sarcoma of extranodal AND/OR solid organ site	Cancer
496821	SNOMED CT	94144008	Primary malignant neoplasm of Waldeyer ring	Cancer
496825	SNOMED CT	94719007	Granulocytic sarcoma	Cancer
497604	SNOMED CT	109350007	Lipoma of trunk	Cancer
497607	SNOMED CT	109388009	Kaposi sarcoma of palate	Cancer
497609	SNOMED CT	109390005	Kaposi sarcoma of lung	Cancer
497625	SNOMED CT	109962001	Nonfollicular lymphoma	Cancer
497626	SNOMED CT	109964000	Diffuse non-Hodgkin lymphoma, undifferentiated	Cancer
497627	SNOMED CT	109965004	Lymphoblastoma	Cancer
497628	SNOMED CT	109966003	Malignant lymphoma - immunoblastic	Cancer
497630	SNOMED CT	109967007	Diffuse non-Hodgkin lymphoma, small cleaved cell	Cancer
497631	SNOMED CT	109968002	Diffuse non-Hodgkin lymphoma, small cell	Cancer
497633	SNOMED CT	109969005	DLBCL - diffuse large B cell lymphoma	Cancer
497634	SNOMED CT	109970006	Follicular lymphoma, grade 1	Cancer
497635	SNOMED CT	109971005	Follicular lymphoma grade 2	Cancer
497636	SNOMED CT	109972003	Follicular lymphoma grade 3	Cancer

497637	SNOMED CT	109977009	Peripheral T-cell lymphoma pleomorphic small cell	Cancer
497638	SNOMED CT	109979007	B-cell lymphoma	Cancer
497642	SNOMED CT	110004001	Acute promyelocytic leukemia (clinical)	Cancer
497643	SNOMED CT	110005000	Acute myelomonocytic leukemia (clinical)	Cancer
497644	SNOMED CT	110007008	Adult T-cell leukemia	Cancer
498772	SNOMED CT	118599009	Lymphoma, Hodgkins	Cancer
498773	SNOMED CT	118600007	Malignant lymphoma (clinical)	Cancer
498776	SNOMED CT	118601006	Non-Hodgkin lymphoma	Cancer
498777	SNOMED CT	118602004	Hodgkin granuloma	Cancer
498778	SNOMED CT	118605002	Hodgkin lymphoma, nodular lymphocyte predominance	Cancer
498779	SNOMED CT	118606001	Hodgkin sarcoma	Cancer
498780	SNOMED CT	118608000	Hodgkin disease, nodular sclerosis	Cancer
498781	SNOMED CT	118609008	Hodgkin mixed cellularity lymphoma	Cancer
498782	SNOMED CT	118610003	Hodgkin disease, lymphocytic depletion	Cancer
498783	SNOMED CT	118613001	Leukemic reticuloendotheliosis	Cancer
498784	SNOMED CT	118614007	Acute infancy reticulosis	Cancer
498785	SNOMED CT	118617000	Burkitt lymphoma	Cancer
499140	SNOMED CT	128875000	Primary cutaneous CD30 antigen positive large T-cell lymphoma	Cancer
500367	SNOMED CT	187716008	Malignant tumour of Waldeyer ring	Cancer
500369	SNOMED CT	187734007	Malignant neoplasm of gastroesophageal junction of stomach	Cancer
500377	SNOMED CT	188511002	Burkitt tumor of intrathoracic lymph nodes	Cancer
500379	SNOMED CT	188512009	Burkitt tumor of intra-abdominal lymph nodes	Cancer
500381	SNOMED CT	188513004	Burkitt lymphoma of lymph nodes of axilla and upper limb	Cancer
500382	SNOMED CT	188514005	Burkitt lymphoma of lymph nodes of inguinal region and lower limb	Cancer
500383	SNOMED CT	188515006	Burkitt tumor of intrapelvic lymph nodes	Cancer

500385	SNOMED CT	188516007	Burkitt tumor of spleen	Cancer
500387	SNOMED CT	188517003	Burkitt tumor of lymph nodes of multiple sites	Cancer
500389	SNOMED CT	188524002	Hodgkin paragranuloma of intrathoracic lymph nodes	Cancer
500394	SNOMED CT	188529007	Hodgkin paragranuloma of intrapelvic lymph nodes	Cancer
500396	SNOMED CT	188531003	Hodgkin paragranuloma of lymph nodes of multiple sites	Cancer
500400	SNOMED CT	188536008	Hodgkin granuloma of intra-abdominal lymph nodes	Cancer
500401	SNOMED CT	188537004	Hodgkin granuloma of lymph nodes of axilla and upper limb	Cancer
500402	SNOMED CT	188538009	Hodgkin granuloma of lymph nodes of inguinal region and lower limb	Cancer
500403	SNOMED CT	188541000	Hodgkin granuloma of lymph nodes of multiple sites	Cancer
500406	SNOMED CT	188547001	Hodgkin sarcoma of lymph nodes of axilla and upper limb	Cancer
500407	SNOMED CT	188548006	Hodgkin sarcoma of lymph nodes of inguinal region and lower limb	Cancer
500408	SNOMED CT	188551004	Hodgkin sarcoma of lymph nodes of multiple sites	Cancer
500410	SNOMED CT	188554007	Hodgkin disease, lymphocytic-histiocytic predominance of lymph nodes of head, face and neck	Cancer
500411	SNOMED CT	188558005	Hodgkin disease, lymphocytic-histiocytic predominance of lymph nodes of axilla and upper limb	Cancer
500412	SNOMED CT	188559002	Hodgkin disease, lymphocytic-histiocytic predominance of lymph nodes of inguinal region and lower limb	Cancer
500413	SNOMED CT	188562004	Hodgkin disease, lymphocytic-histiocytic predominance of lymph nodes of multiple sites	Cancer
500414	SNOMED CT	188565002	Hodgkin disease, nodular sclerosis of lymph nodes of head, face and neck	Cancer
500415	SNOMED CT	188566001	Hodgkin disease, nodular sclerosis of intrathoracic lymph nodes	Cancer
500416	SNOMED CT	188567005	Hodgkin disease, nodular sclerosis of intra-abdominal lymph nodes	Cancer

500417	SNOMED CT	188568000	Hodgkin disease, nodular sclerosis of lymph nodes of axilla and upper limb	Cancer
500418	SNOMED CT	188569008	Hodgkin disease, nodular sclerosis of lymph nodes of inguinal region and lower limb	Cancer
500419	SNOMED CT	188570009	Hodgkin disease, nodular sclerosis of intrapelvic lymph nodes	Cancer
500420	SNOMED CT	188572001	Hodgkin disease, nodular sclerosis of lymph nodes of multiple sites	Cancer
500421	SNOMED CT	188575004	Hodgkin disease, mixed cellularity of lymph nodes of head, face and neck	Cancer
500422	SNOMED CT	188576003	Hodgkin disease, mixed cellularity of intrathoracic lymph nodes	Cancer
500423	SNOMED CT	188577007	Hodgkin disease, mixed cellularity of intra-abdominal lymph nodes	Cancer
500424	SNOMED CT	188578002	Hodgkin disease, mixed cellularity of lymph nodes of axilla and upper limb	Cancer
500425	SNOMED CT	188579005	Hodgkin disease, mixed cellularity of lymph nodes of inguinal region and lower limb	Cancer
500426	SNOMED CT	188580008	Hodgkin disease, mixed cellularity of intrapelvic lymph nodes	Cancer
500427	SNOMED CT	188582000	Hodgkin disease, mixed cellularity of lymph nodes of multiple sites	Cancer
500428	SNOMED CT	188585003	Hodgkin disease, lymphocytic depletion of lymph nodes of head, face and neck	Cancer
500429	SNOMED CT	188586002	Hodgkin disease, lymphocytic depletion of intrathoracic lymph nodes	Cancer
500430	SNOMED CT	188587006	Hodgkin disease, lymphocytic depletion of intra-abdominal lymph nodes	Cancer
500431	SNOMED CT	188589009	Hodgkin disease, lymphocytic depletion of lymph nodes of axilla and upper limb	Cancer
500432	SNOMED CT	188590000	Hodgkin disease, lymphocytic depletion of lymph nodes of inguinal region and lower limb	Cancer
500433	SNOMED CT	188591001	Hodgkin disease, lymphocytic depletion of intrapelvic lymph nodes	Cancer

500434	SNOMED CT	188592008	Hodgkin disease, lymphocytic depletion of spleen	Cancer
500435	SNOMED CT	188593003	Hodgkin disease, lymphocytic depletion of lymph nodes of multiple sites	Cancer
500436	SNOMED CT	188632001	Lymphoma intra abdominal, Sezarys	Cancer
500437	SNOMED CT	188672005	Follicular non-Hodgkin mixed small cleaved and large cell lymphoma	Cancer
500438	SNOMED CT	188679001	Diffuse non-Hodgkin lymphoma undifferentiated (diffuse)	Cancer
501333	SNOMED CT	231830001	Bowen disease of eyelid	Cancer
501335	SNOMED CT	231835006	Kaposi sarcoma of eyelid	Cancer
501569	SNOMED CT	240531002	African Burkitt lymphoma	Cancer
501925	SNOMED CT	255114007	Kaposi sarcoma of conjunctiva	Cancer
501927	SNOMED CT	255115008	Kaposi sarcoma of cornea	Cancer
501931	SNOMED CT	255121007	Peritoneal carcinomatosis	Cancer
502620	SNOMED CT	276876007	Carcinoma of Bartholin gland	Cancer
502642	SNOMED CT	277550009	Richter syndrome	Cancer
502645	SNOMED CT	277609007	Hodgkin disease, lymphocytic predominance - diffuse	Cancer
502646	SNOMED CT	277610002	Hodgkin disease, nodular sclerosis - lymphocytic predominance	Cancer
502647	SNOMED CT	277611003	Hodgkin disease, nodular sclerosis - mixed cellularity	Cancer
502648	SNOMED CT	277612005	Hodgkin disease, nodular sclerosis - lymphocytic depletion	Cancer
502649	SNOMED CT	277654008	Enteropathy-type T-cell lymphoma	Cancer
503284	SNOMED CT	307608006	Ewing sarcoma of bone	Cancer
503286	SNOMED CT	307633009	Hodgkin disease, lymphocytic depletion, diffuse fibrosis	Cancer
503287	SNOMED CT	307634003	Hodgkin disease, lymphocytic depletion, reticular type	Cancer
503288	SNOMED CT	307635002	Hodgkin disease, nodular sclerosis - cellular phase	Cancer
503296	SNOMED CT	308121000	Follicular non-Hodgkin lymphoma	Cancer
503511	SNOMED CT	315058005	Lynch syndrome	Cancer

507688	SNOMED CT	359631009	M0 - Acute myeloblastic leukemia - undifferentiated	Cancer
507690	SNOMED CT	359640008	Acute myelogenous leukemia without maturation	Cancer
507693	SNOMED CT	359648001	M2 - Acute myeloblastic leukemia with maturation	Cancer
507769	SNOMED CT	363510005	CA - Cancer of large bowel	Cancer
508366	SNOMED CT	372131006	Ca upper limb bones/scapula	Cancer
511622	SNOMED CT	402881008	Cutaneous B-cell lymphoma	Cancer
511811	SNOMED CT	404106004	Lymphomatoid papulosis with Hodgkin disease	Cancer
511812	SNOMED CT	404122003	Leukaemic infiltration of skin (chronic T-cell lymphocytic leukaemia)	Cancer
511813	SNOMED CT	404123008	Leukaemic infiltration of skin (T-cell prolymphocytic leukaemia)	Cancer
511814	SNOMED CT	404124002	Leukaemic infiltration of skin (T-cell lymphoblastic leukaemia)	Cancer
511815	SNOMED CT	404135007	Angiocentric lymphoma involving skin	Cancer
511817	SNOMED CT	404136008	Aggressive natural killer-cell leukemia involving skin	Cancer
511819	SNOMED CT	404143002	Crosti's lymphoma	Cancer
511821	SNOMED CT	404157000	Specific skin infiltration in Hodgkin disease	Cancer
512852	SNOMED CT	414166008	Extranodal natural killer/T-cell lymphoma, nasal type	Cancer
512952	SNOMED CT	414780005	Mucosa-associated lymphoid tissue lymphoma of orbit	Cancer
513392	SNOMED CT	420302007	Reticulosarcoma with AIDS (acquired immunodeficiency syndrome)	Cancer
513521	SNOMED CT	421283008	Primary lymphoma of brain with AIDS (acquired immunodeficiency syndrome)	Cancer
513761	SNOMED CT	424779008	Primary Kaposi sarcoma of oral cavity	Cancer
514925	SNOMED CT	445269007	Extranodal marginal zone B-cell lymphoma of mucosa-associated lymphoid tissue	Cancer
515664	SNOMED CT	733627006	Primary cutaneous gamma-delta T-cell lymphoma	Cancer
72107	SNOMED CT	76564002	Catecholamine secretion by pheochromocytoma	Cancer
76205	SNOMED CT	80914001	Anaplasia of cervix	Cancer

76303	SNOMED CT	81021006	Myoma of prostate	Cancer
86531	SNOMED CT	91854005	Acute leukemia in remission	Cancer
86532	SNOMED CT	91855006	Acute leukemia	Cancer
86533	SNOMED CT	91856007	Acute lymphoid leukemia in remission	Cancer
86534	SNOMED CT	91857003	Acute lymphoid leukemia	Cancer
86535	SNOMED CT	91858008	Acute monocytic leukemia in remission	Cancer
86536	SNOMED CT	91860005	Acute myeloid leukemia in remission	Cancer
86537	SNOMED CT	91861009	Acute myeloid leukemia, disease	Cancer
86761	SNOMED CT	92087008	Benign neoplasm of epicardium	Cancer
87412	SNOMED CT	92752000	Carcinoma in situ of spermatic cord	Cancer
87453	SNOMED CT	92793008	Carcinoma in situ of vas deferens	Cancer
87470	SNOMED CT	92811003	Chronic leukemia in remission	Cancer
87471	SNOMED CT	92812005	Chronic leukemia	Cancer
87472	SNOMED CT	92813000	Chronic lymphoid leukemia in remission	Cancer
87473	SNOMED CT	92814006	Chronic lymphoid leukemia, disease	Cancer
87476	SNOMED CT	92817004	Chronic myeloid leukemia in remission	Cancer
87477	SNOMED CT	92818009	Chronic myeloid leukemia	Cancer
87788	SNOMED CT	93133006	Letterer-Siwe disease of intra-abdominal lymph nodes	Cancer
87789	SNOMED CT	93134000	Letterer-Siwe disease of intrapelvic lymph nodes	Cancer
87790	SNOMED CT	93135004	Letterer-Siwe disease of intrathoracic lymph nodes	Cancer
87791	SNOMED CT	93136003	Letterer-Siwe disease of lymph nodes of axilla AND/OR upper limb	Cancer
87792	SNOMED CT	93137007	Letterer-Siwe disease of lymph nodes of head, face AND/OR neck	Cancer
87793	SNOMED CT	93138002	Letterer-Siwe disease of lymph nodes of inguinal region AND/OR lower limb	Cancer
87794	SNOMED CT	93139005	Letterer-Siwe disease of lymph nodes of multiple sites	Cancer

87795	SNOMED CT	93140007	Letterer-Siwe disease of spleen	Cancer
87796	SNOMED CT	93141006	Letterer-Siwe disease of extranodal AND/OR solid organ site	Cancer
87797	SNOMED CT	93142004	Leukemia in remission	Cancer
87798	SNOMED CT	93143009	Leukemia	Cancer
87799	SNOMED CT	93144003	Leukemic reticuloendotheliosis of intra-abdominal lymph nodes	Cancer
87800	SNOMED CT	93145002	Leukemic reticuloendotheliosis of intrapelvic lymph nodes	Cancer
87801	SNOMED CT	93146001	Leukemic reticuloendotheliosis of intrathoracic lymph nodes	Cancer
87805	SNOMED CT	93150008	Leukemic reticuloendotheliosis of lymph nodes of multiple sites	Cancer
87806	SNOMED CT	93151007	Hairy cell leukemia of spleen	Cancer
87807	SNOMED CT	93152000	Leukemic reticuloendotheliosis of extranodal AND/OR solid organ site	Cancer
87823	SNOMED CT	93169003	Lymphoid leukemia in remission	Cancer
87836	SNOMED CT	93182006	Malignant histiocytosis of intra-abdominal lymph nodes	Cancer
87837	SNOMED CT	93183001	Malignant histiocytosis of intrapelvic lymph nodes	Cancer
87838	SNOMED CT	93184007	Malignant histiocytosis of intrathoracic lymph nodes	Cancer
87839	SNOMED CT	93185008	Malignant histiocytosis of lymph nodes of axilla AND/OR upper limb	Cancer
87840	SNOMED CT	93186009	Malignant histiocytosis of lymph nodes of head, face AND/OR neck	Cancer
87841	SNOMED CT	93187000	Malignant histiocytosis of lymph nodes of inguinal region AND/OR lower limb	Cancer
87842	SNOMED CT	93188005	Malignant histiocytosis of lymph nodes of multiple sites	Cancer
87843	SNOMED CT	93189002	Malignant histiocytosis of spleen	Cancer
87844	SNOMED CT	93190006	Malignant histiocytosis of extranodal AND/OR solid organ site	Cancer
87845	SNOMED CT	93191005	Malignant lymphoma of intra-abdominal lymph nodes	Cancer
87846	SNOMED CT	93192003	Malignant lymphoma of intrapelvic lymph nodes	Cancer

87847	SNOMED CT	93193008	Malignant lymphoma of intrathoracic lymph nodes	Cancer
87848	SNOMED CT	93194002	Malignant lymphoma of lymph nodes of axilla AND/OR upper limb	Cancer
87849	SNOMED CT	93195001	Malignant lymphoma of lymph nodes of head, face AND/OR neck	Cancer
87850	SNOMED CT	93196000	Malignant lymphoma of lymph nodes of inguinal region AND/OR lower limb	Cancer
87851	SNOMED CT	93197009	Malignant lymphoma of lymph nodes of multiple sites	Cancer
87852	SNOMED CT	93198004	Malignant lymphoma of spleen	Cancer
87853	SNOMED CT	93199007	Malignant lymphoma of extranodal AND/OR solid organ site	Cancer
87854	SNOMED CT	93200005	Malignant mast cell tumor of intra-abdominal lymph nodes	Cancer
87855	SNOMED CT	93201009	Malignant mast cell tumor of intrapelvic lymph nodes	Cancer
87856	SNOMED CT	93202002	Malignant mast cell tumor of intrathoracic lymph nodes	Cancer
87857	SNOMED CT	93203007	Malignant mast cell tumor of lymph nodes of axilla AND/OR upper limb	Cancer
87858	SNOMED CT	93204001	Malignant mast cell tumor of lymph nodes of head, face AND/OR neck	Cancer
87859	SNOMED CT	93205000	Malignant mast cell tumor of lymph nodes of inguinal region AND/OR lower limb	Cancer
87863	SNOMED CT	93209006	Malignant melanoma of perianal skin	Cancer
87864	SNOMED CT	93210001	Malignant melanoma of skin of abdomen	Cancer
87865	SNOMED CT	93211002	Malignant melanoma of skin of ankle	Cancer
87867	SNOMED CT	93213004	Malignant melanoma of skin of axilla	Cancer
87868	SNOMED CT	93214005	Malignant melanoma of skin of back	Cancer
87869	SNOMED CT	93215006	Malignant melanoma of skin of breast	Cancer
87870	SNOMED CT	93216007	Malignant melanoma of skin of buttock	Cancer
87871	SNOMED CT	93217003	Malignant melanoma of skin of cheek	Cancer
87872	SNOMED CT	93218008	Malignant melanoma of skin of chest	Cancer

87873	SNOMED CT	93219000	Malignant melanoma of skin of chin	Cancer
87874	SNOMED CT	93220006	Malignant melanoma of skin of ear	Cancer
87875	SNOMED CT	93221005	Malignant melanoma of skin of elbow	Cancer
87876	SNOMED CT	93222003	Malignant melanoma of skin of external auditory canal	Cancer
87877	SNOMED CT	93223008	Malignant melanoma of skin of eyebrow	Cancer
87878	SNOMED CT	93224002	Malignant melanoma of skin of eyelid	Cancer
87879	SNOMED CT	93225001	Malignant melanoma of skin of face	Cancer
87880	SNOMED CT	93226000	Malignant melanoma of skin of finger	Cancer
87881	SNOMED CT	93227009	Malignant melanoma of skin of foot	Cancer
87882	SNOMED CT	93228004	Malignant melanoma of skin of forearm	Cancer
87883	SNOMED CT	93229007	Malignant melanoma of skin of forehead	Cancer
87884	SNOMED CT	93230002	Malignant melanoma of skin of groin	Cancer
88234	SNOMED CT	93636004	Malignant melanoma of skin of hand	Cancer
88235	SNOMED CT	93637008	Malignant melanoma of skin of hip	Cancer
88236	SNOMED CT	93638003	Malignant melanoma of skin of knee	Cancer
88238	SNOMED CT	93640008	Malignant melanoma of skin of lip	Cancer
88239	SNOMED CT	93641007	Malignant melanoma of skin of lower limb	Cancer
88240	SNOMED CT	93642000	Malignant melanoma of skin of neck	Cancer
88241	SNOMED CT	93643005	Malignant melanoma of skin of nose	Cancer
88242	SNOMED CT	93644004	Malignant melanoma of skin of perineum	Cancer
88243	SNOMED CT	93645003	Malignant melanoma of skin of popliteal area	Cancer
88244	SNOMED CT	93646002	Malignant melanoma of skin of scalp	Cancer
88245	SNOMED CT	93647006	Malignant melanoma of skin of shoulder	Cancer
88246	SNOMED CT	93648001	Malignant melanoma of skin of temporal region	Cancer
88247	SNOMED CT	93649009	Malignant melanoma of skin of thigh	Cancer
88248	SNOMED CT	93650009	Malignant melanoma of skin of toe	Cancer

88249	SNOMED CT	93651008	Malignant melanoma of skin of trunk	Cancer
88250	SNOMED CT	93652001	Malignant melanoma of skin of umbilicus	Cancer
88251	SNOMED CT	93653006	Malignant melanoma of skin of upper limb	Cancer
88252	SNOMED CT	93654000	Malignant melanoma of skin of wrist	Cancer
88253	SNOMED CT	93655004	Malignant melanoma of skin	Cancer
88257	SNOMED CT	93659005	Primary malignant neoplasm of accessory sinus	Cancer
88259	SNOMED CT	93661001	Primary malignant neoplasm of acromion	Cancer
88260	SNOMED CT	93662008	Primary malignant neoplasm of adenoid	Cancer
88263	SNOMED CT	93665005	Primary malignant neoplasm of adrenal gland	Cancer
88265	SNOMED CT	93667002	Primary malignant neoplasm of alveolar ridge mucosa	Cancer
88267	SNOMED CT	93669004	Primary malignant neoplasm of anal canal	Cancer
88268	SNOMED CT	93670003	Primary malignant neoplasm of anterior aspect of epiglottis	Cancer
88269	SNOMED CT	93671004	Primary malignant neoplasm of anterior mediastinum	Cancer
88270	SNOMED CT	93672006	Primary malignant neoplasm of anterior portion of floor of mouth	Cancer
88272	SNOMED CT	93674007	Primary malignant neoplasm of anterior wall of nasopharynx	Cancer
88274	SNOMED CT	93676009	Primary malignant neoplasm of anus	Cancer
88278	SNOMED CT	93680004	Primary malignant neoplasm of areola of female breast	Cancer
88282	SNOMED CT	93684008	Primary malignant neoplasm of axilla	Cancer
88285	SNOMED CT	93687001	Primary malignant neoplasm of base of tongue	Cancer
88287	SNOMED CT	93689003	Primary malignant neoplasm of bladder	Cancer
88290	SNOMED CT	93692004	Primary malignant neoplasm of blood vessel of axilla	Cancer
88308	SNOMED CT	93710000	Primary malignant neoplasm of blood vessel of thorax	Cancer
88314	SNOMED CT	93716006	Primary malignant neoplasm of body of penis	Cancer
88315	SNOMED CT	93717002	Primary malignant neoplasm of body of stomach	Cancer

88319	SNOMED CT	93721009	Primary malignant neoplasm of bone of face	Cancer
88320	SNOMED CT	93722002	Primary malignant neoplasm of bone of lower limb	Cancer
88321	SNOMED CT	93723007	Primary malignant neoplasm of bone of skull	Cancer
88322	SNOMED CT	93724001	Primary malignant neoplasm of bone of upper limb	Cancer
88323	SNOMED CT	93725000	Primary malignant neoplasm of bone	Cancer
88324	SNOMED CT	93726004	Primary malignant neoplasm of brain stem	Cancer
88325	SNOMED CT	93727008	Primary malignant neoplasm of brain	Cancer
88326	SNOMED CT	93728003	Primary malignant neoplasm of broad ligament	Cancer
88327	SNOMED CT	93729006	Primary malignant neoplasm of bronchus of left lower lobe	Cancer
88328	SNOMED CT	93730001	Primary malignant neoplasm of bronchus of left upper lobe	Cancer
88329	SNOMED CT	93731002	Primary malignant neoplasm of bronchus of right lower lobe	Cancer
88330	SNOMED CT	93732009	Primary malignant neoplasm of bronchus of right middle lobe	Cancer
88331	SNOMED CT	93733004	Primary malignant neoplasm of bronchus of right upper lobe	Cancer
88332	SNOMED CT	93734005	Primary malignant neoplasm of bronchus	Cancer
88335	SNOMED CT	93737003	Primary malignant neoplasm of calcaneus	Cancer
88336	SNOMED CT	93738008	Primary malignant neoplasm of cardia of stomach	Cancer
88337	SNOMED CT	93739000	Primary malignant neoplasm of carina	Cancer
88339	SNOMED CT	93741004	Primary malignant neoplasm of carpal bone	Cancer
88340	SNOMED CT	93742006	Primary malignant neoplasm of cartilage of nose	Cancer
88342	SNOMED CT	93744007	Primary malignant neoplasm of central nervous system	Cancer
88343	SNOMED CT	93745008	Primary malignant neoplasm of central portion of female breast	Cancer
88344	SNOMED CT	93746009	Primary malignant neoplasm of cerebellum	Cancer
88345	SNOMED CT	93747000	Primary malignant neoplasm of cerebral meninges	Cancer
88346	SNOMED CT	93748005	Primary malignant neoplasm of cerebral ventricle	Cancer
88347	SNOMED CT	93749002	Primary malignant neoplasm of cerebrum	Cancer

88351	SNOMED CT	93753000	Primary malignant neoplasm of cheek	Cancer
88352	SNOMED CT	93754006	Primary malignant neoplasm of chest wall	Cancer
88353	SNOMED CT	93755007	Primary malignant neoplasm of choroid	Cancer
88354	SNOMED CT	93756008	Primary malignant neoplasm of ciliary body (primary)	Cancer
88355	SNOMED CT	93757004	Primary malignant neoplasm of clavicle	Cancer
88358	SNOMED CT	93760006	Primary malignant neoplasm of coccyx	Cancer
88359	SNOMED CT	93761005	Primary malignant neoplasm of colon	Cancer
88362	SNOMED CT	93764002	Primary malignant neoplasm of conjunctiva of eye	Cancer
88364	SNOMED CT	93766000	Primary malignant neoplasm of cornea of eye	Cancer
88366	SNOMED CT	93768004	Primary malignant neoplasm of craniopharyngeal duct	Cancer
88367	SNOMED CT	93769007	Primary malignant neoplasm of cuboid	Cancer
88369	SNOMED CT	93771007	Primary malignant neoplasm of descending colon	Cancer
88370	SNOMED CT	93772000	Primary malignant neoplasm of diaphragm	Cancer
88371	SNOMED CT	93773005	Primary malignant neoplasm of dorsal surface of tongue	Cancer
88373	SNOMED CT	93775003	Primary malignant neoplasm of duodenum	Cancer
88374	SNOMED CT	93776002	Primary malignant neoplasm of ectopic female breast tissue	Cancer
88376	SNOMED CT	93778001	Primary malignant neoplasm of endocardium	Cancer
88377	SNOMED CT	93779009	Primary malignant neoplasm of endocervix	Cancer
88379	SNOMED CT	93781006	Primary malignant neoplasm of endometrium	Cancer
88380	SNOMED CT	93782004	Primary malignant neoplasm of epicardium	Cancer
88381	SNOMED CT	93783009	Primary malignant neoplasm of epididymis	Cancer
88382	SNOMED CT	93784003	Primary malignant neoplasm of epiglottis	Cancer
88384	SNOMED CT	93786001	Primary malignant neoplasm of ethmoid bone	Cancer
88385	SNOMED CT	93787005	Primary malignant neoplasm of ethmoidal sinus	Cancer
88386	SNOMED CT	93788000	Primary malignant neoplasm of eustachian tube	Cancer

88387	SNOMED CT	93789008	Primary malignant neoplasm of exocervix	Cancer
88394	SNOMED CT	93796005	Primary malignant neoplasm of female breast	Cancer
88395	SNOMED CT	93797001	Primary malignant neoplasm of female genital organ	Cancer
88396	SNOMED CT	93798006	Primary malignant neoplasm of femur	Cancer
88397	SNOMED CT	93799003	Primary malignant neoplasm of fibula	Cancer
88398	SNOMED CT	93800004	Primary malignant neoplasm of first cuneiform bone of foot	Cancer
88400	SNOMED CT	93802007	Primary malignant neoplasm of floor of mouth	Cancer
88404	SNOMED CT	93806005	Primary malignant neoplasm of frontal bone	Cancer
88405	SNOMED CT	93807001	Primary malignant neoplasm of frontal lobe	Cancer
88406	SNOMED CT	93808006	Primary malignant neoplasm of frontal sinus	Cancer
88407	SNOMED CT	93809003	Primary malignant neoplasm of fundus of stomach	Cancer
88410	SNOMED CT	93812000	Primary malignant neoplasm of gingival mucosa	Cancer
88414	SNOMED CT	93816002	Primary malignant neoplasm of glottis	Cancer
88416	SNOMED CT	93818001	Primary malignant neoplasm of greater curvature of stomach	Cancer
88418	SNOMED CT	93820003	Primary malignant neoplasm of hamate bone	Cancer
88423	SNOMED CT	93825008	Primary malignant neoplasm of heart	Cancer
88424	SNOMED CT	93826009	Primary malignant neoplasm of hepatic flexure of colon	Cancer
88425	SNOMED CT	93827000	Primary malignant neoplasm of hilus of lung	Cancer
88427	SNOMED CT	93829002	Primary malignant neoplasm of hypopharyngeal aspect of aryepiglottic fold	Cancer
88428	SNOMED CT	93830007	Primary malignant neoplasm of hypopharyngeal aspect of interarytenoid fold	Cancer
88429	SNOMED CT	93831006	Primary malignant neoplasm of hypopharynx	Cancer
88431	SNOMED CT	93833009	Primary malignant neoplasm of ilium	Cancer
88433	SNOMED CT	93835002	Primary malignant neoplasm of inner aspect of lip	Cancer
88434	SNOMED CT	93836001	Primary malignant neoplasm of inner aspect of lower lip	Cancer

88435	SNOMED CT	93837005	Primary malignant neoplasm of inner aspect of upper lip	Cancer
88439	SNOMED CT	93841009	Primary malignant neoplasm of intrathoracic organs	Cancer
88440	SNOMED CT	93842002	Primary malignant neoplasm of ischium	Cancer
88441	SNOMED CT	93843007	Primary malignant neoplasm of islets of Langerhans	Cancer
88442	SNOMED CT	93844001	Primary malignant neoplasm of isthmus of uterus	Cancer
88443	SNOMED CT	93845000	Primary malignant neoplasm of jaw	Cancer
88446	SNOMED CT	93848003	Primary malignant neoplasm of junctional zone of tongue	Cancer
88447	SNOMED CT	93849006	Primary malignant neoplasm of kidney	Cancer
88448	SNOMED CT	93850006	Primary malignant neoplasm of labia majora	Cancer
88449	SNOMED CT	93851005	Primary malignant neoplasm of labia minora	Cancer
88455	SNOMED CT	93857009	Primary malignant neoplasm of laryngeal commissure	Cancer
88456	SNOMED CT	93858004	Primary malignant neoplasm of laryngeal surface of epiglottis	Cancer
88458	SNOMED CT	93860002	Primary malignant neoplasm of lateral portion of floor of mouth	Cancer
88459	SNOMED CT	93861003	Primary malignant neoplasm of lateral wall of nasopharynx	Cancer
88460	SNOMED CT	93862005	Primary malignant neoplasm of lateral wall of oropharynx	Cancer
88462	SNOMED CT	93864006	Primary malignant neoplasm of lower lobe of left lung	Cancer
88463	SNOMED CT	93865007	Primary malignant neoplasm of left upper lobe of lung	Cancer
88465	SNOMED CT	93867004	Primary malignant neoplasm of lesser curvature of stomach	Cancer
88466	SNOMED CT	93868009	Primary malignant neoplasm of lingual tonsil	Cancer
88469	SNOMED CT	93871001	Primary malignant neoplasm of long bone of lower limb	Cancer
88470	SNOMED CT	93872008	Primary malignant neoplasm of long bone of upper limb	Cancer
88472	SNOMED CT	93874009	Primary malignant neoplasm of lower inner quadrant of female breast	Cancer
88474	SNOMED CT	93876006	Primary malignant neoplasm of lower outer quadrant of female breast	Cancer
88477	SNOMED CT	93879004	Primary malignant neoplasm of lunate bone	Cancer

88478	SNOMED CT	93880001	Primary malignant neoplasm of lung	Cancer
88480	SNOMED CT	93882009	Primary malignant neoplasm of main bronchus	Cancer
88481	SNOMED CT	93883004	Primary malignant neoplasm of major salivary gland	Cancer
88483	SNOMED CT	93885006	Primary malignant neoplasm of male genital organ	Cancer
88484	SNOMED CT	93886007	Primary malignant neoplasm of mandible	Cancer
88485	SNOMED CT	93887003	Primary malignant neoplasm of mastoid air cells	Cancer
88486	SNOMED CT	93888008	Primary malignant neoplasm of maxilla	Cancer
88487	SNOMED CT	93889000	Primary malignant neoplasm of maxillary sinus	Cancer
88488	SNOMED CT	93891008	Primary malignant neoplasm of mediastinum	Cancer
88489	SNOMED CT	93892001	Primary malignant neoplasm of metacarpal bone	Cancer
88490	SNOMED CT	93893006	Primary malignant neoplasm of metatarsal bone	Cancer
88491	SNOMED CT	93894000	Primary malignant neoplasm of middle ear	Cancer
88495	SNOMED CT	93898002	Primary malignant neoplasm of multiple endocrine glands	Cancer
88507	SNOMED CT	93910009	Primary malignant neoplasm of muscle of thorax	Cancer
88512	SNOMED CT	93915004	Primary malignant neoplasm of myometrium	Cancer
88513	SNOMED CT	93916003	Primary malignant neoplasm of nasal bone	Cancer
88514	SNOMED CT	93917007	Primary malignant neoplasm of nasal cavity	Cancer
88515	SNOMED CT	93918002	Primary malignant neoplasm of nasal concha	Cancer
88517	SNOMED CT	93920004	Primary malignant neoplasm of navicular bone of foot	Cancer
88521	SNOMED CT	93924008	Primary malignant neoplasm of nipple of female breast	Cancer
88524	SNOMED CT	93927001	Primary malignant neoplasm of occipital bone	Cancer
88525	SNOMED CT	93928006	Primary malignant neoplasm of occipital lobe	Cancer
88527	SNOMED CT	93930008	Primary malignant neoplasm of olfactory nerve	Cancer
88528	SNOMED CT	93931007	Primary malignant neoplasm of optic nerve	Cancer
88529	SNOMED CT	93932000	Primary malignant neoplasm of orbit	Cancer

88530	SNOMED CT	93933005	Primary malignant neoplasm of oropharynx	Cancer
88531	SNOMED CT	93934004	Primary malignant neoplasm of ovary	Cancer
88533	SNOMED CT	93936002	Primary malignant neoplasm of palatine bone	Cancer
88539	SNOMED CT	93942003	Primary malignant neoplasm of parametrium	Cancer
88540	SNOMED CT	93943008	Primary malignant neoplasm of parathyroid gland	Cancer
88541	SNOMED CT	93944002	Primary malignant neoplasm of paraurethral glands	Cancer
88542	SNOMED CT	93945001	Primary malignant neoplasm of parietal bone	Cancer
88543	SNOMED CT	93946000	Primary malignant neoplasm of parietal lobe	Cancer
88544	SNOMED CT	93947009	Primary malignant neoplasm of parietal peritoneum	Cancer
88545	SNOMED CT	93948004	Primary malignant neoplasm of parietal pleura	Cancer
88547	SNOMED CT	93950007	Primary malignant neoplasm of patella	Cancer
88548	SNOMED CT	93951006	Primary malignant neoplasm of pelvic bone	Cancer
88549	SNOMED CT	93952004	Primary malignant neoplasm of pelvic peritoneum	Cancer
88552	SNOMED CT	93955002	Primary malignant neoplasm of periadrenal tissue	Cancer
88554	SNOMED CT	93957005	Primary malignant neoplasm of pericardium	Cancer
88555	SNOMED CT	93958000	Primary malignant neoplasm of perirenal tissue	Cancer
88556	SNOMED CT	93959008	Primary malignant neoplasm of phalanx of foot	Cancer
88557	SNOMED CT	93960003	Primary malignant neoplasm of phalanx of hand	Cancer
88558	SNOMED CT	93961004	Primary malignant neoplasm of pharynx	Cancer
88559	SNOMED CT	93962006	Primary malignant neoplasm of pineal gland	Cancer
88560	SNOMED CT	93963001	Primary malignant neoplasm of pisiform bone of hand	Cancer
88561	SNOMED CT	93964007	Primary malignant neoplasm of pituitary gland	Cancer
88563	SNOMED CT	93966009	Primary malignant neoplasm of pleura	Cancer
88564	SNOMED CT	93967000	Primary malignant neoplasm of postcricoid region	Cancer
88565	SNOMED CT	93968005	Primary malignant neoplasm of posterior hypopharyngeal wall	Cancer

88566	SNOMED CT	93969002	Primary malignant neoplasm of posterior mediastinum	Cancer
88567	SNOMED CT	93970001	Primary malignant neoplasm of posterior wall of nasopharynx	Cancer
88568	SNOMED CT	93971002	Primary malignant neoplasm of posterior wall of oropharynx	Cancer
88571	SNOMED CT	93974005	Primary malignant neoplasm of prostate	Cancer
88572	SNOMED CT	93975006	Primary malignant neoplasm of pubis	Cancer
88573	SNOMED CT	93976007	Primary malignant neoplasm of pyloric antrum	Cancer
88574	SNOMED CT	93977003	Primary malignant neoplasm of pylorus	Cancer
88575	SNOMED CT	93978008	Primary malignant neoplasm of pyriform sinus	Cancer
88576	SNOMED CT	93979000	Primary malignant neoplasm of radius	Cancer
88577	SNOMED CT	93980002	Primary malignant neoplasm of rectosigmoid junction	Cancer
88578	SNOMED CT	93981003	Primary malignant neoplasm of rectouterine pouch	Cancer
88579	SNOMED CT	93982005	Primary malignant neoplasm of rectovaginal septum	Cancer
88580	SNOMED CT	93983000	Primary malignant neoplasm of rectovesical septum	Cancer
88582	SNOMED CT	93985007	Primary malignant neoplasm of renal pelvis	Cancer
88583	SNOMED CT	93986008	Primary malignant neoplasm of respiratory tract	Cancer
88584	SNOMED CT	93987004	Primary malignant neoplasm of retina	Cancer
88586	SNOMED CT	93989001	Primary malignant neoplasm of retromolar area	Cancer
88587	SNOMED CT	93990005	Primary malignant neoplasm of rib	Cancer
88588	SNOMED CT	93991009	Primary malignant neoplasm of right lower lobe of lung	Cancer
88589	SNOMED CT	93992002	Primary malignant neoplasm of right middle lobe of lung	Cancer
88590	SNOMED CT	93993007	Primary malignant neoplasm of upper lobe of right lung	Cancer
88591	SNOMED CT	93994001	Primary malignant neoplasm of round ligament of uterus	Cancer
88593	SNOMED CT	93996004	Primary malignant neoplasm of sacrum	Cancer
88594	SNOMED CT	93997008	Primary malignant neoplasm of scapula	Cancer
88595	SNOMED CT	93998003	Primary malignant neoplasm of sclera of eye	Cancer

88598	SNOMED CT	94001007	Primary malignant neoplasm of second cuneiform bone of foot	Cancer
88599	SNOMED CT	94002000	Primary malignant neoplasm of septum of nose	Cancer
88600	SNOMED CT	94003005	Primary malignant neoplasm of short bone of lower limb	Cancer
88603	SNOMED CT	94006002	Primary malignant neoplasm of sigmoid colon	Cancer
88607	SNOMED CT	94010004	Primary malignant neoplasm of skin of axilla	Cancer
88611	SNOMED CT	94014008	Primary malignant neoplasm of skin of cheek	Cancer
88612	SNOMED CT	94015009	Primary malignant neoplasm of skin of chest	Cancer
88617	SNOMED CT	94020009	Primary malignant neoplasm of skin of eyebrow	Cancer
88618	SNOMED CT	94021008	Primary malignant neoplasm of skin of eyelid	Cancer
88646	SNOMED CT	94049001	Primary malignant neoplasm of soft palate	Cancer
88648	SNOMED CT	94051002	Primary malignant neoplasm of soft tissues of axilla	Cancer
88659	SNOMED CT	94062002	Primary malignant neoplasm of soft tissues of thorax	Cancer
88663	SNOMED CT	94066004	Primary malignant neoplasm of sphenoid bone	Cancer
88664	SNOMED CT	94067008	Primary malignant neoplasm of sphenoidal sinus	Cancer
88665	SNOMED CT	94068003	Primary malignant neoplasm of spinal cord	Cancer
88666	SNOMED CT	94069006	Primary malignant neoplasm of spinal meninges	Cancer
88668	SNOMED CT	94071006	Primary malignant neoplasm of spleen	Cancer
88669	SNOMED CT	94072004	Primary malignant neoplasm of splenic flexure of colon	Cancer
88670	SNOMED CT	94073009	Primary malignant neoplasm of sternum	Cancer
88672	SNOMED CT	94075002	Primary malignant neoplasm of subglottis	Cancer
88673	SNOMED CT	94076001	Primary malignant neoplasm of sublingual gland	Cancer
88674	SNOMED CT	94077005	Primary malignant neoplasm of submaxillary gland	Cancer
88675	SNOMED CT	94078000	Primary malignant neoplasm of superior wall of nasopharynx	Cancer
88677	SNOMED CT	94080006	Primary malignant neoplasm of supraglottis	Cancer
88680	SNOMED CT	94083008	Primary malignant neoplasm of talus	Cancer

88681	SNOMED CT	94084002	Primary malignant neoplasm of tarsal bone	Cancer
88682	SNOMED CT	94085001	Primary malignant neoplasm of temporal bone	Cancer
88683	SNOMED CT	94086000	Primary malignant neoplasm of temporal lobe	Cancer
88684	SNOMED CT	94087009	Primary malignant neoplasm of testis	Cancer
88686	SNOMED CT	94089007	Primary malignant neoplasm of the mesocolon	Cancer
88687	SNOMED CT	94090003	Primary malignant neoplasm of omentum	Cancer
88691	SNOMED CT	94094007	Primary malignant neoplasm of third cuneiform bone of foot	Cancer
88693	SNOMED CT	94096009	Primary malignant neoplasm of thymus	Cancer
88695	SNOMED CT	94098005	Primary malignant neoplasm of thyroid gland	Cancer
88696	SNOMED CT	94099002	Primary malignant neoplasm of tibia	Cancer
88698	SNOMED CT	94101009	Primary malignant neoplasm of tongue	Cancer
88699	SNOMED CT	94102002	Primary malignant neoplasm of tonsillar fossa	Cancer
88700	SNOMED CT	94103007	Primary malignant neoplasm of tonsillar pillar	Cancer
88701	SNOMED CT	94104001	Primary malignant neoplasm of trachea	Cancer
88702	SNOMED CT	94105000	Primary malignant neoplasm of transverse colon	Cancer
88704	SNOMED CT	94107008	Primary malignant neoplasm of trapezoid bone	Cancer
88706	SNOMED CT	94109006	Primary malignant neoplasm of trigone of urinary bladder	Cancer
88709	SNOMED CT	94112009	Primary malignant neoplasm of ulna	Cancer
88710	SNOMED CT	94113004	Primary malignant neoplasm of undescended testis	Cancer
88712	SNOMED CT	94115006	Primary malignant neoplasm of upper inner quadrant of female breast	Cancer
88714	SNOMED CT	94117003	Primary malignant neoplasm of upper outer quadrant of female breast	Cancer
88715	SNOMED CT	94118008	Primary malignant neoplasm of upper respiratory tract	Cancer
88717	SNOMED CT	94120006	Primary malignant neoplasm of urachus	Cancer
88718	SNOMED CT	94121005	Primary malignant neoplasm of ureter	Cancer

88719	SNOMED CT	94122003	Primary malignant neoplasm of ureteric orifice of urinary bladder	Cancer
88720	SNOMED CT	94123008	Primary malignant neoplasm of urethra	Cancer
88721	SNOMED CT	94124002	Primary malignant neoplasm of urinary bladder neck	Cancer
88722	SNOMED CT	94125001	Primary malignant neoplasm of urinary system	Cancer
88725	SNOMED CT	94128004	Primary malignant neoplasm of uveal tract of eye	Cancer
88726	SNOMED CT	94129007	Primary malignant neoplasm of uvula	Cancer
88729	SNOMED CT	94132005	Primary malignant neoplasm of vallecula	Cancer
88730	SNOMED CT	94133000	Primary malignant neoplasm of vas deferens	Cancer
88731	SNOMED CT	94134006	Primary malignant neoplasm of ventral surface of tongue	Cancer
88732	SNOMED CT	94135007	Primary malignant neoplasm of vermilion border of lip	Cancer
88735	SNOMED CT	94138009	Primary malignant neoplasm of vestibule of mouth	Cancer
88736	SNOMED CT	94139001	Primary malignant neoplasm of vestibule of nose	Cancer
88737	SNOMED CT	94140004	Primary malignant neoplasm of visceral pleura	Cancer
88739	SNOMED CT	94142007	Primary malignant neoplasm of vomer	Cancer
88740	SNOMED CT	94143002	Primary malignant neoplasm of vulva	Cancer
88741	SNOMED CT	94145009	Primary malignant neoplasm of zygomatic bone	Cancer
88744	SNOMED CT	94148006	Megakaryocytic leukemia in remission	Cancer
88775	SNOMED CT	94179005	Secondary malignant neoplasm of ascending colon	Cancer
88856	SNOMED CT	94260004	Secondary malignant neoplasm of colon	Cancer
88867	SNOMED CT	94271003	Secondary malignant neoplasm of descending colon	Cancer
88924	SNOMED CT	94328005	Secondary malignant neoplasm of hepatic flexure of colon	Cancer
89103	SNOMED CT	94509004	Secondary malignant neoplasm of rectosigmoid junction	Cancer
89132	SNOMED CT	94538001	Secondary malignant neoplasm of sigmoid colon	Cancer
89198	SNOMED CT	94604000	Secondary malignant neoplasm of splenic flexure of colon	Cancer

89237	SNOMED CT	94643001	Secondary malignant neoplasm of transverse colon	Cancer
89279	SNOMED CT	94686001	Mixed cell type lymphosarcoma of intra-abdominal lymph nodes	Cancer
89280	SNOMED CT	94687005	Mixed cell type lymphosarcoma of intrapelvic lymph nodes	Cancer
89281	SNOMED CT	94688000	Mixed cell type lymphosarcoma of intrathoracic lymph nodes	Cancer
89283	SNOMED CT	94690004	Mixed cell type lymphosarcoma of lymph nodes of head, face, and neck	Cancer
89300	SNOMED CT	94707004	Mycosis fungoides of intra-abdominal lymph nodes	Cancer
89301	SNOMED CT	94708009	Mycosis fungoides of intrapelvic lymph nodes	Cancer
89302	SNOMED CT	94709001	Mycosis fungoides of intrathoracic lymph nodes	Cancer
89303	SNOMED CT	94710006	Mycosis fungoides of lymph nodes of axilla AND/OR upper limb	Cancer
89304	SNOMED CT	94711005	Mycosis fungoides of lymph nodes of head, face AND/OR neck	Cancer
89307	SNOMED CT	94714002	Mycosis fungoides of spleen	Cancer
89308	SNOMED CT	94715001	Mycosis fungoides of extranodal AND/OR solid organ site	Cancer
89309	SNOMED CT	94716000	Myeloid leukemia in remission	Cancer
89311	SNOMED CT	94718004	Myeloid sarcoma in remission	Cancer
89318	SNOMED CT	94726007	Neoplasm of uncertain behavior of acromion	Cancer
89337	SNOMED CT	94745003	Neoplasm of uncertain behavior of areola of female breast	Cancer
89342	SNOMED CT	94750009	Neoplasm of uncertain behavior of axillary tail of female breast	Cancer
89776	SNOMED CT	95186006	Nodular lymphoma of intra-abdominal lymph nodes	Cancer
89777	SNOMED CT	95187002	Nodular lymphoma of intrapelvic lymph nodes	Cancer
89778	SNOMED CT	95188007	Nodular lymphoma of intrathoracic lymph nodes	Cancer
89782	SNOMED CT	95192000	Nodular lymphoma of lymph nodes of multiple sites	Cancer
89783	SNOMED CT	95193005	Nodular lymphoma of spleen	Cancer
89784	SNOMED CT	95194004	Nodular lymphoma of extranodal AND/OR solid organ site	Cancer

89799	SNOMED CT	95209008	Plasma cell leukemia in remission	Cancer
89800	SNOMED CT	95210003	Plasma cell leukemia	Cancer
89814	SNOMED CT	95224004	Reticulosarcoma of intra-abdominal lymph nodes	Cancer
89815	SNOMED CT	95225003	Reticulosarcoma of intrapelvic lymph nodes	Cancer
89816	SNOMED CT	95226002	Reticulosarcoma of intrathoracic lymph nodes	Cancer
89820	SNOMED CT	95230004	Reticulosarcoma of lymph nodes of multiple sites	Cancer
89821	SNOMED CT	95231000	Reticulosarcoma of spleen	Cancer
89850	SNOMED CT	95260009	S√©zary's disease of lymph nodes of head, face AND/OR neck	Cancer
89853	SNOMED CT	95263006	S√©zary's disease of spleen	Cancer
89854	SNOMED CT	95264000	S√©zary's disease of extranodal AND/OR solid organ site	Cancer
90002	SNOMED CT	95413004	Nodular tenosynovitis	Cancer
99031	SNOMED CT	109267002	Overlapping malignant melanoma of skin	Cancer
99112	SNOMED CT	109348004	Overlapping malignant neoplasm of bone and articular cartilage of limb	Cancer
99114	SNOMED CT	109351006	Lipoma of skin and subcutaneous tissue of limb	Cancer
99126	SNOMED CT	109366009	Overlapping malignant neoplasm of accessory sinuses	Cancer
99127	SNOMED CT	109367000	Overlapping malignant neoplasm of nasopharynx	Cancer
99128	SNOMED CT	109368005	Malignant neoplasm of overlapping sites of hypopharynx	Cancer
99129	SNOMED CT	109369002	Malignant neoplasm of overlapping sites of larynx	Cancer
99130	SNOMED CT	109370001	Primary malignant neoplasm of laryngeal cartilage	Cancer
99131	SNOMED CT	109371002	Malignant neoplasm of overlapping sites of bronchus and lung	Cancer
99134	SNOMED CT	109374005	Overlapping malignant neoplasm of mediastinum and pleura	Cancer
99143	SNOMED CT	109383000	Malignant mesothelioma of pericardium	Cancer
99144	SNOMED CT	109384006	Malignant neoplasm of overlapping sites of heart mediastinum and pleura	Cancer

99564	SNOMED CT	109822001	Overlapping malignant neoplasm of lip	Cancer
99565	SNOMED CT	109823006	Malignant neoplasm of overlapping sites of tongue	Cancer
99566	SNOMED CT	109824000	Malignant neoplasm of overlapping sites of major salivary gland	Cancer
99570	SNOMED CT	109828002	Primary malignant neoplasm of salivary gland duct	Cancer
99572	SNOMED CT	109830000	Malignant neoplasm of overlapping sites of floor of mouth	Cancer
99573	SNOMED CT	109831001	Malignant neoplasm of overlapping sites of palate	Cancer
99574	SNOMED CT	109832008	Overlapping malignant neoplasm of oropharynx	Cancer
99577	SNOMED CT	109835005	Malignant neoplasm of overlapping sites of esophagus	Cancer
99578	SNOMED CT	109836006	Malignant neoplasm of overlapping sites of stomach	Cancer
99580	SNOMED CT	109838007	Malignant neoplasm of overlapping sites of colon	Cancer
99581	SNOMED CT	109839004	Overlapping malignant neoplasm of rectum, anus and anal canal	Cancer
99582	SNOMED CT	109840002	Primary malignant neoplasm of cloacogenic zone	Cancer
99593	SNOMED CT	109851002	Overlapping malignant neoplasm of retroperitoneum and peritoneum	Cancer
99595	SNOMED CT	109853004	Mesothelioma of peritoneum	Cancer
99596	SNOMED CT	109854005	Mesothelioma of parietal peritoneum	Cancer
99597	SNOMED CT	109855006	Mesothelioma of pelvic peritoneum	Cancer
99598	SNOMED CT	109856007	Mesothelioma of mesentery	Cancer
99599	SNOMED CT	109857003	Mesothelioma of mesocolon	Cancer
99600	SNOMED CT	109858008	Mesothelioma of omentum	Cancer
99612	SNOMED CT	109870007	Overlapping malignant neoplasm of urinary system	Cancer
99614	SNOMED CT	109874003	Malignant neoplasm of overlapping sites of male genital organs	Cancer
99615	SNOMED CT	109875002	Malignant neoplasm of overlapping sites of penis	Cancer
99616	SNOMED CT	109876001	Primary malignant neoplasm of descended testis	Cancer

99617	SNOMED CT	109878000	Overlapping malignant neoplasm of female genital organs	Cancer
99618	SNOMED CT	109879008	Overlapping malignant neoplasm of body of uterus	Cancer
99619	SNOMED CT	109880006	Overlapping malignant neoplasm of uterine cervix	Cancer
99621	SNOMED CT	109882003	Primary malignant neoplasm of fundus uteri	Cancer
99623	SNOMED CT	109886000	Overlapping malignant neoplasm of female breast	Cancer
99648	SNOMED CT	109911004	Overlapping malignant neoplasm of brain and other parts of the central nervous system	Cancer
99649	SNOMED CT	109912006	Malignant neoplasm of overlapping sites of brain	Cancer
99652	SNOMED CT	109915008	Primary malignant neoplasm of meninges	Cancer
99673	SNOMED CT	109941002	Primary malignant neoplasm of peripheral nerves of thorax	Cancer
99680	SNOMED CT	109948008	Malignant neoplasm of overlapping sites of eye and adnexa	Cancer
99683	SNOMED CT	109951001	Overlapping malignant neoplasm of multiple endocrine glands	Cancer
99691	SNOMED CT	109975001	T-zone lymphoma (clinical)	Cancer
99692	SNOMED CT	109976000	Lymphoepithelioid lymphoma (clinical)	Cancer
99693	SNOMED CT	109978004	T-cell lymphoma (clinical)	Cancer
99694	SNOMED CT	109980005	Malignant immunoproliferative disease (clinical)	Cancer
99700	SNOMED CT	109988003	Histiocytic sarcoma (clinical)	Cancer
99705	SNOMED CT	109994006	Essential thrombocythemia	Cancer
99706	SNOMED CT	109995007	Myelodysplastic syndrome (clinical)	Cancer
99707	SNOMED CT	109996008	Refractory anemia (clinical)	Cancer
99708	SNOMED CT	109998009	Refractory anemia with ringed sideroblasts associated with marked thrombocytosis	Cancer
99710	SNOMED CT	110000005	Refractory anemia with excess blasts in transformation (clinical)	Cancer
99711	SNOMED CT	110002002	Mast cell leukemia (clinical)	Cancer
99712	SNOMED CT	110006004	Prolymphocytic leukemia (clinical)	Cancer
99714	SNOMED CT	110013004	Overlapping malignant neoplasm of tonsil	Cancer

107141	SNOMED CT	118607005	Lymphocyte-rich classical Hodgkin lymphoma	Cancer
107142	SNOMED CT	118611004	S√©zary's disease (clinical)	Cancer
107143	SNOMED CT	118612006	Malignant histiocytosis (clinical)	Cancer
107144	SNOMED CT	118615008	Malignant mast cell tumor (clinical)	Cancer
107146	SNOMED CT	118618005	Mycosis fungoides (clinical)	Cancer
111003	SNOMED CT	123842006	Endocervical adenocarcinoma	Cancer
113399	SNOMED CT	126948004	Cerebellopontine angle meningioma	Cancer
113515	SNOMED CT	127070008	Malignant histiocytic disorder	Cancer
113665	SNOMED CT	127220001	Malignant lymphoma of lymph nodes	Cancer
113670	SNOMED CT	127225006	Chronic myelomonocytic leukemia	Cancer
170394	SNOMED CT	187601000	Malignant neoplasm of upper lip, lipstick area	Cancer
170397	SNOMED CT	187604008	Malignant neoplasm of lower lip, external	Cancer
170399	SNOMED CT	187606005	Malignant tumor of upper labial mucosa	Cancer
170401	SNOMED CT	187608006	Malignant tumor of frenum of upper lip	Cancer
170406	SNOMED CT	187613005	Malignant neoplasm of lower lip, buccal aspect	Cancer
170407	SNOMED CT	187614004	Malignant tumor of frenum of lower lip	Cancer
170415	SNOMED CT	187622006	Malignant tumor of labial mucosa	Cancer
170424	SNOMED CT	187631006	Malignant neoplasm of base of tongue dorsal surface	Cancer
170427	SNOMED CT	187634003	Malignant tumor of anterior two-thirds of tongue - dorsal surface	Cancer
170428	SNOMED CT	187635002	Malignant neoplasm of midline of tongue	Cancer
170430	SNOMED CT	187637005	Malignant neoplasm of tongue, tip and lateral border	Cancer
170433	SNOMED CT	187640005	Malignant tumor of anterior two-thirds of tongue - ventral surface	Cancer
170434	SNOMED CT	187641009	Malignant tumor of frenum linguae	Cancer
170437	SNOMED CT	187644001	Malignant tumor of junctional zone of tongue	Cancer

170445	SNOMED CT	187652003	Malignant tumor of anterior floor of mouth	Cancer
170446	SNOMED CT	187653008	Malignant tumor of lateral floor of mouth	Cancer
170451	SNOMED CT	187658004	Malignant tumor of vestibule of mouth	Cancer
170452	SNOMED CT	187659007	Malignant tumor of upper buccal sulcus	Cancer
170453	SNOMED CT	187660002	Malignant tumor of lower buccal sulcus	Cancer
170454	SNOMED CT	187661003	Malignant tumor of upper labial sulcus	Cancer
170455	SNOMED CT	187662005	Malignant tumor of lower labial sulcus	Cancer
170459	SNOMED CT	187666008	Malignant neoplasm of junction of hard and soft palate	Cancer
170468	SNOMED CT	187675005	Malignant tumor of tonsillar pillar	Cancer
170474	SNOMED CT	187681002	Malignant neoplasm of anterior epiglottis	Cancer
170475	SNOMED CT	187682009	Malignant neoplasm of epiglottis, free border	Cancer
170476	SNOMED CT	187683004	Malignant neoplasm of glossoepiglottic fold	Cancer
170478	SNOMED CT	187685006	Malignant neoplasm of junctional region of epiglottis	Cancer
170481	SNOMED CT	187688008	Malignant tumor of posterior wall of oropharynx	Cancer
170485	SNOMED CT	187692001	Malignant tumor of nasopharynx	Cancer
170486	SNOMED CT	187693006	Malignant tumor of posterior wall of nasopharynx	Cancer
170487	SNOMED CT	187694000	Malignant tumor of adenoid	Cancer
170490	SNOMED CT	187697007	Malignant tumor of pharyngeal recess	Cancer
170491	SNOMED CT	187698002	Malignant tumor of opening of auditory tube	Cancer
170493	SNOMED CT	187700006	Malignant tumor of anterior wall of nasopharynx	Cancer
170494	SNOMED CT	187701005	Malignant neoplasm of floor of nasopharynx	Cancer
170501	SNOMED CT	187708004	Malignant tumor aryepiglottic fold - hypopharyngeal aspect	Cancer
170502	SNOMED CT	187709007	Malignant neoplasm of posterior pharynx	Cancer
170514	SNOMED CT	187722004	Malignant tumor of cervical part of esophagus	Cancer
170515	SNOMED CT	187723009	Malignant tumor of thoracic part of esophagus	Cancer

170516	SNOMED CT	187724003	Malignant tumor of abdominal part of esophagus	Cancer
170517	SNOMED CT	187725002	Malignant tumor of upper third of esophagus	Cancer
170518	SNOMED CT	187726001	Malignant tumor of middle third of esophagus	Cancer
170519	SNOMED CT	187727005	Malignant tumor of lower third of esophagus	Cancer
170524	SNOMED CT	187732006	Malignant tumor of cardia	Cancer
170525	SNOMED CT	187733001	Malignant neoplasm of cardiac orifice of stomach	Cancer
170527	SNOMED CT	187736009	Malignant tumor of pylorus	Cancer
170529	SNOMED CT	187738005	Malignant neoplasm of pyloric canal of stomach	Cancer
170531	SNOMED CT	187740000	Malignant tumor of pyloric antrum	Cancer
170532	SNOMED CT	187741001	Malignant tumor of fundus of stomach	Cancer
170533	SNOMED CT	187742008	Malignant tumor of body of stomach	Cancer
170547	SNOMED CT	187757001	Malignant neoplasm, overlapping lesion of colon	Cancer
170576	SNOMED CT	187786003	Malignant neoplasm of sphincter of Oddi	Cancer
170584	SNOMED CT	187794005	Malignant tumor of Islets of Langerhans	Cancer
170591	SNOMED CT	187801002	Malignant tumor of peritoneum and retroperitoneum	Cancer
170593	SNOMED CT	187803004	Malignant neoplasm of perinephric tissue	Cancer
170599	SNOMED CT	187809000	Malignant neoplasm of mesocolon	Cancer
170600	SNOMED CT	187810005	Malignant neoplasm of mesocecum	Cancer
170601	SNOMED CT	187811009	Malignant neoplasm of mesorectum	Cancer
170604	SNOMED CT	187814001	Malignant neoplasm of the pouch of Douglas	Cancer
170612	SNOMED CT	187822008	Fibrosarcoma of spleen	Cancer
170619	SNOMED CT	187829004	Malignant neoplasm of cartilage of nose	Cancer
170620	SNOMED CT	187830009	Malignant neoplasm of nasal conchae	Cancer
170621	SNOMED CT	187831008	Malignant tumor of nasal vestibule	Cancer
170623	SNOMED CT	187833006	Malignant neoplasm of auditory tube, middle ear and mastoid air cells	Cancer

170624	SNOMED CT	187834000	Malignant tumor of Eustachian tube	Cancer
170625	SNOMED CT	187835004	Malignant tumor of tympanic cavity	Cancer
170626	SNOMED CT	187836003	Malignant tumor of tympanic antrum	Cancer
170631	SNOMED CT	187841006	Malignant tumor of glottis	Cancer
170632	SNOMED CT	187842004	Malignant tumor of supraglottis	Cancer
170633	SNOMED CT	187843009	Malignant neoplasm of arytenoid cartilage	Cancer
170634	SNOMED CT	187844003	Malignant neoplasm of cricoid cartilage	Cancer
170635	SNOMED CT	187845002	Malignant neoplasm of cuneiform cartilage	Cancer
170636	SNOMED CT	187846001	Malignant neoplasm of thyroid cartilage	Cancer
170643	SNOMED CT	187853005	Malignant neoplasm of cartilage of trachea	Cancer
170644	SNOMED CT	187854004	Malignant neoplasm of mucosa of trachea	Cancer
170647	SNOMED CT	187857006	Malignant neoplasm of carina of bronchus	Cancer
170651	SNOMED CT	187861000	Malignant neoplasm of upper lobe bronchus	Cancer
170652	SNOMED CT	187862007	Malignant neoplasm of upper lobe of lung	Cancer
170655	SNOMED CT	187865009	Malignant neoplasm of middle lobe bronchus	Cancer
170656	SNOMED CT	187866005	Malignant neoplasm of middle lobe of lung	Cancer
170659	SNOMED CT	187869003	Malignant neoplasm of lower lobe bronchus	Cancer
170660	SNOMED CT	187870002	Malignant neoplasm of lower lobe of lung	Cancer
170690	SNOMED CT	187900002	Malignant neoplasm of bones of skull and face	Cancer
170693	SNOMED CT	187903000	Malignant neoplasm of malar bone	Cancer
170696	SNOMED CT	187906008	Malignant neoplasm of orbital bone	Cancer
170706	SNOMED CT	187916000	Malignant neoplasm of cervical vertebra	Cancer
170707	SNOMED CT	187917009	Malignant neoplasm of thoracic vertebra	Cancer
170708	SNOMED CT	187918004	Malignant neoplasm of lumbar vertebra	Cancer
170710	SNOMED CT	187920001	Malignant neoplasm of ribs and/or sternum and/or clavicle	Cancer

170715	SNOMED CT	187925006	Malignant neoplasm of costal cartilage	Cancer
170716	SNOMED CT	187926007	Malignant neoplasm of costovertebral joint	Cancer
170717	SNOMED CT	187927003	Malignant neoplasm of xiphoid process	Cancer
170719	SNOMED CT	187929000	Malignant neoplasm of scapula and long bones of upper arm	Cancer
170722	SNOMED CT	187932002	Malignant neoplasm of humerus	Cancer
170727	SNOMED CT	187937008	Malignant neoplasm of carpal bone - scaphoid	Cancer
170728	SNOMED CT	187938003	Malignant neoplasm of carpal bone - lunate	Cancer
170729	SNOMED CT	187939006	Malignant neoplasm of carpal bone - triquetrum	Cancer
170730	SNOMED CT	187940008	Malignant neoplasm of carpal bone - pisiform	Cancer
170731	SNOMED CT	187941007	Malignant neoplasm of carpal bone - trapezium	Cancer
170732	SNOMED CT	187942000	Malignant neoplasm of carpal bone - trapezoid	Cancer
170733	SNOMED CT	187943005	Malignant neoplasm of carpal bone - capitate	Cancer
170734	SNOMED CT	187944004	Malignant neoplasm of carpal bone - hamate	Cancer
170735	SNOMED CT	187945003	Malignant neoplasm of first metacarpal bone	Cancer
170736	SNOMED CT	187946002	Malignant neoplasm of second metacarpal bone	Cancer
170737	SNOMED CT	187947006	Malignant neoplasm of third metacarpal bone	Cancer
170738	SNOMED CT	187948001	Malignant neoplasm of fourth metacarpal bone	Cancer
170739	SNOMED CT	187949009	Malignant neoplasm of fifth metacarpal bone	Cancer
170740	SNOMED CT	187950009	Malignant neoplasm of phalanges of hand	Cancer
170742	SNOMED CT	187952001	Malignant neoplasm of pelvic bones, sacrum and coccyx	Cancer
170746	SNOMED CT	187956003	Malignant neoplasm of sacral vertebra	Cancer
170747	SNOMED CT	187957007	Malignant neoplasm of coccygeal vertebra	Cancer
170757	SNOMED CT	187967002	Malignant neoplasm of calcaneum	Cancer
170758	SNOMED CT	187968007	Malignant neoplasm of medial cuneiform	Cancer
170759	SNOMED CT	187969004	Malignant neoplasm of intermediate cuneiform	Cancer

170760	SNOMED CT	187970003	Malignant neoplasm of lateral cuneiform	Cancer
170762	SNOMED CT	187972006	Malignant neoplasm of navicular	Cancer
170763	SNOMED CT	187973001	Malignant neoplasm of first metatarsal bone	Cancer
170764	SNOMED CT	187974007	Malignant neoplasm of second metatarsal bone	Cancer
170765	SNOMED CT	187975008	Malignant neoplasm of third metatarsal bone	Cancer
170766	SNOMED CT	187976009	Malignant neoplasm of fourth metatarsal bone	Cancer
170767	SNOMED CT	187977000	Malignant neoplasm of fifth metatarsal bone	Cancer
170768	SNOMED CT	187978005	Malignant neoplasm of phalanges of foot	Cancer
170778	SNOMED CT	187988006	Malignant neoplasm of tarsus of eyelid	Cancer
170800	SNOMED CT	188010006	Malignant neoplasm of connective and soft tissue of axilla	Cancer
170803	SNOMED CT	188013008	Malignant neoplasm of connective and soft tissues of thoracic spine	Cancer
170838	SNOMED CT	188049009	Malignant melanoma of axilla	Cancer
170819	SNOMED CT	188030005	Malignant melanoma of lip	Cancer
170822	SNOMED CT	188033007	Malignant melanoma of auricle (ear)	Cancer
170860	SNOMED CT	188071004	Malignant melanoma of popliteal fossa area	Cancer
170861	SNOMED CT	188072006	Malignant melanoma of lower leg	Cancer
170884	SNOMED CT	188095002	Malignant neoplasm of skin of cheek, external	Cancer
170896	SNOMED CT	188107002	Malignant neoplasm of skin of axillary fold	Cancer
170936	SNOMED CT	188147009	Malignant neoplasm of nipple and areola of female breast	Cancer
170940	SNOMED CT	188151006	Malignant neoplasm of central part of female breast	Cancer
170941	SNOMED CT	188152004	Malignant neoplasm of upper-inner quadrant of female breast	Cancer
170942	SNOMED CT	188153009	Malignant neoplasm of lower-inner quadrant of female breast	Cancer
170943	SNOMED CT	188154003	Malignant neoplasm of upper-outer quadrant of female breast	Cancer
170944	SNOMED CT	188155002	Malignant neoplasm of lower-outer quadrant of female breast	Cancer

170945	SNOMED CT	188156001	Malignant neoplasm of axillary tail of female breast	Cancer
170948	SNOMED CT	188159008	Malignant neoplasm of ectopic site of female breast	Cancer
170965	SNOMED CT	188176007	Malignant neoplasm of endocervical canal	Cancer
170966	SNOMED CT	188177003	Malignant neoplasm of endocervical gland	Cancer
170969	SNOMED CT	188180002	Primary malignant neoplasm of overlapping sites of cervix uteri	Cancer
170972	SNOMED CT	188183000	Malignant neoplasm of cervical stump	Cancer
170973	SNOMED CT	188184006	Malignant neoplasm of squamocolumnar junction of cervix	Cancer
170978	SNOMED CT	188189001	Malignant neoplasm of corpus uteri, excluding isthmus	Cancer
170979	SNOMED CT	188190005	Malignant neoplasm of cornu of corpus uteri	Cancer
170980	SNOMED CT	188191009	Malignant neoplasm of fundus of corpus uteri	Cancer
170981	SNOMED CT	188192002	Malignant neoplasm of endometrium of corpus uteri	Cancer
170982	SNOMED CT	188193007	Malignant neoplasm of myometrium of corpus uteri	Cancer
170984	SNOMED CT	188195000	Malignant neoplasm of isthmus of uterine body	Cancer
170993	SNOMED CT	188204000	Malignant neoplasm of round ligament	Cancer
170997	SNOMED CT	188209005	Malignant neoplasm of vaginal vault	Cancer
171007	SNOMED CT	188219004	Malignant tumor of undescended testis	Cancer
171008	SNOMED CT	188220005	Malignant tumor of ectopic testis	Cancer
171018	SNOMED CT	188230001	Malignant tumor of body of penis	Cancer
171022	SNOMED CT	188234005	Malignant tumor of seminal vesicle	Cancer
171023	SNOMED CT	188235006	Malignant tumor of tunica vaginalis	Cancer
171027	SNOMED CT	188239000	Malignant tumor of trigone of urinary bladder	Cancer
171028	SNOMED CT	188240003	Malignant tumor of vault of bladder	Cancer
171029	SNOMED CT	188241004	Malignant neoplasm of lateral wall of urinary bladder	Cancer
171030	SNOMED CT	188242006	Malignant neoplasm of anterior wall of urinary bladder	Cancer
171031	SNOMED CT	188243001	Malignant neoplasm of posterior wall of urinary bladder	Cancer

171032	SNOMED CT	188244007	Malignant tumor of bladder neck	Cancer
171033	SNOMED CT	188245008	Malignant tumor of ureteric orifice	Cancer
171035	SNOMED CT	188247000	Malignant neoplasm of overlapping sites of bladder	Cancer
171038	SNOMED CT	188250002	Malignant tumor of kidney parenchyma	Cancer
171040	SNOMED CT	188252005	Malignant tumor of renal calyx	Cancer
171041	SNOMED CT	188253000	Malignant tumor of pelviureteric junction	Cancer
171044	SNOMED CT	188256008	Malignant neoplasm of overlapping sites of urinary organs	Cancer
171049	SNOMED CT	188261005	Malignant neoplasm of eyeball excluding conjunctiva, cornea, retina and choroid	Cancer
171051	SNOMED CT	188263008	Malignant tumor of ciliary body	Cancer
171052	SNOMED CT	188264002	Malignant tumor of iris	Cancer
171053	SNOMED CT	188265001	Malignant neoplasm of crystalline lens	Cancer
171054	SNOMED CT	188266000	Malignant neoplasm of sclera	Cancer
171056	SNOMED CT	188268004	Malignant neoplasm of connective tissue of orbit	Cancer
171057	SNOMED CT	188269007	Malignant neoplasm of extraocular muscle of orbit	Cancer
171060	SNOMED CT	188272000	Malignant tumor of lacrimal gland	Cancer
171061	SNOMED CT	188273005	Malignant neoplasm of lacrimal sac	Cancer
171062	SNOMED CT	188274004	Malignant neoplasm of nasolacrimal duct	Cancer
171068	SNOMED CT	188280007	Malignant neoplasm of cerebrum (excluding lobes and ventricles)	Cancer
171069	SNOMED CT	188281006	Malignant neoplasm of basal ganglia	Cancer
171070	SNOMED CT	188282004	Malignant neoplasm of cerebral cortex	Cancer
171071	SNOMED CT	188283009	Malignant neoplasm of corpus striatum	Cancer
171073	SNOMED CT	188285002	Malignant neoplasm of globus pallidus	Cancer
171074	SNOMED CT	188286001	Malignant tumor of hypothalamus	Cancer
171075	SNOMED CT	188287005	Malignant neoplasm of thalamus	Cancer

171077	SNOMED CT	188289008	Malignant neoplasm of hippocampus	Cancer
171078	SNOMED CT	188290004	Malignant neoplasm of uncus	Cancer
171080	SNOMED CT	188292007	Malignant tumor of choroid plexus	Cancer
171081	SNOMED CT	188293002	Malignant neoplasm of floor of cerebral ventricle	Cancer
171083	SNOMED CT	188295009	Malignant neoplasm of cerebral peduncle	Cancer
171084	SNOMED CT	188296005	Malignant neoplasm of medulla oblongata	Cancer
171085	SNOMED CT	188297001	Malignant neoplasm of midbrain	Cancer
171086	SNOMED CT	188298006	Malignant neoplasm of pons	Cancer
171089	SNOMED CT	188301005	Malignant neoplasm of corpus callosum	Cancer
171090	SNOMED CT	188302003	Malignant neoplasm of tapetum	Cancer
171096	SNOMED CT	188308004	Malignant neoplasm of olfactory bulb	Cancer
171100	SNOMED CT	188312005	Malignant neoplasm of cerebral dura mater	Cancer
171101	SNOMED CT	188313000	Malignant neoplasm of cerebral arachnoid mater	Cancer
171103	SNOMED CT	188315007	Malignant neoplasm of cerebral pia mater	Cancer
171105	SNOMED CT	188317004	Malignant neoplasm of spinal dura mater	Cancer
171106	SNOMED CT	188318009	Malignant neoplasm of spinal arachnoid mater	Cancer
171107	SNOMED CT	188319001	Malignant neoplasm of spinal pia mater	Cancer
171113	SNOMED CT	188325002	Malignant neoplasm of peripheral nerve of thorax	Cancer
171127	SNOMED CT	188339002	Malignant neoplasm of pituitary gland and craniopharyngeal duct	Cancer
171128	SNOMED CT	188340000	Malignant tumor of craniopharyngeal duct	Cancer
171149	SNOMED CT	188361007	Malignant neoplasm of thorax	Cancer
171275	SNOMED CT	188487008	Lymphosarcoma and reticulosarcoma	Cancer
171277	SNOMED CT	188489006	Reticulosarcoma of lymph nodes of head, face and neck	Cancer
171280	SNOMED CT	188492005	Reticulosarcoma of lymph nodes of axilla and upper limb	Cancer
171281	SNOMED CT	188493000	Reticulosarcoma of lymph nodes of inguinal region and lower limb	Cancer

171286	SNOMED CT	188498009	Lymphosarcoma	Cancer
171288	SNOMED CT	188500005	Lymphosarcoma of lymph nodes of head, face and neck	Cancer
171289	SNOMED CT	188501009	Lymphosarcoma of intrathoracic lymph nodes	Cancer
171290	SNOMED CT	188502002	Lymphosarcoma of intra-abdominal lymph nodes	Cancer
171291	SNOMED CT	188503007	Lymphosarcoma of lymph nodes of axilla and upper limb	Cancer
171292	SNOMED CT	188504001	Lymphosarcoma of lymph nodes of inguinal region and lower limb	Cancer
171293	SNOMED CT	188505000	Lymphosarcoma of intrapelvic lymph nodes	Cancer
171294	SNOMED CT	188506004	Lymphosarcoma of spleen	Cancer
171295	SNOMED CT	188507008	Lymphosarcoma of lymph nodes of multiple sites	Cancer
171350	SNOMED CT	188609000	Nodular lymphoma of lymph nodes of head, face and neck	Cancer
171353	SNOMED CT	188612002	Nodular lymphoma of lymph nodes of axilla and upper limb	Cancer
171354	SNOMED CT	188613007	Nodular lymphoma of lymph nodes of inguinal region and lower limb	Cancer
171368	SNOMED CT	188627002	Mycosis fungoides of lymph nodes of multiple sites	Cancer
171372	SNOMED CT	188631008	S√©zary's disease of intrathoracic lymph nodes	Cancer
171373	SNOMED CT	188633006	S√©zary's disease of lymph nodes of axilla AND/OR upper limb	Cancer
171374	SNOMED CT	188634000	S√©zary's disease of lymph nodes of inguinal region and lower limb	Cancer
171375	SNOMED CT	188635004	S√©zary's disease of intrapelvic lymph nodes	Cancer
171377	SNOMED CT	188637007	S√©zary's disease of lymph nodes of multiple sites	Cancer
171381	SNOMED CT	188641006	Malignant histiocytosis of lymph nodes of axilla and upper limb	Cancer
171382	SNOMED CT	188642004	Malignant histiocytosis of lymph nodes of inguinal region and lower limb	Cancer
171385	SNOMED CT	188645002	Leukemic reticuloendotheliosis of lymph nodes of head, face and neck	Cancer
171388	SNOMED CT	188648000	Leukemic reticuloendotheliosis of lymph nodes of axilla and upper limb	Cancer

171389	SNOMED CT	188649008	Leukemic reticuloendotheliosis of lymph nodes of inguinal region and lower limb	Cancer
171402	SNOMED CT	188662007	Mast cell malignancy of lymph nodes of head, face and neck	Cancer
171405	SNOMED CT	188665009	Mast cell malignancy of lymph nodes of axilla and upper limb	Cancer
171406	SNOMED CT	188666005	Mast cell malignancy of lymph nodes of inguinal region and lower limb	Cancer
171408	SNOMED CT	188668006	Mast cell malignancy of spleen	Cancer
171409	SNOMED CT	188669003	Mast cell malignancy of lymph nodes of multiple sites	Cancer
171413	SNOMED CT	188674006	Diffuse malignant lymphoma - small non-cleaved cell	Cancer
171414	SNOMED CT	188675007	Malignant lymphoma - small cleaved cell	Cancer
171415	SNOMED CT	188676008	Malignant lymphoma - mixed small and large cell	Cancer
171456	SNOMED CT	188718006	Extramedullary plasmacytoma	Cancer
171463	SNOMED CT	188725004	Lymphoid leukemia	Cancer
171464	SNOMED CT	188726003	Subacute lymphoid leukemia	Cancer
171466	SNOMED CT	188728002	Aleukemic lymphoid leukemia	Cancer
171467	SNOMED CT	188729005	Adult T-cell leukemia	Cancer
171470	SNOMED CT	188732008	Myeloid leukemia	Cancer
171471	SNOMED CT	188733003	Chronic eosinophilic leukemia	Cancer
171472	SNOMED CT	188734009	Chronic neutrophilic leukemia	Cancer
171474	SNOMED CT	188736006	Subacute myeloid leukemia	Cancer
171475	SNOMED CT	188737002	Chloroma	Cancer
171479	SNOMED CT	188741003	Aleukemic myeloid leukemia	Cancer
171482	SNOMED CT	188744006	Monocytic leukemia	Cancer
171483	SNOMED CT	188745007	Chronic monocytic leukemia	Cancer
171484	SNOMED CT	188746008	Subacute monocytic leukemia	Cancer
171486	SNOMED CT	188748009	Aleukemic monocytic leukemia	Cancer
171492	SNOMED CT	188754005	Megakaryocytic leukemia	Cancer

171506	SNOMED CT	188768003	Myelomonocytic leukemia	Cancer
171508	SNOMED CT	188770007	Subacute myelomonocytic leukemia	Cancer
171900	SNOMED CT	189164002	Cerebral meningioma	Cancer
172130	SNOMED CT	189397006	Neoplasm of uncertain behavior of sphincter of Oddi	Cancer
172139	SNOMED CT	189406008	Neoplasm of uncertain behavior of anal canal and sphincter	Cancer
172242	SNOMED CT	189509003	Refractory anemia without sideroblasts, so stated	Cancer
172747	SNOMED CT	190030009	Compound leukemias	Cancer
20954	SNOMED CT	22288000	Tumor of cervix affecting pregnancy	Cancer
26468	SNOMED CT	28122003	Pulmonary eosinophilic granuloma	Cancer
28845	SNOMED CT	30664006	Multiple endocrine neoplasia, type 1	Cancer
29206	SNOMED CT	31047003	Lymphomatoid papulosis	Cancer
29526	SNOMED CT	31383003	Tumor of vagina affecting pregnancy	Cancer
44036	SNOMED CT	46724008	Polyglandular activity in multiple endocrine adenomatosis	Cancer
55523	SNOMED CT	58961005	Lethal midline granuloma	Cancer
57943	SNOMED CT	61530001	Multiple endocrine neoplasia, type 3	Cancer
58211	SNOMED CT	61808009	Multiple endocrine neoplasia, type 2	Cancer
61082	SNOMED CT	64862009	Congenital rhabdomyoma of heart	Cancer
63991	SNOMED CT	67944007	Lhermitte-Duclos disease	Cancer
290793	SNOMED CT	311779007	Malignant neoplasm of skin of scapular region	Cancer
292228	SNOMED CT	313250007	Malignant neoplasm of lower eyelid	Cancer
292385	SNOMED CT	313427003	Lambda light chain myeloma	Cancer
293742	SNOMED CT	314955001	Local recurrence of malignant tumor of breast	Cancer
293753	SNOMED CT	314966008	Local recurrence of malignant tumor of rectum	Cancer
293773	SNOMED CT	314987003	Metastasis from malignant melanoma of skin	Cancer
303055	SNOMED CT	359780007	Metastatic malignant neoplasm to lateral axillary lymph nodes	Cancer

306578	SNOMED CT	363350007	Malignant tumor of cecum	Cancer
306579	SNOMED CT	363351006	Malignant tumor of rectum	Cancer
306638	SNOMED CT	363411007	Malignant tumor of appendix	Cancer
306677	SNOMED CT	363450006	Malignant tumor of foreskin	Cancer
312584	SNOMED CT	369448007	Malignant tumor involving rectum by direct extension from endometrium	Cancer
312585	SNOMED CT	369449004	Malignant tumor involving rectum by direct extension from fallopian tube	Cancer
312586	SNOMED CT	369450004	Malignant tumor involving rectum by direct extension from ovary	Cancer
312587	SNOMED CT	369451000	Malignant tumor involving rectum by direct extension from prostate	Cancer
312588	SNOMED CT	369452007	Malignant tumor involving rectum by direct extension from uterine cervix	Cancer
312589	SNOMED CT	369453002	Malignant tumor involving rectum by direct extension from uterus	Cancer
312590	SNOMED CT	369454008	Malignant tumor involving rectum by direct extension from vagina	Cancer
312591	SNOMED CT	369455009	Malignant tumor involving rectum by separate metastasis from endometrium	Cancer
312592	SNOMED CT	369456005	Malignant tumor involving rectum by separate metastasis from fallopian tube	Cancer
312593	SNOMED CT	369457001	Malignant tumor involving rectum by separate metastasis from ovary	Cancer
312594	SNOMED CT	369458006	Malignant tumor involving rectum by separate metastasis from prostate	Cancer
312595	SNOMED CT	369459003	Malignant tumor involving rectum by separate metastasis from uterine cervix	Cancer
312596	SNOMED CT	369460008	Malignant tumor involving rectum by separate metastasis from uterus	Cancer
312597	SNOMED CT	369461007	Malignant tumor involving rectum by separate metastasis from vagina	Cancer
312609	SNOMED CT	369473007	Malignant tumor involving bladder by direct extension from uterine cervix	Cancer

312610	SNOMED CT	369474001	Malignant tumor involving bladder by direct extension from uterus	Cancer
312624	SNOMED CT	369488002	Secondary malignant neoplasm of seminal vesicle	Cancer
312650	SNOMED CT	369514009	Secondary malignant neoplasm of left fallopian tube	Cancer
312657	SNOMED CT	369521009	Secondary malignant neoplasm of right fallopian tube	Cancer
312659	SNOMED CT	369523007	Secondary malignant neoplasm of left ovary	Cancer
312666	SNOMED CT	369530001	Secondary malignant neoplasm of right ovary	Cancer
312671	SNOMED CT	369535006	Secondary neoplasm of left broad ligament	Cancer
312672	SNOMED CT	369536007	Secondary neoplasm of right broad ligament	Cancer
312678	SNOMED CT	369542006	Malignant tumor involving left fallopian tube by separate metastasis from endometrium	Cancer
312679	SNOMED CT	369543001	Malignant tumor involving left fallopian tube by separate metastasis from ovary	Cancer
312680	SNOMED CT	369544007	Malignant tumor involving left fallopian tube by separate metastasis from right fallopian tube	Cancer
312681	SNOMED CT	369545008	Malignant tumor involving left fallopian tube by separate metastasis from uterus	Cancer
312682	SNOMED CT	369546009	Malignant tumor involving left fallopian tube by separate metastasis from vagina	Cancer
312689	SNOMED CT	369553000	Malignant tumor involving right fallopian tube by separate metastasis from endometrium	Cancer
312690	SNOMED CT	369554006	Malignant tumor involving right fallopian tube by separate metastasis from left fallopian tube	Cancer
312691	SNOMED CT	369555007	Malignant tumor involving right fallopian tube by separate metastasis from ovary	Cancer
312692	SNOMED CT	369556008	Malignant tumor involving right fallopian tube by separate metastasis from uterine cervix	Cancer
312693	SNOMED CT	369557004	Malignant tumor involving right fallopian tube by separate metastasis from uterus	Cancer

312694	SNOMED CT	369558009	Malignant tumor involving right fallopian tube by separate metastasis from vagina	Cancer
312696	SNOMED CT	369560006	Malignant tumor involving left ovary by separate metastasis from endometrium	Cancer
312697	SNOMED CT	369561005	Malignant tumor involving left ovary by separate metastasis from fallopian tube	Cancer
312698	SNOMED CT	369562003	Malignant tumor involving left ovary by separate metastasis from right ovary	Cancer
312699	SNOMED CT	369563008	Malignant tumor involving left ovary by separate metastasis from uterine cervix	Cancer
312700	SNOMED CT	369564002	Malignant tumor involving left ovary by separate metastasis from vagina	Cancer
312701	SNOMED CT	369565001	Malignant tumor involving left ovary by separate metastasis uterus	Cancer
312704	SNOMED CT	369568004	Malignant tumor involving right ovary by separate metastasis from endometrium	Cancer
312705	SNOMED CT	369569007	Malignant tumor involving right ovary by separate metastasis from fallopian tube	Cancer
312706	SNOMED CT	369570008	Malignant tumor involving right ovary by separate metastasis from left ovary	Cancer
312707	SNOMED CT	369571007	Malignant tumor involving right ovary by separate metastasis from uterine cervix	Cancer
312708	SNOMED CT	369572000	Malignant tumor involving right ovary by separate metastasis from uterus	Cancer
312709	SNOMED CT	369573005	Malignant tumor involving right ovary by separate metastasis from vagina	Cancer
312717	SNOMED CT	369581006	Malignant tumor involving vagina by separate metastasis from bladder	Cancer
312718	SNOMED CT	369582004	Malignant tumor involving vagina by separate metastasis from endometrium	Cancer
312719	SNOMED CT	369583009	Malignant tumor involving vagina by separate metastasis from fallopian tube	Cancer
312720	SNOMED CT	369584003	Malignant tumor involving vagina by separate metastasis from ovary	Cancer

312721	SNOMED CT	369585002	Malignant tumor involving vagina by separate metastasis from uterine cervix	Cancer
312722	SNOMED CT	369586001	Malignant tumor involving vagina by separate metastasis from uterus	Cancer
312724	SNOMED CT	369588000	Malignant tumor involving vulva by separate metastasis from endometrium	Cancer
312725	SNOMED CT	369589008	Malignant tumor involving vulva by separate metastasis from fallopian tube	Cancer
312726	SNOMED CT	369590004	Malignant tumor involving vulva by separate metastasis from ovary	Cancer
312727	SNOMED CT	369591000	Malignant tumor involving vulva by separate metastasis from uterine cervix	Cancer
312728	SNOMED CT	369592007	Malignant tumor involving vulva by separate metastasis from uterus	Cancer
312729	SNOMED CT	369593002	Malignant tumor involving vulva by separate metastasis from vagina	Cancer
312746	SNOMED CT	369610009	Malignant tumor involving left fallopian tube by separate metastasis from uterine cervix	Cancer
314774	SNOMED CT	371977004	Primary malignant neoplasm of cecum	Cancer
314820	SNOMED CT	372023003	Primary malignant neoplasm of upper third of esophagus	Cancer
314889	SNOMED CT	372092003	Primary malignant neoplasm of axillary tail of breast	Cancer
314890	SNOMED CT	372093008	Secondary malignant neoplasm of axillary tail of breast	Cancer
314891	SNOMED CT	372094002	Malignant neoplasm of axillary tail of breast	Cancer
314892	SNOMED CT	372095001	Malignant neoplasm of male breast	Cancer
314893	SNOMED CT	372096000	Carcinoma of male breast	Cancer
314909	SNOMED CT	372112000	Primary malignant neoplasm of middle lobe, bronchus or lung	Cancer
314910	SNOMED CT	372113005	Carcinoma of middle lobe, bronchus or lung	Cancer
314930	SNOMED CT	372135002	Primary malignant neoplasm of upper lobe, bronchus or lung	Cancer
314931	SNOMED CT	372136001	Carcinoma of upper lobe, bronchus or lung	Cancer
314932	SNOMED CT	372137005	Primary malignant neoplasm of breast	Cancer

315738	SNOMED CT	373080008	Malignant neoplasm of breast lower inner quadrant	Cancer
315739	SNOMED CT	373081007	Malignant neoplasm of breast lower outer quadrant	Cancer
315740	SNOMED CT	373082000	Malignant neoplasm of breast upper inner quadrant	Cancer
315741	SNOMED CT	373083005	Malignant neoplasm of breast upper outer quadrant	Cancer
315746	SNOMED CT	373088001	Primary malignant neoplasm of breast upper outer quadrant	Cancer
315747	SNOMED CT	373089009	Primary malignant neoplasm of breast upper inner quadrant	Cancer
315748	SNOMED CT	373090000	Primary malignant neoplasm of breast lower inner quadrant	Cancer
315749	SNOMED CT	373091001	Primary malignant neoplasm of breast lower outer quadrant	Cancer
320198	SNOMED CT	385427008	Osteochondroma of elbow	Cancer
320199	SNOMED CT	385428003	Osteochondroma of femur	Cancer
320200	SNOMED CT	385429006	Osteochondroma of foot	Cancer
320201	SNOMED CT	385430001	Osteochondroma of scapula	Cancer
320202	SNOMED CT	385431002	Osteochondroma of tibia	Cancer
323120	SNOMED CT	388872005	Secondary optic nerve sheath meningioma	Cancer
323516	SNOMED CT	389272007	Carpotarsal osteochondromatosis	Cancer
335903	SNOMED CT	402517006	Basal cell carcinoma of upper back	Cancer
336226	SNOMED CT	402841001	Angiokeratoma of vulva	Cancer
336535	SNOMED CT	403274000	Hypomelanosis surrounding malignant melanoma	Cancer
336719	SNOMED CT	403458008	Localized skin involvement by breast carcinoma	Cancer
337149	SNOMED CT	403918002	Basal cell carcinoma of glabella	Cancer
337177	SNOMED CT	403946000	Paget's disease of nipple	Cancer
337367	SNOMED CT	404145009	Primary cutaneous anaplastic large cell B-cell lymphoma	Cancer
339248	SNOMED CT	406103009	Squamous cell carcinoma in situ of uterine cervix	Cancer
341547	SNOMED CT	408643008	Infiltrating duct carcinoma of breast	Cancer
341549	SNOMED CT	408645001	Adenocarcinoma of large intestine	Cancer

346082	SNOMED CT	413445002	Adenocarcinoma of appendix	Cancer
346083	SNOMED CT	413446001	Adenocarcinoma of cecum	Cancer
346208	SNOMED CT	413587002	Smoldering myeloma	Cancer
347085	SNOMED CT	414553000	Kappa light chain myeloma	Cancer
347606	SNOMED CT	415110002	Plasma cell myeloma/plasmacytoma	Cancer
348998	SNOMED CT	416769008	Malignant teratoma of testis	Cancer
349142	SNOMED CT	416921001	Carcinoma in situ of nasolacrimal duct	Cancer
349392	SNOMED CT	417181009	Hormone receptor positive malignant neoplasm of breast	Cancer
349441	SNOMED CT	417234002	Benign sacral teratoma	Cancer
350620	SNOMED CT	418529003	Secondary malignant neoplasm of lacrimal drainage structure	Cancer
354433	SNOMED CT	422607004	Carcinoma in situ of lacrimal drainage system	Cancer
354714	SNOMED CT	422893006	Carcinoma in situ of lacrimal gland duct	Cancer
355131	SNOMED CT	423318000	Adenoid cystic carcinoma of oropharynx	Cancer
355197	SNOMED CT	423384009	Secondary malignant neoplasm of lacrimal gland duct	Cancer
355347	SNOMED CT	423535002	Basal cell carcinoma of chest wall	Cancer
355604	SNOMED CT	423793008	Mucoepidermoid carcinoma of parotid gland	Cancer
357417	SNOMED CT	425657001	Osteosclerotic myeloma	Cancer
359399	SNOMED CT	427685000	HER2-positive carcinoma of breast	Cancer
362833	SNOMED CT	431396003	Human epidermal growth factor 2 negative carcinoma of breast	Cancer
368568	SNOMED CT	440422002	Asymptomatic multiple myeloma	Cancer
369446	SNOMED CT	441313008	Indolent multiple myeloma	Cancer
371398	SNOMED CT	443333004	Medulloblastoma	Cancer
372410	SNOMED CT	444374006	Type C thymoma	Cancer
372606	SNOMED CT	444596001	Malignant thymoma	Cancer
372614	SNOMED CT	444604002	Mixed ductal and lobular carcinoma of breast	Cancer
372721	SNOMED CT	444712000	Mucinous carcinoma of breast	Cancer

373098	SNOMED CT	445105005	Blastic plasmacytoid dendritic cell neoplasm	Cancer
374875	SNOMED CT	446939001	Chordoma of clivus	Cancer
374881	SNOMED CT	446945009	Invasive pituitary adenoma	Cancer
375636	SNOMED CT	447729009	Ameloblastoma of mandible	Cancer
375637	SNOMED CT	447730004	Chordoma of sacrum	Cancer
375699	SNOMED CT	447792005	Chondrosarcoma of bone	Cancer
376034	SNOMED CT	448135004	Benign teratoma of pineal region	Cancer
376155	SNOMED CT	448257000	Sarcoma of male breast	Cancer
376194	SNOMED CT	448296006	Dermatofibrosarcoma protuberans of skin of chest	Cancer
376212	SNOMED CT	448314007	Carcinoma of spinal cord	Cancer
376452	SNOMED CT	448563005	Functionless pituitary neoplasm	Cancer
376876	SNOMED CT	448992002	Carcinoma of appendix	Cancer
376878	SNOMED CT	448994001	Carcinoma of upper rectum	Cancer
377488	SNOMED CT	449627008	Malignant neoplasm of long bone of lower limb	Cancer
377660	SNOMED CT	449799008	Subependymal giant cell astrocytoma	Cancer
377664	SNOMED CT	449803009	Primary malignant neoplasm of dome of urinary bladder	Cancer
386876	SNOMED CT	608817003	Pituicytoma	Cancer
387772	SNOMED CT	697993003	Undifferentiated carcinoma of nasal sinus	Cancer
387790	SNOMED CT	698011002	Keratinizing squamous cell carcinoma of nasopharynx	Cancer
387819	SNOMED CT	698040004	Malignant melanoma of nasal cavity	Cancer
387820	SNOMED CT	698041000	Malignant melanoma of vestibule of mouth	Cancer
387821	SNOMED CT	698042007	Malignant melanoma of tongue	Cancer
387822	SNOMED CT	698043002	Malignant melanoma of floor of mouth	Cancer
387823	SNOMED CT	698044008	Malignant melanoma of palatine arch	Cancer
387824	SNOMED CT	698045009	Malignant melanoma of buccal mucosa	Cancer
387827	SNOMED CT	698048006	Undifferentiated nonkeratinizing carcinoma of nasopharynx	Cancer

387975	SNOMED CT	698200003	Large cell Ewing sarcoma of bone	Cancer
388057	SNOMED CT	698285005	Malignant melanoma of ethmoid sinus	Cancer
388058	SNOMED CT	698286006	Malignant melanoma of palate	Cancer
388059	SNOMED CT	698287002	Malignant melanoma of gum	Cancer
388060	SNOMED CT	698288007	Malignant melanoma of maxillary sinus	Cancer
388452	SNOMED CT	698770000	Multicystic intraosseous ameloblastoma	Cancer
2930795	SNOMED CT	1.197E+09	Metastatic carcinoma of thoracic lymph node	Cancer
2930796	SNOMED CT	1.197E+09	Metastatic malignant melanoma of skin	Cancer
2930800	SNOMED CT	1.197E+09	Metastatic squamous cell carcinoma of lymph nodes of head and neck	Cancer
2932022	SNOMED CT	1.208E+09	Small cell neuroendocrine carcinoma of prostate	Cancer
2928528	SNOMED CT	1.197E+09	Benign neoplasm of lacrimal apparatus	Cancer
2932024	SNOMED CT	1.209E+09	SMARCA4-deficient undifferentiated neoplasm of thorax	Cancer
2930803	SNOMED CT	1.197E+09	Metastatic squamous cell carcinoma of thoracic lymph node	Cancer
2932364	SNOMED CT	1.197E+09	T-cell lymphoma of small intestine	Cancer
2928922	SNOMED CT	1.202E+09	Chondrosarcoma of gingiva	Cancer
2931304	SNOMED CT	1.208E+09	Plasmacytoid urothelial carcinoma of urinary bladder	Cancer
2931305	SNOMED CT	1.197E+09	Pleomorphic adenoma of lacrimal system	Cancer
2929763	SNOMED CT	3.532E+14	Extramedullary plasmacytoma in remission	Cancer
2645082	SNOMED CT	1.179E+09	Adenocarcinoma of parathyroid gland	Cancer
2645083	SNOMED CT	1.179E+09	Adenocarcinoma of pituitary gland	Cancer
2647397	SNOMED CT	1.187E+09	Mixed phenotype acute leukemia	Cancer
2646777	SNOMED CT	1.173E+09	High grade B-cell lymphoma with MYC and BCL2 and/or BCL6 rearrangements	Cancer
2647223	SNOMED CT	1.162E+09	Malignant lymphoma of uveal tract	Cancer
2647712	SNOMED CT	1.162E+09	Osteosarcoma of articular cartilage of rib	Cancer

2647714	SNOMED CT	1.162E+09	Osteosarcoma of articular cartilage of vertebra	Cancer
2647715	SNOMED CT	1.162E+09	Osteosarcoma of bone of clavicle	Cancer
2647716	SNOMED CT	1.162E+09	Osteosarcoma of bone of rib	Cancer
2647717	SNOMED CT	1.162E+09	Osteosarcoma of bone of vertebra	Cancer
2647718	SNOMED CT	1.162E+09	Osteosarcoma of sternum	Cancer
2648374	SNOMED CT	1.162E+09	Rhabdomyosarcoma of endometrium of corpus uteri	Cancer
2648375	SNOMED CT	1.162E+09	Rhabdomyosarcoma of fundus uteri	Cancer
2648450	SNOMED CT	1.162E+09	Sarcoma of corpus uteri	Cancer
2645758	SNOMED CT	1.187E+09	Clear cell sarcoma of kidney	Cancer
214020	SNOMED CT	231828003	Capillary hemangioma of eyelid	Cancer
214634	SNOMED CT	232457008	Laryngeal papillomatosis	Cancer
216108	SNOMED CT	233940007	Pulmonary tumor embolism	Cancer
219819	SNOMED CT	237721006	Tumor of pituitary and suprasellar region	Cancer
224838	SNOMED CT	242862004	Secondary malignant neoplasm of nasopharyngeal wall	Cancer
234590	SNOMED CT	253010003	Microprolactinoma	Cancer
234591	SNOMED CT	253011004	Macroprolactinoma	Cancer
235987	SNOMED CT	254436005	Carcinoma of uvula	Cancer
236054	SNOMED CT	254504001	Benign tumor of inferior surface of soft palate	Cancer
236132	SNOMED CT	254582000	Adenocarcinoma of rectum	Cancer
236383	SNOMED CT	254837009	Malignant tumor of breast	Cancer
236384	SNOMED CT	254838004	Carcinoma of breast	Cancer
236385	SNOMED CT	254839007	Scirrhous carcinoma of breast	Cancer
236386	SNOMED CT	254840009	Inflammatory carcinoma of breast	Cancer
236387	SNOMED CT	254841008	Cancer en cuirasse	Cancer
236389	SNOMED CT	254843006	Familial cancer of breast	Cancer
236390	SNOMED CT	254844000	Malignant phyllodes tumor of breast	Cancer

236484	SNOMED CT	254939008	Ependymoma of brain	Cancer
236494	SNOMED CT	254949006	Ependymoma of spinal cord	Cancer
236537	SNOMED CT	254992001	Pleomorphic adenoma of lacrimal gland	Cancer
236612	SNOMED CT	255068000	Carcinoma of bone, connective tissue, skin and breast	Cancer
236625	SNOMED CT	255081007	Carcinoma of cecum	Cancer
236645	SNOMED CT	255101006	S√©zary disease of skin	Cancer
236646	SNOMED CT	255102004	Angioendotheliomatosis	Cancer
236651	SNOMED CT	255107005	Seminoma of testis	Cancer
236739	SNOMED CT	255200003	Benign tumor of hypothalamus	Cancer
251865	SNOMED CT	271467005	Malignant neoplasm of bone, connective tissue, skin and breast	Cancer
251877	SNOMED CT	271479005	Benign neoplasm of pituitary gland and craniopharyngeal duct	Cancer
256997	SNOMED CT	276822007	Malignant melanoma of rectum	Cancer
257733	SNOMED CT	277579002	Light chain myeloma	Cancer
257734	SNOMED CT	277580004	Non-secretory myeloma	Cancer
258194	SNOMED CT	278050001	Sarcoma of breast	Cancer
258198	SNOMED CT	278054005	Infiltrating lobular carcinoma of breast	Cancer
261060	SNOMED CT	280960002	Benign tumor of lacrimal drainage structure	Cancer
265615	SNOMED CT	285610008	Metastasis to large intestine of unknown primary	Cancer
265617	SNOMED CT	285612000	Metastasis to rectum of unknown primary	Cancer
265643	SNOMED CT	285638000	Metastasis to vagina of unknown primary	Cancer
266873	SNOMED CT	286893002	Carcinoma of breast - upper, inner quadrant	Cancer
266874	SNOMED CT	286894008	Carcinoma of breast - lower, inner quadrant	Cancer
266875	SNOMED CT	286895009	Carcinoma of breast - upper, outer quadrant	Cancer
266876	SNOMED CT	286896005	Carcinoma breast - lower, outer quadrant	Cancer
266877	SNOMED CT	286897001	Carcinoma of breast - axillary tail	Cancer

287108	SNOMED CT	307591004	Malignant mastocytosis	Cancer
2930951	SNOMED CT	1.23E+09	Myeloid and/or lymphoid neoplasm with PCM1-JAK2	Cancer
119778	SNOMED CT	134209002	Prolactinoma	Cancer
119987	SNOMED CT	134421000	Pathologic fracture of bone at site of metastatic neoplasm	Cancer
2931494	SNOMED CT	3.526E+14	Primary malignant neoplasm of right descended testis	Cancer
2645926	SNOMED CT	1.177E+09	Congenital progressive bone marrow failure, B-cell immunodeficiency, skeletal dysplasia syndrome	Cancer
2645073	SNOMED CT	1.163E+09	Acute myeloid leukemia in complete remission	Cancer
2928971	SNOMED CT	1.231E+09	Clear cell urothelial carcinoma of urinary system	Cancer
2929781	SNOMED CT	1.197E+09	Familial colorectal cancer type X	Cancer
2929788	SNOMED CT	1.197E+09	Familial ovarian cancer	Cancer
2930050	SNOMED CT	1.217E+09	HER2-expressing (human epidermal growth factor 2-expressing) colorectal malignant neoplasm	Cancer
2930426	SNOMED CT	1.217E+09	Kaposi sarcoma of gingiva	Cancer
2930614	SNOMED CT	1.223E+09	Lipid-rich urothelial carcinoma of urinary system	Cancer
2930660	SNOMED CT	1.197E+09	Lymphangioma of conjunctiva	Cancer
2930684	SNOMED CT	1.453E+17	Malignant carcinoid tumor of left lung	Cancer
2930685	SNOMED CT	7.932E+17	Malignant carcinoid tumor of right lung	Cancer
2930686	SNOMED CT	1.237E+14	Malignant carcinoid tumor of sigmoid colon	Cancer
2930687	SNOMED CT	1.197E+09	Malignant melanoma arising in melanocytic nevus	Cancer
2930688	SNOMED CT	1.197E+09	Malignant melanoma of uveal tract	Cancer
2930785	SNOMED CT	1.197E+09	Metastatic adenocarcinoma of lymph node of head and neck	Cancer
2930790	SNOMED CT	1.197E+09	Metastatic adenocarcinoma of thoracic lymph node	Cancer
2930792	SNOMED CT	1.197E+09	Metastatic carcinoma of lymph node of head and neck	Cancer
2931151	SNOMED CT	1.197E+09	Oxyphilic adenoma of lacrimal system	Cancer

2931466	SNOMED CT	1.197E+09	Primary biphasic malignant mesothelioma of pleura	Cancer
2931469	SNOMED CT	1.208E+09	Primary choriocarcinoma of central nervous system	Cancer
2931472	SNOMED CT	1.197E+09	Primary desmoplastic mesothelioma of pleura	Cancer
2931475	SNOMED CT	1.197E+09	Primary embryonal carcinoma of brain	Cancer
2931477	SNOMED CT	1.197E+09	Primary epithelioid malignant mesothelioma of pleura	Cancer
2931484	SNOMED CT	1.197E+09	Primary malignant astrocytoma of brain	Cancer
2931485	SNOMED CT	1.197E+09	Primary malignant atypical teratoid rhabdoid neoplasm of brain	Cancer
2931486	SNOMED CT	1.231E+09	Primary malignant ependymoma of optic nerve	Cancer
2931487	SNOMED CT	1.197E+09	Primary malignant germ cell neoplasm of pineal gland	Cancer
2931488	SNOMED CT	1.197E+09	Primary malignant germ cell neoplasm of pleura	Cancer
2931489	SNOMED CT	1.197E+09	Primary malignant melanoma of central nervous system	Cancer
2931490	SNOMED CT	1.197E+09	Primary malignant mesenchymal neoplasm of colon	Cancer
2931491	SNOMED CT	1.197E+09	Primary malignant mesenchymal neoplasm of rectum	Cancer
2931499	SNOMED CT	1.197E+09	Primary papillary carcinoma of body of uterus	Cancer
2931500	SNOMED CT	1.197E+09	Primary papillary carcinoma of cervix uteri	Cancer
2931501	SNOMED CT	1.197E+09	Primary poorly differentiated endocrine carcinoma of cervix uteri	Cancer
2931502	SNOMED CT	1.197E+09	Primary poorly differentiated endocrine carcinoma of corpus uteri	Cancer
2931503	SNOMED CT	1.197E+09	Primary salivary gland type carcinoma of palate	Cancer
2931504	SNOMED CT	1.197E+09	Primary sarcomatoid malignant mesothelioma of pleura	Cancer
2931506	SNOMED CT	1.197E+09	Primary squamous cell carcinoma of fallopian tube	Cancer
2931507	SNOMED CT	1.23E+09	Primary squamous cell carcinoma of nasal cavity and paranasal sinus	Cancer
2931508	SNOMED CT	1.69E+16	Primary squamous cell carcinoma of skin of chest	Cancer
2931509	SNOMED CT	1.197E+09	Primary squamous cell carcinoma of Waldeyer ring	Cancer

2931511	SNOMED CT	1.197E+09	Primary teratoma of brain	Cancer
2931912	SNOMED CT	1.217E+09	Sarcomatoid urothelial carcinoma of ureter	Cancer
2931913	SNOMED CT	1.208E+09	Sarcomatoid urothelial carcinoma of urinary bladder	Cancer
2931977	SNOMED CT	1.23E+09	Serous carcinoma of body of uterus	Cancer
2932450	SNOMED CT	1.208E+09	Transitional cell carcinoma of upper urinary tract	Cancer
2932618	SNOMED CT	1.197E+09	Yolk sac tumor of central nervous system	Cancer
2928464	SNOMED CT	1.197E+09	B cell lymphoma of small intestine	Cancer
2931456	SNOMED CT	1.197E+09	Primary acinar cell carcinoma of parotid gland	Cancer
2931458	SNOMED CT	1.197E+09	Primary adenocarcinoma of iris neuroepithelium	Cancer
2931462	SNOMED CT	6.825E+14	Primary astrocytoma of occipital lobe	Cancer
2931459	SNOMED CT	6.819E+14	Primary adenocarcinoma of posterior wall of urinary bladder	Cancer
2647227	SNOMED CT	1.18E+09	Malignant neoplasm of middle lobe of right lung	Cancer
2931461	SNOMED CT	6.824E+14	Primary astrocytoma of cerebral ventricle	Cancer
2931492	SNOMED CT	1.197E+09	Primary malignant M√ºllerian mixed neoplasm of cervix uteri	Cancer
2931493	SNOMED CT	3.526E+14	Primary malignant neoplasm of left descended testis	Cancer
2931460	SNOMED CT	1.197E+09	Primary anaplastic large cell medulloblastoma of brain	Cancer
2647228	SNOMED CT	1.163E+09	Malignant neoplasm of oral cavity and lip and salivary gland	Cancer
2648225	SNOMED CT	1.187E+09	Primary Kaposi sarcoma of palate	Cancer
2648228	SNOMED CT	1.164E+09	Primary malignant neoplasm of overlapping sites of colon	Cancer
64967	SNOMED CT	68979007	Heavy chain disease	Cancer
77729	SNOMED CT	82546001	Reactive immunoproliferative disease	Cancer
86746	SNOMED CT	92072003	Benign neoplasm of craniopharyngeal duct	Cancer
86843	SNOMED CT	92169007	Benign neoplasm of lacrimal gland	Cancer
86969	SNOMED CT	92296004	Benign neoplasm of pituitary gland	Cancer
87226	SNOMED CT	92564006	Carcinoma in situ of uterine cervix	Cancer

87235	SNOMED CT	92573003	Carcinoma in situ of craniopharyngeal duct	Cancer
87242	SNOMED CT	92580001	Carcinoma in situ of endocervix	Cancer
87250	SNOMED CT	92588008	Carcinoma in situ of exocervix	Cancer
87290	SNOMED CT	92628002	Carcinoma in situ of lacrimal gland	Cancer
87343	SNOMED CT	92683008	Carcinoma in situ of pituitary gland	Cancer
87451	SNOMED CT	92791005	Carcinoma in situ of vagina	Cancer
88273	SNOMED CT	93675008	Primary malignant neoplasm of anterior wall of urinary bladder	Cancer
88277	SNOMED CT	93679002	Primary malignant neoplasm of appendix	Cancer
88279	SNOMED CT	93681000	Primary malignant neoplasm of areola of male breast	Cancer
88281	SNOMED CT	93683002	Primary malignant neoplasm of ascending colon	Cancer
88349	SNOMED CT	93751003	Primary malignant neoplasm of cervical vertebral column	Cancer
88375	SNOMED CT	93777006	Primary malignant neoplasm of ectopic male breast tissue	Cancer
88452	SNOMED CT	93854002	Primary malignant neoplasm of large intestine	Cancer
88461	SNOMED CT	93863000	Primary malignant neoplasm of lateral wall of urinary bladder	Cancer
88476	SNOMED CT	93878007	Primary malignant neoplasm of lumbar vertebral column	Cancer
88482	SNOMED CT	93884005	Primary malignant neoplasm of male breast	Cancer
88511	SNOMED CT	93914000	Primary malignant neoplasm of myocardium	Cancer
88522	SNOMED CT	93925009	Primary malignant neoplasm of nipple of male breast	Cancer
88569	SNOMED CT	93972009	Primary malignant neoplasm of posterior wall of urinary bladder	Cancer
88581	SNOMED CT	93984006	Primary malignant neoplasm of rectum	Cancer
88601	SNOMED CT	94004004	Primary malignant neoplasm of short bone of upper limb	Cancer
88609	SNOMED CT	94012007	Primary malignant neoplasm of skin of breast	Cancer
88613	SNOMED CT	94016005	Primary malignant neoplasm of skin of chin	Cancer
88629	SNOMED CT	94032008	Primary malignant neoplasm of skin of lip	Cancer
88748	SNOMED CT	94152006	Secondary malignant neoplasm of abdominal esophagus	Cancer

88751	SNOMED CT	94155008	Secondary malignant neoplasm of accessory sinus	Cancer
88753	SNOMED CT	94157000	Secondary malignant neoplasm of acromion	Cancer
88754	SNOMED CT	94158005	Secondary malignant neoplasm of adenoid	Cancer
88756	SNOMED CT	94160007	Secondary malignant neoplasm of adrenal cortex	Cancer
88757	SNOMED CT	94161006	Secondary malignant neoplasm of adrenal gland	Cancer
88758	SNOMED CT	94162004	Secondary malignant neoplasm of adrenal medulla	Cancer
88759	SNOMED CT	94163009	Secondary malignant neoplasm of alveolar ridge mucosa	Cancer
88760	SNOMED CT	94164003	Secondary malignant neoplasm of ampulla of Vater	Cancer
88761	SNOMED CT	94165002	Secondary malignant neoplasm of anal canal	Cancer
88762	SNOMED CT	94166001	Secondary malignant neoplasm of anterior aspect of epiglottis	Cancer
88763	SNOMED CT	94167005	Secondary malignant neoplasm of anterior mediastinum	Cancer
88764	SNOMED CT	94168000	Secondary malignant neoplasm of anterior portion of floor of mouth	Cancer
88765	SNOMED CT	94169008	Secondary malignant neoplasm of anterior two-thirds of tongue	Cancer
88766	SNOMED CT	94170009	Secondary malignant neoplasm of anterior wall of nasopharynx	Cancer
88767	SNOMED CT	94171008	Secondary malignant neoplasm of anterior wall of urinary bladder	Cancer
88768	SNOMED CT	94172001	Secondary malignant neoplasm of anus	Cancer
88771	SNOMED CT	94175004	Secondary malignant neoplasm of appendix	Cancer
88772	SNOMED CT	94176003	Secondary malignant neoplasm of areola of female breast	Cancer
88773	SNOMED CT	94177007	Secondary malignant neoplasm of areola of male breast	Cancer
88776	SNOMED CT	94180008	Secondary malignant neoplasm of axilla	Cancer
88777	SNOMED CT	94181007	Secondary malignant neoplasm of axillary lymph nodes	Cancer
88778	SNOMED CT	94182000	Secondary malignant neoplasm of axillary tail of female breast	Cancer
88780	SNOMED CT	94184004	Secondary malignant neoplasm of base of tongue	Cancer
88782	SNOMED CT	94186002	Secondary malignant neoplasm of bladder	Cancer

88785	SNOMED CT	94189009	Secondary malignant neoplasm of blood vessel of axilla	Cancer
88803	SNOMED CT	94207003	Secondary malignant neoplasm of blood vessel of thorax	Cancer
88809	SNOMED CT	94213007	Secondary malignant neoplasm of body of penis	Cancer
88810	SNOMED CT	94214001	Secondary malignant neoplasm of body of stomach	Cancer
88811	SNOMED CT	94215000	Secondary malignant neoplasm of body of uterus	Cancer
88814	SNOMED CT	94218003	Secondary malignant neoplasm of bone of face	Cancer
88815	SNOMED CT	94219006	Secondary malignant neoplasm of bone of lower limb	Cancer
88816	SNOMED CT	94220000	Secondary malignant neoplasm of bone of skull	Cancer
88817	SNOMED CT	94221001	Secondary malignant neoplasm of bone of upper limb	Cancer
88818	SNOMED CT	94222008	Secondary malignant neoplasm of bone	Cancer
88820	SNOMED CT	94224009	Secondary malignant neoplasm of brain stem	Cancer
88821	SNOMED CT	94225005	Secondary malignant neoplasm of brain	Cancer
88822	SNOMED CT	94226006	Secondary malignant neoplasm of broad ligament	Cancer
88823	SNOMED CT	94227002	Secondary malignant neoplasm of bronchopulmonary lymph nodes	Cancer
88824	SNOMED CT	94228007	Secondary malignant neoplasm of bronchus of left lower lobe	Cancer
88825	SNOMED CT	94229004	Secondary malignant neoplasm of bronchus of left upper lobe	Cancer
88826	SNOMED CT	94230009	Secondary malignant neoplasm of bronchus of right lower lobe	Cancer
88827	SNOMED CT	94231008	Secondary malignant neoplasm of bronchus of right middle lobe	Cancer
88828	SNOMED CT	94232001	Secondary malignant neoplasm of bronchus of right upper lobe	Cancer
88829	SNOMED CT	94233006	Secondary malignant neoplasm of bronchus	Cancer
88830	SNOMED CT	94234000	Secondary malignant neoplasm of buccal mucosa	Cancer
88831	SNOMED CT	94235004	Secondary malignant neoplasm of cecum	Cancer
88832	SNOMED CT	94236003	Secondary malignant neoplasm of calcaneus	Cancer
88833	SNOMED CT	94237007	Secondary malignant neoplasm of cardia of stomach	Cancer

88834	SNOMED CT	94238002	Secondary malignant neoplasm of carina	Cancer
88836	SNOMED CT	94240007	Secondary malignant neoplasm of carpal bone	Cancer
88837	SNOMED CT	94241006	Secondary malignant neoplasm of cartilage of nose	Cancer
88839	SNOMED CT	94243009	Secondary malignant neoplasm of central nervous system	Cancer
88840	SNOMED CT	94244003	Secondary malignant neoplasm of central portion of female breast	Cancer
88841	SNOMED CT	94245002	Secondary malignant neoplasm of cerebellum	Cancer
88842	SNOMED CT	94246001	Secondary malignant neoplasm of cerebral meninges	Cancer
88843	SNOMED CT	94247005	Secondary malignant neoplasm of cerebral ventricle	Cancer
88844	SNOMED CT	94248000	Secondary malignant neoplasm of cerebrum	Cancer
88845	SNOMED CT	94249008	Secondary malignant neoplasm of cervical esophagus	Cancer
88846	SNOMED CT	94250008	Secondary malignant neoplasm of cervical vertebral column	Cancer
88849	SNOMED CT	94253005	Secondary malignant neoplasm of chest wall	Cancer
88850	SNOMED CT	94254004	Secondary malignant neoplasm of choroid	Cancer
88851	SNOMED CT	94255003	Secondary malignant neoplasm of ciliary body	Cancer
88852	SNOMED CT	94256002	Secondary malignant neoplasm of clavicle	Cancer
88853	SNOMED CT	94257006	Secondary malignant neoplasm of clitoris	Cancer
88855	SNOMED CT	94259009	Secondary malignant neoplasm of coccyx	Cancer
88857	SNOMED CT	94261000	Secondary malignant neoplasm of commissure of lip	Cancer
88859	SNOMED CT	94263002	Secondary malignant neoplasm of conjunctiva	Cancer
88861	SNOMED CT	94265009	Secondary malignant neoplasm of cornea	Cancer
88863	SNOMED CT	94267001	Secondary malignant neoplasm of craniopharyngeal duct	Cancer
88865	SNOMED CT	94269003	Secondary malignant neoplasm of cuboid	Cancer
88868	SNOMED CT	94272005	Secondary malignant neoplasm of diaphragm	Cancer
88869	SNOMED CT	94273000	Secondary malignant neoplasm of dorsal surface of tongue	Cancer

88870	SNOMED CT	94274006	Secondary malignant neoplasm of thoracic vertebral column	Cancer
88871	SNOMED CT	94275007	Secondary malignant neoplasm of duodenum	Cancer
88872	SNOMED CT	94276008	Secondary malignant neoplasm of ectopic female breast tissue	Cancer
88873	SNOMED CT	94277004	Secondary malignant neoplasm of ectopic male breast tissue	Cancer
88874	SNOMED CT	94278009	Secondary malignant neoplasm of endocardium	Cancer
88875	SNOMED CT	94279001	Secondary malignant neoplasm of endocervix	Cancer
88876	SNOMED CT	94280003	Secondary malignant neoplasm of endocrine gland	Cancer
88877	SNOMED CT	94281004	Secondary malignant neoplasm of endometrium	Cancer
88878	SNOMED CT	94282006	Secondary malignant neoplasm of epicardium	Cancer
88879	SNOMED CT	94283001	Secondary malignant neoplasm of epididymis	Cancer
88880	SNOMED CT	94284007	Secondary malignant neoplasm of epiglottis	Cancer
88882	SNOMED CT	94286009	Secondary malignant neoplasm of esophagus	Cancer
88883	SNOMED CT	94287000	Secondary malignant neoplasm of ethmoid bone	Cancer
88884	SNOMED CT	94288005	Secondary malignant neoplasm of ethmoidal sinus	Cancer
88885	SNOMED CT	94289002	Secondary malignant neoplasm of eustachian tube	Cancer
88886	SNOMED CT	94290006	Secondary malignant neoplasm of exocervix	Cancer
88888	SNOMED CT	94292003	Secondary malignant neoplasm of eye	Cancer
88891	SNOMED CT	94295001	Secondary malignant neoplasm of fallopian tube	Cancer
88892	SNOMED CT	94296000	Secondary malignant neoplasm of false vocal cord	Cancer
88893	SNOMED CT	94297009	Secondary malignant neoplasm of female breast	Cancer
88894	SNOMED CT	94298004	Secondary malignant neoplasm of female genital organ	Cancer
88896	SNOMED CT	94300004	Secondary malignant neoplasm of femur	Cancer
88897	SNOMED CT	94301000	Secondary malignant neoplasm of fibula	Cancer
88898	SNOMED CT	94302007	Secondary malignant neoplasm of first cuneiform bone of foot	Cancer
88900	SNOMED CT	94304008	Secondary malignant neoplasm of floor of mouth	Cancer

88903	SNOMED CT	94307001	Secondary malignant neoplasm of prepuce	Cancer
88904	SNOMED CT	94308006	Secondary malignant neoplasm of frontal bone	Cancer
88905	SNOMED CT	94309003	Secondary malignant neoplasm of frontal lobe	Cancer
88906	SNOMED CT	94310008	Secondary malignant neoplasm of frontal sinus	Cancer
88907	SNOMED CT	94311007	Secondary malignant neoplasm of fundus of stomach	Cancer
88910	SNOMED CT	94314004	Secondary malignant neoplasm of gingival mucosa	Cancer
88911	SNOMED CT	94315003	Secondary malignant neoplasm of glans penis	Cancer
88914	SNOMED CT	94318001	Secondary malignant neoplasm of glottis	Cancer
88916	SNOMED CT	94320003	Secondary malignant neoplasm of greater curvature of stomach	Cancer
88917	SNOMED CT	94321004	Secondary malignant neoplasm of gum	Cancer
88918	SNOMED CT	94322006	Secondary malignant neoplasm of hamate bone	Cancer
88920	SNOMED CT	94324007	Secondary malignant neoplasm of hard palate	Cancer
88923	SNOMED CT	94327000	Secondary malignant neoplasm of heart	Cancer
88925	SNOMED CT	94329002	Secondary malignant neoplasm of hilus of lung	Cancer
88928	SNOMED CT	94332004	Secondary malignant neoplasm of hypopharyngeal aspect of aryepiglottic fold	Cancer
88929	SNOMED CT	94333009	Secondary malignant neoplasm of hypopharyngeal aspect of interarytenoid fold	Cancer
88930	SNOMED CT	94334003	Secondary malignant neoplasm of hypopharynx	Cancer
88933	SNOMED CT	94337005	Secondary malignant neoplasm of ilium	Cancer
88934	SNOMED CT	94338000	Secondary malignant neoplasm of infraclavicular lymph nodes	Cancer
88937	SNOMED CT	94341009	Secondary malignant neoplasm of inner aspect of lip	Cancer
88938	SNOMED CT	94342002	Secondary malignant neoplasm of inner aspect of lower lip	Cancer
88939	SNOMED CT	94343007	Secondary malignant neoplasm of inner aspect of upper lip	Cancer
88940	SNOMED CT	94344001	Secondary malignant neoplasm of intercostal lymph nodes	Cancer
88947	SNOMED CT	94351005	Secondary malignant neoplasm of intrathoracic lymph nodes	Cancer

88948	SNOMED CT	94352003	Secondary malignant neoplasm of intrathoracic organs	Cancer
88949	SNOMED CT	94353008	Secondary malignant neoplasm of ischium	Cancer
88950	SNOMED CT	94354002	Secondary malignant neoplasm of islets of Langerhans	Cancer
88951	SNOMED CT	94355001	Secondary malignant neoplasm of isthmus of uterus	Cancer
88954	SNOMED CT	94358004	Secondary malignant neoplasm of junctional region of epiglottis	Cancer
88955	SNOMED CT	94359007	Secondary malignant neoplasm of junctional zone of tongue	Cancer
88956	SNOMED CT	94360002	Secondary malignant neoplasm of kidney	Cancer
88957	SNOMED CT	94361003	Secondary malignant neoplasm of labia majora	Cancer
88958	SNOMED CT	94362005	Secondary malignant neoplasm of labia minora	Cancer
88960	SNOMED CT	94364006	Secondary malignant neoplasm of lacrimal gland	Cancer
88961	SNOMED CT	94366008	Secondary malignant neoplasm of laryngeal aspect of aryepiglottic fold	Cancer
88962	SNOMED CT	94367004	Secondary malignant neoplasm of laryngeal aspect of interarytenoid fold	Cancer
88963	SNOMED CT	94368009	Secondary malignant neoplasm of laryngeal commissure	Cancer
88964	SNOMED CT	94369001	Secondary malignant neoplasm of laryngeal surface of epiglottis	Cancer
88965	SNOMED CT	94370000	Secondary malignant neoplasm of larynx	Cancer
88966	SNOMED CT	94371001	Secondary malignant neoplasm of lateral portion of floor of mouth	Cancer
88967	SNOMED CT	94372008	Secondary malignant neoplasm of lateral wall of nasopharynx	Cancer
88968	SNOMED CT	94373003	Secondary malignant neoplasm of lateral wall of oropharynx	Cancer
88969	SNOMED CT	94374009	Secondary malignant neoplasm of lateral wall of urinary bladder	Cancer
88970	SNOMED CT	94375005	Secondary malignant neoplasm of left lower lobe of lung	Cancer
88971	SNOMED CT	94376006	Secondary malignant neoplasm of left upper lobe of lung	Cancer
88973	SNOMED CT	94378007	Secondary malignant neoplasm of lesser curvature of stomach	Cancer
88974	SNOMED CT	94379004	Secondary malignant neoplasm of lingual tonsil	Cancer

88975	SNOMED CT	94380001	Secondary malignant neoplasm of lip	Cancer
88977	SNOMED CT	94382009	Secondary malignant neoplasm of long bone of lower limb	Cancer
88978	SNOMED CT	94383004	Secondary malignant neoplasm of long bone of upper limb	Cancer
88979	SNOMED CT	94384005	Secondary malignant neoplasm of lower gum	Cancer
88980	SNOMED CT	94385006	Secondary malignant neoplasm of lower inner quadrant of female breast	Cancer
88982	SNOMED CT	94387003	Secondary malignant neoplasm of lower outer quadrant of female breast	Cancer
88983	SNOMED CT	94388008	Secondary malignant neoplasm of lower third of esophagus	Cancer
88984	SNOMED CT	94389000	Secondary malignant neoplasm of lumbar vertebral column	Cancer
88985	SNOMED CT	94390009	Secondary malignant neoplasm of lunate bone	Cancer
88986	SNOMED CT	94391008	Secondary malignant neoplasm of lung	Cancer
88988	SNOMED CT	94393006	Secondary malignant neoplasm of lymph nodes of face	Cancer
88989	SNOMED CT	94394000	Secondary malignant neoplasm of lymph nodes of head	Cancer
88992	SNOMED CT	94397007	Secondary malignant neoplasm of lymph nodes of neck	Cancer
88994	SNOMED CT	94399005	Secondary malignant neoplasm of main bronchus	Cancer
88995	SNOMED CT	94400003	Secondary malignant neoplasm of major salivary gland	Cancer
88996	SNOMED CT	94401004	Secondary malignant neoplasm of male breast	Cancer
88997	SNOMED CT	94402006	Secondary malignant neoplasm of male genital organ	Cancer
88998	SNOMED CT	94403001	Secondary malignant neoplasm of mandible	Cancer
88999	SNOMED CT	94404007	Secondary malignant neoplasm of mastoid air cells	Cancer
89000	SNOMED CT	94405008	Secondary malignant neoplasm of maxilla	Cancer
89001	SNOMED CT	94406009	Secondary malignant neoplasm of maxillary sinus	Cancer
89002	SNOMED CT	94408005	Secondary malignant neoplasm of mediastinal lymph nodes	Cancer
89003	SNOMED CT	94409002	Secondary malignant neoplasm of mediastinum	Cancer
89005	SNOMED CT	94411006	Secondary malignant neoplasm of metacarpal bone	Cancer

89006	SNOMED CT	94412004	Secondary malignant neoplasm of metatarsal bone	Cancer
89007	SNOMED CT	94413009	Secondary malignant neoplasm of middle ear	Cancer
89008	SNOMED CT	94414003	Secondary malignant neoplasm of middle third of esophagus	Cancer
89009	SNOMED CT	94415002	Secondary malignant neoplasm of minor salivary gland	Cancer
89011	SNOMED CT	94417005	Secondary malignant neoplasm of multiple endocrine glands	Cancer
89023	SNOMED CT	94429001	Secondary malignant neoplasm of muscle of thorax	Cancer
89027	SNOMED CT	94433008	Secondary malignant neoplasm of myocardium	Cancer
89028	SNOMED CT	94434002	Secondary malignant neoplasm of myometrium	Cancer
89029	SNOMED CT	94435001	Secondary malignant neoplasm of nasal bone	Cancer
89030	SNOMED CT	94436000	Secondary malignant neoplasm of nasal cavity	Cancer
89031	SNOMED CT	94437009	Secondary malignant neoplasm of nasal concha	Cancer
89033	SNOMED CT	94439007	Secondary malignant neoplasm of navicular bone of foot	Cancer
89034	SNOMED CT	94440009	Secondary malignant neoplasm of scaphoid bone	Cancer
89037	SNOMED CT	94443006	Secondary malignant neoplasm of nipple of female breast	Cancer
89038	SNOMED CT	94444000	Secondary malignant neoplasm of nipple of male breast	Cancer
89041	SNOMED CT	94447007	Secondary malignant neoplasm of occipital bone	Cancer
89042	SNOMED CT	94448002	Secondary malignant neoplasm of occipital lobe	Cancer
89043	SNOMED CT	94449005	Secondary malignant neoplasm of occipital lymph nodes	Cancer
89046	SNOMED CT	94452002	Secondary malignant neoplasm of optic nerve	Cancer
89047	SNOMED CT	94453007	Secondary malignant neoplasm of orbit	Cancer
89048	SNOMED CT	94454001	Secondary malignant neoplasm of oropharynx	Cancer
89049	SNOMED CT	94455000	Secondary malignant neoplasm of ovary	Cancer
89050	SNOMED CT	94456004	Secondary malignant neoplasm of palate	Cancer
89051	SNOMED CT	94457008	Secondary malignant neoplasm of palatine bone	Cancer
89052	SNOMED CT	94458003	Secondary malignant neoplasm of tonsil	Cancer

89057	SNOMED CT	94463004	Secondary malignant neoplasm of paramammary lymph nodes	Cancer
89058	SNOMED CT	94464005	Secondary malignant neoplasm of parametrial lymph nodes	Cancer
89059	SNOMED CT	94465006	Secondary malignant neoplasm of parametrium	Cancer
89061	SNOMED CT	94467003	Secondary malignant neoplasm of parathyroid gland	Cancer
89062	SNOMED CT	94468008	Secondary malignant neoplasm of paraurethral glands	Cancer
89064	SNOMED CT	94470004	Secondary malignant neoplasm of parietal bone	Cancer
89065	SNOMED CT	94471000	Secondary malignant neoplasm of parietal lobe	Cancer
89066	SNOMED CT	94472007	Secondary malignant neoplasm of parietal peritoneum	Cancer
89067	SNOMED CT	94473002	Secondary malignant neoplasm of parietal pleura	Cancer
89068	SNOMED CT	94474008	Secondary malignant neoplasm of parotid gland	Cancer
89069	SNOMED CT	94475009	Secondary malignant neoplasm of parotid lymph nodes	Cancer
89070	SNOMED CT	94476005	Secondary malignant neoplasm of patella	Cancer
89071	SNOMED CT	94477001	Secondary malignant neoplasm of pectoral axillary lymph nodes	Cancer
89072	SNOMED CT	94478006	Secondary malignant neoplasm of pelvic bone	Cancer
89073	SNOMED CT	94479003	Secondary malignant neoplasm of pelvic peritoneum	Cancer
89075	SNOMED CT	94481001	Secondary malignant neoplasm of penis	Cancer
89078	SNOMED CT	94484009	Secondary malignant neoplasm of pericardium	Cancer
89079	SNOMED CT	94485005	Secondary malignant neoplasm of perirenal tissue	Cancer
89080	SNOMED CT	94486006	Secondary malignant neoplasm of phalanx of foot	Cancer
89081	SNOMED CT	94487002	Secondary malignant neoplasm of phalanx of hand	Cancer
89082	SNOMED CT	94488007	Secondary malignant neoplasm of pharynx	Cancer
89083	SNOMED CT	94489004	Secondary malignant neoplasm of pineal gland	Cancer
89084	SNOMED CT	94490008	Secondary malignant neoplasm of pisiform bone of hand	Cancer
89085	SNOMED CT	94491007	Secondary malignant neoplasm of pituitary gland	Cancer
89087	SNOMED CT	94493005	Secondary malignant neoplasm of pleura	Cancer

89089	SNOMED CT	94495003	Secondary malignant neoplasm of postcricoid region	Cancer
89090	SNOMED CT	94496002	Secondary malignant neoplasm of posterior hypopharyngeal wall	Cancer
89091	SNOMED CT	94497006	Secondary malignant neoplasm of posterior mediastinum	Cancer
89092	SNOMED CT	94498001	Secondary malignant neoplasm of posterior wall of nasopharynx	Cancer
89093	SNOMED CT	94499009	Secondary malignant neoplasm of posterior wall of oropharynx	Cancer
89094	SNOMED CT	94500000	Secondary malignant neoplasm of posterior wall of urinary bladder	Cancer
89095	SNOMED CT	94501001	Secondary malignant neoplasm of preauricular lymph nodes	Cancer
89097	SNOMED CT	94503003	Secondary malignant neoplasm of prostate	Cancer
89098	SNOMED CT	94504009	Secondary malignant neoplasm of pubis	Cancer
89099	SNOMED CT	94505005	Secondary malignant neoplasm of pyloric antrum	Cancer
89100	SNOMED CT	94506006	Secondary malignant neoplasm of pylorus	Cancer
89101	SNOMED CT	94507002	Secondary malignant neoplasm of pyriform sinus	Cancer
89102	SNOMED CT	94508007	Secondary malignant neoplasm of radius	Cancer
89104	SNOMED CT	94510009	Secondary malignant neoplasm of rectouterine pouch	Cancer
89105	SNOMED CT	94511008	Secondary malignant neoplasm of rectovaginal septum	Cancer
89106	SNOMED CT	94512001	Secondary malignant neoplasm of rectovesical septum	Cancer
89107	SNOMED CT	94513006	Secondary malignant neoplasm of rectum	Cancer
89108	SNOMED CT	94514000	Secondary malignant neoplasm of renal pelvis	Cancer
89110	SNOMED CT	94516003	Secondary malignant neoplasm of retina	Cancer
89112	SNOMED CT	94518002	Secondary malignant neoplasm of retromolar area	Cancer
89114	SNOMED CT	94520004	Secondary malignant neoplasm of retropharyngeal lymph nodes	Cancer
89115	SNOMED CT	94521000	Secondary malignant neoplasm of rib	Cancer
89116	SNOMED CT	94522007	Secondary malignant neoplasm of right lower lobe of lung	Cancer
89117	SNOMED CT	94523002	Secondary malignant neoplasm of right middle lobe of lung	Cancer

89118	SNOMED CT	94524008	Secondary malignant neoplasm of right upper lobe of lung	Cancer
89119	SNOMED CT	94525009	Secondary malignant neoplasm of round ligament of uterus	Cancer
89121	SNOMED CT	94527001	Secondary malignant neoplasm of sacrum	Cancer
89122	SNOMED CT	94528006	Secondary malignant neoplasm of scalene lymph nodes	Cancer
89123	SNOMED CT	94529003	Secondary malignant neoplasm of scapula	Cancer
89124	SNOMED CT	94530008	Secondary malignant neoplasm of sclera	Cancer
89125	SNOMED CT	94531007	Secondary malignant neoplasm of scrotum	Cancer
89127	SNOMED CT	94533005	Secondary malignant neoplasm of second cuneiform bone of foot	Cancer
89128	SNOMED CT	94534004	Secondary malignant neoplasm of septum of nose	Cancer
89129	SNOMED CT	94535003	Secondary malignant neoplasm of short bone of lower limb	Cancer
89130	SNOMED CT	94536002	Secondary malignant neoplasm of short bone of upper limb	Cancer
89136	SNOMED CT	94542003	Secondary malignant neoplasm of skin of axilla	Cancer
89138	SNOMED CT	94544002	Secondary malignant neoplasm of skin of breast	Cancer
89140	SNOMED CT	94546000	Secondary malignant neoplasm of skin of cheek	Cancer
89141	SNOMED CT	94547009	Secondary malignant neoplasm of skin of chest	Cancer
89147	SNOMED CT	94553009	Secondary malignant neoplasm of skin of eyelid	Cancer
89158	SNOMED CT	94564007	Secondary malignant neoplasm of skin of lip	Cancer
89175	SNOMED CT	94581003	Secondary malignant neoplasm of soft palate	Cancer
89177	SNOMED CT	94583000	Secondary malignant neoplasm of soft tissues of axilla	Cancer
89188	SNOMED CT	94594001	Secondary malignant neoplasm of soft tissues of thorax	Cancer
89191	SNOMED CT	94597008	Secondary malignant neoplasm of spermatic cord	Cancer
89192	SNOMED CT	94598003	Secondary malignant neoplasm of sphenoid bone	Cancer
89193	SNOMED CT	94599006	Secondary malignant neoplasm of sphenoidal sinus	Cancer
89194	SNOMED CT	94600009	Secondary malignant neoplasm of spinal cord	Cancer

89195	SNOMED CT	94601008	Secondary malignant neoplasm of spinal meninges	Cancer
89196	SNOMED CT	94602001	Secondary malignant neoplasm of vertebral column	Cancer
89199	SNOMED CT	94605004	Secondary malignant neoplasm of sternum	Cancer
89200	SNOMED CT	94606003	Secondary malignant neoplasm of stomach	Cancer
89201	SNOMED CT	94607007	Secondary malignant neoplasm of subglottis	Cancer
89202	SNOMED CT	94608002	Secondary malignant neoplasm of sublingual gland	Cancer
89203	SNOMED CT	94609005	Secondary malignant neoplasm of submandibular lymph nodes	Cancer
89205	SNOMED CT	94611001	Secondary malignant neoplasm of submental lymph nodes	Cancer
89207	SNOMED CT	94613003	Secondary malignant neoplasm of superior wall of nasopharynx	Cancer
89208	SNOMED CT	94614009	Secondary malignant neoplasm of supraclavicular lymph nodes	Cancer
89210	SNOMED CT	94616006	Secondary malignant neoplasm of supraglottis	Cancer
89213	SNOMED CT	94619004	Secondary malignant neoplasm of talus	Cancer
89214	SNOMED CT	94620005	Secondary malignant neoplasm of tarsal bone	Cancer
89215	SNOMED CT	94621009	Secondary malignant neoplasm of temporal bone	Cancer
89216	SNOMED CT	94622002	Secondary malignant neoplasm of temporal lobe	Cancer
89217	SNOMED CT	94623007	Secondary malignant neoplasm of testis	Cancer
89218	SNOMED CT	94624001	Secondary malignant neoplasm of the mesentery	Cancer
89219	SNOMED CT	94625000	Secondary malignant neoplasm of the mesocolon	Cancer
89220	SNOMED CT	94626004	Secondary malignant neoplasm of omentum	Cancer
89221	SNOMED CT	94627008	Secondary malignant neoplasm of peritoneum	Cancer
89224	SNOMED CT	94630001	Secondary malignant neoplasm of third cuneiform bone of foot	Cancer
89225	SNOMED CT	94631002	Secondary malignant neoplasm of thoracic esophagus	Cancer
89226	SNOMED CT	94632009	Secondary malignant neoplasm of thymus	Cancer
89228	SNOMED CT	94634005	Secondary malignant neoplasm of thyroid gland	Cancer
89229	SNOMED CT	94635006	Secondary malignant neoplasm of tibia	Cancer

89231	SNOMED CT	94637003	Secondary malignant neoplasm of tip and lateral border of tongue	Cancer
89232	SNOMED CT	94638008	Secondary malignant neoplasm of tongue	Cancer
89233	SNOMED CT	94639000	Secondary malignant neoplasm of tonsillar fossa	Cancer
89234	SNOMED CT	94640003	Secondary malignant neoplasm of tonsillar pillar	Cancer
89235	SNOMED CT	94641004	Secondary malignant neoplasm of trachea	Cancer
89236	SNOMED CT	94642006	Secondary malignant neoplasm of tracheobronchial lymph nodes	Cancer
89238	SNOMED CT	94644007	Secondary malignant neoplasm of trapezium	Cancer
89239	SNOMED CT	94645008	Secondary malignant neoplasm of trapezoid bone	Cancer
89241	SNOMED CT	94647000	Secondary malignant neoplasm of trigone of urinary bladder	Cancer
89244	SNOMED CT	94650002	Secondary malignant neoplasm of ulna	Cancer
89245	SNOMED CT	94651003	Secondary malignant neoplasm of undescended testis	Cancer
89246	SNOMED CT	94652005	Secondary malignant neoplasm of upper gum	Cancer
89247	SNOMED CT	94653000	Secondary malignant neoplasm of upper inner quadrant of female breast	Cancer
89249	SNOMED CT	94655007	Secondary malignant neoplasm of upper outer quadrant of female breast	Cancer
89250	SNOMED CT	94656008	Secondary malignant neoplasm of upper respiratory tract	Cancer
89251	SNOMED CT	94657004	Secondary malignant neoplasm of upper third of esophagus	Cancer
89252	SNOMED CT	94658009	Secondary malignant neoplasm of urachus	Cancer
89253	SNOMED CT	94659001	Secondary malignant neoplasm of ureter	Cancer
89254	SNOMED CT	94660006	Secondary malignant neoplasm of ureteric orifice of urinary bladder	Cancer
89255	SNOMED CT	94661005	Secondary malignant neoplasm of urethra	Cancer
89256	SNOMED CT	94662003	Secondary malignant neoplasm of urinary bladder neck	Cancer
89257	SNOMED CT	94663008	Secondary malignant neoplasm of urinary system	Cancer
89259	SNOMED CT	94665001	Secondary malignant neoplasm of uterus	Cancer

89260	SNOMED CT	94666000	Secondary malignant neoplasm of uveal tract	Cancer
89261	SNOMED CT	94667009	Secondary malignant neoplasm of uvula	Cancer
89262	SNOMED CT	94668004	Secondary malignant neoplasm of vagina	Cancer
89264	SNOMED CT	94670008	Secondary malignant neoplasm of vallecula	Cancer
89265	SNOMED CT	94671007	Secondary malignant neoplasm of vas deferens	Cancer
89266	SNOMED CT	94672000	Secondary malignant neoplasm of ventral surface of tongue	Cancer
89267	SNOMED CT	94673005	Secondary malignant neoplasm of vermilion border of lip	Cancer
89268	SNOMED CT	94674004	Secondary malignant neoplasm of vermilion border of lower lip	Cancer
89269	SNOMED CT	94675003	Secondary malignant neoplasm of vermilion border of upper lip	Cancer
89270	SNOMED CT	94676002	Secondary malignant neoplasm of vestibule of mouth	Cancer
89271	SNOMED CT	94677006	Secondary malignant neoplasm of vestibule of nose	Cancer
89272	SNOMED CT	94678001	Secondary malignant neoplasm of visceral pleura	Cancer
89273	SNOMED CT	94679009	Secondary malignant neoplasm of vocal cord	Cancer
89274	SNOMED CT	94680007	Secondary malignant neoplasm of vomer	Cancer
89276	SNOMED CT	94683009	Secondary malignant neoplasm of zygomatic bone	Cancer
89297	SNOMED CT	94704006	Multiple myeloma in remission	Cancer
89305	SNOMED CT	94712003	Mycosis fungoides of lymph nodes of inguinal region AND/OR lower limb	Cancer
89350	SNOMED CT	94758002	Neoplasm of uncertain behavior of body of stomach	Cancer
89369	SNOMED CT	94777000	Neoplasm of uncertain behavior of cardia of stomach	Cancer
89400	SNOMED CT	94808000	Neoplasm of uncertain behavior of craniopharyngeal duct	Cancer
89441	SNOMED CT	94849000	Neoplasm of uncertain behavior of fundus of stomach	Cancer
89450	SNOMED CT	94858007	Neoplasm of uncertain behavior of greater curvature of stomach	Cancer
89485	SNOMED CT	94893000	Neoplasm of uncertain behavior of lacrimal gland	Cancer
89499	SNOMED CT	94907009	Neoplasm of uncertain behavior of lesser curvature of stomach	Cancer

89595	SNOMED CT	95004009	Neoplasm of uncertain behavior of pituitary gland	Cancer
89607	SNOMED CT	95016003	Neoplasm of uncertain behavior of pyloric antrum	Cancer
89608	SNOMED CT	95017007	Neoplasm of uncertain behavior of pylorus	Cancer
89851	SNOMED CT	95261008	S√©zary's disease of lymph nodes of inguinal region AND/OR lower limb	Cancer
99111	SNOMED CT	109347009	Overlapping malignant neoplasm of bone and articular cartilage	Cancer
99575	SNOMED CT	109833003	Overlapping malignant neoplasm of lip, oral cavity and pharynx	Cancer
99622	SNOMED CT	109885001	Overlapping malignant neoplasm of vulva	Cancer
99624	SNOMED CT	109887009	Overlapping malignant neoplasm of male breast	Cancer
99696	SNOMED CT	109982002	Alpha heavy chain disease (clinical)	Cancer
99697	SNOMED CT	109984001	Gamma heavy chain disease (clinical)	Cancer
99698	SNOMED CT	109985000	Immunoproliferative small intestinal disease (clinical)	Cancer
99701	SNOMED CT	109989006	Multiple myeloma	Cancer
99702	SNOMED CT	109991003	Acute panmyelosis with myelofibrosis	Cancer
110688	SNOMED CT	123313007	Alpha heavy chain disease, enteric form	Cancer
111006	SNOMED CT	123845008	Adenocarcinoma of endometrium	Cancer
113453	SNOMED CT	127004000	Neoplasm of lacrimal gland	Cancer
113471	SNOMED CT	127024001	Neoplasm of pituitary gland	Cancer
113472	SNOMED CT	127025000	Neoplasm of craniopharyngeal duct	Cancer
113663	SNOMED CT	127218004	Reactive follicular hyperplasia in the elderly	Cancer
2648376	SNOMED CT	1.162E+09	Rhabdomyosarcoma of isthmus uteri	Cancer
2648377	SNOMED CT	1.162E+09	Rhabdomyosarcoma of myometrium of corpus uteri	Cancer
2931386	SNOMED CT	1.217E+09	Precursor cell lymphoblastic lymphoma	Cancer
388985	SNOMED CT	699317002	Paratesticular malignant neoplasm	Cancer
388986	SNOMED CT	699318007	Supratentorial primitive neuroectodermal tumor	Cancer
388999	SNOMED CT	699331002	Granular cell tumor of neurohypophysis	Cancer

389021	SNOMED CT	699354006	Sarcoma of orbit	Cancer
389022	SNOMED CT	699355007	Leiomyosarcoma of orbit	Cancer
389023	SNOMED CT	699356008	Endometrial stromal sarcoma	Cancer
389024	SNOMED CT	699357004	Low grade endometrial stromal sarcoma	Cancer
389025	SNOMED CT	699358009	High grade endometrial stromal sarcoma	Cancer
389358	SNOMED CT	699704002	Classic medulloblastoma	Cancer
389702	SNOMED CT	700057001	Emberger syndrome	Cancer
390090	SNOMED CT	700488005	Malignant sex cord tumor of testis	Cancer
391814	SNOMED CT	702346005	Potocki-Shaffer syndrome	Cancer
391835	SNOMED CT	702368000	Carcinosarcoma of ovary	Cancer
391836	SNOMED CT	702369008	Carcinosarcoma of uterus	Cancer
391842	SNOMED CT	702375004	Familial isolated pituitary adenoma	Cancer
391858	SNOMED CT	702391001	Renal cell carcinoma	Cancer
391872	SNOMED CT	702405001	Malignant granulosa cell tumor of testis	Cancer
391932	SNOMED CT	702467006	Malignant neoplasm of augmented bladder	Cancer
392671	SNOMED CT	703228009	Non-small cell lung cancer with mutation in epidermal growth factor receptor	Cancer
392673	SNOMED CT	703230006	Non-small cell lung cancer without mutation in epidermal growth factor receptor	Cancer
392868	SNOMED CT	703429003	Malignant optic glioma of adulthood	Cancer
395802	SNOMED CT	706970001	Triple-negative breast cancer	Cancer
396162	SNOMED CT	707337006	Primary adenocarcinoma of accessory sinus	Cancer
396164	SNOMED CT	707339009	Primary adenocarcinoma of maxillary sinus	Cancer
396167	SNOMED CT	707342003	Primary adenocarcinoma of ethmoidal sinus	Cancer
396168	SNOMED CT	707343008	Primary adenocarcinoma of frontal sinus	Cancer
396169	SNOMED CT	707344002	Primary adenocarcinoma of sphenoidal sinus	Cancer
396170	SNOMED CT	707345001	Primary carcinoma of accessory sinus	Cancer

396171	SNOMED CT	707346000	Primary carcinoma of ethmoidal sinus	Cancer
396172	SNOMED CT	707347009	Primary carcinoma of maxillary sinus	Cancer
396173	SNOMED CT	707348004	Primary carcinoma of sphenoidal sinus	Cancer
396174	SNOMED CT	707349007	Primary carcinoma of frontal sinus	Cancer
396175	SNOMED CT	707350007	Malignant melanoma of accessory sinus	Cancer
396178	SNOMED CT	707353009	Primary squamous cell carcinoma of accessory sinus	Cancer
396179	SNOMED CT	707354003	Primary squamous cell carcinoma of maxillary sinus	Cancer
396180	SNOMED CT	707355002	Primary squamous cell carcinoma of sphenoidal sinus	Cancer
396181	SNOMED CT	707356001	Primary squamous cell carcinoma of frontal sinus	Cancer
396182	SNOMED CT	707357005	Primary squamous cell carcinoma of laryngeal cartilage	Cancer
396183	SNOMED CT	707358000	Primary squamous cell carcinoma of larynx	Cancer
396184	SNOMED CT	707359008	Primary squamous cell carcinoma of ethmoidal sinus	Cancer
396185	SNOMED CT	707360003	Primary lymphoepithelial carcinoma of larynx	Cancer
396186	SNOMED CT	707361004	Malignant melanoma of frontal sinus	Cancer
396187	SNOMED CT	707362006	Malignant melanoma of sphenoidal sinus	Cancer
396202	SNOMED CT	707377003	Primary signet ring cell carcinoma of trachea	Cancer
396203	SNOMED CT	707378008	Primary myoepithelial carcinoma of trachea	Cancer
396204	SNOMED CT	707379000	Primary mucoepidermoid carcinoma of trachea	Cancer
396205	SNOMED CT	707380002	Primary salivary gland type carcinoma of trachea	Cancer
396206	SNOMED CT	707381003	Solitary hamartoma of lung	Cancer
396207	SNOMED CT	707382005	Multiple hamartoma of lung	Cancer
396208	SNOMED CT	707383000	Primary mucinous cystadenocarcinoma of trachea	Cancer
396209	SNOMED CT	707384006	Primary solid carcinoma of trachea	Cancer
396210	SNOMED CT	707385007	Primary undifferentiated carcinoma of trachea	Cancer
396211	SNOMED CT	707386008	Primary acinar cell carcinoma of trachea	Cancer

396213	SNOMED CT	707388009	Primary squamous cell carcinoma of trachea	Cancer
396214	SNOMED CT	707389001	Primary clear cell squamous cell carcinoma of trachea	Cancer
396215	SNOMED CT	707390005	Primary basaloid squamous cell carcinoma of trachea	Cancer
396216	SNOMED CT	707391009	Primary papillary squamous cell carcinoma of trachea	Cancer
396217	SNOMED CT	707392002	Primary giant cell carcinoma of trachea	Cancer
396218	SNOMED CT	707393007	Primary adenosquamous carcinoma of trachea	Cancer
396219	SNOMED CT	707394001	Primary spindle cell carcinoma of trachea	Cancer
396220	SNOMED CT	707395000	Primary adenocarcinoma of hypopharynx	Cancer
396221	SNOMED CT	707396004	Primary oxyphilic adenocarcinoma of oropharynx	Cancer
396222	SNOMED CT	707397008	Primary basal cell adenocarcinoma of oropharynx	Cancer
396223	SNOMED CT	707398003	Primary polymorphous low grade adenocarcinoma of oropharynx	Cancer
396224	SNOMED CT	707399006	Primary papillary adenocarcinoma of oropharynx	Cancer
396225	SNOMED CT	707400004	Primary mucinous adenocarcinoma of oropharynx	Cancer
396226	SNOMED CT	707401000	Primary clear cell adenocarcinoma of oropharynx	Cancer
396227	SNOMED CT	707402007	Primary adenocarcinoma of oropharynx	Cancer
396228	SNOMED CT	707403002	Primary fetal adenocarcinoma of lung	Cancer
396229	SNOMED CT	707404008	Primary mixed subtype adenocarcinoma of lung	Cancer
396230	SNOMED CT	707405009	Primary adenosquamous carcinoma of lung	Cancer
396231	SNOMED CT	707406005	Primary mucoepidermoid carcinoma of hypopharynx	Cancer
396232	SNOMED CT	707407001	Primary signet ring cell carcinoma of lung	Cancer
396233	SNOMED CT	707408006	Primary small cell non-keratinizing squamous cell carcinoma of lung	Cancer
396234	SNOMED CT	707409003	Primary acinar cell carcinoma of lung	Cancer
396235	SNOMED CT	707410008	Primary solid carcinoma of lung	Cancer
396236	SNOMED CT	707411007	Primary papillary adenocarcinoma of lung	Cancer

396246	SNOMED CT	707421004	Primary undifferentiated carcinoma of larynx	Cancer
396247	SNOMED CT	707422006	Primary spindle cell squamous cell carcinoma of larynx	Cancer
396248	SNOMED CT	707423001	Primary basaloid carcinoma of larynx	Cancer
396249	SNOMED CT	707424007	Primary adenosquamous cell carcinoma of larynx	Cancer
396250	SNOMED CT	707425008	Primary adenoid squamous cell carcinoma of larynx	Cancer
396251	SNOMED CT	707426009	Primary papillary squamous cell carcinoma of larynx	Cancer
396252	SNOMED CT	707427000	Primary verrucous carcinoma of larynx	Cancer
396253	SNOMED CT	707429002	Overlapping squamous cell carcinoma of larynx	Cancer
396254	SNOMED CT	707430007	Overlapping squamous cell carcinoma of laryngeal cartilage	Cancer
396273	SNOMED CT	707451005	Primary adenocarcinoma of lung	Cancer
396274	SNOMED CT	707452003	Primary mucinous adenocarcinoma of lung	Cancer
396275	SNOMED CT	707453008	Primary clear cell squamous cell carcinoma of lung	Cancer
396276	SNOMED CT	707454002	Primary basaloid squamous cell carcinoma of lung	Cancer
396277	SNOMED CT	707455001	Primary papillary squamous cell carcinoma of lung	Cancer
396278	SNOMED CT	707456000	Primary undifferentiated carcinoma of lung	Cancer
396279	SNOMED CT	707457009	Primary spindle cell carcinoma of lung	Cancer
396280	SNOMED CT	707458004	Primary pleomorphic carcinoma of lung	Cancer
396281	SNOMED CT	707460002	Primary pseudosarcomatous carcinoma of lung	Cancer
396285	SNOMED CT	707464006	Primary myoepithelial carcinoma of lung	Cancer
396286	SNOMED CT	707465007	Primary mucoepidermoid carcinoma of lung	Cancer
396287	SNOMED CT	707466008	Primary adenoid cystic carcinoma of lung	Cancer
396288	SNOMED CT	707467004	Primary salivary gland type carcinoma of lung	Cancer
396289	SNOMED CT	707468009	Primary mixed mucinous and non-mucinous bronchiolo-alveolar carcinoma of lung	Cancer
396290	SNOMED CT	707469001	Primary non-mucinous bronchiolo-alveolar carcinoma of lung	Cancer

396291	SNOMED CT	707470000	Primary mucinous bronchiolo-alveolar carcinoma of lung	Cancer
396292	SNOMED CT	707471001	Primary clear cell adenocarcinoma of trachea	Cancer
396293	SNOMED CT	707472008	Primary papillary adenocarcinoma of trachea	Cancer
396294	SNOMED CT	707473003	Primary mucinous adenocarcinoma of trachea	Cancer
396296	SNOMED CT	707475005	Primary adenocarcinoma of trachea	Cancer
396300	SNOMED CT	707479004	Primary adenocarcinoma of subglottis	Cancer
396302	SNOMED CT	707481002	Primary basaloid squamous cell carcinoma of hypopharynx	Cancer
396303	SNOMED CT	707482009	Primary papillary squamous cell carcinoma of hypopharynx	Cancer
396304	SNOMED CT	707483004	Primary undifferentiated carcinoma of hypopharynx	Cancer
396305	SNOMED CT	707484005	Primary adenoid squamous cell carcinoma of hypopharynx	Cancer
396306	SNOMED CT	707485006	Primary adenosquamous carcinoma of hypopharynx	Cancer
396307	SNOMED CT	707486007	Primary basaloid carcinoma of hypopharynx	Cancer
396308	SNOMED CT	707487003	Primary giant cell carcinoma of hypopharynx	Cancer
396309	SNOMED CT	707489000	Primary spindle cell squamous cell carcinoma of hypopharynx	Cancer
396310	SNOMED CT	707490009	Primary verrucous carcinoma of hypopharynx	Cancer
396311	SNOMED CT	707491008	Primary lymphoepithelial carcinoma of hypopharynx	Cancer
396312	SNOMED CT	707492001	Primary squamous cell carcinoma of hypopharynx	Cancer
396313	SNOMED CT	707493006	Primary lymphoepithelial carcinoma of trachea	Cancer
396314	SNOMED CT	707494000	Primary verrucous carcinoma of trachea	Cancer
396315	SNOMED CT	707495004	Primary squamous cell adenoid carcinoma of trachea	Cancer
396342	SNOMED CT	707528007	Primary squamous cell carcinoma of nasopharynx	Cancer
396343	SNOMED CT	707529004	Overlapping squamous cell carcinoma of oropharynx	Cancer
396344	SNOMED CT	707530009	Cystic hamartoma of lung and kidney	Cancer
396346	SNOMED CT	707532001	Primary squamous cell carcinoma of posterior wall of oropharynx	Cancer

396348	SNOMED CT	707535004	Primary squamous cell carcinoma of lateral wall of oropharynx	Cancer
396350	SNOMED CT	707537007	Primary squamous cell carcinoma of anterior surface of epiglottis	Cancer
396351	SNOMED CT	707538002	Primary squamous cell carcinoma of vallecula	Cancer
396352	SNOMED CT	707539005	Primary adenoid cystic carcinoma of hypopharynx	Cancer
396387	SNOMED CT	707575007	Primary squamous cell carcinoma of supraglottis	Cancer
396388	SNOMED CT	707576008	Primary squamous cell carcinoma of subglottis	Cancer
396391	SNOMED CT	707579001	Primary basaloid squamous cell carcinoma of oropharynx	Cancer
396392	SNOMED CT	707580003	Primary basaloid carcinoma of oropharynx	Cancer
396393	SNOMED CT	707581004	Primary papillary squamous cell carcinoma of oropharynx	Cancer
396394	SNOMED CT	707582006	Primary spindle cell squamous cell carcinoma of oropharynx	Cancer
396395	SNOMED CT	707583001	Primary adenosquamous carcinoma of oropharynx	Cancer
396396	SNOMED CT	707584007	Primary lymphoepithelial carcinoma of oropharynx	Cancer
396397	SNOMED CT	707585008	Primary squamous cell carcinoma of oropharynx	Cancer
396398	SNOMED CT	707586009	Primary myoepithelial carcinoma of oropharynx	Cancer
396399	SNOMED CT	707587000	Primary carcinoma ex pleomorphic adenoma of oropharynx	Cancer
396400	SNOMED CT	707588005	Primary epithelial-myoepithelial carcinoma of oropharynx	Cancer
396401	SNOMED CT	707589002	Primary cystadenocarcinoma of oropharynx	Cancer
396402	SNOMED CT	707590006	Primary acinar cell carcinoma of oropharynx	Cancer
396403	SNOMED CT	707591005	Primary mucoepidermoid carcinoma of oropharynx	Cancer
396404	SNOMED CT	707592003	Primary infiltrating duct carcinoma of oropharynx	Cancer
396405	SNOMED CT	707593008	Primary salivary gland-type tumor of oropharynx	Cancer
396407	SNOMED CT	707595001	Primary mucinous cystadenocarcinoma of lung	Cancer
396408	SNOMED CT	707596000	Primary carcinosarcoma of lung	Cancer

396438	SNOMED CT	707627009	Primary salivary gland type carcinoma of hypopharynx	Cancer
396439	SNOMED CT	707628004	Overlapping squamous cell carcinoma of hypopharynx	Cancer
396471	SNOMED CT	707660007	Primary giant cell carcinoma of larynx	Cancer
396473	SNOMED CT	707662004	Primary basaloid squamous cell carcinoma of larynx	Cancer
396475	SNOMED CT	707664003	Primary squamous cell carcinoma of glottis	Cancer
396479	SNOMED CT	707670009	Pleuropulmonary blastoma	Cancer
396480	SNOMED CT	707671008	Pleuropulmonary blastoma type I	Cancer
396481	SNOMED CT	707672001	Pleuropulmonary blastoma type II	Cancer
396482	SNOMED CT	707673006	Pleuropulmonary blastoma type III	Cancer
396483	SNOMED CT	707674000	Primary malignant epithelial neoplasm of trachea	Cancer
396495	SNOMED CT	707686002	Primary squamous cell carcinoma of posterior wall of hypopharynx	Cancer
396506	SNOMED CT	707697002	Primary squamous cell carcinoma of hypopharyngeal aspect of aryepiglottic fold	Cancer
396512	SNOMED CT	707703001	Primary squamous cell carcinoma of postcricoid region	Cancer
396513	SNOMED CT	707704007	Primary squamous cell carcinoma of pyriform sinus	Cancer
396514	SNOMED CT	707705008	Nonkeratinizing carcinoma of the nasopharynx	Cancer
397277	SNOMED CT	708504008	Periosteal osteosarcoma of jaw	Cancer
397683	SNOMED CT	708921005	Carcinoma of central portion of breast	Cancer
397730	SNOMED CT	708971008	Diffuse sclerosing papillary thyroid carcinoma	Cancer
397783	SNOMED CT	709031009	Malignant neoplasm of superior wall of nasopharynx	Cancer
398534	SNOMED CT	709830006	Malignant carcinoid tumor of stomach	Cancer
398847	SNOMED CT	710193006	Benign odontogenic tumor of lower jaw	Cancer
398848	SNOMED CT	710194000	Benign odontogenic tumor of upper jaw	Cancer
398849	SNOMED CT	710195004	Malignant odontogenic neoplasm of lower jaw	Cancer
398850	SNOMED CT	710196003	Malignant odontogenic tumor of upper jaw	Cancer

400043	SNOMED CT	711414003	Primary clear cell adenocarcinoma of lung	Cancer
400329	SNOMED CT	712525007	Malignant neoplasm of short bone of lower limb	Cancer
400544	SNOMED CT	712750007	Malignant neoplasm of chest wall	Cancer
400636	SNOMED CT	712849003	Prostate cancer metastatic to bone	Cancer
400817	SNOMED CT	713038003	Overlapping primary malignant neoplasm of bone and articular cartilage of upper limb	Cancer
401048	SNOMED CT	713290004	Malignant ameloblastoma of mandible	Cancer
401050	SNOMED CT	713293002	Malignant germ cell neoplasm of mediastinum	Cancer
401081	SNOMED CT	713327005	Malignant meningioma of meninges of brain	Cancer
401316	SNOMED CT	713573006	Malignant carcinoid tumor of rectum	Cancer
401317	SNOMED CT	713574000	Malignant carcinoid tumor of kidney	Cancer
401351	SNOMED CT	713609000	Invasive carcinoma of breast	Cancer
401386	SNOMED CT	713646001	Malignant germ cell tumor of testis	Cancer
402862	SNOMED CT	715215007	WAGR syndrome	Cancer
403024	SNOMED CT	715412008	Familial prostate cancer	Cancer
403203	SNOMED CT	715634002	Florid cemento-osseous dysplasia	Cancer
403444	SNOMED CT	715904005	Pineal parenchymal tumor of intermediate differentiation	Cancer
403446	SNOMED CT	715907003	Multiple endocrine neoplasia type 4	Cancer
404030	SNOMED CT	716586009	Epstein-Barr virus associated gastric carcinoma	Cancer
404032	SNOMED CT	716588005	Primary non-gestational choriocarcinoma of ovary	Cancer
404037	SNOMED CT	716593008	Carcinoma of salivary gland type of breast	Cancer
404088	SNOMED CT	716649003	Extraovarian primary peritoneal carcinoma	Cancer
404092	SNOMED CT	716653001	Neuroendocrine carcinoma of thymus	Cancer
404094	SNOMED CT	716655008	Aggressive systemic mastocytosis	Cancer
404095	SNOMED CT	716657000	Familial papillary thyroid carcinoma with renal papillary neoplasia syndrome	Cancer

404163	SNOMED CT	716742001	Multiple osteochondroma	Cancer
404260	SNOMED CT	716855006	Theca steroid producing cell malignant neoplasm of ovary	Cancer
404262	SNOMED CT	716859000	Hereditary diffuse carcinoma of stomach	Cancer
404397	SNOMED CT	717003001	Hereditary cavernous hemangioma of brain	Cancer
404826	SNOMED CT	717734005	Papillary thyroid carcinoma with renal papillary neoplasia	Cancer
404827	SNOMED CT	717735006	Renal cell carcinoma of kidney except renal pelvis	Cancer
404828	SNOMED CT	717736007	Familial renal cell carcinoma	Cancer
404993	SNOMED CT	717916003	Neuroendocrine carcinoma of appendix	Cancer
404998	SNOMED CT	717921000	Poorly-differentiated neuroendocrine carcinoma of thymus	Cancer
404999	SNOMED CT	717922007	Well-differentiated neuroendocrine carcinoma of thymus	Cancer
405044	SNOMED CT	717968005	Melanoma and neural system tumor syndrome	Cancer
405276	SNOMED CT	718220008	Hereditary breast and ovarian cancer syndrome	Cancer
405598	SNOMED CT	718604008	Small cell neuroendocrine carcinoma of bladder	Cancer
407757	SNOMED CT	721188000	Multiple endocrine neoplasia type 2A	Cancer
407870	SNOMED CT	721311006	Systemic Epstein-Barr virus positive T-cell lymphoproliferative disease of childhood	Cancer
408103	SNOMED CT	721546004	Primary adenocarcinoma of ciliary epithelium	Cancer
408104	SNOMED CT	721547008	Primary adenocarcinoma of epithelium of iris	Cancer
408105	SNOMED CT	721548003	Primary malignant neoplasm of lacrimal apparatus	Cancer
408106	SNOMED CT	721549006	Primary adenocarcinoma of lacrimal apparatus	Cancer
408113	SNOMED CT	721556000	Primary adenocarcinoma of palate	Cancer
408114	SNOMED CT	721557009	Primary adenocarcinoma of parotid gland	Cancer
408117	SNOMED CT	721560002	Primary adenocarcinoma of nasal cavity	Cancer
408118	SNOMED CT	721561003	Primary adenocarcinoma of middle ear	Cancer

408119	SNOMED CT	721562005	Primary adenocarcinoma of overlapping sites of retroperitoneum, peritoneum and omentum	Cancer
408120	SNOMED CT	721563000	Primary malignant melanoma of vagina	Cancer
408122	SNOMED CT	721568009	Primary adenocarcinoma of parametrium	Cancer
408123	SNOMED CT	721569001	Primary adenocarcinoma of uterine ligament	Cancer
408125	SNOMED CT	721571001	Benign endometrial stromal neoplasm	Cancer
408128	SNOMED CT	721574009	Primary rhabdomyosarcoma of male genital organ	Cancer
408129	SNOMED CT	721575005	Primary angiosarcoma of heart	Cancer
408130	SNOMED CT	721576006	Primary angiosarcoma of breast	Cancer
408132	SNOMED CT	721578007	Primary liposarcoma of male genital organ	Cancer
408134	SNOMED CT	721580001	Primary myosarcoma of omentum	Cancer
408156	SNOMED CT	721602000	Primary embryonal carcinoma of testis	Cancer
408157	SNOMED CT	721603005	Primary choriocarcinoma of testis	Cancer
408158	SNOMED CT	721604004	Primary squamous cell carcinoma of overlapping sites of male genital organ	Cancer
408159	SNOMED CT	721605003	Primary squamous cell carcinoma of overlapping sites of accessory sinuses	Cancer
408160	SNOMED CT	721606002	Primary adenocarcinoma of overlapping sites of accessory sinuses	Cancer
408161	SNOMED CT	721607006	Primary undifferentiated carcinoma of oropharynx	Cancer
408170	SNOMED CT	721617001	Primary adenocarcinoma of lower third of esophagus due to Barrett esophagus	Cancer
408171	SNOMED CT	721618006	Primary squamous cell carcinoma of upper third of esophagus	Cancer
408172	SNOMED CT	721619003	Primary squamous cell carcinoma of middle third of esophagus	Cancer
408173	SNOMED CT	721620009	Primary squamous cell carcinoma of lower third of esophagus	Cancer
408174	SNOMED CT	721621008	Primary squamous cell carcinoma of overlapping sites of esophagus	Cancer

408175	SNOMED CT	721622001	Primary adenocarcinoma of upper third of esophagus	Cancer
408176	SNOMED CT	721623006	Primary adenocarcinoma of middle third of esophagus	Cancer
408177	SNOMED CT	721624000	Primary adenocarcinoma of overlapping sites of esophagus	Cancer
408178	SNOMED CT	721625004	Primary neuroendocrine carcinoma of esophagus	Cancer
408179	SNOMED CT	721626003	Primary malignant neuroendocrine neoplasm of esophagus	Cancer
408180	SNOMED CT	721627007	Malignant melanoma of esophagus	Cancer
408181	SNOMED CT	721628002	Primary adenocarcinoma of esophagogastric junction	Cancer
408182	SNOMED CT	721629005	Linitis plastica of stomach	Cancer
408183	SNOMED CT	721630000	Primary adenocarcinoma of cardia of stomach	Cancer
408185	SNOMED CT	721632008	Primary adenocarcinoma of pyloric antrum of stomach	Cancer
408186	SNOMED CT	721633003	Primary adenocarcinoma of overlapping sites of stomach	Cancer
408187	SNOMED CT	721634009	Primary malignant neuroendocrine neoplasm of stomach	Cancer
408188	SNOMED CT	721635005	Primary malignant neuroendocrine neoplasm of cardia of stomach	Cancer
408189	SNOMED CT	721636006	Primary malignant neuroendocrine neoplasm of body of stomach	Cancer
408190	SNOMED CT	721637002	Primary malignant neuroendocrine neoplasm of pyloric antrum of stomach	Cancer
408191	SNOMED CT	721638007	Primary neuroendocrine carcinoma of stomach	Cancer
408192	SNOMED CT	721639004	Primary neuroendocrine carcinoma of cardia of stomach	Cancer
408193	SNOMED CT	721640002	Primary neuroendocrine carcinoma of body of stomach	Cancer
408194	SNOMED CT	721641003	Primary neuroendocrine carcinoma of pyloric antrum of stomach	Cancer
408195	SNOMED CT	721642005	Primary neuroendocrine carcinoma of overlapping sites of stomach	Cancer
408196	SNOMED CT	721643000	Primary malignant mesenchymal neoplasm of stomach	Cancer

408197	SNOMED CT	721644006	Primary malignant neuroendocrine neoplasm of duodenum	Cancer
408198	SNOMED CT	721645007	Primary neuroendocrine carcinoma of duodenum	Cancer
408225	SNOMED CT	721672004	Primary mucinous adenocarcinoma of appendix	Cancer
408226	SNOMED CT	721673009	Primary malignant neuroendocrine neoplasm of appendix	Cancer
408250	SNOMED CT	721697000	Primary neuroendocrine carcinoma of colon	Cancer
408251	SNOMED CT	721698005	Primary malignant neuroendocrine neoplasm of colon	Cancer
408253	SNOMED CT	721700001	Primary malignant neuroendocrine neoplasm of rectum	Cancer
408254	SNOMED CT	721701002	Primary neuroendocrine carcinoma of rectum	Cancer
408262	SNOMED CT	721709000	Primary cloacogenic carcinoma of anal canal	Cancer
408271	SNOMED CT	721718003	Primary adenocarcinoma of ampulla of Vater	Cancer
408278	SNOMED CT	721725005	Primary adenocarcinoma of peritoneum	Cancer
408580	SNOMED CT	722103009	Hormone sensitive prostate cancer	Cancer
408860	SNOMED CT	722425009	Reactive oxygen species 1 positive non-small cell lung cancer	Cancer
408874	SNOMED CT	722445001	Primary giant cell sarcoma of peritoneum	Cancer
408929	SNOMED CT	722509001	Primary rhabdomyosarcoma of oral cavity	Cancer
408930	SNOMED CT	722510006	Primary rhabdomyosarcoma of pharynx	Cancer
408932	SNOMED CT	722512003	Primary rhabdomyosarcoma of intrathoracic organ	Cancer
408934	SNOMED CT	722514002	Primary leiomyosarcoma of peritoneum	Cancer
408936	SNOMED CT	722516000	Primary liposarcoma of peritoneum	Cancer
408939	SNOMED CT	722519007	Primary sarcoma of peritoneum	Cancer
408944	SNOMED CT	722524005	Primary invasive pleomorphic lobular carcinoma of breast	Cancer
408946	SNOMED CT	722527003	Primary malignant neuroendocrine neoplasm of bronchus	Cancer

408947	SNOMED CT	722528008	Primary malignant neuroendocrine neoplasm of lung	Cancer
408948	SNOMED CT	722529000	Primary malignant epithelial neoplasm of nasopharynx	Cancer
408949	SNOMED CT	722530005	Primary squamous cell carcinoma of pharyngeal tonsil	Cancer
408952	SNOMED CT	722533007	Primary malignant neoplasm of esophagogastric junction	Cancer
408961	SNOMED CT	722542000	Primary squamous cell carcinoma of anal canal	Cancer
408962	SNOMED CT	722543005	Primary malignant melanoma of anal canal	Cancer
409071	SNOMED CT	722665003	Primary malignant melanoma of cornea	Cancer
409072	SNOMED CT	722666002	Primary squamous cell carcinoma of lacrimal apparatus	Cancer
409075	SNOMED CT	722670005	Primary thymic carcinoma	Cancer
409076	SNOMED CT	722671009	Metastatic malignant neoplasm of meninges	Cancer
409077	SNOMED CT	722672002	Primary squamous cell carcinoma of base of tongue	Cancer
409078	SNOMED CT	722673007	Primary squamous cell carcinoma of lingual tonsil	Cancer
409079	SNOMED CT	722674001	Primary squamous cell carcinoma of parotid gland	Cancer
409081	SNOMED CT	722676004	Primary squamous cell carcinoma of middle ear	Cancer
409082	SNOMED CT	722677008	Primary mesothelioma of overlapping sites of retroperitoneum, peritoneum and omentum	Cancer
409083	SNOMED CT	722678003	Primary squamous cell carcinoma of vagina	Cancer
409084	SNOMED CT	722679006	Primary mucinous adenocarcinoma of endometrium	Cancer
409085	SNOMED CT	722680009	Primary serous adenocarcinoma of endometrium	Cancer
409086	SNOMED CT	722681008	Primary mixed adenocarcinoma of endometrium	Cancer
409087	SNOMED CT	722682001	Primary small cell carcinoma of endometrium	Cancer
409088	SNOMED CT	722683006	Primary neuroendocrine carcinoma of cervix uteri	Cancer
409089	SNOMED CT	722684000	Primary low grade serous adenocarcinoma of ovary	Cancer
409090	SNOMED CT	722685004	Primary high grade serous adenocarcinoma of ovary	Cancer

409110	SNOMED CT	722714004	Primary mucoepidermoid carcinoma of lacrimal apparatus	Cancer
409111	SNOMED CT	722718001	Primary malignant meningioma	Cancer
409198	SNOMED CT	722828003	Primary synovial sarcoma of intrathoracic organ	Cancer
409202	SNOMED CT	722832009	Primary solid papillary carcinoma with invasion of breast	Cancer
409418	SNOMED CT	723077004	Primary myosarcoma of uterus	Cancer
409517	SNOMED CT	723182009	Primary squamous cell carcinoma of nasal cavity	Cancer
409563	SNOMED CT	723265000	Primary squamous cell carcinoma of anus	Cancer
409577	SNOMED CT	723281005	Primary malignant melanoma of anus	Cancer
409587	SNOMED CT	723301009	Squamous non-small cell lung cancer	Cancer
409963	SNOMED CT	723843005	Primary chondrosarcoma of bone of limb	Cancer
409966	SNOMED CT	723846002	Primary chondrosarcoma of bone of rib	Cancer
409967	SNOMED CT	723847006	Primary chondrosarcoma of articular cartilage of rib	Cancer
409968	SNOMED CT	723848001	Primary osteosarcoma of bone of jaw	Cancer
409969	SNOMED CT	723849009	Primary osteosarcoma of articular cartilage of jaw	Cancer
409970	SNOMED CT	723850009	Primary osteosarcoma of bone of limb	Cancer
409973	SNOMED CT	723853006	Primary Ewing sarcoma of bone of limb	Cancer
409976	SNOMED CT	723856003	Primary Ewing sarcoma of articular cartilage of rib	Cancer
409999	SNOMED CT	723879002	Primary adenocarcinoma of overlapping sites of urinary organ	Cancer
410006	SNOMED CT	723887001	Primary Ewing sarcoma of bone of rib	Cancer
410118	SNOMED CT	724056005	Malignant neoplasm of lower lobe of right lung	Cancer
410119	SNOMED CT	724058006	Malignant neoplasm of upper lobe of left lung	Cancer
410120	SNOMED CT	724059003	Malignant neoplasm of lower lobe of left lung	Cancer
410121	SNOMED CT	724060008	Malignant neoplasm of right upper lobe of lung	Cancer
410390	SNOMED CT	724467001	Primary squamous cell carcinoma of overlapping sites of urinary organs	Cancer

410391	SNOMED CT	724468006	Primary urothelial carcinoma of overlapping sites of urinary organs	Cancer
410467	SNOMED CT	724552002	Primary poorly differentiated carcinoma of thyroid gland	Cancer
410468	SNOMED CT	724553007	Primary undifferentiated carcinoma of thyroid gland	Cancer
410469	SNOMED CT	724554001	Primary malignant epithelial neoplasm of endocrine gland	Cancer
411625	SNOMED CT	726019003	Familial malignant melanoma of skin	Cancer
412004	SNOMED CT	726652005	Malignant carcinoid tumor of thymus	Cancer
412005	SNOMED CT	726653000	Malignant carcinoid tumor of bronchus	Cancer
412006	SNOMED CT	726654006	Malignant carcinoid tumor of colon	Cancer
417456	SNOMED CT	732201008	Endometrial carcinosarcoma	Cancer
418270	SNOMED CT	733064004	Osteosarcoma, limb anomalies, erythroid macrocytosis syndrome	Cancer
418333	SNOMED CT	733134009	Primary squamous cell carcinoma of paraurethral gland	Cancer
418334	SNOMED CT	733135005	Primary urothelial carcinoma of paraurethral gland	Cancer
418335	SNOMED CT	733136006	Primary adenocarcinoma of urethra	Cancer
418342	SNOMED CT	733144006	Malignant epithelial neoplasm of bronchus	Cancer
418358	SNOMED CT	733162002	Primary malignant neuroendocrine neoplasm of anus	Cancer
418359	SNOMED CT	733163007	Primary malignant neuroendocrine neoplasm of anal canal	Cancer
418525	SNOMED CT	733343005	Primary squamous cell carcinoma of oral cavity	Cancer
418526	SNOMED CT	733344004	Primary squamous cell carcinoma of lip	Cancer
418527	SNOMED CT	733345003	Primary squamous cell carcinoma of pharynx	Cancer
418533	SNOMED CT	733351008	Primary malignant neuroendocrine neoplasm of ampulla of Vater	Cancer
418539	SNOMED CT	733357007	Primary squamous cell carcinoma of intrathoracic organ	Cancer
418541	SNOMED CT	733359005	Primary squamous cell carcinoma of endometrium	Cancer
418542	SNOMED CT	733360000	Primary undifferentiated carcinoma of endometrium	Cancer

418543	SNOMED CT	733361001	Primary mucinous adenocarcinoma of ovary	Cancer
418544	SNOMED CT	733362008	Primary adenocarcinoma of paraurethral gland	Cancer
418627	SNOMED CT	733470002	Collecting duct carcinoma of kidney	Cancer
418628	SNOMED CT	733471003	Chromophobe renal cell carcinoma	Cancer
418646	SNOMED CT	733491005	Carney complex	Cancer
418694	SNOMED CT	733603009	Tubulocystic renal cell carcinoma	Cancer
418699	SNOMED CT	733608000	Papillary renal cell carcinoma	Cancer
418816	SNOMED CT	733834006	Invasive carcinoma of uterine cervix co-occurrent with human immunodeficiency virus infection	Cancer
418898	SNOMED CT	733926004	Ganglioneuroblastoma of central nervous system	Cancer
418968	SNOMED CT	734015000	Clear cell papillary renal cell carcinoma	Cancer
419038	SNOMED CT	734099007	Neuroblastoma of central nervous system	Cancer
419768	SNOMED CT	735082004	Fordyce angiokeratoma of scrotum	Cancer
419997	SNOMED CT	735450006	Primary malignant neuroepitheliomatous neoplasm of nasal cavity	Cancer
420220	SNOMED CT	735679005	Primary chondrosarcoma of bone	Cancer
420266	SNOMED CT	735757008	Primary ganglioneuroblastoma of brain	Cancer
420421	SNOMED CT	735916009	Primary malignant neuroepithelial neoplasm of retina	Cancer
420423	SNOMED CT	735918005	Primary malignant neoplasm of lacrimal caruncle	Cancer
420424	SNOMED CT	735919002	Primary malignant neuroepithelial neoplasm of iris	Cancer
420425	SNOMED CT	735920008	Primary malignant neuroepithelial neoplasm of ciliary body	Cancer
420426	SNOMED CT	735921007	Primary malignant neuroepithelial neoplasm of orbit	Cancer
421411	SNOMED CT	737267003	Hemolymphangioma of conjunctiva	Cancer
421447	SNOMED CT	737308008	Primary adenocarcinoma of sublingual gland	Cancer

421448	SNOMED CT	737309000	Primary adenocarcinoma of submandibular gland	Cancer
421449	SNOMED CT	737310005	Primary squamous cell carcinoma of submandibular gland	Cancer
421450	SNOMED CT	737311009	Primary squamous cell carcinoma of sublingual gland	Cancer
421800	SNOMED CT	738770003	Anaplastic lymphoma kinase positive anaplastic large cell lymphoma	Cancer
421901	SNOMED CT	739301006	Osteoporosis co-occurrent and due to multiple myeloma	Cancer
422547	SNOMED CT	762457009	Astroblastoma of brain	Cancer
422548	SNOMED CT	762458004	Primary endometrioid carcinoma of endometrium of body of uterus	Cancer
422898	SNOMED CT	763063001	Adenoid basal carcinoma of cervix uteri	Cancer
422899	SNOMED CT	763064007	Adenoid cystic carcinoma of cervix uteri	Cancer
422961	SNOMED CT	763131005	Clear cell adenocarcinoma of ovary	Cancer
423182	SNOMED CT	763408003	Rhabdomyosarcoma of cervix uteri	Cancer
423183	SNOMED CT	763409006	Rhabdomyosarcoma of corpus uteri	Cancer
423236	SNOMED CT	763479005	Metaplastic carcinoma of breast	Cancer
423461	SNOMED CT	763771009	Leiomyosarcoma of cervix uteri	Cancer
424046	SNOMED CT	764694005	MiT family translocation renal cell carcinoma	Cancer
424088	SNOMED CT	764737005	Squamous cell carcinoma of corpus uteri	Cancer
424179	SNOMED CT	764845008	Adenocarcinoma of anal canal	Cancer
424180	SNOMED CT	764846009	Adenocarcinoma of penis	Cancer
424181	SNOMED CT	764847000	Adenosarcoma of cervix uteri	Cancer
424190	SNOMED CT	764856008	Acquired cystic disease associated renal cell carcinoma	Cancer
424272	SNOMED CT	764951002	Carcinosarcoma of cervix uteri	Cancer
424273	SNOMED CT	764952009	Carcinosarcoma of corpus uteri	Cancer
424281	SNOMED CT	764961009	Hereditary clear cell renal cell carcinoma	Cancer
424306	SNOMED CT	764990003	Mucinous tubular and spindle cell renal carcinoma	Cancer

424383	SNOMED CT	765095002	Renal medullary carcinoma	Cancer
424464	SNOMED CT	765190005	Fibrothecoma of ovary	Cancer
424799	SNOMED CT	765740002	Adenosarcoma of corpus uteri	Cancer
425052	SNOMED CT	766247009	Primitive neuroectodermal tumor of corpus uteri	Cancer
425053	SNOMED CT	766248004	Primitive neuroectodermal tumor of cervix uteri	Cancer
425357	SNOMED CT	766757006	Undifferentiated carcinoma of stomach	Cancer
425358	SNOMED CT	766758001	Undifferentiated carcinoma of corpus uteri	Cancer
425359	SNOMED CT	766759009	Vulvovaginal rhabdomyosarcoma	Cancer
425427	SNOMED CT	766881008	Carney complex, trismus, pseudocamptodactyly syndrome	Cancer
425467	SNOMED CT	766930002	Glassy cell carcinoma of cervix uteri	Cancer
425511	SNOMED CT	766979005	Squamous cell carcinoma of rectum	Cancer
425512	SNOMED CT	766980008	Squamous cell carcinoma of stomach	Cancer
425873	SNOMED CT	767444009	Germline BRCA-mutated, HER2-negative metastatic breast cancer	Cancer
425877	SNOMED CT	767448007	Pineoblastoma	Cancer
426755	SNOMED CT	768470007	Aldosterone-producing adenoma	Cancer
428272	SNOMED CT	770601003	Small cell carcinoma of ovary	Cancer
428295	SNOMED CT	770628008	Diffuse leptomeningeal melanocytosis	Cancer
428340	SNOMED CT	770686005	Malignant germ cell neoplasm of vagina	Cancer
428615	SNOMED CT	771080008	Hereditary site-specific ovarian cancer syndrome	Cancer
430159	SNOMED CT	772992009	Primary differentiated carcinoma of thyroid gland	Cancer
430303	SNOMED CT	773283006	Malignant germ cell neoplasm of cervix uteri	Cancer
430304	SNOMED CT	773284000	Malignant germ cell neoplasm of corpus uteri	Cancer
430584	SNOMED CT	773624006	Primary ameloblastic carcinoma	Cancer
430697	SNOMED CT	773774000	High-grade neuroendocrine carcinoma of corpus uteri	Cancer
430698	SNOMED CT	773775004	High-grade neuroendocrine carcinoma of cervix uteri	Cancer

434763	SNOMED CT	778046002	Somatomammotropinoma	Cancer
434778	SNOMED CT	778066006	Carcinofibroma of corpus uteri	Cancer
437488	SNOMED CT	781076008	Metastatic colorectal cancer	Cancer
437747	SNOMED CT	781382000	Malignant neoplasm of colon and/or rectum	Cancer
438765	SNOMED CT	782722002	Global developmental delay, lung cysts, overgrowth, Wilms tumor syndrome	Cancer
438992	SNOMED CT	783006001	Low-grade neuroendocrine neoplasm of corpus uteri	Cancer
438994	SNOMED CT	783008000	Pituitary dermoid and epidermoid cysts	Cancer
439037	SNOMED CT	783056006	Paratesticular adenocarcinoma	Cancer
439150	SNOMED CT	783183009	Salivary gland type carcinoma of esophagus	Cancer
439474	SNOMED CT	783704005	Undifferentiated carcinoma of esophagus	Cancer
441021	SNOMED CT	785807007	Transitional cell carcinoma of corpus uteri	Cancer
441224	SNOMED CT	786038001	Familial nonmedullary thyroid carcinoma	Cancer
442864	SNOMED CT	788756004	Spindle cell oncocytoma of posterior pituitary gland	Cancer
442865	SNOMED CT	788757008	Pituicytoma of posterior pituitary gland	Cancer
442866	SNOMED CT	788758003	Sellar ependymoma of posterior pituitary gland	Cancer
443513	SNOMED CT	789689004	Malignant lymphomatoid granulomatosis grade 3	Cancer
443514	SNOMED CT	789690008	Malignant lymphomatoid granulomatosis grade 3 of lung	Cancer
443543	SNOMED CT	789721005	Malignant middle ear paraganglioma	Cancer
443886	SNOMED CT	816205008	Malignant pituitary blastoma	Cancer
443988	SNOMED CT	818967003	Medulloepithelioma of central nervous system	Cancer
444093	SNOMED CT	822969007	Acinar cell cystadenocarcinoma of lung	Cancer
444094	SNOMED CT	822970008	Acinar cell cystadenocarcinoma of prostate	Cancer
444129	SNOMED CT	823017009	Infiltrating duct carcinoma of prostate	Cancer
444285	SNOMED CT	827162007	Malignant immature teratoma of ovary	Cancer
444353	SNOMED CT	830032008	Dermoid cyst of occipital lobe	Cancer

444354	SNOMED CT	830033003	Dermoid cyst of brain	Cancer
444375	SNOMED CT	830055006	Anaplastic lymphoma kinase fusion oncogene negative non-small cell lung cancer	Cancer
444379	SNOMED CT	830060005	Reactive oxygen species 1 negative non-small cell lung cancer	Cancer
444442	SNOMED CT	830151004	Anaplastic lymphoma kinase fusion oncogene positive non-small cell lung cancer	Cancer
444475	SNOMED CT	830196006	Chordoma of cervical spine	Cancer
444547	SNOMED CT	833285007	Chordoma of lumbar spine	Cancer
444556	SNOMED CT	833295000	Chordoma of thoracic spine	Cancer
444609	SNOMED CT	836274002	Carcinomatosis of peritoneum	Cancer
445251	SNOMED CT	840573001	Carcinoma of epididymis and spermatic cord	Cancer
445718	SNOMED CT	860830009	Gastrointestinal stromal neoplasm of overlapping sites of stomach	Cancer
446017	SNOMED CT	865954003	Adenocarcinoma of breast	Cancer
446099	SNOMED CT	866050001	Mixed germ cell neoplasm of central nervous system	Cancer
446120	SNOMED CT	866072004	Malignant melanoma of orbit	Cancer
446121	SNOMED CT	866073009	Malignant melanoma of overlapping sites of eye and adnexa	Cancer
446127	SNOMED CT	866079008	Malignant melanoma of lacrimal gland	Cancer
446128	SNOMED CT	866080006	Malignant germ cell neoplasm of heart	Cancer
446130	SNOMED CT	866082003	Malignant melanoma of lacrimal drainage system	Cancer
446135	SNOMED CT	866087009	Pathologic fracture of vertebra at site of metastatic neoplasm	Cancer
446212	SNOMED CT	866176003	Primary clear cell peritoneal carcinoma	Cancer
446499	SNOMED CT	870327007	Papillary squamous cell carcinoma of nasopharynx	Cancer
446490	SNOMED CT	870318006	Osteosarcoma of bone of head	Cancer
446527	SNOMED CT	870355006	Neuroendocrine carcinoma of anus	Cancer
447879	SNOMED CT	1.661E+12	Metastasis to lung from adenocarcinoma	Cancer

449241	SNOMED CT	7.391E+12	Primary adenoid cystic carcinoma of nasopharynx	Cancer
449485	SNOMED CT	1.147E+13	Diffuse intrinsic pontine glioma	Cancer
449838	SNOMED CT	1.812E+13	Primary squamous cell carcinoma of palatine tonsil	Cancer
449867	SNOMED CT	1.883E+13	Benign multiple endocrine neoplasia type 2a	Cancer
450361	SNOMED CT	3.358E+13	Primary malignant neoplasm of extradural spinal cord	Cancer
450461	SNOMED CT	4.522E+13	Primary invasive malignant neoplasm of female breast	Cancer
450581	SNOMED CT	6.147E+13	Basal cell carcinoma of naris	Cancer
450695	SNOMED CT	6.777E+13	Grade 2 astrocytoma of brain	Cancer
450696	SNOMED CT	6.781E+13	Primary small cell malignant neoplasm of lung, TNM stage 1	Cancer
450697	SNOMED CT	6.782E+13	Primary small cell malignant neoplasm of lung, TNM stage 2	Cancer
450698	SNOMED CT	6.783E+13	Primary small cell malignant neoplasm of lung, TNM stage 3	Cancer
450699	SNOMED CT	6.784E+13	Primary small cell malignant neoplasm of lung, TNM stage 4	Cancer
450751	SNOMED CT	7.111E+13	Recurrent primary malignant neoplasm of vulva	Cancer
450819	SNOMED CT	7.441E+13	Prostatic intraepithelial neoplasia and adjacent atypia	Cancer
450849	SNOMED CT	7.841E+13	Ewing sarcoma of bone of pelvis	Cancer
450882	SNOMED CT	8.25E+13	Anaplastic astrocytoma of central nervous system	Cancer
450889	SNOMED CT	8.276E+13	Osteosarcoma of bone of upper limb	Cancer
450945	SNOMED CT	8.709E+13	Malignant glioma of cerebrum	Cancer
450946	SNOMED CT	8.71E+13	Primary malignant glioma of brain	Cancer
450947	SNOMED CT	8.711E+13	Malignant glioma of hypothalamus	Cancer
450948	SNOMED CT	8.712E+13	Malignant glioma of cerebellum	Cancer
450949	SNOMED CT	8.713E+13	Primary extramedullary malignant tumor of spinal cord	Cancer
450950	SNOMED CT	8.715E+13	Malignant glioma of central nervous system	Cancer
451021	SNOMED CT	9.081E+13	Low grade malignant glioma of brain	Cancer
451022	SNOMED CT	9.083E+13	Grade 4 malignant glioma of brain	Cancer

451025	SNOMED CT	9.103E+13	Primary chondrosarcoma of bone of upper limb	Cancer
451026	SNOMED CT	9.104E+13	Ewing sarcoma of bone structure of upper limb	Cancer
451027	SNOMED CT	9.106E+13	Primary chondrosarcoma of bone of pelvis	Cancer
451028	SNOMED CT	9.108E+13	Primary chondrosarcoma of bone of lower limb	Cancer
451029	SNOMED CT	9.113E+13	Primary adenocarcinoma of vulva	Cancer
451030	SNOMED CT	9.114E+13	Primary spindle cell carcinoma of urinary bladder	Cancer
451031	SNOMED CT	9.115E+13	Primary squamous cell carcinoma of chest wall	Cancer
451032	SNOMED CT	9.116E+13	Primary adenocarcinoma of chest wall	Cancer
451033	SNOMED CT	9.117E+13	Primary undifferentiated large cell malignancy of chest wall	Cancer
451034	SNOMED CT	9.118E+13	Squamous cell carcinoma metastatic to pleura	Cancer
451036	SNOMED CT	9.128E+13	Secondary adenocarcinoma of bone	Cancer
451066	SNOMED CT	9.428E+13	Malignant multiple endocrine neoplasia type 2a	Cancer
451092	SNOMED CT	9.629E+13	Primary malignant inflammatory neoplasm of female breast	Cancer
451104	SNOMED CT	9.69E+13	Prostate cancer metastatic to eye	Cancer
451154	SNOMED CT	9.898E+13	Primary malignant neoplasm of ileocecal valve	Cancer
451158	SNOMED CT	9.91E+13	Primary adenocarcinoma of fallopian tube	Cancer
451160	SNOMED CT	9.912E+13	Primary adenocarcinoma of vagina	Cancer
451161	SNOMED CT	9.913E+13	Astrocytoma of cerebrum	Cancer
451181	SNOMED CT	1.007E+14	High grade astrocytoma of brain	Cancer
451182	SNOMED CT	1.007E+14	Low grade astrocytoma of brain	Cancer
451192	SNOMED CT	1.014E+14	Adamantinoma of long bone of lower limb	Cancer
451199	SNOMED CT	1.016E+14	Ependymoma of cerebrum	Cancer
451253	SNOMED CT	1.05E+14	Oligodendroglioma of cerebrum	Cancer
451255	SNOMED CT	1.051E+14	Primary squamous cell carcinoma of thymus	Cancer
451256	SNOMED CT	1.051E+14	Squamous cell carcinoma of vagina	Cancer

451302	SNOMED CT	1.076E+14	Ependymoma of brain stem	Cancer
451303	SNOMED CT	1.076E+14	Oligodendroglioma of brain stem	Cancer
451304	SNOMED CT	1.076E+14	Astrocytoma of brain stem	Cancer
451305	SNOMED CT	1.076E+14	Primary squamous cell carcinoma of urethra	Cancer
451306	SNOMED CT	1.076E+14	Primary transitional cell carcinoma of urethra	Cancer
451308	SNOMED CT	1.077E+14	Non-seminomatous germ cell neoplasm of testis	Cancer
451311	SNOMED CT	1.078E+14	Primary malignant mixed Mullerian neoplasm of endometrium	Cancer
451312	SNOMED CT	1.078E+14	Primary malignant clear cell neoplasm of endometrium	Cancer
451314	SNOMED CT	1.078E+14	Primary adenosquamous carcinoma of endometrium	Cancer
451320	SNOMED CT	1.081E+14	Malignant melanoma metastatic to kidney	Cancer
451322	SNOMED CT	1.081E+14	Small cell carcinoma metastatic to kidney	Cancer
451323	SNOMED CT	1.082E+14	Secondary undifferentiated large cell malignant neoplasm of kidney	Cancer
451324	SNOMED CT	1.082E+14	Secondary adenocarcinoma of kidney	Cancer
451401	SNOMED CT	1.131E+14	Chondromyxoid fibroma of long bone of lower limb	Cancer
451402	SNOMED CT	1.131E+14	Chondromyxoid fibroma of long bone of upper limb	Cancer
451405	SNOMED CT	1.131E+14	Osteochondroma of fibula	Cancer
451406	SNOMED CT	1.131E+14	Osteochondroma of humerus	Cancer
451407	SNOMED CT	1.131E+14	Osteochondroma of pelvis	Cancer
451618	SNOMED CT	1.232E+14	Benign carcinoid tumor of stomach	Cancer
451636	SNOMED CT	1.237E+14	Malignant carcinoid tumor of descending colon	Cancer
451732	SNOMED CT	1.28E+14	Primary adenocarcinoma of distal third of esophagus	Cancer
451778	SNOMED CT	1.304E+14	Primary malignant neuroendocrine neoplasm of large intestine	Cancer
451780	SNOMED CT	1.304E+14	Primary malignant neuroendocrine neoplasm of cecum	Cancer

451802	SNOMED CT	1.309E+14	Chordoma of coccyx	Cancer
451803	SNOMED CT	1.309E+14	Chordoma of pelvis	Cancer
451903	SNOMED CT	1.351E+14	Secondary neuroendocrine carcinoma of peritoneum	Cancer
452158	SNOMED CT	1.471E+14	Primary malignant astrocytoma of central nervous system	Cancer
452160	SNOMED CT	1.471E+14	Glioblastoma multiforme of central nervous system	Cancer
452261	SNOMED CT	1.847E+14	Primary adenocarcinoma of anus	Cancer
452262	SNOMED CT	1.848E+14	Primary adenocarcinoma of cervix uteri	Cancer
452263	SNOMED CT	1.848E+14	Primary adenocarcinoma of lacrimal gland	Cancer
452264	SNOMED CT	1.849E+14	Primary adenocarcinoma of nasopharynx	Cancer
452282	SNOMED CT	2.08E+14	Primary adenocarcinoma of endocervix	Cancer
452291	SNOMED CT	2.265E+14	Primary malignant neoplasm of nasopharynx	Cancer
452298	SNOMED CT	2.349E+14	Malignant glioma of eye	Cancer
452300	SNOMED CT	2.368E+14	Paget disease of anal canal	Cancer
452306	SNOMED CT	2.43E+14	Primary squamous cell carcinoma of vermilion border of lip	Cancer
454376	SNOMED CT	3.509E+14	Primary malignant neoplasm of right adrenal gland	Cancer
454381	SNOMED CT	3.509E+14	Primary malignant neoplasm of left adrenal gland	Cancer
454384	SNOMED CT	3.512E+14	Benign carcinoid of lung	Cancer
454406	SNOMED CT	3.52E+14	Malignant melanoma of skin of right upper limb	Cancer
454407	SNOMED CT	3.52E+14	Malignant melanoma of skin of right lower limb	Cancer
454412	SNOMED CT	3.522E+14	Malignant melanoma of skin of left upper limb	Cancer
454423	SNOMED CT	3.534E+14	Primary malignant neoplasm of axillary tail of left female breast	Cancer
454424	SNOMED CT	3.534E+14	Primary malignant neoplasm of female left breast	Cancer
454425	SNOMED CT	3.534E+14	Primary malignant neoplasm of central portion of female left breast	Cancer
454426	SNOMED CT	3.535E+14	Primary malignant neoplasm of axillary tail of right female breast	Cancer

454427	SNOMED CT	3.535E+14	Primary malignant neoplasm of central portion of female right breast	Cancer
454428	SNOMED CT	3.536E+14	Secondary malignant neoplasm of right lung	Cancer
454429	SNOMED CT	3.537E+14	Overlapping primary malignant neoplasm of bone and articular cartilage of lower limb	Cancer
454430	SNOMED CT	3.537E+14	Overlapping primary malignant neoplasm of bone and articular cartilage of right upper limb	Cancer
454431	SNOMED CT	3.537E+14	Secondary malignant neoplasm of left lung	Cancer
454452	SNOMED CT	3.544E+14	Primary malignant neoplasm of left kidney	Cancer
454453	SNOMED CT	3.544E+14	Primary malignant neoplasm of right kidney	Cancer
454456	SNOMED CT	3.545E+14	Primary malignant neoplasm of left male breast	Cancer
454457	SNOMED CT	3.546E+14	Primary malignant neoplasm of right male breast	Cancer
454458	SNOMED CT	3.546E+14	Overlapping primary malignant neoplasm of bone and articular cartilage of left upper limb	Cancer
454459	SNOMED CT	3.546E+14	Primary malignant neoplasm of bone of left upper limb	Cancer
454460	SNOMED CT	3.547E+14	Primary malignant neoplasm of bone of left lower limb	Cancer
454461	SNOMED CT	3.547E+14	Primary malignant neoplasm of bone of right upper limb	Cancer
454462	SNOMED CT	3.547E+14	Primary malignant neoplasm of bone of right lower limb	Cancer
454463	SNOMED CT	3.547E+14	Primary malignant neoplasm of left lung	Cancer
454464	SNOMED CT	3.547E+14	Primary malignant neoplasm of right lung	Cancer
455099	SNOMED CT	4.319E+14	Gnathic osteosarcoma	Cancer
455121	SNOMED CT	4.321E+14	Malignant mesothelioma of the tunica vaginalis	Cancer
456399	SNOMED CT	4.465E+14	Meningioma of cerebellum	Cancer
456914	SNOMED CT	4.523E+14	Relapse multiple myeloma	Cancer
457455	SNOMED CT	4.583E+14	Metastatic urothelial carcinoma	Cancer
457481	SNOMED CT	4.586E+14	Metastatic HER2 positive gastroesophageal junction cancer	Cancer

457545	SNOMED CT	4.594E+14	Metastatic castration-resistant prostate cancer	Cancer
457546	SNOMED CT	4.594E+14	Metastatic human epidermal growth factor 2 positive carcinoma of breast	Cancer
457548	SNOMED CT	4.594E+14	Metastatic collecting duct carcinoma	Cancer
457549	SNOMED CT	4.594E+14	Metastatic penile cancer	Cancer
457725	SNOMED CT	4.614E+14	Breast implant-associated anaplastic large cell lymphoma	Cancer
457727	SNOMED CT	4.614E+14	Recurrent ovarian cancer	Cancer
458066	SNOMED CT	6.816E+14	Primary adenocarcinoma of body of stomach	Cancer
458067	SNOMED CT	6.817E+14	Primary adenocarcinoma of descending colon	Cancer
458069	SNOMED CT	6.827E+14	Benign neoplasm of left lacrimal gland	Cancer
458070	SNOMED CT	6.828E+14	Benign neoplasm of right lacrimal gland	Cancer
458087	SNOMED CT	6.84E+14	Extensive stage primary small cell carcinoma of lung	Cancer
458088	SNOMED CT	6.849E+14	Primary glioblastoma multiforme of frontal lobe	Cancer
458557	SNOMED CT	1.079E+15	Primary adenocarcinoma of lower lobe of left lung	Cancer
458558	SNOMED CT	1.079E+15	Primary adenocarcinoma of upper lobe of left lung	Cancer
458559	SNOMED CT	1.079E+15	Primary adenocarcinoma of lower lobe of right lung	Cancer
458560	SNOMED CT	1.079E+15	Primary adenocarcinoma of upper lobe of right lung	Cancer
458561	SNOMED CT	1.079E+15	Angiomyolipoma of left kidney	Cancer
458562	SNOMED CT	1.079E+15	Angiomyolipoma of right kidney	Cancer
458566	SNOMED CT	1.079E+15	Primary basal cell carcinoma of left eyelid	Cancer
458570	SNOMED CT	1.079E+15	Primary basal cell carcinoma of right eyelid	Cancer
458618	SNOMED CT	1.08E+15	Infiltrating ductal carcinoma of axillary tail of left female breast	Cancer
458620	SNOMED CT	1.08E+15	Infiltrating duct carcinoma of left female breast	Cancer
458621	SNOMED CT	1.08E+15	Infiltrating ductal carcinoma of central portion of left female breast	Cancer
458622	SNOMED CT	1.08E+15	Infiltrating ductal carcinoma of lower inner quadrant of left female breast	Cancer

458623	SNOMED CT	1.08E+15	Infiltrating ductal carcinoma of lower outer quadrant of left female breast	Cancer
458624	SNOMED CT	1.08E+15	Infiltrating ductal carcinoma of upper inner quadrant of left female breast	Cancer
458625	SNOMED CT	1.08E+15	Infiltrating ductal carcinoma of upper outer quadrant of left female breast	Cancer
458626	SNOMED CT	1.08E+15	Infiltrating ductal carcinoma of axillary tail of right female breast	Cancer
458627	SNOMED CT	1.08E+15	Invasive ductal carcinoma of right female breast	Cancer
458628	SNOMED CT	1.08E+15	Infiltrating ductal carcinoma of central portion of right female breast	Cancer
458630	SNOMED CT	1.08E+15	Infiltrating ductal carcinoma of lower inner quadrant of right female breast	Cancer
458631	SNOMED CT	1.08E+15	Infiltrating ductal carcinoma of lower outer quadrant of right female breast	Cancer
458632	SNOMED CT	1.08E+15	Infiltrating ductal carcinoma of upper inner quadrant of right female breast	Cancer
458633	SNOMED CT	1.08E+15	Infiltrating ductal carcinoma of upper outer quadrant of right female breast	Cancer
458634	SNOMED CT	1.08E+15	Infiltrating lobular carcinoma of left female breast	Cancer
458636	SNOMED CT	1.08E+15	Infiltrating lobular carcinoma of right female breast	Cancer
458652	SNOMED CT	1.081E+15	Malignant melanoma of left choroid	Cancer
458653	SNOMED CT	1.081E+15	Malignant melanoma of right choroid	Cancer
458655	SNOMED CT	1.081E+15	Malignant melanoma of skin of left lower limb	Cancer
458658	SNOMED CT	1.081E+15	Oncocytoma of left kidney	Cancer
458659	SNOMED CT	1.081E+15	Oncocytoma of right kidney	Cancer
458668	SNOMED CT	1.082E+15	Recurrent primary malignant neoplasm of left female breast	Cancer
458669	SNOMED CT	1.082E+15	Recurrent primary malignant neoplasm of right female breast	Cancer
458670	SNOMED CT	1.082E+15	Clear cell carcinoma of left kidney	Cancer
458671	SNOMED CT	1.082E+15	Clear cell carcinoma of right kidney	Cancer
458674	SNOMED CT	1.082E+15	Primary seminoma of left testis	Cancer

458676	SNOMED CT	1.082E+15	Primary seminoma of right testis	Cancer
458684	SNOMED CT	1.082E+15	Transitional cell carcinoma of left renal pelvis	Cancer
458685	SNOMED CT	1.082E+15	Transitional cell carcinoma of left ureter	Cancer
458686	SNOMED CT	1.082E+15	Transitional cell carcinoma of right renal pelvis	Cancer
458687	SNOMED CT	1.082E+15	Transitional cell carcinoma of right ureter	Cancer
458688	SNOMED CT	1.082E+15	Nephroblastoma of left kidney	Cancer
458689	SNOMED CT	1.082E+15	Nephroblastoma of right kidney	Cancer
458706	SNOMED CT	1.083E+15	Locally advanced breast cancer	Cancer
458718	SNOMED CT	1.083E+15	Primary malignant neoplasm of breast with axillary lymph node invasion	Cancer
459300	SNOMED CT	1.099E+15	Recurrent malignant neoplasm of prostate	Cancer
459770	SNOMED CT	1.071E+16	Primary malignant neoplasm of uterus	Cancer
459785	SNOMED CT	1.074E+16	Malignant germ cell neoplasm of right ovary	Cancer
459786	SNOMED CT	1.074E+16	Malignant germ cell neoplasm of left ovary	Cancer
462201	SNOMED CT	1.224E+16	Large cell carcinoma of left lung	Cancer
462202	SNOMED CT	1.224E+16	Large cell carcinoma of right lung	Cancer
462287	SNOMED CT	1.224E+16	Squamous cell carcinoma of left lung	Cancer
462288	SNOMED CT	1.224E+16	Squamous cell carcinoma of right lung	Cancer
462337	SNOMED CT	1.225E+16	Secondary malignant neoplasm of bilateral adrenal glands	Cancer
462338	SNOMED CT	1.225E+16	Secondary malignant neoplasm of bilateral lungs	Cancer
462431	SNOMED CT	1.335E+16	Secondary malignant neoplasm of lymph nodes of neck from thyroid	Cancer
462643	SNOMED CT	1.564E+16	Primary malignant neoplasm of both ovaries	Cancer
462644	SNOMED CT	1.564E+16	Infiltrating duct carcinoma of bilateral female breasts	Cancer
462645	SNOMED CT	1.564E+16	Primary malignant neoplasm of bilateral female breasts	Cancer
462670	SNOMED CT	1.564E+16	Renal cell carcinoma of bilateral kidneys	Cancer

462698	SNOMED CT	1.564E+16	Angiomyolipoma of bilateral kidneys	Cancer
463878	SNOMED CT	1.593E+16	Carcinosarcoma of left ovary	Cancer
463879	SNOMED CT	1.593E+16	Carcinosarcoma of right ovary	Cancer
463880	SNOMED CT	1.593E+16	Carcinosarcoma of bilateral ovaries	Cancer
463997	SNOMED CT	1.596E+16	Secondary adenocarcinoma of bilateral lungs	Cancer
463998	SNOMED CT	1.596E+16	Adenocarcinoma of left lung	Cancer
463999	SNOMED CT	1.596E+16	Adenocarcinoma of right lung	Cancer
464001	SNOMED CT	1.596E+16	Transitional cell carcinoma of left kidney	Cancer
464002	SNOMED CT	1.596E+16	Transitional cell carcinoma of right kidney	Cancer
464070	SNOMED CT	1.598E+16	Capillary hemangioma of right orbit region	Cancer
464071	SNOMED CT	1.598E+16	Capillary hemangioma of left orbit region	Cancer
464699	SNOMED CT	1.609E+16	Angiosarcoma of skin of cheek	Cancer
465538	SNOMED CT	1.909E+16	Metastatic neoplasm of left basal ganglion	Cancer
496730	SNOMED CT	92511007	Burkitt's tumor of lymph nodes of axilla and upper limb	Cancer
496731	SNOMED CT	92512000	Burkitt's tumor of lymph nodes of head, face, and neck	Cancer
496733	SNOMED CT	92513005	Burkitt's tumor of lymph nodes of inguinal region and lower limb	Cancer
496822	SNOMED CT	94365007	Cancer metastatic to large intestine	Cancer
496824	SNOMED CT	94682004	Secondary malignant neoplasm of Waldeyer ring	Cancer
501921	SNOMED CT	254898001	Paget disease vulvar cancer	Cancer
502797	SNOMED CT	285420006	Immunoglobulin A myeloma	Cancer
502799	SNOMED CT	285421005	Immunoglobulin G myeloma	Cancer
502801	SNOMED CT	285422003	Immunoglobulin D myeloma	Cancer
511632	SNOMED CT	402911002	Buschke-L√∂wenstein tumor of penis	Cancer
511634	SNOMED CT	402912009	Vulval verrucous carcinoma of Buschke-Lowenstein	Cancer
513707	SNOMED CT	423829008	Invasive vulval Paget disease	Cancer

516796	SNOMED CT	1.237E+14	Malignant carcinoid tumour of lung	Cancer
516799	SNOMED CT	1.237E+14	Malignant carcinoid tumour of ascending colon	Cancer
516800	SNOMED CT	1.237E+14	Malignant carcinoid tumour of caecum	Cancer
516801	SNOMED CT	1.237E+14	Malignant carcinoid tumour of large intestine	Cancer
516803	SNOMED CT	1.238E+14	Malignant carcinoid tumour of duodenum	Cancer
516833	SNOMED CT	1.434E+14	Malignant carcinoid tumour of pancreas	Cancer
516840	SNOMED CT	1.927E+14	Malignant carcinoid tumour of appendix	Cancer
516895	SNOMED CT	3.535E+14	Primary malignant neoplasm of female right breast	Cancer
170426	SNOMED CT	187633009	Malignant neoplasm of dorsal surface of tongue	Cancer
170550	SNOMED CT	187760008	Malignant neoplasm of rectum, rectosigmoid junction and anus	Cancer
170618	SNOMED CT	187828007	Malignant neoplasm of nasal cavities, middle ear and accessory sinuses	Cancer
170654	SNOMED CT	187864008	Malignant neoplasm of middle lobe, bronchus or lung	Cancer
170671	SNOMED CT	187881004	Malignant neoplasm of thymus, heart and mediastinum	Cancer
170779	SNOMED CT	187989003	Malignant neoplasm soft tissues of cervical spine	Cancer
170799	SNOMED CT	188009001	Malignant neoplasm of connective and soft tissue of thorax	Cancer
170831	SNOMED CT	188042000	Malignant melanoma of temple	Cancer
170839	SNOMED CT	188050009	Malignant melanoma of breast	Cancer
170840	SNOMED CT	188051008	Malignant melanoma of buttock	Cancer
170857	SNOMED CT	188068007	Malignant melanoma of hip	Cancer
170858	SNOMED CT	188069004	Malignant melanoma of thigh	Cancer
170859	SNOMED CT	188070003	Malignant melanoma of knee	Cancer
170862	SNOMED CT	188073001	Malignant melanoma of ankle	Cancer
170863	SNOMED CT	188074007	Malignant melanoma of heel	Cancer
170864	SNOMED CT	188075008	Malignant melanoma of foot	Cancer

170865	SNOMED CT	188076009	Malignant melanoma of toe	Cancer
170866	SNOMED CT	188077000	Malignant melanoma of great toe	Cancer
170946	SNOMED CT	188157005	Malignant neoplasm of overlapping sites of breast	Cancer
170952	SNOMED CT	188163001	Malignant neoplasm of nipple and areola of male breast	Cancer
170957	SNOMED CT	188168005	Malignant neoplasm of ectopic site of male breast	Cancer
171233	SNOMED CT	188445006	Secondary malignant neoplasm of retroperitoneum and peritoneum	Cancer
171250	SNOMED CT	188462001	Secondary malignant neoplasm of brain and spinal cord	Cancer
171257	SNOMED CT	188469005	Secondary malignant neoplasm of cervix uteri	Cancer
171259	SNOMED CT	188471005	Secondary malignant neoplasm of epididymis and vas deferens	Cancer
171862	SNOMED CT	189125000	Skin tag in vagina	Cancer
171891	SNOMED CT	189155007	Benign neoplasm lacrimal sac	Cancer
171892	SNOMED CT	189156008	Benign neoplasm nasolacrimal duct	Cancer
171915	SNOMED CT	189179009	Craniopharyngioma	Cancer
172543	SNOMED CT	189815007	Pulmonary blastoma	Cancer
173520	SNOMED CT	190818004	Waldenstr√∂m macroglobulinemia	Cancer
428231	SNOMED CT	770559003	Leiomyosarcoma of corpus uteri	Cancer
1201162	SNOMED CT	1.011E+09	Primary mucoepidermoid carcinoma of nasopharynx	Cancer
1201165	SNOMED CT	1.011E+09	Primary lymphoepithelial carcinoma of nasopharynx	Cancer
1201179	SNOMED CT	1.011E+09	Primary salivary gland type neoplasm of nasopharynx	Cancer
1201185	SNOMED CT	1.011E+09	Primary malignant melanoma of overlapping sites of accessory sinuses	Cancer
1201187	SNOMED CT	1.011E+09	Primary papillary adenocarcinoma of nasopharynx	Cancer
1201208	SNOMED CT	1.011E+09	Adenoid squamous cell carcinoma of nasopharynx	Cancer
1201219	SNOMED CT	1.011E+09	Primary squamous cell carcinoma of superior wall of nasopharynx	Cancer
1201223	SNOMED CT	1.011E+09	Basaloid squamous cell carcinoma of nasopharynx	Cancer

1201224	SNOMED CT	1.011E+09	Squamous cell carcinoma of posterior wall of nasopharynx	Cancer
1201226	SNOMED CT	1.011E+09	Primary squamous cell carcinomas of anterior wall of nasopharynx	Cancer
1201228	SNOMED CT	1.011E+09	Primary squamous cell carcinoma of lateral wall of nasopharynx	Cancer
1201230	SNOMED CT	1.011E+09	Primary squamous cell carcinoma of overlapping sites of nasopharynx	Cancer
1201295	SNOMED CT	1.05E+14	Secondary squamous cell carcinoma of lung	Cancer
1201296	SNOMED CT	1.051E+14	Secondary undifferentiated large cell carcinoma of lung	Cancer
1201776	SNOMED CT	1.433E+14	Non-Hodgkin's lymphoma in remission	Cancer
1201779	SNOMED CT	1.563E+16	Subependymoma of brain	Cancer
1201860	SNOMED CT	1.596E+16	Bilateral oncocytoma of kidneys	Cancer
1202044	SNOMED CT	1.69E+16	Atypical meningioma of cerebral meninges	Cancer
1202071	SNOMED CT	2.552E+13	Neoplasm of uncertain behavior of hypothalamus	Cancer
1202806	SNOMED CT	7.11E+13	Recurrent primary malignant neoplasm of vagina	Cancer
1203019	SNOMED CT	878805009	Renal granular cell carcinoma	Cancer
1203021	SNOMED CT	878807001	HER2-positive gastric cancer	Cancer
1203022	SNOMED CT	878808006	Nongerminomatous germ cell tumor of central nervous system	Cancer
1203052	SNOMED CT	878857009	Malignant lymphomatoid granulomatosis grade 3	Cancer
1203053	SNOMED CT	878858004	Malignant lymphomatoid granulomatosis grade 3 of lung	Cancer
1203115	SNOMED CT	879818005	Fetal sacral teratoma	Cancer
1203228	SNOMED CT	879991002	Gastrinoma of pancreas	Cancer
1203229	SNOMED CT	879992009	Gastrinoma of duodenum	Cancer
1203288	SNOMED CT	880078001	11p15 deletion syndrome	Cancer
1203686	SNOMED CT	890528009	Cancer of left lung	Cancer
1203687	SNOMED CT	890529001	Cancer of right lung	Cancer
1203688	SNOMED CT	890534002	Bilateral primary malignant neoplasm of lungs	Cancer

1203757	SNOMED CT	895111002	Chordoma of vertebral column	Cancer
1203788	SNOMED CT	895345000	Bilateral primary malignant neoplasm of kidneys	Cancer
1203797	SNOMED CT	895354002	Primary malignant neoplasm of left ureter	Cancer
1203798	SNOMED CT	895355001	Primary malignant neoplasm of right ureter	Cancer
1203799	SNOMED CT	895356000	Bilateral primary malignant neoplasm of ureters	Cancer
1203800	SNOMED CT	895358004	Primary malignant neoplasm of left testis	Cancer
1203801	SNOMED CT	895359007	Primary malignant neoplasm of right testis	Cancer
1203802	SNOMED CT	895361003	Bilateral primary malignant neoplasm of testes	Cancer
1204400	SNOMED CT	898197001	Adult T-cell leukemia/lymphoma in remission	Cancer
1204401	SNOMED CT	898198006	T-cell prolymphocytic leukemia in remission	Cancer
1208521	SNOMED CT	1.074E+16	Primary adenocarcinoma of right fallopian tube	Cancer
1208522	SNOMED CT	1.074E+16	Primary adenocarcinoma of left fallopian tube	Cancer
1208523	SNOMED CT	1.074E+16	Right ovarian primary mucinous cystadenocarcinoma	Cancer
1208524	SNOMED CT	1.074E+16	Left ovarian primary mucinous cystadenocarcinoma	Cancer
1208525	SNOMED CT	1.074E+16	Primary serous papillary cystadenocarcinoma of right ovary	Cancer
1208526	SNOMED CT	1.074E+16	Primary serous papillary cystadenocarcinoma of left ovary	Cancer
1208527	SNOMED CT	1.074E+16	Right ovarian primary endometrioid carcinoma	Cancer
1208528	SNOMED CT	1.074E+16	Left ovarian primary endometrioid carcinoma	Cancer
1208535	SNOMED CT	1.079E+15	Primary adenocarcinoma of left main bronchus	Cancer
1208536	SNOMED CT	1.079E+15	Primary adenocarcinoma of right main bronchus	Cancer
1208537	SNOMED CT	1.079E+15	Primary adenocarcinoma of middle lobe of right lung	Cancer
1208538	SNOMED CT	1.079E+15	Left lacrimal adenoid cystic carcinoma	Cancer
1208539	SNOMED CT	1.079E+15	Right lacrimal adenoid cystic carcinoma	Cancer
1208550	SNOMED CT	1.08E+15	Secondary malignant neoplasm of left kidney	Cancer

1208551	SNOMED CT	1.08E+15	Secondary malignant neoplasm of right kidney	Cancer
1208552	SNOMED CT	1.08E+15	Primary chondrosarcoma of bone of left upper limb	Cancer
1208553	SNOMED CT	1.08E+15	Primary chondrosarcoma of bone of left foot	Cancer
1208554	SNOMED CT	1.08E+15	Primary chondrosarcoma of bone of left hand	Cancer
1208555	SNOMED CT	1.08E+15	Primary chondrosarcoma of bone of left lower limb	Cancer
1208556	SNOMED CT	1.08E+15	Primary chondrosarcoma of left scapula	Cancer
1208557	SNOMED CT	1.08E+15	Primary chondrosarcoma of bone of right upper limb	Cancer
1208558	SNOMED CT	1.08E+15	Primary chondrosarcoma of bone of right foot	Cancer
1208559	SNOMED CT	1.08E+15	Primary chondrosarcoma of bone of right hand	Cancer
1208560	SNOMED CT	1.08E+15	Primary chondrosarcoma of bone of right lower limb	Cancer
1208561	SNOMED CT	1.08E+15	Primary chondrosarcoma of right scapula	Cancer
1208570	SNOMED CT	1.08E+15	Primary Ewing sarcoma of bone of left upper limb	Cancer
1208571	SNOMED CT	1.08E+15	Primary Ewing sarcoma of bone of left foot	Cancer
1208572	SNOMED CT	1.08E+15	Primary Ewing sarcoma of bone of left lower limb	Cancer
1208573	SNOMED CT	1.08E+15	Primary Ewing sarcoma of bone of right upper limb	Cancer
1208574	SNOMED CT	1.08E+15	Primary Ewing sarcoma of bone of right foot	Cancer
1208575	SNOMED CT	1.08E+15	Primary Ewing sarcoma of bone of right lower limb	Cancer
1208576	SNOMED CT	1.08E+15	Primary large cell carcinoma of lower lobe of left lung	Cancer
1208577	SNOMED CT	1.08E+15	Primary large cell carcinoma of upper lobe of left lung	Cancer
1208578	SNOMED CT	1.081E+15	Primary large cell carcinoma of lower lobe of right lung	Cancer
1208579	SNOMED CT	1.081E+15	Primary large cell carcinoma of upper lobe of right lung	Cancer
1208582	SNOMED CT	1.081E+15	Primary leiomyosarcoma of left kidney	Cancer
1208587	SNOMED CT	1.081E+15	Primary leiomyosarcoma of right kidney	Cancer
1208599	SNOMED CT	1.081E+15	Primary osteosarcoma of bone of left upper limb	Cancer
1208600	SNOMED CT	1.081E+15	Primary osteosarcoma of bone of left foot	Cancer

1208601	SNOMED CT	1.081E+15	Primary osteosarcoma of bone of left hand	Cancer
1208602	SNOMED CT	1.081E+15	Primary osteosarcoma of bone of left lower limb	Cancer
1208603	SNOMED CT	1.081E+15	Primary osteosarcoma of left scapula	Cancer
1208604	SNOMED CT	1.081E+15	Primary osteosarcoma of bone of right upper limb	Cancer
1208605	SNOMED CT	1.081E+15	Primary osteosarcoma of bone of right foot	Cancer
1208606	SNOMED CT	1.081E+15	Primary osteosarcoma of bone of right hand	Cancer
1208607	SNOMED CT	1.081E+15	Primary osteosarcoma of bone of right lower limb	Cancer
1208608	SNOMED CT	1.081E+15	Primary osteosarcoma of right scapula	Cancer
1208609	SNOMED CT	1.082E+15	Left primary retinoblastoma	Cancer
1208610	SNOMED CT	1.082E+15	Right primary retinoblastoma	Cancer
1208621	SNOMED CT	1.082E+15	Primary small cell carcinoma of lower lobe of left lung	Cancer
1208622	SNOMED CT	1.082E+15	Primary small cell carcinoma of left main bronchus	Cancer
1208623	SNOMED CT	1.082E+15	Primary small cell carcinoma of upper lobe of left lung	Cancer
1208624	SNOMED CT	1.082E+15	Primary small cell carcinoma of lower lobe of right lung	Cancer
1208625	SNOMED CT	1.082E+15	Primary small cell carcinoma of right main bronchus	Cancer
1208626	SNOMED CT	1.082E+15	Primary small cell carcinoma of middle lobe of right lung	Cancer
1208627	SNOMED CT	1.082E+15	Primary small cell carcinoma of upper lobe of right lung	Cancer
1208629	SNOMED CT	1.082E+15	Primary squamous cell carcinoma of conjunctiva of left eye	Cancer
1208631	SNOMED CT	1.082E+15	Primary squamous cell carcinoma of lower lobe of left lung	Cancer
1208632	SNOMED CT	1.082E+15	Primary squamous cell carcinoma of left main bronchus	Cancer
1208634	SNOMED CT	1.082E+15	Primary squamous cell carcinoma of upper lobe of left lung	Cancer
1208636	SNOMED CT	1.082E+15	Primary squamous cell carcinoma of conjunctiva of right eye	Cancer
1208638	SNOMED CT	1.082E+15	Primary squamous cell carcinoma of lower lobe of right lung	Cancer

1208639	SNOMED CT	1.082E+15	Primary squamous cell carcinoma of right main bronchus	Cancer
1208640	SNOMED CT	1.082E+15	Primary squamous cell carcinoma of middle lobe of right lung	Cancer
1208642	SNOMED CT	1.082E+15	Primary squamous cell carcinoma of upper lobe of right lung	Cancer
1208669	SNOMED CT	1.098E+16	Leiomyosarcoma of skin of chest	Cancer
1208670	SNOMED CT	1.098E+16	Primary primitive neuroectodermal neoplasm of central nervous system	Cancer
1208671	SNOMED CT	1.1E+15	Primary malignant neoplasm of left lacrimal sac	Cancer
1208672	SNOMED CT	1.1E+15	Primary malignant neoplasm of right lacrimal sac	Cancer
1208804	SNOMED CT	1.138E+09	Leiomyosarcoma of fundus of stomach	Cancer
1208884	SNOMED CT	1.142E+09	Carcinoma of pyriform fossa	Cancer
1208890	SNOMED CT	1.142E+09	Non-small cell lung carcinoma with NRG1 fusion	Cancer
1209337	SNOMED CT	1.144E+09	Malignant neoplasm of lateral border of tongue	Cancer
1209374	SNOMED CT	1.144E+09	Leiomyosarcoma of pylorus of stomach	Cancer
1209375	SNOMED CT	1.144E+09	Leiomyosarcoma of pyloric antrum of stomach	Cancer
1209376	SNOMED CT	1.144E+09	Leiomyosarcoma of gastric corpus structure	Cancer
1209377	SNOMED CT	1.144E+09	Leiomyosarcoma of lesser curvature of stomach	Cancer
1209378	SNOMED CT	1.144E+09	Leiomyosarcoma of greater curvature of stomach	Cancer
1209580	SNOMED CT	1.145E+09	Malignant tumor of esophagus with NRG1 fusion	Cancer
1209674	SNOMED CT	1.145E+09	Thymoma type AB	Cancer
1209676	SNOMED CT	1.145E+09	Thymoma type B	Cancer
1209680	SNOMED CT	1.145E+09	Thymoma type A	Cancer
1209696	SNOMED CT	1.145E+09	Adnexal carcinoma of vulva	Cancer
1209890	SNOMED CT	1.145E+09	Carcinoma of meibomian gland	Cancer
1210305	SNOMED CT	1.149E+09	Follicular lymphoma grade 3b	Cancer

1210307	SNOMED CT	1.149E+09	Follicular non-Hodgkin's lymphoma in situ	Cancer
1210310	SNOMED CT	1.149E+09	Follicular lymphoma grade 3a	Cancer
1210311	SNOMED CT	1.149E+09	Malignant cystic nephroma	Cancer
1210349	SNOMED CT	1.149E+09	Squamous cell carcinoma of male genital	Cancer
1210352	SNOMED CT	1.149E+09	Spermatocytic seminoma of testis	Cancer
1210353	SNOMED CT	1.149E+09	Acute myeloid leukemia with t(8;21)(q22;q22) RUNX1-RUNX1T1	Cancer
1210376	SNOMED CT	1.149E+09	Cystic renal cell carcinoma of kidney	Cancer
1210838	SNOMED CT	1.153E+09	Pediatric nodal marginal zone B cell lymphoma	Cancer
1210847	SNOMED CT	1.153E+09	Myelodysplastic syndrome with single lineage dysplasia	Cancer
1210848	SNOMED CT	1.153E+09	Primary non-Hodgkin malignant lymphoma of uveal tract	Cancer
1210849	SNOMED CT	1.153E+09	Primary non-Hodgkin malignant lymphoma of vitreoretinal tract	Cancer
1210850	SNOMED CT	1.153E+09	Primary effusion lymphoma due to human immune deficiency virus infection	Cancer
1210856	SNOMED CT	1.153E+09	Malignant lymphoma of duodenum	Cancer
1210857	SNOMED CT	1.153E+09	Malignant lymphoma of esophagus	Cancer
1210858	SNOMED CT	1.153E+09	Lymphoma of anus	Cancer
1210859	SNOMED CT	1.153E+09	Lymphoma of appendix	Cancer
1210877	SNOMED CT	1.153E+09	Mixed phenotype acute leukemia with t(9;22)(q34;q11.2); BCR-ABL1	Cancer
1210879	SNOMED CT	1.153E+09	Mixed phenotype acute leukemia with T-cell and myeloid lineage	Cancer
1210881	SNOMED CT	1.153E+09	Mixed phenotype acute leukemia with myeloid and B-cell lymphoid phenotypes	Cancer
1210882	SNOMED CT	1.153E+09	Lymphoma of lacrimal gland	Cancer
1210883	SNOMED CT	1.153E+09	Lymphoma of liver	Cancer
1210886	SNOMED CT	1.153E+09	Gastrointestinal stromal neoplasm of cardia of stomach	Cancer
1210888	SNOMED CT	1.153E+09	Gastrointestinal stromal neoplasm of body of stomach	Cancer

1210901	SNOMED CT	1.153E+09	Gastrointestinal stromal neoplasm of pyloric antrum of stomach	Cancer
1210916	SNOMED CT	1.153E+09	Chondrosarcoma of sternum	Cancer
1210917	SNOMED CT	1.153E+09	Chondrosarcoma of mandible	Cancer
1210918	SNOMED CT	1.153E+09	Chondrosarcoma of rib	Cancer
1210919	SNOMED CT	1.153E+09	Chondrosarcoma of bone of pelvic wall	Cancer
1210920	SNOMED CT	1.153E+09	Chondrosarcoma of clavicle	Cancer
1210921	SNOMED CT	1.153E+09	Chondrosarcoma of skull	Cancer
1210922	SNOMED CT	1.153E+09	Chondrosarcoma of vertebral column	Cancer
1211348	SNOMED CT	1.156E+09	Secondary malignant neoplasm of leptomeninges	Cancer
1211625	SNOMED CT	1.156E+09	Composite Hodgkin and non-Hodgkin lymphoma	Cancer
1211627	SNOMED CT	1.156E+09	Anaplastic oligodendroglioma of central nervous system	Cancer
1211628	SNOMED CT	1.156E+09	Myxopapillary ependymoma of spinal cord	Cancer
1211630	SNOMED CT	1.156E+09	Anaplastic ependymoma of central nervous system	Cancer
1211631	SNOMED CT	1.156E+09	Anaplastic oligoastrocytoma of central nervous system	Cancer
1211634	SNOMED CT	1.156E+09	Gliomatosis cerebri	Cancer
1211635	SNOMED CT	1.156E+09	Giant cell glioblastoma of brain	Cancer
1211636	SNOMED CT	1.156E+09	Protoplasmic astrocytoma of brain	Cancer
1211637	SNOMED CT	1.156E+09	Gemistocytic astrocytoma of brain	Cancer
1211638	SNOMED CT	1.156E+09	Fibrillary astrocytoma of brain	Cancer
1211655	SNOMED CT	1.156E+09	Pleomorphic xanthoastrocytoma of brain	Cancer
1211656	SNOMED CT	1.156E+09	Pilomyxoid astrocytoma of brain	Cancer
1211658	SNOMED CT	1.156E+09	Anaplastic ganglioglioma of central nervous system	Cancer
1211659	SNOMED CT	1.156E+09	Desmoplastic medulloblastoma of brain	Cancer
1211660	SNOMED CT	1.156E+09	Gliosarcoma of brain	Cancer
1211664	SNOMED CT	1.156E+09	Large cell medulloblastoma of brain	Cancer

1211666	SNOMED CT	1.156E+09	Choroid plexus carcinoma	Cancer
1211667	SNOMED CT	1.156E+09	Papillary tumor of pineal region	Cancer
1211675	SNOMED CT	1.156E+09	Dendritic cell sarcoma	Cancer
1211791	SNOMED CT	1.157E+09	Leiomyosarcoma of rectum	Cancer
1211793	SNOMED CT	1.157E+09	Leiomyosarcoma of duodenum	Cancer
1211794	SNOMED CT	1.157E+09	Leiomyosarcoma of esophagus	Cancer
1211795	SNOMED CT	1.157E+09	Leiomyosarcoma of colon	Cancer
1211802	SNOMED CT	1.157E+09	Kaposi sarcoma of colon	Cancer
1211804	SNOMED CT	1.157E+09	Kaposi sarcoma of rectum	Cancer
1211811	SNOMED CT	1.157E+09	Peripheral neuroectodermal neoplasm of corpus uteri	Cancer
1211814	SNOMED CT	1.157E+09	Peripheral neuroectodermal neoplasm of cervix uteri	Cancer
1211815	SNOMED CT	1.157E+09	Malignant mixed M√ºllerian neoplasm of corpus uteri	Cancer
1211816	SNOMED CT	1.157E+09	Malignant mesenchymal neoplasm of duodenum	Cancer
1211817	SNOMED CT	1.157E+09	Malignant mesenchymal neoplasm of esophagus	Cancer
1211818	SNOMED CT	1.157E+09	Malignant mesenchymal neoplasm of anus	Cancer
1211819	SNOMED CT	1.157E+09	Malignant mesenchymal neoplasm of appendix	Cancer
1211833	SNOMED CT	1.157E+09	Germinoma of central nervous system	Cancer
1212007	SNOMED CT	1.157E+09	Diffuse astrocytoma of brain	Cancer
1212008	SNOMED CT	1.157E+09	Gliosarcoma of central nervous system	Cancer
1212009	SNOMED CT	1.157E+09	Giant cell glioblastoma of central nervous system	Cancer
1212011	SNOMED CT	1.157E+09	Gemistocytic astrocytoma of central nervous system	Cancer
1212014	SNOMED CT	1.157E+09	Malignant neoplasm of vertebra	Cancer
1212015	SNOMED CT	1.157E+09	Fibrillary astrocytoma of central nervous system	Cancer
1212018	SNOMED CT	1.157E+09	Gliosarcoma of spinal cord	Cancer
1212042	SNOMED CT	1.157E+09	Astroblastoma of central nervous system	Cancer

1212053	SNOMED CT	1.157E+09	Acute myeloid leukemia with 11q23 abnormality	Cancer
1212058	SNOMED CT	1.157E+09	Intravascular large B-cell lymphoma	Cancer
1212135	SNOMED CT	1.157E+09	Invasive benign pituitary adenoma	Cancer
1212311	SNOMED CT	1.224E+16	Secondary malignant neoplasm of left breast	Cancer
1212312	SNOMED CT	1.224E+16	Secondary malignant neoplasm of right breast	Cancer
1212313	SNOMED CT	1.225E+16	Bilateral secondary malignant neoplasm of kidneys	Cancer
1212314	SNOMED CT	1.225E+16	Bilateral secondary malignant neoplasm of breasts	Cancer
1212320	SNOMED CT	1.309E+14	Primary chordoma of bone of skull	Cancer
1212328	SNOMED CT	1.455E+14	Secondary malignant neoplasm of breast	Cancer
1212389	SNOMED CT	1.593E+16	Bilateral secondary malignant neoplasm of ovaries	Cancer
1212390	SNOMED CT	1.593E+16	Left ovarian primary sarcoma	Cancer
1212391	SNOMED CT	1.593E+16	Right ovarian primary sarcoma	Cancer
1212393	SNOMED CT	1.595E+16	Primary squamous cell carcinoma of skin of left breast	Cancer
1212394	SNOMED CT	1.595E+16	Primary squamous cell carcinoma of skin of right breast	Cancer
1212395	SNOMED CT	1.595E+16	Primary malignant neoplasm of skin of right breast	Cancer
1212396	SNOMED CT	1.595E+16	Primary basal cell carcinoma of skin of right breast	Cancer
1212397	SNOMED CT	1.595E+16	Primary basal cell carcinoma of skin of left breast	Cancer
1212398	SNOMED CT	1.595E+16	Primary malignant neoplasm of skin of left breast	Cancer
1212399	SNOMED CT	1.595E+16	Bilateral malignant melanoma of skin of lower limbs	Cancer
1212400	SNOMED CT	1.595E+16	Bilateral malignant melanoma of skin of upper limbs	Cancer
1212407	SNOMED CT	1.596E+16	Primary malignant neoplasm of right greater vestibular gland	Cancer
1212408	SNOMED CT	1.596E+16	Primary malignant neoplasm of left greater vestibular gland	Cancer
1212409	SNOMED CT	1.596E+16	Primary small cell carcinoma of right lung	Cancer
1212410	SNOMED CT	1.596E+16	Primary small cell carcinoma of left lung	Cancer
1212411	SNOMED CT	1.596E+16	Secondary adenocarcinoma of right lung	Cancer

1212412	SNOMED CT	1.596E+16	Secondary adenocarcinoma of left lung	Cancer
1212413	SNOMED CT	1.596E+16	Secondary small cell carcinoma of right lung	Cancer
1212414	SNOMED CT	1.596E+16	Secondary small cell carcinoma of left lung	Cancer
1212415	SNOMED CT	1.596E+16	Bilateral secondary small cell carcinoma of lungs	Cancer
1212416	SNOMED CT	1.596E+16	Bilateral secondary squamous cell carcinoma of lungs	Cancer
1212417	SNOMED CT	1.596E+16	Secondary squamous cell carcinoma of right lung	Cancer
1212418	SNOMED CT	1.596E+16	Secondary squamous cell carcinoma of left lung	Cancer
1212419	SNOMED CT	1.596E+16	Bilateral Kaposi sarcoma of lungs	Cancer
1212421	SNOMED CT	1.596E+16	Primary sarcoma of left kidney	Cancer
1212422	SNOMED CT	1.596E+16	Primary sarcoma of right kidney	Cancer
1212423	SNOMED CT	1.596E+16	Bilateral secondary adenocarcinoma of kidneys	Cancer
1212424	SNOMED CT	1.596E+16	Secondary adenocarcinoma of left kidney	Cancer
1212425	SNOMED CT	1.596E+16	Secondary adenocarcinoma of right kidney	Cancer
1212426	SNOMED CT	1.596E+16	Secondary squamous cell carcinoma of right kidney	Cancer
1212427	SNOMED CT	1.596E+16	Secondary squamous cell carcinoma of left kidney	Cancer
1212428	SNOMED CT	1.596E+16	Secondary malignant melanoma of left kidney	Cancer
1212429	SNOMED CT	1.596E+16	Bilateral secondary malignant melanoma of kidneys	Cancer
1212430	SNOMED CT	1.596E+16	Secondary malignant melanoma of right kidney	Cancer
1212439	SNOMED CT	1.598E+16	Bilateral secondary malignant neoplasm of eyes	Cancer
1212440	SNOMED CT	1.598E+16	Secondary malignant neoplasm of right eye	Cancer
1212441	SNOMED CT	1.598E+16	Secondary malignant neoplasm of left eye	Cancer
1212463	SNOMED CT	1.609E+16	Primary angiosarcoma of skin of lip	Cancer
1212468	SNOMED CT	1.622E+16	Primary squamous cell carcinoma of skin of lip	Cancer
1212477	SNOMED CT	1.653E+16	Primary non-small cell carcinoma of lower lobe of left lung	Cancer
1212478	SNOMED CT	1.653E+16	Primary non-small cell carcinoma of middle lobe of right lung	Cancer

1212479	SNOMED CT	1.653E+16	Primary non-small cell carcinoma of upper lobe of left lung	Cancer
1212480	SNOMED CT	1.653E+16	Primary non-small cell carcinoma of lower lobe of right lung	Cancer
1212481	SNOMED CT	1.653E+16	Primary non-small cell carcinoma of right lung	Cancer
1212482	SNOMED CT	1.653E+16	Primary non-small cell carcinoma of left lung	Cancer
1212483	SNOMED CT	1.653E+16	Primary non-small cell carcinoma of upper lobe of right lung	Cancer
1212487	SNOMED CT	1.664E+16	Primary malignant gastrointestinal stromal neoplasm of colon	Cancer
1212488	SNOMED CT	1.664E+16	Primary malignant gastrointestinal stromal neoplasm of rectum	Cancer
1212497	SNOMED CT	1.677E+16	Primary basal cell carcinoma of skin of labium majus	Cancer
1212498	SNOMED CT	1.678E+16	Mycosis fungoides of facial lymph node	Cancer
1212502	SNOMED CT	1.685E+16	Primary glioblastoma multiforme of cerebellum	Cancer
1212503	SNOMED CT	1.685E+16	Primary glioblastoma multiforme of brainstem	Cancer
1212504	SNOMED CT	1.685E+16	Primary chondrosarcoma of sternum	Cancer
1212506	SNOMED CT	1.69E+16	Primary squamous cell carcinoma of skin of anus	Cancer
1212507	SNOMED CT	1.691E+16	Primary malignant neoplasm of skin of right upper eyelid	Cancer
1212508	SNOMED CT	1.691E+16	Primary malignant neoplasm of skin of right lower eyelid	Cancer
1212509	SNOMED CT	1.691E+16	Primary basal cell carcinoma of left upper eyelid	Cancer
1212510	SNOMED CT	1.691E+16	Primary basal cell carcinoma of right upper eyelid	Cancer
1212511	SNOMED CT	1.691E+16	Primary basal cell carcinoma of right lower eyelid	Cancer
1212512	SNOMED CT	1.691E+16	Primary basal cell carcinoma of left lower eyelid	Cancer
1212513	SNOMED CT	1.691E+16	Primary malignant neoplasm of skin of left lower eyelid	Cancer
1212514	SNOMED CT	1.691E+16	Primary malignant neoplasm of skin of left upper eyelid	Cancer
1212515	SNOMED CT	1.691E+16	Primary squamous cell carcinoma of right upper eyelid	Cancer
1212516	SNOMED CT	1.691E+16	Primary squamous cell carcinoma of right lower eyelid	Cancer
1212517	SNOMED CT	1.691E+16	Primary squamous cell carcinoma of left upper eyelid	Cancer

1212518	SNOMED CT	1.691E+16	Primary squamous cell carcinoma of left lower eyelid	Cancer
1212523	SNOMED CT	1.691E+16	Squamous cell carcinoma in situ of skin of right lower eyelid	Cancer
1212524	SNOMED CT	1.691E+16	Squamous cell carcinoma in situ of skin of left lower eyelid	Cancer
1212545	SNOMED CT	2.44E+17	Malignant melanoma of skin of right ankle	Cancer
1212546	SNOMED CT	2.494E+17	Malignant melanoma of skin of right wrist	Cancer
1212651	SNOMED CT	3.507E+14	Primary malignant neoplasm of adrenal medulla of right adrenal gland	Cancer
1212655	SNOMED CT	3.508E+14	Primary malignant neoplasm of left adrenal cortex	Cancer
1212657	SNOMED CT	3.509E+14	Primary malignant neoplasm of adrenal medulla of left adrenal gland	Cancer
1212658	SNOMED CT	3.509E+14	Primary malignant neoplasm of right adrenal cortex	Cancer
1212662	SNOMED CT	3.513E+14	Acute myelofibrosis in remission	Cancer
1212666	SNOMED CT	3.523E+14	Primary malignant neoplasm of left spermatic cord	Cancer
1212667	SNOMED CT	3.523E+14	Primary malignant neoplasm of right spermatic cord	Cancer
1212679	SNOMED CT	3.529E+14	Primary malignant neoplasm of left optic nerve	Cancer
1212680	SNOMED CT	3.529E+14	Primary malignant neoplasm of right optic nerve	Cancer
1212681	SNOMED CT	3.53E+14	Primary malignant neoplasm of right renal pelvis	Cancer
1212682	SNOMED CT	3.531E+14	Right retinal primary malignant neoplasm	Cancer
1212683	SNOMED CT	3.531E+14	Left retinal primary malignant neoplasm	Cancer
1212684	SNOMED CT	3.531E+14	Primary malignant neoplasm of left renal pelvis	Cancer
1212685	SNOMED CT	3.536E+14	Secondary malignant neoplasm of right adrenal gland	Cancer
1212688	SNOMED CT	3.537E+14	Secondary malignant neoplasm of left adrenal gland	Cancer
1212689	SNOMED CT	3.538E+14	Left corneal primary malignant neoplasm	Cancer
1212690	SNOMED CT	3.538E+14	Right conjunctival primary malignant neoplasm	Cancer
1212691	SNOMED CT	3.538E+14	Right choroidal primary malignant neoplasm	Cancer
1212692	SNOMED CT	3.538E+14	Right corneal primary malignant neoplasm	Cancer

1212693	SNOMED CT	3.538E+14	Left conjunctival primary malignant neoplasm	Cancer
1212695	SNOMED CT	3.543E+14	Left choroidal primary malignant neoplasm	Cancer
1212697	SNOMED CT	3.544E+14	Kaposi sarcoma of left lung	Cancer
1212699	SNOMED CT	3.545E+14	Kaposi sarcoma of right lung	Cancer
1212700	SNOMED CT	3.547E+14	Primary malignant neoplasm of left main bronchus	Cancer
1212701	SNOMED CT	3.547E+14	Primary malignant neoplasm of right main bronchus	Cancer
1212713	SNOMED CT	4.004E+17	Malignant melanoma of skin of left ankle	Cancer
1213226	SNOMED CT	6.816E+14	Primary adenocarcinoma of anterior wall of urinary bladder	Cancer
1213227	SNOMED CT	6.816E+14	Primary adenocarcinoma of neck of urinary bladder	Cancer
1213228	SNOMED CT	6.817E+14	Primary adenocarcinoma of dome of urinary bladder	Cancer
1213229	SNOMED CT	6.817E+14	Primary adenocarcinoma of fundus of stomach	Cancer
1213231	SNOMED CT	6.817E+14	Primary adenocarcinoma of greater curvature of stomach	Cancer
1213234	SNOMED CT	6.818E+14	Primary adenocarcinoma of lesser curvature of stomach	Cancer
1213238	SNOMED CT	6.819E+14	Primary adenocarcinoma of pylorus	Cancer
1213240	SNOMED CT	6.82E+14	Primary adenocarcinoma of trigone of urinary bladder	Cancer
1213241	SNOMED CT	6.82E+14	Primary adenocarcinoma of urachus	Cancer
1213242	SNOMED CT	6.82E+14	Primary adenocarcinoma of ureteral orifice	Cancer
1213243	SNOMED CT	6.822E+14	Primary anaplastic astrocytoma of cerebrum	Cancer
1213244	SNOMED CT	6.822E+14	Primary anaplastic astrocytoma of frontal lobe	Cancer
1213245	SNOMED CT	6.822E+14	Primary anaplastic astrocytoma of occipital lobe	Cancer
1213246	SNOMED CT	6.823E+14	Primary anaplastic astrocytoma of parietal lobe	Cancer
1213247	SNOMED CT	6.823E+14	Primary anaplastic astrocytoma of temporal lobe	Cancer
1213249	SNOMED CT	6.823E+14	Primary angiosarcoma of axillary region	Cancer
1213254	SNOMED CT	6.824E+14	Primary angiosarcoma of thorax	Cancer
1213256	SNOMED CT	6.825E+14	Primary astrocytoma of frontal lobe	Cancer

1213257	SNOMED CT	6.825E+14	Primary astrocytoma of parietal lobe	Cancer
1213258	SNOMED CT	6.825E+14	Primary astrocytoma of temporal lobe	Cancer
1213262	SNOMED CT	6.83E+14	Primary carcinoma ex pleomorphic adenoma of salivary gland	Cancer
1213263	SNOMED CT	6.83E+14	Primary carcinoma ex pleomorphic adenoma of submandibular gland	Cancer
1213265	SNOMED CT	6.831E+14	Primary chondrosarcoma of mandible	Cancer
1213266	SNOMED CT	6.831E+14	Primary chondrosarcoma of vertebral column	Cancer
1213270	SNOMED CT	6.838E+14	Primary ependymoma of brain ventricle	Cancer
1213271	SNOMED CT	6.838E+14	Primary ependymoma of parietal lobe	Cancer
1213273	SNOMED CT	6.84E+14	Primary Ewing sarcoma of bone of spine	Cancer
1213274	SNOMED CT	6.849E+14	Primary glioblastoma multiforme of cerebrum	Cancer
1213275	SNOMED CT	6.849E+14	Primary glioblastoma multiforme of occipital lobe	Cancer
1213276	SNOMED CT	6.849E+14	Primary glioblastoma multiforme of parietal lobe	Cancer
1213277	SNOMED CT	6.849E+14	Primary glioblastoma multiforme of temporal lobe	Cancer
1213286	SNOMED CT	6.857E+14	Primary leiomyosarcoma of thorax	Cancer
1213289	SNOMED CT	6.859E+14	Primary liposarcoma of soft tissue of axilla	Cancer
1213295	SNOMED CT	6.86E+14	Primary liposarcoma of soft tissue of thorax	Cancer
1213297	SNOMED CT	6.864E+14	Primary malignant glioma of frontal lobe	Cancer
1213298	SNOMED CT	6.864E+14	Primary malignant glioma of occipital lobe	Cancer
1213299	SNOMED CT	6.865E+14	Primary malignant glioma of parietal lobe	Cancer
1213300	SNOMED CT	6.865E+14	Primary malignant glioma of temporal lobe	Cancer
1213301	SNOMED CT	6.866E+14	Primary malignant pheochromocytoma of left adrenal gland	Cancer
1213302	SNOMED CT	6.866E+14	Primary malignant pheochromocytoma of right adrenal gland	Cancer
1213303	SNOMED CT	6.87E+14	Mycosis fungoides of inguinal lymph node	Cancer

1213304	SNOMED CT	6.87E+14	Mycosis fungoides of lower limb lymph node	Cancer
1213306	SNOMED CT	6.873E+14	Primary oligodendroglioma of frontal lobe	Cancer
1213307	SNOMED CT	6.874E+14	Primary oligodendroglioma of parietal lobe	Cancer
1213308	SNOMED CT	6.874E+14	Primary oligodendroglioma of temporal lobe	Cancer
1213309	SNOMED CT	6.874E+14	Primary osteosarcoma of mandible	Cancer
1213310	SNOMED CT	6.874E+14	Primary osteosarcoma of vertebral column	Cancer
1213319	SNOMED CT	6.879E+14	Primary rhabdomyosarcoma of thorax	Cancer
1213321	SNOMED CT	6.88E+14	Primary sarcoma of axilla	Cancer
1213327	SNOMED CT	6.881E+14	Primary sarcoma of thorax	Cancer
1213332	SNOMED CT	6.887E+14	Primary squamous cell carcinoma of anterior floor of mouth	Cancer
1213333	SNOMED CT	6.887E+14	Primary squamous cell carcinoma of body of tongue	Cancer
1213334	SNOMED CT	6.887E+14	Primary squamous cell carcinoma of anterior wall of urinary bladder	Cancer
1213335	SNOMED CT	6.887E+14	Primary squamous cell carcinoma of aryepiglottic fold	Cancer
1213336	SNOMED CT	6.887E+14	Primary squamous cell carcinoma of neck of urinary bladder	Cancer
1213337	SNOMED CT	6.888E+14	Primary squamous cell carcinoma of border of tongue	Cancer
1213338	SNOMED CT	6.888E+14	Primary squamous cell carcinoma of commissure of lip	Cancer
1213339	SNOMED CT	6.888E+14	Primary squamous cell carcinoma of dome of urinary bladder	Cancer
1213340	SNOMED CT	6.888E+14	Primary squamous cell carcinoma of dorsal surface of tongue	Cancer
1213341	SNOMED CT	6.889E+14	Primary squamous cell carcinoma of hard palate	Cancer
1213342	SNOMED CT	6.889E+14	Primary squamous cell carcinoma of mucous membrane of lip	Cancer
1213343	SNOMED CT	6.889E+14	Primary squamous cell carcinoma of mucous membrane of lower lip	Cancer

1213344	SNOMED CT	6.889E+14	Primary squamous cell carcinoma of mucous membrane of upper lip	Cancer
1213345	SNOMED CT	6.889E+14	Primary squamous cell carcinoma of lateral floor of mouth	Cancer
1213346	SNOMED CT	6.889E+14	Primary squamous cell carcinoma of lateral wall of urinary bladder	Cancer
1213347	SNOMED CT	6.89E+14	Primary squamous cell carcinoma of lower gum	Cancer
1213349	SNOMED CT	6.892E+14	Primary squamous cell carcinoma of posterior wall of urinary bladder	Cancer
1213350	SNOMED CT	6.893E+14	Primary squamous cell carcinoma of posterior wall of nasopharynx	Cancer
1213351	SNOMED CT	6.893E+14	Primary squamous cell carcinoma of retromolar area	Cancer
1213352	SNOMED CT	6.893E+14	Primary squamous cell carcinoma of soft palate	Cancer
1213353	SNOMED CT	6.894E+14	Primary squamous cell carcinoma of tonsillar fossa	Cancer
1213354	SNOMED CT	6.894E+14	Primary squamous cell carcinoma of tonsillar pillar	Cancer
1213355	SNOMED CT	6.894E+14	Primary squamous cell carcinoma of upper gum	Cancer
1213356	SNOMED CT	6.894E+14	Primary squamous cell carcinoma of ureteral orifice	Cancer
1213357	SNOMED CT	6.895E+14	Primary squamous cell carcinoma of uvula	Cancer
1213358	SNOMED CT	6.895E+14	Primary squamous cell carcinoma of inferior surface of tongue	Cancer
1213359	SNOMED CT	6.895E+14	Primary squamous cell carcinoma of vermilion border of lower lip	Cancer
1213360	SNOMED CT	6.895E+14	Primary squamous cell carcinoma of vermilion border of upper lip	Cancer
1213361	SNOMED CT	6.895E+14	Primary squamous cell carcinoma of vestibule of mouth	Cancer
1213362	SNOMED CT	6.898E+14	Primary transitional cell carcinoma of anterior wall of urinary bladder	Cancer
1213363	SNOMED CT	6.898E+14	Primary transitional cell carcinoma of neck of urinary bladder	Cancer
1213364	SNOMED CT	6.898E+14	Primary transitional cell carcinoma of dome of urinary bladder	Cancer

1213365	SNOMED CT	6.898E+14	Primary transitional cell carcinoma of lateral wall of urinary bladder	Cancer
1213366	SNOMED CT	6.898E+14	Primary transitional cell carcinoma of posterior wall of urinary bladder	Cancer
1213367	SNOMED CT	6.898E+14	Primary transitional cell carcinoma of trigone of urinary bladder	Cancer
1213368	SNOMED CT	6.899E+14	Primary transitional cell carcinoma of ureteral orifice	Cancer
1213369	SNOMED CT	6.901E+14	Primary basal cell carcinoma of skin of scrotum	Cancer
1213370	SNOMED CT	6.901E+14	Primary basal cell carcinoma of skin of male genitalia	Cancer
1213385	SNOMED CT	7.631E+17	Primary renal cell carcinoma of left kidney	Cancer
1213395	SNOMED CT	8.086E+17	Malignant melanoma of skin of left forearm	Cancer
1213398	SNOMED CT	8.482E+13	Primary follicular dendritic cell sarcoma of spleen	Cancer
1213407	SNOMED CT	8.714E+13	Primary malignant neoplasm of intramedullary spinal cord	Cancer
1213410	SNOMED CT	8.942E+17	Primary renal cell carcinoma of right kidney	Cancer
1213440	SNOMED CT	9.119E+13	Secondary adenocarcinoma of pleura	Cancer
1213441	SNOMED CT	9.121E+13	Secondary small cell carcinoma of pleura	Cancer
1213442	SNOMED CT	9.122E+13	Secondary malignant melanoma of pleura	Cancer
1213444	SNOMED CT	9.127E+13	Secondary squamous cell carcinoma of bone	Cancer
1213445	SNOMED CT	9.396E+17	Malignant melanoma of skin of right forearm	Cancer
1213452	SNOMED CT	9.854E+17	Malignant melanoma of skin of left wrist	Cancer
2931751	SNOMED CT	1.217E+09	RELA fusion-positive supratentorial ependymoma	Cancer
19005	SNOMED CT	20224008	Delta heavy chain disease	Cancer
23569	SNOMED CT	25050002	Alpha heavy chain disease, respiratory form	Cancer
37494	SNOMED CT	39795003	Hand-Sch√ºller-Christian disease	Cancer
57084	SNOMED CT	60620005	Epsilon heavy chain disease	Cancer
57906	SNOMED CT	61493004	Mu heavy chain disease	Cancer

2639023	CPT	37186	Secondary percutaneous transluminal thrombectomy (eg, nonprimary mechanical, snare basket, suction technique), noncoronary, non-intracranial, arterial or arterial bypass graft, including fluoroscopic guidance and intraprocedural pharmacological thrombolytic injections, provided in conjunction with another percutaneous intervention other than primary mechanical thrombectomy (List separately in addition to code for primary procedure)	Catheter Directed Thrombectomy
2636954	CPT	37188	Percutaneous transluminal mechanical thrombectomy, vein(s), including intraprocedural pharmacological thrombolytic injections and fluoroscopic guidance, repeat treatment on subsequent day during course of thrombolytic therapy	Catheter Directed Thrombectomy
2630156	CPT	37184	Primary percutaneous transluminal mechanical thrombectomy, noncoronary, non-intracranial, arterial or arterial bypass graft, including fluoroscopic guidance and intraprocedural pharmacological thrombolytic injection(s); initial vessel	Catheter Directed Thrombectomy
2630381	CPT	37185	Primary percutaneous transluminal mechanical thrombectomy, noncoronary, non-intracranial, arterial or arterial bypass graft, including fluoroscopic guidance and intraprocedural pharmacological thrombolytic injection(s); second and all subsequent vessel(s) within the same vascular family (List separately in addition to code for primary mechanical thrombectomy procedure)	Catheter Directed Thrombectomy
2630396	CPT	37212	Transcatheter therapy, venous infusion for thrombolysis, any method, including radiological supervision and interpretation, initial treatment day	Catheter Directed Thrombectomy

2630177	CPT	37211	Transcatheter therapy, arterial infusion for thrombolysis other than coronary or intracranial, any method, including radiological supervision and interpretation, initial treatment day	Catheter Directed Thrombectomy
2635962	CPT	37213	Transcatheter therapy, arterial or venous infusion for thrombolysis other than coronary, any method, including radiological supervision and interpretation, continued treatment on subsequent day during course of thrombolytic therapy, including follow-up catheter contrast injection, position change, or exchange, when performed	Catheter Directed Thrombectomy
2631543	CPT	37187	Percutaneous transluminal mechanical thrombectomy, vein(s), including intraprocedural pharmacological thrombolytic injections and fluoroscopic guidance	Catheter Directed Thrombectomy
2636029	CPT	37214	Transcatheter therapy, arterial or venous infusion for thrombolysis other than coronary, any method, including radiological supervision and interpretation, continued treatment on subsequent day during course of thrombolytic therapy, including follow-up catheter contrast injection, position change, or exchange, when performed; cessation of thrombolysis including removal of catheter and vessel closure by any method	Catheter Directed Thrombectomy
1204944	ICD10CM	N18.30	Chronic kidney disease, stage 3 unspecified	Coronary Artery Disease
1204945	ICD10CM	N18.31	Chronic kidney disease, stage 3a	Coronary Artery Disease
1204946	ICD10CM	N18.32	Chronic kidney disease, stage 3b	Coronary Artery Disease
520764	ICD10CM	D63.1	Anemia in chronic kidney disease	Coronary Artery Disease
521049	ICD10CM	E08.22	Diabetes mellitus due to underlying condition with diabetic chronic kidney disease	Coronary Artery Disease
521166	ICD10CM	E09.22	Drug or chemical induced diabetes mellitus with diabetic chronic kidney disease	Coronary Artery Disease

521514	ICD10CM	E13.22	Other specified diabetes mellitus with diabetic chronic kidney disease	Coronary Artery Disease
528298	ICD10CM	I12	Hypertensive chronic kidney disease	Coronary Artery Disease
528299	ICD10CM	I12.0	Hypertensive chronic kidney disease with stage 5 chronic kidney disease or end stage renal disease	Coronary Artery Disease
528300	ICD10CM	I12.9	Hypertensive chronic kidney disease with stage 1 through stage 4 chronic kidney disease, or unspecified chronic kidney disease	Coronary Artery Disease
528301	ICD10CM	I13	Hypertensive heart and chronic kidney disease	Coronary Artery Disease
528302	ICD10CM	I13.0	Hypertensive heart and chronic kidney disease with heart failure and stage 1 through stage 4 chronic kidney disease, or unspecified chronic kidney disease	Coronary Artery Disease
528303	ICD10CM	I13.1	Hypertensive heart and chronic kidney disease without heart failure	Coronary Artery Disease
528304	ICD10CM	I13.10	Hypertensive heart and chronic kidney disease without heart failure, with stage 1 through stage 4 chronic kidney disease, or unspecified chronic kidney disease	Coronary Artery Disease
528305	ICD10CM	I13.11	Hypertensive heart and chronic kidney disease without heart failure, with stage 5 chronic kidney disease, or end stage renal disease	Coronary Artery Disease
528306	ICD10CM	I13.2	Hypertensive heart and chronic kidney disease with heart failure and with stage 5 chronic kidney disease, or end stage renal disease	Coronary Artery Disease
541187	ICD10CM	N18	Chronic kidney disease (CKD)	Coronary Artery Disease
541188	ICD10CM	N18.1	Chronic kidney disease, stage 1	Coronary Artery Disease
541189	ICD10CM	N18.2	Chronic kidney disease, stage 2 (mild)	Coronary Artery Disease
541190	ICD10CM	N18.3	Chronic kidney disease, stage 3 (moderate)	Coronary Artery Disease
541191	ICD10CM	N18.4	Chronic kidney disease, stage 4 (severe)	Coronary Artery Disease
541192	ICD10CM	N18.5	Chronic kidney disease, stage 5	Coronary Artery Disease
541193	ICD10CM	N18.6	End stage renal disease	Coronary Artery Disease

541194	ICD10CM	N18.9	Chronic kidney disease, unspecified	Coronary Artery Disease
542111	ICD10CM	O10.211	Pre-existing hypertensive chronic kidney disease complicating pregnancy, first trimester	Coronary Artery Disease
542112	ICD10CM	O10.212	Pre-existing hypertensive chronic kidney disease complicating pregnancy, second trimester	Coronary Artery Disease
542113	ICD10CM	O10.213	Pre-existing hypertensive chronic kidney disease complicating pregnancy, third trimester	Coronary Artery Disease
542114	ICD10CM	O10.219	Pre-existing hypertensive chronic kidney disease complicating pregnancy, unspecified trimester	Coronary Artery Disease
542115	ICD10CM	O10.22	Pre-existing hypertensive chronic kidney disease complicating childbirth	Coronary Artery Disease
542118	ICD10CM	O10.31	Pre-existing hypertensive heart and chronic kidney disease complicating pregnancy	Coronary Artery Disease
542119	ICD10CM	O10.311	Pre-existing hypertensive heart and chronic kidney disease complicating pregnancy, first trimester	Coronary Artery Disease
542120	ICD10CM	O10.312	Pre-existing hypertensive heart and chronic kidney disease complicating pregnancy, second trimester	Coronary Artery Disease
542121	ICD10CM	O10.313	Pre-existing hypertensive heart and chronic kidney disease complicating pregnancy, third trimester	Coronary Artery Disease
542122	ICD10CM	O10.319	Pre-existing hypertensive heart and chronic kidney disease complicating pregnancy, unspecified trimester	Coronary Artery Disease
542123	ICD10CM	O10.32	Pre-existing hypertensive heart and chronic kidney disease complicating childbirth	Coronary Artery Disease
542124	ICD10CM	O10.33	Pre-existing hypertensive heart and chronic kidney disease complicating the puerperium	Coronary Artery Disease
545746	ICD10CM	Q61.2	Polycystic kidney, adult type	Coronary Artery Disease
545747	ICD10CM	Q61.3	Polycystic kidney, unspecified	Coronary Artery Disease
545750	ICD10CM	Q61.8	Other cystic kidney diseases	Coronary Artery Disease
613290	ICD10CM	Z94.0	Kidney transplant status	Coronary Artery Disease

530251	ICD10CM	J44.0	Chronic obstructive pulmonary disease with (acute) lower respiratory infection	Chronic Obstructive Pulmonary Disease
530252	ICD10CM	J44.1	Chronic obstructive pulmonary disease with (acute) exacerbation	Chronic Obstructive Pulmonary Disease
530253	ICD10CM	J44.9	Chronic obstructive pulmonary disease, unspecified	Chronic Obstructive Pulmonary Disease
1176055	ICD9CM	493.2	Chronic obstructive asthma, unspecified	Chronic Obstructive Pulmonary Disease
1176056	ICD9CM	493.21	Chronic obstructive asthma with status asthmaticus	Chronic Obstructive Pulmonary Disease
1176057	ICD9CM	493.22	Chronic obstructive asthma with (acute) exacerbation	Chronic Obstructive Pulmonary Disease
1176079	ICD9CM	496	Chronic airway obstruction, not elsewhere classified	Chronic Obstructive Pulmonary Disease
292274	SNOMED CT	313296004	Mild chronic obstructive pulmonary disease	Chronic Obstructive Pulmonary Disease
292275	SNOMED CT	313297008	Moderate chronic obstructive pulmonary disease	Chronic Obstructive Pulmonary Disease
292277	SNOMED CT	313299006	Severe chronic obstructive pulmonary disease	Chronic Obstructive Pulmonary Disease
12781	SNOMED CT	13645005	Chronic obstructive lung disease	Chronic Obstructive Pulmonary Disease
120270	SNOMED CT	135836000	End stage chronic obstructive airways disease	Chronic Obstructive Pulmonary Disease
265395	SNOMED CT	285381006	Acute infective exacerbation of chronic obstructive airways disease	Chronic Obstructive Pulmonary Disease
178595	SNOMED CT	195951007	Acute exacerbation of chronic obstructive airways disease	Chronic Obstructive Pulmonary Disease
178645	SNOMED CT	196001008	Chronic obstructive pulmonary disease with acute lower respiratory infection	Chronic Obstructive Pulmonary Disease
396822	SNOMED CT	708030004	Pulmonary emphysema co-occurrent with fibrosis of lung	Chronic Obstructive Pulmonary Disease
422688	SNOMED CT	762618008	Bronchiolitis obliterans syndrome due to and after lung transplantation	Chronic Obstructive Pulmonary Disease
444755	SNOMED CT	836477007	Chronic emphysema due to vapor	Chronic Obstructive Pulmonary Disease
445075	SNOMED CT	840350008	Chronic obliterative bronchiolitis due to chemical fumes	Chronic Obstructive Pulmonary Disease
445076	SNOMED CT	840351007	Chronic obliterative bronchiolitis due to vapor	Chronic Obstructive Pulmonary Disease
447915	SNOMED CT	1.751E+12	Acute exacerbation of chronic obstructive airways disease with asthma	Chronic Obstructive Pulmonary Disease
451279	SNOMED CT	1.06E+14	Chronic obstructive lung disease co-occurrent with acute bronchitis	Chronic Obstructive Pulmonary Disease

459765	SNOMED CT	1.069E+16	Asthma-chronic obstructive pulmonary disease overlap syndrome	Chronic Obstructive Pulmonary Disease
501367	SNOMED CT	233674008	Pulmonary emphysema in alpha-1 primary immunodeficiency deficiency	Chronic Obstructive Pulmonary Disease
2645559	SNOMED CT	1.177E+09	Bronchiolitis obliterans syndrome due to and following allogeneic stem cell transplant	Chronic Obstructive Pulmonary Disease
66635	SNOMED CT	70756004	Bronchial atresia with segmental pulmonary emphysema	Chronic Obstructive Pulmonary Disease
73155	SNOMED CT	77690003	Interstitial emphysema of lung	Chronic Obstructive Pulmonary Disease
81627	SNOMED CT	86680006	Ruptured emphysematous bleb of lung	Chronic Obstructive Pulmonary Disease
82335	SNOMED CT	87433001	Pulmonary emphysema	Chronic Obstructive Pulmonary Disease
4602	SNOMED CT	4981000	Panacinar emphysema	Chronic Obstructive Pulmonary Disease
15002	SNOMED CT	16003001	Giant bullous emphysema	Chronic Obstructive Pulmonary Disease
215843	SNOMED CT	233675009	Toxic emphysema	Chronic Obstructive Pulmonary Disease
215845	SNOMED CT	233677001	Scar emphysema	Chronic Obstructive Pulmonary Disease
247531	SNOMED CT	266355005	Bullous emphysema with collapse	Chronic Obstructive Pulmonary Disease
247532	SNOMED CT	266356006	Atrophic (senile) emphysema	Chronic Obstructive Pulmonary Disease
178601	SNOMED CT	195957006	Chronic bullous emphysema	Chronic Obstructive Pulmonary Disease
178602	SNOMED CT	195958001	Segmental bullous emphysema	Chronic Obstructive Pulmonary Disease
178603	SNOMED CT	195959009	Zonal bullous emphysema	Chronic Obstructive Pulmonary Disease
178669	SNOMED CT	196026004	Chronic emphysema due to chemical fumes	Chronic Obstructive Pulmonary Disease
1201025	SNOMED CT	1.01E+09	Emphysema of left lung	Chronic Obstructive Pulmonary Disease
1201026	SNOMED CT	1.01E+09	Emphysema of right lung	Chronic Obstructive Pulmonary Disease
15806	SNOMED CT	16846004	Obstructive emphysema	Chronic Obstructive Pulmonary Disease
22527	SNOMED CT	23958009	Vanishing lung	Chronic Obstructive Pulmonary Disease
30018	SNOMED CT	31898008	Paraseptal emphysema	Chronic Obstructive Pulmonary Disease
31368	SNOMED CT	33325001	Compensatory emphysema	Chronic Obstructive Pulmonary Disease
45126	SNOMED CT	47895001	Congenital emphysema	Chronic Obstructive Pulmonary Disease

45165	SNOMED CT	47938003	Chronic obliterative bronchiolitis	Chronic Obstructive Pulmonary Disease
54330	SNOMED CT	57686001	Emphysematous bleb of lung	Chronic Obstructive Pulmonary Disease
57258	SNOMED CT	60805002	Hemolytic anemia with emphysema AND cutis laxa	Chronic Obstructive Pulmonary Disease
63084	SNOMED CT	66987001	Congenital lobar emphysema	Chronic Obstructive Pulmonary Disease
64357	SNOMED CT	68328006	Centriacinar emphysema	Chronic Obstructive Pulmonary Disease
818673	LOINC	48058-2	Fibrin D-dimer DDU [Mass/volume] in Platelet poor plasma by Immunoassay	D Dimer
818680	LOINC	48065-7	Fibrin D-dimer FEU [Mass/volume] in Platelet poor plasma	D Dimer
818682	LOINC	48067-3	Fibrin D-dimer FEU [Mass/volume] in Platelet poor plasma by Immunoassay	D Dimer
841384	LOINC	71427-9	Fibrin D-dimer FEU [Mass/volume] in Blood by Immunoassay	D Dimer
773718	LOINC	3173-2	aPTT in Blood by Coagulation assay	D Dimer
773799	LOINC	3246-6	Fibrin D-dimer [Units/volume] in Platelet poor plasma by Immunoassay	D Dimer
773809	LOINC	3255-7	Fibrinogen [Mass/volume] in Platelet poor plasma by Coagulation assay	D Dimer
776750	LOINC	5902-2	Prothrombin time (PT)	D Dimer
777601	LOINC	6301-6	INR in Platelet poor plasma by Coagulation assay	D Dimer
779099	LOINC	7799-0	Fibrin D-dimer [Units/volume] in Platelet poor plasma	D Dimer
872167	RxNorm	1037178	dabigatran etexilate 75 MG	Direct Oral Anticoagulants
872168	RxNorm	1037179	dabigatran etexilate 75 MG Oral Capsule	Direct Oral Anticoagulants
872169	RxNorm	1037180	dabigatran etexilate 75 MG [Pradaxa]	Direct Oral Anticoagulants
872170	RxNorm	1037181	dabigatran etexilate 75 MG Oral Capsule [Pradaxa]	Direct Oral Anticoagulants
873354	RxNorm	1037041	dabigatran etexilate mesylate	Direct Oral Anticoagulants
873355	RxNorm	1037042	dabigatran etexilate	Direct Oral Anticoagulants
873356	RxNorm	1037043	dabigatran etexilate 150 MG	Direct Oral Anticoagulants
873357	RxNorm	1037044	dabigatran etexilate Oral Capsule	Direct Oral Anticoagulants
873358	RxNorm	1037045	dabigatran etexilate 150 MG Oral Capsule	Direct Oral Anticoagulants
873360	RxNorm	1037047	dabigatran etexilate 150 MG [Pradaxa]	Direct Oral Anticoagulants

873361	RxNorm	1037048	dabigatran etexilate Oral Capsule [Pradaxa]	Direct Oral Anticoagulants
873362	RxNorm	1037049	dabigatran etexilate 150 MG Oral Capsule [Pradaxa]	Direct Oral Anticoagulants
883422	RxNorm	1114195	rivaroxaban	Direct Oral Anticoagulants
883423	RxNorm	1114196	rivaroxaban 10 MG	Direct Oral Anticoagulants
883424	RxNorm	1114197	rivaroxaban Oral Tablet	Direct Oral Anticoagulants
883425	RxNorm	1114198	rivaroxaban 10 MG Oral Tablet	Direct Oral Anticoagulants
883427	RxNorm	1114200	rivaroxaban 10 MG [Xarelto]	Direct Oral Anticoagulants
883428	RxNorm	1114201	rivaroxaban Oral Tablet [Xarelto]	Direct Oral Anticoagulants
883429	RxNorm	1114202	rivaroxaban 10 MG Oral Tablet [Xarelto]	Direct Oral Anticoagulants
883514	RxNorm	1157968	rivaroxaban Oral Product	Direct Oral Anticoagulants
883515	RxNorm	1157969	rivaroxaban Pill	Direct Oral Anticoagulants
885939	RxNorm	1156646	dabigatran etexilate Oral Product	Direct Oral Anticoagulants
885940	RxNorm	1156647	dabigatran etexilate Pill	Direct Oral Anticoagulants
888407	RxNorm	1161790	warfarin Oral Product	Direct Oral Anticoagulants
893665	RxNorm	1173301	Fragmin Injectable Product	Direct Oral Anticoagulants
896875	RxNorm	1180930	Lovenox Injectable Product	Direct Oral Anticoagulants
903989	RxNorm	1232081	rivaroxaban 15 MG	Direct Oral Anticoagulants
903990	RxNorm	1232082	rivaroxaban 15 MG Oral Tablet	Direct Oral Anticoagulants
903991	RxNorm	1232083	rivaroxaban 15 MG [Xarelto]	Direct Oral Anticoagulants
903992	RxNorm	1232084	rivaroxaban 15 MG Oral Tablet [Xarelto]	Direct Oral Anticoagulants
903993	RxNorm	1232085	rivaroxaban 20 MG	Direct Oral Anticoagulants
903994	RxNorm	1232086	rivaroxaban 20 MG Oral Tablet	Direct Oral Anticoagulants
903995	RxNorm	1232087	rivaroxaban 20 MG [Xarelto]	Direct Oral Anticoagulants
903996	RxNorm	1232088	rivaroxaban 20 MG Oral Tablet [Xarelto]	Direct Oral Anticoagulants
1011873	RxNorm	406078	warfarin Oral Tablet [Jantoven]	Direct Oral Anticoagulants
1050054	RxNorm	855288	warfarin sodium 1 MG Oral Tablet	Direct Oral Anticoagulants
1050056	RxNorm	855290	warfarin sodium 1 MG Oral Tablet [Coumadin]	Direct Oral Anticoagulants
1050058	RxNorm	855292	warfarin sodium 1 MG Oral Tablet [Jantoven]	Direct Oral Anticoagulants
1050062	RxNorm	855296	warfarin sodium 10 MG Oral Tablet	Direct Oral Anticoagulants
1050064	RxNorm	855298	warfarin sodium 10 MG Oral Tablet [Coumadin]	Direct Oral Anticoagulants
1050066	RxNorm	855300	warfarin sodium 10 MG Oral Tablet [Jantoven]	Direct Oral Anticoagulants
1050068	RxNorm	855302	warfarin sodium 2 MG Oral Tablet	Direct Oral Anticoagulants
1050070	RxNorm	855304	warfarin sodium 2 MG Oral Tablet [Coumadin]	Direct Oral Anticoagulants
1050072	RxNorm	855306	warfarin sodium 2 MG Oral Tablet [Jantoven]	Direct Oral Anticoagulants

1050078	RxNorm	855312	warfarin sodium 2.5 MG Oral Tablet	Direct Oral Anticoagulants
1050080	RxNorm	855314	warfarin sodium 2.5 MG Oral Tablet [Coumadin]	Direct Oral Anticoagulants
1050082	RxNorm	855316	warfarin sodium 2.5 MG Oral Tablet [Jantoven]	Direct Oral Anticoagulants
1050084	RxNorm	855318	warfarin sodium 3 MG Oral Tablet	Direct Oral Anticoagulants
1050249	RxNorm	855320	warfarin sodium 3 MG Oral Tablet [Coumadin]	Direct Oral Anticoagulants
1050251	RxNorm	855322	warfarin sodium 3 MG Oral Tablet [Jantoven]	Direct Oral Anticoagulants
1050253	RxNorm	855324	warfarin sodium 4 MG Oral Tablet	Direct Oral Anticoagulants
1050255	RxNorm	855326	warfarin sodium 4 MG Oral Tablet [Coumadin]	Direct Oral Anticoagulants
1050257	RxNorm	855328	warfarin sodium 4 MG Oral Tablet [Jantoven]	Direct Oral Anticoagulants
1050261	RxNorm	855332	warfarin sodium 5 MG Oral Tablet	Direct Oral Anticoagulants
1050263	RxNorm	855334	warfarin sodium 5 MG Oral Tablet [Coumadin]	Direct Oral Anticoagulants
1050265	RxNorm	855336	warfarin sodium 5 MG Oral Tablet [Jantoven]	Direct Oral Anticoagulants
1050267	RxNorm	855338	warfarin sodium 6 MG Oral Tablet	Direct Oral Anticoagulants
1050269	RxNorm	855340	warfarin sodium 6 MG Oral Tablet [Coumadin]	Direct Oral Anticoagulants
1050271	RxNorm	855342	warfarin sodium 6 MG Oral Tablet [Jantoven]	Direct Oral Anticoagulants
1050273	RxNorm	855344	warfarin sodium 7.5 MG Oral Tablet	Direct Oral Anticoagulants
1050275	RxNorm	855346	warfarin sodium 7.5 MG Oral Tablet [Coumadin]	Direct Oral Anticoagulants
1050277	RxNorm	855348	warfarin sodium 7.5 MG Oral Tablet [Jantoven]	Direct Oral Anticoagulants
1050344	RxNorm	855350	warfarin sodium 0.5 MG Oral Tablet	Direct Oral Anticoagulants
924402	RxNorm	1364430	apixaban	Direct Oral Anticoagulants
924403	RxNorm	1364431	apixaban 2.5 MG	Direct Oral Anticoagulants
924404	RxNorm	1364432	apixaban Oral Product	Direct Oral Anticoagulants
924405	RxNorm	1364433	apixaban Pill	Direct Oral Anticoagulants
924406	RxNorm	1364434	apixaban Oral Tablet	Direct Oral Anticoagulants
924407	RxNorm	1364435	apixaban 2.5 MG Oral Tablet	Direct Oral Anticoagulants
924409	RxNorm	1364437	apixaban 2.5 MG [Eliquis]	Direct Oral Anticoagulants
924410	RxNorm	1364438	apixaban Oral Tablet [Eliquis]	Direct Oral Anticoagulants
924658	RxNorm	1364441	apixaban 2.5 MG Oral Tablet [Eliquis]	Direct Oral Anticoagulants
924660	RxNorm	1364444	apixaban 5 MG	Direct Oral Anticoagulants
924661	RxNorm	1364445	apixaban 5 MG Oral Tablet	Direct Oral Anticoagulants

924662	RxNorm	1364446	apixaban 5 MG [Eliquis]	Direct Oral Anticoagulants
924663	RxNorm	1364447	apixaban 5 MG Oral Tablet [Eliquis]	Direct Oral Anticoagulants
935977	RxNorm	1546356	dabigatran	Direct Oral Anticoagulants
936754	RxNorm	1549682	{42 (rivaroxaban 15 MG Oral Tablet) / 9 (rivaroxaban 20 MG Oral Tablet) } Pack	Direct Oral Anticoagulants
936755	RxNorm	1549683	{42 (rivaroxaban 15 MG Oral Tablet [Xarelto]) / 9 (rivaroxaban 20 MG Oral Tablet [Xarelto]) } Pack [Xarelto Kit]	Direct Oral Anticoagulants
939163	RxNorm	1599538	edoxaban	Direct Oral Anticoagulants
939164	RxNorm	1599539	edoxaban 15 MG	Direct Oral Anticoagulants
939165	RxNorm	1599540	edoxaban Oral Product	Direct Oral Anticoagulants
939166	RxNorm	1599541	edoxaban Pill	Direct Oral Anticoagulants
939167	RxNorm	1599542	edoxaban Oral Tablet	Direct Oral Anticoagulants
939168	RxNorm	1599543	edoxaban 15 MG Oral Tablet	Direct Oral Anticoagulants
939170	RxNorm	1599545	edoxaban 15 MG [Savaysa]	Direct Oral Anticoagulants
939171	RxNorm	1599546	edoxaban Oral Tablet [Savaysa]	Direct Oral Anticoagulants
939477	RxNorm	1599549	edoxaban 15 MG Oral Tablet [Savaysa]	Direct Oral Anticoagulants
939478	RxNorm	1599550	edoxaban 30 MG	Direct Oral Anticoagulants
939479	RxNorm	1599551	edoxaban 30 MG Oral Tablet	Direct Oral Anticoagulants
939480	RxNorm	1599552	edoxaban 30 MG [Savaysa]	Direct Oral Anticoagulants
939481	RxNorm	1599553	edoxaban 30 MG Oral Tablet [Savaysa]	Direct Oral Anticoagulants
939482	RxNorm	1599554	edoxaban 60 MG	Direct Oral Anticoagulants
939483	RxNorm	1599555	edoxaban 60 MG Oral Tablet	Direct Oral Anticoagulants
939484	RxNorm	1599556	edoxaban 60 MG [Savaysa]	Direct Oral Anticoagulants
939485	RxNorm	1599557	edoxaban 60 MG Oral Tablet [Savaysa]	Direct Oral Anticoagulants
939486	RxNorm	1599564	edoxaban tosylate	Direct Oral Anticoagulants
940239	RxNorm	1723475	dabigatran etexilate 110 MG	Direct Oral Anticoagulants
940240	RxNorm	1723476	dabigatran etexilate 110 MG Oral Capsule	Direct Oral Anticoagulants
940241	RxNorm	1723477	dabigatran etexilate 110 MG [Pradaxa]	Direct Oral Anticoagulants
940242	RxNorm	1723478	dabigatran etexilate 110 MG Oral Capsule [Pradaxa]	Direct Oral Anticoagulants
974704	RxNorm	225036	Lovenox	Direct Oral Anticoagulants
984713	RxNorm	281554	Fragmin	Direct Oral Anticoagulants
999598	RxNorm	368417	warfarin Oral Tablet [Coumadin]	Direct Oral Anticoagulants
1001478	RxNorm	374319	warfarin Oral Tablet	Direct Oral Anticoagulants
2650008	RxNorm	2590633	dabigatran etexilate 40 MG Oral Pellet [Pradaxa]	Direct Oral Anticoagulants
2650009	RxNorm	2590634	dabigatran etexilate 50 MG	Direct Oral Anticoagulants
2650010	RxNorm	2590636	dabigatran etexilate 50 MG [Pradaxa]	Direct Oral Anticoagulants
2650011	RxNorm	2590635	dabigatran etexilate 50 MG Oral Pellet	Direct Oral Anticoagulants

2650012	RxNorm	2590637	dabigatran etexilate 50 MG Oral Pellet [Pradaxa]	Direct Oral Anticoagulants
2650013	RxNorm	2590615	dabigatran etexilate Oral Pellet	Direct Oral Anticoagulants
2650014	RxNorm	2590618	dabigatran etexilate Oral Pellet [Pradaxa]	Direct Oral Anticoagulants
2650015	RxNorm	2590614	dabigatran etexilate Pellet Product	Direct Oral Anticoagulants
2649993	RxNorm	2590616	dabigatran etexilate 110 MG Oral Pellet	Direct Oral Anticoagulants
2649994	RxNorm	2590619	dabigatran etexilate 110 MG Oral Pellet [Pradaxa]	Direct Oral Anticoagulants
2649995	RxNorm	2590620	dabigatran etexilate 150 MG Oral Pellet	Direct Oral Anticoagulants
2650499	RxNorm	2590617	Pradaxa Pellet Product	Direct Oral Anticoagulants
3001768	RxNorm	2618842	4 ML dalteparin sodium 2500 UNT/ML Injection [Fragmin]	Direct Oral Anticoagulants
1104920	RxNorm	759601	0.3 ML Enoxaparin 100 MG/ML Prefilled Syringe [Lovenox]	Direct Oral Anticoagulants
1105125	RxNorm	759600	1 ML Enoxaparin 100 MG/ML Prefilled Syringe [Lovenox]	Direct Oral Anticoagulants
1105145	RxNorm	540217	Warfarin 2 MG Oral Tablet [Warfaren]	Direct Oral Anticoagulants
1105557	RxNorm	540229	Warfarin 4 MG Oral Tablet [Marfarin]	Direct Oral Anticoagulants
1106121	RxNorm	540208	Warfarin 1 MG Oral Tablet [Narfarin]	Direct Oral Anticoagulants
1107513	RxNorm	543268	Dalteparin 250000 UNT/ML Injectable Solution [Fragmin]	Direct Oral Anticoagulants
1108799	RxNorm	540213	Warfarin 10 MG Oral Tablet [Warfin]	Direct Oral Anticoagulants
1146731	RxNorm	540216	Warfarin Oral Tablet [Warfaren]	Direct Oral Anticoagulants
1146781	RxNorm	540212	Warfarin Oral Tablet [Warfin]	Direct Oral Anticoagulants
1149831	RxNorm	644655	Warfarin Oral Tablet [Warfaring]	Direct Oral Anticoagulants
1150633	RxNorm	644656	Warfarin 10 MG Oral Tablet [Warfaring]	Direct Oral Anticoagulants
1156479	RxNorm	854259	Enoxaparin sodium 150 MG/ML Injectable Solution [Lovenox]	Direct Oral Anticoagulants
1158963	RxNorm	978789	Dalteparin Sodium 7500 UNT/ML [Fragmin]	Direct Oral Anticoagulants
1159831	RxNorm	978762	Dalteparin Sodium 12500 UNT/ML Injectable Solution [Fragmin]	Direct Oral Anticoagulants
1159833	RxNorm	978782	Dalteparin Sodium 250000 UNT/ML Injectable Solution [Fragmin]	Direct Oral Anticoagulants
1159978	RxNorm	978785	Dalteparin Sodium 5000 UNT/ML [Fragmin]	Direct Oral Anticoagulants
1160081	RxNorm	978786	Dalteparin Sodium 5000 UNT/ML Injectable Solution [Fragmin]	Direct Oral Anticoagulants
1160082	RxNorm	978790	Dalteparin Sodium 7500 UNT/ML Injectable Solution [Fragmin]	Direct Oral Anticoagulants

1160162	RxNorm	978781	Dalteparin Sodium 250000 UNT/ML [Fragmin]	Direct Oral Anticoagulants
2503210	RxNorm	2589624	rivaroxaban 1 MG/ML	Direct Oral Anticoagulants
2503211	RxNorm	2589628	rivaroxaban 1 MG/ML [Xarelto]	Direct Oral Anticoagulants
2503212	RxNorm	2589625	rivaroxaban Oral Liquid Product	Direct Oral Anticoagulants
2503213	RxNorm	2589626	rivaroxaban Oral Suspension	Direct Oral Anticoagulants
2503274	RxNorm	2589629	Xarelto Oral Liquid Product	Direct Oral Anticoagulants
2649999	RxNorm	2590623	dabigatran etexilate 20 MG Oral Pellet	Direct Oral Anticoagulants
2650000	RxNorm	2590625	dabigatran etexilate 20 MG Oral Pellet [Pradaxa]	Direct Oral Anticoagulants
2650001	RxNorm	2590626	dabigatran etexilate 30 MG	Direct Oral Anticoagulants
2650002	RxNorm	2590628	dabigatran etexilate 30 MG [Pradaxa]	Direct Oral Anticoagulants
2650003	RxNorm	2590627	dabigatran etexilate 30 MG Oral Pellet	Direct Oral Anticoagulants
2650004	RxNorm	2590629	dabigatran etexilate 30 MG Oral Pellet [Pradaxa]	Direct Oral Anticoagulants
2650005	RxNorm	2590630	dabigatran etexilate 40 MG	Direct Oral Anticoagulants
2650006	RxNorm	2590632	dabigatran etexilate 40 MG [Pradaxa]	Direct Oral Anticoagulants
2650007	RxNorm	2590631	dabigatran etexilate 40 MG Oral Pellet	Direct Oral Anticoagulants
2649996	RxNorm	2590621	dabigatran etexilate 150 MG Oral Pellet [Pradaxa]	Direct Oral Anticoagulants
2649997	RxNorm	2590622	dabigatran etexilate 20 MG	Direct Oral Anticoagulants
3001838	RxNorm	2618841	dalteparin Injection [Fragmin]	Direct Oral Anticoagulants
3001840	RxNorm	2618843	dalteparin sodium 2500 UNT/ML Injection [Fragmin]	Direct Oral Anticoagulants
1216880	RxNorm	2588059	rivaroxaban 155 MG	Direct Oral Anticoagulants
1216881	RxNorm	2588063	rivaroxaban 155 MG [Xarelto]	Direct Oral Anticoagulants
1216882	RxNorm	2588062	rivaroxaban 155 MG Granules for Oral Suspension	Direct Oral Anticoagulants
1216883	RxNorm	2588066	rivaroxaban 155 MG Granules for Oral Suspension [Xarelto]	Direct Oral Anticoagulants
1216884	RxNorm	2588060	rivaroxaban Granule Product	Direct Oral Anticoagulants
1216885	RxNorm	2588061	rivaroxaban Granules for Oral Suspension	Direct Oral Anticoagulants
1216886	RxNorm	2588065	rivaroxaban Granules for Oral Suspension [Xarelto]	Direct Oral Anticoagulants
1217087	RxNorm	2588064	Xarelto Granule Product	Direct Oral Anticoagulants
2649998	RxNorm	2590624	dabigatran etexilate 20 MG [Pradaxa]	Direct Oral Anticoagulants
873359	RxNorm	1037046	Pradaxa	Direct Oral Anticoagulants
883426	RxNorm	1114199	Xarelto	Direct Oral Anticoagulants
895087	RxNorm	1171655	Coumadin Oral Product	Direct Oral Anticoagulants
897801	RxNorm	1181005	Marfarin Oral Product	Direct Oral Anticoagulants
900223	RxNorm	1167808	Jantoven Oral Product	Direct Oral Anticoagulants
902658	RxNorm	1184616	Pradaxa Oral Product	Direct Oral Anticoagulants
902659	RxNorm	1184617	Pradaxa Pill	Direct Oral Anticoagulants

903787	RxNorm	1179316	Narfarin Oral Product	Direct Oral Anticoagulants
906135	RxNorm	1186304	Xarelto Oral Product	Direct Oral Anticoagulants
906136	RxNorm	1186305	Xarelto Pill	Direct Oral Anticoagulants
1025434	RxNorm	540207	warfarin Oral Tablet [Narfarin]	Direct Oral Anticoagulants
1025436	RxNorm	540228	warfarin Oral Tablet [Marfarin]	Direct Oral Anticoagulants
1038683	RxNorm	753110	dalteparin Prefilled Syringe [Fragmin]	Direct Oral Anticoagulants
1041968	RxNorm	759594	enoxaparin Prefilled Syringe [Lovenox]	Direct Oral Anticoagulants
1048513	RxNorm	854231	enoxaparin sodium 100 MG/ML [Lovenox]	Direct Oral Anticoagulants
1048514	RxNorm	854232	0.3 ML enoxaparin sodium 100 MG/ML Prefilled Syringe [Lovenox]	Direct Oral Anticoagulants
1048516	RxNorm	854236	0.4 ML enoxaparin sodium 100 MG/ML Prefilled Syringe [Lovenox]	Direct Oral Anticoagulants
1048642	RxNorm	854239	0.6 ML enoxaparin sodium 100 MG/ML Prefilled Syringe [Lovenox]	Direct Oral Anticoagulants
1048644	RxNorm	854242	0.8 ML enoxaparin sodium 100 MG/ML Prefilled Syringe [Lovenox]	Direct Oral Anticoagulants
1048647	RxNorm	854246	enoxaparin sodium 150 MG/ML [Lovenox]	Direct Oral Anticoagulants
1048648	RxNorm	854247	0.8 ML enoxaparin sodium 150 MG/ML Prefilled Syringe [Lovenox]	Direct Oral Anticoagulants
1048650	RxNorm	854249	1 ML enoxaparin sodium 100 MG/ML Prefilled Syringe [Lovenox]	Direct Oral Anticoagulants
1048652	RxNorm	854253	1 ML enoxaparin sodium 150 MG/ML Prefilled Syringe [Lovenox]	Direct Oral Anticoagulants
1048654	RxNorm	854256	enoxaparin sodium 100 MG/ML Injectable Solution [Lovenox]	Direct Oral Anticoagulants
1050060	RxNorm	855294	warfarin sodium 1 MG Oral Tablet [Narfarin]	Direct Oral Anticoagulants
1050259	RxNorm	855330	warfarin sodium 4 MG Oral Tablet [Marfarin]	Direct Oral Anticoagulants
1060010	RxNorm	978726	dalteparin sodium 12500 UNT/ML [Fragmin]	Direct Oral Anticoagulants
1060011	RxNorm	978727	0.2 ML dalteparin sodium 12500 UNT/ML Prefilled Syringe [Fragmin]	Direct Oral Anticoagulants
1060014	RxNorm	978734	dalteparin sodium 25000 UNT/ML [Fragmin]	Direct Oral Anticoagulants
1060015	RxNorm	978735	0.2 ML dalteparin sodium 25000 UNT/ML Prefilled Syringe [Fragmin]	Direct Oral Anticoagulants

1060017	RxNorm	978737	0.3 ML dalteparin sodium 25000 UNT/ML Prefilled Syringe [Fragmin]	Direct Oral Anticoagulants
1060019	RxNorm	978739	0.4 ML dalteparin sodium 25000 UNT/ML Prefilled Syringe [Fragmin]	Direct Oral Anticoagulants
1060021	RxNorm	978741	0.5 ML dalteparin sodium 25000 UNT/ML Prefilled Syringe [Fragmin]	Direct Oral Anticoagulants
1060122	RxNorm	978745	0.6 ML dalteparin sodium 25000 UNT/ML Prefilled Syringe [Fragmin]	Direct Oral Anticoagulants
1060124	RxNorm	978747	0.72 ML dalteparin sodium 25000 UNT/ML Prefilled Syringe [Fragmin]	Direct Oral Anticoagulants
1060127	RxNorm	978756	dalteparin sodium 10000 UNT/ML [Fragmin]	Direct Oral Anticoagulants
1060128	RxNorm	978757	1 ML dalteparin sodium 10000 UNT/ML Prefilled Syringe [Fragmin]	Direct Oral Anticoagulants
1060130	RxNorm	978760	dalteparin sodium 10000 UNT/ML Injectable Solution [Fragmin]	Direct Oral Anticoagulants
1060139	RxNorm	978775	dalteparin sodium 2500 UNT/ML [Fragmin]	Direct Oral Anticoagulants
1060140	RxNorm	978776	dalteparin sodium 2500 UNT/ML Injectable Solution [Fragmin]	Direct Oral Anticoagulants
1060142	RxNorm	978778	dalteparin sodium 25000 UNT/ML Injectable Solution [Fragmin]	Direct Oral Anticoagulants
1070413	RxNorm	313735	Warfarin 2 MG Oral Tablet	Direct Oral Anticoagulants
1070523	RxNorm	313739	Warfarin 7.5 MG Oral Tablet	Direct Oral Anticoagulants
1070660	RxNorm	759595	0.8 ML Enoxaparin 150 MG/ML Prefilled Syringe [Lovenox]	Direct Oral Anticoagulants
1070661	RxNorm	759597	0.4 ML Enoxaparin 100 MG/ML Prefilled Syringe [Lovenox]	Direct Oral Anticoagulants
1070662	RxNorm	212155	Dalteparin 25000 UNT/ML Injectable Solution [Fragmin]	Direct Oral Anticoagulants
1070663	RxNorm	827129	0.4 ML Dalteparin 25000 UNT/ML Prefilled Syringe [Fragmin]	Direct Oral Anticoagulants
1070735	RxNorm	759598	0.6 ML Enoxaparin 100 MG/ML Prefilled Syringe [Lovenox]	Direct Oral Anticoagulants
1070736	RxNorm	753113	0.5 ML Dalteparin 25000 UNT/ML Prefilled Syringe [Fragmin]	Direct Oral Anticoagulants
1070739	RxNorm	759599	0.8 ML Enoxaparin 100 MG/ML Prefilled Syringe [Lovenox]	Direct Oral Anticoagulants
1070764	RxNorm	753111	0.6 ML Dalteparin 25000 UNT/ML Prefilled Syringe [Fragmin]	Direct Oral Anticoagulants

1070770	RxNorm	314279	Warfarin 5 MG Oral Tablet	Direct Oral Anticoagulants
1070824	RxNorm	313734	Warfarin 2.5 MG Oral Tablet	Direct Oral Anticoagulants
1070908	RxNorm	313732	Warfarin 10 MG Oral Tablet	Direct Oral Anticoagulants
1071178	RxNorm	313738	Warfarin 6 MG Oral Tablet	Direct Oral Anticoagulants
1071202	RxNorm	198349	Warfarin 4 MG Oral Tablet	Direct Oral Anticoagulants
1071221	RxNorm	212123	Warfarin 3 MG Oral Tablet [Coumadin]	Direct Oral Anticoagulants
1071478	RxNorm	759596	1 ML Enoxaparin 150 MG/ML Prefilled Syringe [Lovenox]	Direct Oral Anticoagulants
1072643	RxNorm	753112	0.72 ML Dalteparin 25000 UNT/ML Prefilled Syringe [Fragmin]	Direct Oral Anticoagulants
1072884	RxNorm	212124	Warfarin 6 MG Oral Tablet [Coumadin]	Direct Oral Anticoagulants
1072908	RxNorm	205791	Enoxaparin 100 MG/ML Injectable Solution [Lovenox]	Direct Oral Anticoagulants
1073224	RxNorm	313733	Warfarin 1 MG Oral Tablet	Direct Oral Anticoagulants
1078207	RxNorm	352081	Enoxaparin 150 MG/ML Injectable Solution [Lovenox]	Direct Oral Anticoagulants
1080366	RxNorm	209086	Warfarin 4 MG Oral Tablet [Coumadin]	Direct Oral Anticoagulants
1080367	RxNorm	209088	Warfarin 10 MG Oral Tablet [Coumadin]	Direct Oral Anticoagulants
1081166	RxNorm	209083	Warfarin 2 MG Oral Tablet [Coumadin]	Direct Oral Anticoagulants
1081547	RxNorm	389189	Warfarin 0.5 MG Oral Tablet	Direct Oral Anticoagulants
1081848	RxNorm	209087	Warfarin 7.5 MG Oral Tablet [Coumadin]	Direct Oral Anticoagulants
1081874	RxNorm	209081	Warfarin 5 MG Oral Tablet [Coumadin]	Direct Oral Anticoagulants
1082242	RxNorm	209082	Warfarin 1 MG Oral Tablet [Coumadin]	Direct Oral Anticoagulants
1082243	RxNorm	209084	Warfarin 2.5 MG Oral Tablet [Coumadin]	Direct Oral Anticoagulants
1083057	RxNorm	404260	Warfarin 6 MG Oral Tablet [Jantoven]	Direct Oral Anticoagulants
1083484	RxNorm	404147	Warfarin 7.5 MG Oral Tablet [Jantoven]	Direct Oral Anticoagulants
1083495	RxNorm	404144	Warfarin 2.5 MG Oral Tablet [Jantoven]	Direct Oral Anticoagulants
1083620	RxNorm	404141	Warfarin 5 MG Oral Tablet [Jantoven]	Direct Oral Anticoagulants
1083621	RxNorm	404142	Warfarin 1 MG Oral Tablet [Jantoven]	Direct Oral Anticoagulants
1083924	RxNorm	404148	Warfarin 10 MG Oral Tablet [Jantoven]	Direct Oral Anticoagulants
1083990	RxNorm	827001	0.3 ML Dalteparin 25000 UNT/ML Prefilled Syringe [Fragmin]	Direct Oral Anticoagulants
1083991	RxNorm	827003	0.2 ML Dalteparin 25000 UNT/ML Prefilled Syringe [Fragmin]	Direct Oral Anticoagulants

1084796	RxNorm	827099	0.2 ML Dalteparin 12500 UNT/ML Prefilled Syringe [Fragmin]	Direct Oral Anticoagulants
1084839	RxNorm	404143	Warfarin 2 MG Oral Tablet [Jantoven]	Direct Oral Anticoagulants
1084845	RxNorm	404259	Warfarin 3 MG Oral Tablet [Jantoven]	Direct Oral Anticoagulants
1085193	RxNorm	404146	Warfarin 4 MG Oral Tablet [Jantoven]	Direct Oral Anticoagulants
1085360	RxNorm	827069	1 ML Dalteparin 10000 UNT/ML Prefilled Syringe [Fragmin]	Direct Oral Anticoagulants
1089872	RxNorm	211763	Dalteparin 12500 UNT/ML Injectable Solution [Fragmin]	Direct Oral Anticoagulants
1090093	RxNorm	150775	Dalteparin 10000 UNT/ML Injectable Solution [Fragmin]	Direct Oral Anticoagulants
1092924	RxNorm	645891	Dalteparin 5000 UNT/ML Injectable Solution [Fragmin]	Direct Oral Anticoagulants
1093545	RxNorm	645895	Dalteparin 7500 UNT/ML Injectable Solution [Fragmin]	Direct Oral Anticoagulants
1093553	RxNorm	645887	Dalteparin 2500 UNT/ML Injectable Solution [Fragmin]	Direct Oral Anticoagulants
1098824	RxNorm	314280	Warfarin 3 MG Oral Tablet	Direct Oral Anticoagulants
2503214	RxNorm	2589630	rivaroxaban Oral Suspension [Xarelto]	Direct Oral Anticoagulants
918020	RxNorm	1359890	dalteparin sodium 25000 UNT/ML Prefilled Syringe [Fragmin]	Direct Oral Anticoagulants
919017	RxNorm	1360220	enoxaparin sodium 150 MG/ML Prefilled Syringe [Lovenox]	Direct Oral Anticoagulants
924408	RxNorm	1364436	Eliquis	Direct Oral Anticoagulants
924656	RxNorm	1364439	Eliquis Oral Product	Direct Oral Anticoagulants
924657	RxNorm	1364440	Eliquis Pill	Direct Oral Anticoagulants
927611	RxNorm	1359477	enoxaparin sodium 100 MG/ML Prefilled Syringe [Lovenox]	Direct Oral Anticoagulants
928397	RxNorm	1359549	dalteparin sodium 12500 UNT/ML Prefilled Syringe [Fragmin]	Direct Oral Anticoagulants
928631	RxNorm	1359604	dalteparin sodium 10000 UNT/ML Prefilled Syringe [Fragmin]	Direct Oral Anticoagulants
939169	RxNorm	1599544	Savaysa	Direct Oral Anticoagulants
939172	RxNorm	1599547	Savaysa Oral Product	Direct Oral Anticoagulants
939173	RxNorm	1599548	Savaysa Pill	Direct Oral Anticoagulants
954705	RxNorm	1992427	{74 (apixaban 5 MG Oral Tablet) } Pack	Direct Oral Anticoagulants
954706	RxNorm	1992428	{74 (apixaban 5 MG Oral Tablet [Eliquis]) } Pack [Eliquis 30-Day Starter Pack]	Direct Oral Anticoagulants
962747	RxNorm	2059014	rivaroxaban 2.5 MG	Direct Oral Anticoagulants
962748	RxNorm	2059015	rivaroxaban 2.5 MG Oral Tablet	Direct Oral Anticoagulants
962749	RxNorm	2059016	rivaroxaban 2.5 MG [Xarelto]	Direct Oral Anticoagulants

962750	RxNorm	2059017	rivaroxaban 2.5 MG Oral Tablet [Xarelto]	Direct Oral Anticoagulants
995068	RxNorm	362788	enoxaparin Injectable Solution [Lovenox]	Direct Oral Anticoagulants
996029	RxNorm	363135	dalteparin Injectable Solution [Fragmin]	Direct Oral Anticoagulants
529579	ICD10CM	I82.220	Acute embolism and thrombosis of inferior vena cava	Deep Vein Thrombosis
529587	ICD10CM	I82.401	Acute embolism and thrombosis of unspecified deep veins of right lower extremity	Deep Vein Thrombosis
529588	ICD10CM	I82.402	Acute embolism and thrombosis of unspecified deep veins of left lower extremity	Deep Vein Thrombosis
529589	ICD10CM	I82.403	Acute embolism and thrombosis of unspecified deep veins of lower extremity, bilateral	Deep Vein Thrombosis
529590	ICD10CM	I82.409	Acute embolism and thrombosis of unspecified deep veins of unspecified lower extremity	Deep Vein Thrombosis
529592	ICD10CM	I82.411	Acute embolism and thrombosis of right femoral vein	Deep Vein Thrombosis
529593	ICD10CM	I82.412	Acute embolism and thrombosis of left femoral vein	Deep Vein Thrombosis
529594	ICD10CM	I82.413	Acute embolism and thrombosis of femoral vein, bilateral	Deep Vein Thrombosis
529595	ICD10CM	I82.419	Acute embolism and thrombosis of unspecified femoral vein	Deep Vein Thrombosis
529597	ICD10CM	I82.421	Acute embolism and thrombosis of right iliac vein	Deep Vein Thrombosis
529598	ICD10CM	I82.422	Acute embolism and thrombosis of left iliac vein	Deep Vein Thrombosis
529599	ICD10CM	I82.423	Acute embolism and thrombosis of iliac vein, bilateral	Deep Vein Thrombosis
529600	ICD10CM	I82.429	Acute embolism and thrombosis of unspecified iliac vein	Deep Vein Thrombosis
529602	ICD10CM	I82.431	Acute embolism and thrombosis of right popliteal vein	Deep Vein Thrombosis
529603	ICD10CM	I82.432	Acute embolism and thrombosis of left popliteal vein	Deep Vein Thrombosis
529604	ICD10CM	I82.433	Acute embolism and thrombosis of popliteal vein, bilateral	Deep Vein Thrombosis
529605	ICD10CM	I82.439	Acute embolism and thrombosis of unspecified popliteal vein	Deep Vein Thrombosis
529607	ICD10CM	I82.441	Acute embolism and thrombosis of right tibial vein	Deep Vein Thrombosis
529608	ICD10CM	I82.442	Acute embolism and thrombosis of left tibial vein	Deep Vein Thrombosis
529609	ICD10CM	I82.443	Acute embolism and thrombosis of tibial vein, bilateral	Deep Vein Thrombosis

529610	ICD10CM	I82.449	Acute embolism and thrombosis of unspecified tibial vein	Deep Vein Thrombosis
529612	ICD10CM	I82.451	Acute embolism and thrombosis of right peroneal vein	Deep Vein Thrombosis
529613	ICD10CM	I82.452	Acute embolism and thrombosis of left peroneal vein	Deep Vein Thrombosis
529614	ICD10CM	I82.453	Acute embolism and thrombosis of peroneal vein, bilateral	Deep Vein Thrombosis
529615	ICD10CM	I82.459	Acute embolism and thrombosis of unspecified peroneal vein	Deep Vein Thrombosis
529617	ICD10CM	I82.461	Acute embolism and thrombosis of right calf muscular vein	Deep Vein Thrombosis
529618	ICD10CM	I82.462	Acute embolism and thrombosis of left calf muscular vein	Deep Vein Thrombosis
529619	ICD10CM	I82.463	Acute embolism and thrombosis of calf muscular vein, bilateral	Deep Vein Thrombosis
529620	ICD10CM	I82.469	Acute embolism and thrombosis of unspecified calf muscular vein	Deep Vein Thrombosis
529622	ICD10CM	I82.491	Acute embolism and thrombosis of other specified deep vein of right lower extremity	Deep Vein Thrombosis
529623	ICD10CM	I82.492	Acute embolism and thrombosis of other specified deep vein of left lower extremity	Deep Vein Thrombosis
529624	ICD10CM	I82.493	Acute embolism and thrombosis of other specified deep vein of lower extremity, bilateral	Deep Vein Thrombosis
529625	ICD10CM	I82.499	Acute embolism and thrombosis of other specified deep vein of unspecified lower extremity	Deep Vein Thrombosis
529627	ICD10CM	I82.4Y1	Acute embolism and thrombosis of unspecified deep veins of right proximal lower extremity	Deep Vein Thrombosis
529628	ICD10CM	I82.4Y2	Acute embolism and thrombosis of unspecified deep veins of left proximal lower extremity	Deep Vein Thrombosis
529629	ICD10CM	I82.4Y3	Acute embolism and thrombosis of unspecified deep veins of proximal lower extremity, bilateral	Deep Vein Thrombosis
529630	ICD10CM	I82.4Y9	Acute embolism and thrombosis of unspecified deep veins of unspecified proximal lower extremity	Deep Vein Thrombosis
529632	ICD10CM	I82.4Z1	Acute embolism and thrombosis of unspecified deep veins of right distal lower extremity	Deep Vein Thrombosis
529633	ICD10CM	I82.4Z2	Acute embolism and thrombosis of unspecified deep veins of left distal lower extremity	Deep Vein Thrombosis

529634	ICD10CM	I82.4Z3	Acute embolism and thrombosis of unspecified deep veins of distal lower extremity, bilateral	Deep Vein Thrombosis
529635	ICD10CM	I82.4Z9	Acute embolism and thrombosis of unspecified deep veins of unspecified distal lower extremity	Deep Vein Thrombosis
529699	ICD10CM	I82.621	Acute embolism and thrombosis of deep veins of right upper extremity	Deep Vein Thrombosis
529700	ICD10CM	I82.622	Acute embolism and thrombosis of deep veins of left upper extremity	Deep Vein Thrombosis
529701	ICD10CM	I82.623	Acute embolism and thrombosis of deep veins of upper extremity, bilateral	Deep Vein Thrombosis
529702	ICD10CM	I82.629	Acute embolism and thrombosis of deep veins of unspecified upper extremity	Deep Vein Thrombosis
528294	ICD10CM	I10	Essential (primary) hypertension	Hypertension
528296	ICD10CM	I11.0	Hypertensive heart disease with heart failure	Hypertension
528297	ICD10CM	I11.9	Hypertensive heart disease without heart failure	Hypertension
528299	ICD10CM	I12.0	Hypertensive chronic kidney disease with stage 5 chronic kidney disease or end stage renal disease	Hypertension
528300	ICD10CM	I12.9	Hypertensive chronic kidney disease with stage 1 through stage 4 chronic kidney disease, or unspecified chronic kidney disease	Hypertension
528302	ICD10CM	I13.0	Hypertensive heart and chronic kidney disease with heart failure and stage 1 through stage 4 chronic kidney disease, or unspecified chronic kidney disease	Hypertension
528303	ICD10CM	I13.1	Hypertensive heart and chronic kidney disease without heart failure	Hypertension
528304	ICD10CM	I13.10	Hypertensive heart and chronic kidney disease without heart failure, with stage 1 through stage 4 chronic kidney disease, or unspecified chronic kidney disease	Hypertension
528305	ICD10CM	I13.11	Hypertensive heart and chronic kidney disease without heart failure, with stage 5 chronic	Hypertension

			kidney disease, or end stage renal disease	
528306	ICD10CM	I13.2	Hypertensive heart and chronic kidney disease with heart failure and with stage 5 chronic kidney disease, or end stage renal disease	Hypertension
528307	ICD10CM	I15	Secondary hypertension	Hypertension
528308	ICD10CM	I15.0	Renovascular hypertension	Hypertension
528309	ICD10CM	I15.1	Hypertension secondary to other renal disorders	Hypertension
528310	ICD10CM	I15.2	Hypertension secondary to endocrine disorders	Hypertension
528311	ICD10CM	I15.8	Other secondary hypertension	Hypertension
528312	ICD10CM	I15.9	Secondary hypertension, unspecified	Hypertension
528313	ICD10CM	I16	Hypertensive crisis	Hypertension
528314	ICD10CM	I16.0	Hypertensive urgency	Hypertension
528315	ICD10CM	I16.1	Hypertensive emergency	Hypertension
528316	ICD10CM	I16.9	Hypertensive crisis, unspecified	Hypertension
1174907	ICD9CM	401	Malignant essential hypertension	Hypertension
1174908	ICD9CM	401.1	Benign essential hypertension	Hypertension
1174909	ICD9CM	401.9	Unspecified essential hypertension	Hypertension
1174912	ICD9CM	402	Malignant hypertensive heart disease without heart failure	Hypertension
1174913	ICD9CM	402.01	Malignant hypertensive heart disease with heart failure	Hypertension
1174915	ICD9CM	402.1	Benign hypertensive heart disease without heart failure	Hypertension
1174916	ICD9CM	402.11	Benign hypertensive heart disease with heart failure	Hypertension
1174918	ICD9CM	402.9	Unspecified hypertensive heart disease without heart failure	Hypertension
1174919	ICD9CM	402.91	Unspecified hypertensive heart disease with heart failure	Hypertension
1174922	ICD9CM	403	Hypertensive chronic kidney disease, malignant, with chronic kidney disease stage I through stage IV, or unspecified	Hypertension
1174923	ICD9CM	403.01	Hypertensive chronic kidney disease, malignant, with chronic kidney disease stage V or end stage renal disease	Hypertension
1174925	ICD9CM	403.1	Hypertensive chronic kidney disease, benign, with chronic kidney disease stage I through stage IV, or unspecified	Hypertension

1174926	ICD9CM	403.11	Hypertensive chronic kidney disease, benign, with chronic kidney disease stage V or end stage renal disease	Hypertension
1174928	ICD9CM	403.9	Hypertensive chronic kidney disease, unspecified, with chronic kidney disease stage I through stage IV, or unspecified	Hypertension
1174929	ICD9CM	403.91	Hypertensive chronic kidney disease, unspecified, with chronic kidney disease stage V or end stage renal disease	Hypertension
1174932	ICD9CM	404	Hypertensive heart and chronic kidney disease, malignant, without heart failure and with chronic kidney disease stage I through stage IV, or unspecified	Hypertension
1174933	ICD9CM	404.01	Hypertensive heart and chronic kidney disease, malignant, with heart failure and with chronic kidney disease stage I through stage IV, or unspecified	Hypertension
1174934	ICD9CM	404.02	Hypertensive heart and chronic kidney disease, malignant, without heart failure and with chronic kidney disease stage V or end stage renal disease	Hypertension
1174935	ICD9CM	404.03	Hypertensive heart and chronic kidney disease, malignant, with heart failure and with chronic kidney disease stage V or end stage renal disease	Hypertension
1174937	ICD9CM	404.1	Hypertensive heart and chronic kidney disease, benign, without heart failure and with chronic kidney disease stage I through stage IV, or unspecified	Hypertension
1174938	ICD9CM	404.11	Hypertensive heart and chronic kidney disease, benign, with heart failure and with chronic kidney disease stage I through stage IV, or unspecified	Hypertension
1174939	ICD9CM	404.12	Hypertensive heart and chronic kidney disease, benign, without heart failure and with chronic kidney disease stage V or end stage renal disease	Hypertension
1174940	ICD9CM	404.13	Hypertensive heart and chronic kidney disease, benign, with heart failure and chronic kidney disease stage V or end stage renal disease	Hypertension

1174942	ICD9CM	404.9	Hypertensive heart and chronic kidney disease, unspecified, without heart failure and with chronic kidney disease stage I through stage IV, or unspecified	Hypertension
1174943	ICD9CM	404.91	Hypertensive heart and chronic kidney disease, unspecified, with heart failure and with chronic kidney disease stage I through stage IV, or unspecified	Hypertension
1174944	ICD9CM	404.92	Hypertensive heart and chronic kidney disease, unspecified, without heart failure and with chronic kidney disease stage V or end stage renal disease	Hypertension
1174945	ICD9CM	404.93	Hypertensive heart and chronic kidney disease, unspecified, with heart failure and chronic kidney disease stage V or end stage renal disease	Hypertension
1174948	ICD9CM	405.01	Malignant renovascular hypertension	Hypertension
1174949	ICD9CM	405.09	Other malignant secondary hypertension	Hypertension
1174951	ICD9CM	405.11	Benign renovascular hypertension	Hypertension
1174952	ICD9CM	405.19	Other benign secondary hypertension	Hypertension
1174954	ICD9CM	405.91	Unspecified renovascular hypertension	Hypertension
1174955	ICD9CM	405.99	Other unspecified secondary hypertension	Hypertension
314022	SNOMED CT	371125006	Labile essential hypertension	Hypertension
359571	SNOMED CT	427889009	Hypertension associated with transplantation	Hypertension
360213	SNOMED CT	428575007	Hypertension secondary to kidney transplant	Hypertension
361053	SNOMED CT	429457004	Systolic essential hypertension	Hypertension
1045	SNOMED CT	1201005	Benign essential hypertension	Hypertension
10028	SNOMED CT	10725009	Benign hypertension	Hypertension
14041	SNOMED CT	14973001	Renal sclerosis with hypertension	Hypertension
153251	SNOMED CT	169465000	Hypertension induced by oral contraceptive pill	Hypertension
422553	SNOMED CT	762463000	Diastolic hypertension and systolic hypertension	Hypertension
452334	SNOMED CT	2.85E+14	Chronic kidney disease stage 2 due to benign hypertension	Hypertension

452335	SNOMED CT	2.85E+14	Chronic kidney disease stage 3 due to benign hypertension	Hypertension
457721	SNOMED CT	4.613E+14	Resistant hypertensive disorder	Hypertension
458524	SNOMED CT	1.078E+15	Multiple drug intolerant hypertension	Hypertension
69145	SNOMED CT	73410007	Benign secondary renovascular hypertension	Hypertension
70135	SNOMED CT	74451002	Secondary diastolic hypertension	Hypertension
74366	SNOMED CT	78975002	Malignant essential hypertension	Hypertension
84051	SNOMED CT	89242004	Malignant secondary hypertension	Hypertension
100826	SNOMED CT	111438007	Hypertension secondary to renal disease in obstetric context	Hypertension
110962	SNOMED CT	123799005	Renovascular hypertension	Hypertension
110963	SNOMED CT	123800009	Goldblatt hypertension	Hypertension
177436	SNOMED CT	194783001	Malignant secondary renovascular hypertension	Hypertension
177438	SNOMED CT	194785008	Benign secondary hypertension	Hypertension
177441	SNOMED CT	194788005	Hypertension secondary to endocrine disorder	Hypertension
177444	SNOMED CT	194791005	Hypertension secondary to drug	Hypertension
181626	SNOMED CT	199008003	Pre-existing secondary hypertension complicating pregnancy, childbirth and puerperium	Hypertension
24540	SNOMED CT	26078007	Hypertension secondary to renal disease complicating AND/OR reason for care during childbirth	Hypertension
26465	SNOMED CT	28119000	Renal hypertension	Hypertension
30108	SNOMED CT	31992008	Secondary hypertension	Hypertension
36126	SNOMED CT	38341003	Hypertensive disorder	Hypertension
36758	SNOMED CT	39018007	Renal arterial hypertension	Hypertension
37431	SNOMED CT	39727004	Hypertension secondary to renal disease complicating AND/OR reason for care during puerperium	Hypertension
43802	SNOMED CT	46481004	Low-renin essential hypertension	Hypertension
45363	SNOMED CT	48146000	Diastolic hypertension	Hypertension

45743	SNOMED CT	48552006	Hypertension secondary to renal disease complicating AND/OR reason for care during pregnancy	Hypertension
52940	SNOMED CT	56218007	Systolic hypertension	Hypertension
54328	SNOMED CT	57684003	Parenchymal renal hypertension	Hypertension
56139	SNOMED CT	59621000	Essential hypertension	Hypertension
56236	SNOMED CT	59720008	Sustained diastolic hypertension	Hypertension
61705	SNOMED CT	65518004	Labile diastolic hypertension	Hypertension
394020	SNOMED CT	704667004	Hypertension concurrent and due to end stage renal disease on dialysis	Hypertension
395714	SNOMED CT	706882009	Hypertensive crisis	Hypertension
400620	SNOMED CT	712832005	Supine hypertension	Hypertension
407265	SNOMED CT	720568003	Brachydactyly and arterial hypertension syndrome	Hypertension
424456	SNOMED CT	765182005	Postpartum pre-eclampsia	Hypertension
425474	SNOMED CT	766937004	Hypertension due to gain-of-function mutation in mineralocorticoid receptor	Hypertension
447000	SNOMED CT	871642009	Hypertension due to aortic arch obstruction	Hypertension
447469	SNOMED CT	5.41E+11	Hypertension complicating pregnancy, childbirth and the puerperium, antepartum	Hypertension
448894	SNOMED CT	5.501E+12	Postoperative hypertension	Hypertension
450437	SNOMED CT	4.051E+13	Postpartum pre-existing essential hypertension	Hypertension
450438	SNOMED CT	4.052E+13	Postpartum pregnancy-induced hypertension	Hypertension
450760	SNOMED CT	7.142E+13	Hypertension in chronic kidney disease due to type 2 diabetes mellitus	Hypertension
450762	SNOMED CT	7.17E+13	Hypertension in chronic kidney disease due to type 1 diabetes mellitus	Hypertension
450890	SNOMED CT	8.277E+13	Hypertension complicating pregnancy	Hypertension
451515	SNOMED CT	1.188E+14	Pre-existing hypertensive chronic kidney disease in mother complicating pregnancy	Hypertension

451729	SNOMED CT	1.28E+14	Hypertension concurrent and due to end stage renal disease on dialysis due to type 2 diabetes mellitus	Hypertension
451730	SNOMED CT	1.28E+14	Hypertension concurrent and due to end stage renal disease on dialysis due to type 1 diabetes mellitus	Hypertension
451840	SNOMED CT	1.327E+14	Hypertensive emergency	Hypertension
452017	SNOMED CT	1.401E+14	Hypertension in chronic kidney disease stage 5 due to type 2 diabetes mellitus	Hypertension
452018	SNOMED CT	1.401E+14	Hypertension in chronic kidney disease stage 4 due to type 2 diabetes mellitus	Hypertension
452019	SNOMED CT	1.401E+14	Hypertension in chronic kidney disease stage 3 due to type 2 diabetes mellitus	Hypertension
452020	SNOMED CT	1.401E+14	Hypertension in chronic kidney disease stage 2 due to type 2 diabetes mellitus	Hypertension
454522	SNOMED CT	3.678E+14	Page kidney	Hypertension
455357	SNOMED CT	4.347E+14	Perioperative hypertension	Hypertension
459844	SNOMED CT	1.075E+16	Eclampsia with pre-existing hypertension in childbirth	Hypertension
459865	SNOMED CT	1.076E+16	Pre-existing hypertensive heart and chronic kidney disease in mother complicating childbirth	Hypertension
464947	SNOMED CT	1.623E+16	Labile systemic arterial hypertension	Hypertension
493750	SNOMED CT	48194001	GH - Gestational hypertension	Hypertension
496833	SNOMED CT	95605009	Hemolysis-elevated liver enzymes-low platelet count syndrome	Hypertension
310567	SNOMED CT	367390009	Hypertension in the obstetric context	Hypertension
331337	SNOMED CT	397748008	Hypertension with albuminuria	Hypertension
331832	SNOMED CT	398254007	Toxemia of pregnancy	Hypertension
360807	SNOMED CT	429198000	Exertional hypertension	Hypertension
371545	SNOMED CT	443482000	Hypertensive urgency	Hypertension
387710	SNOMED CT	697929007	Intermittent hypertension	Hypertension
387711	SNOMED CT	697930002	Labile hypertension due to being in a clinical environment	Hypertension

388323	SNOMED CT	698638005	Pregnancy induced hypertension with pulmonary edema	Hypertension
388324	SNOMED CT	698640000	Hypertension in the puerperium with pulmonary edema	Hypertension
219391	SNOMED CT	237279007	Transient hypertension of pregnancy	Hypertension
219393	SNOMED CT	237281009	Moderate proteinuric hypertension of pregnancy	Hypertension
219394	SNOMED CT	237282002	Impending eclampsia	Hypertension
268224	SNOMED CT	288250001	Maternal hypertension	Hypertension
287147	SNOMED CT	307632004	Non-proteinuric hypertension of pregnancy	Hypertension
7651	SNOMED CT	8218002	Chronic hypertension complicating AND/OR reason for care during childbirth	Hypertension
8163	SNOMED CT	8762007	Chronic hypertension in obstetric context	Hypertension
9249	SNOMED CT	9901000	Essential hypertension complicating AND/OR reason for care during puerperium	Hypertension
9870	SNOMED CT	10562009	Malignant hypertension complicating AND/OR reason for care during childbirth	Hypertension
14430	SNOMED CT	15394000	Toxemia of pregnancy	Hypertension
2930151	SNOMED CT	1.204E+09	Hypertension due to congenital adrenal hyperplasia	Hypertension
2931930	SNOMED CT	1.209E+09	Secondary hypertension due to congenital heart disorder	Hypertension
2931931	SNOMED CT	1.209E+09	Secondary hypertension due to renal tubular disorder	Hypertension
65829	SNOMED CT	69909000	Eclampsia added to pre-existing hypertension	Hypertension
66176	SNOMED CT	70272006	Malignant hypertension	Hypertension
67688	SNOMED CT	71874008	Benign essential hypertension complicating AND/OR reason for care during childbirth	Hypertension
67832	SNOMED CT	72022006	Essential hypertension in obstetric context	Hypertension
74209	SNOMED CT	78808002	Essential hypertension complicating AND/OR reason for care during pregnancy	Hypertension
76870	SNOMED CT	81626002	Malignant hypertension in obstetric context	Hypertension
79180	SNOMED CT	84094009	Rebound hypertension	Hypertension

81021	SNOMED CT	86041002	Pre-existing hypertension in obstetric context	Hypertension
181559	SNOMED CT	198941007	Hypertension complicating pregnancy, childbirth and the puerperium	Hypertension
181560	SNOMED CT	198942000	Benign essential hypertension complicating pregnancy, childbirth and the puerperium	Hypertension
181562	SNOMED CT	198944004	Benign essential hypertension complicating pregnancy, childbirth and the puerperium - delivered	Hypertension
181563	SNOMED CT	198945003	Benign essential hypertension complicating pregnancy, childbirth and the puerperium - delivered with postnatal complication	Hypertension
181564	SNOMED CT	198946002	Benign essential hypertension complicating pregnancy, childbirth and the puerperium - not delivered	Hypertension
181565	SNOMED CT	198947006	Benign essential hypertension complicating pregnancy, childbirth and the puerperium with postnatal complication	Hypertension
181567	SNOMED CT	198949009	Renal hypertension complicating pregnancy, childbirth and the puerperium	Hypertension
181569	SNOMED CT	198951008	Renal hypertension complicating pregnancy, childbirth and the puerperium - delivered	Hypertension
181570	SNOMED CT	198952001	Renal hypertension complicating pregnancy, childbirth and the puerperium - delivered with postnatal complication	Hypertension
181571	SNOMED CT	198953006	Renal hypertension complicating pregnancy, childbirth and the puerperium - not delivered	Hypertension
181583	SNOMED CT	198965005	Transient hypertension of pregnancy - delivered	Hypertension
181584	SNOMED CT	198966006	Transient hypertension of pregnancy - delivered with postnatal complication	Hypertension
181585	SNOMED CT	198967002	Transient hypertension of pregnancy - not delivered	Hypertension
181586	SNOMED CT	198968007	Transient hypertension of pregnancy with postnatal complication	Hypertension
181601	SNOMED CT	198983002	Severe pre-eclampsia - delivered	Hypertension

181602	SNOMED CT	198984008	Severe pre-eclampsia - delivered with postnatal complication	Hypertension
181603	SNOMED CT	198985009	Severe pre-eclampsia - not delivered	Hypertension
181604	SNOMED CT	198986005	Severe pre-eclampsia with postnatal complication	Hypertension
181615	SNOMED CT	198997005	Pre-eclampsia or eclampsia with pre-existing hypertension	Hypertension
181617	SNOMED CT	198999008	Pre-eclampsia or eclampsia with pre-existing hypertension - delivered	Hypertension
181618	SNOMED CT	199000005	Pre-eclampsia or eclampsia with pre-existing hypertension - delivered with postnatal complication	Hypertension
181620	SNOMED CT	199002002	Pre-eclampsia or eclampsia with pre-existing hypertension - not delivered	Hypertension
181621	SNOMED CT	199003007	Pre-eclampsia or eclampsia with pre-existing hypertension with postnatal complication	Hypertension
181623	SNOMED CT	199005000	Pre-existing hypertension complicating pregnancy, childbirth and puerperium	Hypertension
181625	SNOMED CT	199007008	Pre-existing hypertensive heart and renal disease complicating pregnancy, childbirth and the puerperium	Hypertension
189085	SNOMED CT	206596003	Neonatal hypertension	Hypertension
17294	SNOMED CT	18416000	Essential hypertension complicating AND/OR reason for care during childbirth	Hypertension
18576	SNOMED CT	19769006	High-renin essential hypertension	Hypertension
21747	SNOMED CT	23130000	Paroxysmal hypertension	Hypertension
22300	SNOMED CT	23717007	Benign essential hypertension complicating AND/OR reason for care during pregnancy	Hypertension
22364	SNOMED CT	23786008	Malignant hypertension complicating AND/OR reason for care during puerperium	Hypertension
22608	SNOMED CT	24042004	Chronic hypertension complicating AND/OR reason for care during puerperium	Hypertension
27527	SNOMED CT	29259002	Malignant hypertension complicating AND/OR reason for care during pregnancy	Hypertension

29550	SNOMED CT	31407004	Pre-existing hypertension complicating AND/OR reason for care during puerperium	Hypertension
32668	SNOMED CT	34694006	Pre-existing hypertension complicating AND/OR reason for care during childbirth	Hypertension
33239	SNOMED CT	35303009	Benign essential hypertension complicating AND/OR reason for care during puerperium	Hypertension
35436	SNOMED CT	37618003	Chronic hypertension complicating AND/OR reason for care during pregnancy	Hypertension
38737	SNOMED CT	41114007	Mild pre-eclampsia	Hypertension
44073	SNOMED CT	46764007	Severe pre-eclampsia	Hypertension
49641	SNOMED CT	52698002	Transient hypertension	Hypertension
59587	SNOMED CT	63287004	Benign essential hypertension in obstetric context	Hypertension
61595	SNOMED CT	65402008	Pre-existing hypertension complicating AND/OR reason for care during pregnancy	Hypertension
63441	SNOMED CT	67359005	Pre-eclampsia added to pre-existing hypertension	Hypertension
528678	ICD10CM	I63.10	Cerebral infarction due to embolism of unspecified precerebral artery	Ischemic Stroke
528680	ICD10CM	I63.111	Cerebral infarction due to embolism of right vertebral artery	Ischemic Stroke
528681	ICD10CM	I63.112	Cerebral infarction due to embolism of left vertebral artery	Ischemic Stroke
528682	ICD10CM	I63.113	Cerebral infarction due to embolism of bilateral vertebral arteries	Ischemic Stroke
528683	ICD10CM	I63.119	Cerebral infarction due to embolism of unspecified vertebral artery	Ischemic Stroke
528684	ICD10CM	I63.12	Cerebral infarction due to embolism of basilar artery	Ischemic Stroke
528686	ICD10CM	I63.131	Cerebral infarction due to embolism of right carotid artery	Ischemic Stroke
528687	ICD10CM	I63.132	Cerebral infarction due to embolism of left carotid artery	Ischemic Stroke
528688	ICD10CM	I63.133	Cerebral infarction due to embolism of bilateral carotid arteries	Ischemic Stroke
528689	ICD10CM	I63.139	Cerebral infarction due to embolism of unspecified carotid artery	Ischemic Stroke

528690	ICD10CM	I63.19	Cerebral infarction due to embolism of other precerebral artery	Ischemic Stroke
528729	ICD10CM	I63.40	Cerebral infarction due to embolism of unspecified cerebral artery	Ischemic Stroke
528731	ICD10CM	I63.411	Cerebral infarction due to embolism of right middle cerebral artery	Ischemic Stroke
528732	ICD10CM	I63.412	Cerebral infarction due to embolism of left middle cerebral artery	Ischemic Stroke
528733	ICD10CM	I63.413	Cerebral infarction due to embolism of bilateral middle cerebral arteries	Ischemic Stroke
528734	ICD10CM	I63.419	Cerebral infarction due to embolism of unspecified middle cerebral artery	Ischemic Stroke
528736	ICD10CM	I63.421	Cerebral infarction due to embolism of right anterior cerebral artery	Ischemic Stroke
528737	ICD10CM	I63.422	Cerebral infarction due to embolism of left anterior cerebral artery	Ischemic Stroke
528738	ICD10CM	I63.423	Cerebral infarction due to embolism of bilateral anterior cerebral arteries	Ischemic Stroke
528739	ICD10CM	I63.429	Cerebral infarction due to embolism of unspecified anterior cerebral artery	Ischemic Stroke
528741	ICD10CM	I63.431	Cerebral infarction due to embolism of right posterior cerebral artery	Ischemic Stroke
528742	ICD10CM	I63.432	Cerebral infarction due to embolism of left posterior cerebral artery	Ischemic Stroke
528743	ICD10CM	I63.433	Cerebral infarction due to embolism of bilateral posterior cerebral arteries	Ischemic Stroke
528744	ICD10CM	I63.439	Cerebral infarction due to embolism of unspecified posterior cerebral artery	Ischemic Stroke
528746	ICD10CM	I63.441	Cerebral infarction due to embolism of right cerebellar artery	Ischemic Stroke
528747	ICD10CM	I63.442	Cerebral infarction due to embolism of left cerebellar artery	Ischemic Stroke
528748	ICD10CM	I63.443	Cerebral infarction due to embolism of bilateral cerebellar arteries	Ischemic Stroke

528749	ICD10CM	I63.449	Cerebral infarction due to embolism of unspecified cerebellar artery	Ischemic Stroke
528750	ICD10CM	I63.49	Cerebral infarction due to embolism of other cerebral artery	Ischemic Stroke
1175289	ICD9CM	433.01	Occlusion and stenosis of basilar artery with cerebral infarction	Ischemic Stroke
1175291	ICD9CM	433.1	Occlusion and stenosis of carotid artery without mention of cerebral infarction	Ischemic Stroke
1175292	ICD9CM	433.11	Occlusion and stenosis of carotid artery with cerebral infarction	Ischemic Stroke
1175295	ICD9CM	433.21	Occlusion and stenosis of vertebral artery with cerebral infarction	Ischemic Stroke
1175298	ICD9CM	433.31	Occlusion and stenosis of multiple and bilateral precerebral arteries with cerebral infarction	Ischemic Stroke
1175301	ICD9CM	433.81	Occlusion and stenosis of other specified precerebral artery with cerebral infarction	Ischemic Stroke
1175304	ICD9CM	433.91	Occlusion and stenosis of unspecified precerebral artery with cerebral infarction	Ischemic Stroke
1175307	ICD9CM	434	Cerebral thrombosis without mention of cerebral infarction	Ischemic Stroke
1175308	ICD9CM	434.01	Cerebral thrombosis with cerebral infarction	Ischemic Stroke
1175311	ICD9CM	434.11	Cerebral embolism with cerebral infarction	Ischemic Stroke
1175314	ICD9CM	434.91	Cerebral artery occlusion, unspecified with cerebral infarction	Ischemic Stroke
1175322	ICD9CM	436	Acute, but ill-defined, cerebrovascular disease	Ischemic Stroke
177837	SNOMED CT	195186005	Cerebral infarction due to embolism of precerebral arteries	Ischemic Stroke
177841	SNOMED CT	195190007	Cerebral infarction due to embolism of cerebral arteries	Ischemic Stroke
1035677	RxNorm	67108	enoxaparin	Low Molecular Weight Heparin
1035678	RxNorm	67109	dalteparin	Low Molecular Weight Heparin
1037316	RxNorm	69646	tinzaparin	Low Molecular Weight Heparin
3001837	RxNorm	2618838	dalteparin Injection	Low Molecular Weight Heparin
3001838	RxNorm	2618841	dalteparin Injection [Fragmin]	Low Molecular Weight Heparin
3001839	RxNorm	2618840	dalteparin sodium 2500 UNT/ML Injection	Low Molecular Weight Heparin
3001840	RxNorm	2618843	dalteparin sodium 2500 UNT/ML Injection [Fragmin]	Low Molecular Weight Heparin

1104533	RxNorm	248140	Dalteparin 12500 UNT/ML Injectable Solution	Low Molecular Weight Heparin
1104920	RxNorm	759601	0.3 ML Enoxaparin 100 MG/ML Prefilled Syringe [Lovenox]	Low Molecular Weight Heparin
1105125	RxNorm	759600	1 ML Enoxaparin 100 MG/ML Prefilled Syringe [Lovenox]	Low Molecular Weight Heparin
1106196	RxNorm	792059	Dalteparin 25000 MG/ML	Low Molecular Weight Heparin
1106207	RxNorm	331530	tinzaparin 20000 UNT/ML	Low Molecular Weight Heparin
1106704	RxNorm	792060	0.3 ML Dalteparin 25000 MG/ML Prefilled Syringe	Low Molecular Weight Heparin
1107129	RxNorm	566681	Enoxaparin 100 MG/ML [Lovenox]	Low Molecular Weight Heparin
1107240	RxNorm	543266	Dalteparin 250000 UNT/ML Injectable Solution	Low Molecular Weight Heparin
1107513	RxNorm	543268	Dalteparin 250000 UNT/ML Injectable Solution [Fragmin]	Low Molecular Weight Heparin
1108089	RxNorm	543265	Dalteparin 250000 UNT/ML	Low Molecular Weight Heparin
1108134	RxNorm	543267	Dalteparin 250000 UNT/ML [Fragmin]	Low Molecular Weight Heparin
1108152	RxNorm	575826	Enoxaparin 150 MG/ML [Lovenox]	Low Molecular Weight Heparin
1108983	RxNorm	562130	Dalteparin 10000 UNT/ML Injectable Solution	Low Molecular Weight Heparin
1109244	RxNorm	802057	Dalteparin 36 MG/ML Injectable Solution	Low Molecular Weight Heparin
1110396	RxNorm	562550	Dalteparin 25000 UNT/ML Injectable Solution	Low Molecular Weight Heparin
1111311	RxNorm	564999	tinzaparin 1000 UNT/ML [Innohep]	Low Molecular Weight Heparin
1117010	RxNorm	349270	Enoxaparin 150 MG/ML Injectable Solution	Low Molecular Weight Heparin
1127208	RxNorm	248141	Dalteparin Sodium 25000 UNT/ML Injectable Solution	Low Molecular Weight Heparin
1127791	RxNorm	242501	Dalteparin Sodium 10000 UNT/ML Injectable Solution	Low Molecular Weight Heparin
1127955	RxNorm	415058	Dalteparin Sodium 55.2 MG/ML Injectable Solution	Low Molecular Weight Heparin
1129362	RxNorm	450533	Dalteparin Sodium 10000 UNT/ML	Low Molecular Weight Heparin
1130015	RxNorm	451379	Dalteparin Sodium 25000 UNT/ML	Low Molecular Weight Heparin
1130016	RxNorm	451381	Dalteparin Sodium 55.2 MG/ML	Low Molecular Weight Heparin
1140389	RxNorm	283855	TINZAPARIN SODIUM,PORCINE 20000 UNT/ML Injectable Solution	Low Molecular Weight Heparin
1141958	RxNorm	333556	TINZAPARIN SODIUM,PORCINE 20000 UNT/ML	Low Molecular Weight Heparin
1153179	RxNorm	792841	0.3 ML Enoxaparin 100 MG/ML Prefilled Syringe [Lovenox Novaplus]	Low Molecular Weight Heparin

1153182	RxNorm	792843	0.6 ML Enoxaparin 100 MG/ML Prefilled Syringe [Lovenox Novaplus]	Low Molecular Weight Heparin
1153184	RxNorm	792835	1 ML Enoxaparin 100 MG/ML Prefilled Syringe [Lovenox Novaplus]	Low Molecular Weight Heparin
1153507	RxNorm	792840	Enoxaparin 100 MG/ML Injectable Solution [Lovenox Novaplus]	Low Molecular Weight Heparin
1153692	RxNorm	792839	Enoxaparin Injectable Solution [Lovenox Novaplus]	Low Molecular Weight Heparin
1154193	RxNorm	792837	0.8 ML Enoxaparin 150 MG/ML Prefilled Syringe [Lovenox Novaplus]	Low Molecular Weight Heparin
1154197	RxNorm	795086	Enoxaparin 150 MG/ML Injectable Solution [Lovenox Novaplus]	Low Molecular Weight Heparin
1154907	RxNorm	854258	Enoxaparin sodium 150 MG/ML Injectable Solution	Low Molecular Weight Heparin
1155179	RxNorm	792842	0.4 ML Enoxaparin 100 MG/ML Prefilled Syringe [Lovenox Novaplus]	Low Molecular Weight Heparin
1155181	RxNorm	792844	0.8 ML Enoxaparin 100 MG/ML Prefilled Syringe [Lovenox Novaplus]	Low Molecular Weight Heparin
1155505	RxNorm	792833	Enoxaparin 100 MG/ML [Lovenox Novaplus]	Low Molecular Weight Heparin
1155884	RxNorm	792834	Enoxaparin Prefilled Syringe [Lovenox Novaplus]	Low Molecular Weight Heparin
1156479	RxNorm	854259	Enoxaparin sodium 150 MG/ML Injectable Solution [Lovenox]	Low Molecular Weight Heparin
1156631	RxNorm	792836	Enoxaparin 150 MG/ML [Lovenox Novaplus]	Low Molecular Weight Heparin
1156669	RxNorm	792838	1 ML Enoxaparin 150 MG/ML Prefilled Syringe [Lovenox Novaplus]	Low Molecular Weight Heparin
1158963	RxNorm	978789	Dalteparin Sodium 7500 UNT/ML [Fragmin]	Low Molecular Weight Heparin
1159313	RxNorm	978783	Dalteparin Sodium 5000 UNT/ML	Low Molecular Weight Heparin
1159831	RxNorm	978762	Dalteparin Sodium 12500 UNT/ML Injectable Solution [Fragmin]	Low Molecular Weight Heparin
1159832	RxNorm	978779	Dalteparin Sodium 250000 UNT/ML	Low Molecular Weight Heparin
1159833	RxNorm	978782	Dalteparin Sodium 250000 UNT/ML Injectable Solution [Fragmin]	Low Molecular Weight Heparin
1159834	RxNorm	978787	Dalteparin Sodium 7500 UNT/ML	Low Molecular Weight Heparin
1159978	RxNorm	978785	Dalteparin Sodium 5000 UNT/ML [Fragmin]	Low Molecular Weight Heparin

1160081	RxNorm	978786	Dalteparin Sodium 5000 UNT/ML Injectable Solution [Fragmin]	Low Molecular Weight Heparin
1160082	RxNorm	978790	Dalteparin Sodium 7500 UNT/ML Injectable Solution [Fragmin]	Low Molecular Weight Heparin
1160162	RxNorm	978781	Dalteparin Sodium 250000 UNT/ML [Fragmin]	Low Molecular Weight Heparin
1160200	RxNorm	978788	Dalteparin Sodium 7500 UNT/ML Injectable Solution	Low Molecular Weight Heparin
1160220	RxNorm	978761	Dalteparin Sodium 12500 UNT/ML Injectable Solution	Low Molecular Weight Heparin
1160221	RxNorm	978784	Dalteparin Sodium 5000 UNT/ML Injectable Solution	Low Molecular Weight Heparin
1160241	RxNorm	978780	Dalteparin Sodium 250000 UNT/ML Injectable Solution	Low Molecular Weight Heparin
1161169	RxNorm	1009427	Enoxaparin sodium 96 MG/ML Injectable Solution	Low Molecular Weight Heparin
1163408	RxNorm	1009426	Enoxaparin sodium 96 MG/ML	Low Molecular Weight Heparin
3001767	RxNorm	2618839	4 ML dalteparin sodium 2500 UNT/ML Injection	Low Molecular Weight Heparin
3001768	RxNorm	2618842	4 ML dalteparin sodium 2500 UNT/ML Injection [Fragmin]	Low Molecular Weight Heparin
869653	RxNorm	1008696	dalteparin / dihydroergotamine	Low Molecular Weight Heparin
872908	RxNorm	1036855	tinzaparin sodium 10000 UNT/ML [Innohep]	Low Molecular Weight Heparin
882371	RxNorm	1151836	dalteparin / dihydroergotamine Injectable Product	Low Molecular Weight Heparin
882372	RxNorm	1151837	dalteparin Injectable Product	Low Molecular Weight Heparin
890527	RxNorm	1162099	tinzaparin Injectable Product	Low Molecular Weight Heparin
893665	RxNorm	1173301	Fragmin Injectable Product	Low Molecular Weight Heparin
896875	RxNorm	1180930	Lovenox Injectable Product	Low Molecular Weight Heparin
897719	RxNorm	1162664	enoxaparin Injectable Product	Low Molecular Weight Heparin
898906	RxNorm	1172769	Innohep Injectable Product	Low Molecular Weight Heparin
1011697	RxNorm	413228	tinzaparin 141 MG/ML Injectable Solution	Low Molecular Weight Heparin
1012337	RxNorm	413808	dalteparin sodium 36 MG/ML Injectable Solution	Low Molecular Weight Heparin
1012338	RxNorm	413809	dalteparin 75 MG/ML Injectable Solution	Low Molecular Weight Heparin
1013217	RxNorm	415269	tinzaparin 120 MG/ML Injectable Solution	Low Molecular Weight Heparin
1013224	RxNorm	415320	enoxaparin 60 MG/ML Injectable Solution	Low Molecular Weight Heparin
1014404	RxNorm	439030	dalteparin / dihydroergotamine Injectable Solution	Low Molecular Weight Heparin
1020706	RxNorm	438891	dalteparin 75 MG/ML	Low Molecular Weight Heparin
1021454	RxNorm	451380	dalteparin sodium 36 MG/ML	Low Molecular Weight Heparin
1022710	RxNorm	452215	tinzaparin 141 MG/ML	Low Molecular Weight Heparin
1023596	RxNorm	451808	enoxaparin 60 MG/ML	Low Molecular Weight Heparin
1025307	RxNorm	452838	tinzaparin 120 MG/ML	Low Molecular Weight Heparin

1036817	RxNorm	727382	dalteparin Prefilled Syringe	Low Molecular Weight Heparin
1037812	RxNorm	727722	enoxaparin Prefilled Syringe	Low Molecular Weight Heparin
1038060	RxNorm	727880	tinzaparin Prefilled Syringe	Low Molecular Weight Heparin
1038683	RxNorm	753110	dalteparin Prefilled Syringe [Fragmin]	Low Molecular Weight Heparin
1041968	RxNorm	759594	enoxaparin Prefilled Syringe [Lovenox]	Low Molecular Weight Heparin
1045804	RxNorm	802055	dalteparin 55.2 MG/ML	Low Molecular Weight Heparin
1045805	RxNorm	802056	dalteparin 55.2 MG/ML Injectable Solution	Low Molecular Weight Heparin
1048511	RxNorm	854227	enoxaparin sodium 100 MG/ML	Low Molecular Weight Heparin
1048512	RxNorm	854228	0.3 ML enoxaparin sodium 100 MG/ML Prefilled Syringe	Low Molecular Weight Heparin
1048513	RxNorm	854231	enoxaparin sodium 100 MG/ML [Lovenox]	Low Molecular Weight Heparin
1048514	RxNorm	854232	0.3 ML enoxaparin sodium 100 MG/ML Prefilled Syringe [Lovenox]	Low Molecular Weight Heparin
1048515	RxNorm	854235	0.4 ML enoxaparin sodium 100 MG/ML Prefilled Syringe	Low Molecular Weight Heparin
1048516	RxNorm	854236	0.4 ML enoxaparin sodium 100 MG/ML Prefilled Syringe [Lovenox]	Low Molecular Weight Heparin
1048517	RxNorm	854238	0.6 ML enoxaparin sodium 100 MG/ML Prefilled Syringe	Low Molecular Weight Heparin
1048642	RxNorm	854239	0.6 ML enoxaparin sodium 100 MG/ML Prefilled Syringe [Lovenox]	Low Molecular Weight Heparin
1048643	RxNorm	854241	0.8 ML enoxaparin sodium 100 MG/ML Prefilled Syringe	Low Molecular Weight Heparin
1048644	RxNorm	854242	0.8 ML enoxaparin sodium 100 MG/ML Prefilled Syringe [Lovenox]	Low Molecular Weight Heparin
1048645	RxNorm	854244	enoxaparin sodium 150 MG/ML	Low Molecular Weight Heparin
1048646	RxNorm	854245	0.8 ML enoxaparin sodium 150 MG/ML Prefilled Syringe	Low Molecular Weight Heparin
1048647	RxNorm	854246	enoxaparin sodium 150 MG/ML [Lovenox]	Low Molecular Weight Heparin
1048648	RxNorm	854247	0.8 ML enoxaparin sodium 150 MG/ML Prefilled Syringe [Lovenox]	Low Molecular Weight Heparin
1048649	RxNorm	854248	1 ML enoxaparin sodium 100 MG/ML Prefilled Syringe	Low Molecular Weight Heparin
1048650	RxNorm	854249	1 ML enoxaparin sodium 100 MG/ML Prefilled Syringe [Lovenox]	Low Molecular Weight Heparin
1048651	RxNorm	854252	1 ML enoxaparin sodium 150 MG/ML Prefilled Syringe	Low Molecular Weight Heparin
1048652	RxNorm	854253	1 ML enoxaparin sodium 150 MG/ML Prefilled Syringe [Lovenox]	Low Molecular Weight Heparin

1048653	RxNorm	854255	enoxaparin sodium 100 MG/ML Injectable Solution	Low Molecular Weight Heparin
1048654	RxNorm	854256	enoxaparin sodium 100 MG/ML Injectable Solution [Lovenox]	Low Molecular Weight Heparin
1059996	RxNorm	978712	tinzaparin sodium 20000 UNT/ML	Low Molecular Weight Heparin
1059997	RxNorm	978713	tinzaparin sodium 20000 UNT/ML Injectable Solution	Low Molecular Weight Heparin
1059998	RxNorm	978714	tinzaparin sodium 20000 UNT/ML [Innohep]	Low Molecular Weight Heparin
1059999	RxNorm	978715	tinzaparin sodium 20000 UNT/ML Injectable Solution [Innohep]	Low Molecular Weight Heparin
1060000	RxNorm	978716	tinzaparin sodium 10000 UNT/ML	Low Molecular Weight Heparin
1060001	RxNorm	978717	tinzaparin sodium 10000 UNT/ML Injectable Solution	Low Molecular Weight Heparin
1060002	RxNorm	978718	0.25 ML tinzaparin sodium 10000 UNT/ML Prefilled Syringe	Low Molecular Weight Heparin
1060003	RxNorm	978719	0.35 ML tinzaparin sodium 10000 UNT/ML Prefilled Syringe	Low Molecular Weight Heparin
1060004	RxNorm	978720	0.45 ML tinzaparin sodium 10000 UNT/ML Prefilled Syringe	Low Molecular Weight Heparin
1060005	RxNorm	978721	0.5 ML tinzaparin sodium 20000 UNT/ML Prefilled Syringe	Low Molecular Weight Heparin
1060006	RxNorm	978722	0.7 ML tinzaparin sodium 20000 UNT/ML Prefilled Syringe	Low Molecular Weight Heparin
1060007	RxNorm	978723	0.9 ML tinzaparin sodium 20000 UNT/ML Prefilled Syringe	Low Molecular Weight Heparin
1060008	RxNorm	978724	dalteparin sodium 12500 UNT/ML	Low Molecular Weight Heparin
1060009	RxNorm	978725	0.2 ML dalteparin sodium 12500 UNT/ML Prefilled Syringe	Low Molecular Weight Heparin
1060010	RxNorm	978726	dalteparin sodium 12500 UNT/ML [Fragmin]	Low Molecular Weight Heparin
1060011	RxNorm	978727	0.2 ML dalteparin sodium 12500 UNT/ML Prefilled Syringe [Fragmin]	Low Molecular Weight Heparin
1060012	RxNorm	978732	dalteparin sodium 25000 UNT/ML	Low Molecular Weight Heparin
1060013	RxNorm	978733	0.2 ML dalteparin sodium 25000 UNT/ML Prefilled Syringe	Low Molecular Weight Heparin
1060014	RxNorm	978734	dalteparin sodium 25000 UNT/ML [Fragmin]	Low Molecular Weight Heparin
1060015	RxNorm	978735	0.2 ML dalteparin sodium 25000 UNT/ML Prefilled Syringe [Fragmin]	Low Molecular Weight Heparin
1060016	RxNorm	978736	0.3 ML dalteparin sodium 25000 UNT/ML Prefilled Syringe	Low Molecular Weight Heparin

1060017	RxNorm	978737	0.3 ML dalteparin sodium 25000 UNT/ML Prefilled Syringe [Fragmin]	Low Molecular Weight Heparin
1060018	RxNorm	978738	0.4 ML dalteparin sodium 25000 UNT/ML Prefilled Syringe	Low Molecular Weight Heparin
1060019	RxNorm	978739	0.4 ML dalteparin sodium 25000 UNT/ML Prefilled Syringe [Fragmin]	Low Molecular Weight Heparin
1060020	RxNorm	978740	0.5 ML dalteparin sodium 25000 UNT/ML Prefilled Syringe	Low Molecular Weight Heparin
1060021	RxNorm	978741	0.5 ML dalteparin sodium 25000 UNT/ML Prefilled Syringe [Fragmin]	Low Molecular Weight Heparin
1060022	RxNorm	978744	0.6 ML dalteparin sodium 25000 UNT/ML Prefilled Syringe	Low Molecular Weight Heparin
1060122	RxNorm	978745	0.6 ML dalteparin sodium 25000 UNT/ML Prefilled Syringe [Fragmin]	Low Molecular Weight Heparin
1060123	RxNorm	978746	0.72 ML dalteparin sodium 25000 UNT/ML Prefilled Syringe	Low Molecular Weight Heparin
1060124	RxNorm	978747	0.72 ML dalteparin sodium 25000 UNT/ML Prefilled Syringe [Fragmin]	Low Molecular Weight Heparin
1060125	RxNorm	978754	dalteparin sodium 10000 UNT/ML	Low Molecular Weight Heparin
1060126	RxNorm	978755	1 ML dalteparin sodium 10000 UNT/ML Prefilled Syringe	Low Molecular Weight Heparin
1060127	RxNorm	978756	dalteparin sodium 10000 UNT/ML [Fragmin]	Low Molecular Weight Heparin
1060128	RxNorm	978757	1 ML dalteparin sodium 10000 UNT/ML Prefilled Syringe [Fragmin]	Low Molecular Weight Heparin
1060129	RxNorm	978759	dalteparin sodium 10000 UNT/ML Injectable Solution	Low Molecular Weight Heparin
1060130	RxNorm	978760	dalteparin sodium 10000 UNT/ML Injectable Solution [Fragmin]	Low Molecular Weight Heparin
1060137	RxNorm	978773	dalteparin sodium 2500 UNT/ML	Low Molecular Weight Heparin
1060138	RxNorm	978774	dalteparin sodium 2500 UNT/ML Injectable Solution	Low Molecular Weight Heparin
1060139	RxNorm	978775	dalteparin sodium 2500 UNT/ML [Fragmin]	Low Molecular Weight Heparin
1060140	RxNorm	978776	dalteparin sodium 2500 UNT/ML Injectable Solution [Fragmin]	Low Molecular Weight Heparin
1060141	RxNorm	978777	dalteparin sodium 25000 UNT/ML Injectable Solution	Low Molecular Weight Heparin
1060142	RxNorm	978778	dalteparin sodium 25000 UNT/ML Injectable Solution [Fragmin]	Low Molecular Weight Heparin
1069845	RxNorm	282479	Enoxaparin 100 MG/ML Injectable Solution	Low Molecular Weight Heparin

1070010	RxNorm	727727	0.4 ML Enoxaparin 100 MG/ML Prefilled Syringe	Low Molecular Weight Heparin
1070181	RxNorm	727723	1 ML Enoxaparin 100 MG/ML Prefilled Syringe	Low Molecular Weight Heparin
1070213	RxNorm	727729	0.8 ML Enoxaparin 100 MG/ML Prefilled Syringe	Low Molecular Weight Heparin
1070308	RxNorm	727724	0.8 ML Enoxaparin 150 MG/ML Prefilled Syringe	Low Molecular Weight Heparin
1070309	RxNorm	727726	0.3 ML Enoxaparin 100 MG/ML Prefilled Syringe	Low Molecular Weight Heparin
1070310	RxNorm	727384	0.2 ML Dalteparin 12500 UNT/ML Prefilled Syringe	Low Molecular Weight Heparin
1070311	RxNorm	727718	0.2 ML Dalteparin 25000 UNT/ML Prefilled Syringe	Low Molecular Weight Heparin
1070330	RxNorm	727728	0.6 ML Enoxaparin 100 MG/ML Prefilled Syringe	Low Molecular Weight Heparin
1070331	RxNorm	727838	0.4 ML Dalteparin 25000 UNT/ML Prefilled Syringe	Low Molecular Weight Heparin
1070412	RxNorm	727884	0.7 ML tinzaparin 20000 UNT/ML Prefilled Syringe	Low Molecular Weight Heparin
1070627	RxNorm	315857	Enoxaparin 100 MG/ML	Low Molecular Weight Heparin
1070660	RxNorm	759595	0.8 ML Enoxaparin 150 MG/ML Prefilled Syringe [Lovenox]	Low Molecular Weight Heparin
1070661	RxNorm	759597	0.4 ML Enoxaparin 100 MG/ML Prefilled Syringe [Lovenox]	Low Molecular Weight Heparin
1070662	RxNorm	212155	Dalteparin 25000 UNT/ML Injectable Solution [Fragmin]	Low Molecular Weight Heparin
1070663	RxNorm	827129	0.4 ML Dalteparin 25000 UNT/ML Prefilled Syringe [Fragmin]	Low Molecular Weight Heparin
1070675	RxNorm	284458	tinzaparin 20000 UNT/ML Injectable Solution [Innohep]	Low Molecular Weight Heparin
1070735	RxNorm	759598	0.6 ML Enoxaparin 100 MG/ML Prefilled Syringe [Lovenox]	Low Molecular Weight Heparin
1070736	RxNorm	753113	0.5 ML Dalteparin 25000 UNT/ML Prefilled Syringe [Fragmin]	Low Molecular Weight Heparin
1070739	RxNorm	759599	0.8 ML Enoxaparin 100 MG/ML Prefilled Syringe [Lovenox]	Low Molecular Weight Heparin
1070764	RxNorm	753111	0.6 ML Dalteparin 25000 UNT/ML Prefilled Syringe [Fragmin]	Low Molecular Weight Heparin
1070806	RxNorm	248379	Dalteparin 2500 UNT/ML Injectable Solution	Low Molecular Weight Heparin
1070904	RxNorm	351111	tinzaparin 10000 UNT/ML Injectable Solution	Low Molecular Weight Heparin
1070905	RxNorm	727881	0.45 ML tinzaparin 10000 UNT/ML Prefilled Syringe	Low Molecular Weight Heparin
1071167	RxNorm	727861	0.6 ML Dalteparin 25000 UNT/ML Prefilled Syringe	Low Molecular Weight Heparin

1071478	RxNorm	759596	1 ML Enoxaparin 150 MG/ML Prefilled Syringe [Lovenox]	Low Molecular Weight Heparin
1071553	RxNorm	353346	tinzaparin 10000 UNT/ML	Low Molecular Weight Heparin
1072437	RxNorm	727725	1 ML Enoxaparin 150 MG/ML Prefilled Syringe	Low Molecular Weight Heparin
1072438	RxNorm	727383	1 ML Dalteparin 10000 UNT/ML Prefilled Syringe	Low Molecular Weight Heparin
1072439	RxNorm	727859	0.5 ML Dalteparin 25000 UNT/ML Prefilled Syringe	Low Molecular Weight Heparin
1072456	RxNorm	727883	0.5 ML tinzaparin 20000 UNT/ML Prefilled Syringe	Low Molecular Weight Heparin
1072457	RxNorm	727892	0.35 ML tinzaparin 10000 UNT/ML Prefilled Syringe	Low Molecular Weight Heparin
1072643	RxNorm	753112	0.72 ML Dalteparin 25000 UNT/ML Prefilled Syringe [Fragmin]	Low Molecular Weight Heparin
1072908	RxNorm	205791	Enoxaparin 100 MG/ML Injectable Solution [Lovenox]	Low Molecular Weight Heparin
1075460	RxNorm	564789	Dalteparin 10000 UNT/ML [Fragmin]	Low Molecular Weight Heparin
1077095	RxNorm	351042	Enoxaparin 150 MG/ML	Low Molecular Weight Heparin
1078207	RxNorm	352081	Enoxaparin 150 MG/ML Injectable Solution [Lovenox]	Low Molecular Weight Heparin
1078560	RxNorm	572437	Dalteparin 25000 UNT/ML [Fragmin]	Low Molecular Weight Heparin
1079188	RxNorm	565919	tinzaparin 10000 UNT/ML [Innohep]	Low Molecular Weight Heparin
1081709	RxNorm	385922	tinzaparin 1000 UNT/ML	Low Molecular Weight Heparin
1082198	RxNorm	572099	Dalteparin 12500 UNT/ML [Fragmin]	Low Molecular Weight Heparin
1082659	RxNorm	402604	Dalteparin 10000 UNT/ML	Low Molecular Weight Heparin
1083049	RxNorm	402609	Dalteparin 2500 UNT/ML	Low Molecular Weight Heparin
1083990	RxNorm	827001	0.3 ML Dalteparin 25000 UNT/ML Prefilled Syringe [Fragmin]	Low Molecular Weight Heparin
1083991	RxNorm	827003	0.2 ML Dalteparin 25000 UNT/ML Prefilled Syringe [Fragmin]	Low Molecular Weight Heparin
1084796	RxNorm	827099	0.2 ML Dalteparin 12500 UNT/ML Prefilled Syringe [Fragmin]	Low Molecular Weight Heparin
1085025	RxNorm	827000	0.3 ML Dalteparin 25000 UNT/ML Prefilled Syringe	Low Molecular Weight Heparin
1085129	RxNorm	402608	Dalteparin 12500 UNT/ML	Low Molecular Weight Heparin
1085360	RxNorm	827069	1 ML Dalteparin 10000 UNT/ML Prefilled Syringe [Fragmin]	Low Molecular Weight Heparin
1087161	RxNorm	413573	Dalteparin 36 MG/ML / Dihydroergotamine 1 MG/ML Injectable Solution	Low Molecular Weight Heparin
1089872	RxNorm	211763	Dalteparin 12500 UNT/ML Injectable Solution [Fragmin]	Low Molecular Weight Heparin

1090093	RxNorm	150775	Dalteparin 10000 UNT/ML Injectable Solution [Fragmin]	Low Molecular Weight Heparin
1092085	RxNorm	645890	Dalteparin 5000 UNT/ML [Fragmin]	Low Molecular Weight Heparin
1092480	RxNorm	645894	Dalteparin 7500 UNT/ML [Fragmin]	Low Molecular Weight Heparin
1092691	RxNorm	645888	Dalteparin 5000 UNT/ML	Low Molecular Weight Heparin
1092719	RxNorm	152607	tinzaparin 1000 UNT/ML Injectable Solution [Innohep]	Low Molecular Weight Heparin
1092924	RxNorm	645891	Dalteparin 5000 UNT/ML Injectable Solution [Fragmin]	Low Molecular Weight Heparin
1093329	RxNorm	645889	Dalteparin 5000 UNT/ML Injectable Solution	Low Molecular Weight Heparin
1093507	RxNorm	152604	tinzaparin 1000 UNT/ML Injectable Solution	Low Molecular Weight Heparin
1093545	RxNorm	645895	Dalteparin 7500 UNT/ML Injectable Solution [Fragmin]	Low Molecular Weight Heparin
1093553	RxNorm	645887	Dalteparin 2500 UNT/ML Injectable Solution [Fragmin]	Low Molecular Weight Heparin
1093554	RxNorm	645892	Dalteparin 7500 UNT/ML	Low Molecular Weight Heparin
1094532	RxNorm	645893	Dalteparin 7500 UNT/ML Injectable Solution	Low Molecular Weight Heparin
1094783	RxNorm	645886	Dalteparin 2500 UNT/ML [Fragmin]	Low Molecular Weight Heparin
1096180	RxNorm	438892	Dalteparin 36 MG/ML	Low Molecular Weight Heparin
1097896	RxNorm	574893	tinzaparin 20000 UNT/ML [Innohep]	Low Molecular Weight Heparin
1099483	RxNorm	313410	tinzaparin 20000 UNT/ML Injectable Solution	Low Molecular Weight Heparin
1101316	RxNorm	727730	0.2 ML Enoxaparin 100 MG/ML Prefilled Syringe	Low Molecular Weight Heparin
1101319	RxNorm	727882	0.25 ML tinzaparin 10000 UNT/ML Prefilled Syringe	Low Molecular Weight Heparin
1102317	RxNorm	329206	Dalteparin 25000 UNT/ML	Low Molecular Weight Heparin
1102369	RxNorm	727860	0.72 ML Dalteparin 25000 UNT/ML Prefilled Syringe	Low Molecular Weight Heparin
1102370	RxNorm	727888	0.9 ML tinzaparin 20000 UNT/ML Prefilled Syringe	Low Molecular Weight Heparin
1102402	RxNorm	727719	0.3 ML Dalteparin 2500 UNT/ML Prefilled Syringe	Low Molecular Weight Heparin
908335	RxNorm	1233976	dalteparin sodium 36 MG/ML / dihydroergotamine mesylate 1 MG/ML Injectable Solution	Low Molecular Weight Heparin
909750	RxNorm	1245458	0.2 ML enoxaparin sodium 100 MG/ML Prefilled Syringe	Low Molecular Weight Heparin
917041	RxNorm	1359696	dalteparin sodium 10000 UNT/ML Prefilled Syringe	Low Molecular Weight Heparin
917229	RxNorm	1359740	enoxaparin sodium 150 MG/ML Prefilled Syringe	Low Molecular Weight Heparin

918003	RxNorm	1359868	dalteparin sodium 25000 UNT/ML Prefilled Syringe	Low Molecular Weight Heparin
918020	RxNorm	1359890	dalteparin sodium 25000 UNT/ML Prefilled Syringe [Fragmin]	Low Molecular Weight Heparin
918361	RxNorm	1360007	tinzaparin sodium 10000 UNT/ML Prefilled Syringe	Low Molecular Weight Heparin
918369	RxNorm	1360019	enoxaparin sodium 100 MG/ML Prefilled Syringe	Low Molecular Weight Heparin
918375	RxNorm	1360029	tinzaparin sodium 20000 UNT/ML Prefilled Syringe	Low Molecular Weight Heparin
919017	RxNorm	1360220	enoxaparin sodium 150 MG/ML Prefilled Syringe [Lovenox]	Low Molecular Weight Heparin
920149	RxNorm	1360465	dalteparin sodium 12500 UNT/ML Prefilled Syringe	Low Molecular Weight Heparin
927611	RxNorm	1359477	enoxaparin sodium 100 MG/ML Prefilled Syringe [Lovenox]	Low Molecular Weight Heparin
928397	RxNorm	1359549	dalteparin sodium 12500 UNT/ML Prefilled Syringe [Fragmin]	Low Molecular Weight Heparin
928631	RxNorm	1359604	dalteparin sodium 10000 UNT/ML Prefilled Syringe [Fragmin]	Low Molecular Weight Heparin
933777	RxNorm	151903	Innohep	Low Molecular Weight Heparin
957566	RxNorm	201488	tinzaparin sodium 10000 UNT/ML Injectable Solution [Innohep]	Low Molecular Weight Heparin
974704	RxNorm	225036	Lovenox	Low Molecular Weight Heparin
984713	RxNorm	281554	Fragmin	Low Molecular Weight Heparin
995068	RxNorm	362788	enoxaparin Injectable Solution [Lovenox]	Low Molecular Weight Heparin
995921	RxNorm	363000	tinzaparin Injectable Solution [Innohep]	Low Molecular Weight Heparin
996029	RxNorm	363135	dalteparin Injectable Solution [Fragmin]	Low Molecular Weight Heparin
1002941	RxNorm	374638	tinzaparin Injectable Solution	Low Molecular Weight Heparin
1003400	RxNorm	371679	dalteparin Injectable Solution	Low Molecular Weight Heparin
1004851	RxNorm	372012	enoxaparin Injectable Solution	Low Molecular Weight Heparin
528324	ICD10CM	I21.01	ST elevation (STEMI) myocardial infarction involving left main coronary artery	MI Type I Only
528325	ICD10CM	I21.02	ST elevation (STEMI) myocardial infarction involving left anterior descending coronary artery	MI Type I Only
528326	ICD10CM	I21.09	ST elevation (STEMI) myocardial infarction involving other coronary artery of anterior wall	MI Type I Only
528328	ICD10CM	I21.11	ST elevation (STEMI) myocardial infarction involving right coronary artery	MI Type I Only

528329	ICD10CM	I21.19	ST elevation (STEMI) myocardial infarction involving other coronary artery of inferior wall	MI Type I Only
528331	ICD10CM	I21.21	ST elevation (STEMI) myocardial infarction involving left circumflex coronary artery	MI Type I Only
528332	ICD10CM	I21.29	ST elevation (STEMI) myocardial infarction involving other sites	MI Type I Only
528333	ICD10CM	I21.3	ST elevation (STEMI) myocardial infarction of unspecified site	MI Type I Only
528334	ICD10CM	I21.4	Non-ST elevation (NSTEMI) myocardial infarction	MI Type I Only
528335	ICD10CM	I21.9	Acute myocardial infarction, unspecified	MI Type I Only
528340	ICD10CM	I22.0	Subsequent ST elevation (STEMI) myocardial infarction of anterior wall	MI Type I Only
528341	ICD10CM	I22.1	Subsequent ST elevation (STEMI) myocardial infarction of inferior wall	MI Type I Only
528342	ICD10CM	I22.2	Subsequent non-ST elevation (NSTEMI) myocardial infarction	MI Type I Only
528343	ICD10CM	I22.8	Subsequent ST elevation (STEMI) myocardial infarction of other sites	MI Type I Only
528344	ICD10CM	I22.9	Subsequent ST elevation (STEMI) myocardial infarction of unspecified site	MI Type I Only
1174989	ICD9CM	410	Acute myocardial infarction	MI Type I Only
1174992	ICD9CM	410.01	Acute myocardial infarction of anterolateral wall, initial episode of care	MI Type I Only
1174993	ICD9CM	410.02	Acute myocardial infarction of anterolateral wall, subsequent episode of care	MI Type I Only
1174994	ICD9CM	410.1	Acute myocardial infarction, of other anterior wall	MI Type I Only
1174996	ICD9CM	410.11	Acute myocardial infarction of other anterior wall, initial episode of care	MI Type I Only
1174997	ICD9CM	410.12	Acute myocardial infarction of other anterior wall, subsequent episode of care	MI Type I Only
1174998	ICD9CM	410.2	Acute myocardial infarction, of inferolateral wall	MI Type I Only
1175000	ICD9CM	410.21	Acute myocardial infarction of inferolateral wall, initial episode of care	MI Type I Only
1175001	ICD9CM	410.22	Acute myocardial infarction of inferolateral wall, subsequent episode of care	MI Type I Only

1175002	ICD9CM	410.3	Acute myocardial infarction, of inferoposterior wall	MI Type I Only
1175004	ICD9CM	410.31	Acute myocardial infarction of inferoposterior wall, initial episode of care	MI Type I Only
1175005	ICD9CM	410.32	Acute myocardial infarction of inferoposterior wall, subsequent episode of care	MI Type I Only
1175006	ICD9CM	410.4	Acute myocardial infarction, of other inferior wall	MI Type I Only
1175008	ICD9CM	410.41	Acute myocardial infarction of other inferior wall, initial episode of care	MI Type I Only
1175009	ICD9CM	410.42	Acute myocardial infarction of other inferior wall, subsequent episode of care	MI Type I Only
1175010	ICD9CM	410.5	Acute myocardial infarction, of other lateral wall	MI Type I Only
1175012	ICD9CM	410.51	Acute myocardial infarction of other lateral wall, initial episode of care	MI Type I Only
1175013	ICD9CM	410.52	Acute myocardial infarction of other lateral wall, subsequent episode of care	MI Type I Only
1175014	ICD9CM	410.6	Acute myocardial infarction, true posterior wall infarction	MI Type I Only
1175016	ICD9CM	410.61	True posterior wall infarction, initial episode of care	MI Type I Only
1175017	ICD9CM	410.62	True posterior wall infarction, subsequent episode of care	MI Type I Only
1175018	ICD9CM	410.7	Acute myocardial infarction, subendocardial infarction	MI Type I Only
1175020	ICD9CM	410.71	Subendocardial infarction, initial episode of care	MI Type I Only
1175021	ICD9CM	410.72	Subendocardial infarction, subsequent episode of care	MI Type I Only
1175022	ICD9CM	410.8	Acute myocardial infarction, of other specified sites	MI Type I Only
1175024	ICD9CM	410.81	Acute myocardial infarction of other specified sites, initial episode of care	MI Type I Only
1175025	ICD9CM	410.82	Acute myocardial infarction of other specified sites, subsequent episode of care	MI Type I Only
1175026	ICD9CM	410.9	Acute myocardial infarction, unspecified site	MI Type I Only
1175028	ICD9CM	410.91	Acute myocardial infarction of unspecified site, initial episode of care	MI Type I Only

1175029	ICD9CM	410.92	Acute myocardial infarction of unspecified site, subsequent episode of care	MI Type I Only
2927986	SNOMED CT	1.204E+09	Acute inferior non-ST segment elevation myocardial infarction of right ventricle	MI Type I Only
2927974	SNOMED CT	1.204E+09	Acute anterior non-ST segment elevation myocardial infarction with right ventricular involvement	MI Type I Only
2927973	SNOMED CT	1.204E+09	Acute anterior non-ST segment elevation myocardial infarction	MI Type I Only
2927985	SNOMED CT	1.204E+09	Acute inferior non-ST segment elevation myocardial infarction	MI Type I Only
2927994	SNOMED CT	1.204E+09	Acute non-ST segment elevation myocardial infarction of right ventricle	MI Type I Only
1574	SNOMED CT	1755008	Old myocardial infarction	MI Type I Only
178	SNOMED CT	282006	Acute myocardial infarction of basal-lateral wall	MI Type I Only
9601	SNOMED CT	10273003	Acute infarction of papillary muscle	MI Type I Only
14989	SNOMED CT	15990001	Acute myocardial infarction of posterolateral wall	MI Type I Only
392607	SNOMED CT	703164000	Acute anterior ST segment elevation myocardial infarction	MI Type I Only
392608	SNOMED CT	703165004	Acute ST segment elevation myocardial infarction of anterior wall involving right ventricle	MI Type I Only
392652	SNOMED CT	703209002	Subsequent ST segment elevation myocardial infarction of inferior wall	MI Type I Only
392653	SNOMED CT	703210007	Subsequent ST segment elevation myocardial infarction of anterior wall	MI Type I Only
392654	SNOMED CT	703211006	Subsequent ST segment elevation myocardial infarction	MI Type I Only
392655	SNOMED CT	703212004	Acute myocardial infarction during procedure	MI Type I Only
392656	SNOMED CT	703213009	Acute ST segment elevation myocardial infarction of inferior wall	MI Type I Only
392694	SNOMED CT	703251009	Acute myocardial infarction of inferior wall involving right ventricle	MI Type I Only
392695	SNOMED CT	703252002	Acute myocardial infarction of anterior wall involving right ventricle	MI Type I Only

392696	SNOMED CT	703253007	Acute ST segment elevation myocardial infarction of inferior wall involving right ventricle	MI Type I Only
392802	SNOMED CT	703360004	Subsequent non-ST segment elevation myocardial infarction	MI Type I Only
444626	SNOMED CT	836293000	Acute myocardial infarction of right ventricle	MI Type I Only
444627	SNOMED CT	836294006	Acute myocardial infarction of apex of heart	MI Type I Only
444628	SNOMED CT	836295007	Acute myocardial infarction of inferolateral wall with posterior extension	MI Type I Only
445049	SNOMED CT	840309000	Acute ST segment elevation myocardial infarction due to proximal left anterior descending coronary artery occlusion	MI Type I Only
445051	SNOMED CT	840312002	Acute ST segment elevation myocardial infarction due to mid left anterior descending coronary artery occlusion	MI Type I Only
445054	SNOMED CT	840316004	Acute ST segment elevation myocardial infarction due to distal left anterior descending coronary artery occlusion	MI Type I Only
445280	SNOMED CT	840609007	Acute ST segment elevation myocardial infarction due to occlusion of anterior descending branch of left coronary artery	MI Type I Only
445344	SNOMED CT	840680009	Acute ST segment elevation myocardial infarction due to occlusion of septal branch of anterior descending branch of left coronary artery	MI Type I Only
445464	SNOMED CT	846668006	Acute ST segment elevation myocardial infarction due to occlusion of diagonal branch of anterior descending branch of left coronary artery	MI Type I Only
445474	SNOMED CT	846683001	Acute ST segment elevation myocardial infarction due to occlusion of intermediate artery	MI Type I Only
446318	SNOMED CT	868214006	Acute ST segment elevation myocardial infarction due to occlusion of proximal portion of right coronary artery	MI Type I Only
446321	SNOMED CT	868217004	Acute ST segment elevation myocardial infarction due to occlusion of distal portion of right coronary artery	MI Type I Only

446323	SNOMED CT	868220007	Acute ST segment elevation myocardial infarction due to occlusion of mid portion of right coronary artery	MI Type I Only
446327	SNOMED CT	868224003	Acute ST segment elevation myocardial infarction due to occlusion of marginal branch of right coronary artery	MI Type I Only
446328	SNOMED CT	868225002	Acute ST segment elevation myocardial infarction due to occlusion of posterior descending branch of right coronary artery	MI Type I Only
446329	SNOMED CT	868226001	Acute ST segment elevation myocardial infarction due to occlusion of posterior lateral branch of right coronary artery	MI Type I Only
449814	SNOMED CT	1.753E+13	Acute myocardial infarction due to left coronary artery occlusion	MI Type I Only
450059	SNOMED CT	2.331E+13	Acute myocardial infarction due to right coronary artery occlusion	MI Type I Only
452418	SNOMED CT	2.86E+14	Acute ST segment elevation myocardial infarction involving left anterior descending coronary artery	MI Type I Only
454578	SNOMED CT	3.8E+14	Subendocardial myocardial infarction	MI Type I Only
463271	SNOMED CT	1.571E+16	Acute ST segment elevation myocardial infarction of posterolateral wall	MI Type I Only
463276	SNOMED CT	1.571E+16	Acute ST segment elevation myocardial infarction of septum	MI Type I Only
492714	SNOMED CT	32574007	Past myocardial infarction diagnosed on electrocardiogram AND/OR other special investigation, but currently presenting no symptoms	MI Type I Only
290806	SNOMED CT	311792005	Postoperative transmural myocardial infarction of anterior wall	MI Type I Only
290807	SNOMED CT	311793000	Postoperative transmural myocardial infarction of inferior wall	MI Type I Only
290810	SNOMED CT	311796008	Postoperative subendocardial myocardial infarction	MI Type I Only
293045	SNOMED CT	314207007	Non-Q wave myocardial infarction	MI Type I Only
328447	SNOMED CT	394710008	First myocardial infarction	MI Type I Only
334726	SNOMED CT	401303003	Acute ST segment elevation myocardial infarction	MI Type I Only

334737	SNOMED CT	401314000	Acute non-ST segment elevation myocardial infarction	MI Type I Only
350151	SNOMED CT	418044006	Myocardial infarction in recovery phase	MI Type I Only
359859	SNOMED CT	428196007	Mixed myocardial ischemia and infarction	MI Type I Only
360383	SNOMED CT	428752002	Recent myocardial infarction	MI Type I Only
2647930	SNOMED CT	1.163E+09	Postoperative acute myocardial infarction	MI Type I Only
215993	SNOMED CT	233825009	Acute Q wave infarction - anteroseptal	MI Type I Only
215994	SNOMED CT	233826005	Acute non-Q wave infarction - anteroseptal	MI Type I Only
215995	SNOMED CT	233827001	Acute Q wave infarction - anterolateral	MI Type I Only
215996	SNOMED CT	233828006	Acute non-Q wave infarction - anterolateral	MI Type I Only
215997	SNOMED CT	233829003	Acute Q wave infarction - inferior	MI Type I Only
215998	SNOMED CT	233830008	Acute non-Q wave infarction - inferior	MI Type I Only
215999	SNOMED CT	233831007	Acute Q wave infarction - inferolateral	MI Type I Only
216000	SNOMED CT	233832000	Acute non-Q wave infarction - inferolateral	MI Type I Only
216001	SNOMED CT	233833005	Acute Q wave infarction - lateral	MI Type I Only
216002	SNOMED CT	233834004	Acute non-Q wave infarction - lateral	MI Type I Only
216003	SNOMED CT	233835003	Acute widespread myocardial infarction	MI Type I Only
216004	SNOMED CT	233836002	Acute Q wave infarction - widespread	MI Type I Only
216005	SNOMED CT	233837006	Acute non-Q wave infarction - widespread	MI Type I Only
216006	SNOMED CT	233838001	Acute posterior myocardial infarction	MI Type I Only
216007	SNOMED CT	233839009	Old anterior myocardial infarction	MI Type I Only
216008	SNOMED CT	233840006	Old inferior myocardial infarction	MI Type I Only
216009	SNOMED CT	233841005	Old lateral myocardial infarction	MI Type I Only
216010	SNOMED CT	233842003	Old posterior myocardial infarction	MI Type I Only
216011	SNOMED CT	233843008	Silent myocardial infarction	MI Type I Only
284568	SNOMED CT	304914007	Acute Q wave myocardial infarction	MI Type I Only
286660	SNOMED CT	307140009	Acute non-Q wave infarction	MI Type I Only

2932311	SNOMED CT	1.209E+09	Subsequent anterior non-ST segment elevation myocardial infarction	MI Type I Only
2932312	SNOMED CT	1.209E+09	Subsequent inferior non-ST segment elevation myocardial infarction	MI Type I Only
66118	SNOMED CT	70211005	Acute myocardial infarction of anterolateral wall	MI Type I Only
66317	SNOMED CT	70422006	Acute subendocardial infarction	MI Type I Only
66867	SNOMED CT	70998009	Acute myocardial infarction of posterobasal wall	MI Type I Only
69511	SNOMED CT	73795002	Acute myocardial infarction of inferior wall	MI Type I Only
72136	SNOMED CT	76593002	Acute myocardial infarction of inferoposterior wall	MI Type I Only
74399	SNOMED CT	79009004	Acute myocardial infarction of septum	MI Type I Only
115576	SNOMED CT	129574000	Postoperative myocardial infarction	MI Type I Only
177455	SNOMED CT	194802003	True posterior myocardial infarction	MI Type I Only
177462	SNOMED CT	194809007	Acute myocardial infarction of atrium	MI Type I Only
177509	SNOMED CT	194856005	Subsequent myocardial infarction	MI Type I Only
177510	SNOMED CT	194857001	Subsequent myocardial infarction of anterior wall	MI Type I Only
177511	SNOMED CT	194858006	Subsequent myocardial infarction of inferior wall	MI Type I Only
1203199	SNOMED CT	879955009	Myocardial infarction with non-obstructive coronary artery	MI Type I Only
1204021	SNOMED CT	896689003	Acute myocardial infarction due to occlusion of circumflex branch of left coronary artery	MI Type I Only
1204023	SNOMED CT	896691006	Acute ST segment elevation myocardial infarction due to occlusion of circumflex branch of left coronary artery	MI Type I Only
1204025	SNOMED CT	896696001	Acute ST segment elevation myocardial infarction of apex of heart	MI Type I Only
1204026	SNOMED CT	896697005	Acute ST segment elevation myocardial infarction of right ventricle	MI Type I Only
20963	SNOMED CT	22298006	Myocardial infarction	MI Type I Only
28486	SNOMED CT	30277009	Rupture of ventricle due to acute myocardial infarction	MI Type I Only
40079	SNOMED CT	42531007	Microinfarct of heart	MI Type I Only

49014	SNOMED CT	52035003	Acute anteroapical myocardial infarction	MI Type I Only
51182	SNOMED CT	54329005	Acute myocardial infarction of anterior wall	MI Type I Only
53737	SNOMED CT	57054005	Acute myocardial infarction	MI Type I Only
55196	SNOMED CT	58612006	Acute myocardial infarction of lateral wall	MI Type I Only
55619	SNOMED CT	59063002	Acute myocardial infarction of apical-lateral wall	MI Type I Only
59034	SNOMED CT	62695002	Acute anteroseptal myocardial infarction	MI Type I Only
60862	SNOMED CT	64627002	Acute myocardial infarction of high lateral wall	MI Type I Only
61731	SNOMED CT	65547006	Acute myocardial infarction of inferolateral wall	MI Type I Only
462249	SNOMED CT	1.224E+16	Acute ST segment elevation myocardial infarction of inferolateral wall	MI Type I Only
462250	SNOMED CT	1.224E+16	Acute ST segment elevation myocardial infarction of inferoposterior wall	MI Type I Only
463272	SNOMED CT	1.571E+16	Acute ST segment elevation myocardial infarction of anterolateral wall	MI Type I Only
463273	SNOMED CT	1.571E+16	Acute ST segment elevation myocardial infarction of lateral wall	MI Type I Only
463274	SNOMED CT	1.571E+16	Acute ST segment elevation myocardial infarction of anteroseptal wall	MI Type I Only
463275	SNOMED CT	1.571E+16	Acute ST segment elevation myocardial infarction of posterior wall	MI Type I Only
463277	SNOMED CT	1.571E+16	Acute ST segment elevation myocardial infarction of posterobasal wall	MI Type I Only
464014	SNOMED CT	1.596E+16	Acute ST segment elevation myocardial infarction of anteroapical wall	MI Type I Only
464015	SNOMED CT	1.596E+16	Acute ST segment elevation myocardial infarction due to occlusion of circumflex coronary artery	MI Type I Only
465478	SNOMED CT	1.684E+16	Myocardial infarction due to demand ischemia	MI Type I Only
517083	SNOMED CT	1.571E+16	Acute ST segment elevation myocardial infarction involving left main coronary artery	MI Type I Only

517084	SNOMED CT	1.571E+16	Acute STEMI (ST elevation myocardial infarction) due to RCA (right coronary artery) occlusion	MI Type I Only
1202807	SNOMED CT	7.265E+17	Myocardial infarction due to atherothrombotic coronary artery disease	MI Type I Only
813274	LOINC	42637-9	Natriuretic peptide B [Mass/volume] in Blood	Natriuretic Peptide
801789	LOINC	30934-4	Natriuretic peptide B [Mass/volume] in Serum or Plasma	Natriuretic Peptide
804617	LOINC	33762-6	Natriuretic peptide.B prohormone N-Terminal [Mass/volume] in Serum or Plasma	Natriuretic Peptide
804618	LOINC	33763-4	Natriuretic peptide.B prohormone N-Terminal [Moles/volume] in Serum or Plasma	Natriuretic Peptide
1065593	Truveta	1065593	Entered in Error	Drug Not Administered
1065595	Truveta	1065595	Not Done	Drug Not Administered
1065627	Truveta	1065627	Cancelled	Drug Not Administered
1065629	Truveta	1065629	Draft	Drug Not Administered
1040874	RxNorm	76895	reteplase	tpa
1045710	RxNorm	8410	alteplase	tpa
981706	RxNorm	259280	tenecteplase	tpa
889228	RxNorm	1155608	alteplase Injectable Product	tpa
890138	RxNorm	1164230	tenecteplase Injectable Product	tpa
893597	RxNorm	1164834	reteplase Injectable Product	tpa
895065	RxNorm	1169913	Activase Injectable Product	tpa
901708	RxNorm	1182533	Retavase Injectable Product	tpa
1008649	RxNorm	393311	tenecteplase 4 MG/ML	tpa
1015071	RxNorm	387020	tenecteplase 4 MG/ML Injectable Solution	tpa
1040094	RxNorm	763138	reteplase 10 UNT Injection	tpa
1040095	RxNorm	763141	reteplase 10 UNT Injection [Retavase]	tpa
1073118	RxNorm	211256	alteplase 1 MG/ML Injectable Solution [Activase]	tpa
1073513	RxNorm	337468	alteplase 1 MG/ML	tpa
1077284	RxNorm	352008	Alteplase 0.2 MG/ML Injectable Solution [Cathflo Activase]	tpa
1077629	RxNorm	333784	Tenecteplase 5 MG/ML	tpa
1077995	RxNorm	353353	Alteplase 0.2 MG/ML	tpa
1078576	RxNorm	574861	Tenecteplase 5 MG/ML [Tnkase]	tpa
1080119	RxNorm	352508	Cathflo Activase	tpa
1082162	RxNorm	571707	alteplase 1 MG/ML [Activase]	tpa
1085593	RxNorm	308056	alteplase 1 MG/ML Injectable Solution	tpa

1087368	RxNorm	575752	Alteplase 0.2 MG/ML [Cathflo Activase]	tpa
1090349	RxNorm	150799	alteplase 1 MG/ML Injectable Solution [Actilyse]	tpa
1098678	RxNorm	763139	Reteplase 1 UNT/ML [Retavase]	tpa
1100200	RxNorm	763137	Reteplase 1 UNT/ML	tpa
1107656	RxNorm	544823	alteplase 1 MG/ML [Cathflo Activase]	tpa
1108730	RxNorm	544825	alteplase 1 MG/ML Injectable Solution [Cathflo Activase]	tpa
1110128	RxNorm	564796	alteplase 1 MG/ML [Actilyse]	tpa
1111384	RxNorm	562803	Alteplase 0.2 MG/ML Injectable Solution	tpa
1114528	RxNorm	363509	alteplase Injectable Solution [Actilyse]	tpa
1114586	RxNorm	362613	alteplase Injectable Solution [Activase]	tpa
1115103	RxNorm	337473	Alteplase 10 MG	tpa
1115794	RxNorm	250846	Alteplase 10 MG Injectable Solution	tpa
1115795	RxNorm	370818	alteplase Injectable Solution	tpa
1122529	RxNorm	1176394	Cathflo Activase Injectable Product	tpa
1125628	RxNorm	1169906	Actilyse Injectable Product	tpa
1139077	RxNorm	334490	Reteplase 10.8 UNT	tpa
1139481	RxNorm	249339	Reteplase 10.8 UNT Injectable Solution	tpa
1142869	RxNorm	362670	Tenecteplase Injectable Solution [Tnkase]	tpa
1143035	RxNorm	544824	alteplase Injectable Solution [Cathflo Activase]	tpa
1145216	RxNorm	377197	Reteplase Injectable Solution	tpa
1152690	RxNorm	763140	Reteplase Injectable Solution [Retavase]	tpa
915709	RxNorm	1177312	Tnkase Injectable Product	tpa
947581	RxNorm	1809070	tenecteplase 50 MG	tpa
947582	RxNorm	1809071	tenecteplase Injection	tpa
947583	RxNorm	1809073	tenecteplase 50 MG [Tnkase]	tpa
947584	RxNorm	1809074	tenecteplase Injection [Tnkase]	tpa
951568	RxNorm	1804797	alteplase 100 MG	tpa
951569	RxNorm	1804798	alteplase Injection	tpa
951570	RxNorm	1804799	alteplase 100 MG Injection	tpa
951571	RxNorm	1804800	alteplase 100 MG [Activase]	tpa
951572	RxNorm	1804801	alteplase Injection [Activase]	tpa
951573	RxNorm	1804802	alteplase 100 MG Injection [Activase]	tpa
951574	RxNorm	1804803	alteplase 50 MG	tpa
951575	RxNorm	1804804	alteplase 50 MG Injection	tpa
951576	RxNorm	1804805	alteplase 50 MG [Activase]	tpa
951577	RxNorm	1804806	alteplase 50 MG Injection [Activase]	tpa

951831	RxNorm	1867708	reteplase 10 UNT	tpa
951832	RxNorm	1867709	reteplase Injection	tpa
951833	RxNorm	1867711	reteplase 10 UNT [Retavase]	tpa
951834	RxNorm	1867712	reteplase Injection [Retavase]	tpa
959343	RxNorm	204210	Activase	tpa
970743	RxNorm	219642	Retavase	tpa
981180	RxNorm	284422	tenecteplase 50 MG Injection [Tnkase]	tpa
981909	RxNorm	284879	Tnkase	tpa
985932	RxNorm	313212	tenecteplase 50 MG Injection	tpa
1003912	RxNorm	377078	tenecteplase Injectable Solution	tpa
813394	LOINC	42757-5	Troponin I.cardiac [Mass/volume] in Blood	Troponin
819041	LOINC	48426-1	Troponin T.cardiac [Presence] in Blood	Troponin
834302	LOINC	64110-0	Interpretation	Troponin
837269	LOINC	67151-1	Troponin T.cardiac [Mass/volume] in Serum or Plasma by High sensitivity method	Troponin
859144	LOINC	89579-7	Troponin I.cardiac [Mass/volume] in Serum or Plasma by High sensitivity method	Troponin
777899	LOINC	6598-7	Troponin T.cardiac [Mass/volume] in Serum or Plasma	Troponin
782108	LOINC	10839-9	Troponin I.cardiac [Mass/volume] in Serum or Plasma	Troponin
375547	SNOMED CT	447638001	Map source concept cannot be classified with available data	Troponin
977777	RxNorm	235473	heparin, porcine	Unfractionated heparin
1104419	RxNorm	247801	Heparin 0.16 UNT/MG / Zinc Sulfate 0.005 MG/MG Topical Gel	Unfractionated heparin
1104999	RxNorm	849654	Heparin sodium 10 UNT/ML [Lok-Pak]	Unfractionated heparin
1105169	RxNorm	849659	Heparin sodium 100 UNT/ML [Lok-Pak]	Unfractionated heparin
1106353	RxNorm	331800	Heparin 40 UNT/ML	Unfractionated heparin
1106547	RxNorm	250363	Heparin 0.2 UNT/MG Topical Gel	Unfractionated heparin
1106961	RxNorm	791150	Lok-Pak	Unfractionated heparin
1107569	RxNorm	1165226	Glucose / Heparin Injectable Product	Unfractionated heparin
1107999	RxNorm	542812	Hepflush	Unfractionated heparin
1109707	RxNorm	563644	Heparin 10000 UNT/ML [Unihep]	Unfractionated heparin
1109909	RxNorm	1361849	heparin, porcine Prefilled Syringe	Unfractionated heparin
1109977	RxNorm	563643	Heparin 5000 UNT/ML [Unihep]	Unfractionated heparin

1110016	RxNorm	1362955	heparin sodium, porcine 75 UNT/ML	Unfractionated heparin
1110159	RxNorm	1361042	heparin sodium, porcine 10 UNT/ML [PosiFlush]	Unfractionated heparin
1110448	RxNorm	1361043	heparin, porcine Injectable Solution [PosiFlush]	Unfractionated heparin
1110727	RxNorm	564756	Heparin 10000 UNT/ML [Minihep]	Unfractionated heparin
1110947	RxNorm	1361206	heparin sodium, porcine 100 UNT/ML [Lok-Pak]	Unfractionated heparin
1111105	RxNorm	1362959	heparin sodium, porcine 0.2 UNT/ML	Unfractionated heparin
1111225	RxNorm	564757	Heparin 10000 UNT/ML [Minihep Calcium]	Unfractionated heparin
1111521	RxNorm	564758	Heparin 25000 UNT/ML [Uniparin]	Unfractionated heparin
1111561	RxNorm	563645	Heparin 25000 UNT/ML [Unihep]	Unfractionated heparin
1111589	RxNorm	1361850	0.2 ML heparin sodium, porcine 25000 UNT/ML Prefilled Syringe	Unfractionated heparin
1111590	RxNorm	1362027	2 ML heparin sodium, porcine 1 UNT/ML Prefilled Syringe	Unfractionated heparin
1111591	RxNorm	1362051	10 ML heparin sodium, porcine 10 UNT/ML Prefilled Syringe	Unfractionated heparin
1111592	RxNorm	1362053	2.5 ML heparin sodium, porcine 10 UNT/ML Prefilled Syringe	Unfractionated heparin
1111974	RxNorm	1361028	heparin, porcine Injectable Solution	Unfractionated heparin
1111975	RxNorm	1361034	heparin, porcine Injectable Solution [Hep-Pak]	Unfractionated heparin
1111982	RxNorm	1362818	heparin sodium, porcine 6 UNT/ML Injectable Solution	Unfractionated heparin
1111983	RxNorm	1362956	heparin sodium, porcine 75 UNT/ML Injectable Solution	Unfractionated heparin
1111996	RxNorm	1361031	heparin, porcine Injectable Solution [Hep-Lock]	Unfractionated heparin
1112010	RxNorm	1361611	heparin sodium, porcine 5 UNT/ML Injectable Solution	Unfractionated heparin
1112017	RxNorm	1361851	heparin sodium, porcine 25000 UNT/ML Prefilled Syringe	Unfractionated heparin
1112018	RxNorm	1362964	heparin sodium, porcine 1 UNT/ML Injectable Solution	Unfractionated heparin
1112023	RxNorm	1361037	heparin, porcine Injectable Solution [Hepflush]	Unfractionated heparin
1112095	RxNorm	1362960	heparin sodium, porcine 0.2 UNT/ML Injectable Solution	Unfractionated heparin
1112080	RxNorm	1362024	1 ML heparin sodium, porcine 1 UNT/ML Prefilled Syringe	Unfractionated heparin
1112081	RxNorm	1362066	heparin sodium, porcine 2 UNT/ML Prefilled Syringe	Unfractionated heparin

1112089	RxNorm	1361597	heparin sodium, porcine 30 UNT/ML Injectable Solution	Unfractionated heparin
1112090	RxNorm	1361613	heparin sodium, porcine 50 UNT/ML Injectable Solution	Unfractionated heparin
1112092	RxNorm	1361854	heparin sodium, porcine 10000 UNT/ML Prefilled Syringe	Unfractionated heparin
1112096	RxNorm	1362963	heparin sodium, porcine 0.5 UNT/ML Injectable Solution	Unfractionated heparin
1112101	RxNorm	1361605	heparin sodium, porcine 4 UNT/ML Injectable Solution	Unfractionated heparin
1112104	RxNorm	1362076	heparin sodium, porcine 0.5 UNT/ML Prefilled Syringe	Unfractionated heparin
1112151	RxNorm	1361219	heparin sodium, porcine 100 UNT/ML Injectable Solution [PosiFlush]	Unfractionated heparin
1112172	RxNorm	1361032	heparin sodium, porcine 10 UNT/ML Injectable Solution [Hep-Lock]	Unfractionated heparin
1112173	RxNorm	1361041	heparin sodium, porcine 10 UNT/ML Injectable Solution [Lok-Pak]	Unfractionated heparin
1112179	RxNorm	1361039	heparin sodium, porcine 10 UNT/ML [Lok-Pak]	Unfractionated heparin
1112229	RxNorm	1362074	heparin sodium, porcine 0.5 UNT/ML	Unfractionated heparin
1112231	RxNorm	1361044	heparin sodium, porcine 10 UNT/ML Injectable Solution [PosiFlush]	Unfractionated heparin
1112232	RxNorm	1361050	heparin sodium, porcine 100 UNT/ML Injectable Solution [Hep-Lock]	Unfractionated heparin
1112284	RxNorm	1366636	6 ML heparin sodium, porcine 10 UNT/ML Prefilled Syringe	Unfractionated heparin
1112326	RxNorm	1361038	10 ML heparin sodium, porcine 10 UNT/ML Injection [Hepflush]	Unfractionated heparin
1112383	RxNorm	1362028	2.5 ML heparin sodium, porcine 1 UNT/ML Prefilled Syringe	Unfractionated heparin
1112384	RxNorm	1362057	1 ML heparin sodium, porcine 100 UNT/ML Prefilled Syringe	Unfractionated heparin
1112392	RxNorm	1362052	2 ML heparin sodium, porcine 10 UNT/ML Prefilled Syringe	Unfractionated heparin
1112393	RxNorm	1362065	3 ML heparin sodium, porcine 2 UNT/ML Prefilled Syringe	Unfractionated heparin
1112413	RxNorm	1362029	3 ML heparin sodium, porcine 1 UNT/ML Prefilled Syringe	Unfractionated heparin
1112414	RxNorm	1362055	5 ML heparin sodium, porcine 10 UNT/ML Prefilled Syringe	Unfractionated heparin
1112415	RxNorm	1362075	4 ML heparin sodium, porcine 0.5 UNT/ML Prefilled Syringe	Unfractionated heparin
1112574	RxNorm	1241816	Heparin sodium 0.2 UNT/ML	Unfractionated heparin

1112845	RxNorm	1361030	heparin sodium, porcine 10 UNT/ML [Hep-Lock]	Unfractionated heparin
1112846	RxNorm	1361049	heparin sodium, porcine 100 UNT/ML [Hep-Lock]	Unfractionated heparin
1113034	RxNorm	1361207	heparin sodium, porcine 100 UNT/ML Injectable Solution [Lok-Pak]	Unfractionated heparin
1113116	RxNorm	1361040	heparin, porcine Injectable Solution [Lok-Pak]	Unfractionated heparin
1113430	RxNorm	1361036	heparin sodium, porcine 10 UNT/ML [Hepflush]	Unfractionated heparin
1113431	RxNorm	1361218	heparin sodium, porcine 100 UNT/ML [PosiFlush]	Unfractionated heparin
1113467	RxNorm	1241814	Heparin sodium 0.25 UNT/ML	Unfractionated heparin
1113545	RxNorm	1362030	5 ML heparin sodium, porcine 1 UNT/ML Prefilled Syringe	Unfractionated heparin
1113546	RxNorm	1362048	1 ML heparin sodium, porcine 10 UNT/ML Prefilled Syringe	Unfractionated heparin
1115246	RxNorm	336708	Heparin 0.2 UNT/MG	Unfractionated heparin
1115291	RxNorm	1245687	Heparin sodium 8 UNT/ML	Unfractionated heparin
1115650	RxNorm	1241817	Heparin sodium 0.2 UNT/ML Injectable Solution	Unfractionated heparin
1115689	RxNorm	1241815	Heparin sodium 0.25 UNT/ML Injectable Solution	Unfractionated heparin
1116570	RxNorm	1245688	Heparin sodium 8 UNT/ML Injectable Solution	Unfractionated heparin
1117699	RxNorm	1160318	Heparin Topical Product	Unfractionated heparin
1118054	RxNorm	1160315	Heparin Inhalant Product	Unfractionated heparin
1118055	RxNorm	1160316	Heparin Injectable Product	Unfractionated heparin
1118223	RxNorm	1160314	Heparin / Zinc Sulfate Topical Product	Unfractionated heparin
1118224	RxNorm	1160317	Heparin Irrigation Product	Unfractionated heparin
1121373	RxNorm	1490490	heparin, porcine Prefilled Syringe [PosiFlush]	Unfractionated heparin
1121440	RxNorm	1179643	Lok-Pak Injectable Product	Unfractionated heparin
1121685	RxNorm	1490491	3 ML heparin sodium, porcine 100 UNT/ML Prefilled Syringe [PosiFlush]	Unfractionated heparin
1121716	RxNorm	1490493	5 ML heparin sodium, porcine 100 UNT/ML Prefilled Syringe [PosiFlush]	Unfractionated heparin
1122348	RxNorm	1168383	Hep-Lock Injectable Product	Unfractionated heparin
1122425	RxNorm	1490492	heparin sodium, porcine 100 UNT/ML Prefilled Syringe [PosiFlush]	Unfractionated heparin
1123703	RxNorm	1169105	Hepflush Injectable Product	Unfractionated heparin
1124716	RxNorm	1657983	heparin, porcine Cartridge	Unfractionated heparin
1124884	RxNorm	1656599	5 ML heparin sodium, porcine 10 UNT/ML Prefilled Syringe [PosiFlush]	Unfractionated heparin

1125339	RxNorm	1656596	heparin sodium, porcine 10 UNT/ML Prefilled Syringe [PosiFlush]	Unfractionated heparin
1125738	RxNorm	1656759	heparin, porcine Injection	Unfractionated heparin
1126034	RxNorm	1656764	heparin sodium, porcine 10 UNT/ML Injection [Hepflush]	Unfractionated heparin
1127494	RxNorm	1656761	heparin, porcine Injection [Hepflush]	Unfractionated heparin
1128922	RxNorm	1184078	PosiFlush Injectable Product	Unfractionated heparin
1129220	RxNorm	1656595	3 ML heparin sodium, porcine 10 UNT/ML Prefilled Syringe [PosiFlush]	Unfractionated heparin
1131352	RxNorm	244242	Ergot preparation 0.5 MG / Heparin 5000 UNT Injectable Solution	Unfractionated heparin
1131416	RxNorm	376682	Ergot preparation / Heparin Injectable Solution	Unfractionated heparin
1132009	RxNorm	440614	HEPARIN, PORK 0.3 UNT/MG	Unfractionated heparin
1132010	RxNorm	334931	HEPARIN, PORK 1 UNT/ML	Unfractionated heparin
1132011	RxNorm	334938	HEPARIN, PORK 15000 UNT/ML	Unfractionated heparin
1132012	RxNorm	440613	HEPARIN, PORK 2500 UNT/ML	Unfractionated heparin
1132013	RxNorm	334940	HEPARIN, PORK 40000 UNT/ML	Unfractionated heparin
1132014	RxNorm	452789	HEPARIN, PORK 66 UNT/ML	Unfractionated heparin
1132235	RxNorm	415890	HEPARIN, PORK 0.3 UNT/MG Topical Gel	Unfractionated heparin
1132236	RxNorm	417892	HEPARIN, PORK 0.6 UNT/MG Topical Cream	Unfractionated heparin
1132237	RxNorm	418982	HEPARIN, PORK 1 UNT/MG Topical Ointment	Unfractionated heparin
1132238	RxNorm	418831	HEPARIN, PORK 1.3 UNT/MG Topical Ointment	Unfractionated heparin
1132239	RxNorm	418828	HEPARIN, PORK 1.8 UNT/MG Topical Ointment	Unfractionated heparin
1132268	RxNorm	440617	HEPARIN, PORK 0.05 UNT/MG	Unfractionated heparin
1132357	RxNorm	440610	HEPARIN, PORK 0.15 UNT/MG	Unfractionated heparin
1132358	RxNorm	440606	HEPARIN, PORK 1 UNT/MG	Unfractionated heparin
1132359	RxNorm	440612	HEPARIN, PORK 1.8 UNT/MG	Unfractionated heparin
1132360	RxNorm	334932	HEPARIN, PORK 10 UNT/ML	Unfractionated heparin
1132361	RxNorm	334937	HEPARIN, PORK 10000 UNT/ML	Unfractionated heparin
1132376	RxNorm	440607	HEPARIN, PORK Topical Gel	Unfractionated heparin
1132519	RxNorm	334934	HEPARIN, PORK 2 UNT/ML	Unfractionated heparin
1132520	RxNorm	334925	HEPARIN, PORK 25000 UNT/ML	Unfractionated heparin
1132521	RxNorm	334935	HEPARIN, PORK 40 UNT/ML	Unfractionated heparin
1132522	RxNorm	440304	HEPARIN, PORK 75 UNT/ML	Unfractionated heparin
1132537	RxNorm	104458	Heparin 1000 UNT/ML Injectable Solution [Unihep]	Unfractionated heparin
1132538	RxNorm	333682	Heparin 12500 UNT/ML	Unfractionated heparin
1132539	RxNorm	329811	Heparin 3000 UNT/ML	Unfractionated heparin
1132540	RxNorm	329813	Heparin 50 UNT/ML	Unfractionated heparin

1132855	RxNorm	418985	HEPARIN, PORK 0.05 UNT/MG Topical Ointment	Unfractionated heparin
1132856	RxNorm	244238	HEPARIN, PORK 1 UNT/ML Injectable Solution	Unfractionated heparin
1132857	RxNorm	244239	HEPARIN, PORK 2 UNT/ML Injectable Solution	Unfractionated heparin
1132858	RxNorm	436786	HEPARIN, PORK 66 UNT/ML Injectable Solution	Unfractionated heparin
1132867	RxNorm	440615	HEPARIN, PORK Topical Cream	Unfractionated heparin
1132895	RxNorm	415891	HEPARIN, PORK 0.4 UNT/MG Topical Gel	Unfractionated heparin
1132896	RxNorm	418829	HEPARIN, PORK 0.6 UNT/MG Topical Ointment	Unfractionated heparin
1132897	RxNorm	238729	HEPARIN, PORK 10000 UNT/ML Injectable Solution	Unfractionated heparin
1132898	RxNorm	238731	HEPARIN, PORK 40000 UNT/ML Injectable Solution	Unfractionated heparin
1132948	RxNorm	418984	HEPARIN, PORK 0.5 UNT/MG Topical Ointment	Unfractionated heparin
1132949	RxNorm	415888	HEPARIN, PORK 0.6 UNT/MG Topical Gel	Unfractionated heparin
1132950	RxNorm	415886	HEPARIN, PORK 1.5 UNT/MG Topical Gel	Unfractionated heparin
1132951	RxNorm	244231	HEPARIN, PORK 100 UNT/ML Injectable Solution	Unfractionated heparin
1132952	RxNorm	244233	HEPARIN, PORK 15000 UNT/ML Injectable Solution	Unfractionated heparin
1132953	RxNorm	245578	HEPARIN, PORK 20000 UNT Injectable Solution	Unfractionated heparin
1132972	RxNorm	415885	HEPARIN, PORK 1 UNT/MG Topical Gel	Unfractionated heparin
1132973	RxNorm	244230	HEPARIN, PORK 10 UNT/ML Injectable Solution	Unfractionated heparin
1132974	RxNorm	244232	HEPARIN, PORK 12500 UNT/ML Injectable Solution	Unfractionated heparin
1132975	RxNorm	238727	HEPARIN, PORK 2500 UNT/ML Injectable Solution	Unfractionated heparin
1132982	RxNorm	361835	HEPARIN, PORK Injectable Solution	Unfractionated heparin
1133029	RxNorm	440616	HEPARIN, PORK 0.4 UNT/MG	Unfractionated heparin
1133030	RxNorm	334933	HEPARIN, PORK 100 UNT/ML	Unfractionated heparin
1133031	RxNorm	334936	HEPARIN, PORK 1000 UNT/ML	Unfractionated heparin
1133052	RxNorm	360844	Heparin 1 UNT/ML	Unfractionated heparin
1133053	RxNorm	209332	Heparin 10 UNT/ML Injectable Solution [Hep-Pak CVC]	Unfractionated heparin
1133054	RxNorm	329804	Heparin 100 UNT/ML	Unfractionated heparin
1133060	RxNorm	440619	HEPARIN, PORK 0.6 UNT/MG	Unfractionated heparin
1133061	RxNorm	440609	HEPARIN, PORK 1.3 UNT/MG	Unfractionated heparin
1133062	RxNorm	334944	HEPARIN, PORK 12500 UNT/ML	Unfractionated heparin
1133063	RxNorm	361836	HEPARIN, PORK 2000 UNT/ML	Unfractionated heparin

1133078	RxNorm	310710	Heparin 1000 UNT/ML Injectable Solution	Unfractionated heparin
1133082	RxNorm	310743	Heparin 8.3 UNT/ML Injectable Solution	Unfractionated heparin
1133083	RxNorm	363228	Heparin Injectable Solution [Hep-Lock]	Unfractionated heparin
1133084	RxNorm	385742	Heparin Injectable Solution [Unihep]	Unfractionated heparin
1133085	RxNorm	378055	Heparin Topical Gel	Unfractionated heparin
1133093	RxNorm	378040	Heparin / Zinc Sulfate Topical Gel	Unfractionated heparin
1133094	RxNorm	106659	Heparin 1 UNT/ML Injectable Solution	Unfractionated heparin
1133095	RxNorm	310713	Heparin 100 UNT/ML Injectable Solution	Unfractionated heparin
1133096	RxNorm	310736	Heparin 3000 UNT/ML Injectable Solution	Unfractionated heparin
1133101	RxNorm	363226	Heparin Injectable Solution [Hep-Pak]	Unfractionated heparin
1133220	RxNorm	417991	HEPARIN, PORK 0.3 UNT/MG Topical Cream	Unfractionated heparin
1133221	RxNorm	418827	HEPARIN, PORK 1.5 UNT/MG Topical Ointment	Unfractionated heparin
1133222	RxNorm	415887	HEPARIN, PORK 1.8 UNT/MG Topical Gel	Unfractionated heparin
1133223	RxNorm	381158	HEPARIN, PORK 2000 UNT/ML Injectable Solution	Unfractionated heparin
1133224	RxNorm	244234	HEPARIN, PORK 25000 UNT/ML Injectable Solution	Unfractionated heparin
1133350	RxNorm	204431	Heparin 2 UNT/ML Injectable Solution	Unfractionated heparin
1133351	RxNorm	310731	Heparin 2.5 UNT/ML Injectable Solution	Unfractionated heparin
1133352	RxNorm	310733	Heparin 2000 UNT/ML Injectable Solution	Unfractionated heparin
1133353	RxNorm	310741	Heparin 50 UNT/ML Injectable Solution	Unfractionated heparin
1133367	RxNorm	310723	Heparin 10 UNT/ML Injectable Solution	Unfractionated heparin
1133368	RxNorm	310732	Heparin 20000 UNT/ML Injectable Solution	Unfractionated heparin
1133369	RxNorm	310738	Heparin 4000 UNT/ML Injectable Solution	Unfractionated heparin
1133393	RxNorm	238722	HEPARIN, PORK 1000 UNT/ML Injectable Solution	Unfractionated heparin
1133394	RxNorm	361838	HEPARIN, PORK 50 UNT/ML Injectable Solution	Unfractionated heparin
1133395	RxNorm	251829	HEPARIN, PORK 5000 UNT/ML Injectable Solution	Unfractionated heparin
1133396	RxNorm	413485	HEPARIN, PORK 75 UNT/ML Injectable Solution	Unfractionated heparin

1133414	RxNorm	310735	Heparin 250 UNT/ML Injectable Solution	Unfractionated heparin
1133415	RxNorm	310737	Heparin 40000 UNT/ML Injectable Solution	Unfractionated heparin
1133436	RxNorm	310734	Heparin 2500 UNT/ML Injectable Solution	Unfractionated heparin
1133439	RxNorm	197758	Heparin 7500 UNT/ML Injectable Solution	Unfractionated heparin
1133440	RxNorm	363227	Heparin Injectable Solution [Hep-Pak CVC]	Unfractionated heparin
1133441	RxNorm	385821	Heparin Injectable Solution [Minihep Calcium]	Unfractionated heparin
1133442	RxNorm	385820	Heparin Injectable Solution [Minihep]	Unfractionated heparin
1133456	RxNorm	310730	Heparin 15000 UNT/ML Injectable Solution	Unfractionated heparin
1133457	RxNorm	387049	Heparin 5 UNT/ML Injectable Solution	Unfractionated heparin
1133463	RxNorm	374387	Heparin Injectable Solution	Unfractionated heparin
1133558	RxNorm	209331	Heparin 10 UNT/ML Injectable Solution [Hep-Lock]	Unfractionated heparin
1133559	RxNorm	209333	Heparin 10 UNT/ML Injectable Solution [Hep-Pak]	Unfractionated heparin
1133560	RxNorm	329805	Heparin 20000 UNT/ML	Unfractionated heparin
1133561	RxNorm	336707	Heparin 5000 UNT	Unfractionated heparin
1133563	RxNorm	329816	Heparin 8.3 UNT/ML	Unfractionated heparin
1133585	RxNorm	334939	HEPARIN, PORK 20000 UNT/ML	Unfractionated heparin
1133586	RxNorm	361837	HEPARIN, PORK 50 UNT/ML	Unfractionated heparin
1133594	RxNorm	209334	Heparin 10 UNT/ML Injectable Solution [Lok-Pak-N]	Unfractionated heparin
1133595	RxNorm	330862	Heparin 2500 UNT/ML	Unfractionated heparin
1133596	RxNorm	329806	Heparin 40000 UNT/ML	Unfractionated heparin
1133601	RxNorm	329815	Heparin 7500 UNT/ML	Unfractionated heparin
1133846	RxNorm	334926	HEPARIN, PORK 5000 UNT/ML	Unfractionated heparin
1133858	RxNorm	329812	Heparin 4000 UNT/ML	Unfractionated heparin
1134377	RxNorm	415881	HEPARIN, PORK 0.15 UNT/MG Topical Gel	Unfractionated heparin
1134378	RxNorm	418983	HEPARIN, PORK 0.3 UNT/MG Topical Ointment	Unfractionated heparin
1134379	RxNorm	418830	HEPARIN, PORK 0.4 UNT/MG Topical Ointment	Unfractionated heparin
1134380	RxNorm	415889	HEPARIN, PORK 0.5 UNT/MG Topical Gel	Unfractionated heparin
1134381	RxNorm	238730	HEPARIN, PORK 20000 UNT/ML Injectable Solution	Unfractionated heparin
1134382	RxNorm	244240	HEPARIN, PORK 40 UNT/ML Injectable Solution	Unfractionated heparin
1134383	RxNorm	282475	HEPARIN, PORK 7500 UNT/ML Injectable Solution	Unfractionated heparin

1134395	RxNorm	440608	HEPARIN, PORK Topical Ointment	Unfractionated heparin
1134420	RxNorm	204429	Heparin 12500 UNT/ML Injectable Solution	Unfractionated heparin
1134421	RxNorm	310739	Heparin 40 UNT/ML Injectable Solution	Unfractionated heparin
1134426	RxNorm	310742	Heparin 6000 UNT/ML Injectable Solution	Unfractionated heparin
1134427	RxNorm	363225	Heparin Injectable Solution [Lok-Pak-N]	Unfractionated heparin
1134428	RxNorm	385823	Heparin Injectable Solution [Uniparin]	Unfractionated heparin
1134429	RxNorm	379429	Heparin Irrigation Solution	Unfractionated heparin
1134601	RxNorm	330699	Heparin 2 UNT/ML	Unfractionated heparin
1134602	RxNorm	329808	Heparin 2.5 UNT/ML	Unfractionated heparin
1134603	RxNorm	393260	Heparin 5 UNT/ML	Unfractionated heparin
1134727	RxNorm	329809	Heparin 2000 UNT/ML	Unfractionated heparin
1134728	RxNorm	329810	Heparin 250 UNT/ML	Unfractionated heparin
1138349	RxNorm	440618	HEPARIN, PORK 0.5 UNT/MG	Unfractionated heparin
1138350	RxNorm	440611	HEPARIN, PORK 1.5 UNT/MG	Unfractionated heparin
1138351	RxNorm	334924	HEPARIN, PORK 20000 UNT	Unfractionated heparin
1138352	RxNorm	334941	HEPARIN, PORK 7500 UNT/ML	Unfractionated heparin
1138364	RxNorm	206631	Heparin 100 UNT/ML Injectable Solution [Hep-Pak]	Unfractionated heparin
1138365	RxNorm	329803	Heparin 1000 UNT/ML	Unfractionated heparin
1138366	RxNorm	330698	Heparin 15000 UNT/ML	Unfractionated heparin
1138370	RxNorm	329814	Heparin 6000 UNT/ML	Unfractionated heparin
1144216	RxNorm	542815	Heparin 10 UNT/ML Injectable Solution [Hepflush]	Unfractionated heparin
1144495	RxNorm	569945	Heparin 10 UNT/ML [Hep-Lock]	Unfractionated heparin
1144496	RxNorm	542818	Heparin 100 UNT/ML [Hep-Lock]	Unfractionated heparin
1144978	RxNorm	569946	Heparin 10 UNT/ML [Hep-Pak CVC]	Unfractionated heparin
1144979	RxNorm	542813	Heparin 10 UNT/ML [Hepflush]	Unfractionated heparin
1144980	RxNorm	569948	Heparin 10 UNT/ML [Lok-Pak-N]	Unfractionated heparin
1144981	RxNorm	567455	Heparin 100 UNT/ML [Hep-Pak]	Unfractionated heparin
1144982	RxNorm	563642	Heparin 1000 UNT/ML [Unihep]	Unfractionated heparin
1145287	RxNorm	542822	Heparin 1000 UNT/ML [Hemochron]	Unfractionated heparin
1145375	RxNorm	545553	HEPARIN, PORK 1250 UNT/ML	Unfractionated heparin
1145672	RxNorm	545554	HEPARIN, PORK 1250 UNT/ML Injectable Solution	Unfractionated heparin
1146292	RxNorm	542823	Heparin Injectable Solution [Hemochron]	Unfractionated heparin
1146334	RxNorm	542814	Heparin Injectable Solution [Hepflush]	Unfractionated heparin
1148129	RxNorm	569947	Heparin 10 UNT/ML [Hep-Pak]	Unfractionated heparin
1148786	RxNorm	606499	Glucose 50 MG/ML / Heparin 50 UNT/ML Injectable Solution	Unfractionated heparin

1150113	RxNorm	729969	3 ML Heparin 100 UNT/ML Prefilled Syringe	Unfractionated heparin
1150177	RxNorm	729970	5 ML Heparin 100 UNT/ML Prefilled Syringe	Unfractionated heparin
1150204	RxNorm	727851	0.5 ML Heparin 25000 UNT/ML Prefilled Syringe	Unfractionated heparin
1150226	RxNorm	729974	1 ML Heparin 10 UNT/ML Prefilled Syringe	Unfractionated heparin
1150227	RxNorm	729973	3 ML Heparin 10 UNT/ML Prefilled Syringe	Unfractionated heparin
1150730	RxNorm	542819	Heparin 100 UNT/ML Injectable Solution [Hep-Lock]	Unfractionated heparin
1150731	RxNorm	542824	Heparin 1000 UNT/ML Injectable Solution [Hemochron]	Unfractionated heparin
1150867	RxNorm	730002	0.5 ML HEPARIN, PORK 10000 UNT/ML Prefilled Syringe	Unfractionated heparin
1150873	RxNorm	746573	3 ML Heparin 1 UNT/ML Prefilled Syringe	Unfractionated heparin
1150890	RxNorm	746632	Heparin 75 UNT/ML Injectable Solution	Unfractionated heparin
1150891	RxNorm	746622	Heparin Topical Ointment	Unfractionated heparin
1151375	RxNorm	581236	Glucose 50 MG/ML / Heparin 100 UNT/ML Injectable Solution	Unfractionated heparin
1151710	RxNorm	581235	Glucose / Heparin Injectable Solution	Unfractionated heparin
1151860	RxNorm	729972	5 ML Heparin 10 UNT/ML Prefilled Syringe	Unfractionated heparin
1151861	RxNorm	729975	2.5 ML Heparin 2.5 UNT/ML Prefilled Syringe	Unfractionated heparin
1151977	RxNorm	746617	Heparin 500 UNT/ML Injectable Solution	Unfractionated heparin
1152102	RxNorm	729976	0.5 ML Heparin 10000 UNT/ML Prefilled Syringe	Unfractionated heparin
1152103	RxNorm	729971	2.5 ML Heparin 100 UNT/ML Prefilled Syringe	Unfractionated heparin
1152104	RxNorm	730001	HEPARIN, PORK Prefilled Syringe	Unfractionated heparin
1153150	RxNorm	746631	Heparin 75 UNT/ML	Unfractionated heparin
1153285	RxNorm	727777	Heparin Prefilled Syringe	Unfractionated heparin
1153289	RxNorm	729968	1 ML Heparin 100 UNT/ML Prefilled Syringe	Unfractionated heparin
1153301	RxNorm	746574	3 ML Heparin 2 UNT/ML Prefilled Syringe	Unfractionated heparin
1153402	RxNorm	791153	Heparin 10 UNT/ML Injectable Solution [Lok-Pak]	Unfractionated heparin
1153426	RxNorm	746616	Heparin 500 UNT/ML	Unfractionated heparin
1153437	RxNorm	794048	10 ML Heparin 10 UNT/ML Prefilled Syringe	Unfractionated heparin
1154533	RxNorm	791155	Heparin 100 UNT/ML Injectable Solution [Lok-Pak]	Unfractionated heparin
1155037	RxNorm	746629	Heparin 66 UNT/ML	Unfractionated heparin

1155078	RxNorm	850445	Heparin Injectable Solution [PosiFlush]	Unfractionated heparin
1155289	RxNorm	791151	Heparin 10 UNT/ML [Lok-Pak]	Unfractionated heparin
1155405	RxNorm	727778	0.2 ML Heparin 25000 UNT/ML Prefilled Syringe	Unfractionated heparin
1155452	RxNorm	850448	Heparin sodium 100 UNT/ML Injectable Solution [PosiFlush]	Unfractionated heparin
1155537	RxNorm	791154	Heparin 100 UNT/ML [Lok-Pak]	Unfractionated heparin
1155554	RxNorm	850444	Heparin sodium 10 UNT/ML [PosiFlush]	Unfractionated heparin
1155571	RxNorm	850447	Heparin sodium 100 UNT/ML [PosiFlush]	Unfractionated heparin
1155600	RxNorm	746630	Heparin 66 UNT/ML Injectable Solution	Unfractionated heparin
1155859	RxNorm	763041	1 ML Heparin 1000 UNT/ML Prefilled Syringe	Unfractionated heparin
1156321	RxNorm	849773	Heparin Inhalant Solution	Unfractionated heparin
1156392	RxNorm	794047	2.5 ML Heparin 10 UNT/ML Prefilled Syringe	Unfractionated heparin
1156393	RxNorm	791152	Heparin Injectable Solution [Lok-Pak]	Unfractionated heparin
1156711	RxNorm	850446	Heparin sodium 10 UNT/ML Injectable Solution [PosiFlush]	Unfractionated heparin
1157680	RxNorm	875912	Heparin sodium 10000 UNT/ML [Unihep]	Unfractionated heparin
1158071	RxNorm	875873	Heparin sodium 25000 UNT/ML [Unihep]	Unfractionated heparin
1158097	RxNorm	875939	Heparin sodium 25000 UNT/ML [Uniparin]	Unfractionated heparin
1158140	RxNorm	876030	Heparin sodium 10000 UNT/ML [Minihep]	Unfractionated heparin
1159505	RxNorm	850443	PosiFlush	Unfractionated heparin
1159999	RxNorm	875936	Heparin sodium 10000 UNT/ML [Minihep Calcium]	Unfractionated heparin
1161142	RxNorm	1014208	Heparin sodium 6 UNT/ML Injectable Solution	Unfractionated heparin
1161929	RxNorm	1014212	Heparin sodium 30 UNT/ML Injectable Solution	Unfractionated heparin
1161956	RxNorm	1043523	Heparin sodium 4 UNT/ML Injectable Solution	Unfractionated heparin
1161993	RxNorm	1014211	Heparin sodium 30 UNT/ML	Unfractionated heparin
1162399	RxNorm	1043522	Heparin sodium 4 UNT/ML	Unfractionated heparin
1163420	RxNorm	1014207	Heparin sodium 6 UNT/ML	Unfractionated heparin
1164639	RxNorm	1119528	Heparin sodium 0.5 UNT/ML Injectable Solution	Unfractionated heparin
1165224	RxNorm	1119527	Heparin sodium 0.5 UNT/ML	Unfractionated heparin
1165339	RxNorm	1190807	2.5 ML Heparin sodium 1 UNT/ML Prefilled Syringe	Unfractionated heparin
1192030	RxNorm	1179295	Minihep Injectable Product	Unfractionated heparin
1192348	RxNorm	1167740	Hemochron Injectable Product	Unfractionated heparin

1192742	RxNorm	1186237	Uniparin Injectable Product	Unfractionated heparin
901530	RxNorm	1168384	Hep-Pak Injectable Product	Unfractionated heparin
1023552	RxNorm	5224	heparin	Unfractionated heparin
1047529	RxNorm	849698	heparin calcium 25000 UNT/ML	Unfractionated heparin
1047530	RxNorm	849699	0.2 ML heparin calcium 25000 UNT/ML Prefilled Syringe	Unfractionated heparin
1047544	RxNorm	849760	heparin calcium 25000 UNT/ML Injectable Solution	Unfractionated heparin
1073198	RxNorm	102366	Heparin 10 UNT/ML Irrigation Solution	Unfractionated heparin
1074975	RxNorm	104459	Heparin 5000 UNT/ML Injectable Solution [Unihep]	Unfractionated heparin
1075813	RxNorm	104460	Heparin 10000 UNT/ML Injectable Solution [Unihep]	Unfractionated heparin
1077399	RxNorm	104461	Heparin 25000 UNT/ML Injectable Solution [Unihep]	Unfractionated heparin
1077967	RxNorm	849657	Heparin sodium 100 UNT/ML [Hep-Pak]	Unfractionated heparin
1080031	RxNorm	849644	Heparin sodium 10 UNT/ML [Hep-Pak]	Unfractionated heparin
1082821	RxNorm	849655	Heparin sodium 10 UNT/ML Injectable Solution [Lok-Pak]	Unfractionated heparin
1082822	RxNorm	849713	Heparin sodium 15000 UNT/ML	Unfractionated heparin
1082954	RxNorm	581237	Glucose 50 MG/ML / Heparin 40 UNT/ML Injectable Solution	Unfractionated heparin
1082955	RxNorm	849333	2 ML Heparin sodium 10 UNT/ML Prefilled Syringe	Unfractionated heparin
1082990	RxNorm	848339	Heparin sodium 10 UNT/ML Injectable Solution	Unfractionated heparin
1082991	RxNorm	849317	1 ML Heparin sodium 10 UNT/ML Prefilled Syringe	Unfractionated heparin
1082992	RxNorm	251672	Heparin 0.01 MG/MG Topical Gel	Unfractionated heparin
1082993	RxNorm	829885	0.75 ML Heparin sodium 10000 UNT/ML Prefilled Syringe	Unfractionated heparin
1082994	RxNorm	829886	0.5 ML Heparin sodium 10000 UNT/ML Prefilled Syringe	Unfractionated heparin
1082995	RxNorm	849312	3 ML Heparin sodium 10 UNT/ML Prefilled Syringe	Unfractionated heparin
1082996	RxNorm	849313	5 ML Heparin sodium 10 UNT/ML Prefilled Syringe	Unfractionated heparin
1082997	RxNorm	849341	5 ML Heparin sodium 100 UNT/ML Prefilled Syringe	Unfractionated heparin
1082998	RxNorm	849715	Heparin sodium 2 UNT/ML Injectable Solution	Unfractionated heparin
1082999	RxNorm	849722	Heparin sodium 20000 UNT/ML Injectable Solution	Unfractionated heparin
1083000	RxNorm	849764	Heparin sodium 40 UNT/ML Injectable Solution	Unfractionated heparin

1083008	RxNorm	849344	3 ML Heparin sodium 2 UNT/ML Prefilled Syringe	Unfractionated heparin
1083092	RxNorm	197757	Heparin 25000 UNT/ML Injectable Solution	Unfractionated heparin
1083093	RxNorm	849334	2.5 ML Heparin sodium 10 UNT/ML Prefilled Syringe	Unfractionated heparin
1083094	RxNorm	849338	2 ML Heparin sodium 100 UNT/ML Prefilled Syringe	Unfractionated heparin
1083095	RxNorm	849779	Heparin sodium 6000 UNT/ML Injectable Solution	Unfractionated heparin
1083512	RxNorm	848334	Heparin sodium 100 UNT/ML	Unfractionated heparin
1083513	RxNorm	849296	Heparin sodium 1 UNT/ML	Unfractionated heparin
1083514	RxNorm	849721	Heparin sodium 20000 UNT/ML	Unfractionated heparin
1083639	RxNorm	334942	Heparin 0.16 UNT/MG	Unfractionated heparin
1083640	RxNorm	848338	Heparin sodium 10 UNT/ML	Unfractionated heparin
1083641	RxNorm	849343	Heparin sodium 2 UNT/ML	Unfractionated heparin
1083642	RxNorm	849717	Heparin sodium 2000 UNT/ML	Unfractionated heparin
1083673	RxNorm	829888	0.25 ML Heparin sodium 10000 UNT/ML Prefilled Syringe	Unfractionated heparin
1083674	RxNorm	848335	Heparin sodium 100 UNT/ML Injectable Solution	Unfractionated heparin
1083675	RxNorm	849335	10 ML Heparin sodium 10 UNT/ML Prefilled Syringe	Unfractionated heparin
1083676	RxNorm	849339	2.5 ML Heparin sodium 100 UNT/ML Prefilled Syringe	Unfractionated heparin
1083677	RxNorm	849714	Heparin sodium 15000 UNT/ML Injectable Solution	Unfractionated heparin
1083678	RxNorm	849776	Heparin sodium 5000 UNT/ML Injectable Solution	Unfractionated heparin
1083895	RxNorm	334927	Heparin 0.01 MG/MG	Unfractionated heparin
1083896	RxNorm	848337	Heparin sodium 100 UNT/ML Injectable Solution [Hep-Lock]	Unfractionated heparin
1083897	RxNorm	849658	Heparin sodium 100 UNT/ML Injectable Solution [Hep-Pak]	Unfractionated heparin
1083942	RxNorm	848341	Heparin sodium 10 UNT/ML Injectable Solution [Hep-Lock]	Unfractionated heparin
1083943	RxNorm	849645	Heparin sodium 10 UNT/ML Injectable Solution [Hep-Pak]	Unfractionated heparin
1083944	RxNorm	849725	Heparin sodium 2500 UNT/ML	Unfractionated heparin
1083986	RxNorm	849769	Heparin sodium 50 UNT/ML	Unfractionated heparin
1083987	RxNorm	849775	Heparin sodium 5000 UNT/ML	Unfractionated heparin
1084072	RxNorm	829883	Heparin sodium 10000 UNT/ML	Unfractionated heparin
1084487	RxNorm	830698	Heparin sodium 10000 UNT/ML Injectable Solution	Unfractionated heparin
1084525	RxNorm	849768	Heparin sodium 40000 UNT/ML Injectable Solution	Unfractionated heparin
1084526	RxNorm	849785	Heparin sodium 8.3 UNT/ML Injectable Solution	Unfractionated heparin

1084539	RxNorm	829884	1 ML Heparin sodium 10000 UNT/ML Prefilled Syringe	Unfractionated heparin
1084540	RxNorm	849718	Heparin sodium 2000 UNT/ML Injectable Solution	Unfractionated heparin
1084541	RxNorm	849762	Heparin sodium 3000 UNT/ML Injectable Solution	Unfractionated heparin
1084542	RxNorm	849766	Heparin sodium 4000 UNT/ML Injectable Solution	Unfractionated heparin
1084792	RxNorm	849765	Heparin sodium 4000 UNT/ML	Unfractionated heparin
1084793	RxNorm	849782	Heparin sodium 7500 UNT/ML	Unfractionated heparin
1084818	RxNorm	849763	Heparin sodium 40 UNT/ML	Unfractionated heparin
1084819	RxNorm	849767	Heparin sodium 40000 UNT/ML	Unfractionated heparin
1084820	RxNorm	849784	Heparin sodium 8.3 UNT/ML	Unfractionated heparin
1085024	RxNorm	849783	Heparin sodium 7500 UNT/ML Injectable Solution	Unfractionated heparin
1085357	RxNorm	849761	Heparin sodium 3000 UNT/ML	Unfractionated heparin
1085358	RxNorm	849778	Heparin sodium 6000 UNT/ML	Unfractionated heparin
1085613	RxNorm	849299	3 ML Heparin sodium 1 UNT/ML Prefilled Syringe	Unfractionated heparin
1085614	RxNorm	849337	1 ML Heparin sodium 100 UNT/ML Prefilled Syringe	Unfractionated heparin
1085615	RxNorm	849726	Heparin sodium 2500 UNT/ML Injectable Solution	Unfractionated heparin
1088221	RxNorm	150642	Heparin 10000 UNT/ML Injectable Solution [Minihep Calcium]	Unfractionated heparin
1089451	RxNorm	849660	Heparin sodium 100 UNT/ML Injectable Solution [Lok-Pak]	Unfractionated heparin
1089604	RxNorm	849700	Heparin sodium 25000 UNT/ML	Unfractionated heparin
1089605	RxNorm	849723	Heparin sodium 250 UNT/ML	Unfractionated heparin
1089607	RxNorm	849809	heparin calcium 500 UNT/ML	Unfractionated heparin
1089836	RxNorm	849297	1 ML Heparin sodium 1 UNT/ML Prefilled Syringe	Unfractionated heparin
1089837	RxNorm	849300	5 ML Heparin sodium 1 UNT/ML Prefilled Syringe	Unfractionated heparin
1089857	RxNorm	849340	3 ML Heparin sodium 100 UNT/ML Prefilled Syringe	Unfractionated heparin
1089859	RxNorm	849720	heparin calcium 20000 UNT/ML Injectable Solution	Unfractionated heparin
1089915	RxNorm	849759	Heparin sodium 25000 UNT/ML Injectable Solution	Unfractionated heparin
1090000	RxNorm	849780	Heparin sodium 66 UNT/ML	Unfractionated heparin
1090001	RxNorm	849811	Heparin sodium 75 UNT/ML	Unfractionated heparin
1090039	RxNorm	849301	10 ML Heparin sodium 1 UNT/ML Prefilled Syringe	Unfractionated heparin
1090091	RxNorm	150643	Heparin 25000 UNT/ML Injectable Solution [Uniparin]	Unfractionated heparin
1090231	RxNorm	849653	Heparin sodium 10 UNT/ML Injectable Solution [Hepflush]	Unfractionated heparin
1090234	RxNorm	849703	Heparin sodium 1000 UNT/ML	Unfractionated heparin

1090235	RxNorm	849705	Heparin sodium 2.5 UNT/ML	Unfractionated heparin
1090236	RxNorm	849711	Heparin sodium 12500 UNT/ML	Unfractionated heparin
1090273	RxNorm	849298	2 ML Heparin sodium 1 UNT/ML Prefilled Syringe	Unfractionated heparin
1090345	RxNorm	150641	Heparin 10000 UNT/ML Injectable Solution [Minihep]	Unfractionated heparin
1090389	RxNorm	849709	Heparin sodium 1 UNT/ML Injectable Solution	Unfractionated heparin
1090390	RxNorm	849808	heparin calcium 10000 UNT/ML Injectable Solution	Unfractionated heparin
1090401	RxNorm	849724	Heparin sodium 250 UNT/ML Injectable Solution	Unfractionated heparin
1090402	RxNorm	849774	Heparin sodium 50 UNT/ML Inhalant Solution	Unfractionated heparin
1090403	RxNorm	849810	heparin calcium 500 UNT/ML Injectable Solution	Unfractionated heparin
1090463	RxNorm	848340	Heparin sodium 10 UNT/ML [Hep-Lock]	Unfractionated heparin
1090527	RxNorm	849813	Heparin sodium 1000 UNT/ML [Hemochron]	Unfractionated heparin
1090673	RxNorm	849342	10 ML Heparin sodium 100 UNT/ML Prefilled Syringe	Unfractionated heparin
1090926	RxNorm	848336	Heparin sodium 100 UNT/ML [Hep-Lock]	Unfractionated heparin
1091245	RxNorm	203450	Hep-Lock	Unfractionated heparin
1091385	RxNorm	849652	Heparin sodium 10 UNT/ML [Hepflush]	Unfractionated heparin
1091386	RxNorm	849702	0.5 ML heparin calcium 25000 UNT/ML Prefilled Syringe	Unfractionated heparin
1091669	RxNorm	849716	Heparin sodium 2.5 UNT/ML Injectable Solution	Unfractionated heparin
1091670	RxNorm	849781	Heparin sodium 66 UNT/ML Injectable Solution	Unfractionated heparin
1091671	RxNorm	849812	Heparin sodium 75 UNT/ML Injectable Solution	Unfractionated heparin
1091823	RxNorm	849807	heparin calcium 10000 UNT/ML	Unfractionated heparin
1092284	RxNorm	849704	1 ML Heparin sodium 1000 UNT/ML Prefilled Syringe	Unfractionated heparin
1092285	RxNorm	849706	2.5 ML Heparin sodium 2.5 UNT/ML Prefilled Syringe	Unfractionated heparin
1092286	RxNorm	849710	Heparin sodium 1000 UNT/ML Injectable Solution	Unfractionated heparin
1092287	RxNorm	849712	Heparin sodium 12500 UNT/ML Injectable Solution	Unfractionated heparin
1092288	RxNorm	849770	Heparin sodium 50 UNT/ML Injectable Solution	Unfractionated heparin
1092289	RxNorm	849772	Heparin sodium 5 UNT/ML Injectable Solution	Unfractionated heparin
1092382	RxNorm	849815	Heparin sodium 1000 UNT/ML [Unihep]	Unfractionated heparin

1093300	RxNorm	849701	0.2 ML Heparin sodium 25000 UNT/ML Prefilled Syringe	Unfractionated heparin
1093676	RxNorm	849719	heparin calcium 20000 UNT/ML	Unfractionated heparin
1093701	RxNorm	849771	Heparin sodium 5 UNT/ML	Unfractionated heparin
1093702	RxNorm	849814	Heparin sodium 1000 UNT/ML Injectable Solution [Hemochron]	Unfractionated heparin
1093703	RxNorm	849816	Heparin sodium 1000 UNT/ML Injectable Solution [Unihep]	Unfractionated heparin
1095422	RxNorm	310740	Heparin 5000 UNT/ML Injectable Solution	Unfractionated heparin
1095751	RxNorm	1857418	heparin Injectable Solution [Hep-Lock]	Unfractionated heparin
1096698	RxNorm	1857609	heparin Prefilled Syringe [PosiFlush]	Unfractionated heparin
1097185	RxNorm	1857599	heparin Injection [Hepflush]	Unfractionated heparin
1097864	RxNorm	1857596	heparin Injectable Solution [Lok-Pak]	Unfractionated heparin
1098434	RxNorm	314013	Heparin 10000 UNT/ML Injectable Solution	Unfractionated heparin
1100606	RxNorm	746621	Heparin 1 UNT/MG Topical Gel	Unfractionated heparin
1100607	RxNorm	746708	Heparin 0.4 UNT/MG Topical Ointment	Unfractionated heparin
1100608	RxNorm	746711	Heparin 1.5 UNT/MG Topical Ointment	Unfractionated heparin
1100609	RxNorm	746712	Heparin 0.5 UNT/MG Topical Gel	Unfractionated heparin
1100895	RxNorm	328869	Heparin 10 UNT/ML	Unfractionated heparin
1101261	RxNorm	746618	Heparin 1250 UNT/ML	Unfractionated heparin
1101263	RxNorm	746693	Heparin 0.05 UNT/MG	Unfractionated heparin
1101438	RxNorm	746703	Heparin 0.15 UNT/MG	Unfractionated heparin
1101486	RxNorm	746694	Heparin 0.05 UNT/MG Topical Ointment	Unfractionated heparin
1101487	RxNorm	746696	Heparin 0.6 UNT/MG Topical Gel	Unfractionated heparin
1101752	RxNorm	746704	Heparin 0.15 UNT/MG Topical Gel	Unfractionated heparin
1101906	RxNorm	746624	Heparin 1.3 UNT/MG	Unfractionated heparin
1101907	RxNorm	746695	Heparin 0.6 UNT/MG	Unfractionated heparin
1102382	RxNorm	746619	Heparin 1250 UNT/ML Injectable Solution	Unfractionated heparin
1102383	RxNorm	746623	Heparin 1 UNT/MG Topical Ointment	Unfractionated heparin
1102384	RxNorm	746692	Heparin 0.5 UNT/MG Topical Ointment	Unfractionated heparin
1102488	RxNorm	746706	Heparin 1.8 UNT/MG Topical Ointment	Unfractionated heparin
1102489	RxNorm	746709	Heparin 0.3 UNT/MG Topical Ointment	Unfractionated heparin
1102490	RxNorm	746713	Heparin 1.5 UNT/MG Topical Gel	Unfractionated heparin
1102573	RxNorm	746620	Heparin 1 UNT/MG	Unfractionated heparin
1102575	RxNorm	746691	Heparin 0.5 UNT/MG	Unfractionated heparin
1102576	RxNorm	746710	Heparin 1.5 UNT/MG	Unfractionated heparin
1102655	RxNorm	746702	Heparin 1.8 UNT/MG Topical Gel	Unfractionated heparin

1102656	RxNorm	746707	Heparin 0.6 UNT/MG Topical Ointment	Unfractionated heparin
1102666	RxNorm	746698	Heparin 0.3 UNT/MG Topical Gel	Unfractionated heparin
1102667	RxNorm	746700	Heparin 0.4 UNT/MG Topical Gel	Unfractionated heparin
1102745	RxNorm	746699	Heparin 0.4 UNT/MG	Unfractionated heparin
1102746	RxNorm	746701	Heparin 1.8 UNT/MG	Unfractionated heparin
1102910	RxNorm	746625	Heparin 1.3 UNT/MG Topical Ointment	Unfractionated heparin
1102926	RxNorm	746697	Heparin 0.3 UNT/MG	Unfractionated heparin
1103049	RxNorm	329801	Heparin 25000 UNT/ML	Unfractionated heparin
1103050	RxNorm	329807	Heparin 5000 UNT/ML	Unfractionated heparin
1103140	RxNorm	329802	Heparin 10000 UNT/ML	Unfractionated heparin
1194399	RxNorm	1008073	Heparin / Zinc Sulfate	Unfractionated heparin
1194444	RxNorm	1186234	Unihep Injectable Product	Unfractionated heparin
1196578	RxNorm	1179294	Minihep Calcium Injectable Product	Unfractionated heparin
1196650	RxNorm	1008712	Glucose / Heparin	Unfractionated heparin
917885	RxNorm	1359836	heparin calcium 25000 UNT/ML Prefilled Syringe	Unfractionated heparin
920276	RxNorm	1361026	heparin sodium, porcine 10 UNT/ML	Unfractionated heparin
920277	RxNorm	1361029	heparin sodium, porcine 10 UNT/ML Injectable Solution	Unfractionated heparin
920278	RxNorm	1361033	heparin sodium, porcine 10 UNT/ML [Hep-Pak]	Unfractionated heparin
920279	RxNorm	1361035	heparin sodium, porcine 10 UNT/ML Injectable Solution [Hep-Pak]	Unfractionated heparin
920282	RxNorm	1361047	heparin sodium, porcine 100 UNT/ML	Unfractionated heparin
920283	RxNorm	1361048	heparin sodium, porcine 100 UNT/ML Injectable Solution	Unfractionated heparin
920284	RxNorm	1361197	heparin sodium, porcine 100 UNT/ML [Hep-Pak]	Unfractionated heparin
920285	RxNorm	1361198	heparin sodium, porcine 100 UNT/ML Injectable Solution [Hep-Pak]	Unfractionated heparin
920558	RxNorm	1361225	heparin sodium, porcine 1000 UNT/ML	Unfractionated heparin
920559	RxNorm	1361226	heparin sodium, porcine 1000 UNT/ML Injectable Solution	Unfractionated heparin
920711	RxNorm	1361561	heparin sodium, porcine 2.5 UNT/ML	Unfractionated heparin
920712	RxNorm	1361562	heparin sodium, porcine 2.5 UNT/ML Injectable Solution	Unfractionated heparin
920714	RxNorm	1361567	heparin sodium, porcine 2000 UNT/ML	Unfractionated heparin
920715	RxNorm	1361568	5 ML heparin sodium, porcine 2000 UNT/ML Injection	Unfractionated heparin
920718	RxNorm	1361573	heparin sodium, porcine 20000 UNT/ML	Unfractionated heparin

920719	RxNorm	1361574	heparin sodium, porcine 20000 UNT/ML Injectable Solution	Unfractionated heparin
920720	RxNorm	1361576	heparin sodium, porcine 2500 UNT/ML	Unfractionated heparin
920721	RxNorm	1361577	10 ML heparin sodium, porcine 2500 UNT/ML Injection	Unfractionated heparin
920722	RxNorm	1361593	heparin sodium, porcine 25000 UNT/ML	Unfractionated heparin
920723	RxNorm	1361594	heparin sodium, porcine 25000 UNT/ML Injectable Solution	Unfractionated heparin
920724	RxNorm	1361606	heparin sodium, porcine 40 UNT/ML	Unfractionated heparin
920725	RxNorm	1361607	500 ML heparin sodium, porcine 40 UNT/ML Injection	Unfractionated heparin
920726	RxNorm	1361608	heparin sodium, porcine 40000 UNT/ML	Unfractionated heparin
920727	RxNorm	1361609	heparin sodium, porcine 40000 UNT/ML Injectable Solution	Unfractionated heparin
920728	RxNorm	1361612	heparin sodium, porcine 50 UNT/ML	Unfractionated heparin
920729	RxNorm	1361614	heparin sodium, porcine 5000 UNT/ML	Unfractionated heparin
920730	RxNorm	1361615	heparin sodium, porcine 5000 UNT/ML Injectable Solution	Unfractionated heparin
920765	RxNorm	1361757	heparin sodium, bovine 5000 UNT/ML	Unfractionated heparin
920766	RxNorm	1361760	heparin sodium, bovine 5000 UNT/ML Injectable Solution	Unfractionated heparin
920824	RxNorm	1362054	3 ML heparin sodium, porcine 10 UNT/ML Prefilled Syringe	Unfractionated heparin
920825	RxNorm	1362058	heparin sodium, porcine 100 UNT/ML Prefilled Syringe	Unfractionated heparin
920802	RxNorm	1361852	heparin sodium, porcine 10000 UNT/ML	Unfractionated heparin
920803	RxNorm	1361853	0.5 ML heparin sodium, porcine 10000 UNT/ML Cartridge	Unfractionated heparin
920806	RxNorm	1362023	heparin sodium, porcine 1 UNT/ML	Unfractionated heparin
920807	RxNorm	1362025	heparin sodium, porcine 1 UNT/ML Prefilled Syringe	Unfractionated heparin
920808	RxNorm	1362026	10 ML heparin sodium, porcine 1 UNT/ML Prefilled Syringe	Unfractionated heparin
920822	RxNorm	1362049	heparin sodium, porcine 10 UNT/ML Prefilled Syringe	Unfractionated heparin
920981	RxNorm	1362059	10 ML heparin sodium, porcine 100 UNT/ML Prefilled Syringe	Unfractionated heparin
920982	RxNorm	1362060	2 ML heparin sodium, porcine 100 UNT/ML Prefilled Syringe	Unfractionated heparin
920983	RxNorm	1362061	2.5 ML heparin sodium, porcine 100 UNT/ML Prefilled Syringe	Unfractionated heparin

920984	RxNorm	1362062	3 ML heparin sodium, porcine 100 UNT/ML Prefilled Syringe	Unfractionated heparin
920985	RxNorm	1362063	5 ML heparin sodium, porcine 100 UNT/ML Prefilled Syringe	Unfractionated heparin
920986	RxNorm	1362064	heparin sodium, porcine 2 UNT/ML	Unfractionated heparin
920987	RxNorm	1362067	3 ML heparin sodium, porcine 2000 UNT/ML Prefilled Syringe	Unfractionated heparin
920988	RxNorm	1362068	heparin sodium, porcine 2000 UNT/ML Prefilled Syringe	Unfractionated heparin
920989	RxNorm	1362069	1 ML heparin sodium, porcine 1000 UNT/ML Prefilled Syringe	Unfractionated heparin
920990	RxNorm	1362070	heparin sodium, porcine 1000 UNT/ML Prefilled Syringe	Unfractionated heparin
921437	RxNorm	1362821	heparin sodium, porcine 7500 UNT/ML	Unfractionated heparin
921438	RxNorm	1362822	heparin sodium, porcine 7500 UNT/ML Injectable Solution	Unfractionated heparin
921439	RxNorm	1362823	heparin sodium, porcine 8 UNT/ML	Unfractionated heparin
921528	RxNorm	1362824	heparin sodium, porcine 8 UNT/ML Injectable Solution	Unfractionated heparin
921529	RxNorm	1362829	heparin sodium, bovine 1000 UNT/ML	Unfractionated heparin
921530	RxNorm	1362830	heparin sodium, bovine 1000 UNT/ML Injectable Solution	Unfractionated heparin
921531	RxNorm	1362831	heparin sodium, porcine 10000 UNT/ML Injectable Solution	Unfractionated heparin
921532	RxNorm	1362832	heparin sodium, bovine 10000 UNT/ML	Unfractionated heparin
921533	RxNorm	1362833	heparin sodium, bovine 10000 UNT/ML Injectable Solution	Unfractionated heparin
921534	RxNorm	1362836	heparin sodium, porcine 12500 UNT/ML	Unfractionated heparin
921535	RxNorm	1362837	heparin sodium, porcine 12500 UNT/ML Injectable Solution	Unfractionated heparin
921781	RxNorm	1362935	heparin sodium, porcine 2 UNT/ML Injectable Solution	Unfractionated heparin
921880	RxNorm	1362943	heparin sodium, porcine 15000 UNT/ML	Unfractionated heparin
921881	RxNorm	1362944	heparin sodium, porcine 15000 UNT/ML Injectable Solution	Unfractionated heparin
921882	RxNorm	1362945	heparin sodium, porcine 250 UNT/ML	Unfractionated heparin
921883	RxNorm	1362946	heparin sodium, porcine 250 UNT/ML Injectable Solution	Unfractionated heparin
921884	RxNorm	1362947	heparin sodium, porcine 3000 UNT/ML	Unfractionated heparin
921885	RxNorm	1362948	heparin sodium, porcine 3000 UNT/ML Injectable Solution	Unfractionated heparin

921886	RxNorm	1362949	heparin sodium, porcine 4000 UNT/ML	Unfractionated heparin
921887	RxNorm	1362950	heparin sodium, porcine 4000 UNT/ML Injectable Solution	Unfractionated heparin
921888	RxNorm	1362951	heparin sodium, porcine 6000 UNT/ML	Unfractionated heparin
921889	RxNorm	1362952	heparin sodium, porcine 6000 UNT/ML Injectable Solution	Unfractionated heparin
921890	RxNorm	1362953	heparin sodium, porcine 66 UNT/ML	Unfractionated heparin
921891	RxNorm	1362954	heparin sodium, porcine 66 UNT/ML Injectable Solution	Unfractionated heparin
921892	RxNorm	1362957	heparin sodium, porcine 8.3 UNT/ML	Unfractionated heparin
921893	RxNorm	1362958	heparin sodium, porcine 8.3 UNT/ML Injectable Solution	Unfractionated heparin
921894	RxNorm	1362961	heparin sodium, porcine 0.25 UNT/ML	Unfractionated heparin
921895	RxNorm	1362962	heparin sodium, porcine 0.25 UNT/ML Injectable Solution	Unfractionated heparin
929866	RxNorm	1442414	6 ML heparin sodium, porcine 100 UNT/ML Prefilled Syringe	Unfractionated heparin
941123	RxNorm	1656760	10 ML heparin sodium, porcine 10 UNT/ML Injection	Unfractionated heparin
941124	RxNorm	1656763	heparin sodium, porcine 10 UNT/ML Injection	Unfractionated heparin
943151	RxNorm	1658635	heparin sodium, porcine 10000 UNT/ML Injection	Unfractionated heparin
943152	RxNorm	1658637	1 ML heparin sodium, porcine 10000 UNT/ML Injection	Unfractionated heparin
943153	RxNorm	1658647	2 ML heparin sodium, porcine 1000 UNT/ML Injection	Unfractionated heparin
943154	RxNorm	1658648	heparin sodium, porcine 1000 UNT/ML Injection	Unfractionated heparin
943158	RxNorm	1658659	1 ML heparin sodium, porcine 1000 UNT/ML Injection	Unfractionated heparin
943150	RxNorm	1658634	0.5 ML heparin sodium, porcine 10000 UNT/ML Injection	Unfractionated heparin
943357	RxNorm	1658690	1000 ML heparin sodium, porcine 2 UNT/ML Injection	Unfractionated heparin
943358	RxNorm	1658691	heparin sodium, porcine 2 UNT/ML Injection	Unfractionated heparin
943359	RxNorm	1658692	500 ML heparin sodium, porcine 2 UNT/ML Injection	Unfractionated heparin
943454	RxNorm	1658707	1 ML heparin sodium, porcine 10 UNT/ML Injection	Unfractionated heparin
943464	RxNorm	1658717	250 ML heparin sodium, porcine 100 UNT/ML Injection	Unfractionated heparin
943465	RxNorm	1658718	heparin sodium, porcine 100 UNT/ML Injection	Unfractionated heparin

943466	RxNorm	1658719	1 ML heparin sodium, porcine 100 UNT/ML Injection	Unfractionated heparin
943467	RxNorm	1658720	100 ML heparin sodium, porcine 100 UNT/ML Injection	Unfractionated heparin
943537	RxNorm	1657985	heparin sodium, porcine 10000 UNT/ML Cartridge	Unfractionated heparin
943538	RxNorm	1657987	heparin sodium, porcine 2500 UNT/ML Injection	Unfractionated heparin
943539	RxNorm	1657989	heparin sodium, porcine 2000 UNT/ML Injection	Unfractionated heparin
943544	RxNorm	1657997	heparin sodium, porcine 40 UNT/ML Injection	Unfractionated heparin
944335	RxNorm	1659195	500 ML heparin sodium, porcine 50 UNT/ML Injection	Unfractionated heparin
944336	RxNorm	1659196	heparin sodium, porcine 50 UNT/ML Injection	Unfractionated heparin
944337	RxNorm	1659197	250 ML heparin sodium, porcine 50 UNT/ML Injection	Unfractionated heparin
944345	RxNorm	1659260	1 ML heparin sodium, porcine 5000 UNT/ML Cartridge	Unfractionated heparin
944495	RxNorm	1659261	heparin sodium, porcine 5000 UNT/ML Cartridge	Unfractionated heparin
944496	RxNorm	1659263	1 ML heparin sodium, porcine 5000 UNT/ML Injection	Unfractionated heparin
944497	RxNorm	1659264	heparin sodium, porcine 5000 UNT/ML Injection	Unfractionated heparin
948849	RxNorm	1856274	heparin Injectable Product	Unfractionated heparin
948850	RxNorm	1856275	heparin Injectable Solution	Unfractionated heparin
948851	RxNorm	1856278	heparin Prefilled Syringe	Unfractionated heparin
949230	RxNorm	1857594	heparin Injectable Solution [Hep-Pak]	Unfractionated heparin
949231	RxNorm	1857598	heparin Injection	Unfractionated heparin
949475	RxNorm	1857949	heparin Cartridge	Unfractionated heparin
949496	RxNorm	1798389	1 ML heparin sodium, porcine 5000 UNT/ML Prefilled Syringe	Unfractionated heparin
949497	RxNorm	1798390	heparin sodium, porcine 5000 UNT/ML Prefilled Syringe	Unfractionated heparin
966203	RxNorm	2121591	0.5 ML heparin sodium, porcine 10000 UNT/ML Prefilled Syringe	Unfractionated heparin
966204	RxNorm	2121592	heparin sodium, porcine 10000 UNT/ML Prefilled Syringe	Unfractionated heparin
968167	RxNorm	217517	Hep-Pak	Unfractionated heparin
880495	RxNorm	11289	warfarin	Vitamin K Antagonist
888406	RxNorm	1161789	warfarin Injectable Product	Vitamin K Antagonist
888407	RxNorm	1161790	warfarin Oral Product	Vitamin K Antagonist
888408	RxNorm	1161791	warfarin Pill	Vitamin K Antagonist
895086	RxNorm	1171654	Coumadin Injectable Product	Vitamin K Antagonist
895087	RxNorm	1171655	Coumadin Oral Product	Vitamin K Antagonist
895088	RxNorm	1171656	Coumadin Pill	Vitamin K Antagonist

897801	RxNorm	1181005	Marfarin Oral Product	Vitamin K Antagonist
897802	RxNorm	1181006	Marfarin Pill	Vitamin K Antagonist
900223	RxNorm	1167808	Jantoven Oral Product	Vitamin K Antagonist
900224	RxNorm	1167809	Jantoven Pill	Vitamin K Antagonist
903787	RxNorm	1179316	Narfarin Oral Product	Vitamin K Antagonist
903788	RxNorm	1179317	Narfarin Pill	Vitamin K Antagonist
1010232	RxNorm	405155	Jantoven	Vitamin K Antagonist
1011873	RxNorm	406078	warfarin Oral Tablet [Jantoven]	Vitamin K Antagonist
1025433	RxNorm	540205	Narfarin	Vitamin K Antagonist
1025434	RxNorm	540207	warfarin Oral Tablet [Narfarin]	Vitamin K Antagonist
1025435	RxNorm	540226	Marfarin	Vitamin K Antagonist
1025436	RxNorm	540228	warfarin Oral Tablet [Marfarin]	Vitamin K Antagonist
1049738	RxNorm	855287	warfarin sodium 1 MG	Vitamin K Antagonist
1050054	RxNorm	855288	warfarin sodium 1 MG Oral Tablet	Vitamin K Antagonist
1050055	RxNorm	855289	warfarin sodium 1 MG [Coumadin]	Vitamin K Antagonist
1050056	RxNorm	855290	warfarin sodium 1 MG Oral Tablet [Coumadin]	Vitamin K Antagonist
1050057	RxNorm	855291	warfarin sodium 1 MG [Jantoven]	Vitamin K Antagonist
1050058	RxNorm	855292	warfarin sodium 1 MG Oral Tablet [Jantoven]	Vitamin K Antagonist
1050059	RxNorm	855293	warfarin sodium 1 MG [Narfarin]	Vitamin K Antagonist
1050060	RxNorm	855294	warfarin sodium 1 MG Oral Tablet [Narfarin]	Vitamin K Antagonist
1050061	RxNorm	855295	warfarin sodium 10 MG	Vitamin K Antagonist
1050062	RxNorm	855296	warfarin sodium 10 MG Oral Tablet	Vitamin K Antagonist
1050063	RxNorm	855297	warfarin sodium 10 MG [Coumadin]	Vitamin K Antagonist
1050064	RxNorm	855298	warfarin sodium 10 MG Oral Tablet [Coumadin]	Vitamin K Antagonist
1050065	RxNorm	855299	warfarin sodium 10 MG [Jantoven]	Vitamin K Antagonist
1050066	RxNorm	855300	warfarin sodium 10 MG Oral Tablet [Jantoven]	Vitamin K Antagonist
1050067	RxNorm	855301	warfarin sodium 2 MG	Vitamin K Antagonist
1050068	RxNorm	855302	warfarin sodium 2 MG Oral Tablet	Vitamin K Antagonist
1050069	RxNorm	855303	warfarin sodium 2 MG [Coumadin]	Vitamin K Antagonist
1050070	RxNorm	855304	warfarin sodium 2 MG Oral Tablet [Coumadin]	Vitamin K Antagonist
1050071	RxNorm	855305	warfarin sodium 2 MG [Jantoven]	Vitamin K Antagonist
1050072	RxNorm	855306	warfarin sodium 2 MG Oral Tablet [Jantoven]	Vitamin K Antagonist
1050073	RxNorm	855307	warfarin sodium 2 MG/ML	Vitamin K Antagonist

1050074	RxNorm	855308	warfarin sodium 2 MG/ML Injectable Solution	Vitamin K Antagonist
1050075	RxNorm	855309	warfarin sodium 2 MG/ML [Coumadin]	Vitamin K Antagonist
1050076	RxNorm	855310	warfarin sodium 2 MG/ML Injectable Solution [Coumadin]	Vitamin K Antagonist
1050077	RxNorm	855311	warfarin sodium 2.5 MG	Vitamin K Antagonist
1050078	RxNorm	855312	warfarin sodium 2.5 MG Oral Tablet	Vitamin K Antagonist
1050079	RxNorm	855313	warfarin sodium 2.5 MG [Coumadin]	Vitamin K Antagonist
1050080	RxNorm	855314	warfarin sodium 2.5 MG Oral Tablet [Coumadin]	Vitamin K Antagonist
1050081	RxNorm	855315	warfarin sodium 2.5 MG [Jantoven]	Vitamin K Antagonist
1050082	RxNorm	855316	warfarin sodium 2.5 MG Oral Tablet [Jantoven]	Vitamin K Antagonist
1050083	RxNorm	855317	warfarin sodium 3 MG	Vitamin K Antagonist
1050084	RxNorm	855318	warfarin sodium 3 MG Oral Tablet	Vitamin K Antagonist
1050248	RxNorm	855319	warfarin sodium 3 MG [Coumadin]	Vitamin K Antagonist
1050249	RxNorm	855320	warfarin sodium 3 MG Oral Tablet [Coumadin]	Vitamin K Antagonist
1050250	RxNorm	855321	warfarin sodium 3 MG [Jantoven]	Vitamin K Antagonist
1050251	RxNorm	855322	warfarin sodium 3 MG Oral Tablet [Jantoven]	Vitamin K Antagonist
1050252	RxNorm	855323	warfarin sodium 4 MG	Vitamin K Antagonist
1050253	RxNorm	855324	warfarin sodium 4 MG Oral Tablet	Vitamin K Antagonist
1050254	RxNorm	855325	warfarin sodium 4 MG [Coumadin]	Vitamin K Antagonist
1050255	RxNorm	855326	warfarin sodium 4 MG Oral Tablet [Coumadin]	Vitamin K Antagonist
1050256	RxNorm	855327	warfarin sodium 4 MG [Jantoven]	Vitamin K Antagonist
1050257	RxNorm	855328	warfarin sodium 4 MG Oral Tablet [Jantoven]	Vitamin K Antagonist
1050258	RxNorm	855329	warfarin sodium 4 MG [Marfarin]	Vitamin K Antagonist
1050259	RxNorm	855330	warfarin sodium 4 MG Oral Tablet [Marfarin]	Vitamin K Antagonist
1050260	RxNorm	855331	warfarin sodium 5 MG	Vitamin K Antagonist
1050261	RxNorm	855332	warfarin sodium 5 MG Oral Tablet	Vitamin K Antagonist
1050262	RxNorm	855333	warfarin sodium 5 MG [Coumadin]	Vitamin K Antagonist
1050263	RxNorm	855334	warfarin sodium 5 MG Oral Tablet [Coumadin]	Vitamin K Antagonist

1050264	RxNorm	855335	warfarin sodium 5 MG [Jantoven]	Vitamin K Antagonist
1050265	RxNorm	855336	warfarin sodium 5 MG Oral Tablet [Jantoven]	Vitamin K Antagonist
1050266	RxNorm	855337	warfarin sodium 6 MG	Vitamin K Antagonist
1050267	RxNorm	855338	warfarin sodium 6 MG Oral Tablet	Vitamin K Antagonist
1050268	RxNorm	855339	warfarin sodium 6 MG [Coumadin]	Vitamin K Antagonist
1050269	RxNorm	855340	warfarin sodium 6 MG Oral Tablet [Coumadin]	Vitamin K Antagonist
1050270	RxNorm	855341	warfarin sodium 6 MG [Jantoven]	Vitamin K Antagonist
1050271	RxNorm	855342	warfarin sodium 6 MG Oral Tablet [Jantoven]	Vitamin K Antagonist
1050272	RxNorm	855343	warfarin sodium 7.5 MG	Vitamin K Antagonist
1050273	RxNorm	855344	warfarin sodium 7.5 MG Oral Tablet	Vitamin K Antagonist
1050274	RxNorm	855345	warfarin sodium 7.5 MG [Coumadin]	Vitamin K Antagonist
1050275	RxNorm	855346	warfarin sodium 7.5 MG Oral Tablet [Coumadin]	Vitamin K Antagonist
1050276	RxNorm	855347	warfarin sodium 7.5 MG [Jantoven]	Vitamin K Antagonist
1050277	RxNorm	855348	warfarin sodium 7.5 MG Oral Tablet [Jantoven]	Vitamin K Antagonist
1050278	RxNorm	855349	warfarin sodium 0.5 MG	Vitamin K Antagonist
1050344	RxNorm	855350	warfarin sodium 0.5 MG Oral Tablet	Vitamin K Antagonist
1059791	RxNorm	92279	warfarin Injectable Solution [Coumadin]	Vitamin K Antagonist
1070413	RxNorm	313735	Warfarin 2 MG Oral Tablet	Vitamin K Antagonist
1070414	RxNorm	313737	Warfarin 2 MG/ML Injectable Solution	Vitamin K Antagonist
1070523	RxNorm	313739	Warfarin 7.5 MG Oral Tablet	Vitamin K Antagonist
1070770	RxNorm	314279	Warfarin 5 MG Oral Tablet	Vitamin K Antagonist
1070824	RxNorm	313734	Warfarin 2.5 MG Oral Tablet	Vitamin K Antagonist
1070908	RxNorm	313732	Warfarin 10 MG Oral Tablet	Vitamin K Antagonist
1071007	RxNorm	342939	Warfarin 2 MG/ML	Vitamin K Antagonist
1071178	RxNorm	313738	Warfarin 6 MG Oral Tablet	Vitamin K Antagonist
1071202	RxNorm	198349	Warfarin 4 MG Oral Tablet	Vitamin K Antagonist
1071221	RxNorm	212123	Warfarin 3 MG Oral Tablet [Coumadin]	Vitamin K Antagonist
1071490	RxNorm	328835	Warfarin 10 MG	Vitamin K Antagonist
1071783	RxNorm	328834	Warfarin 4 MG	Vitamin K Antagonist
1071811	RxNorm	328832	Warfarin 2.5 MG	Vitamin K Antagonist
1072884	RxNorm	212124	Warfarin 6 MG Oral Tablet [Coumadin]	Vitamin K Antagonist
1072885	RxNorm	328831	Warfarin 2 MG	Vitamin K Antagonist
1073224	RxNorm	313733	Warfarin 1 MG Oral Tablet	Vitamin K Antagonist

1080366	RxNorm	209086	Warfarin 4 MG Oral Tablet [Coumadin]	Vitamin K Antagonist
1080367	RxNorm	209088	Warfarin 10 MG Oral Tablet [Coumadin]	Vitamin K Antagonist
1081166	RxNorm	209083	Warfarin 2 MG Oral Tablet [Coumadin]	Vitamin K Antagonist
1081547	RxNorm	389189	Warfarin 0.5 MG Oral Tablet	Vitamin K Antagonist
1081848	RxNorm	209087	Warfarin 7.5 MG Oral Tablet [Coumadin]	Vitamin K Antagonist
1081874	RxNorm	209081	Warfarin 5 MG Oral Tablet [Coumadin]	Vitamin K Antagonist
1082096	RxNorm	209080	Warfarin 2 MG/ML Injectable Solution [Coumadin]	Vitamin K Antagonist
1082242	RxNorm	209082	Warfarin 1 MG Oral Tablet [Coumadin]	Vitamin K Antagonist
1082243	RxNorm	209084	Warfarin 2.5 MG Oral Tablet [Coumadin]	Vitamin K Antagonist
1082442	RxNorm	393451	Warfarin 0.5 MG	Vitamin K Antagonist
1083057	RxNorm	404260	Warfarin 6 MG Oral Tablet [Jantoven]	Vitamin K Antagonist
1083283	RxNorm	569713	Warfarin 1 MG [Coumadin]	Vitamin K Antagonist
1083394	RxNorm	539952	Warfarin 10 MG [Coumadin]	Vitamin K Antagonist
1083395	RxNorm	572426	Warfarin 3 MG [Coumadin]	Vitamin K Antagonist
1083421	RxNorm	569712	Warfarin 5 MG [Coumadin]	Vitamin K Antagonist
1083422	RxNorm	569718	Warfarin 7.5 MG [Coumadin]	Vitamin K Antagonist
1083468	RxNorm	569711	Warfarin 2 MG/ML [Coumadin]	Vitamin K Antagonist
1083469	RxNorm	569715	Warfarin 2.5 MG [Coumadin]	Vitamin K Antagonist
1083484	RxNorm	404147	Warfarin 7.5 MG Oral Tablet [Jantoven]	Vitamin K Antagonist
1083495	RxNorm	404144	Warfarin 2.5 MG Oral Tablet [Jantoven]	Vitamin K Antagonist
1083620	RxNorm	404141	Warfarin 5 MG Oral Tablet [Jantoven]	Vitamin K Antagonist
1083621	RxNorm	404142	Warfarin 1 MG Oral Tablet [Jantoven]	Vitamin K Antagonist
1083797	RxNorm	569714	Warfarin 2 MG [Coumadin]	Vitamin K Antagonist
1083924	RxNorm	404148	Warfarin 10 MG Oral Tablet [Jantoven]	Vitamin K Antagonist
1084192	RxNorm	576209	Warfarin 5 MG [Jantoven]	Vitamin K Antagonist
1084549	RxNorm	576210	Warfarin 1 MG [Jantoven]	Vitamin K Antagonist
1084839	RxNorm	404143	Warfarin 2 MG Oral Tablet [Jantoven]	Vitamin K Antagonist
1084845	RxNorm	404259	Warfarin 3 MG Oral Tablet [Jantoven]	Vitamin K Antagonist
1085158	RxNorm	569717	Warfarin 4 MG [Coumadin]	Vitamin K Antagonist
1085159	RxNorm	572427	Warfarin 6 MG [Coumadin]	Vitamin K Antagonist
1085193	RxNorm	404146	Warfarin 4 MG Oral Tablet [Jantoven]	Vitamin K Antagonist
1090456	RxNorm	576211	Warfarin 2 MG [Jantoven]	Vitamin K Antagonist
1090457	RxNorm	576286	Warfarin 6 MG [Jantoven]	Vitamin K Antagonist
1090718	RxNorm	576212	Warfarin 2.5 MG [Jantoven]	Vitamin K Antagonist

1090786	RxNorm	576214	Warfarin 4 MG [Jantoven]	Vitamin K Antagonist
1090787	RxNorm	576215	Warfarin 7.5 MG [Jantoven]	Vitamin K Antagonist
1090822	RxNorm	576285	Warfarin 3 MG [Jantoven]	Vitamin K Antagonist
1093150	RxNorm	576216	Warfarin 10 MG [Jantoven]	Vitamin K Antagonist
1098824	RxNorm	314280	Warfarin 3 MG Oral Tablet	Vitamin K Antagonist
1101417	RxNorm	328830	Warfarin 1 MG	Vitamin K Antagonist
1103038	RxNorm	329201	Warfarin 6 MG	Vitamin K Antagonist
1104375	RxNorm	329200	Warfarin 3 MG	Vitamin K Antagonist
1104610	RxNorm	330536	Warfarin 5 MG	Vitamin K Antagonist
1104963	RxNorm	540211	Warfarin 10 MG [Warfin]	Vitamin K Antagonist
1105145	RxNorm	540217	Warfarin 2 MG Oral Tablet [Warfaren]	Vitamin K Antagonist
1105385	RxNorm	540206	Warfarin 1 MG [Narfarin]	Vitamin K Antagonist
1105386	RxNorm	540215	Warfarin 2 MG [Warfaren]	Vitamin K Antagonist
1105557	RxNorm	540229	Warfarin 4 MG Oral Tablet [Marfarin]	Vitamin K Antagonist
1106085	RxNorm	540227	Warfarin 4 MG [Marfarin]	Vitamin K Antagonist
1106121	RxNorm	540208	Warfarin 1 MG Oral Tablet [Narfarin]	Vitamin K Antagonist
1107419	RxNorm	330537	Warfarin 7.5 MG	Vitamin K Antagonist
1108799	RxNorm	540213	Warfarin 10 MG Oral Tablet [Warfin]	Vitamin K Antagonist
1142571	RxNorm	313736	Warfarin 50 MG Injectable Solution	Vitamin K Antagonist
1146731	RxNorm	540216	Warfarin Oral Tablet [Warfaren]	Vitamin K Antagonist
1146781	RxNorm	540212	Warfarin Oral Tablet [Warfin]	Vitamin K Antagonist
1147230	RxNorm	324013	Warfarin 50 MG	Vitamin K Antagonist
1149831	RxNorm	644655	Warfarin Oral Tablet [Warfaring]	Vitamin K Antagonist
1150633	RxNorm	644656	Warfarin 10 MG Oral Tablet [Warfaring]	Vitamin K Antagonist
1155126	RxNorm	644654	Warfarin 10 MG [Warfaring]	Vitamin K Antagonist
958832	RxNorm	202421	Coumadin	Vitamin K Antagonist
999598	RxNorm	368417	warfarin Oral Tablet [Coumadin]	Vitamin K Antagonist
1001478	RxNorm	374319	warfarin Oral Tablet	Vitamin K Antagonist
1001479	RxNorm	374320	warfarin Injectable Solution	Vitamin K Antagonist
